# The association of ICUC trauma score and quick DASH in a distal radius fracture cohort

**DOI:** 10.1186/s13018-024-04623-0

**Published:** 2024-02-15

**Authors:** Wen-Chih Liu, Magdalena Hartwich, Joseph J. Locascio, Pietro Regazzoni, Jesse B. Jupiter, Alberto Fernandez Dell’Oca

**Affiliations:** 1 Kaohsiung Medical University Hospital, School of Medicine, College of Medicine, Kaohsiung Medical University, Kaohsiung, Taiwan; 2 Hand and Arm Center, Department of Orthopedics, Massachusetts General Hospital, Harvard Medical School, Boston, USA; 3 Department of Orthopedics, Hospital Britanico Montevideo, Montevideo, Uruguay; 4 Orthopedics Specialization, Universidad de Montevideo, Montevideo, Uruguay; 5 Biostatistics Center, Division of Clinical Research, Department of Medicine, Massachusetts General Hospital, Harvard Medical School, Boston, MA USA; 6 University Hospital of Basel, Basel, Switzerland

**Keywords:** Quick DASH, Patient-reported outcomes, ICUC trauma score, Distal radius fracture

## Abstract

**Background:**

This study evaluates the association between ICUC trauma and short-form Disabilities of the Arm, Shoulder, and Hand Questionnaire (Quick DASH) scores among patients who underwent surgery for distal radius fractures.

**Methods:**

This research gathered patient-reported outcomes (PROs) from patients registered in the ICUC database at a single trauma center. The study involved 76 adult patients who underwent surgical treatment for distal radius fractures before 2023. These patients received a volar locking plate for their distal radius fracture. The research utilized two different PROs to evaluate the patients’ conditions. The ICUC trauma score measures functional impairment and pain through two 5-point scale questions, allowing patients to self-assess these aspects. The Quick DASH, comprising 11 questions, was used to evaluate symptoms and functionality of the upper extremity.

**Results:**

For patients aged 55.9 ± 15.3 years and 4.6 ± 3.9 years post-op follow-up, the ICUC trauma score was 0.70 ± 0.95, and Quick DASH was 6.07 ± 10.35. A strong correlation between ICUC and Quick DASH was identified (*r* = 0.71, *P *< 0.01). The interaction between the ICUC trauma score and age at the surgery to Quick DASH revealed a significant unstandardized partial regression coefficient of 0.19 (95% confidence interval 0.08–0.31; *P *< 0.01).

**Conclusion:**

This study demonstrated a strong correlation between the ICUC trauma score and the Quick DASH among patients, especially the elderly. It was noted that an elevation in the ICUC trauma score is linked to a more marked increase in the Quick DASH score, particularly in older patients. Given its simplicity and efficacy, the ICUC trauma score may be a viable alternative to the Quick DASH for assessing the patient’s clinical outcomes.

**Supplementary Information:**

The online version contains supplementary material available at 10.1186/s13018-024-04623-0.

## Introduction

Despite still limited use, patient-related outcomes (PROs) should become the new standard. It would be a logical consequence of the recent changes in the relationship between patients and their doctors. Increasingly Involved in surgical decision-making, the patients should also decide how to judge the result of a treatment [1]. The parameters for the judgment might be very different for patients and doctors. With PROs, patient autonomy and preferences are prioritized over doctor’s potentially paternalistic convictions.

The development of PROs initially involved using standardized measures to assess a person’s general health and quality of life [2]. Rand’s health insurance experiment is one of the most widely used studies to evaluate the effects of different health insurance plans on healthcare outcomes and costs [3]. This research initiative subsequently led to the development of the SF-36 health survey by Ware [4], which has been widely employed across various medical specialties.

In orthopedics, assessments previously relied on radiographic features (such as union, alignment, and arthritis) and physical examinations (including range of motion and joint stability). Since the 1990s, more and more research has emphasized PROs, which gather patient feedback. These PROs include various domain-specific, condition-specific, and anatomic region-specific assessments. Proper utilization of PROs for evaluating a patient’s functional status before surgery and assessing their recovery afterward can provide insights into the benefits patients can expect and their “patient acceptable symptom state” [1].

As an example of an upper extremity-specific PRO, the American Academy of Orthopaedic Surgeons, the Institute for Work and Health, and the Council of Musculoskeletal Specialty Societies developed the Disabilities of the Arm, Shoulder, and Hand (DASH) questionnaire. The DASH questionnaire comprises 30 items and is widely used to assess upper extremity symptoms and function [5]. Subsequently, a concept-retention version known as the short-form DASH (Quick DASH) questionnaire with only 11 items was developed, and its validity and reliability have been confirmed through numerous studies, making it a potential substitute for the full version of DASH [6, 7]. The AAOS recommended DASH and Quick DASH scores to evaluate patients with hand, wrist, and elbow injuries and pathologies [8, 9]. Various PROs specific to the hand, wrist, elbow, and shoulder have also been developed [10–12].

Assessing outcomes at 6 months or longer post-surgery is inherently challenging [13]. However, the increasing number of questionnaires that patients are required to complete affects patient satisfaction with healthcare and impacts the overall workload of healthcare providers. In addition to the Quick DASH questionnaire, there are simplified versions of other assessments, such as the Patient-Health Questionnaire-2 (PHQ-2) for assessing patient mental health, replacing the PHQ-9 [14], and the Pain Self-Efficacy Questionnaire-2 (PSEQ-2) to evaluate the psychometric effects of pain, using only 2 items instead of the original 10 [15]. A meta-analysis study found that as the number and length of questionnaire items increase, the response burden also affects response rates [16]. Therefore, effective and concise PROs offer significant benefits in evaluating a patient’s postoperative condition.

A reason for the still meager use of PROs might be the wrong perception that they are time-consuming. The search for improvements is, therefore, legitimate. Consequently, in assessing patient satisfaction with surgery, more studies are finding that the Single-Assessment Numeric Evaluation (SANE) score can effectively gauge postoperative satisfaction and correlates highly with various legacy PROs [17–19]. The function of the injured limb segment and pain or other subjective derangements are the essential elements to be analyzed. Using a single question to assess functional limitation and pain can make the PRO evaluation process more efficient, but it is uncertain if it is as effective as legacy PROs with numerous questions.

The ICUC trauma score is a concise PRO that allows patients to self-assess the impact of postoperative trauma on that specific joint function recovery and pain. Functional limitation and pain are assessed, each using a 5-point scale for patient self-assessment. This study evaluated a cohort of patients enrolled in the ICUC registry database with distal radius fractures, collecting both Quick DASH and ICUC trauma scores. The aim was to determine the correlation between the Quick DASH score and ICUC trauma score in assessing patients who underwent volar locking plate surgery for distal radius fractures. The hypothesis was that the ICUC trauma score would have a weak correlation with the Quick DASH score at different follow-up time points.

## Methods

### Patients

In this study, we evaluated a prospective cohort of patients with distal radius fractures treated surgically enrolled in the ICUC registry database [20]. All participants in the study provided written informed consent, authorizing the use of their data and images within the ICUC registry database for research and publication purposes. This study was approved by the Institutional Review Board of a university-affiliated hospital (KMUHIRB-E(I)-20230185) to analyze the ICUC registry database. We included 1. patients who had unstable distal radius fracture, which is surgically indicated [21, 22], and 2. patients who were 18 years or older when undergoing surgery for a distal radius fracture. However, those under 18 or those presenting with simultaneous injuries or conditions in the same upper limb were precluded from participation.

### Surgical treatment

Patients with unstable distal radius fractures were treated surgically with a volar locking plate by board-certified orthopedic surgeons at one ICUC center in Montevideo, Uruguay. Preoperative radiographs, intraoperative images, and serial postoperative radiographs were uploaded to the ICUC database. The postoperative images of the wrist and forearm range of motion were presented.

### Quick DASH and ICUC trauma scores

Patients were evaluated during their follow-up visits by surgeons who performed the surgery. The ICUC trauma score assesses the degree of functional limitations in patients compared to their pre-fracture functional state and their level of pain, utilizing a 5-point scale for each. These scores are then summed to create a scale ranging from 0 to 8 points. The assessment of functional limitation and pain scale are presented in Table 1.

**Table 1:** ICUC trauma score

Functional limitation	Pain scale
0, No limitation The patient can perform all activities as they could before the fracture	0, No pain
1, Can do most activities There is some limitation in joint motion and slight functional impairment	1, Mild pain
2, Can only do certain activities There is a clear limitation in joint motion and marked functional impairment	2, Moderate pain
3, Unable to do most activities The patient has a poor range of motion	3, Intense pain
4, Unable to do any activity The joint is stiff, and the patient cannot perform any activities	4, Worst possible pain

The Spanish version of the Quick DASH questionnaire was provided to be circulated by the patients [23]. The Quick DASH consists of 11 items, including 8 related to functions and 3 related to symptoms, with each item scored on a scale from 1 to 5 (Table 2). The Quick DASH score is computed, where a score of zero indicates the worst upper extremity condition and a score of 100 represents the best upper extremity condition.

**Table 2:** Quick disabilities of the arm, shoulder, and hand score

Please rate your ability to do the following activities in the last week:	
Open a tight or new jar	
Do heavy household chores (e.g., wash walls, wash floors)	
Carry a shopping bag or briefcase	
Wash your back	
Use a knife to cut food	
Recreational activities in which you take some force or impact through your arm, shoulder or hand (e.g., golf, hammering, tennis, etc.)	
No difficulty Mild difficulty Moderate difficulty	Severe difficulty Unable
During the past week, to what extent has your arm, shoulder, or hand problem interfered with your normal social activities with family, friends, neighbors, or groups?	
Not at all Slightly Moderately	Quite a bit Extremely
During the past week, were you limited in your work or other regular daily activities as a result of your arm, shoulder, or hand problem?	
Not limited Slightly limited Moderately limited	Very limited Unable
In the last week, please rate the severity of arm, shoulder, or hand pain In the last week, please rate the severity of tingling (pins and needles) in your arm, shoulder, or hand	
None Mild Moderate	Severe Extreme
During the past week, how much difficulty have you had sleeping because of the pain in your arm, shoulder, or hand?	
No difficulty Mild difficulty Moderate Difficulty	Severe difficulty Cannot sleep

Two independent operators checked the ICUC trauma score for the same patients at the follow-up visit. The ICUC trauma and Quick DASH scores were evaluated at the same postoperative visit.

### Statistical analysis

To describe the baseline characteristics, for each variable, we either reported the mean ± standard deviation or a proportion. We utilized Pearson correlation coefficients (*r*) and its 95% confidence interval (CI) to assess the relationships between the ICUC trauma and Quick DASH scores. The strength of these correlations was interpreted as follows: very strong correlation (*r* = 0.90–1.00), strong (*r* = 0.70–0.89), moderate (*r* = 0.40–0.69), weak (*r* = 0.10–0.39), or negligible (*r* = 0.00–0.09) [24]. In our power analysis for a one-sample correlation test using Fisher’s z test, we aimed to investigate whether a specified target validity correlation coefficient of *r*_*a*_ = 0.7, (strong correlation) is greater than a specified “null” value of *r*_0_ = 0.4, (weak correlation). The latter was used instead of the conventional null correlation of *r*_0_ = 0 because we consider a validity correlation < 0.4 as effectively of no validity. We set our significance level at 0.05 and desired statistical power of 0.80. The minimum required sample size estimated for these specifications is 35. The Pearson correlation between ICUC trauma score and Quick DASH score was computed for subgroups by patient age, sex, involvement wrist, and follow-up length. A general linear model (GLM) with backward elimination was performed with the dependent variable quick DASH and an initial predictor set of ICUC trauma score, sex, age at surgery (linear and quadratic terms), and the interaction of ICUC trauma score with sex and age at surgery (linear, quadratic). Residuals were checked for conformance to normality assumptions. Statistical analyses were performed using STATA 17.1 (StataCorp, College Station, TX) and SAS 9.4 (SAS Institute, Cary, NC). *P *< 0.05 was considered statistically significant.

## Results

### Study cohort

A total of 80 patients met the inclusion criteria for this study. We excluded three patients who presented with symptomatic shoulder rotator cuff arthropathy and one patient with ipsilateral hemiplegia. Patient characteristics and fracture patterns are presented in Table 3 and  Additional file 1 :Supplemental Digital Content 1. The mean ICUC trauma and Quick DASH scores were 0.70 ± 0.95 points (median, 0; range 0–4) and 6.07 ± 10.35 points (median, 2.1; range 0–60).

**Table 3:** Characteristics of the study cohort

Age (years)	55.9 ± 15.3
Sex	
Male	23 (30.3%)
Female	53 (69.7%)
Wrist	
Right	29 (38.2%)
Left	47 (61.8%)
Follow-up period (years)	4.6 ± 3.9
≤ 6 months	7 (9.2%)
6–12 months	12 (15.8%)
12–24 months	12 (15.8%)
> 24 months	45 (59.2%)
Quick DASH score	6.07 ± 10.35
ICUC trauma score	0.70 ± 0.95

*Quick DASH, Short-form Disabilities of the Arm, Shoulder, and Hand (DASH) questionnaire; mean ± standard deviation

### Score correlations

In the comprehensive assessment, a strong correlation was observed between the ICUC trauma index and the Quick DASH scores (*r* = 0.71, *n *= 76, 95% CI 0.58–0.81, *P *< 0.01). Subgroup stratifications showed varying degrees of association. At the 6-month follow-up, there was a moderate association (*r *= 0.62, *n *= 7, 95% CI − 0.25 to 0.94, *P *= 0.14), between 6 and 12 months postoperatively, a moderate association (*r* = 0.66, *n *= 12, 95% CI 0.14–0.89, *P *= 0.02), between 12 and 24 months postoperatively, a very strong association (*r* = 0.94, *n *= 12, 95% CI 0.79–0.98, *P *< 0.01), and beyond 24 months postoperatively, a moderate association (*r* = 0.68, *n *= 45, 95% CI 0.49–0.81, *P *< 0.01). When segmented by age, patients aged below 50 years displayed a moderate association (*r* = 0.61, *n *= 22, 95% CI 0.26–0.82, *P *< 0.01). Patients within the age range of 50–70 years showed a strong association (*r* = 0.72, *n *= 36, 95% CI 0.44–0.87, *P *< 0.01). Patients aged above 70 years exhibited a very strong association (*r *= 0.90, *n *= 18, 95% CI 0.74–0.96, *P *< 0.01). This stratified analysis elucidates the varying strength of associations between the ICUC trauma and Quick DASH scores across different follow-up durations and age groups.

### General linear model

**Fig. 1:**
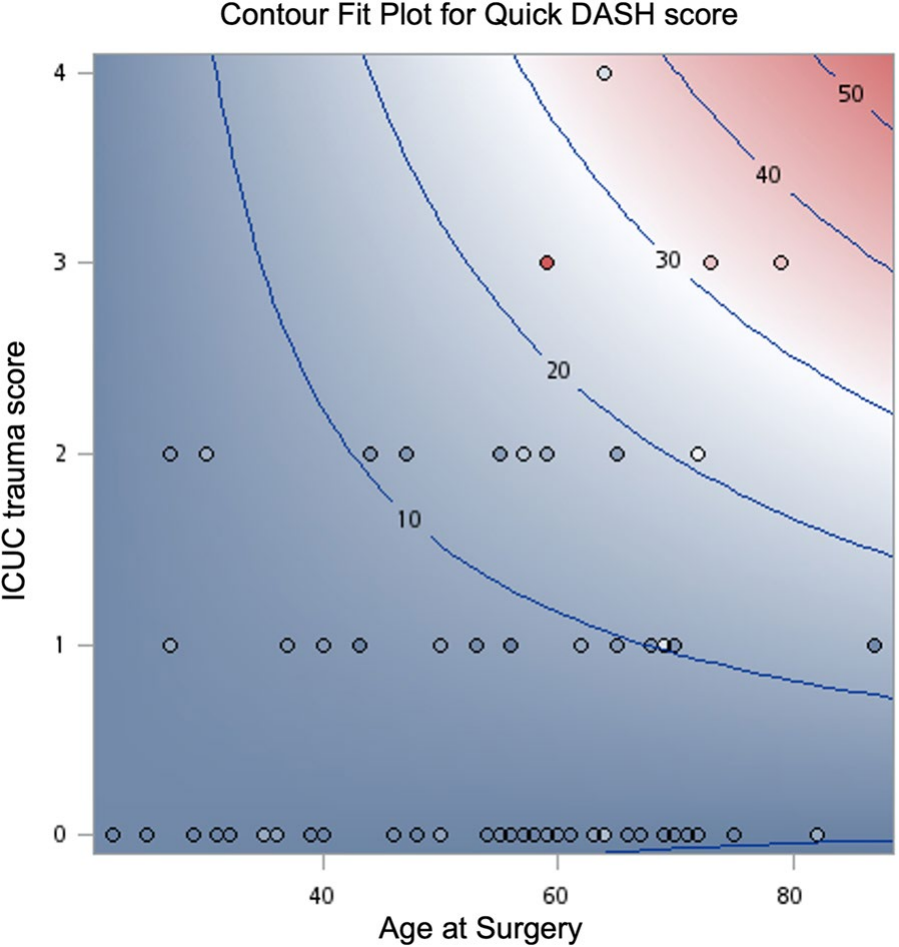
The contour fit plot provides a visual representation of the relationship between ICUC trauma score and age at surgery versus short-form Disability of Arm, Shoulder, and Hand (Quick DASH) scores. Each contour line represents points of equal Quick DASH score

**Fig. 2:**
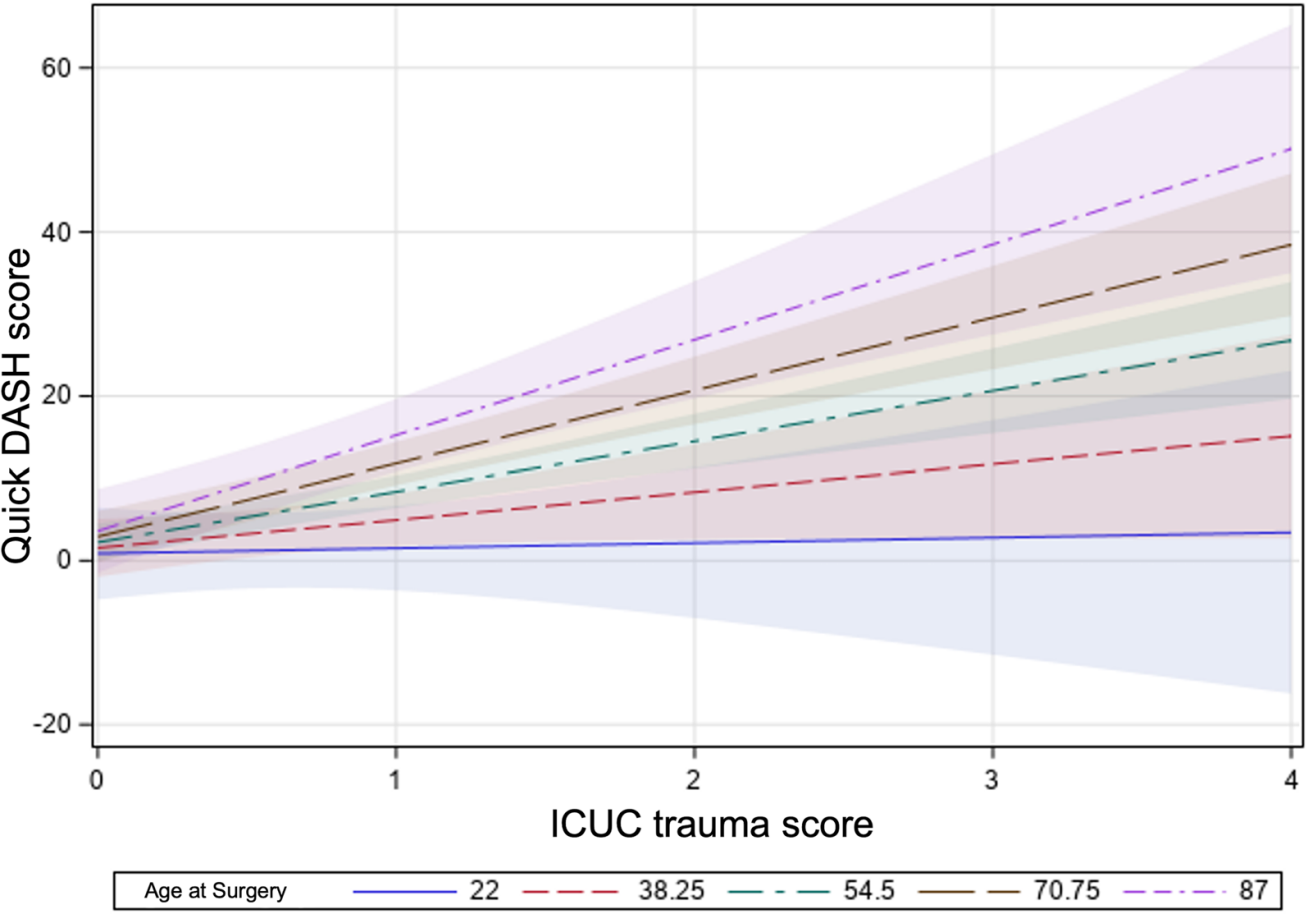
The effect plot portrays the interaction of ICUC trauma score and age at the surgery by plotting model-predicted values for short-form Disability of Arm, Shoulder, and Hand (Quick DASH) versus ICUC trauma scores at the different representative strata of ages at the surgery

Additional file 2:The supplemental digital content 2 shows the GLM using backward elimination to determine the best model. We also verified that the residuals met the assumptions of normality. The interaction between ICUC trauma score and age at the surgery yielded a significant unstandardized partial regression coefficient (*β*) of 0.194 (*t* value = 3.37; degrees of freedom, 72; 95% CI, 0.08–0.31; *P *< 0.01). Thus, older people show a stronger positive relation between ICUC trauma score and Quick DASH scores than younger people. This underscores a modifying effect of age at the surgery on the relationship between ICUC trauma score and Quick DASH score. Figures 1 and 2 illustrate the contour fit plot and effect plot for Quick DASH, respectively.

## Discussions

In orthopedic traumatology, PROs are used preoperatively to evaluate the functional impairment caused by the injury and postoperatively to assess the efficacy of the treatment [25]. However, in cases of acute orthopedic trauma, it is often challenging to accurately assess the patient’s functional status due to limb deformity and pain. Furthermore, using tools like DASH or Quick DASH, which are frequently employed to evaluate upper limb function, patients often find it difficult to precisely determine whether their inability to perform certain activities is due to functional limitations arising from the wrist, elbow, or shoulder joint due to disease or injury. In our study, we concurrently assessed 76 patients who underwent distal radius fracture volar plating surgery. We found that using the ICUC trauma score for patients to self-assess joint function compared to their pre-injury status and pain scale and summing these two scores showed a strong correlation (*r* = 0.71, *n *= 76, 95% CI 0.58–0.81, *P *< 0.01) with the Quick DASH score. After performing a subgroup analysis based on postoperative follow-up period and age at the time of surgery, we found that, in every subgroup, the ICUC trauma score had a moderate to very strong correlation with the Quick DASH score. Through the GLM analysis, we observed that when the age at surgery and the value of the ICUC trauma score increased, the Quick DASH score exhibited a differential response compared to considering each factor individually. Specifically, older individuals displayed a stronger positive relation of ICUC to Quick DASH than younger individuals.

There are notable distinctions between the ICUC trauma score and the Quick DASH score. Firstly, Quick DASH measures functional limitations by contrasting them with an ideal scenario without limitations. In contrast, the ICUC score compares present functional limitations to those existing before the fracture. Secondly, Quick DASH evaluates combined shoulder, elbow, wrist, and hand limitations without distinguishing between left or right-hand injuries. Alternatively, the ICUC trauma score prompts patients to concentrate on the specific injured area, such as wrist restrictions in cases of distal radius fractures, with the intent of excluding limitations related to the shoulder, elbow, and hand. As a result, it is unsurprising that a perfect correlation was not found: Some patients with prior limitations might score worse on the Quick DASH score compared to the ICUC trauma score. Conversely, some patients with moderate functional limitations could achieve a high Quick DASH score but only an acceptable low ICUC score.

When we discussed PROs, we often had to consider many factors, such as age. Asheim et al. conducted a study on the normative values of DASH and Quick DASH using the general population of Norway [26]. They found that the DASH or Quick DASH score tends to increase at a greater rate as age increases. However, the 95% confidence interval also broadens, indicating significant variability among the older population. Therefore, if different groups use DASH or Quick DASH to evaluate treatment outcomes, the results might be biased. Compared to the Quick DASH score, the ICUC trauma score considers the pre-injury activity status, which already considers the potential impact of age. As a result, it does not show great differences with increasing age like the Quick DASH score does. The ICUC trauma score can effectively evaluate postoperative outcomes simply using two questions.

**Fig. 3:**
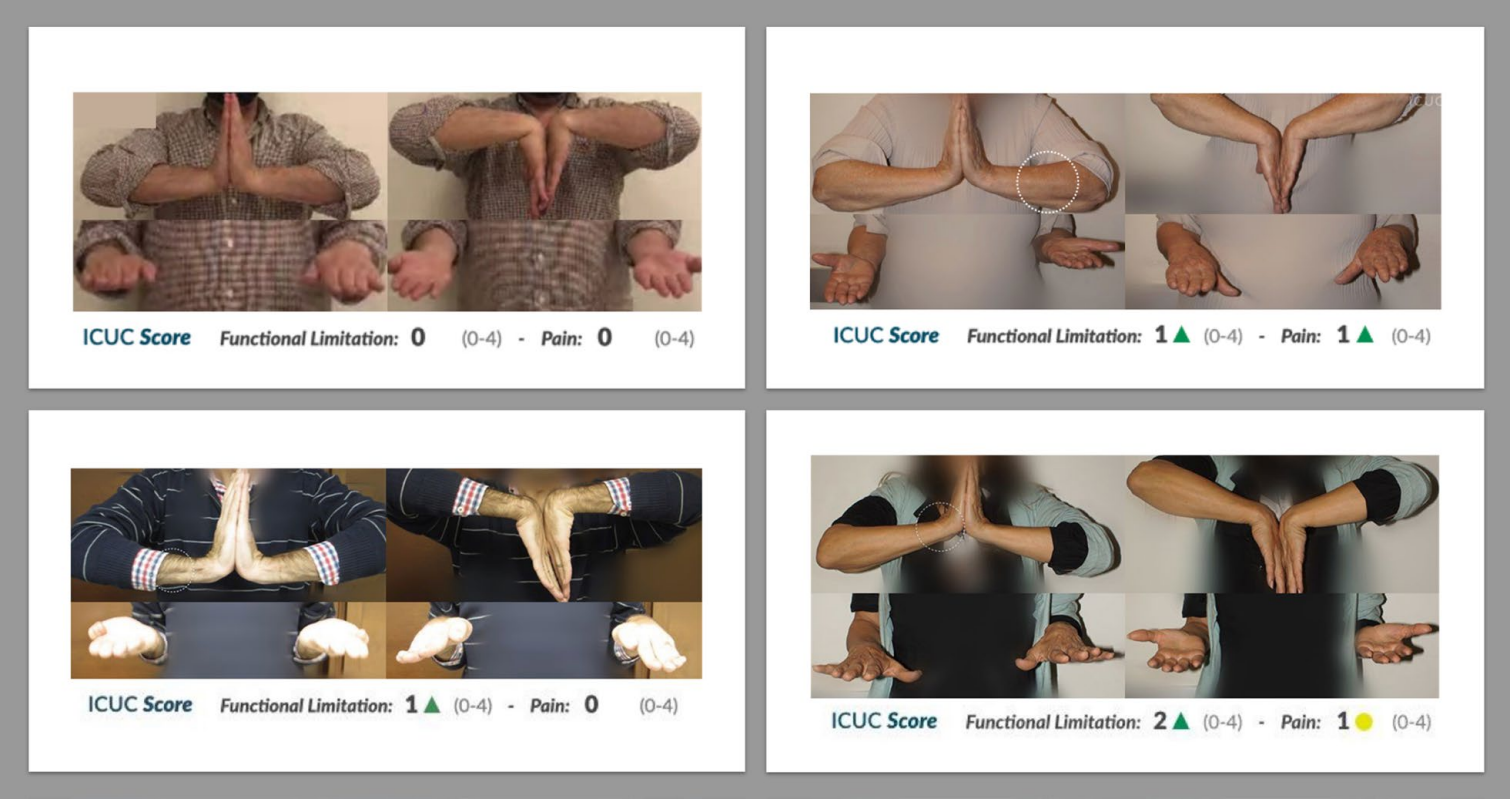
The figure illustrates the images of wrist motion—including extension, flexion, supination, and pronation—that are depicted in conjunction with the ICUC trauma scores, delineating functional limitations and associated pain scales

The essence of ICUC is “I See, You See.” Thus, within this publicly accessible online database, in addition to prospectively recording the patient’s ICUC trauma score, there are also image records of postoperative joint motion (Fig. 3). This ensures a rigorous and reliable evaluation of the surgical effects on the patients [20, 27, 28]. Contemporary medicine emphasizes evidence-based practice, aiming to provide sufficient information for shared decision-making between physicians or surgeons and patients [29]. Numerous PROs are available to assess a patient’s physical and physiological status. However, as the number of patient questions increases, there is a risk of survey fatigue [30]. Addressing this would require additional medical resources to record patient responses to all questions, which is not feasible in real-world settings. While the ICUC trauma score is not a comprehensive PRO, it offers a highly practical assessment method in clinical settings. Furthermore, our study confirms a strong correlation between the ICUC trauma score and the Quick DASH score, making it a suitable and validated PRO measurement for orthopedic upper limb fracture surgeries. We believe there is a strong correlation between the ICUC trauma score and the Quick DASH. This correlation is observed when patients recover to their preoperative status, even if many movements were initially challenging, such as reaching their back during a bath, or infrequently executed tasks like using a knife to cut items. Consequently, we utilize two straightforward questions to inquire about the patients’ self-reported outcomes regarding functional limitations and pain scale. This approach is analogous to using the SANE to evaluate patient satisfaction post-fracture treatment, which is generally correlated with the DASH score [17].

Lastly, this study has several limitations. First, in this study, the patient population was derived from postoperative cases with varying follow-up periods. Data were collected from only one postoperative follow-up for each patient. As a result, the study does not evaluate the variations in ICUC trauma scores over different follow-ups for the same patient, nor the correlation with changes in the Quick DASH score. Second, the cohort in this study comprises postoperative patients. Moreover, the surgeries were conducted by board-certified orthopedic surgeons at an ICUC center. Due to the high success rate of the surgeries, both the Quick DASH scores and ICUC trauma scores were notably low, with a mean Quick DASH score of only 6.1 and a mean ICUC trauma score of just 0.7. Although there is a strong correlation between the ICUC trauma score and the Quick DASH score, we did not have many cases with severely limited functionality. The ICUC trauma score could be applied to populations with fracture malunions or sports injuries preoperatively to assess its correlation with the Quick DASH score. Third, although this study recorded whether the affected hand was left or right, it did not document whether the patient’s injured hand was their dominant or non-dominant one. Nevertheless, the ICUC trauma score allowed patients to self-assess their wrists before and after the injury. Even though hand dominance might impact the Quick DASH score more than the ICUC trauma score, this study cannot draw such a conclusion. Finally, in this cohort, we excluded three patients with shoulder rotator cuff arthropathy and one with ipsilateral hemiplegia. Given their low numbers relative to the entire cohort, conducting a statistical analysis on them was not feasible, leading to their exclusion. If these four patients were included, the correlation between the ICUC trauma and Quick DASH scores would decrease.

## Conclusions

This study demonstrated a strong correlation between the ICUC trauma score and the Quick DASH score among patients of all age groups who underwent volar plating for distal radius fractures. It was noted that an elevation in the ICUC trauma score is linked to a more marked increase in the Quick DASH score, particularly in older patients. Given its simplicity and efficacy, the ICUC trauma score may be a viable alternative to the Quick DASH for assessing the patient’s clinical outcomes.

### Supplementary Information

**Additional  file 1. **Cohort of Distal Radius Fractures: An Image Gallery Showcasing Radiographic and Clinical Images, Accompanied by Patient-Reported Outcomes.

**Additional  file 2. **Sequential Outcome of General Linear Model Utilizing Backward Elimination to Assess Quick DASH Score Influenced by Age, Sex, and ICUC Trauma Score.

## Data Availability

The datasets used and/or analyzed during the current study are available from the corresponding author on reasonable request.

## Abbreviations

CI: Confidence interval
DASH: Disabilities of the Arm, Shoulder, and Hand
PROs: Patient-related outcomes
PHQ: Patient-health questionnaire
PSEQ: Pain self-efficacy questionnaire
SANE: Single-assessment numeric evaluation
