# Supplementary material for: The association of ICUC trauma score and quick DASH in a distal radius fracture cohort
Source: J Orthop Surg Res. 2024 Feb 15;19:141. doi: 10.1186/s13018-024-04623-0 (PMC10870621; doi:10.1186/s13018-024-04623-0)
Supplement: Supplementary file 1 — Additional file 1. Cohort of Distal Radius Fractures: An Image Gallery Showcasing Radiographic and Clinical Images, Accompanied by Patient-Reported Outcomes. [file 13018_2024_4623_MOESM1_ESM.pdf]

The Association of ICUC Trauma Score and Quick DASH in a Distal Radius Fracture Cohort

Supplemental Digital Content 1

Case	ICUC No.	Age_Surgery	Surgery_Date	Exam_Date	Age_Exam	Sex	Quick DASH	ICUC_1	ICUC_2	Limb	Shoulder Pathology
1	23-DC-544	56	7/2/13	5/9/23	66	F	0	0	0	L	
2	23-DC-707	46	15/7/11	5/9/23	58	F	0	0		L	0
3	23-DC-221	60	17/2/12	21/9/23	71	F	30	0		L	1
4	23-DC-256	70	2/2/12	5/9/23	81	F	11	0	0	L	0
5	23-DC-580	64	15/9/11	6/9/23	77	F	14	0	0	R	0
6	23-DC-577	46	4/2/14	5/9/23	55	M	2	0	0	R	0
7	23-DC-224	72	16/10/14	5/9/23	82	F	0	0	0	L	0
8	23-DC-263	35	28/7/14	4/9/23	44	M	9	0	0	R	0
9	23-DC-494	27	6/3/14	21/9/23	36	M	5	2		R	0
10	23-DC-100	32	16/12/14	6/9/23	41	M	0	0	0	L	0
11	23-DC-401	56	5/8/14	7/9/23	65	F	0	0	1	L	0
12	23-DC-208	58	14/7/14	6/9/23	67	F	0	0	0	R	0
13	23-DC-165	57	25/7/13	5/9/23	67	F	4.5	0	0	L	0
14	23-DC-373	53	20/7/15	6/9/23	61	M	4.5	0	1	L	0
15	23-DC-868	70	3/8/15	6/9/23	79	M	0	0	0	L	0
16	23-DC-617	47	24/11/15	12/9/18	50	M	5	0	1	L	0
17	23-DC-606	71	23/1/17	7/9/23	78	F	0	0	0	R	0
18	23-DC-239	75	23/1/17	7/9/23	82	F	16	0	0	L	1
19	23-DC-015	53	17/4/17	6/9/23	59	M	2	1		R	0
20	23-DC-770	72	9/6/17	30/5/18	73	F	0	0		L	0
21	23-DC-003	60	7/11/13	14/8/19	66	F	2.2	0	0	R	0
22	23-VC-192	65	12/4/19	14/8/19	65	F	4.5	0	0	L	0
23	23-VC-054	67	22/8/14	13/9/23	76	F	32	0		R	1
24	23-DC-241	69	15/2/19	21/9/23	73	F	0	0		R	0
25	23-DC-343	31	21/8/18	21/9/23	36	M	0	0		R	0
26	23-DC-404	75	4/7/19	7/9/23	79	F	0	0	0	R	0
27	23-DC-511	59	9/10/19	18/12/19	59	F	2.2	0		L	0
28	23-DC-659	25	30/4/19	21/9/23	29	M	0	0		L	0
29	23-DC-683 at 33w	55	24/7/18	29/5/19	56	M	6	1	1	R	0
30	23-DC-961	72	15/11/18	11/9/23	77	F	27	1	1	L	0
31	23-DC-055	65	10/11/22	7/9/23	66	M	2	1	0	L	0
32	23-DC-175	36	26/9/22	7/9/23	37	F	6.8	0	0	R	0
33	23-DC-599	22	9/11/21	7/9/23	25	M	0	0	0	L	0
34	23-DC-855	50	11/1/22	7/9/23	51	F	6.8	1	1	R	0
35	23-DC-507	70	3/12/21	6/9/23	72	M	4.5	1	1	R	0
36	23-VC-977	22	27/11/10	13/9/23	35	M	0	0		L	0
37	23-VC-635	56	24/4/13	31/10/18	61	F	0	0		L	0
38	23-VC-022	48	18/8/11	22/9/23	60	F	0	0		R	0
39	23-VC-189	61	30/5/13	14/9/23	71	F	0	0		L	0
40	23-VC-705	70	4/10/16	25/4/18	72	F	0	0		L	0

Case	ICUC No.	Age_Surgery	Surgery_Date	Exam_Date	Age_Exam	Sex	Quick DASH	ICUC_1	ICUC_2	Limb	Shoulder Pathology
41	23-VC-547	50	27/12/18	21/9/23	55	M	7	0		L	
42	23-VC-803	59	2/5/19	27/11/19	59	F	60	1	1	L	0
43	23-VC-881	65	9/10/17	5/9/18	66	F	6	1		L	0
44	23-VC-376	39	10/3/20	13/9/23	42	F	0	0		R	0
45	23-VC-761	82	13/8/20	14/9/23	85	F	9	0		L	0
46	23-VC-780	67	30/11/21	7/9/23	69	M	0	0		L	0
47	23-VC-183	75	20/1/22	13/9/23	76	F	0	0		L	0
48	23-VC-255	58	24/6/21	13/9/23	60	M	0	0		L	0
49	23-VC-646	62	13/5/21	14/9/23	64	F	9	1	0	L	0
50	23-DC-826	56	24/7/12	5/9/23	67	F	0	0		L	0
51	23-DC-654	40	18/4/12	7/9/23	51	M	5	0	1	R	0
52	23-DC-572	55	29/7/14	12/9/23	64	F	2	0		R	0
53	23-DC-675	66	10/2/15	12/9/23	74	F	0	0		L	0
54	23-DC-591	30	15/8/14	21/9/23	39	F	11	1	1	R	0
55	23-DC-310	63	14/10/16	6/9/23	70	F	7	0		R	0
56	23-DC-075	37	21/7/20	6/9/23	40	F	5	1	0	L	0
57	23-DC-358	61	2/9/20	7/9/23	64	F	0	0		R	0
58	23-DC-408	72	22/2/22	21/9/23	73	F	2	0		L	0
59	23-DC-453	59	12/8/21	14/9/23	61	M	11	1	1	R	0
60	23-DC-605	54	26/10/22	12/9/23	55	F	0	0		L	0
61	23-DC-623	69	4/8/21	22/9/23	71	F	23	1	0	L	0
62	23-DC-902	70	21/12/22	12/9/23	71	F	5	0	1	L	0
63	23-DI-711	54	17/7/17	30/1/19	56	M	0	0		R	0
64	23-DC-311	40	21/5/15	25/4/18	43	F	0	0		L	0
65	23-VE-821	73	16/5/17	31/10/18	74	F	40	3		L	0
66	23-DE-391	29	13/10/16	25/4/18	31	F	0	0		L	0
67	23-DE-955	79	29/11/10	12/12/18	87	F	0	0	0	L	0
68	23-DU-569	87	27/7/18	9/12/18	87	F	3	1	0	L	0
69	23-DS-041	59	28/9/17	21/3/18	60	F	3	0	0	R	0
70	23-DI-237	64	19/2/18	5/6/19	65	F	22	1	1	L	0
71	23-DE-123	64	21/3/18	13/11/18	64	F	22	2	2	R	0
72	23-DC-1027	27	16/1/19	25/9/19	27	M	8	1	0	L	0
73	23-DI-397	43	9/4/19	7/10/19	43	F	0	1		L	0
74	23-VE-887	44	15/5/19	11/12/19	44	F	6	1	1	R	0
75	23-DS-984	68	11/7/19	11/12/19	68	F	6	1		R	0
76	23-VB-864	57	26/7/19	18/12/19	45	F	17	1	1	L	0
77	23-DS-530	44	19/11/19	4/3/20	45	M	5	1	1	L	0
78	23-VC-121	70	4/4/22	13/9/23	71	F	5	1	1	L	0
79	23-VC-706	70	4/4/22	13/9/23	71	F	5	1	1	R	0
80	23-VC-474	57	8/12/20	5/9/23	60	F	0	0		L	0

ICUC® ID: 23-DC-544 / 55y Female

*Complete case data can be accessed by clicking the **ICUC ID***

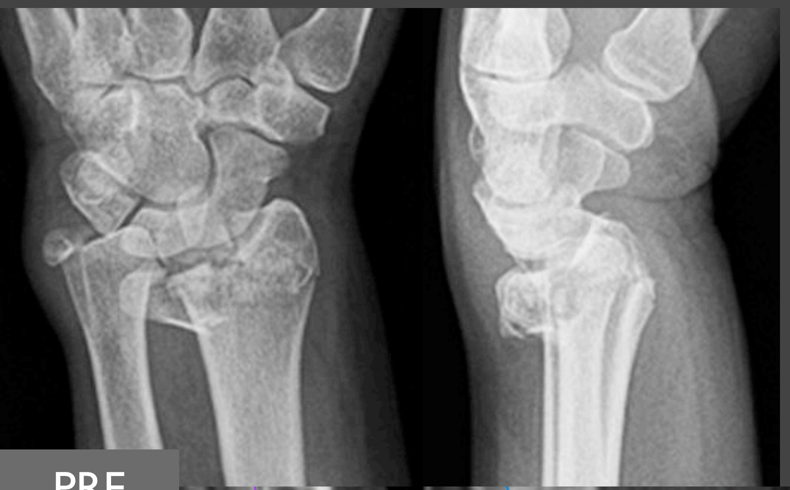

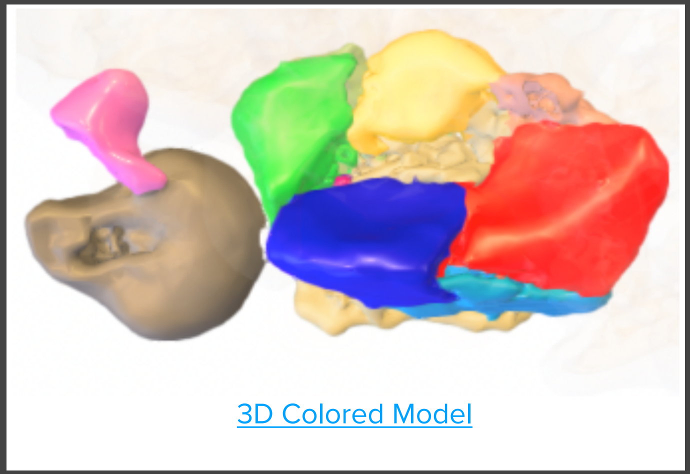

PRE

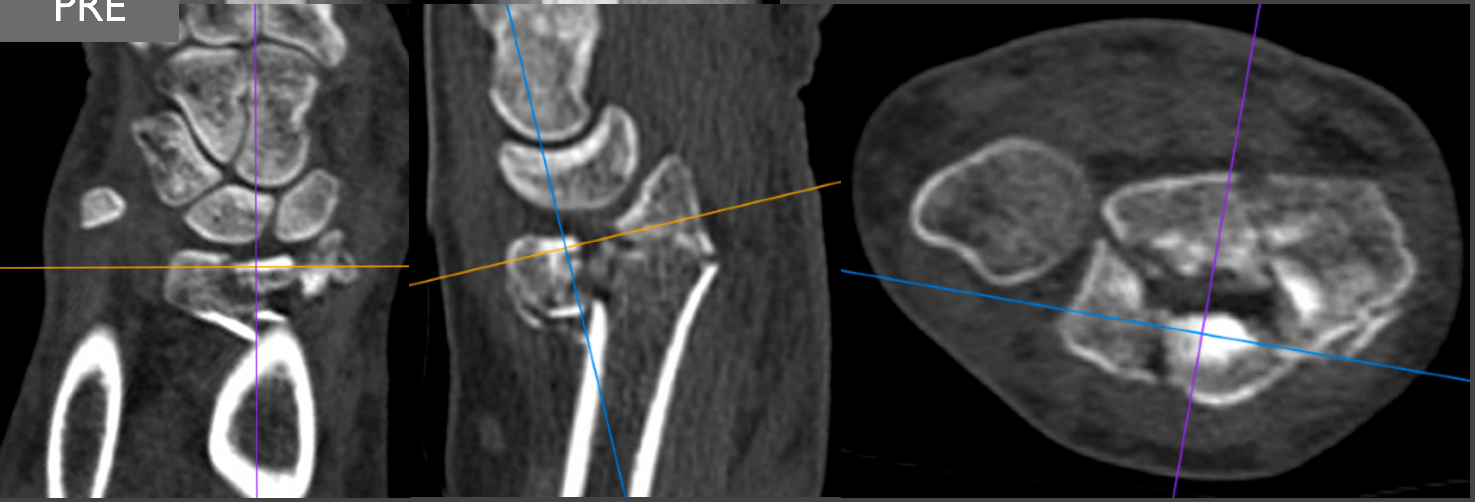

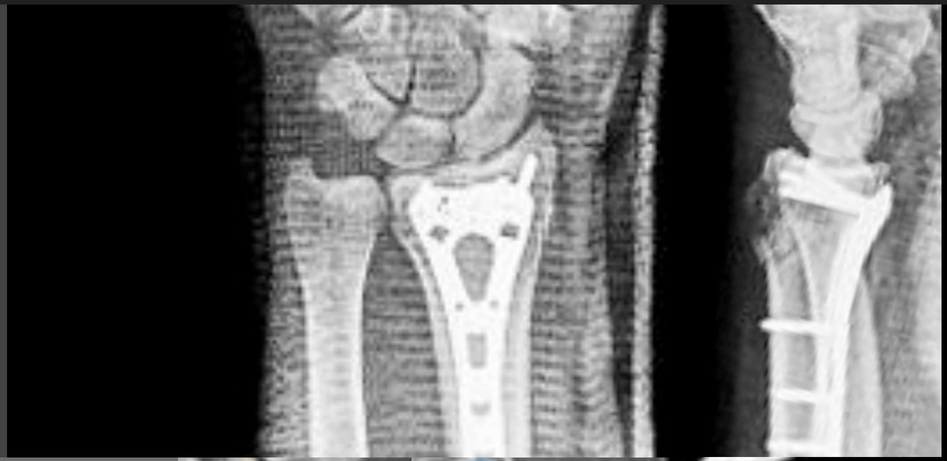

0w

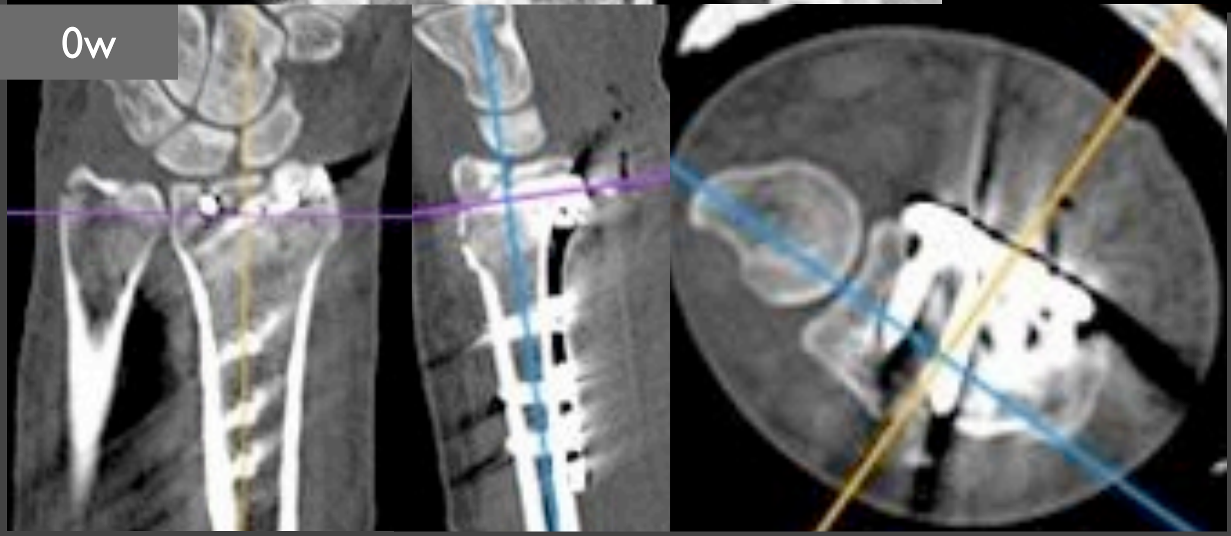

Follow-up

19w

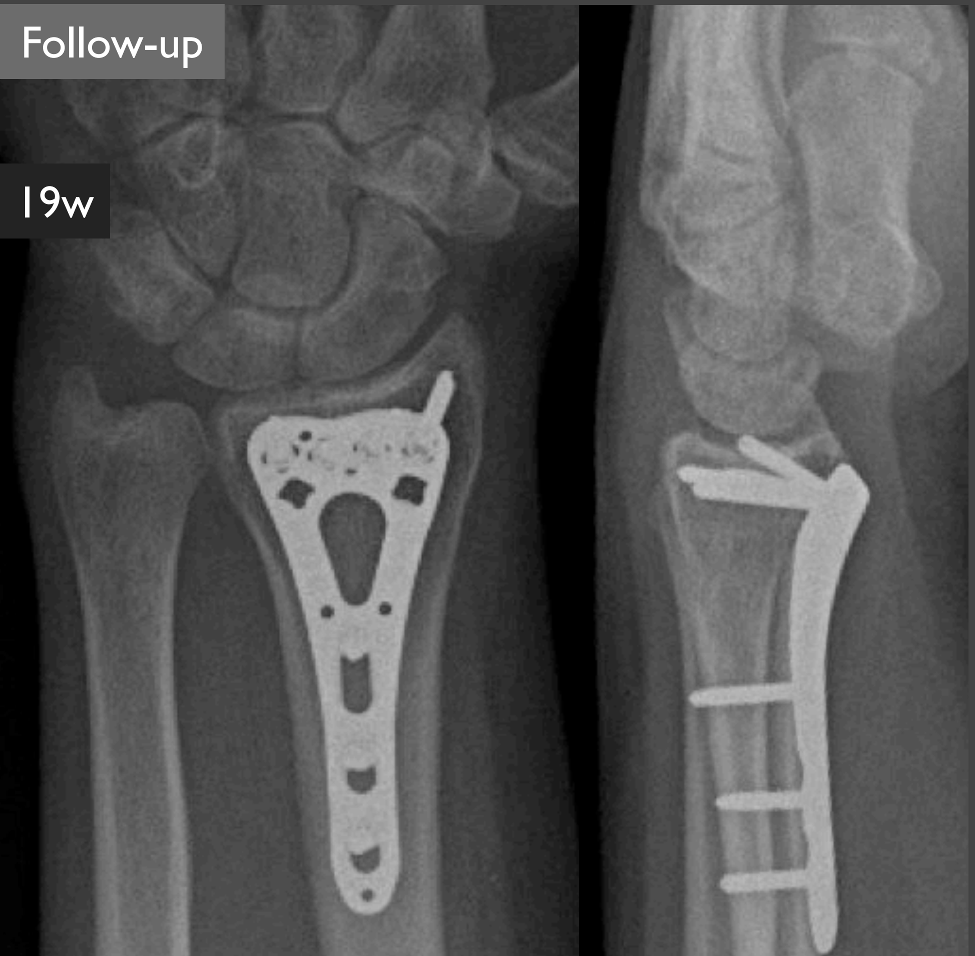

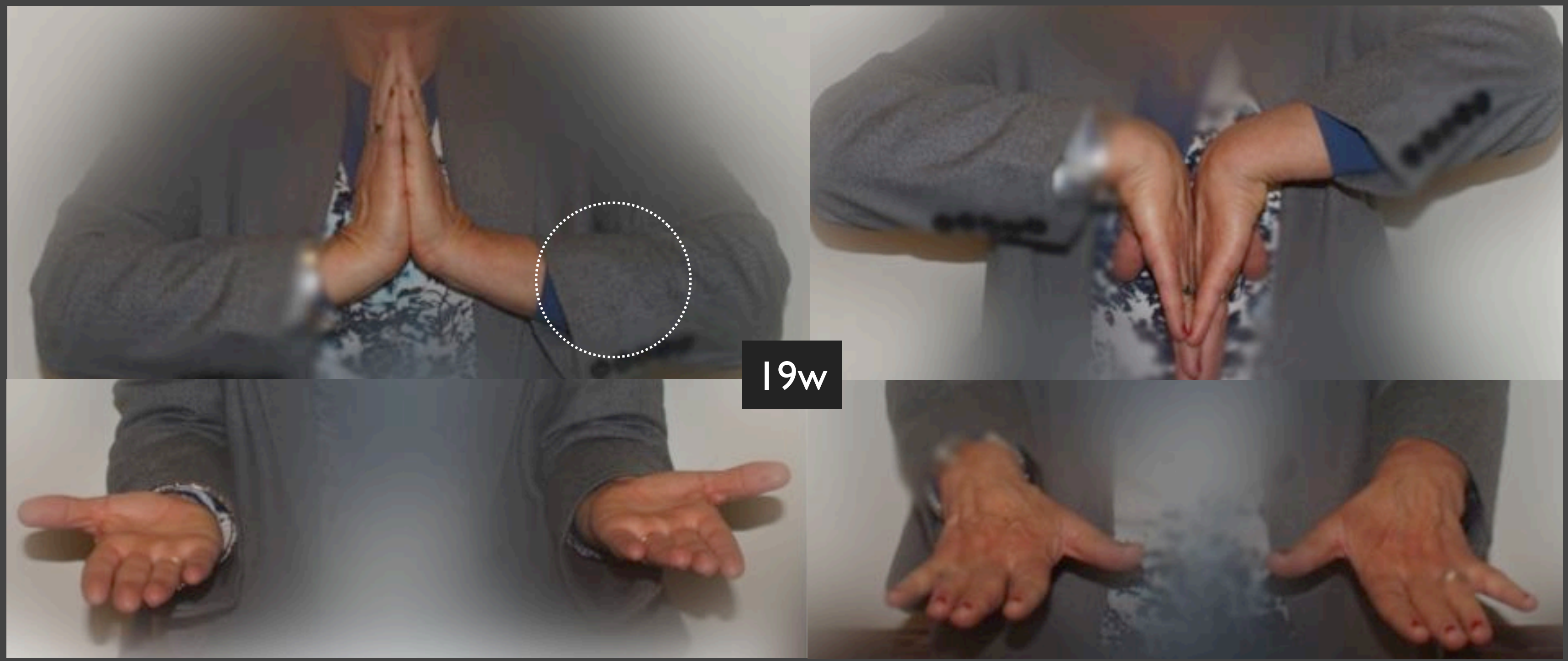

19w

ICUC Score at 345w Functional limitation: 0 Pain: 1

Quick DASH = 0

ICUC® ID: 23-DC-707 / 45y Female

Complete case data can be accessed by clicking the ***ICUC ID***

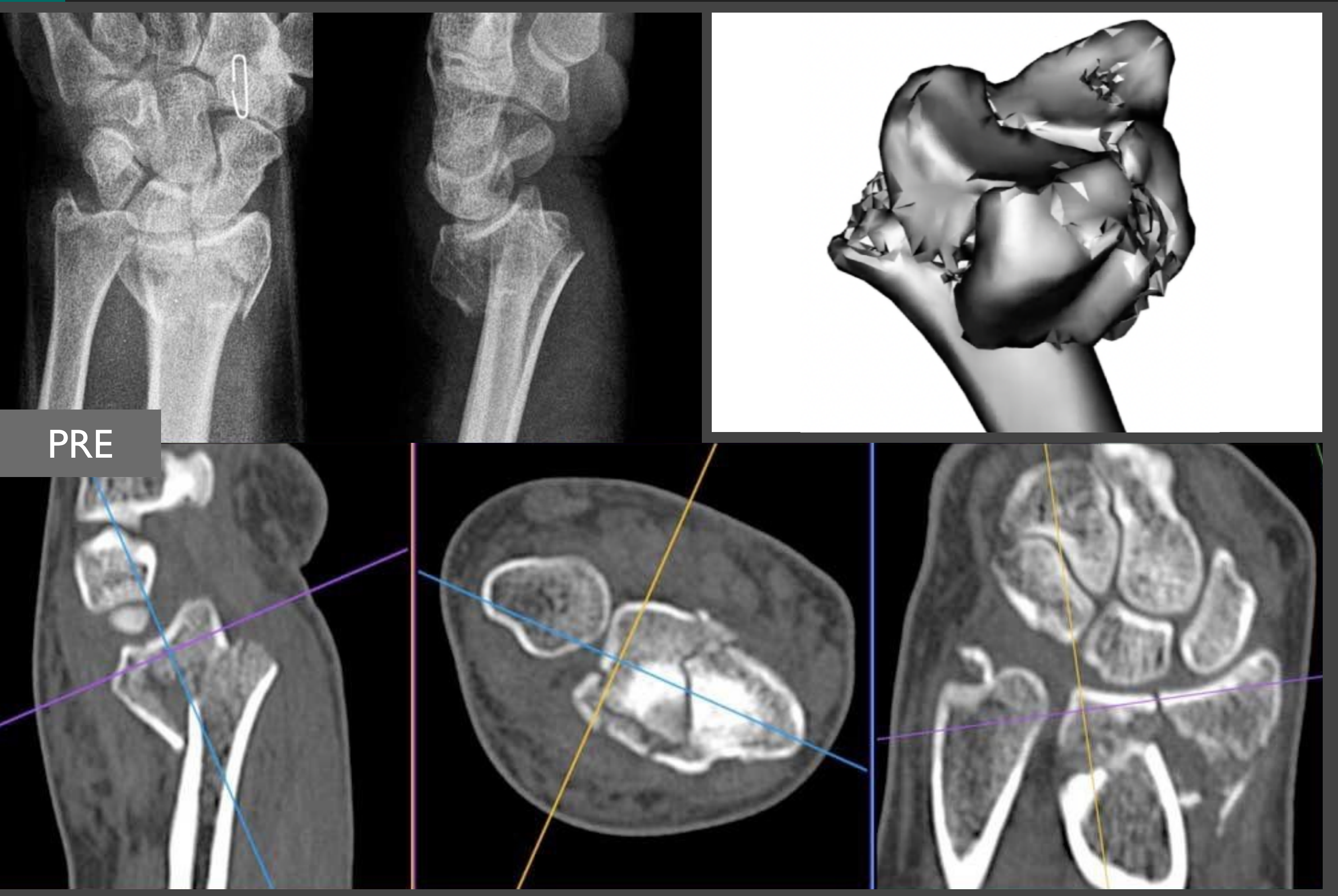

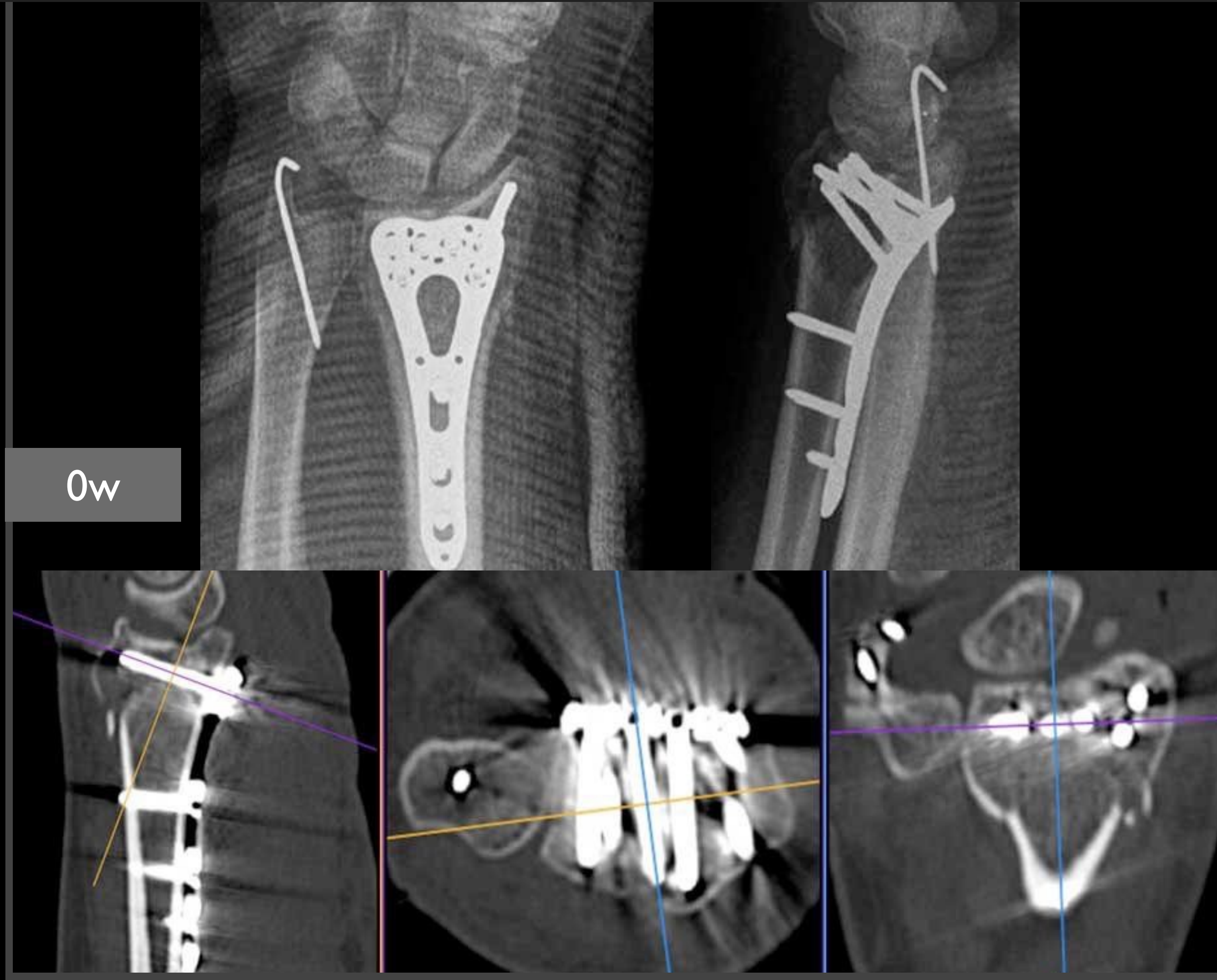

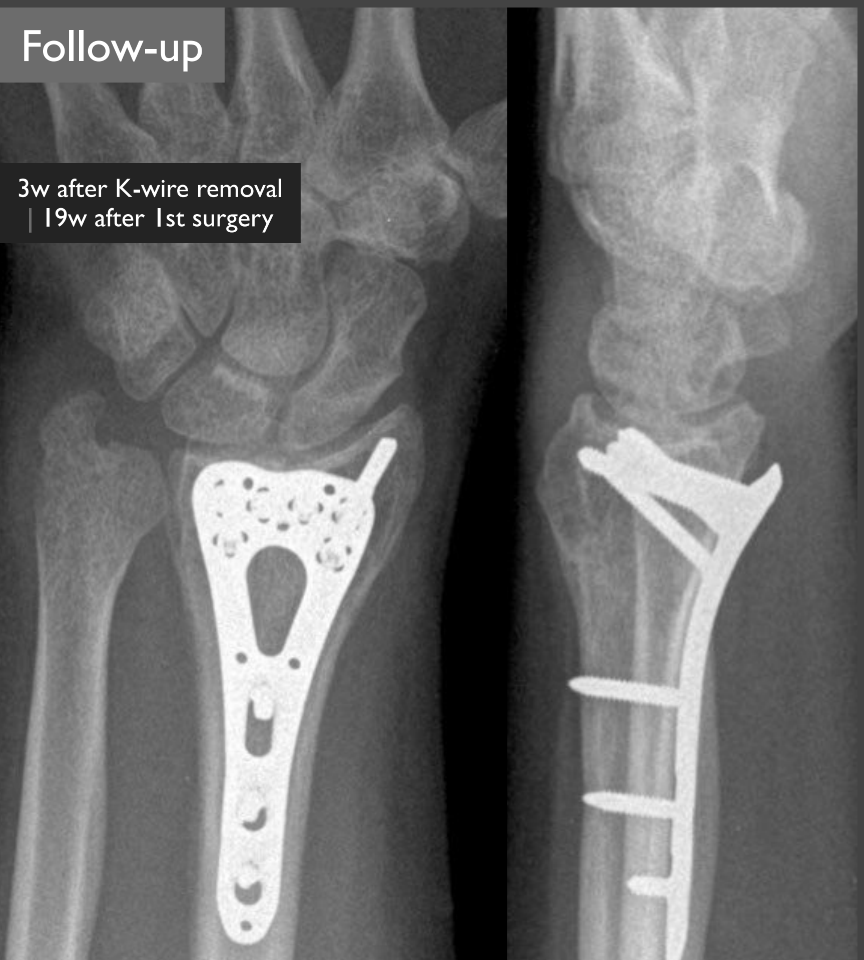

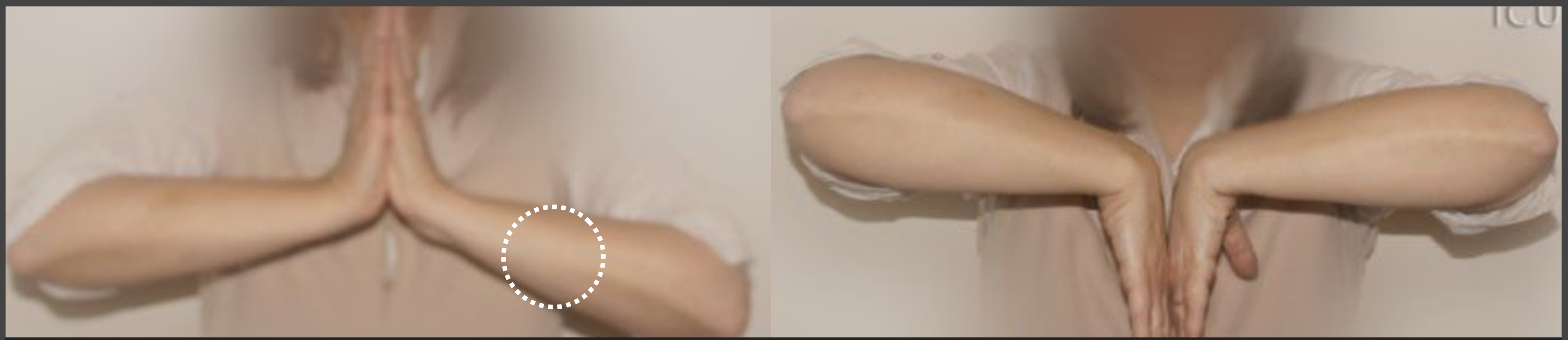

52w after Implant removal | 104w after K-wire removal | 120w after 1st surgery

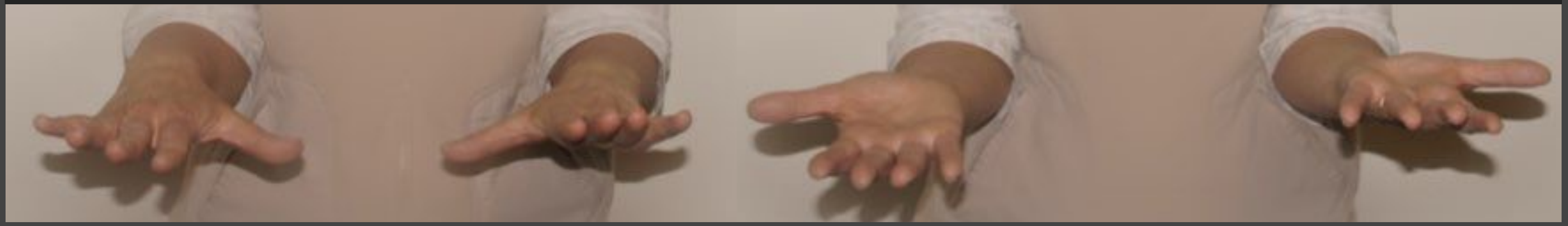

ICUC Score at 426w Functional limitation: 0 Pain: 0

Quick DASH = 0

ICUC® ID: 23-DC-221 / 60y Female

Complete case data can be accessed by clicking the ***ICUC ID***

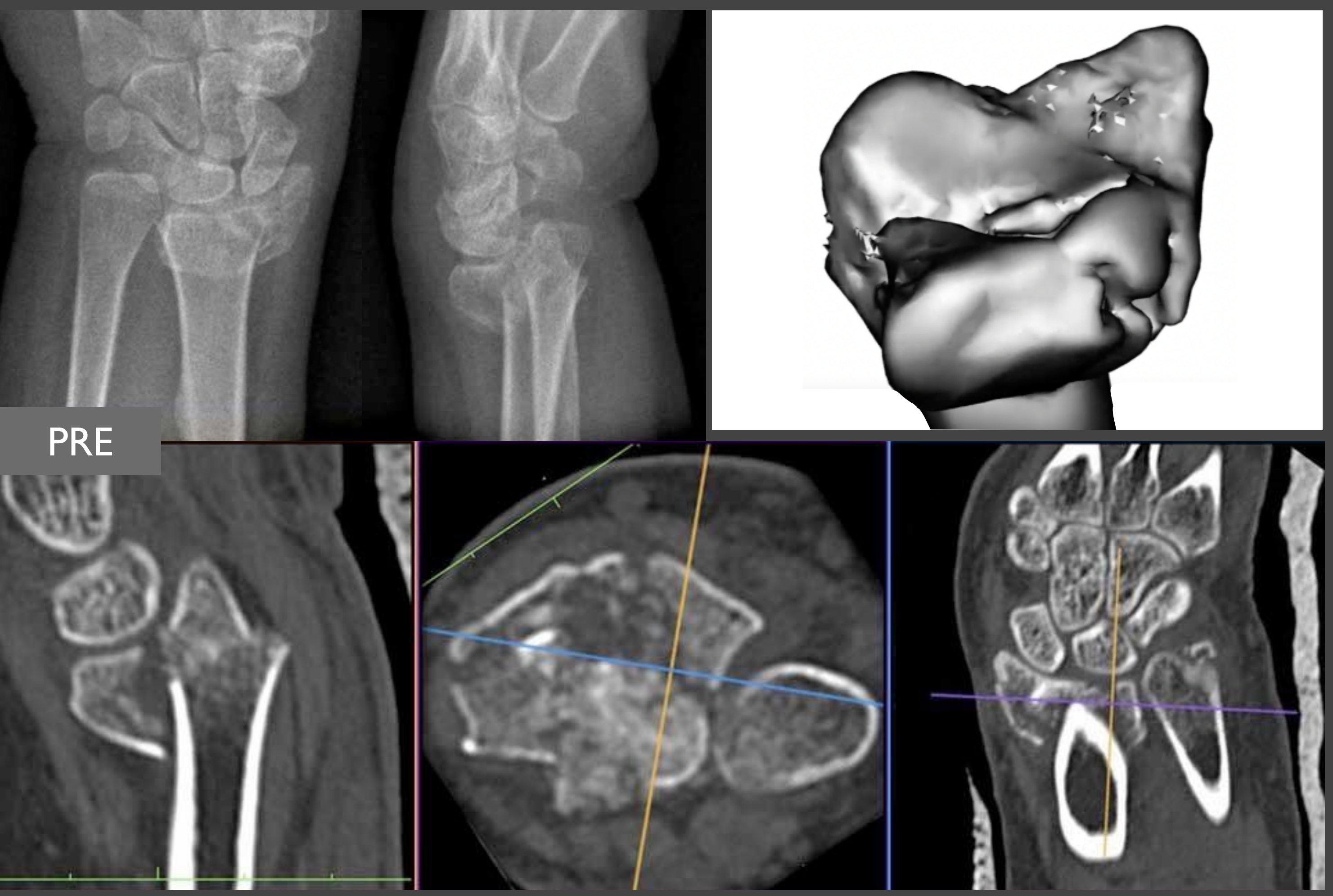

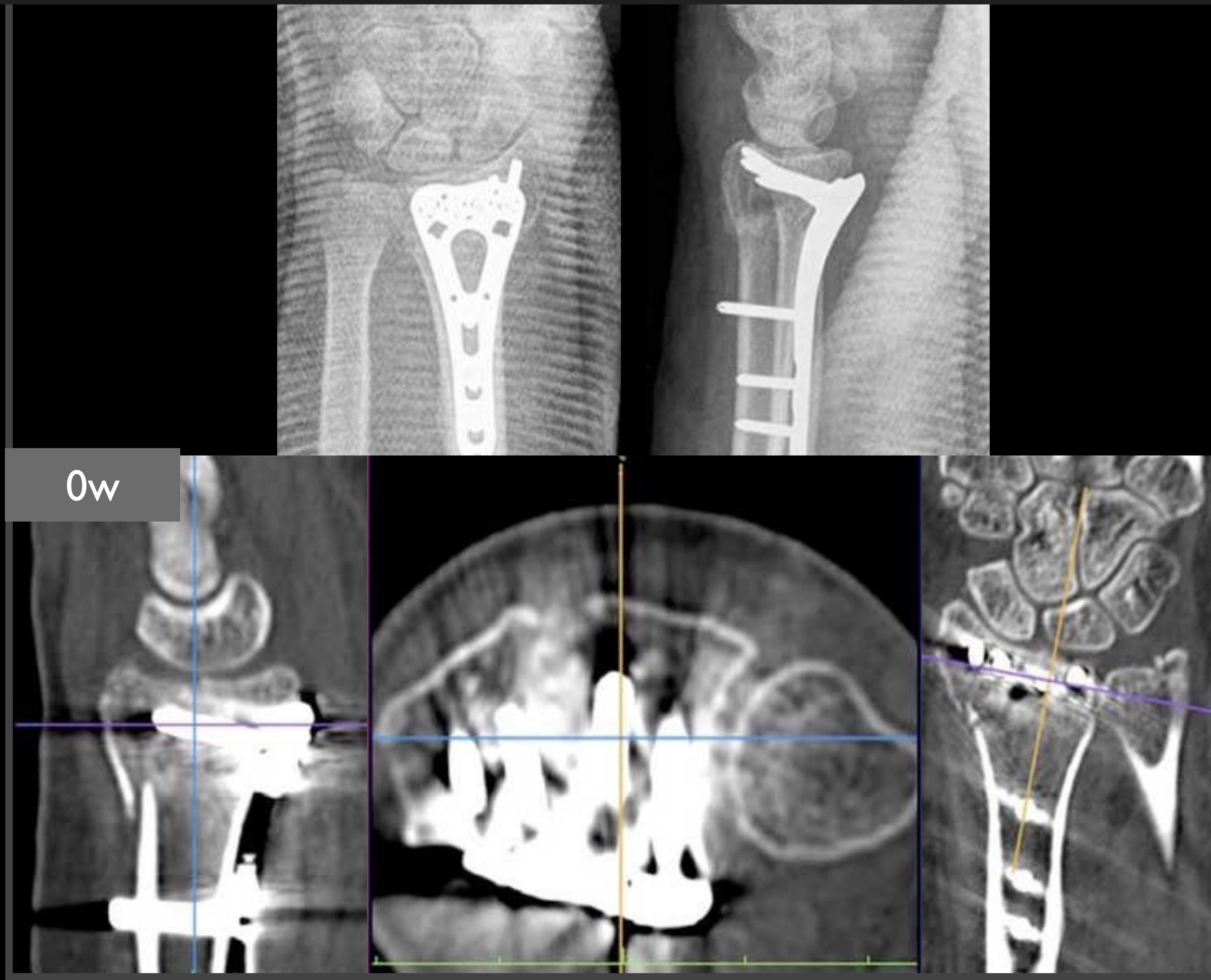

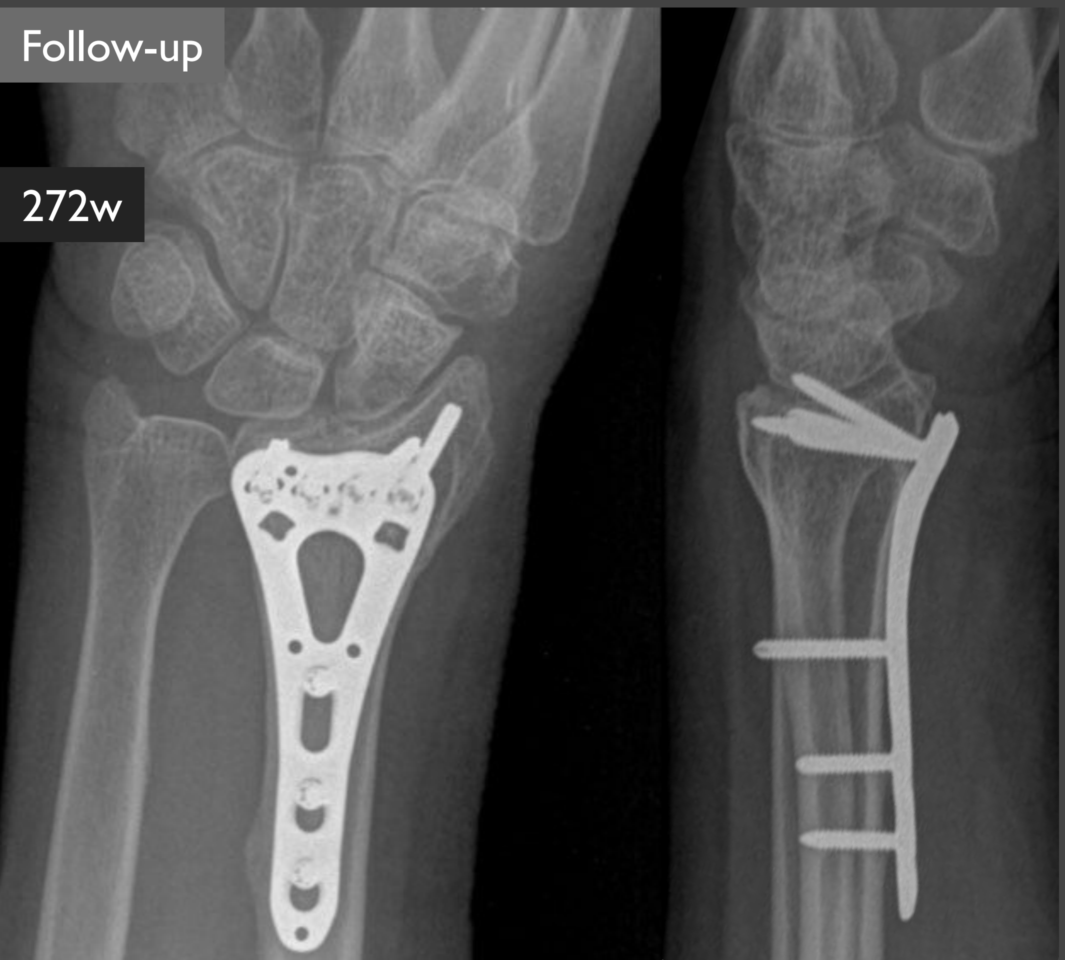

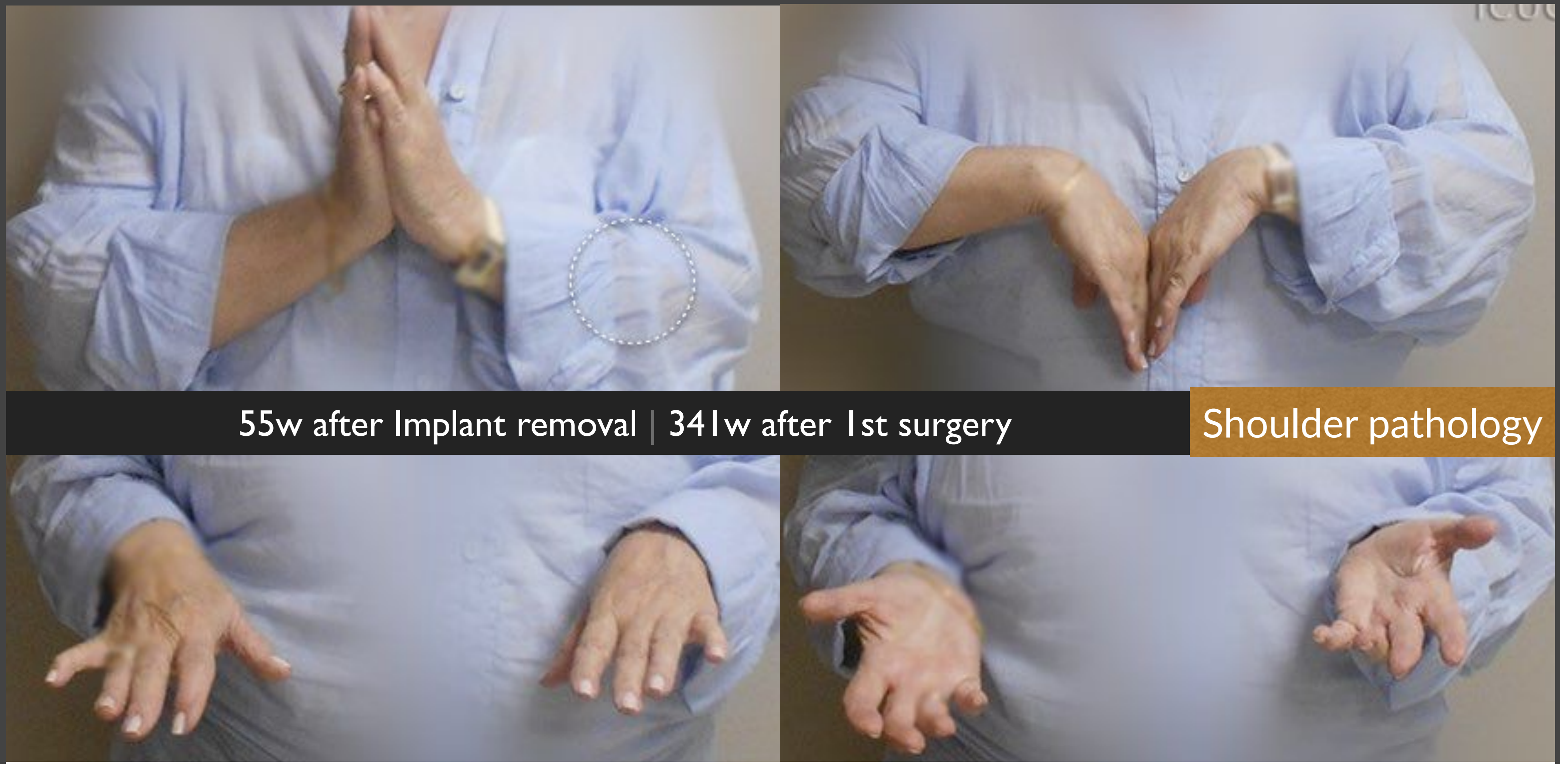

ICUC Score ***Functional Limitation:*** **0** (0-4) - ***Pain:*** **0** (0-4)

Quick DASH = 30

ICUC® ID: 23-DC-256 / 70y Female

*Complete case data can be accessed by clicking the **ICUC ID***

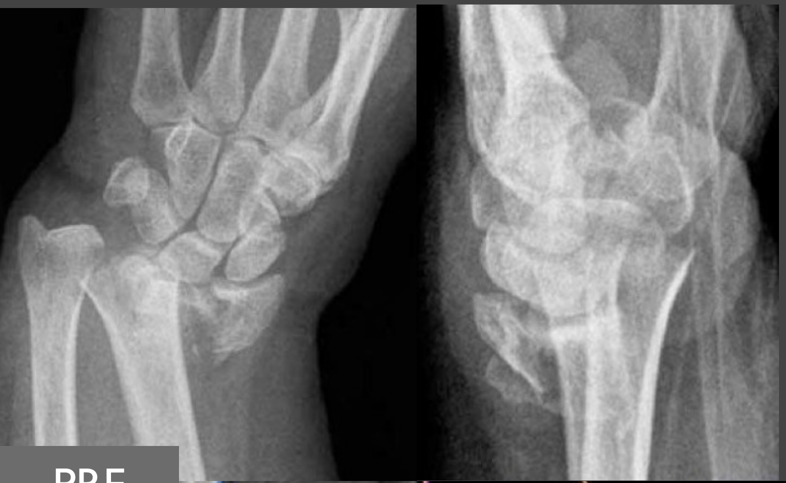

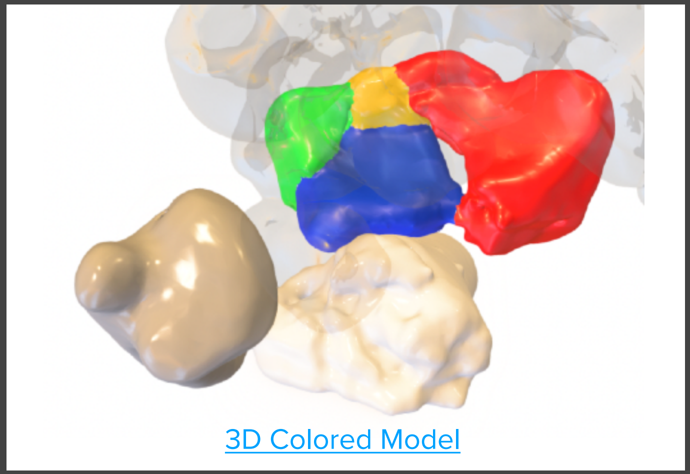

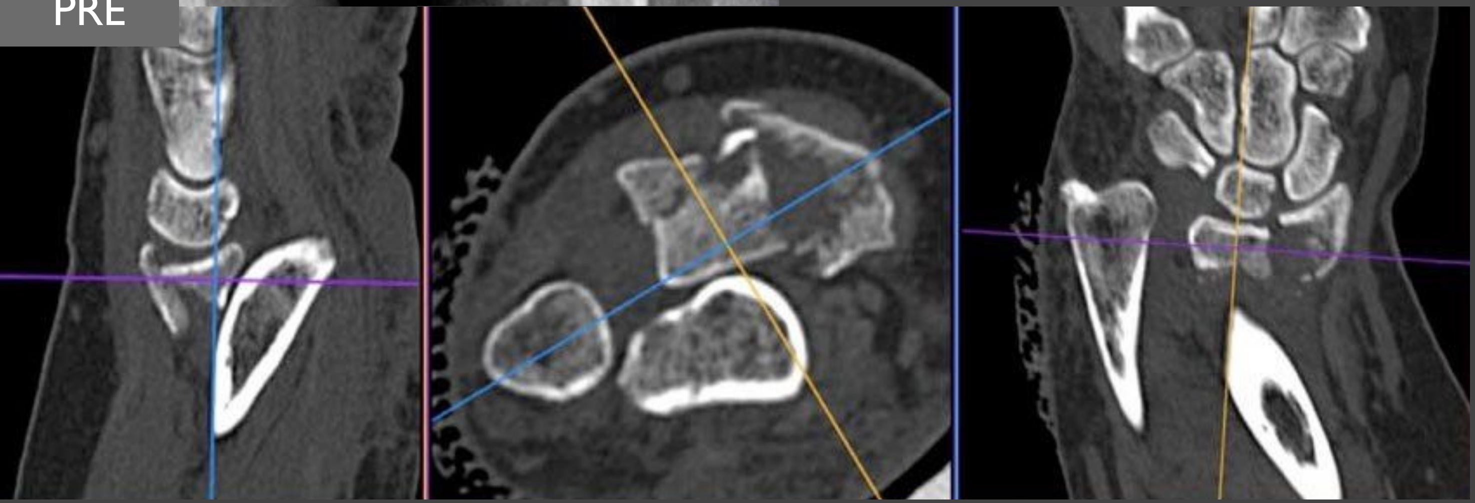

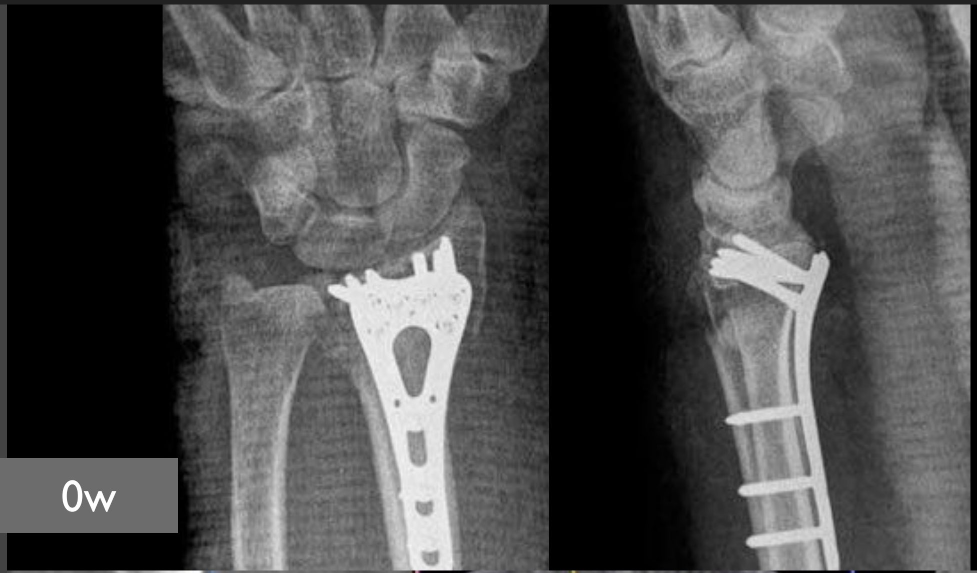

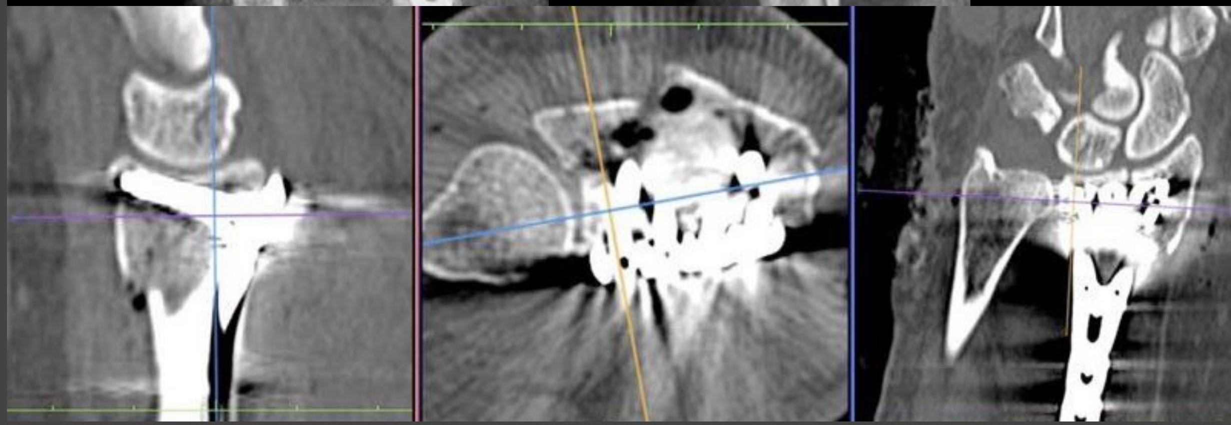

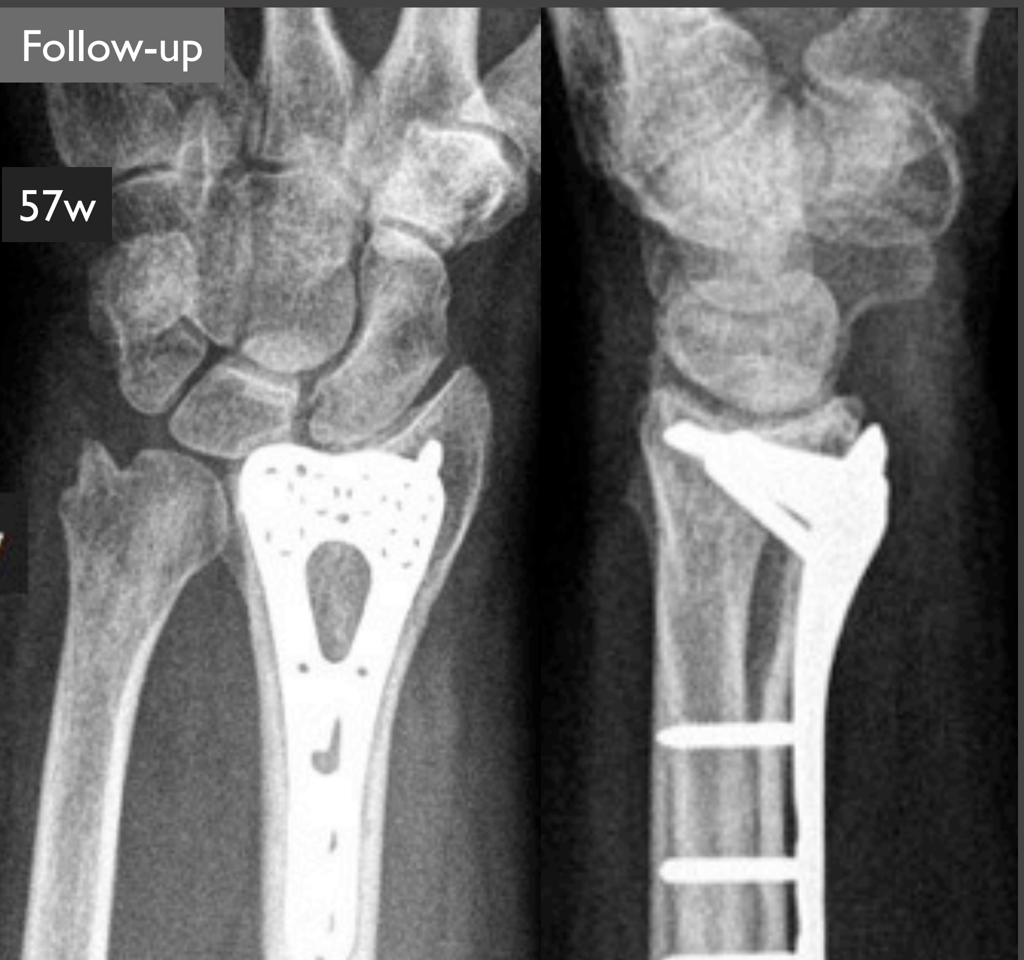

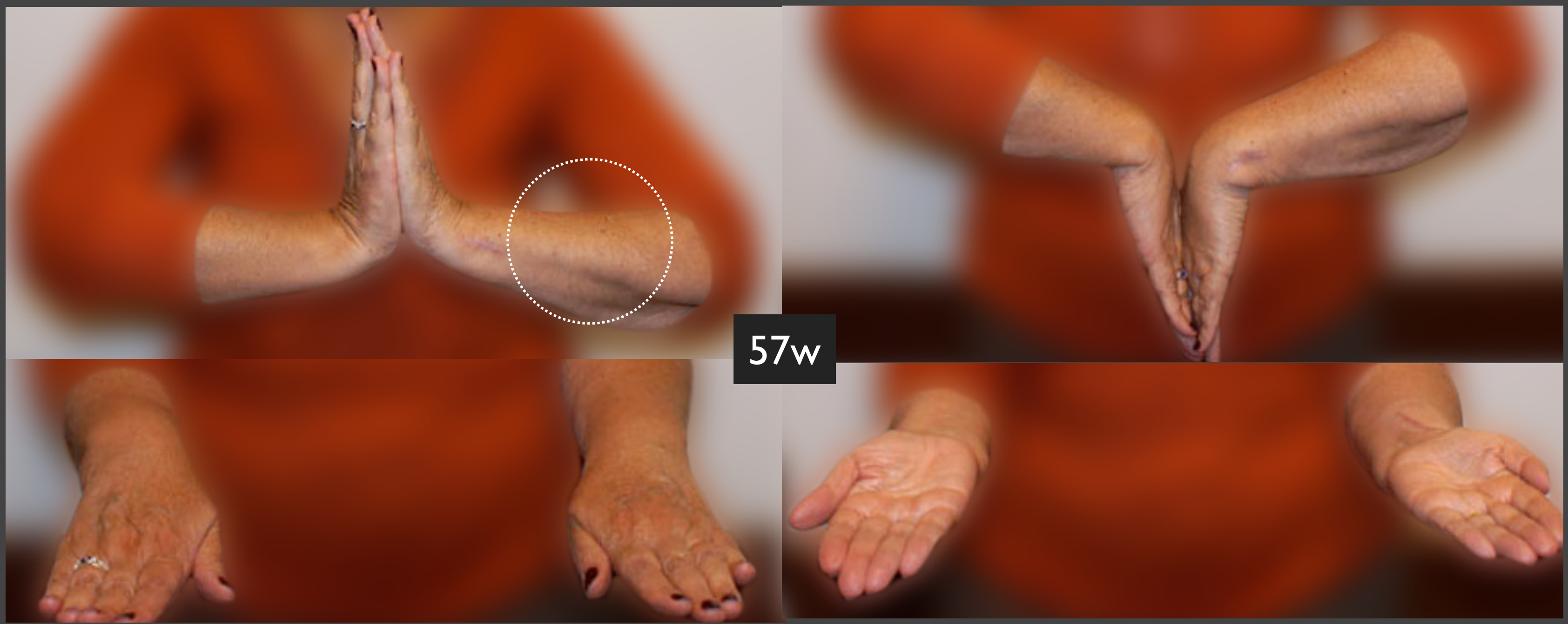

ICUC Score at 244w Functional limitation: 0 Pain: 0

Quick DASH = 11

ICUC® ID: 23-DC-580 / 65y Female

Complete case data can be accessed by clicking the ***ICUC ID***

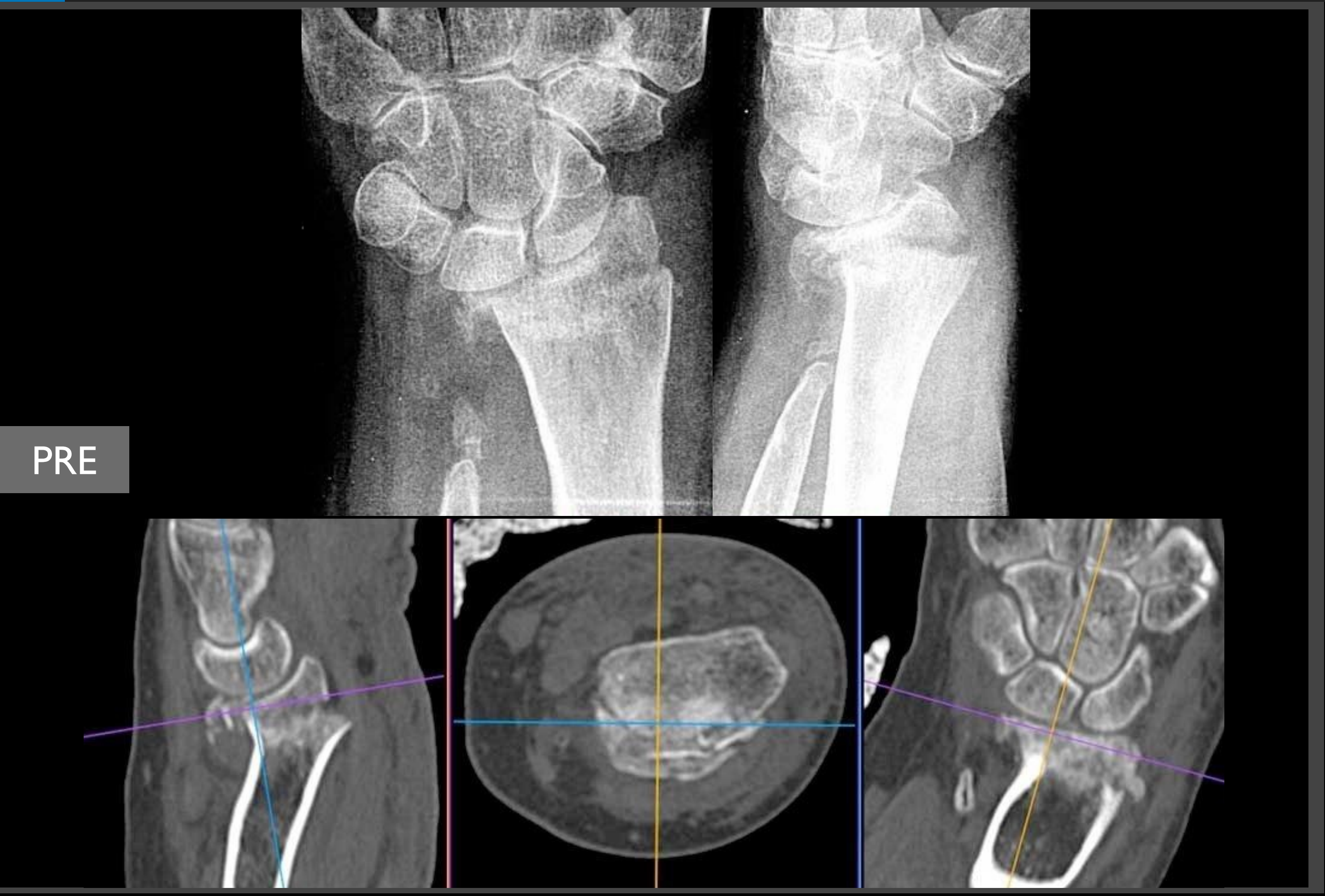

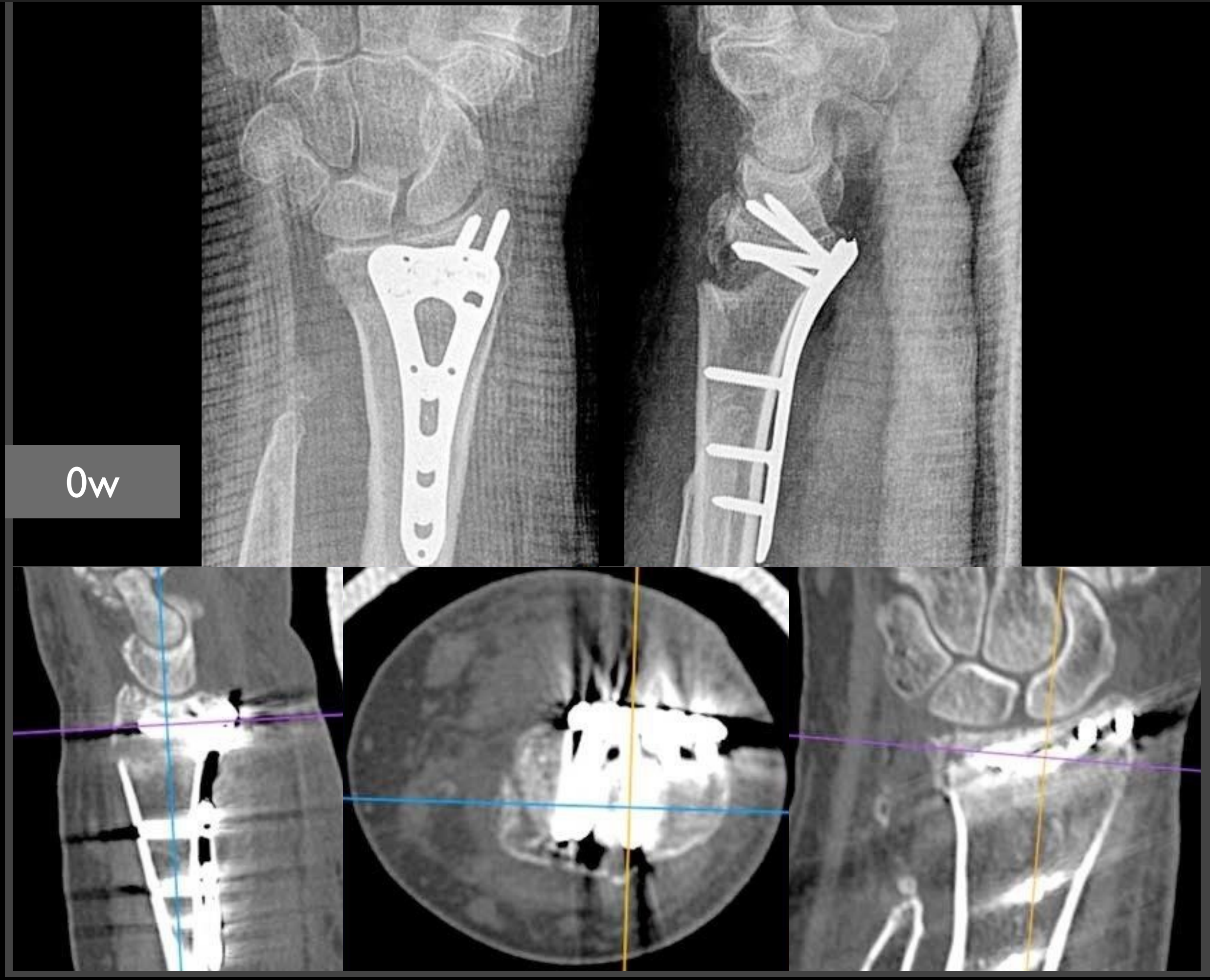

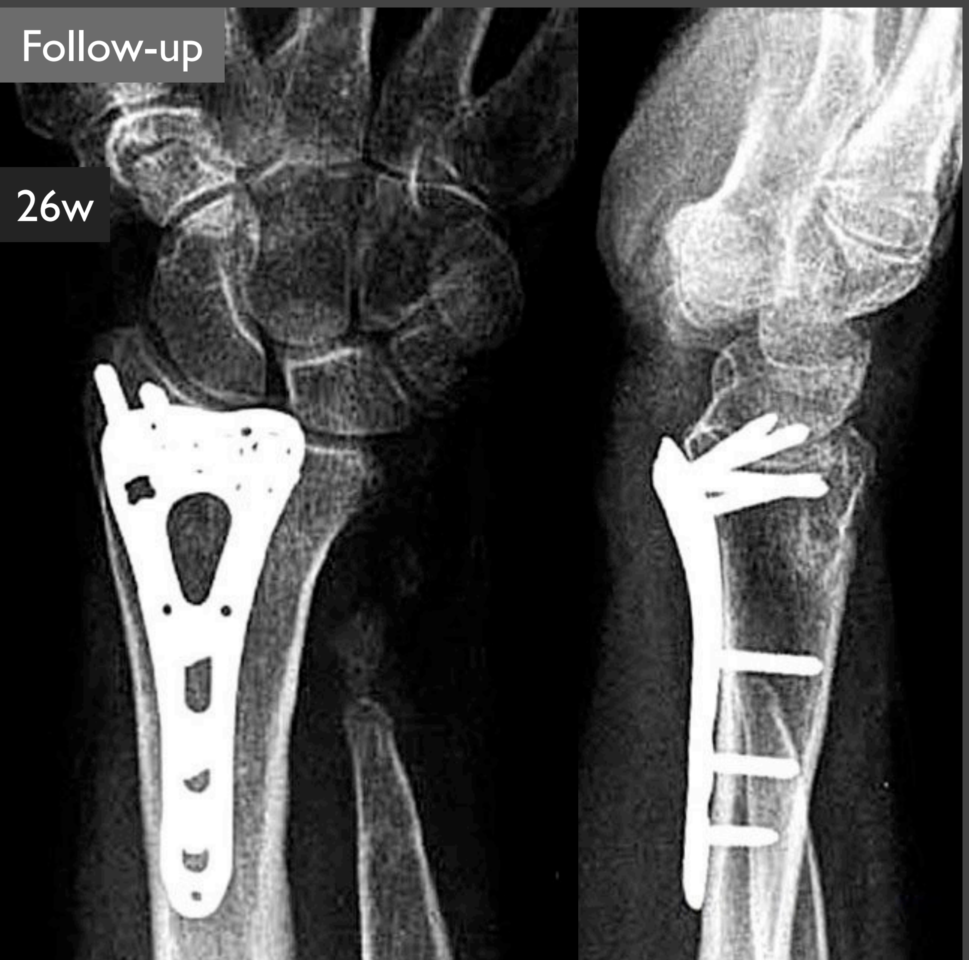

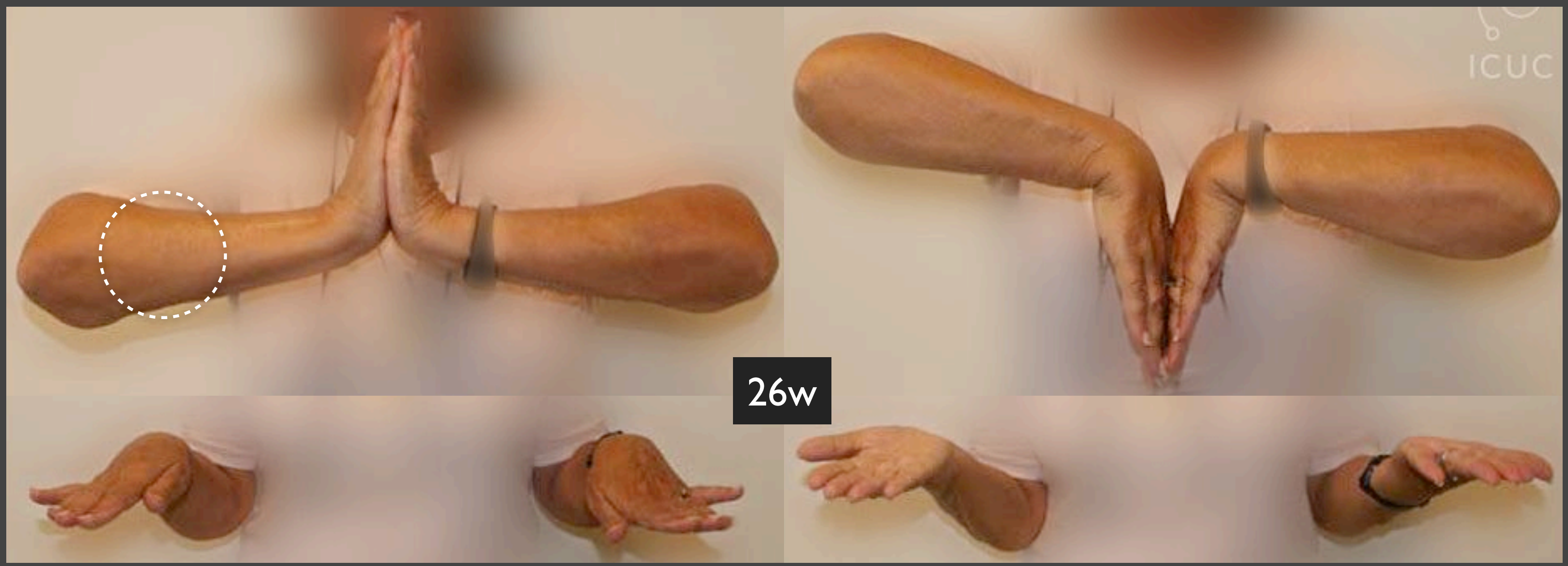

ICUC Score at 350w Functional limitation: 0 Pain: 0

Quick DASH = 14

ICUC® ID: 23-DC-577 / 45y Male

Complete case data can be accessed by clicking the ***ICUC ID***

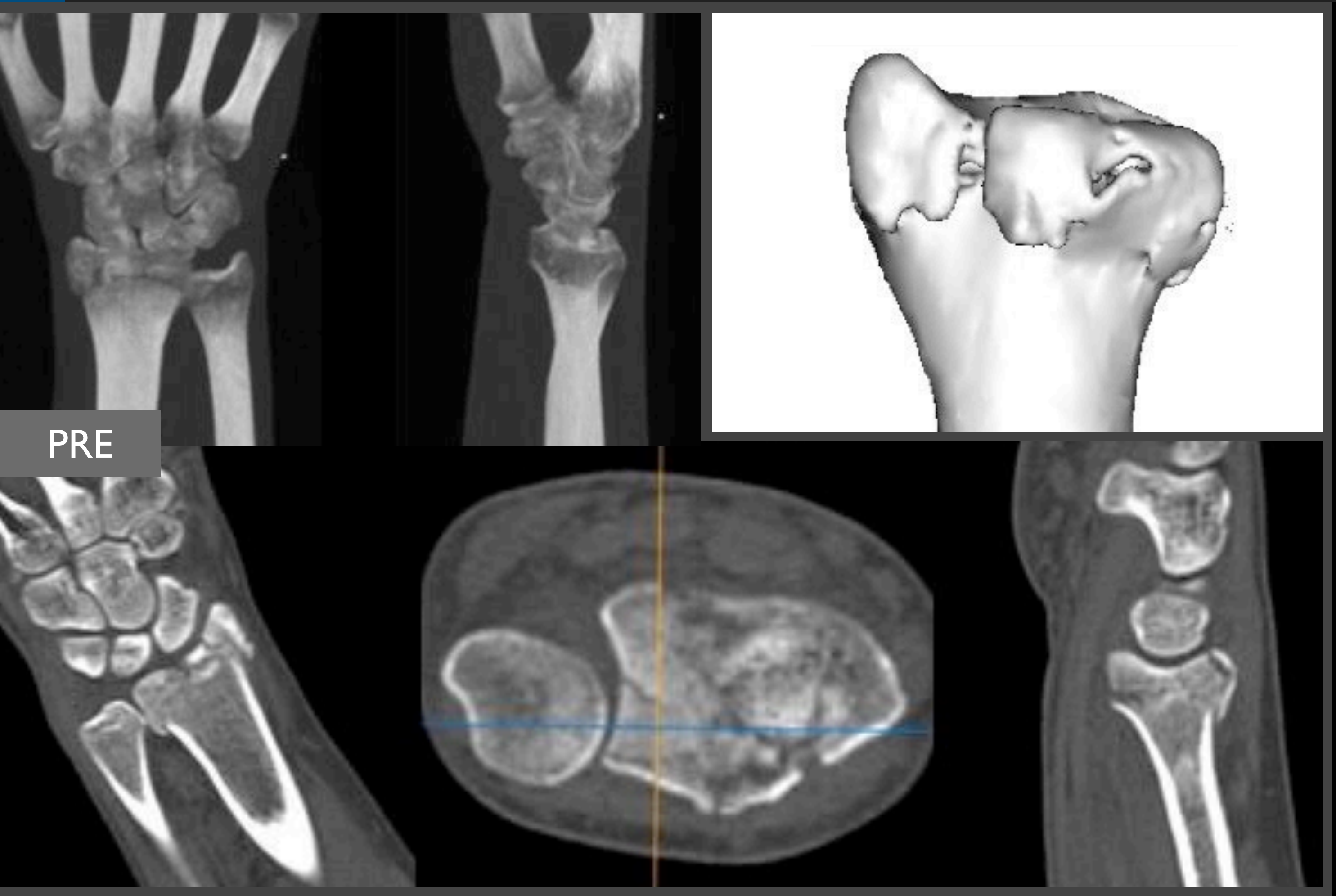

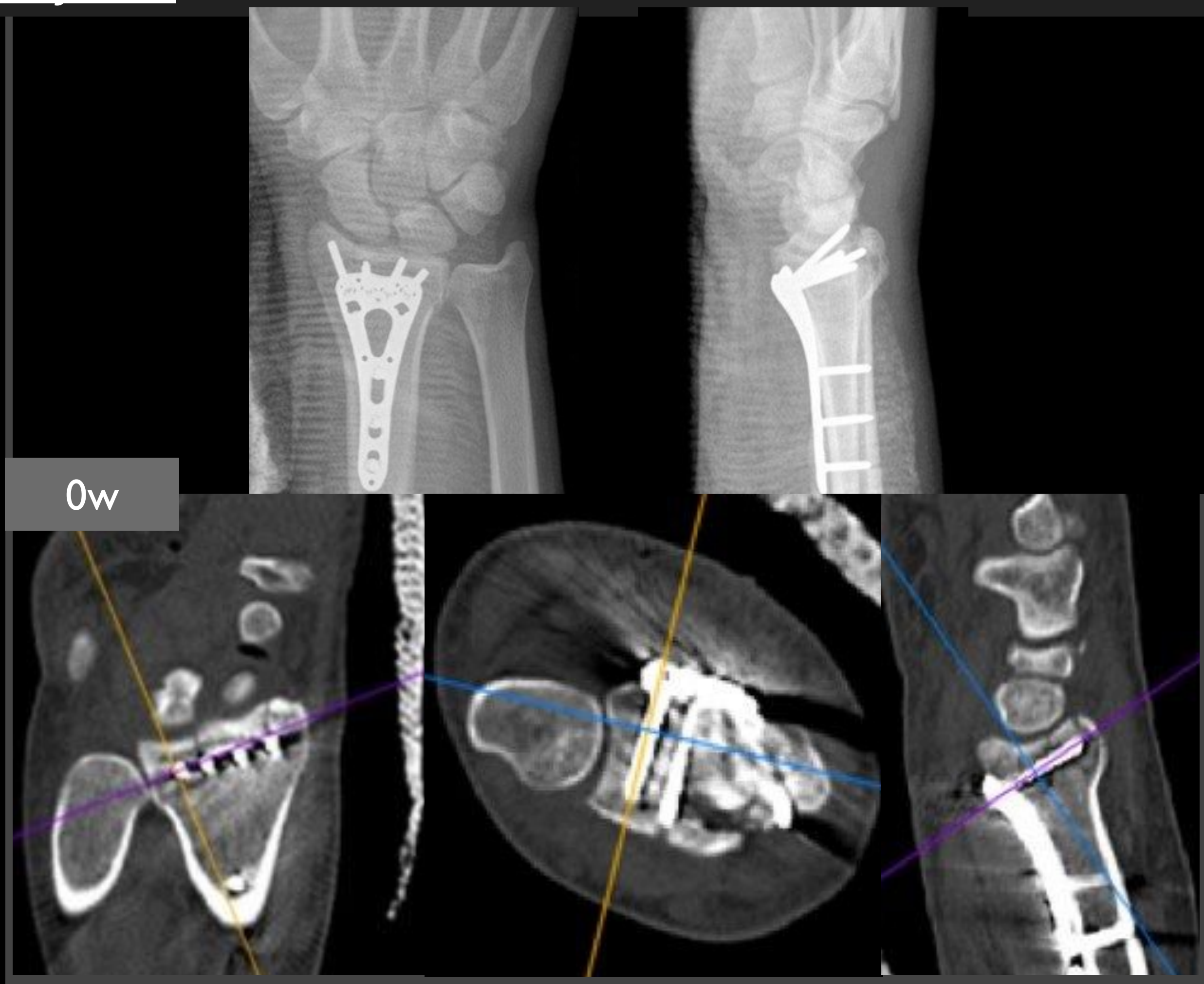

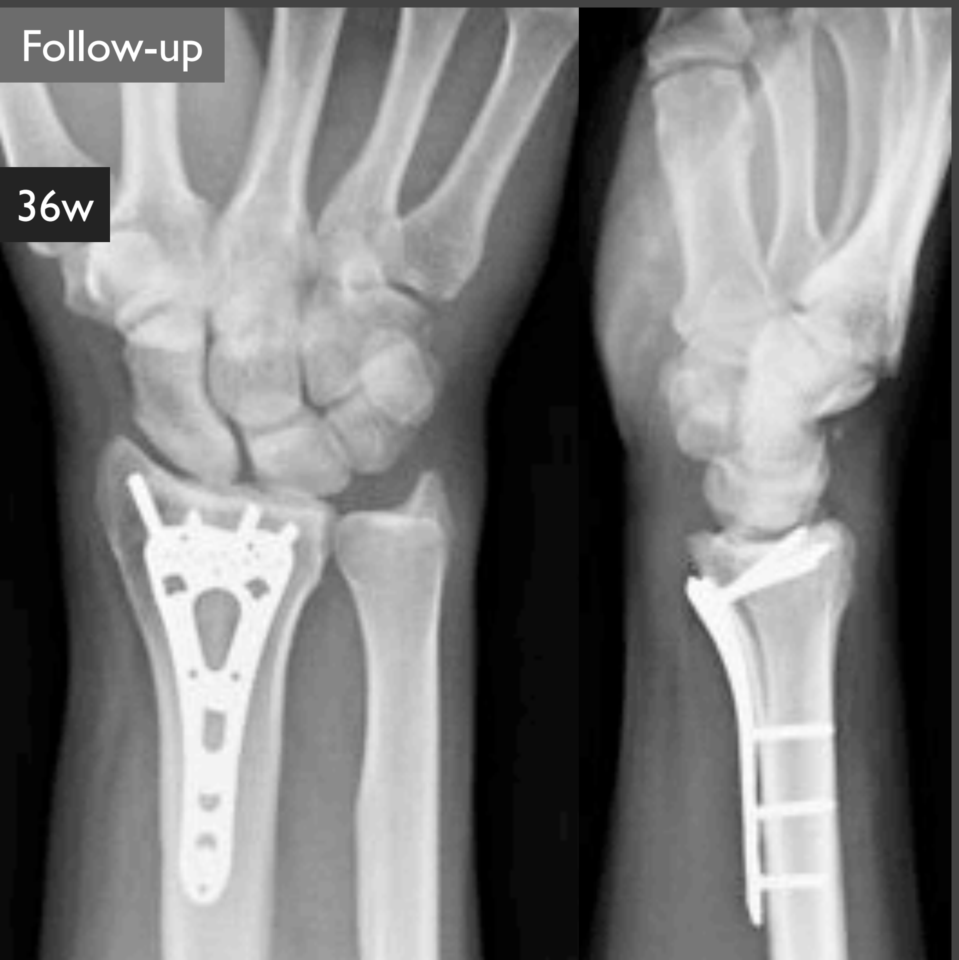

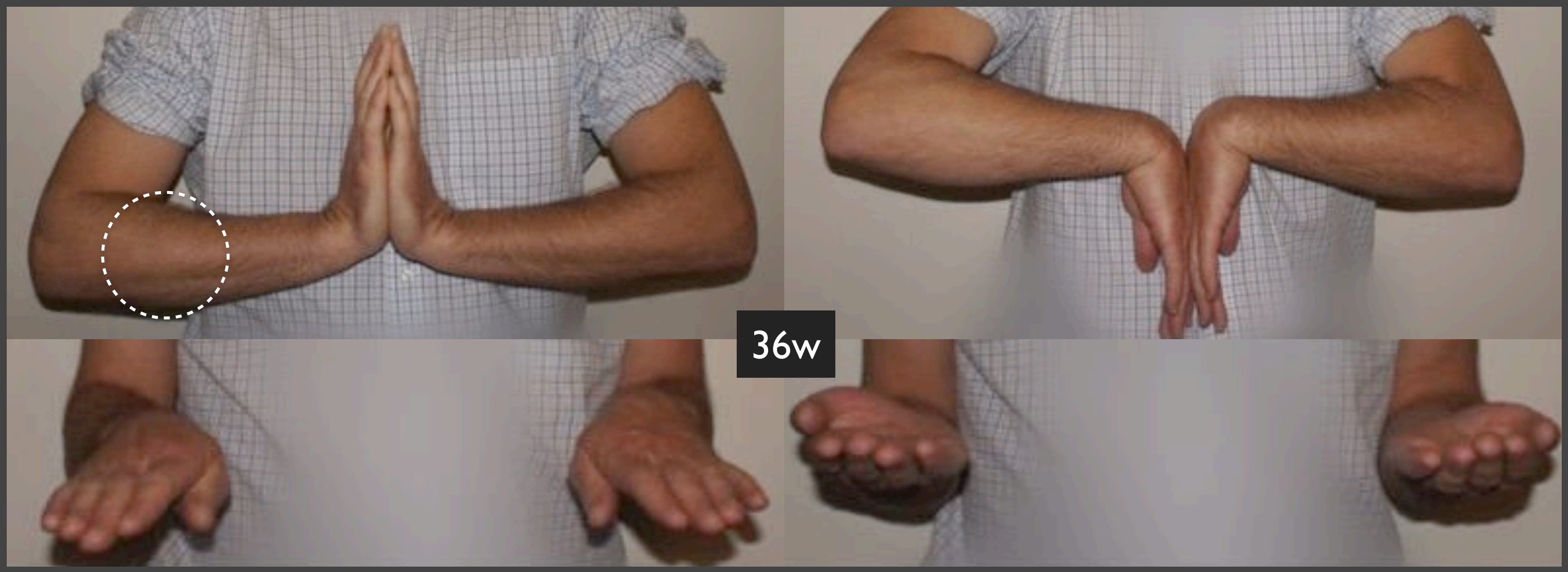

ICUC Score at 153w Functional limitation: 0 Pain: 0

Quick DASH = 2

ICUC® ID: 23-DC-224 / 70y Female

Complete case data can be accessed by clicking the ***ICUC ID***

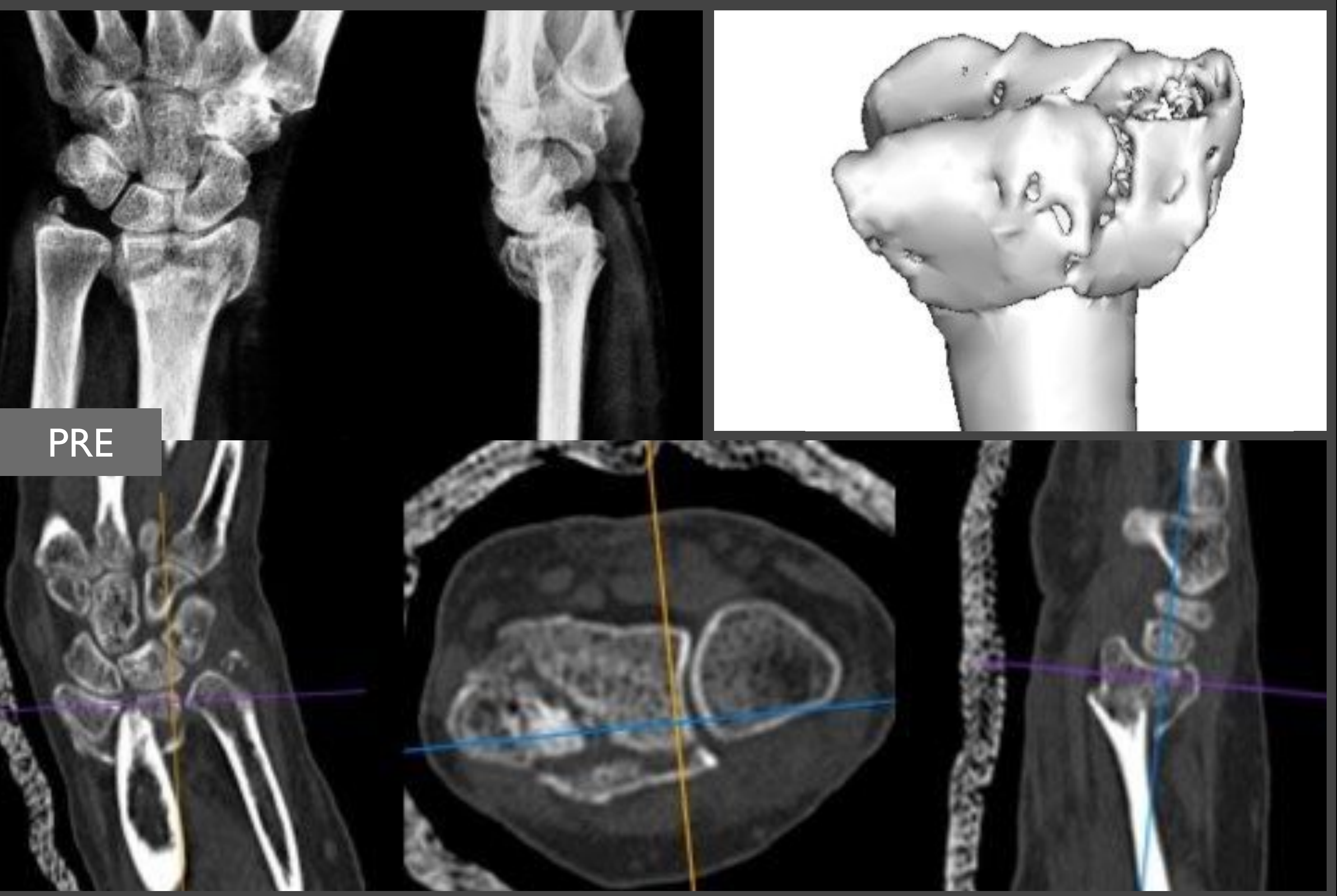

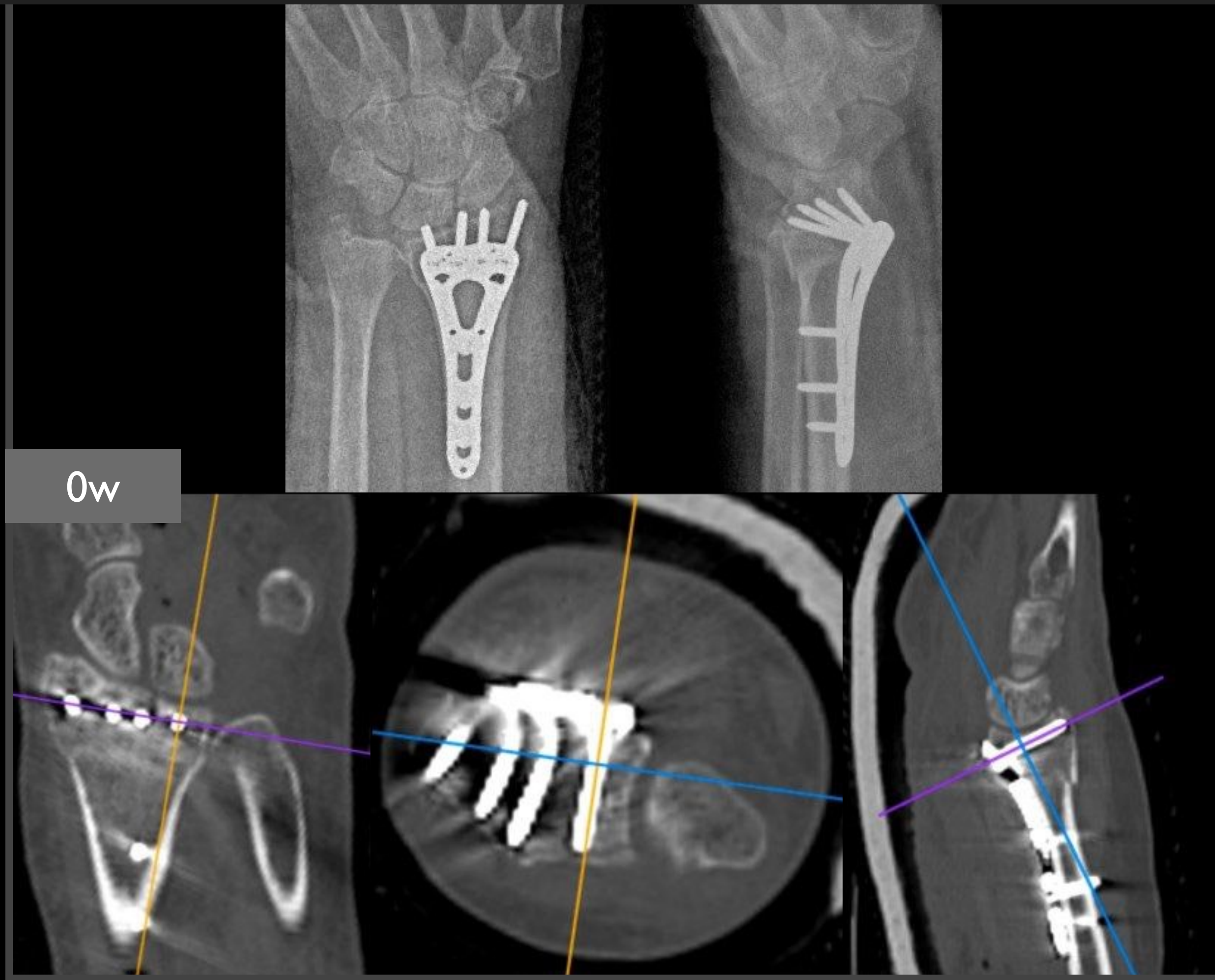

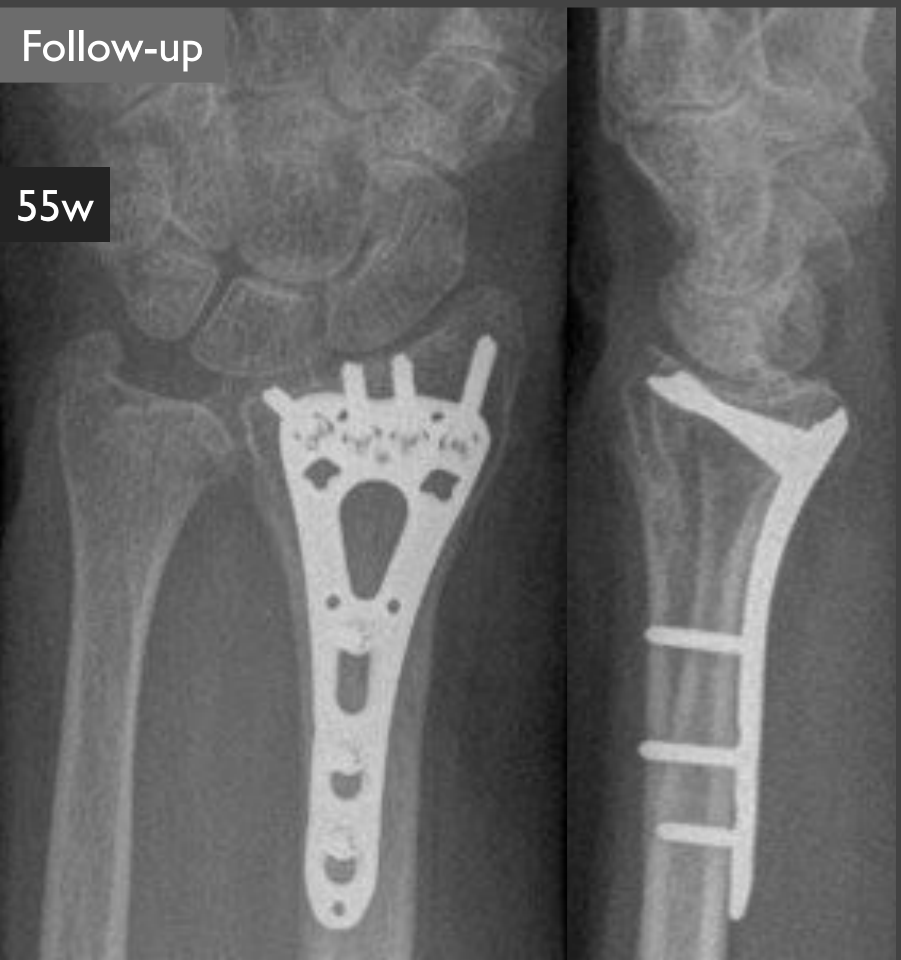

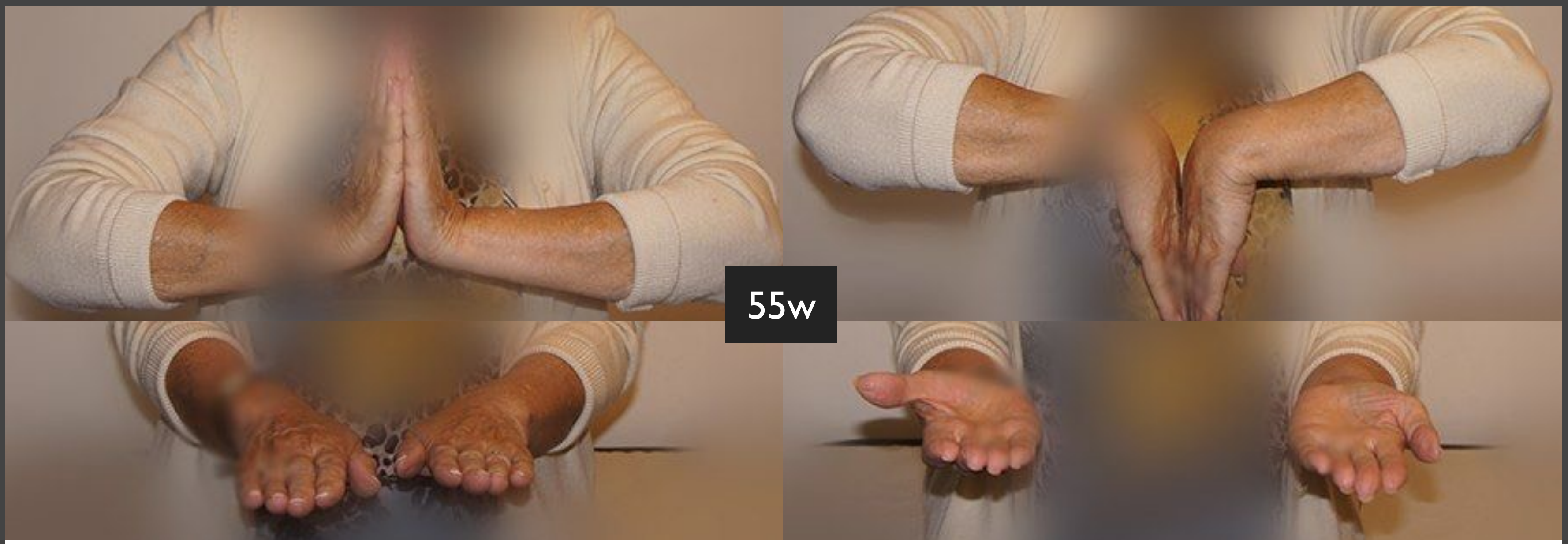

ICUC ***Score*** — ***Functional Limitation:*** **1** (0-4) - ***Pain:*** **1** (0-4)

Quick DASH = 0

ICUC® ID: 23-DC-263 / 35y Male

Complete case data can be accessed by clicking the ***ICUC ID***

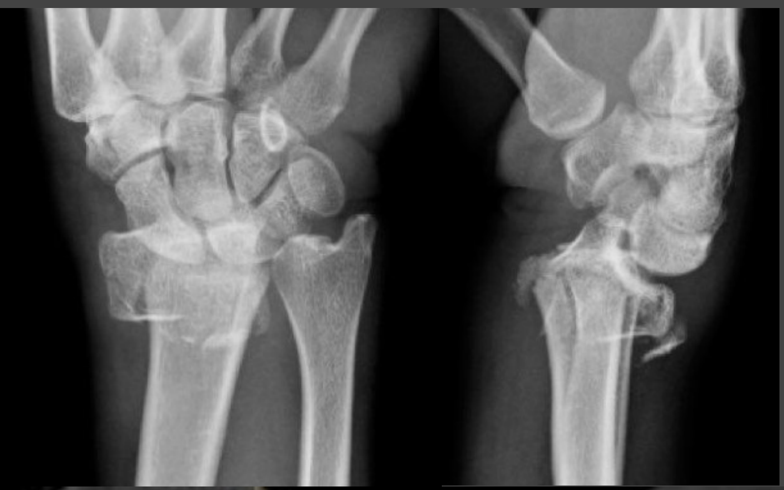

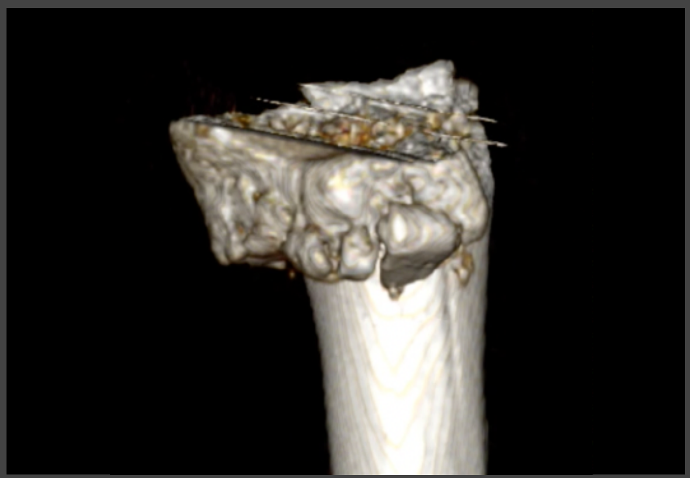

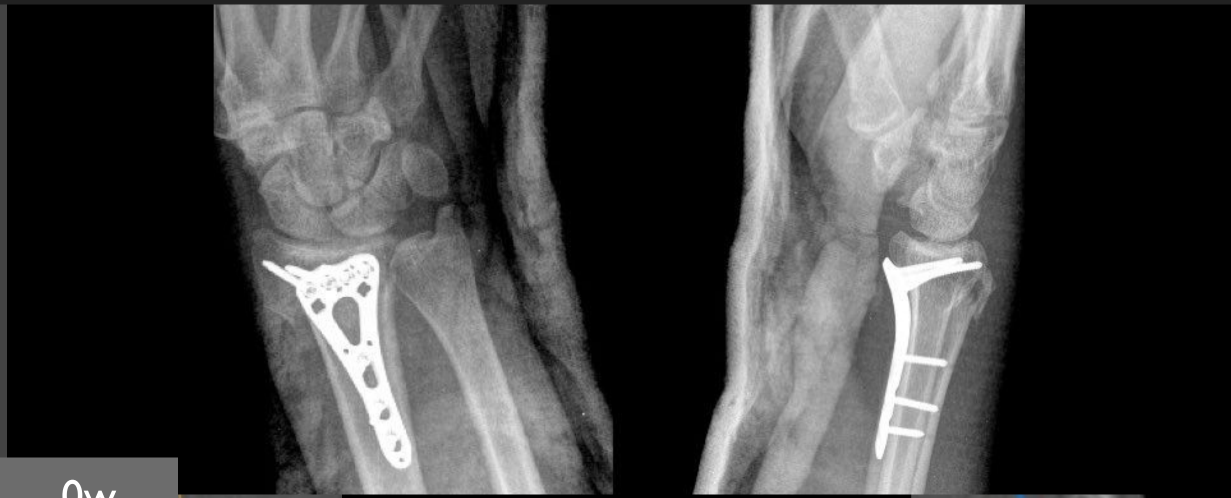

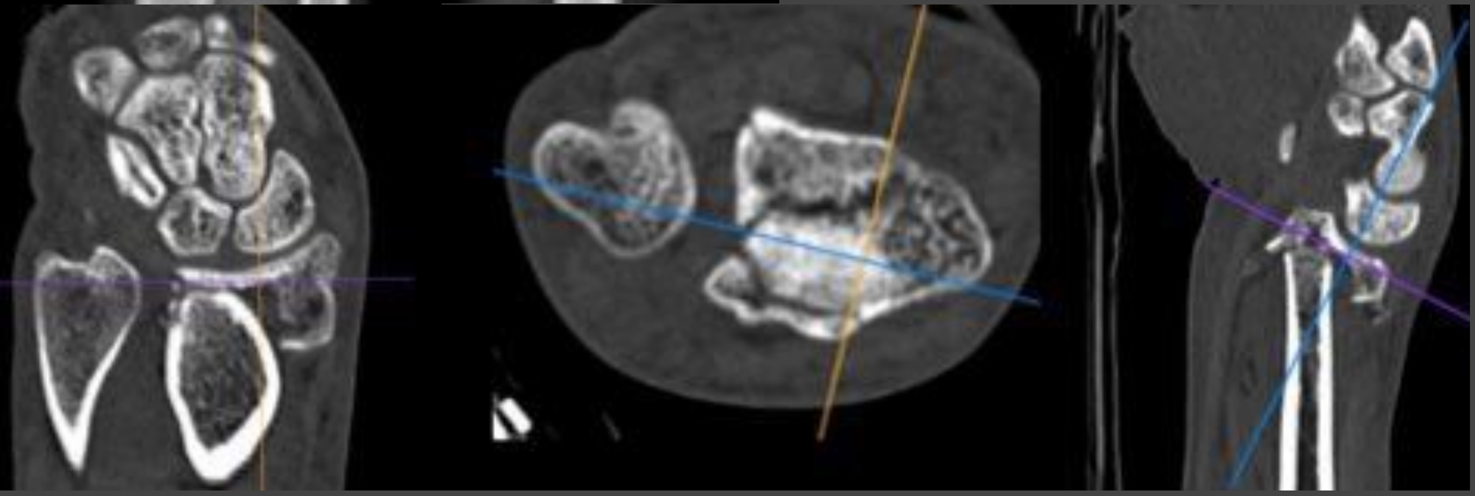

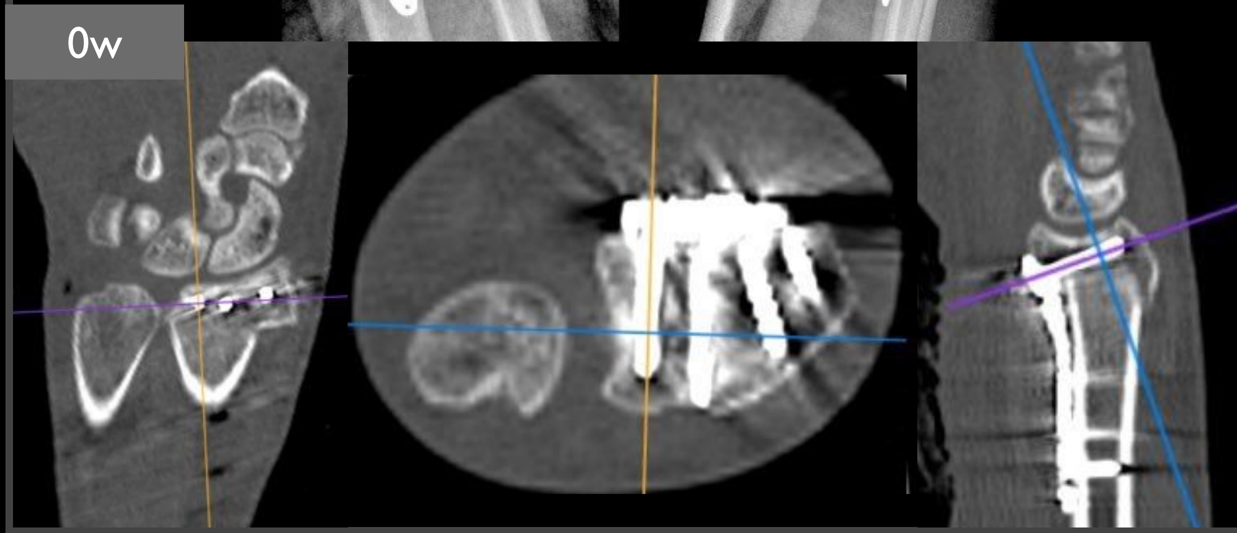

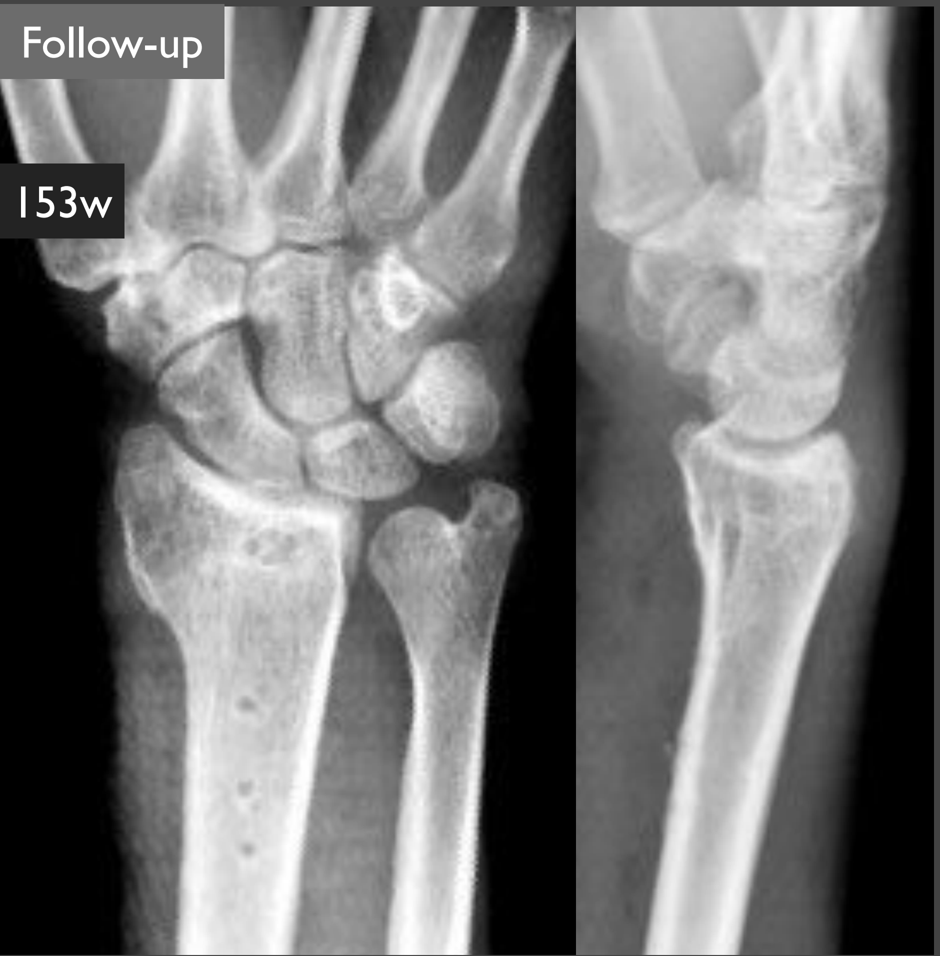

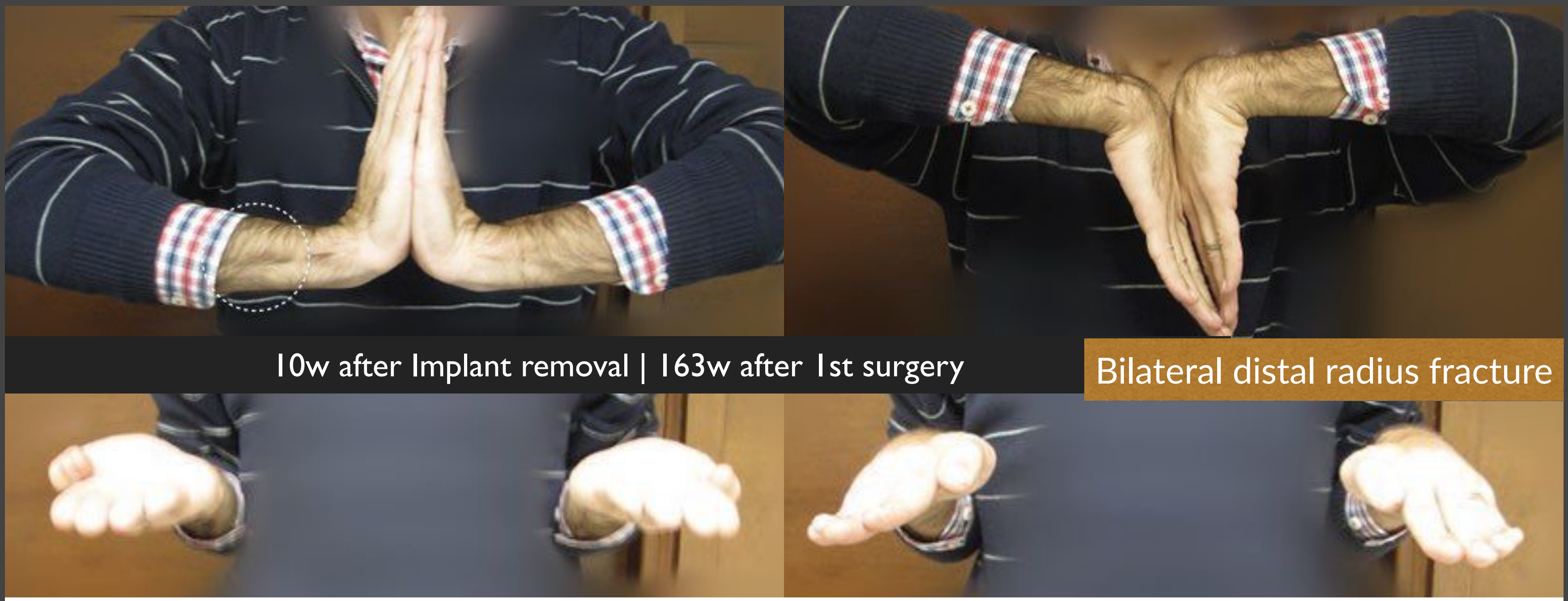

10w after Implant removal | 163w after 1st surgery

Bilateral distal radius fracture

ICUC **Score** *Functional Limitation:* **1** (0-4) - *Pain:* **0** (0-4)

Quick DASH = 9

ICUC® ID: 23-DC-494 / 25y Male

Complete case data can be accessed by clicking the ***ICUC ID***

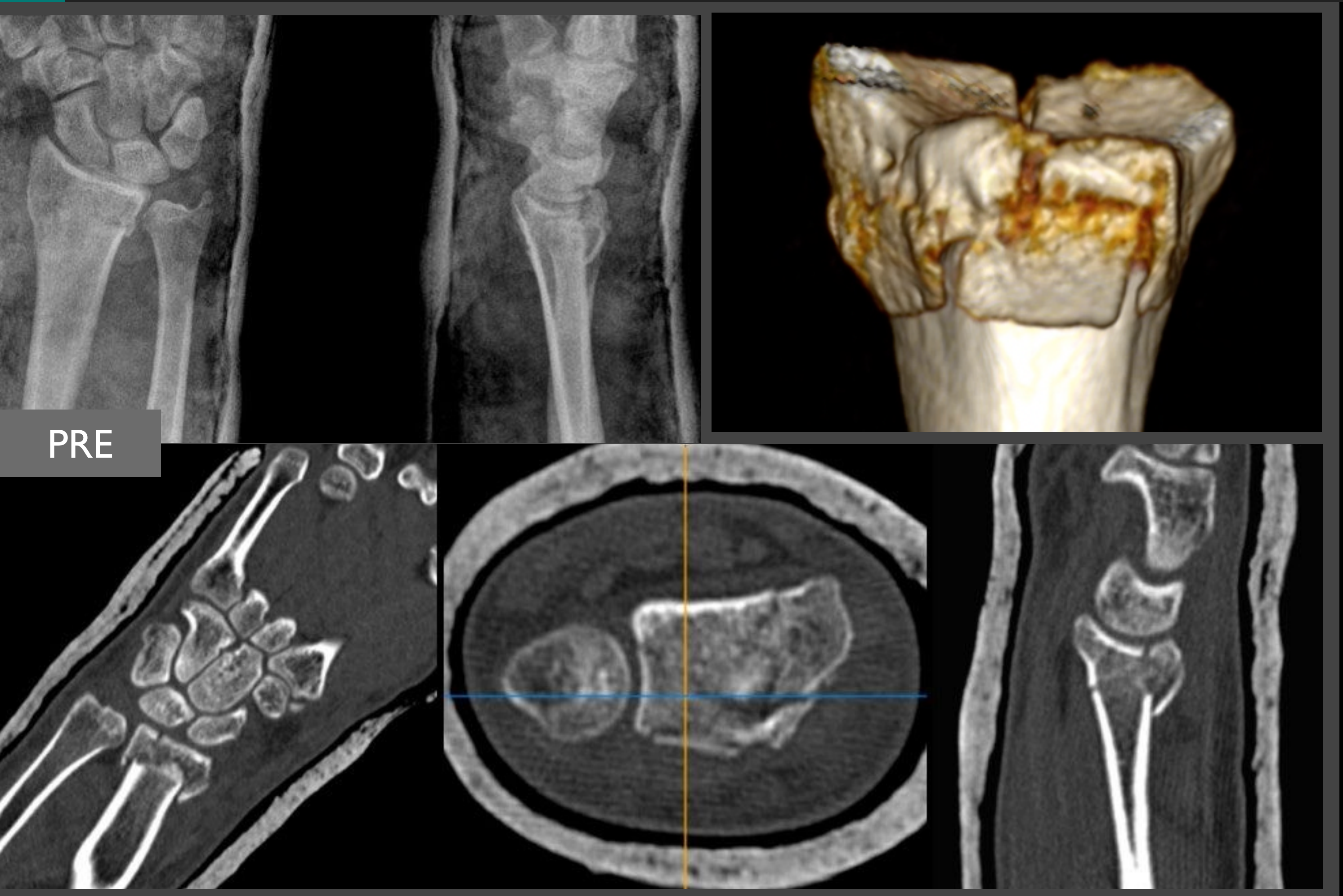

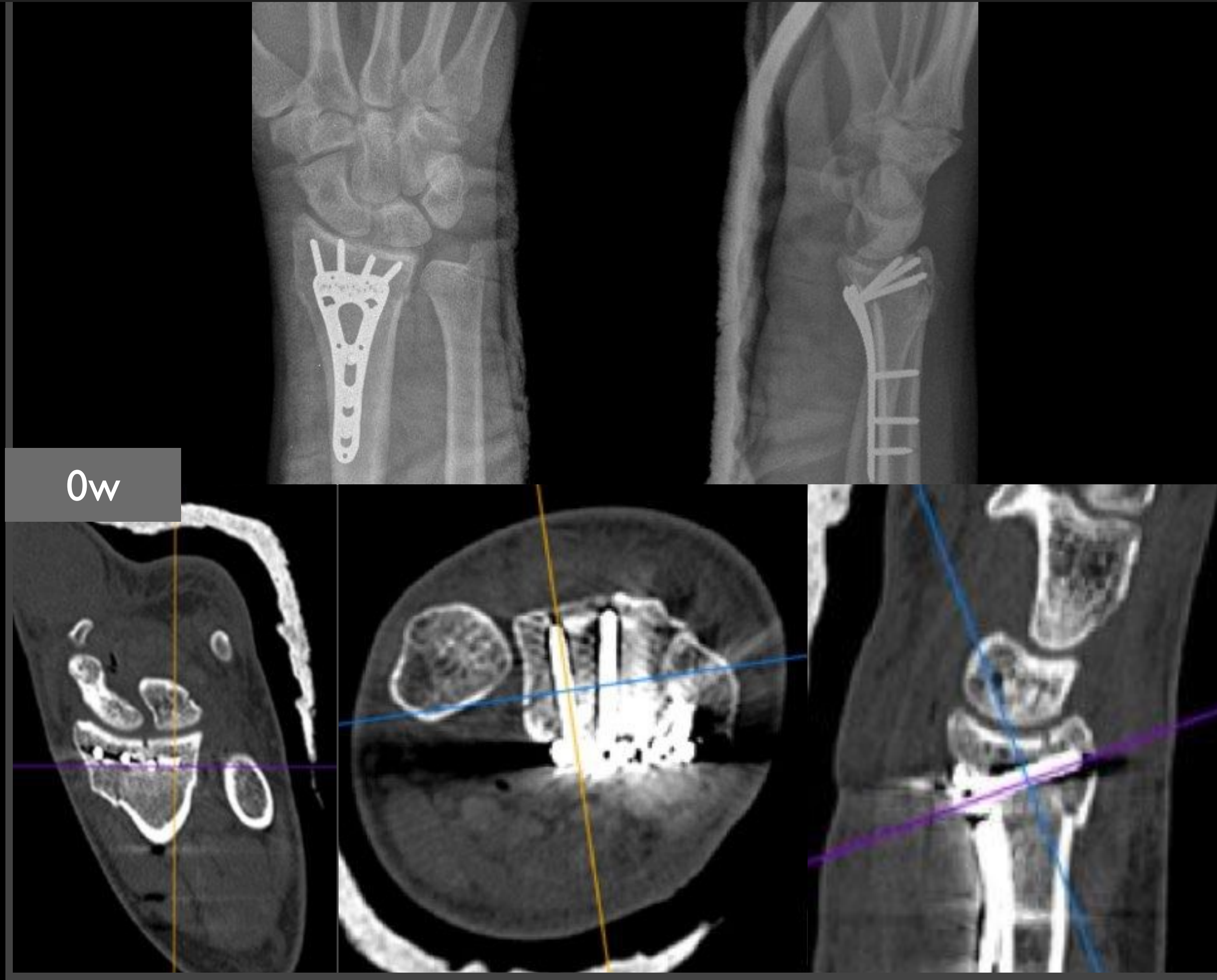

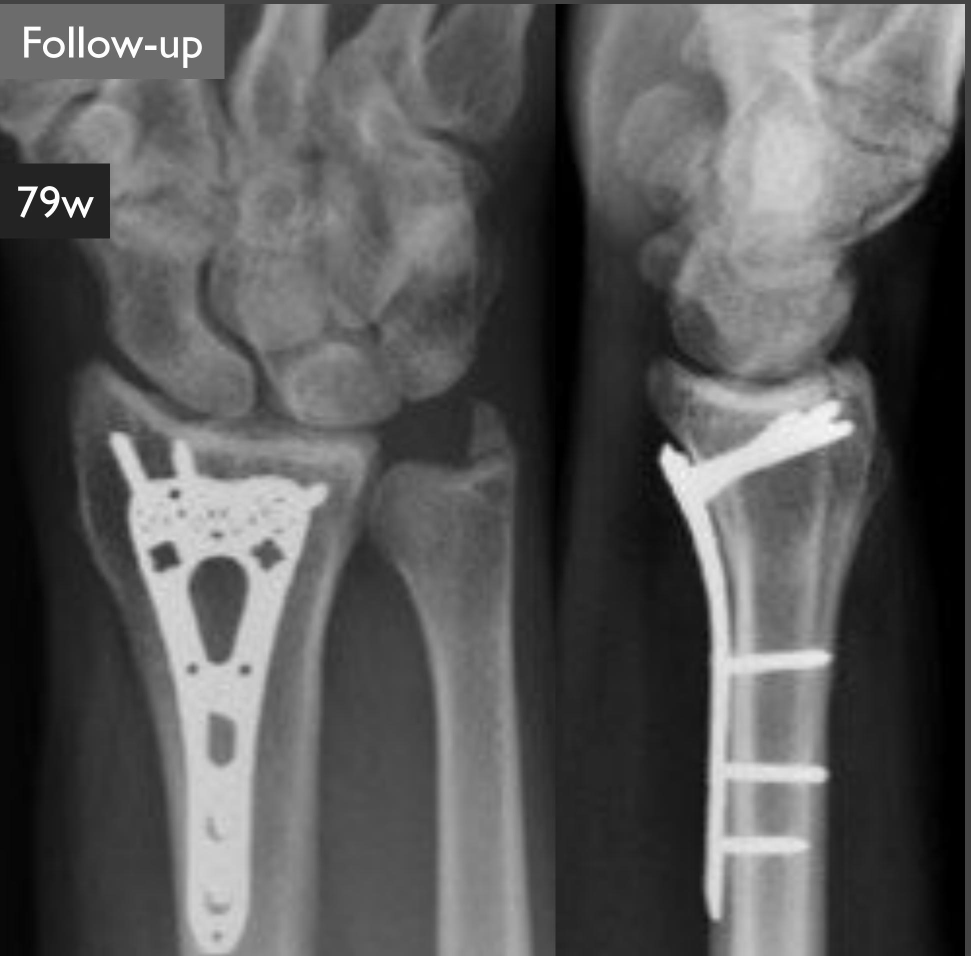

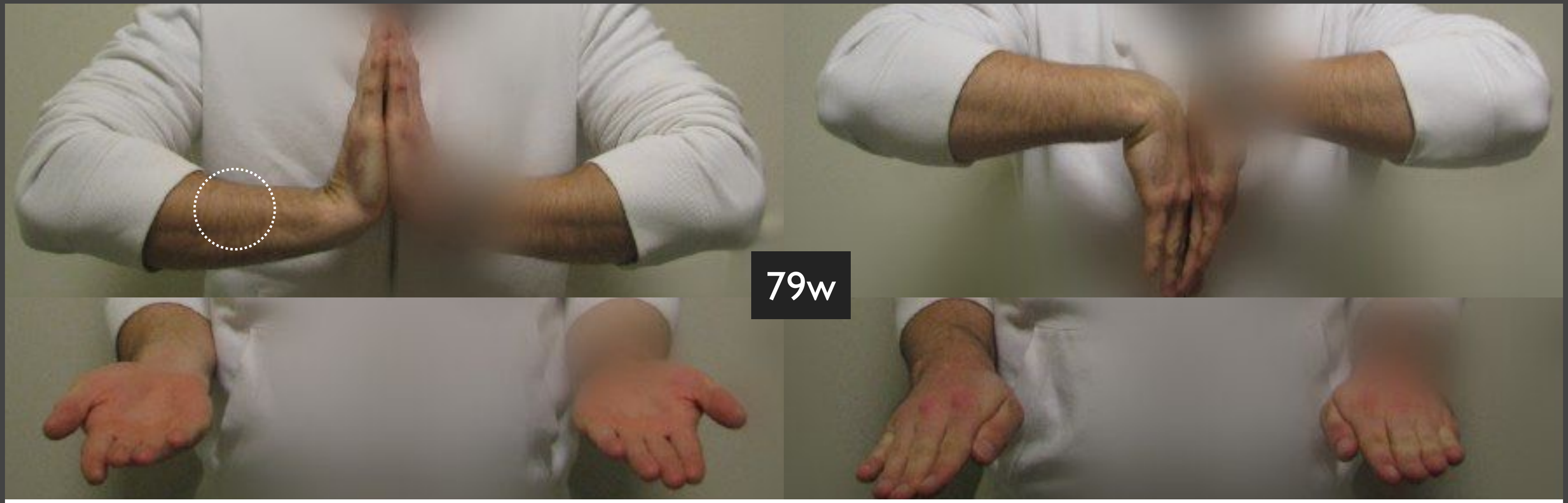

ICUC Score ***Functional Limitation:*** **1** (0-4) - ***Pain:*** **1** (0-4)

Quick DASH = 5

ICUC® ID: 23-DC-100 / 30y Male

*Complete case data can be accessed by clicking the **ICUC ID***

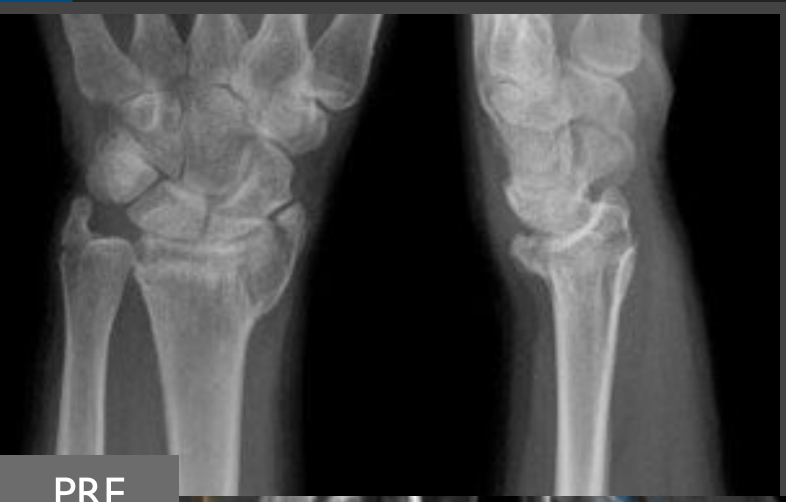

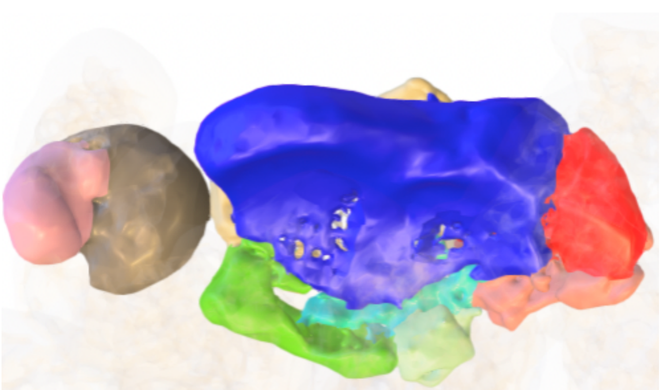

3D Colored Model

PRE

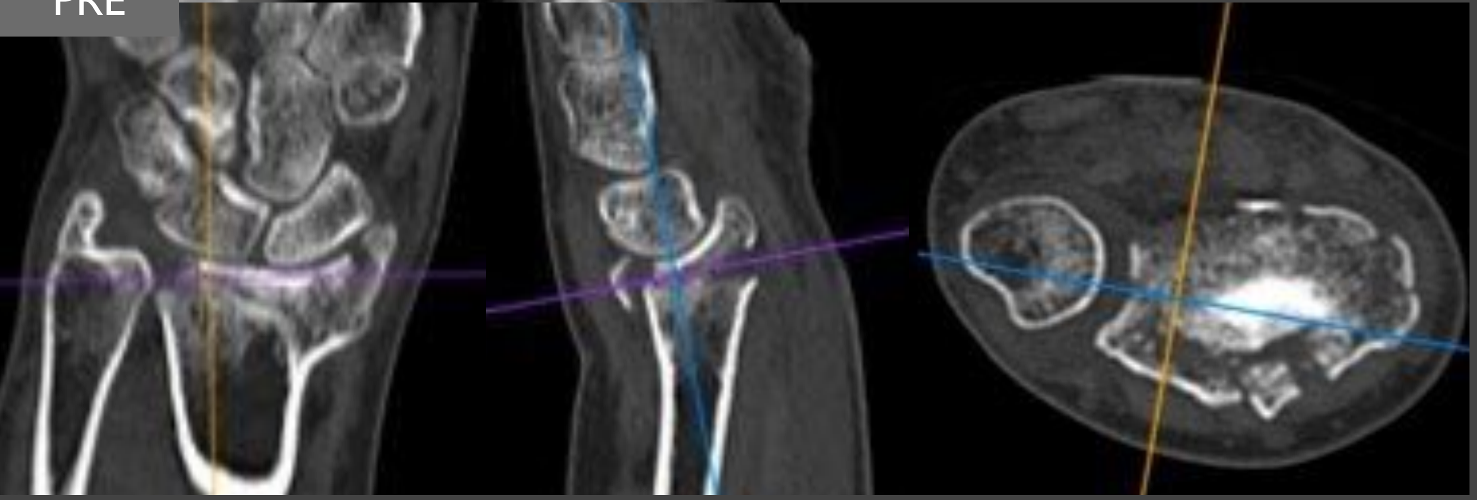

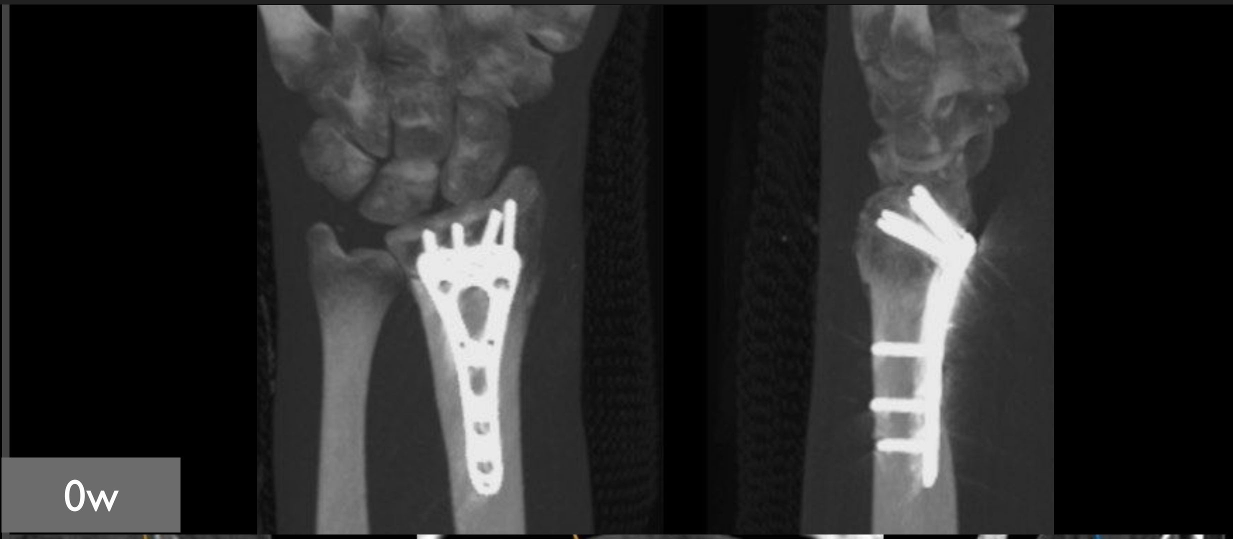

0w

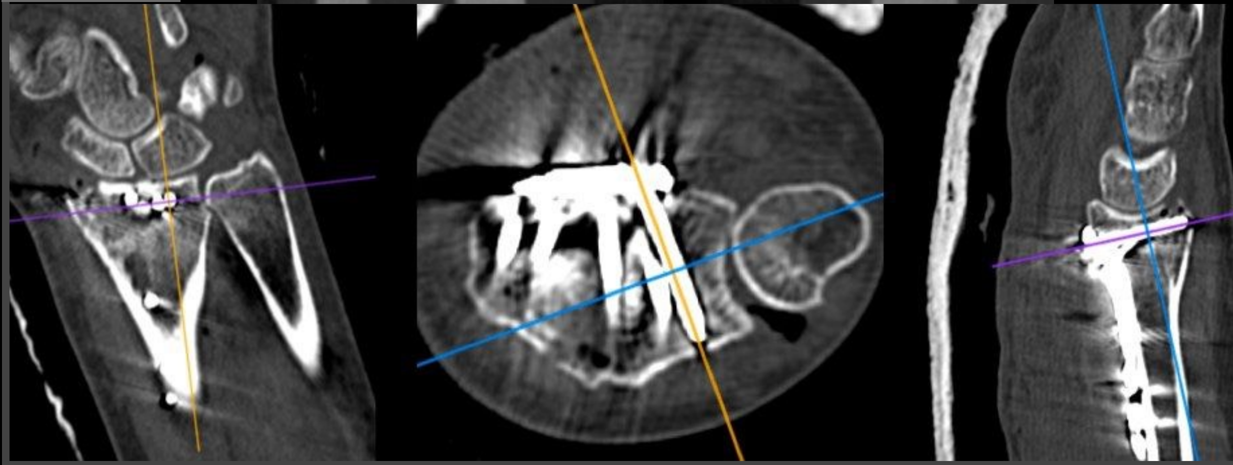

Follow-up

28w

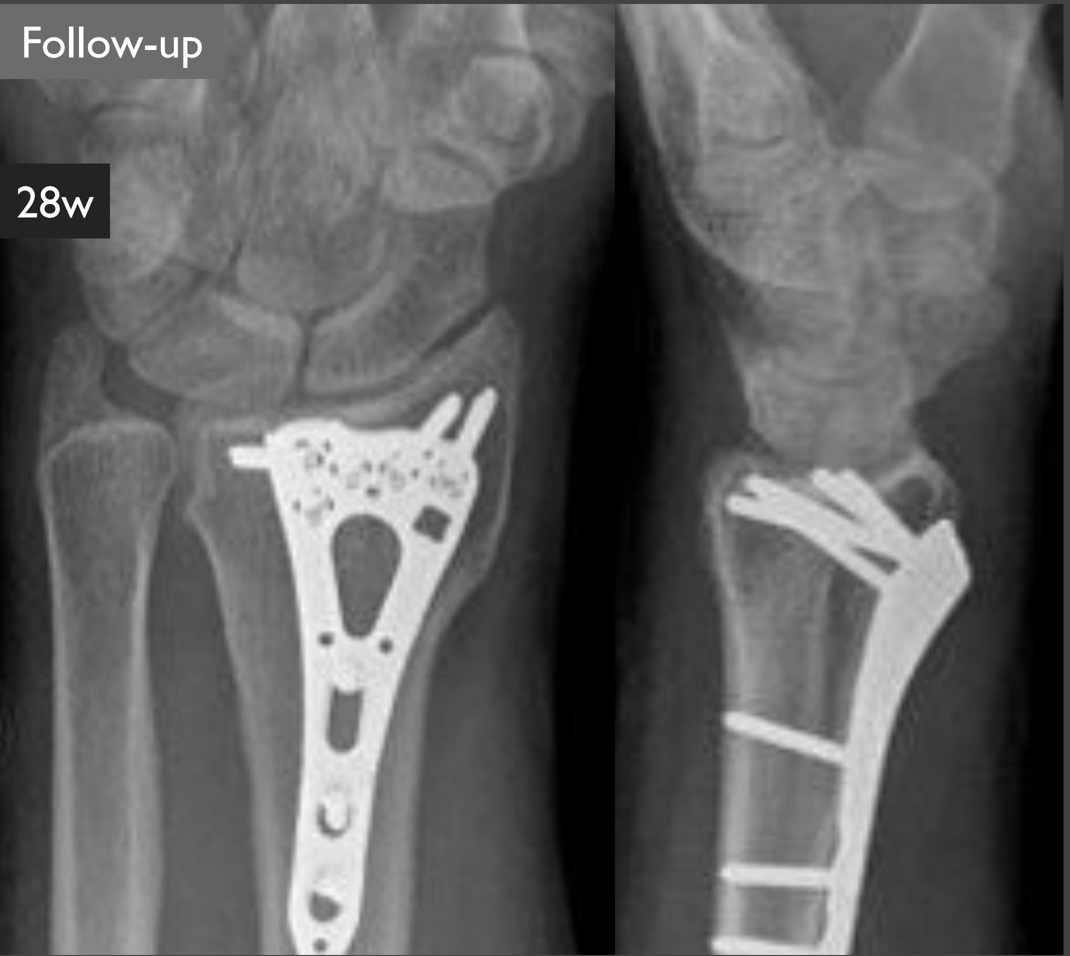

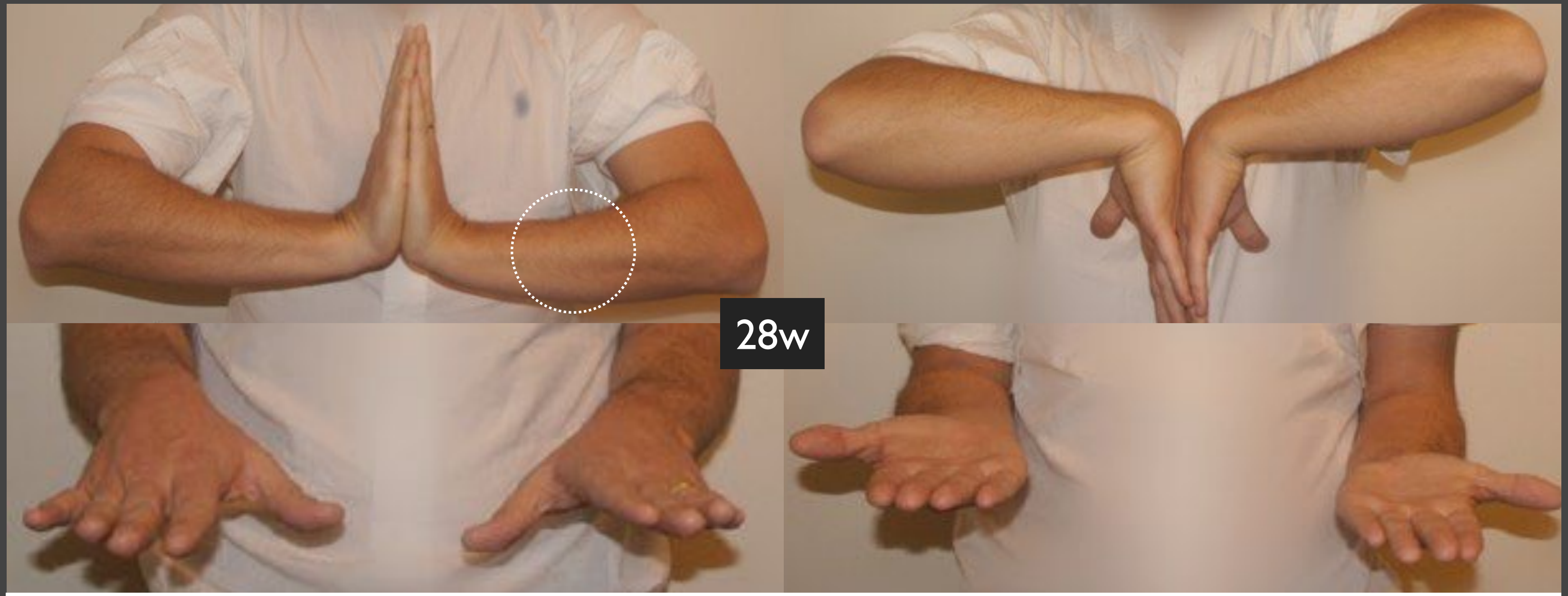

28w

ICUC Score ***Functional Limitation:* 1** (0-4) - ***Pain:* 2** (0-4)

ICUC Score at 455w Functional limitation: 0 Pain: 0

Quick DASH = 0

ICUC® ID: 23-DC-401 / 55y Female

Complete case data can be accessed by clicking the ***ICUC ID***

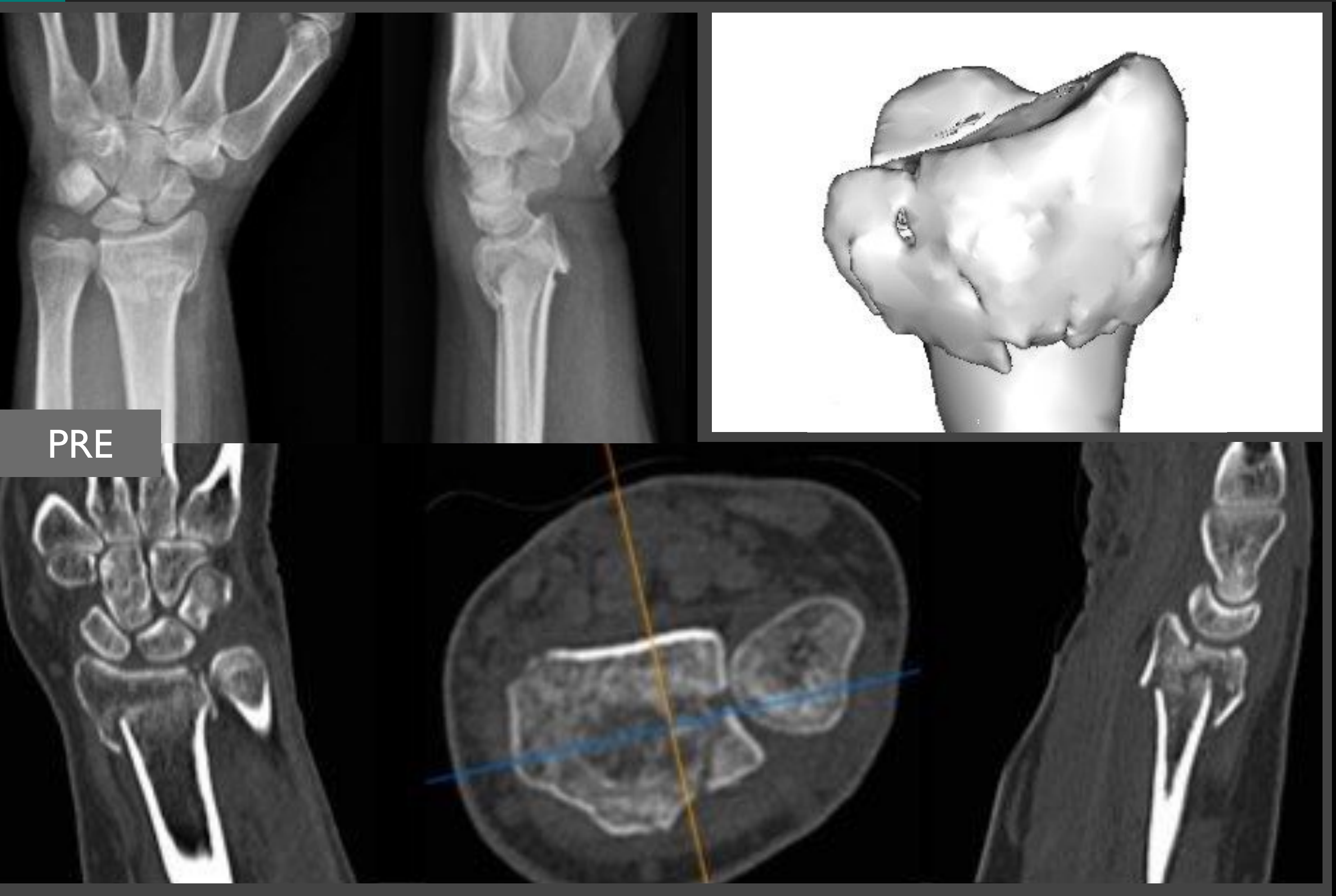

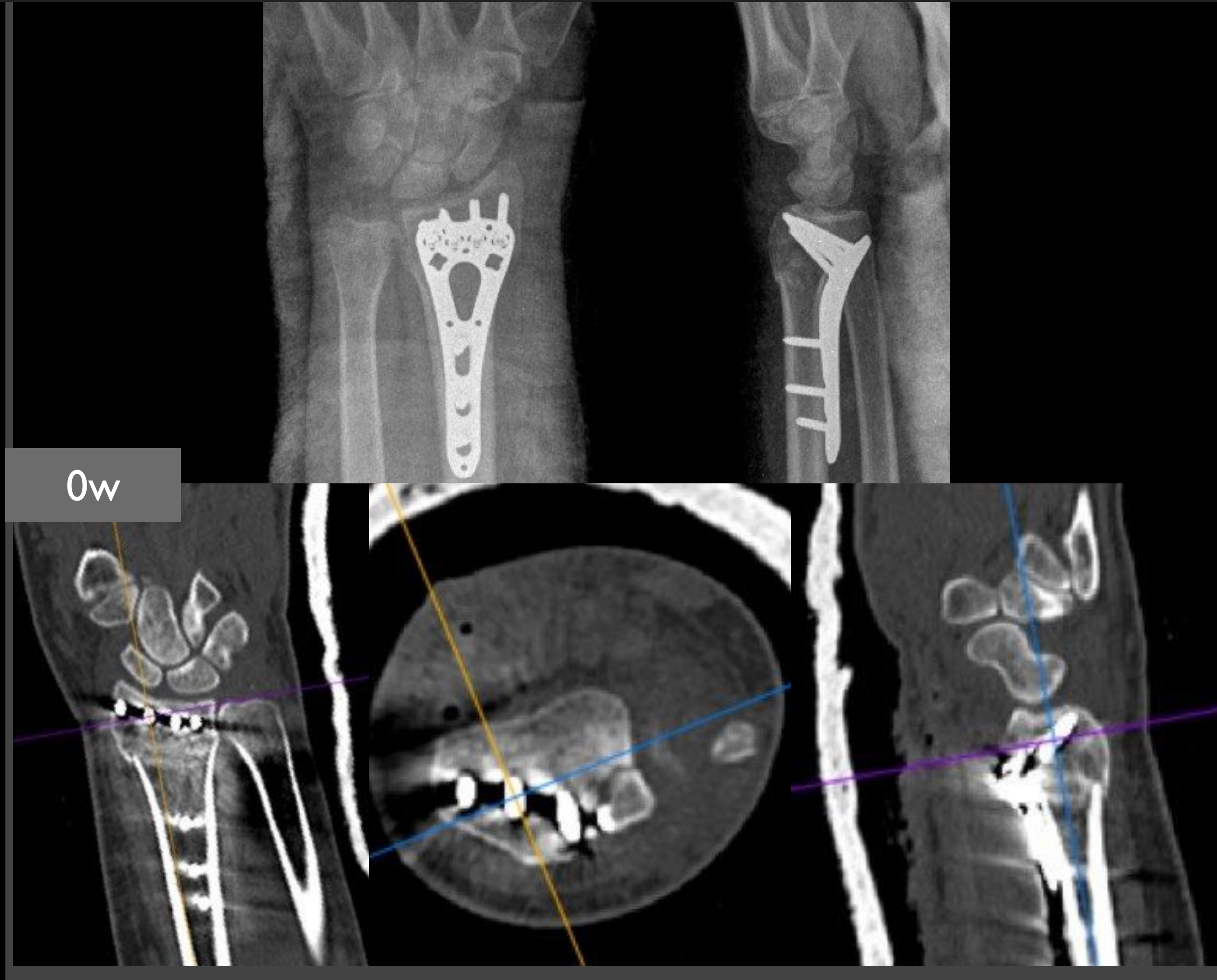

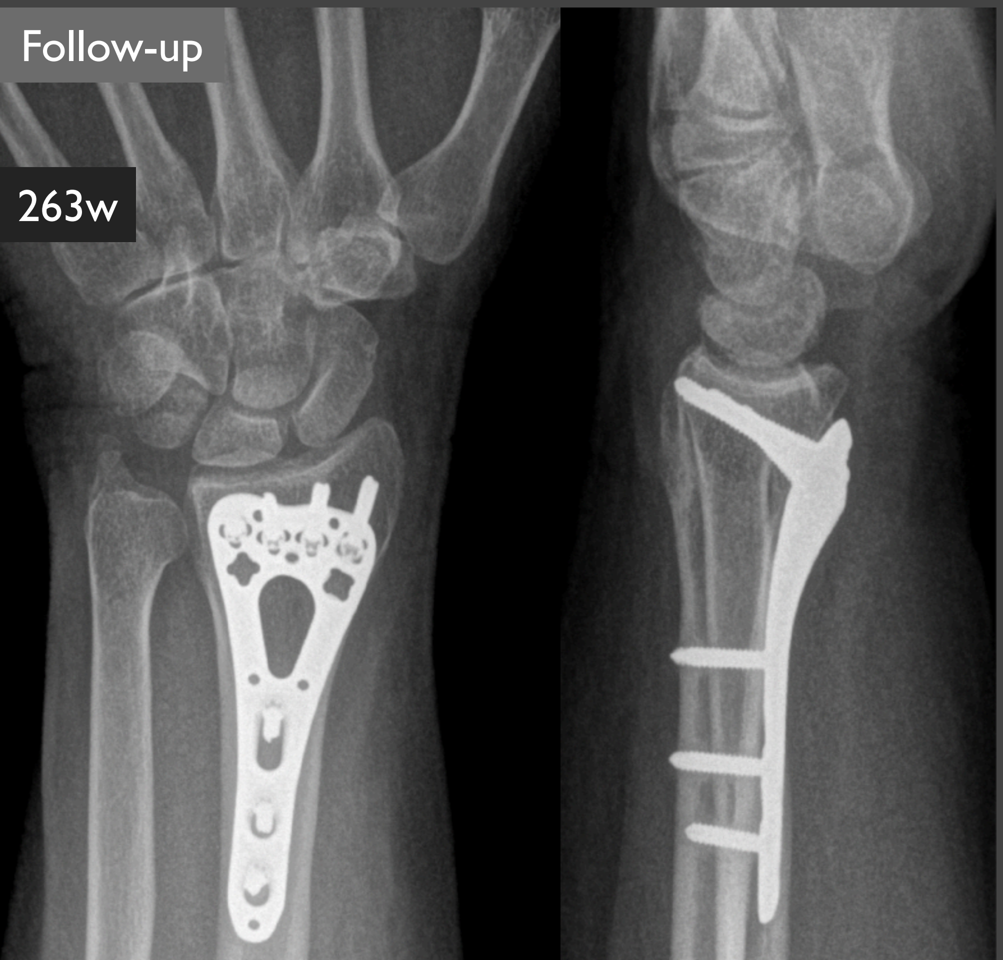

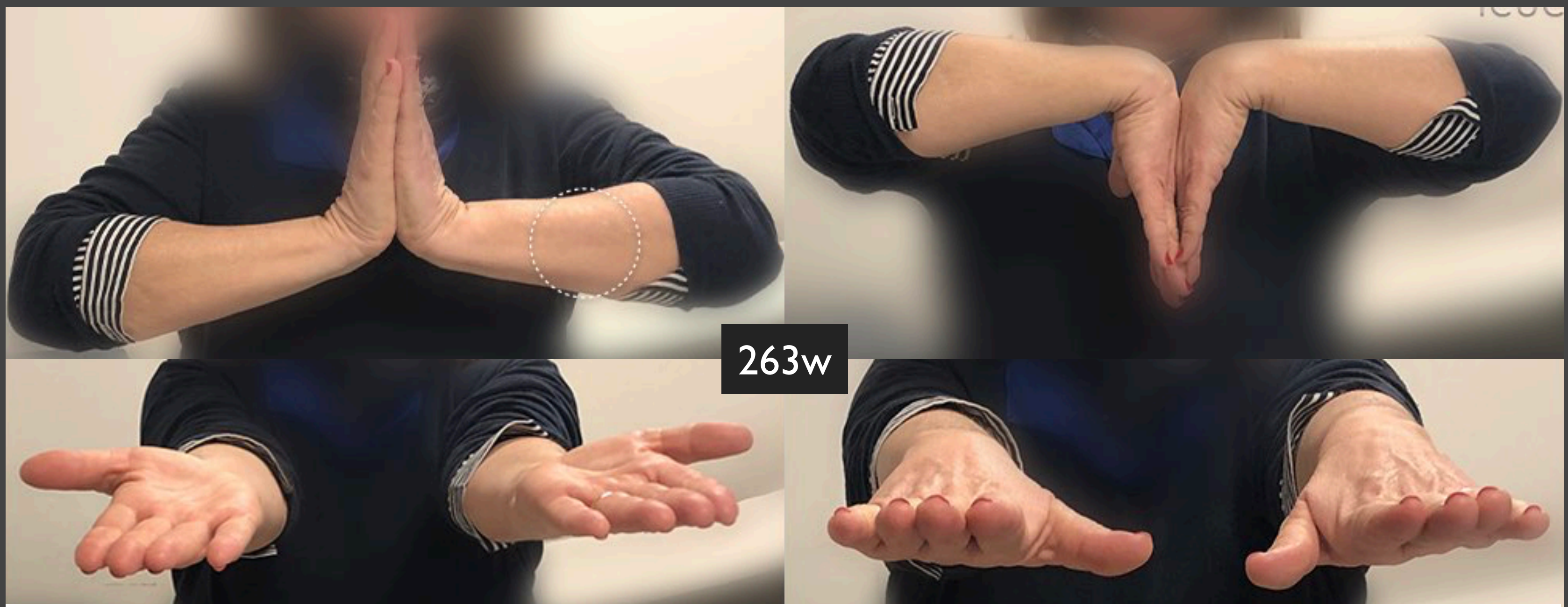

ICUC Score **Functional Limitation: 0** (0-4) - **Pain: 1** (0-4)

ICUC Score at 474w Functional limitation: 1 Pain: 0

Quick DASH = 0

ICUC® ID: 23-DC-208 / 60y Female

Complete case data can be accessed by clicking the ***ICUC ID***

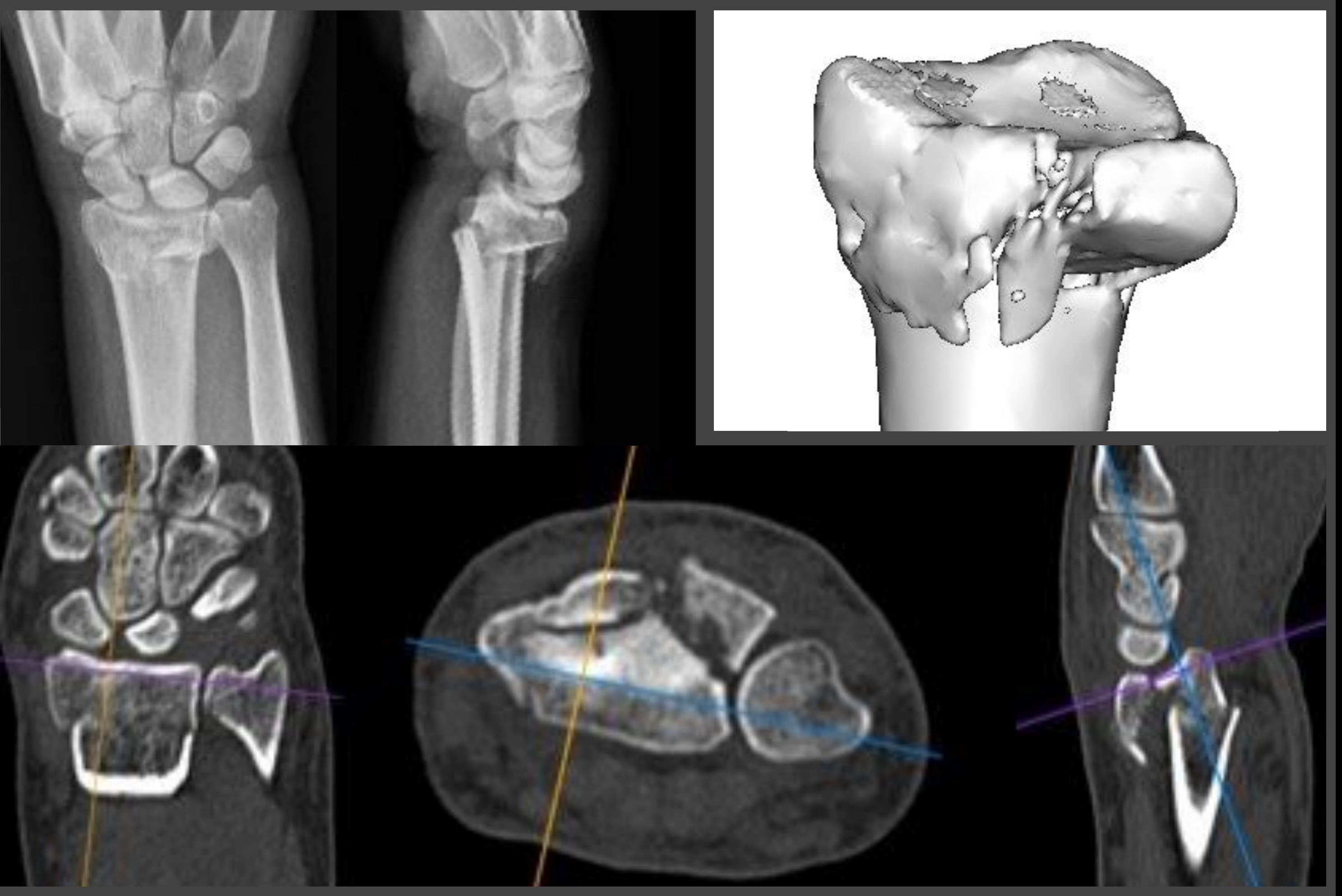

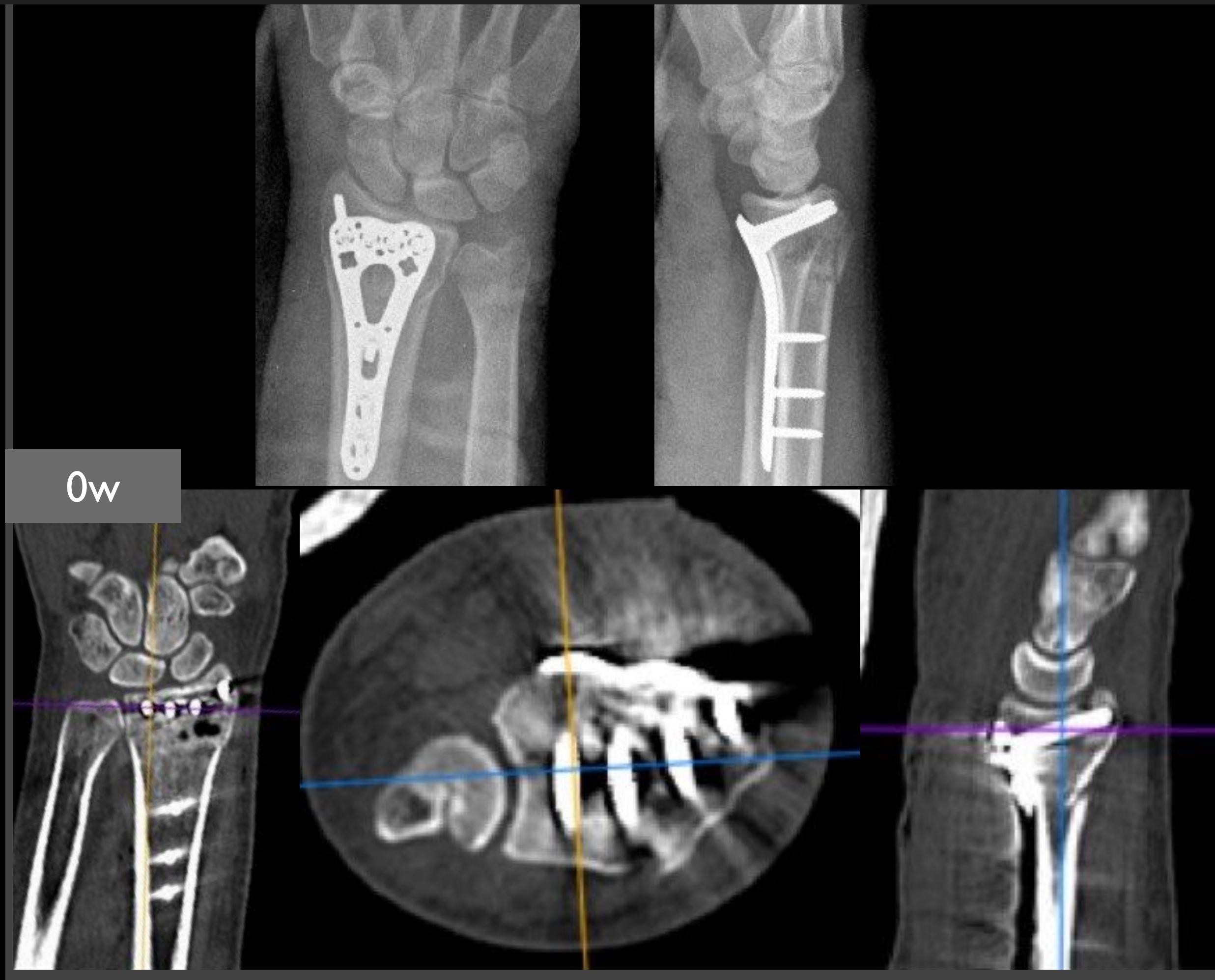

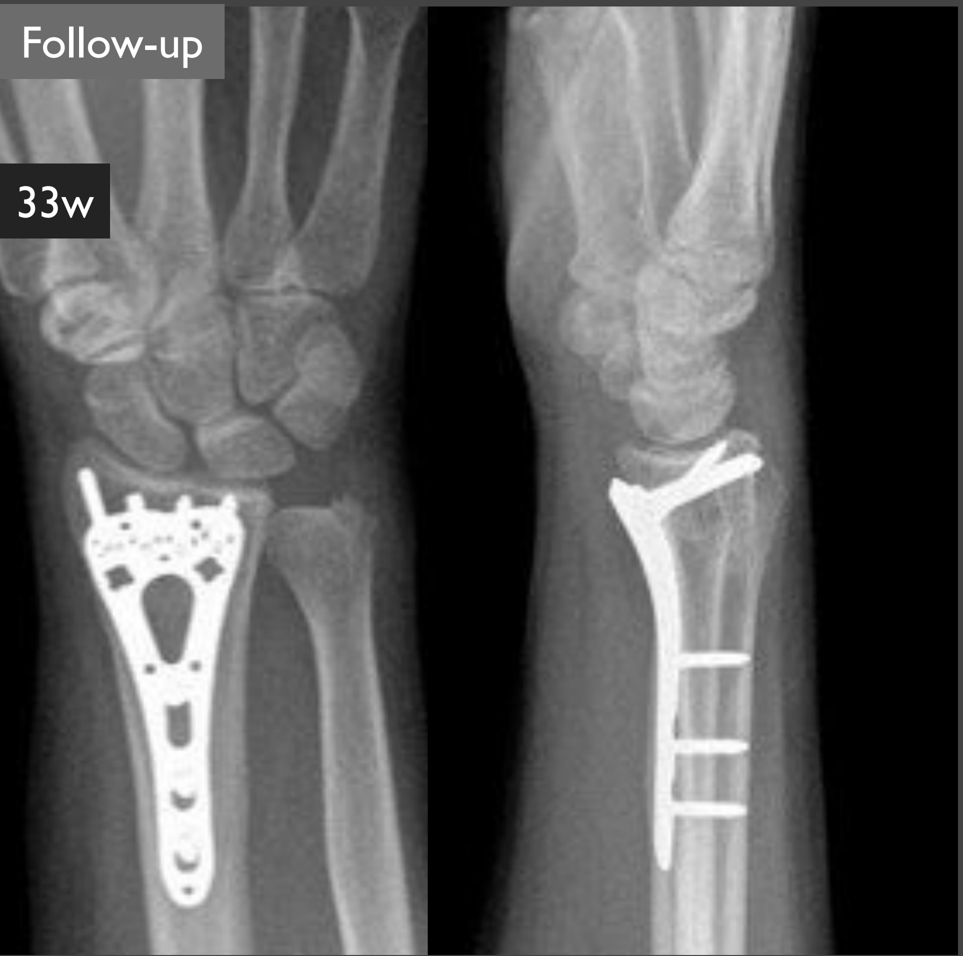

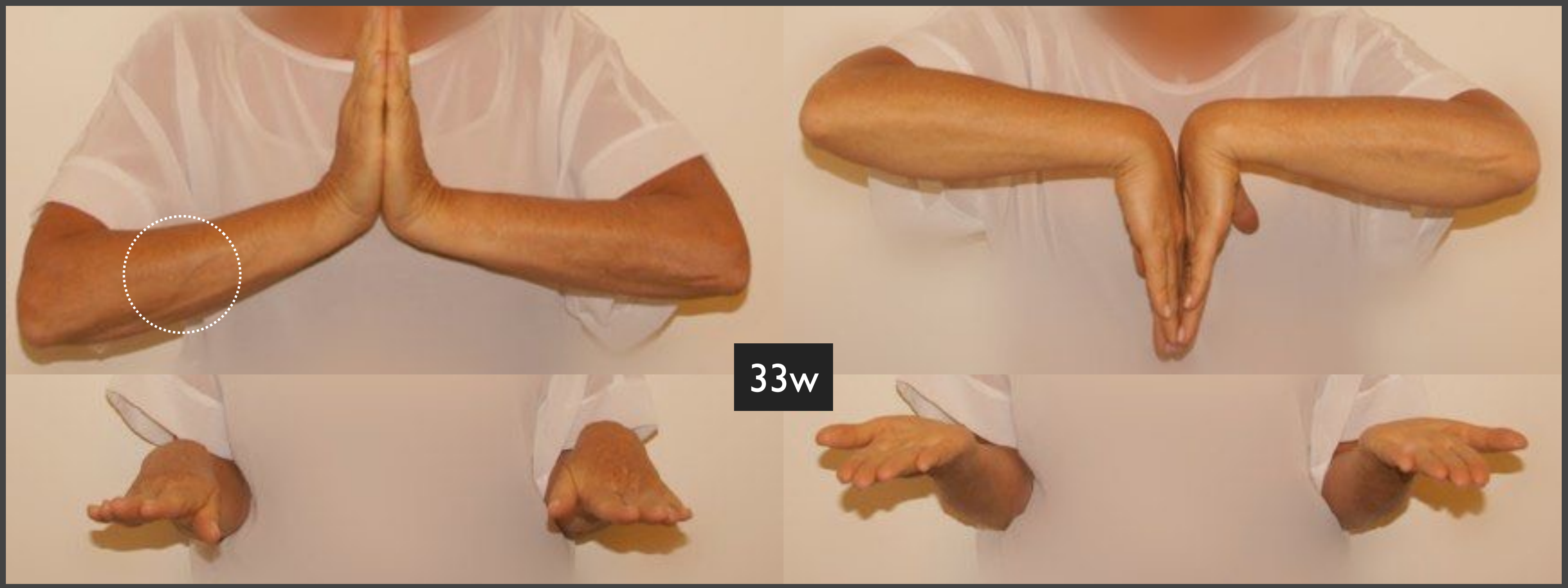

ICUC Score at 477w Functional limitation: 0 Pain: 0

Quick DASH = 0

ICUC® ID: 23-DC-165 / 55y Female

*Complete case data can be accessed by clicking the **ICUC ID***

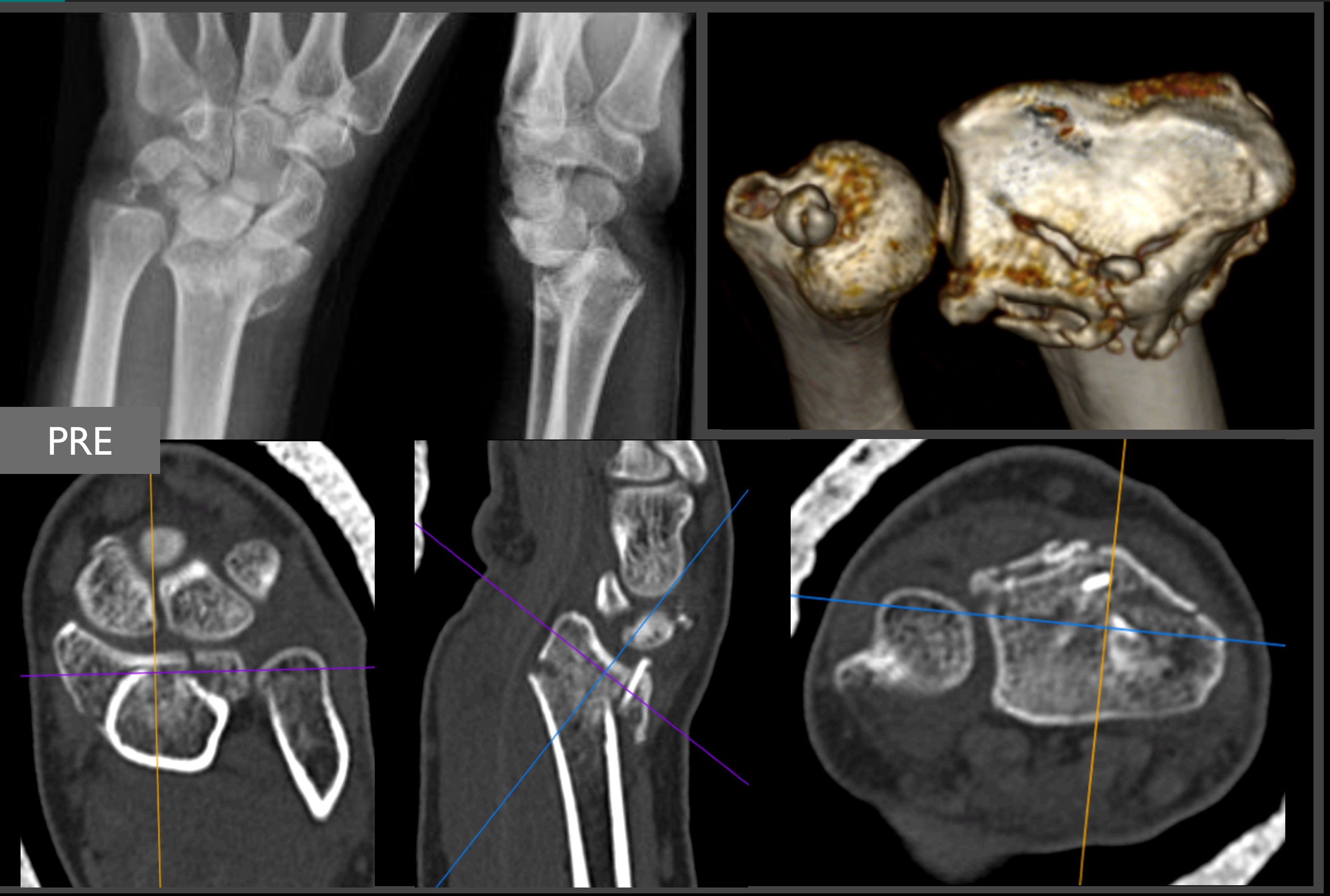

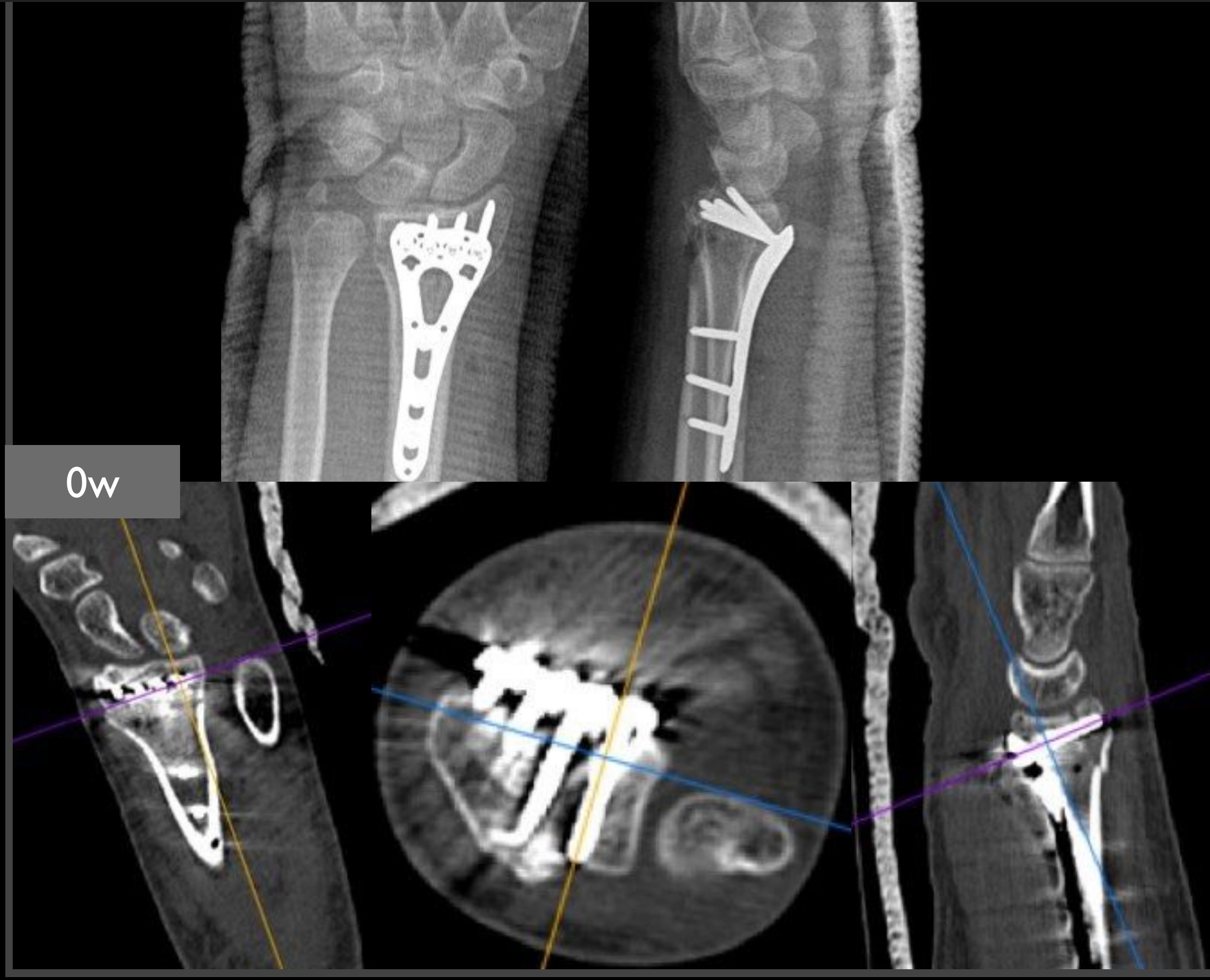

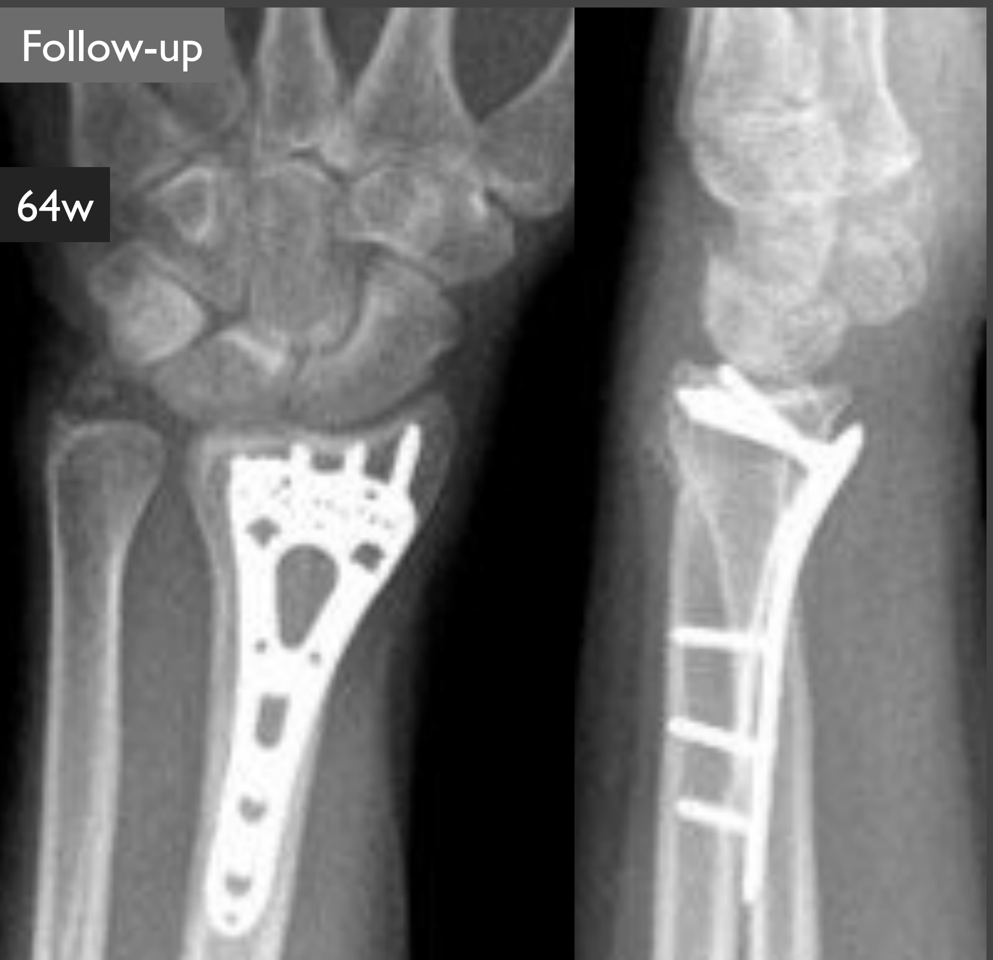

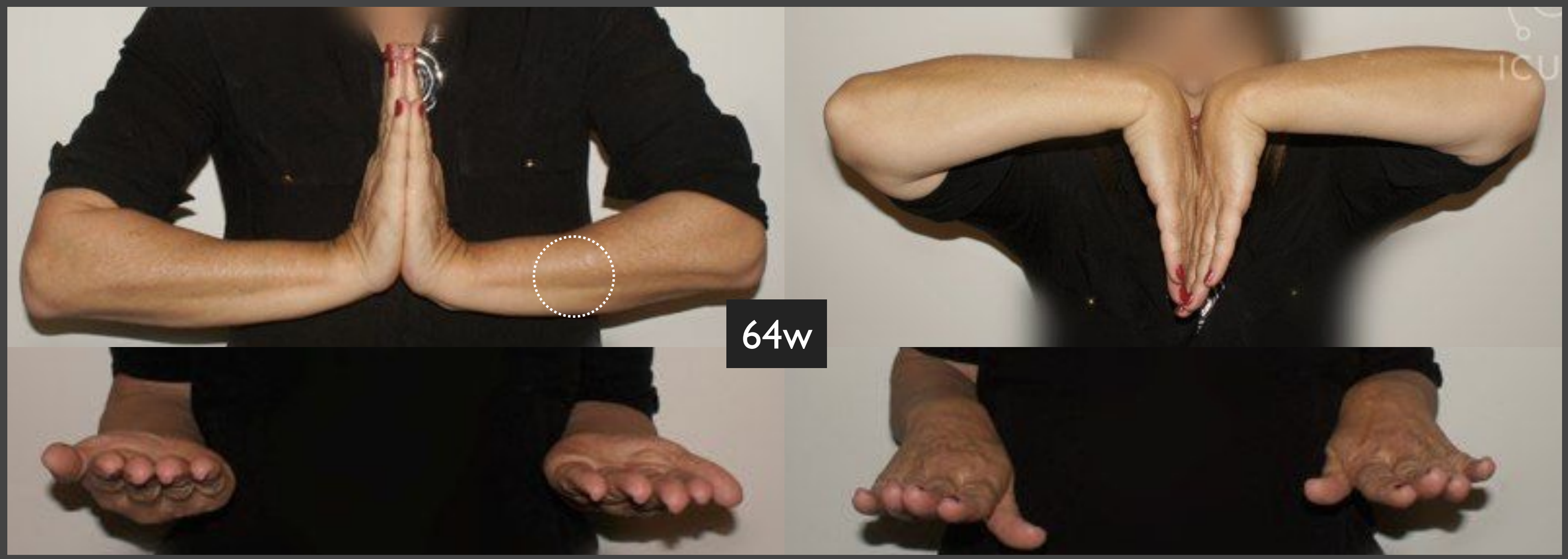

ICUC Score at 186w Functional limitation: 0 Pain: 0

Quick DASH = 4.5

ICUC® ID: 23-DC-373 / 55y Male

Complete case data can be accessed by clicking the ***ICUC ID***

PRE

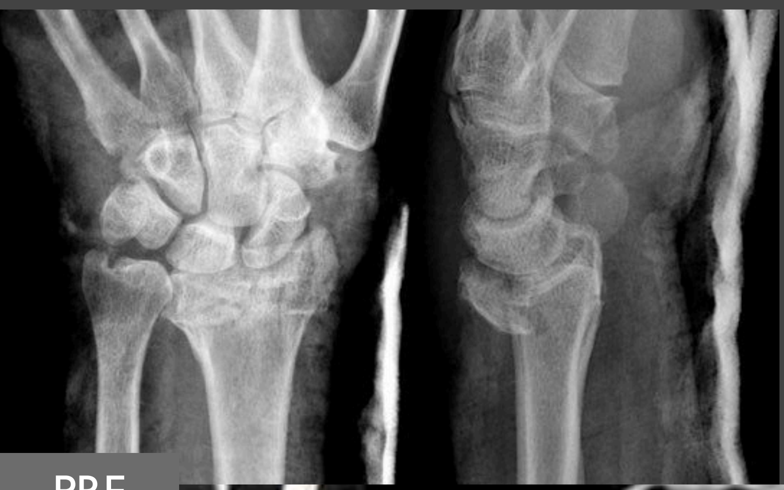

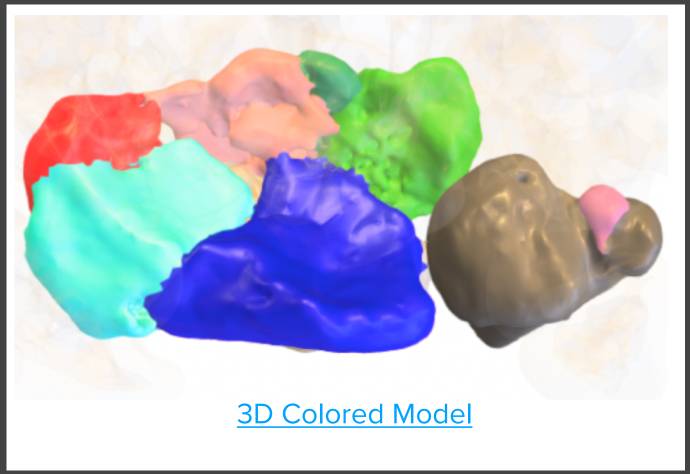

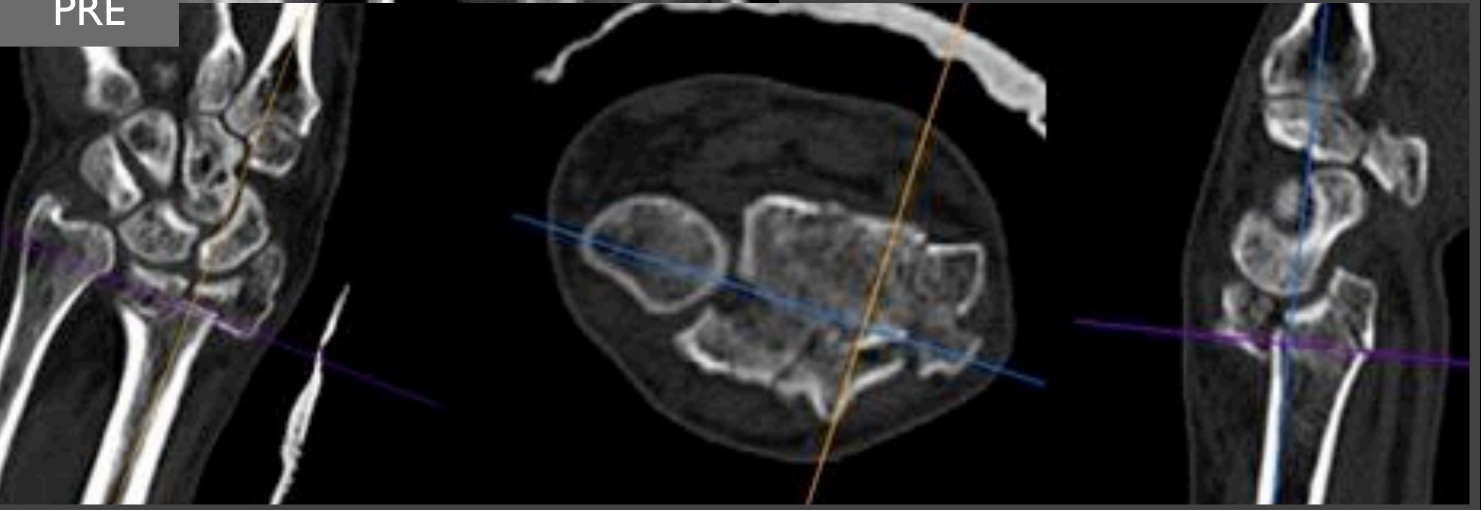

0w

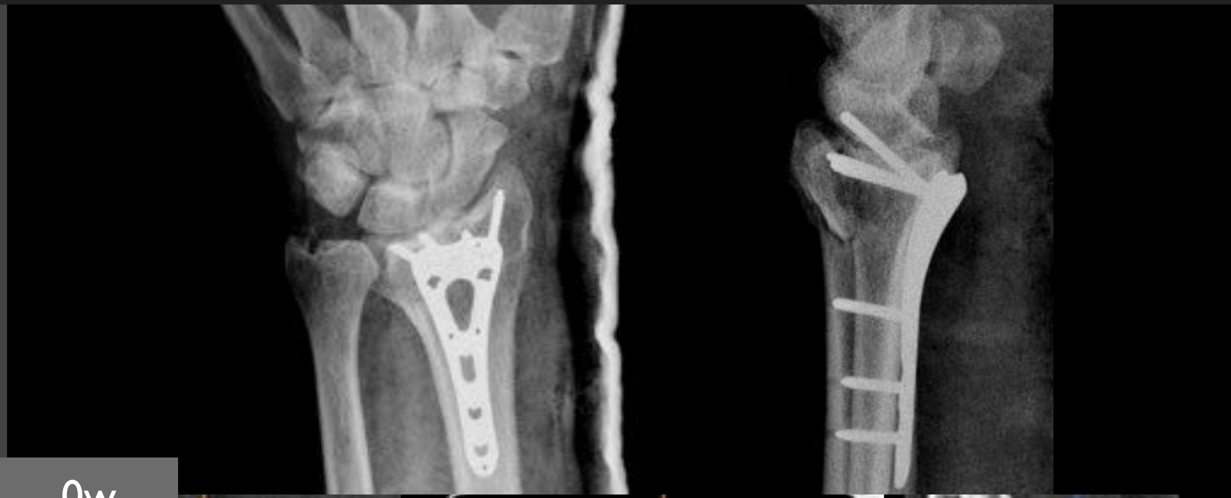

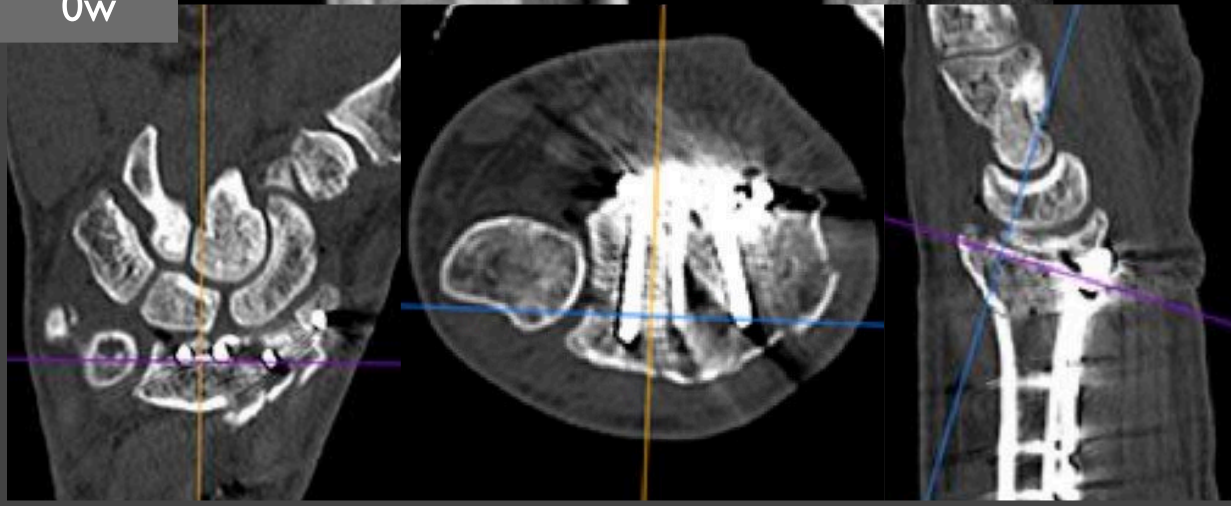

Follow-up

179w

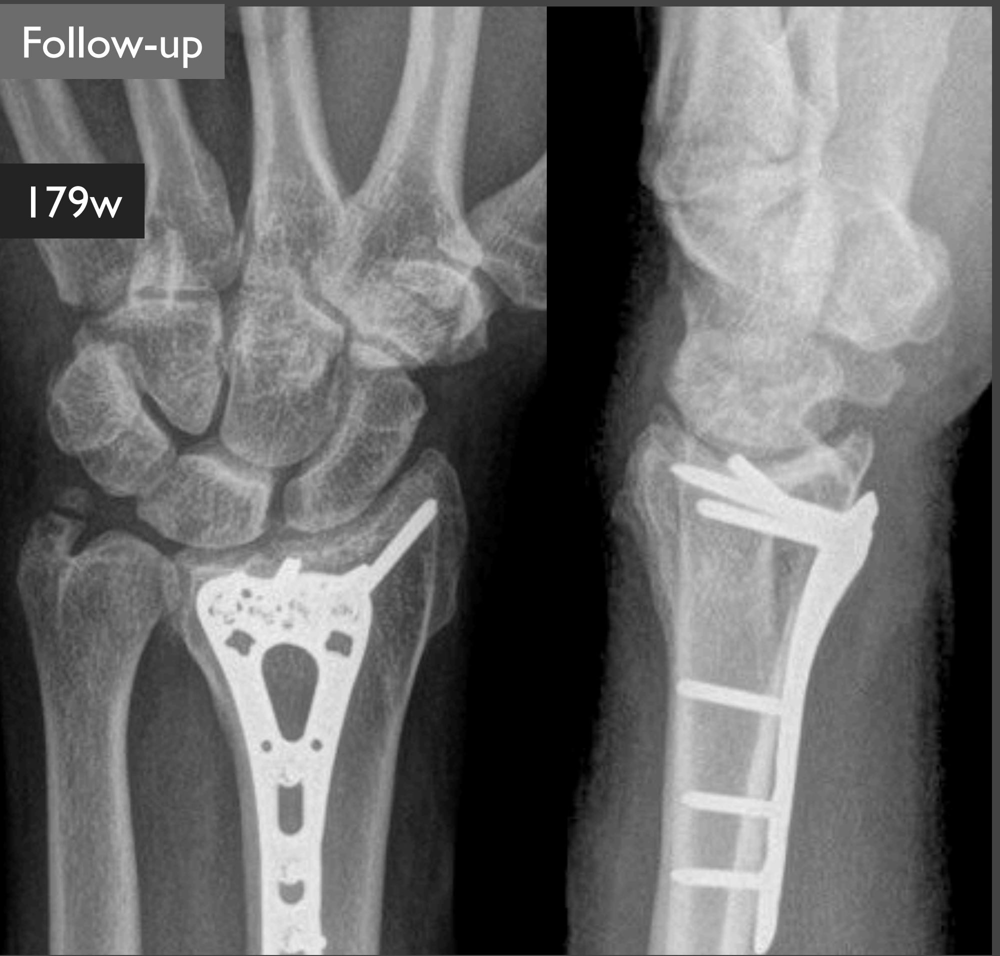

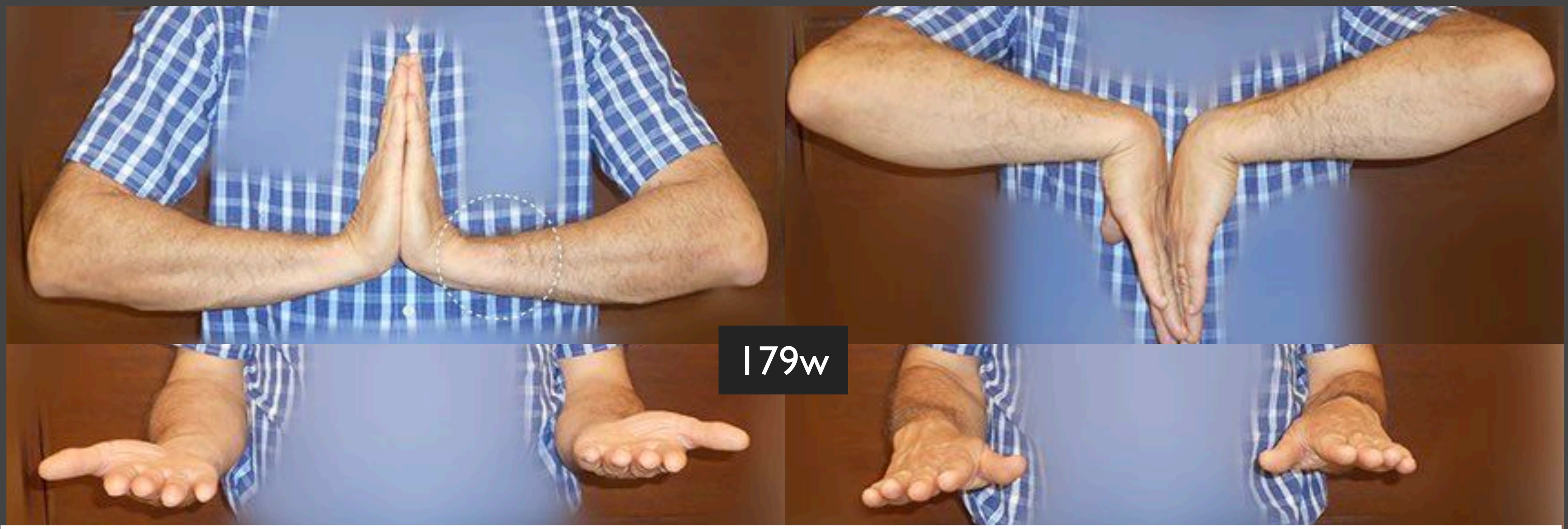

ICUC Score **Functional Limitation: 1** (0-4) - **Pain: 2** (0-4)

ICUC Score at 424w Functional limitation: 0 Pain: 1

Quick DASH = 4.5

ICUC® ID: 23-DC-868 / 70y Male

Complete case data can be accessed by clicking the ***ICUC ID***

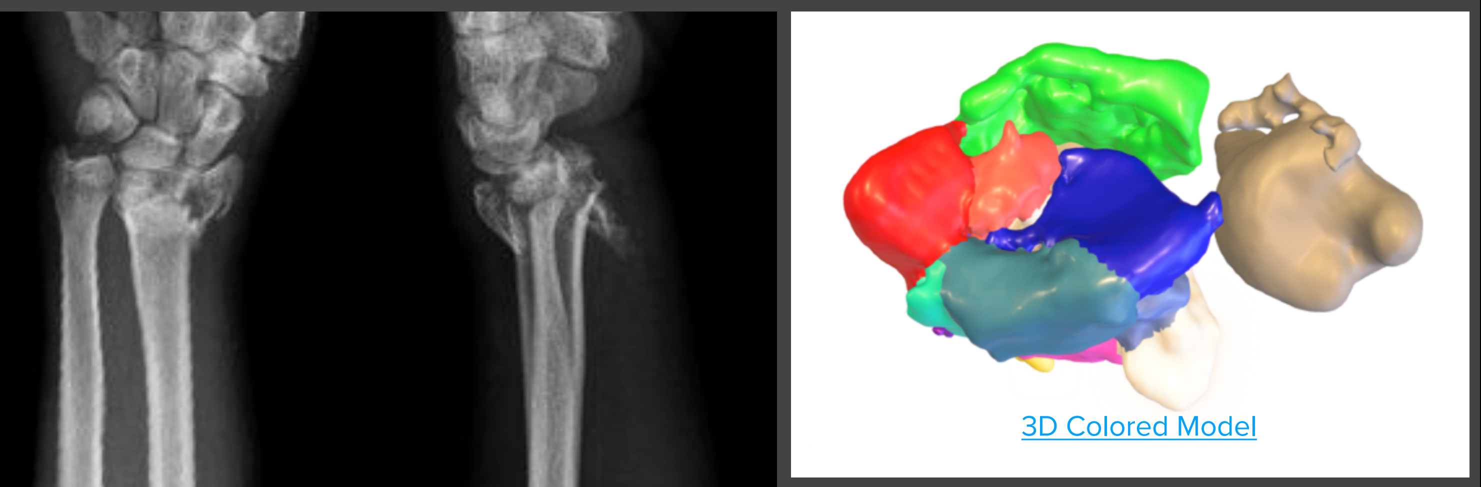

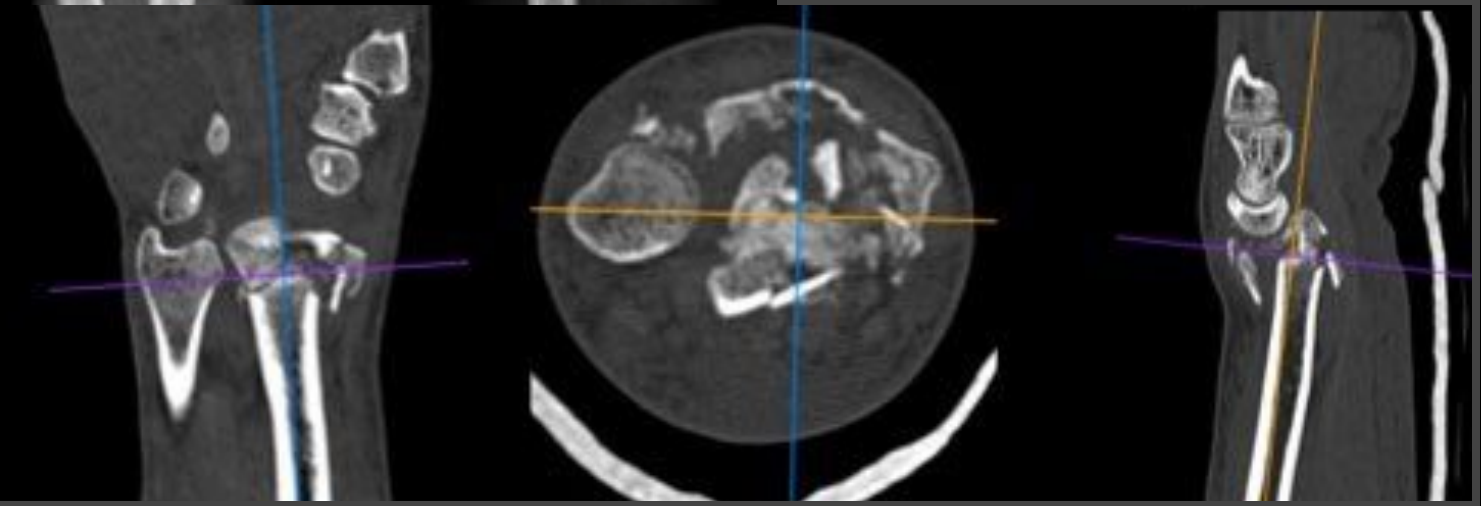

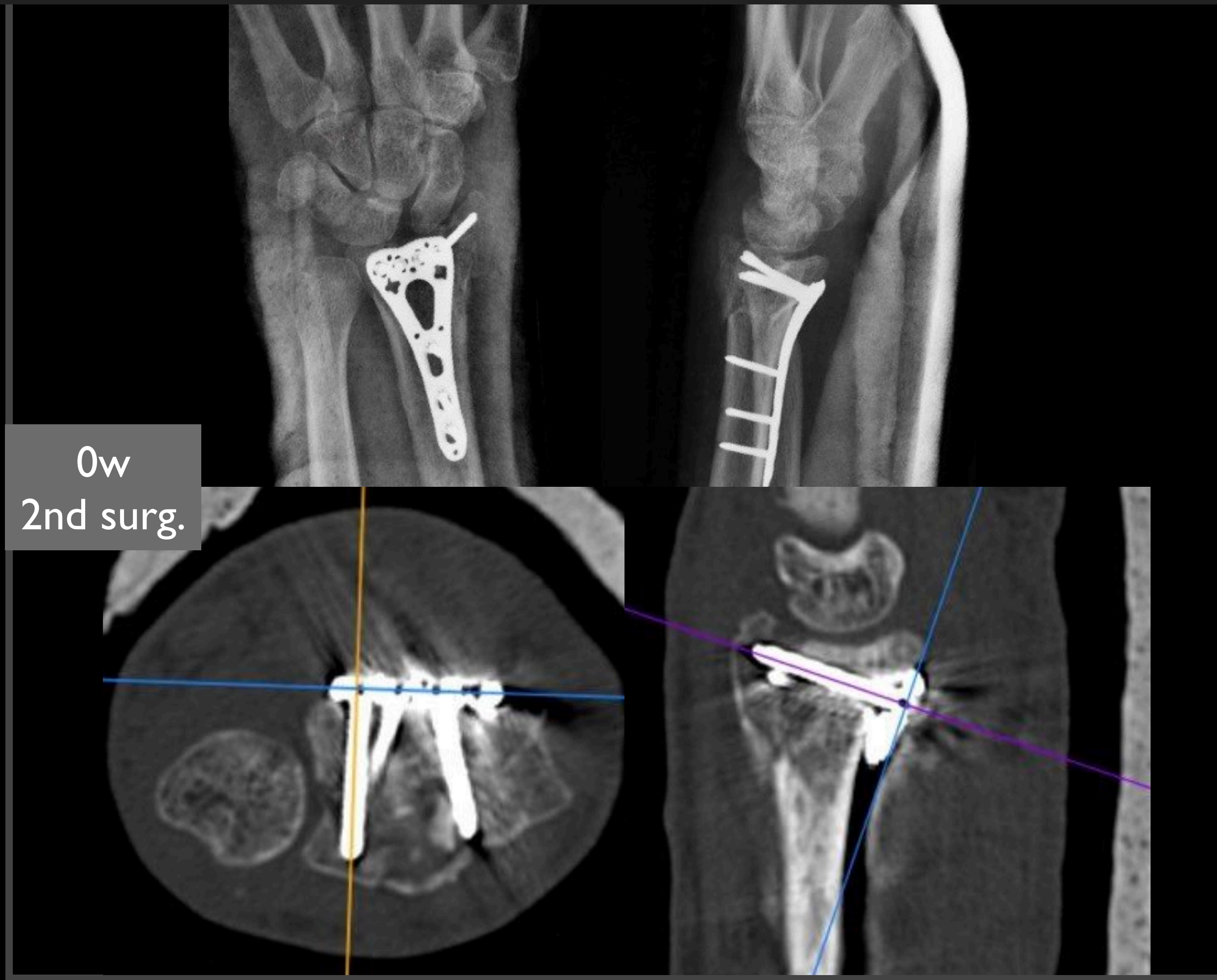

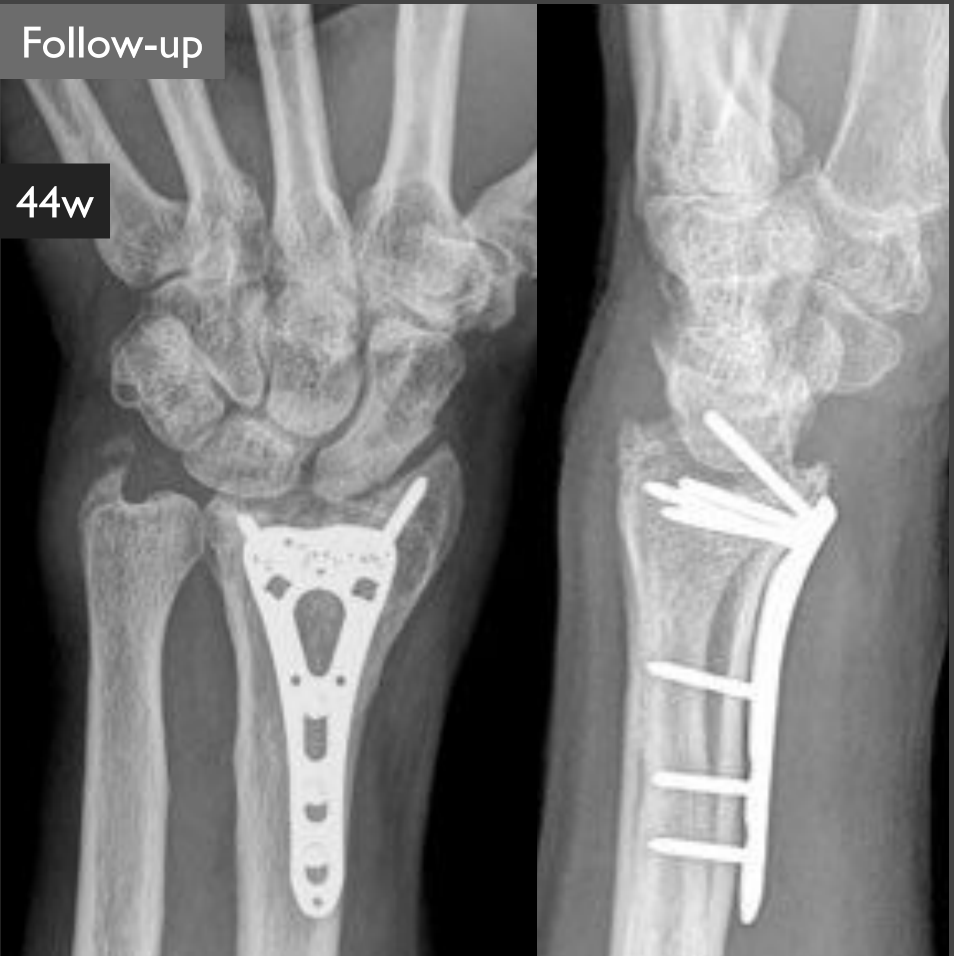

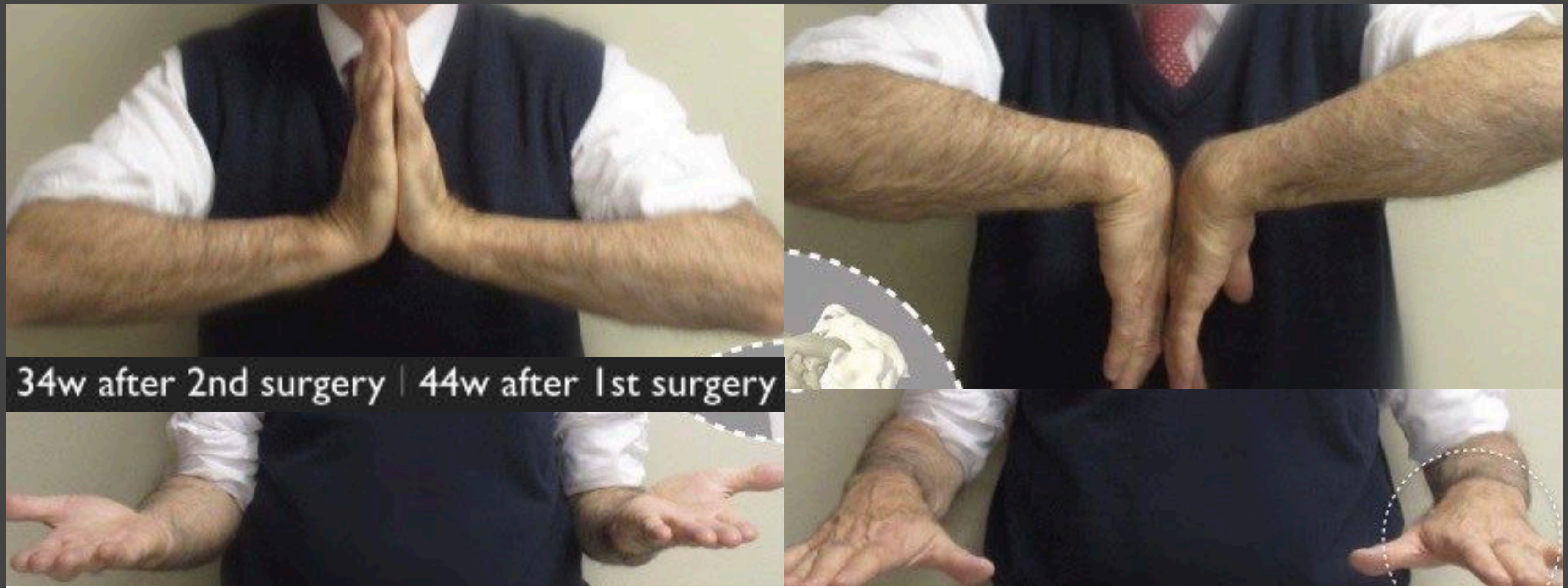

ICUC Score ***Functional Limitation:*** **1** (0-4) - ***Pain:*** **0** (0-4)

ICUC Score at 422w Functional limitation: 0 Pain: 0

Quick DASH = 0

ICUC® ID: 23-DC-617 / 45y Male

Complete case data can be accessed by clicking the ***ICUC ID***

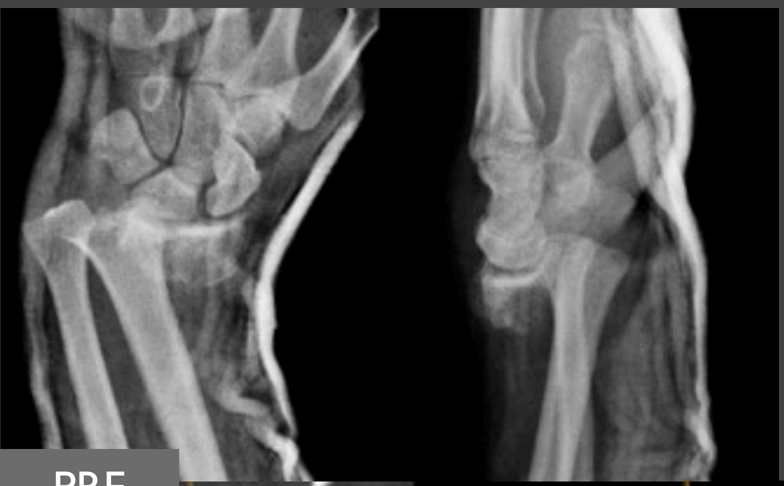

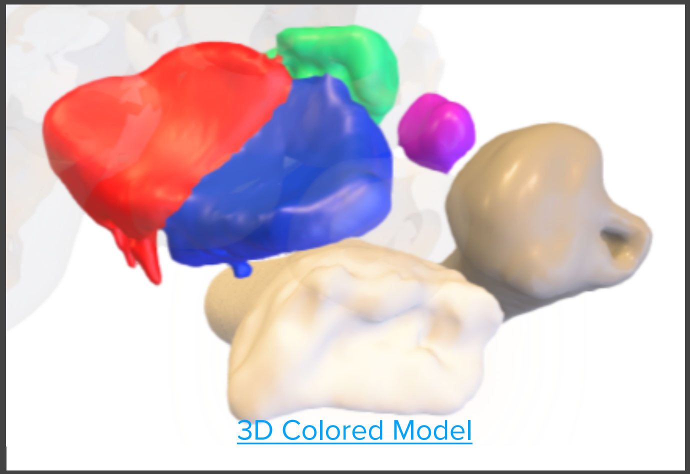

PRE

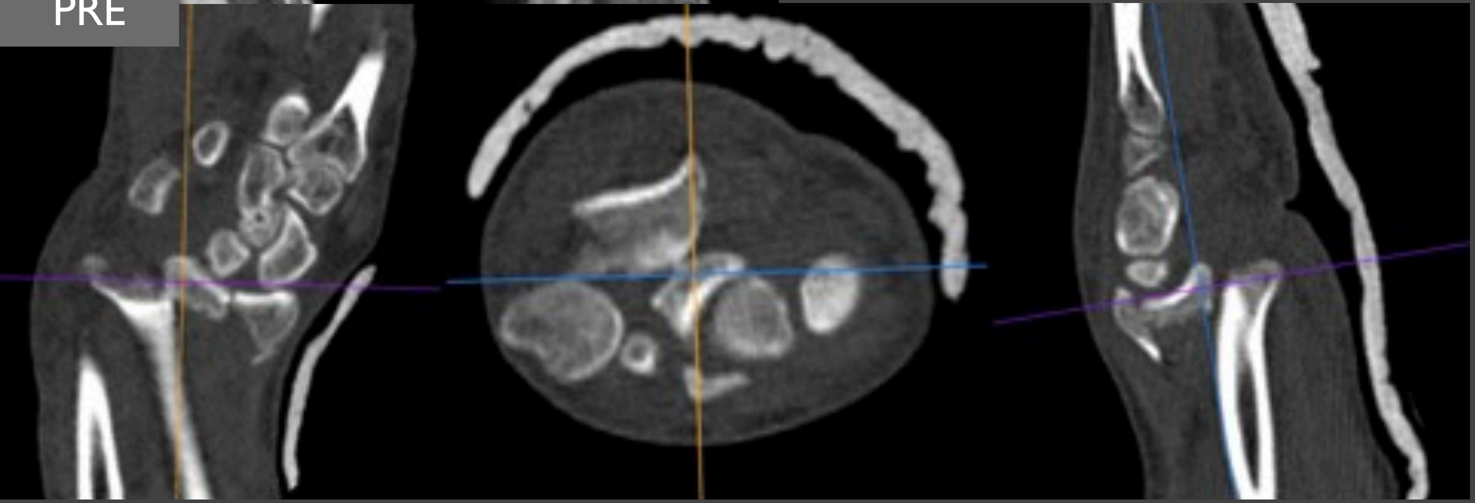

0w
2nd surg.

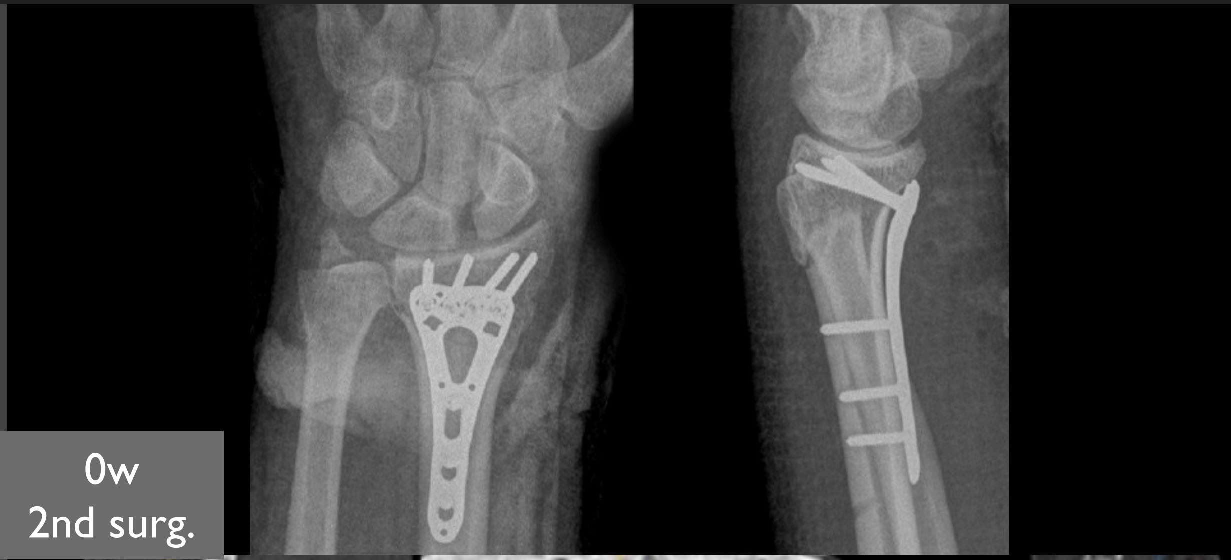

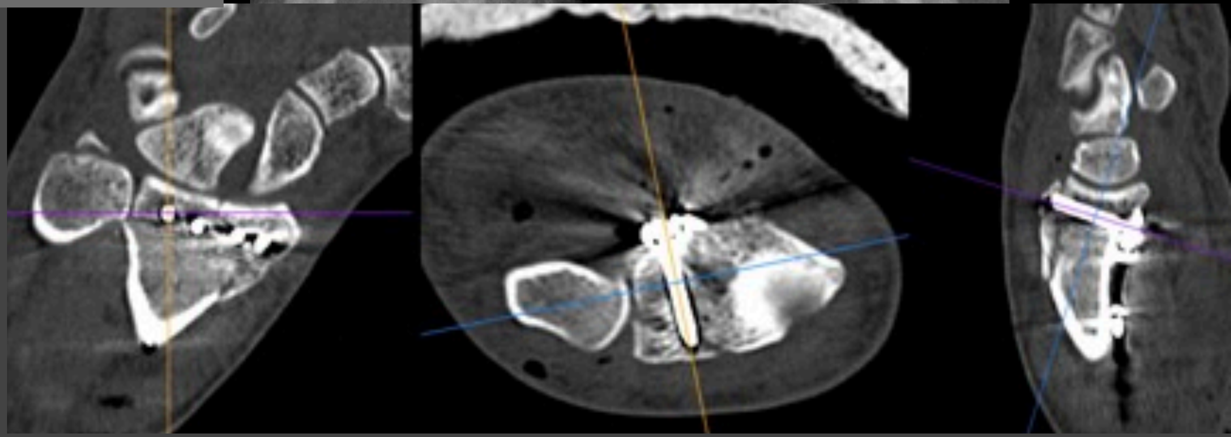

Follow-up

310w

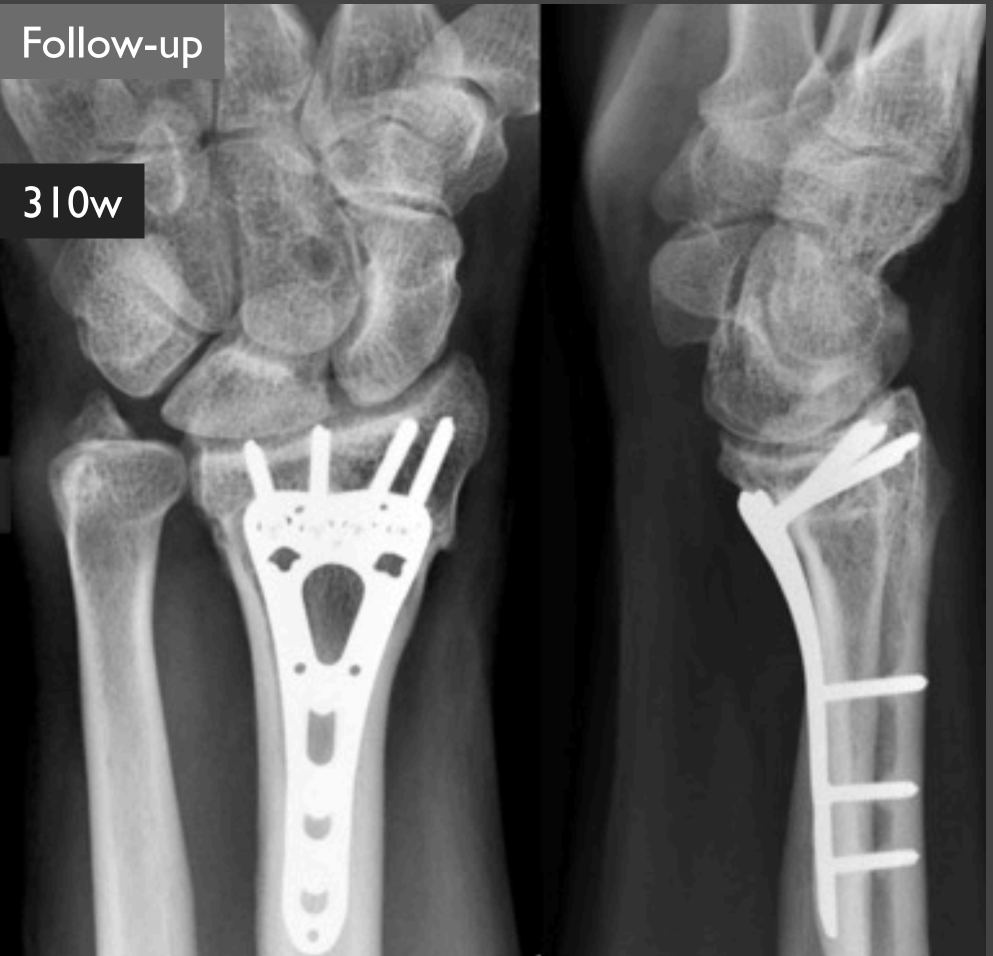

310w

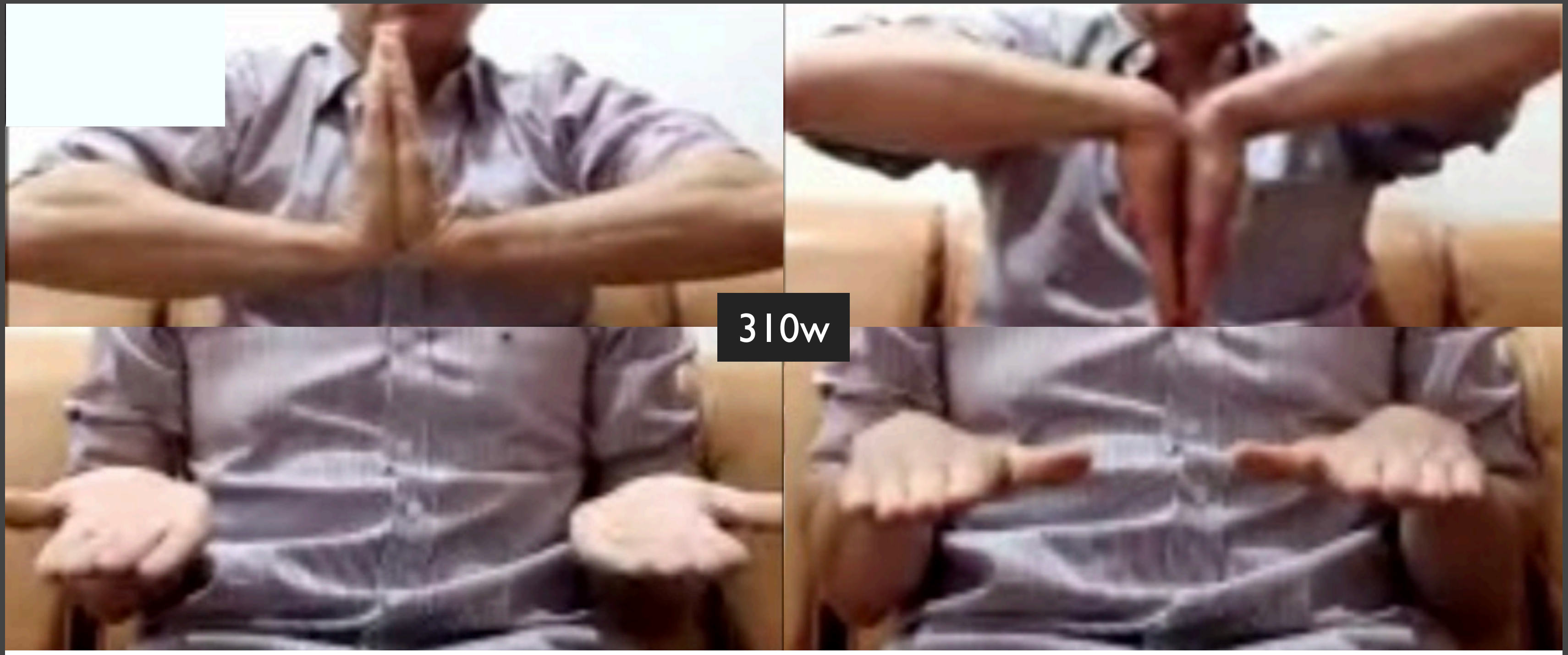

ICUC Score **Functional Limitation: 0** (0-4) - **Pain: 1** (0-4)

Quick DASH = 5

ICUC® ID: 23-DC-606 / 70y Female

Complete case data can be accessed by clicking the ***ICUC ID***

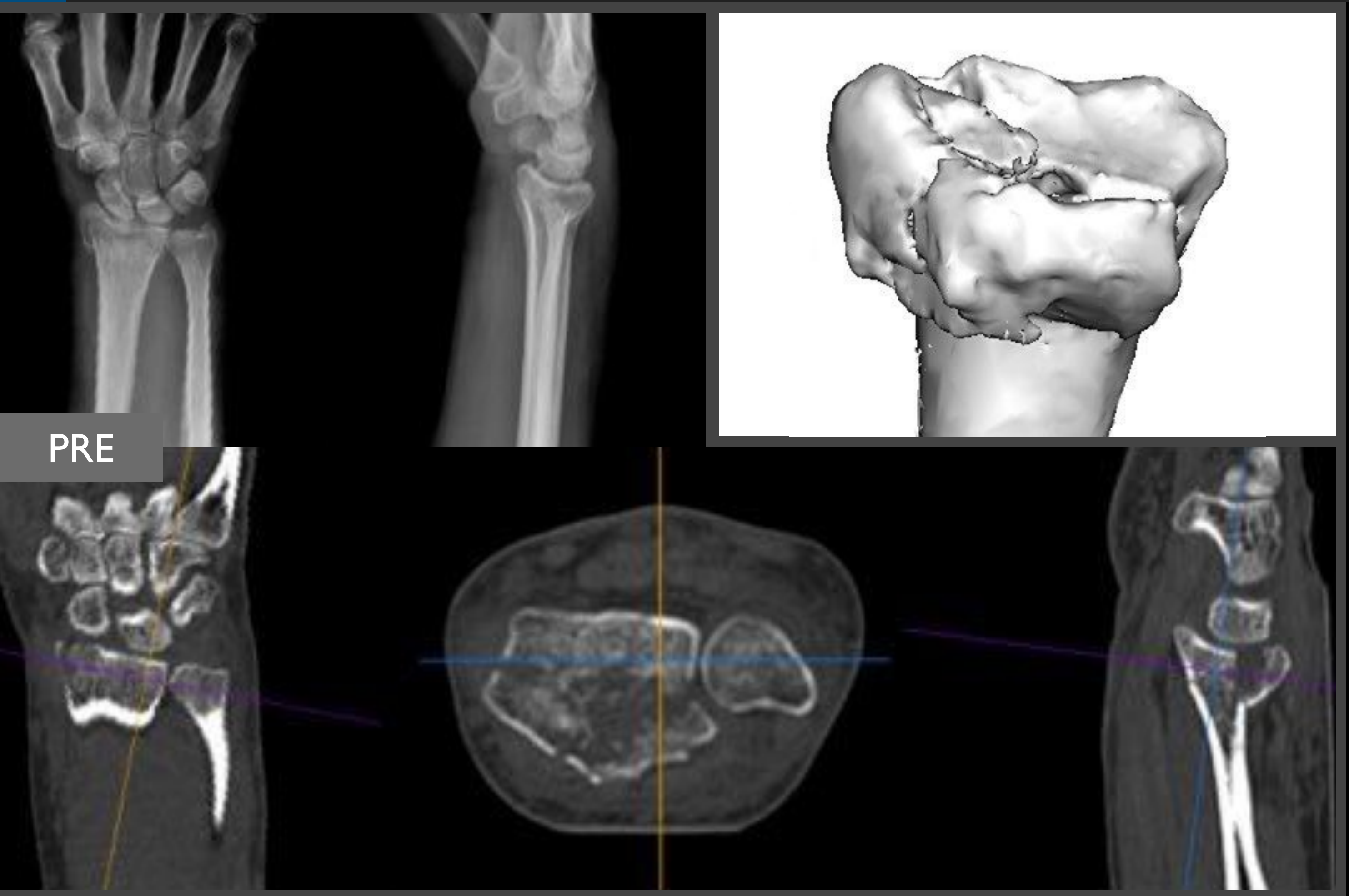

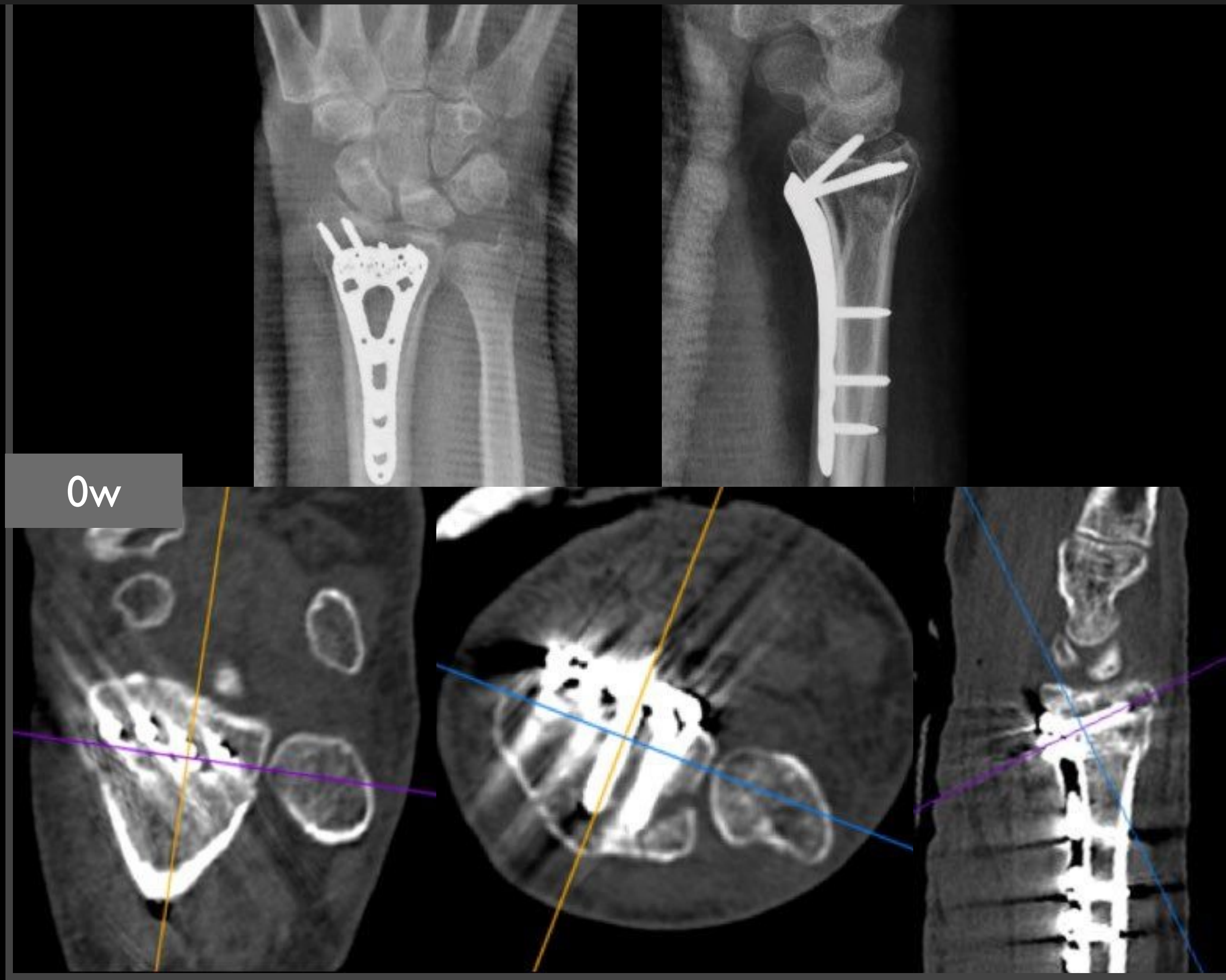

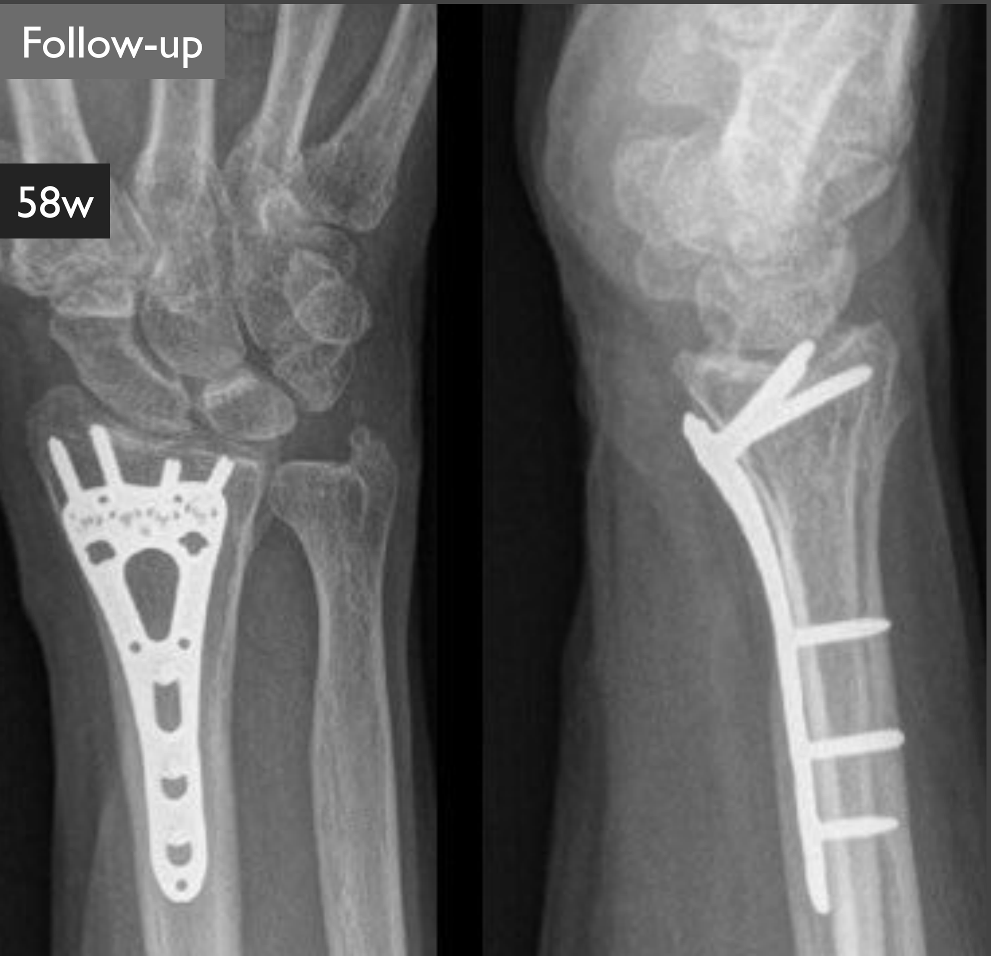

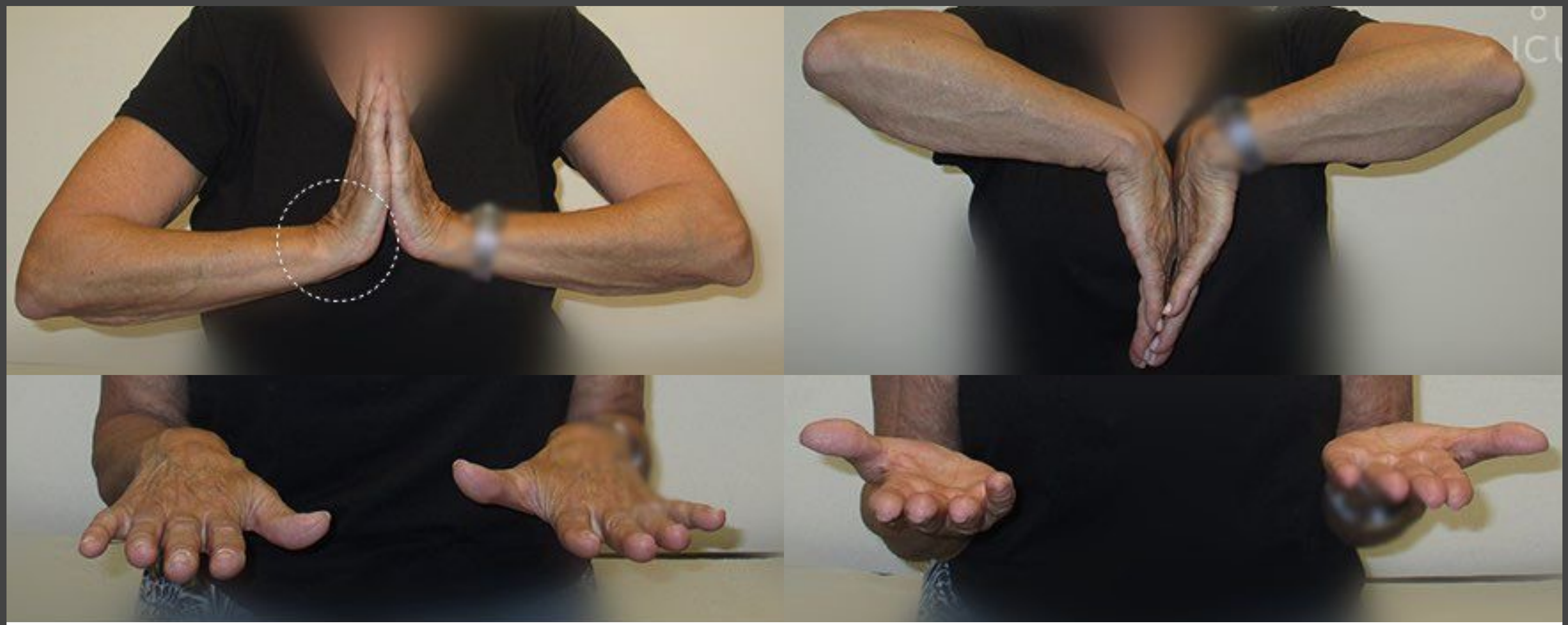

ICUC **Score** *Functional Limitation:* **0** (0-4) - *Pain:* **0** (0-4)

Quick DASH = 0

ICUC® ID: 23-DC-239 / 75y Female

Complete case data can be accessed by clicking the ***ICUC ID***

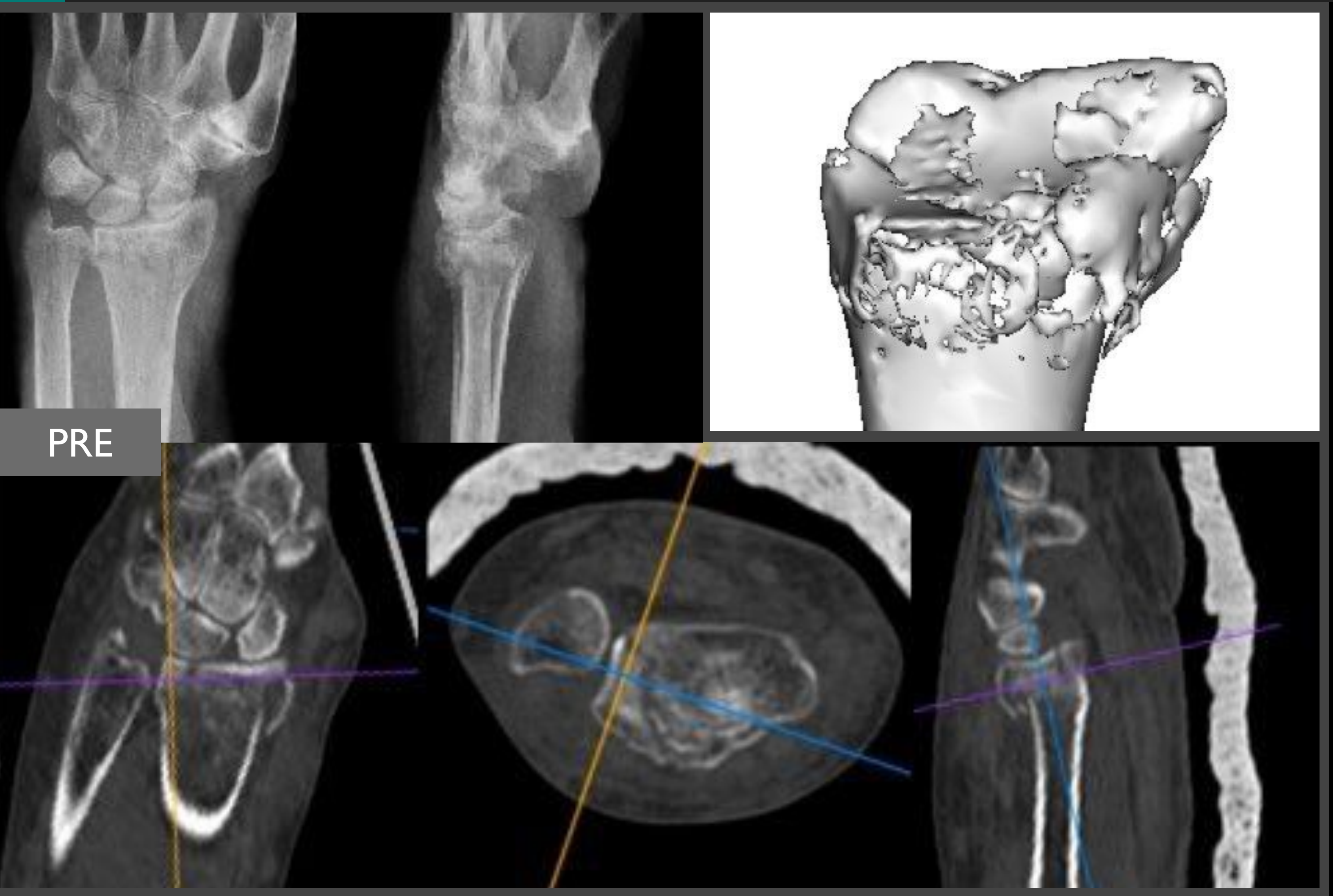

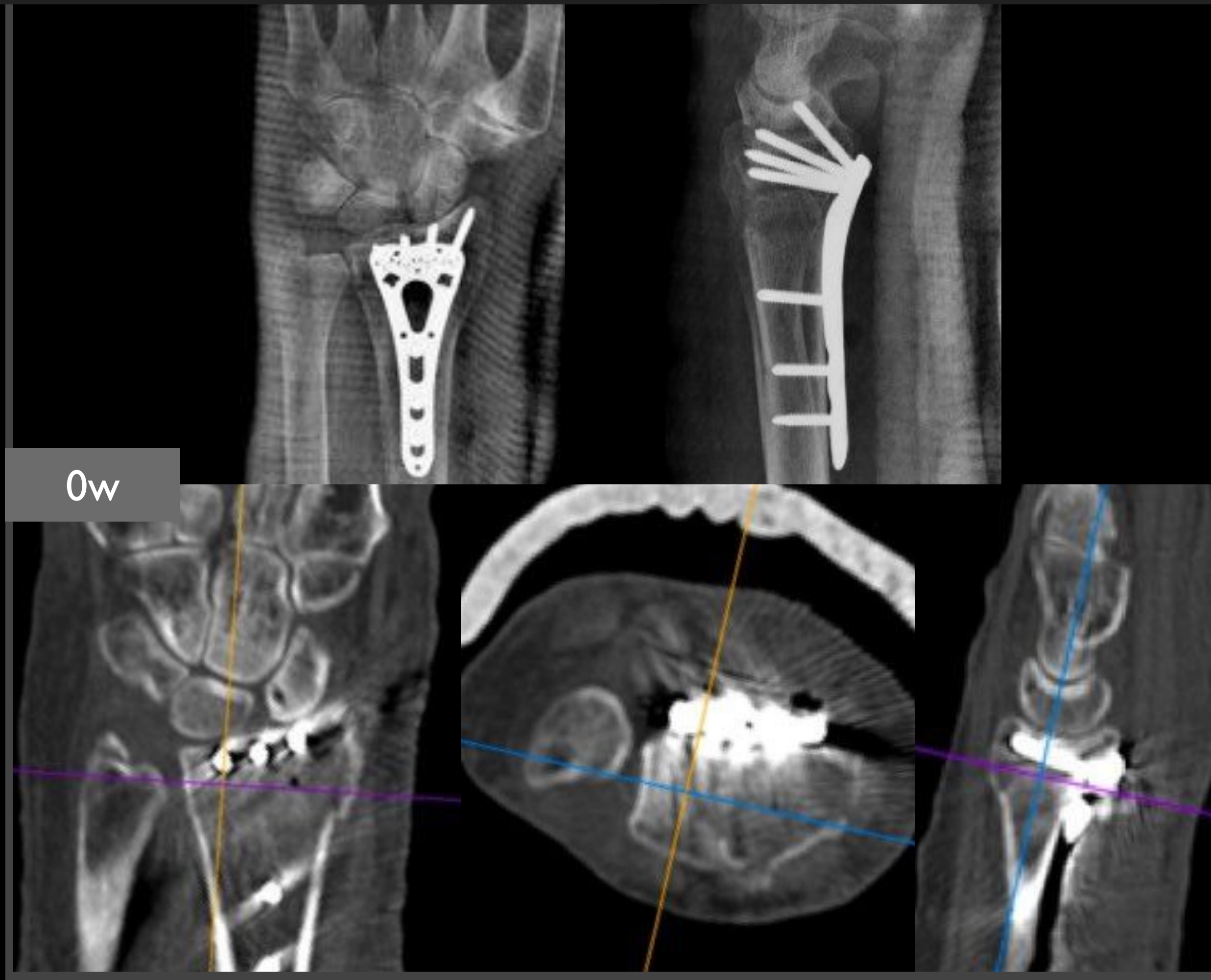

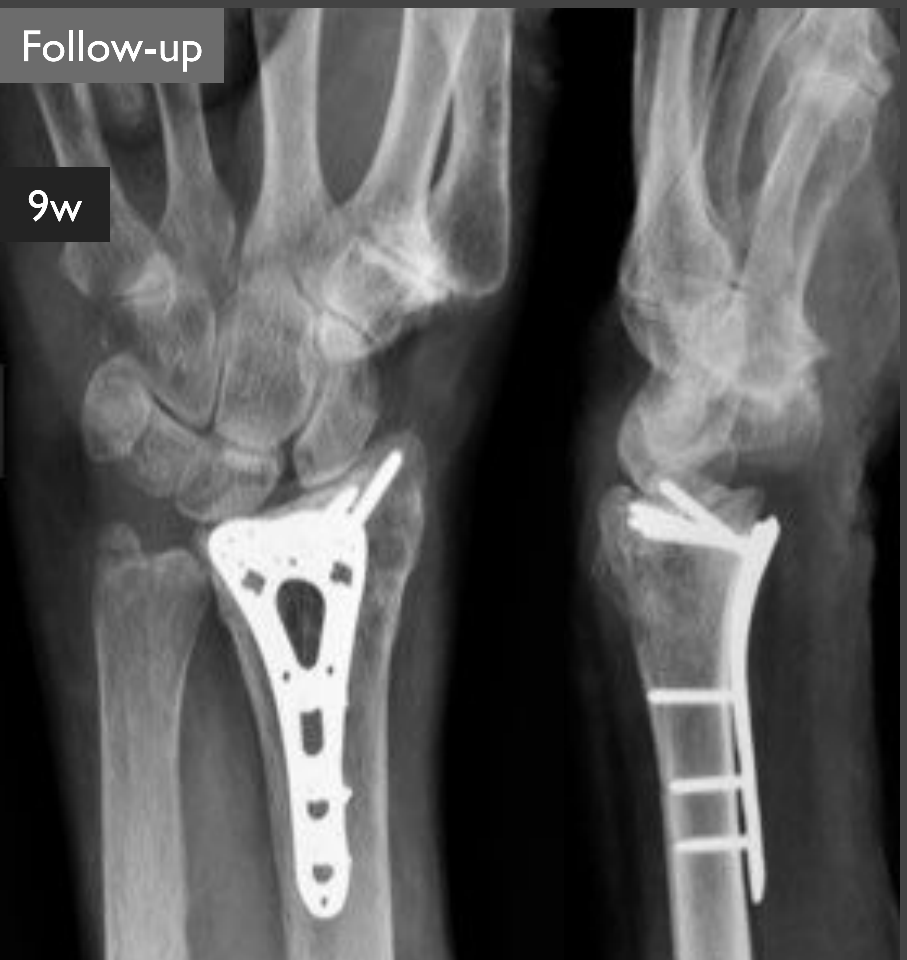

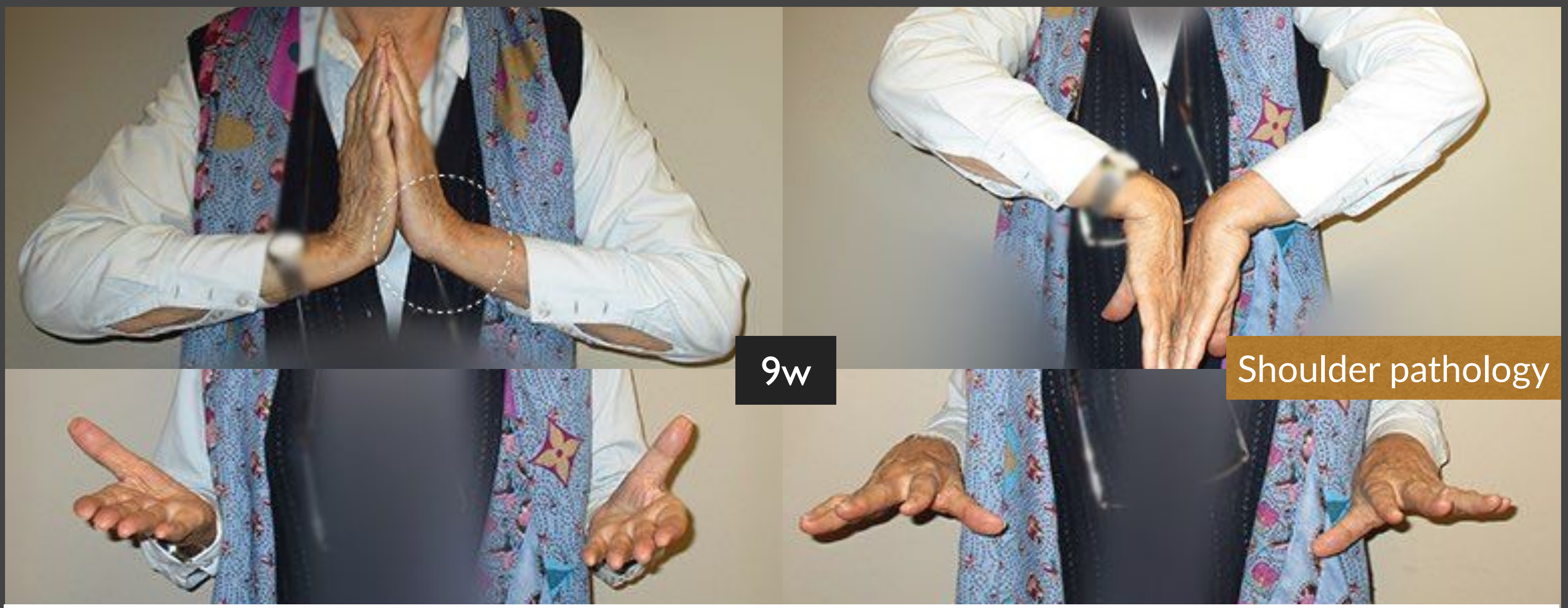

ICUC **Score** ***Functional Limitation:*** **2** (0-4) - ***Pain:*** **1** (0-4)

ICUC Score at 345w Functional limitation: 0 Pain: 0

Quick DASH = 16

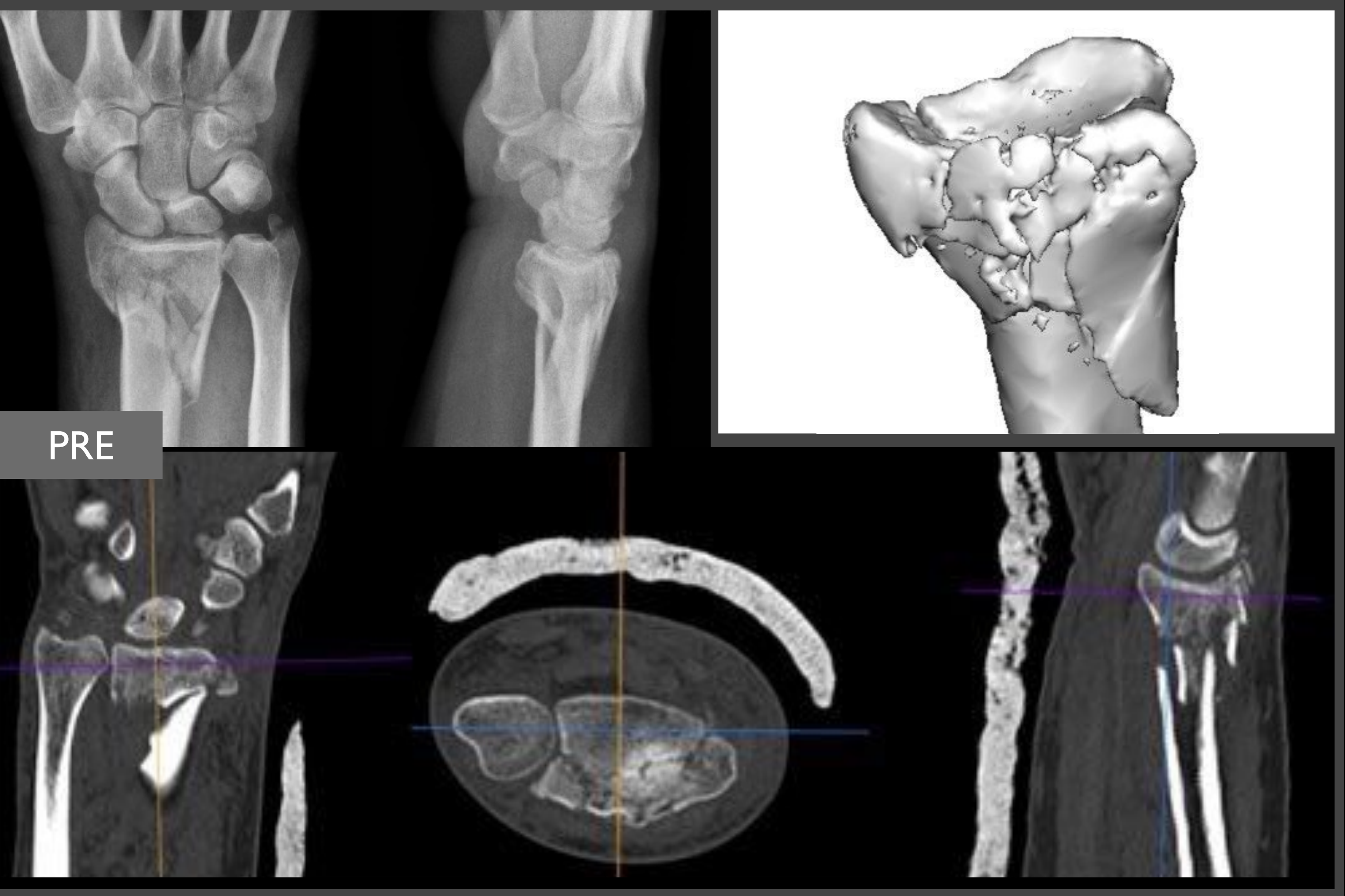

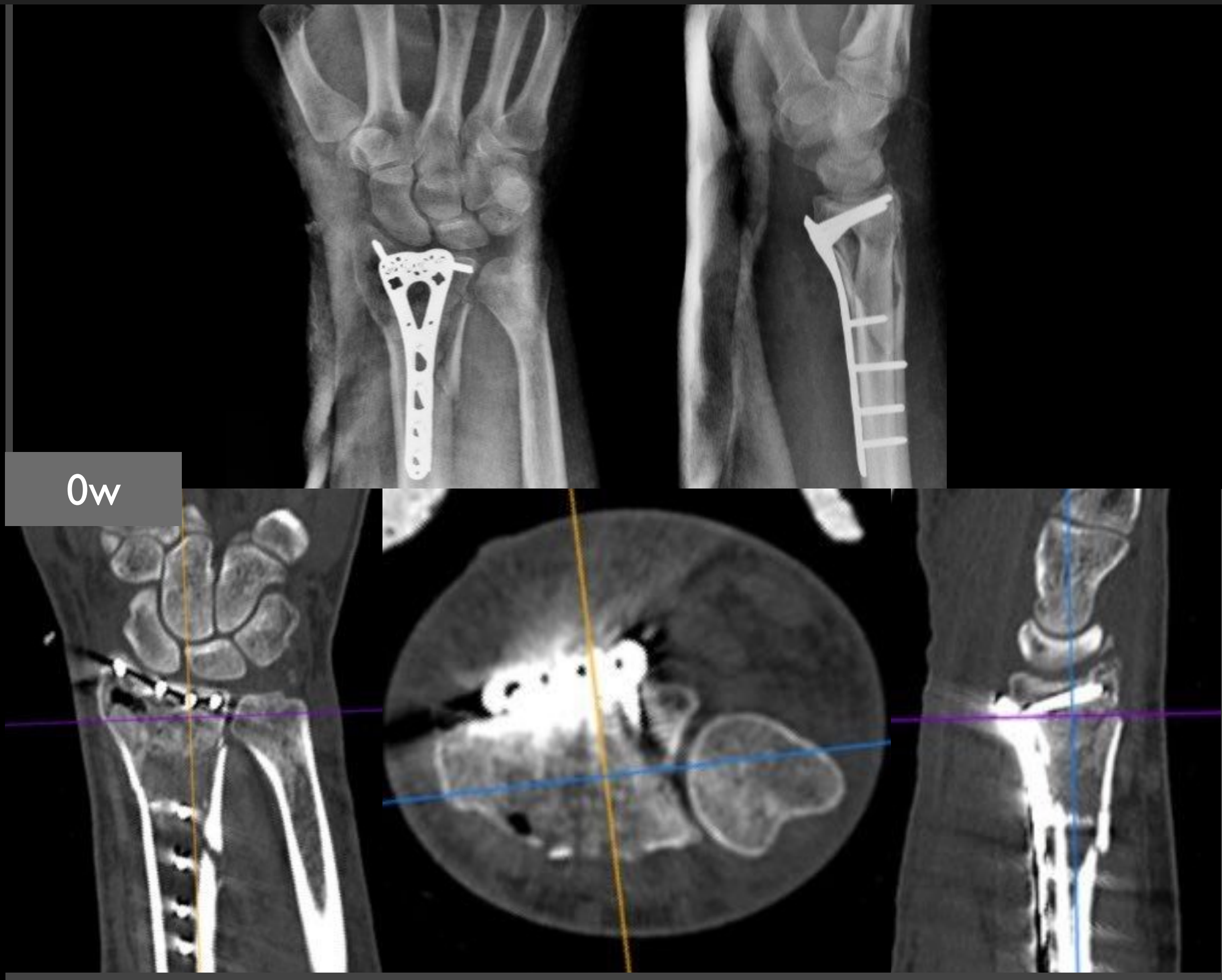

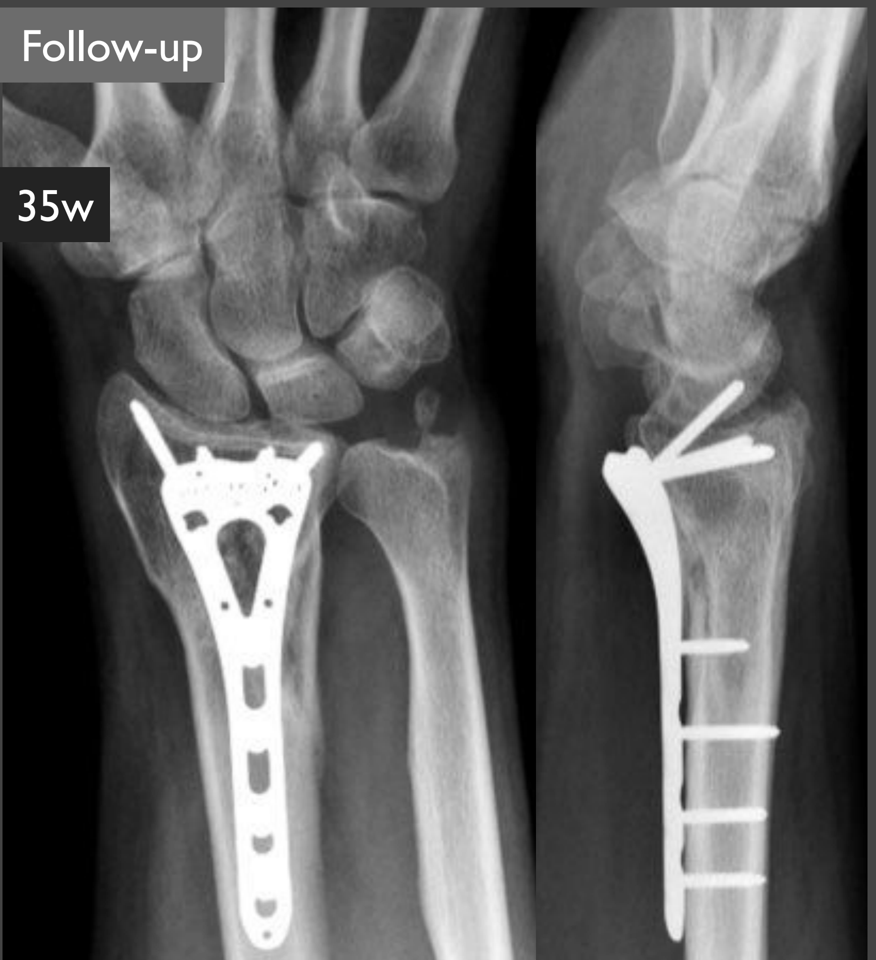

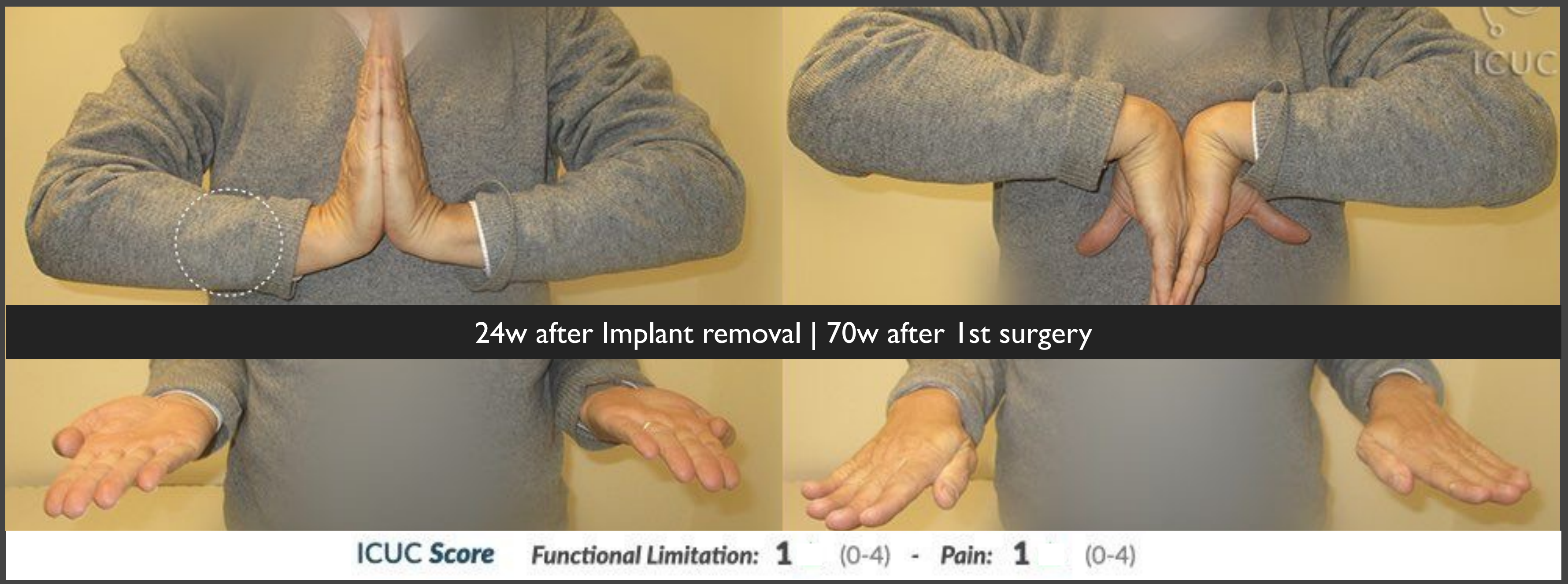

ICUC Score at 333w Functional limitation: 1 Pain: 0

Quick DASH = 2

ICUC® ID: 23-DC-770 / 70y Female

Complete case data can be accessed by clicking the ***ICUC ID***

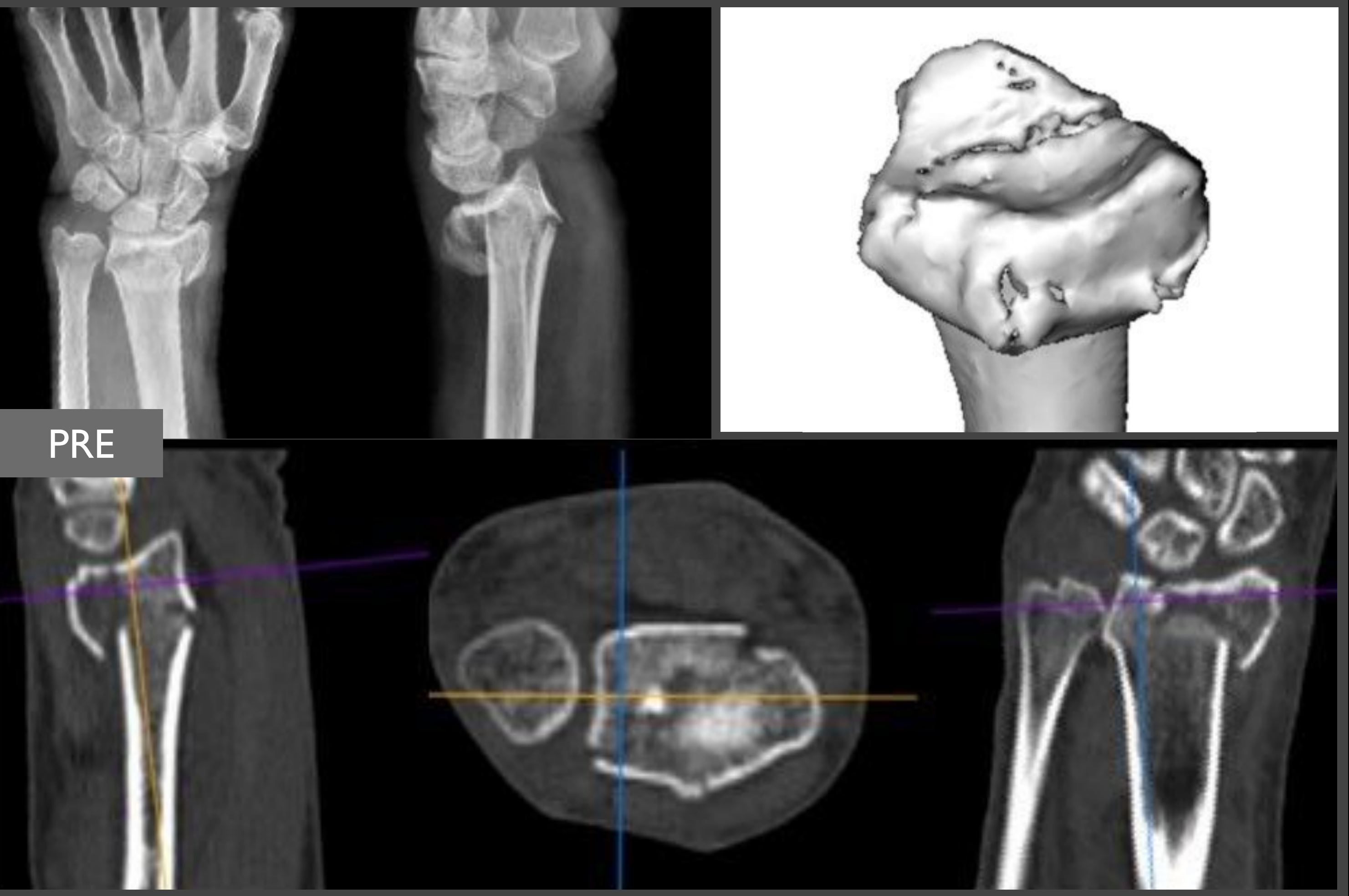

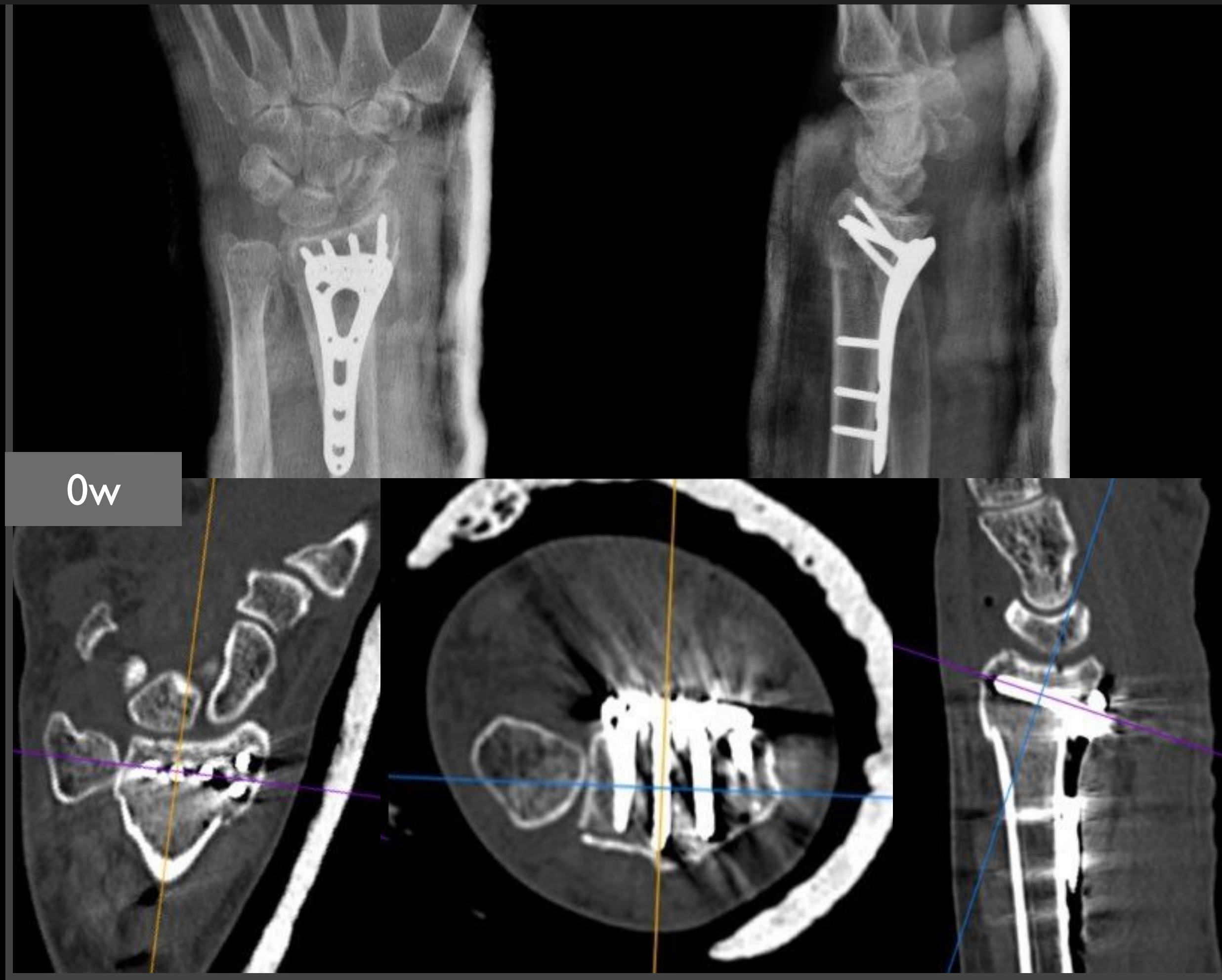

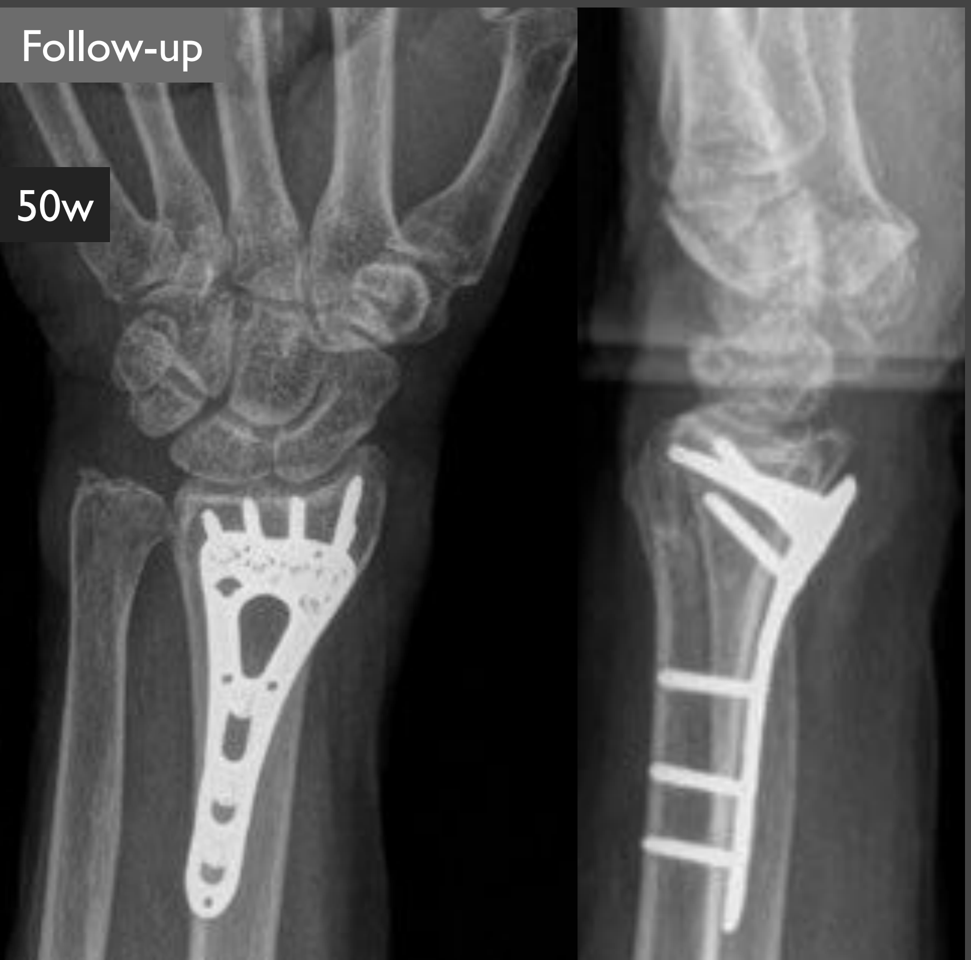

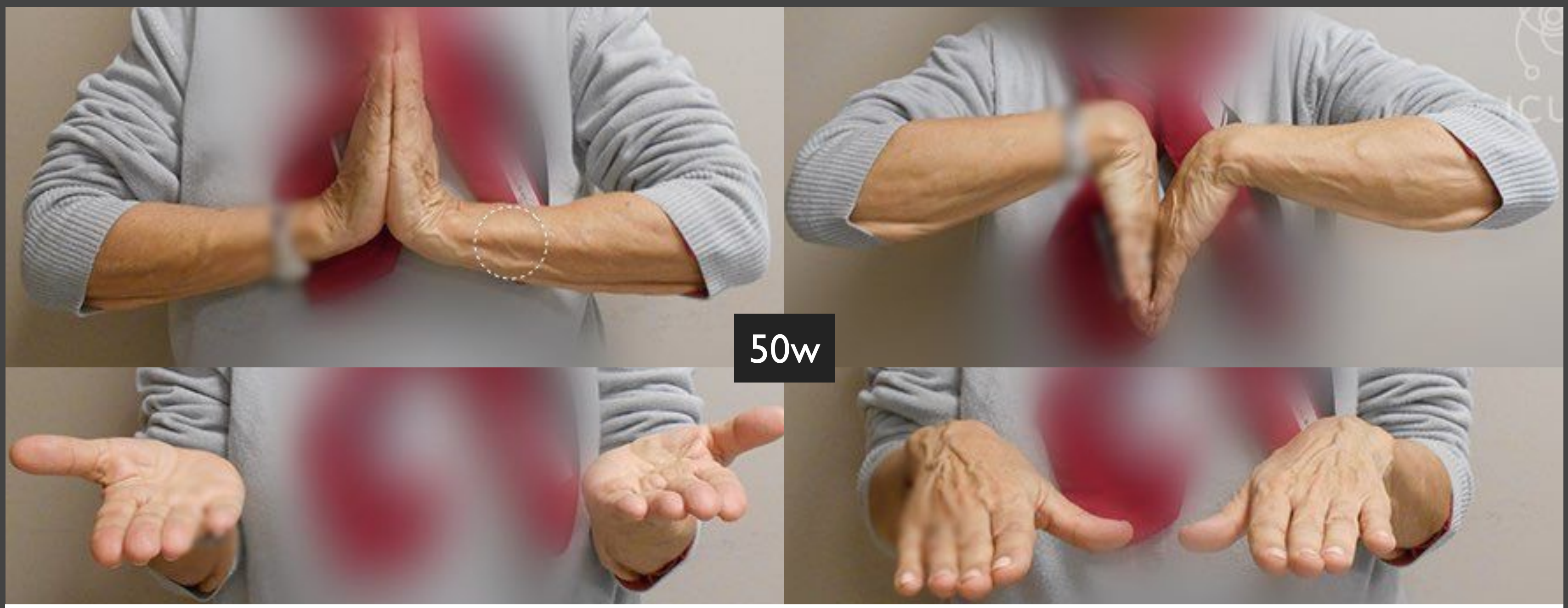

ICUC **Score** ***Functional Limitation:*** **0** (0-4) - ***Pain:*** **0** (0-4)

Quick DASH = 0

ICUC® ID: 23-DC-003 / 65y Female

*Complete case data can be accessed by clicking the **ICUC ID***

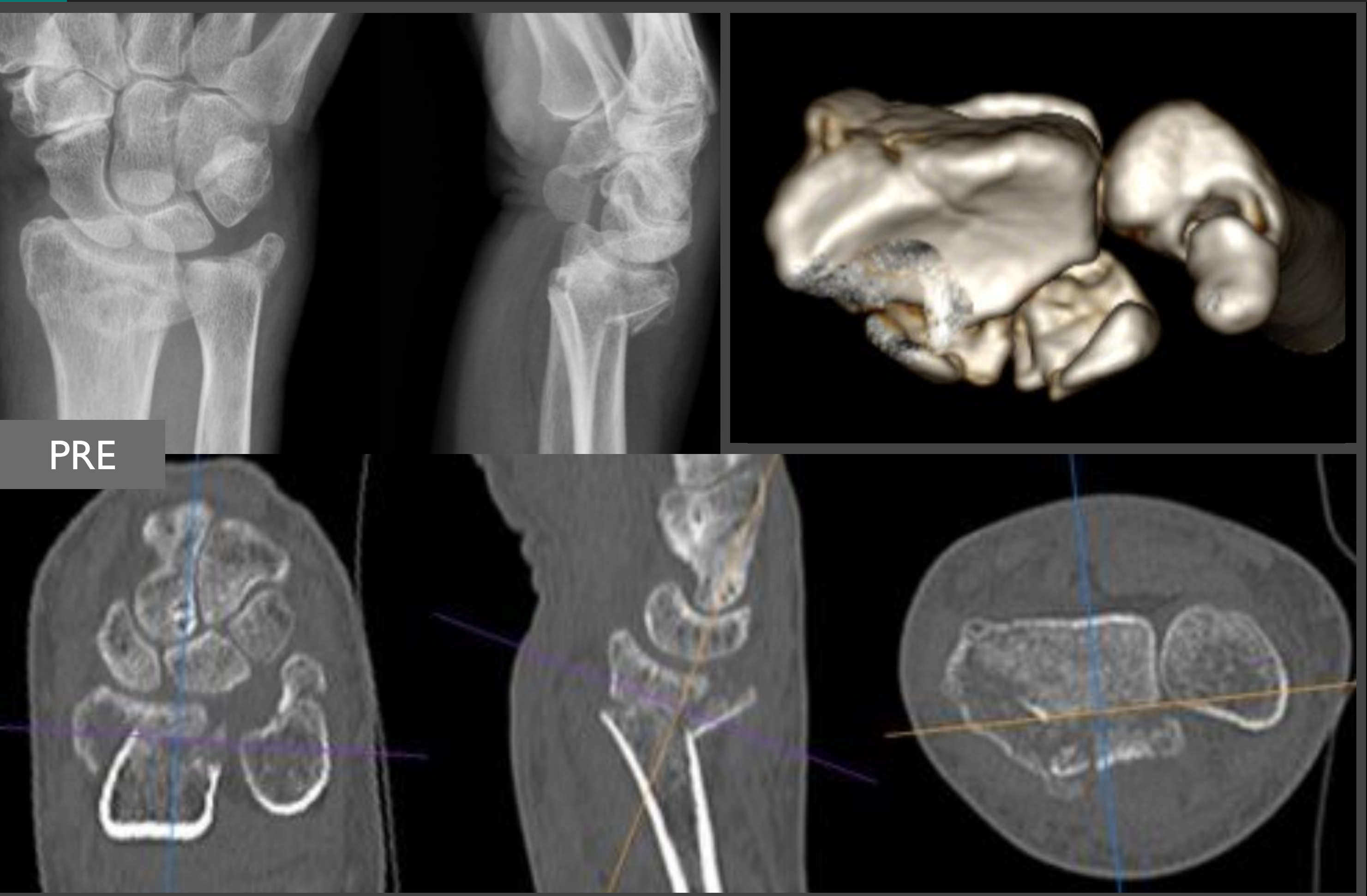

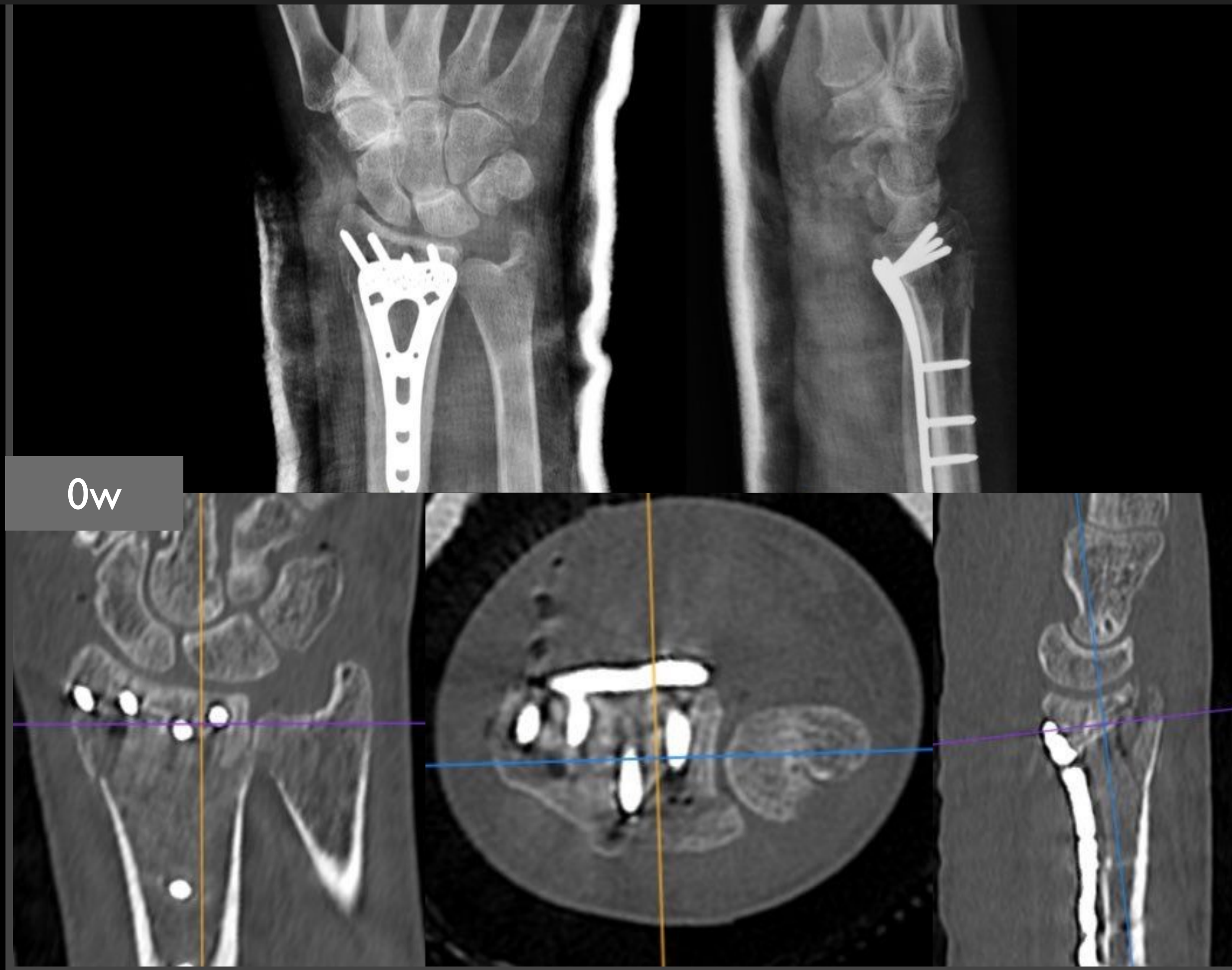

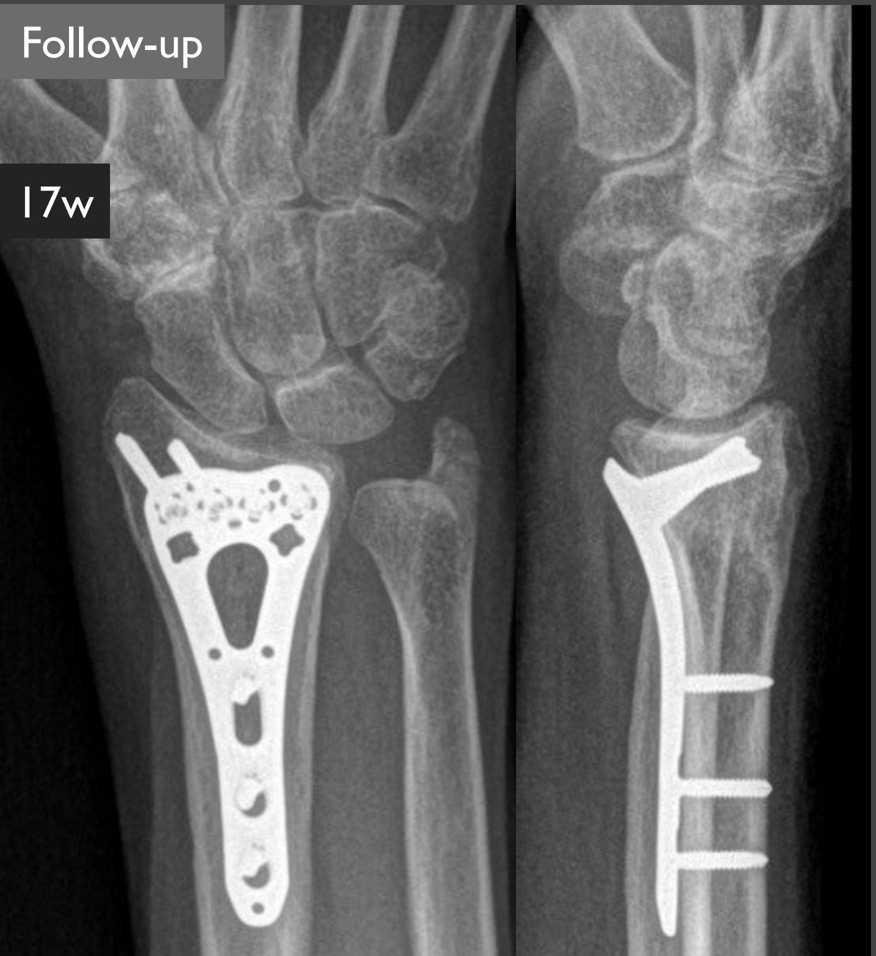

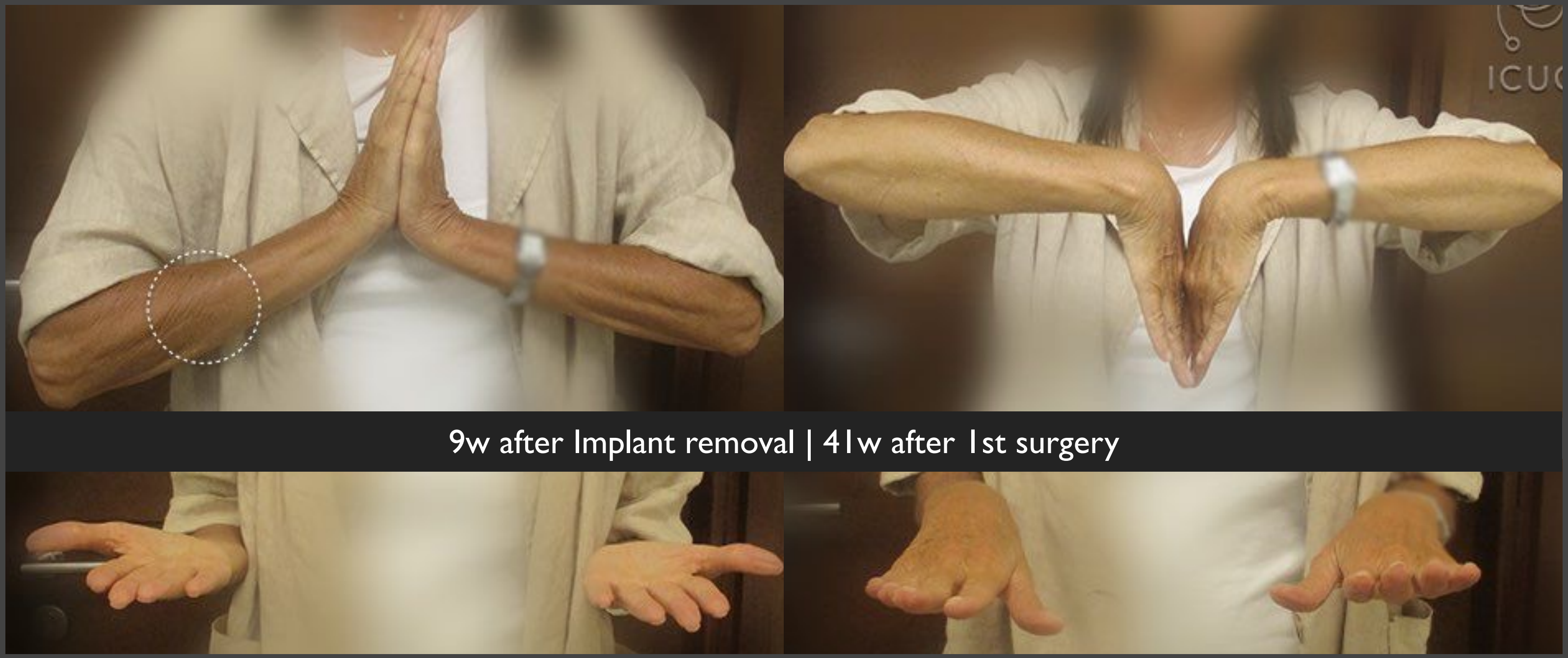

9w after Implant removal | 41w after 1st surgery

ICUC Score at 301w Functional limitation: 0 Pain: 0

Quick DASH = 2.2

ICUC® ID: 23-VC-192 / 60y Female

Complete case data can be accessed by clicking the ***ICUC ID***

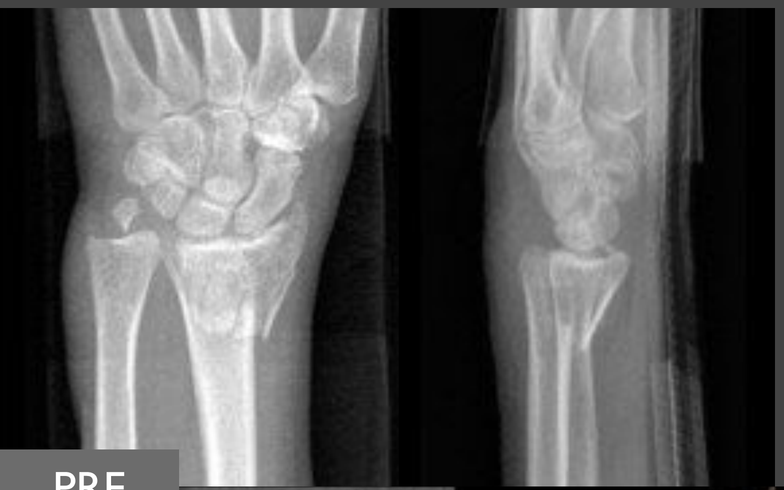

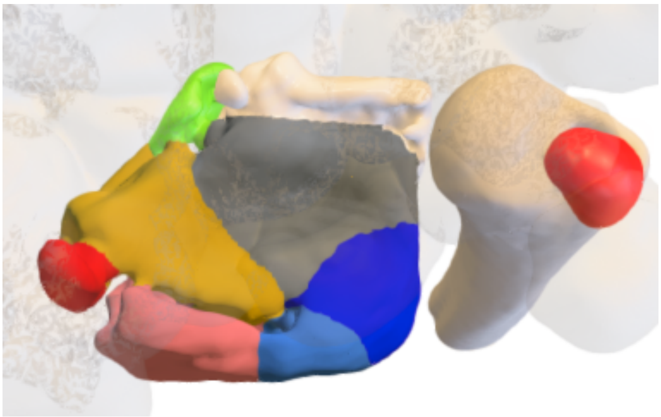

3D Colored Model

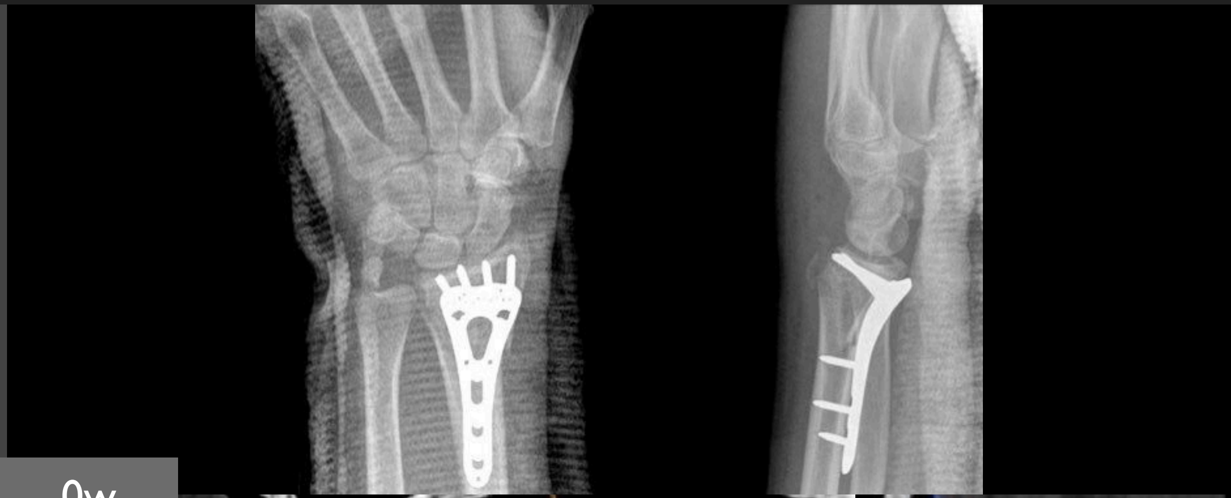

PRE

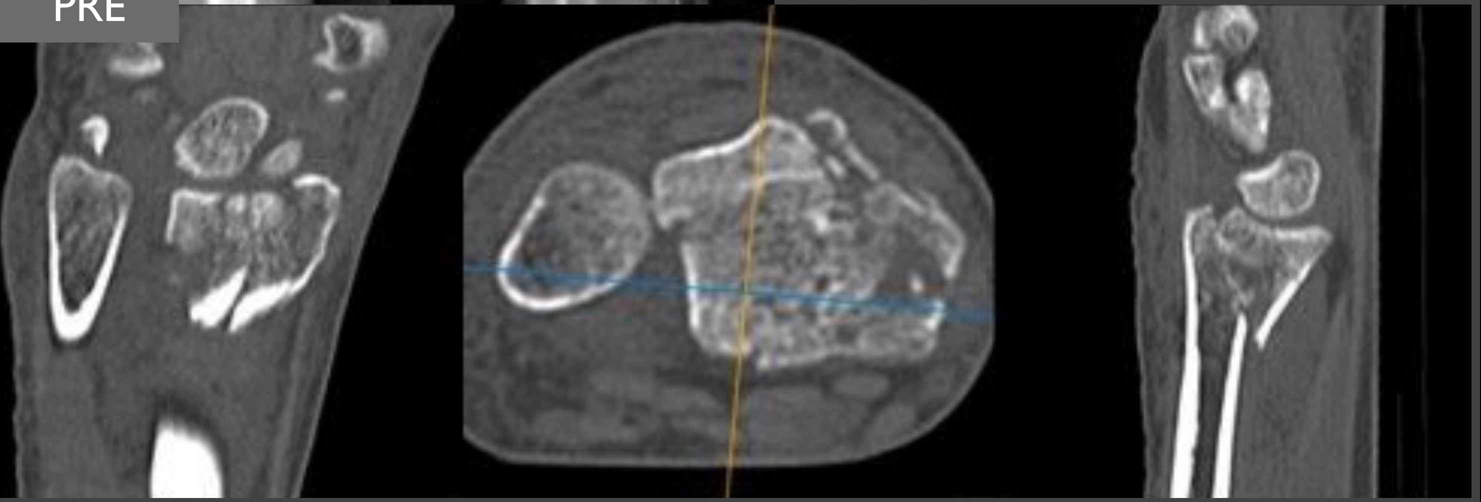

0w

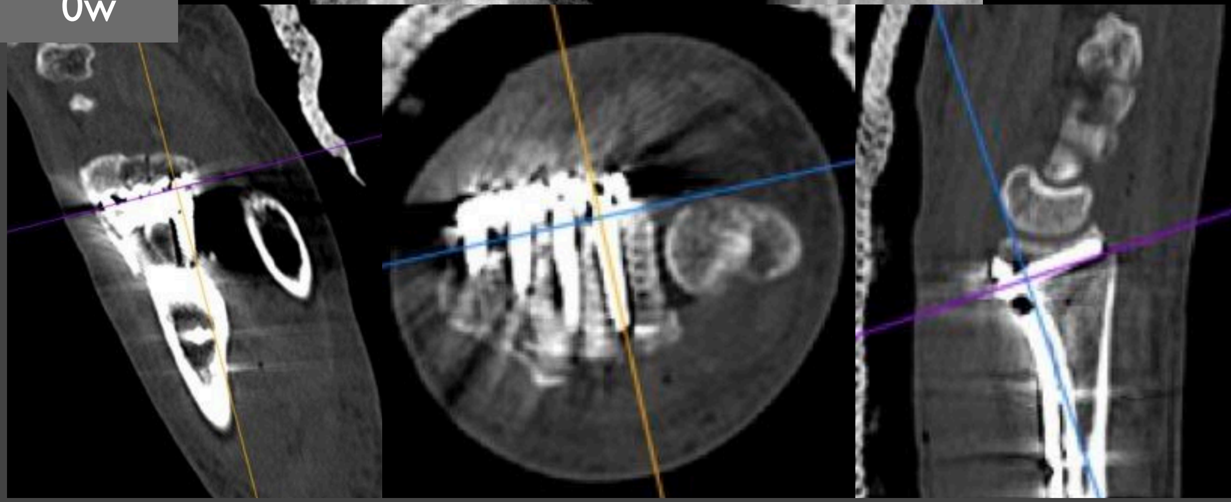

Follow-up

52w

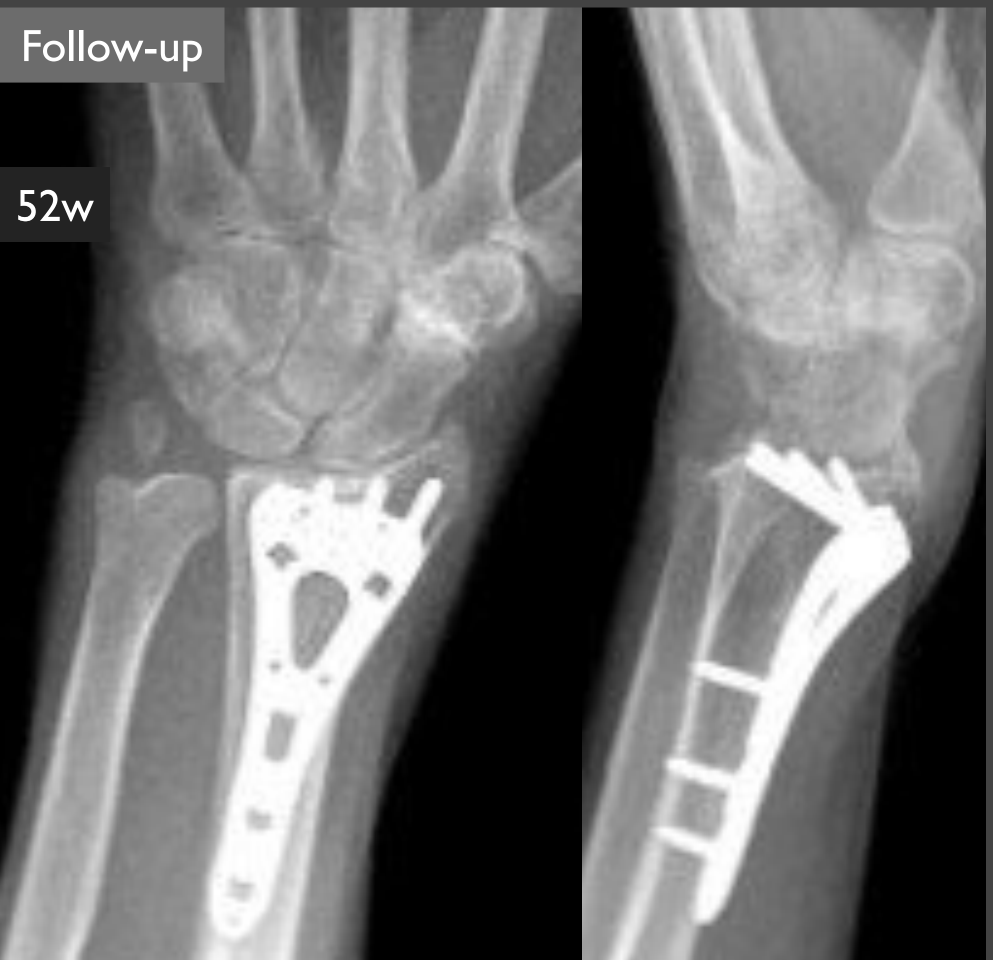

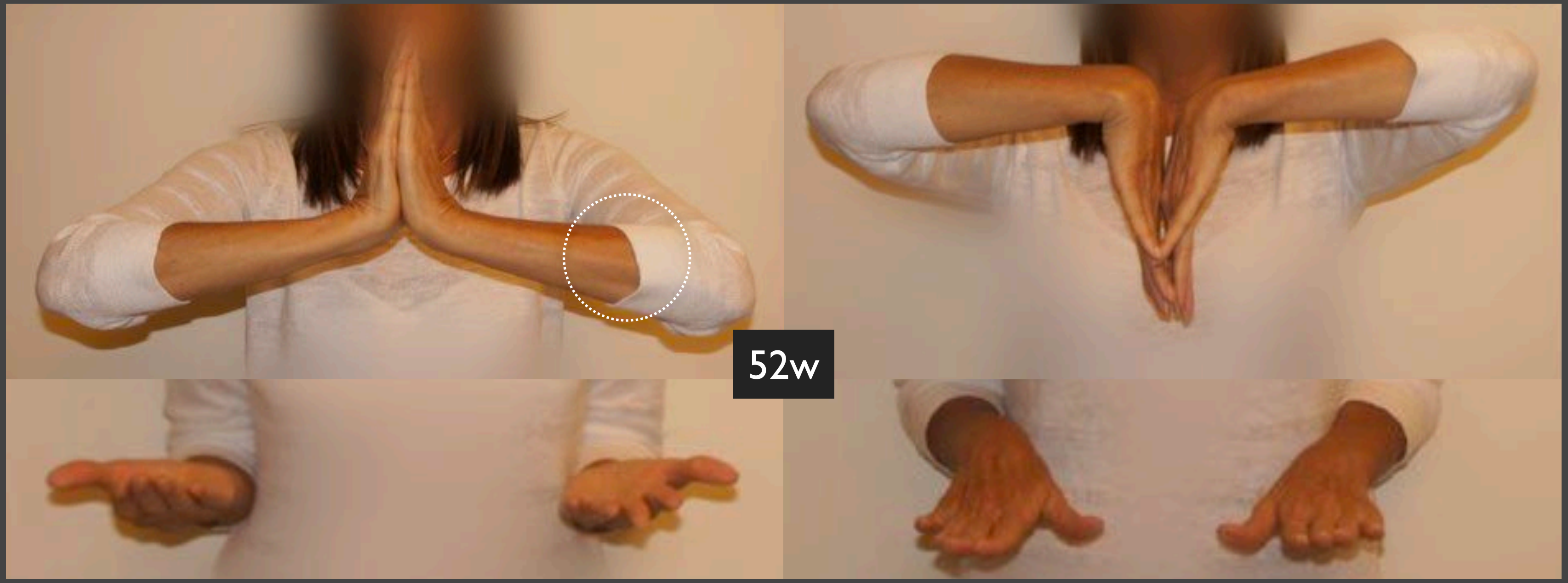

52w

ICUC Score at 166w Functional limitation: 0 Pain: 0

Quick DASH = 4.5

ICUC® ID: 23-VC-054 / 65y Female

Complete case data can be accessed by clicking the ***ICUC ID***

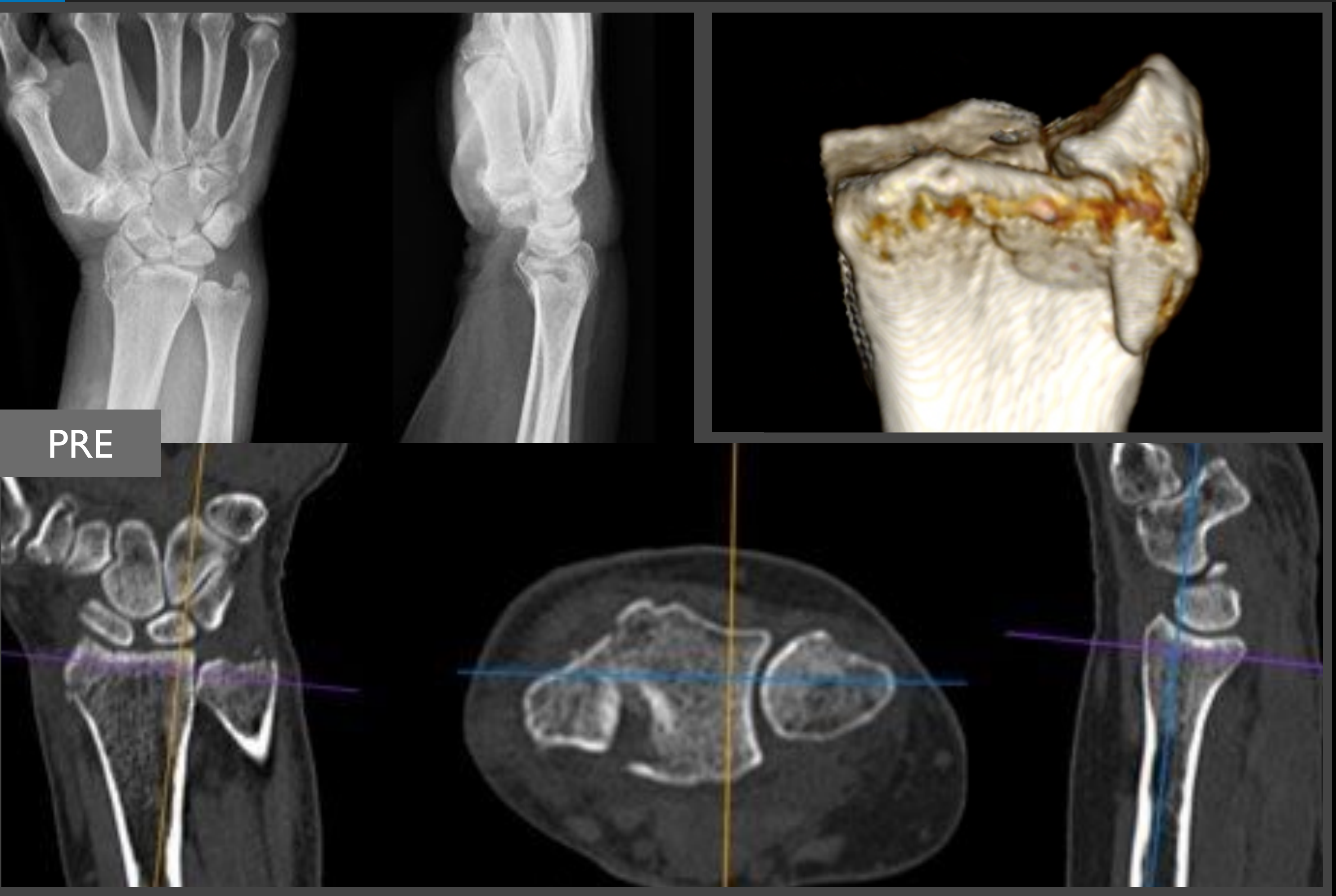

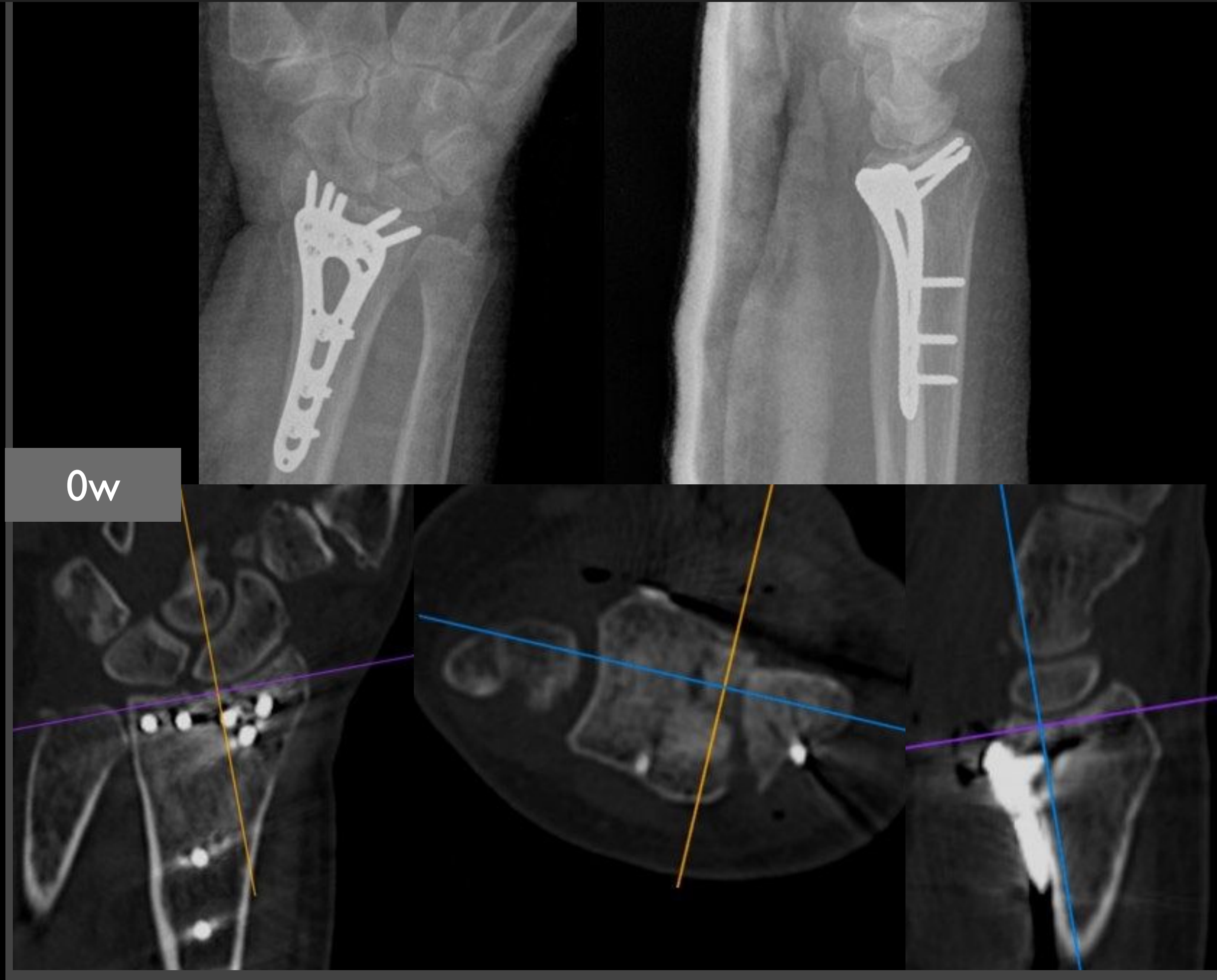

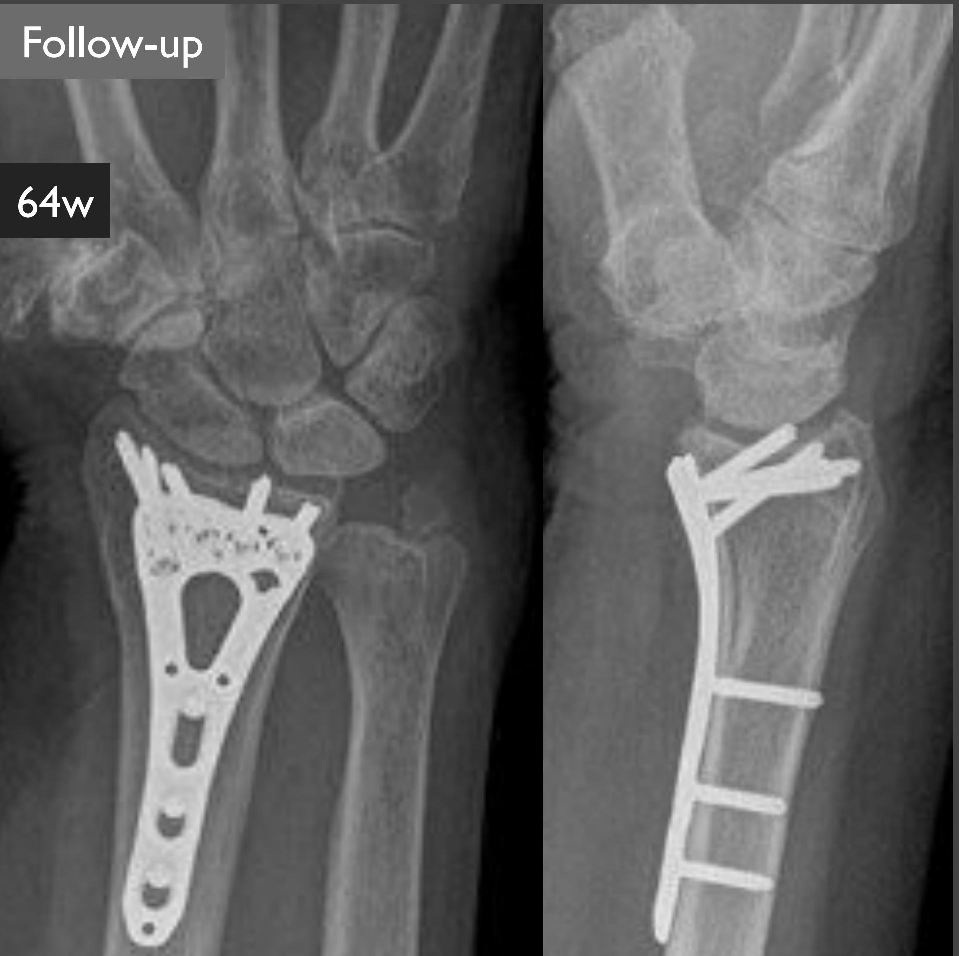

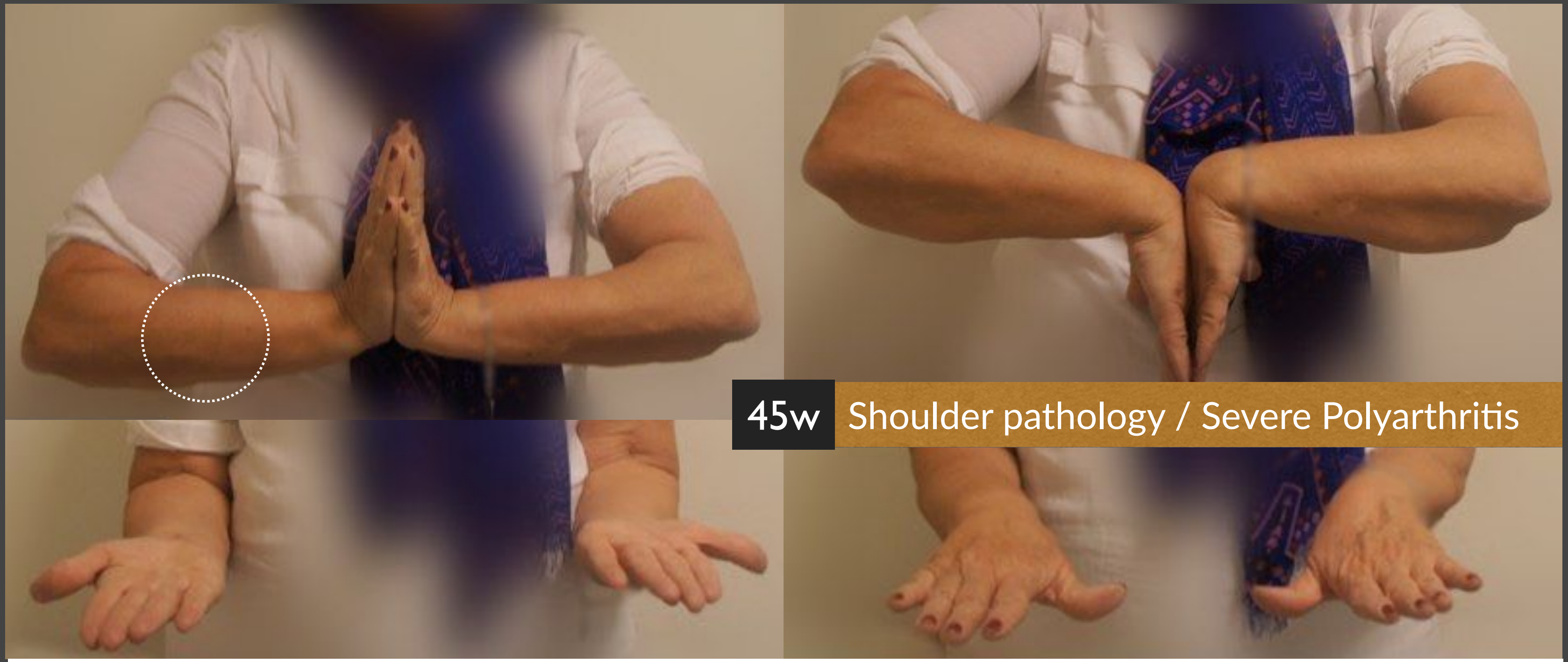

ICUC Score ***Functional Limitation:*** **0** (0-4) - ***Pain:*** **0** (0-4)

Quick DASH = 32

ICUC® ID: 23-DC-241 / 70y Female

*Complete case data can be accessed by clicking the **ICUC ID***

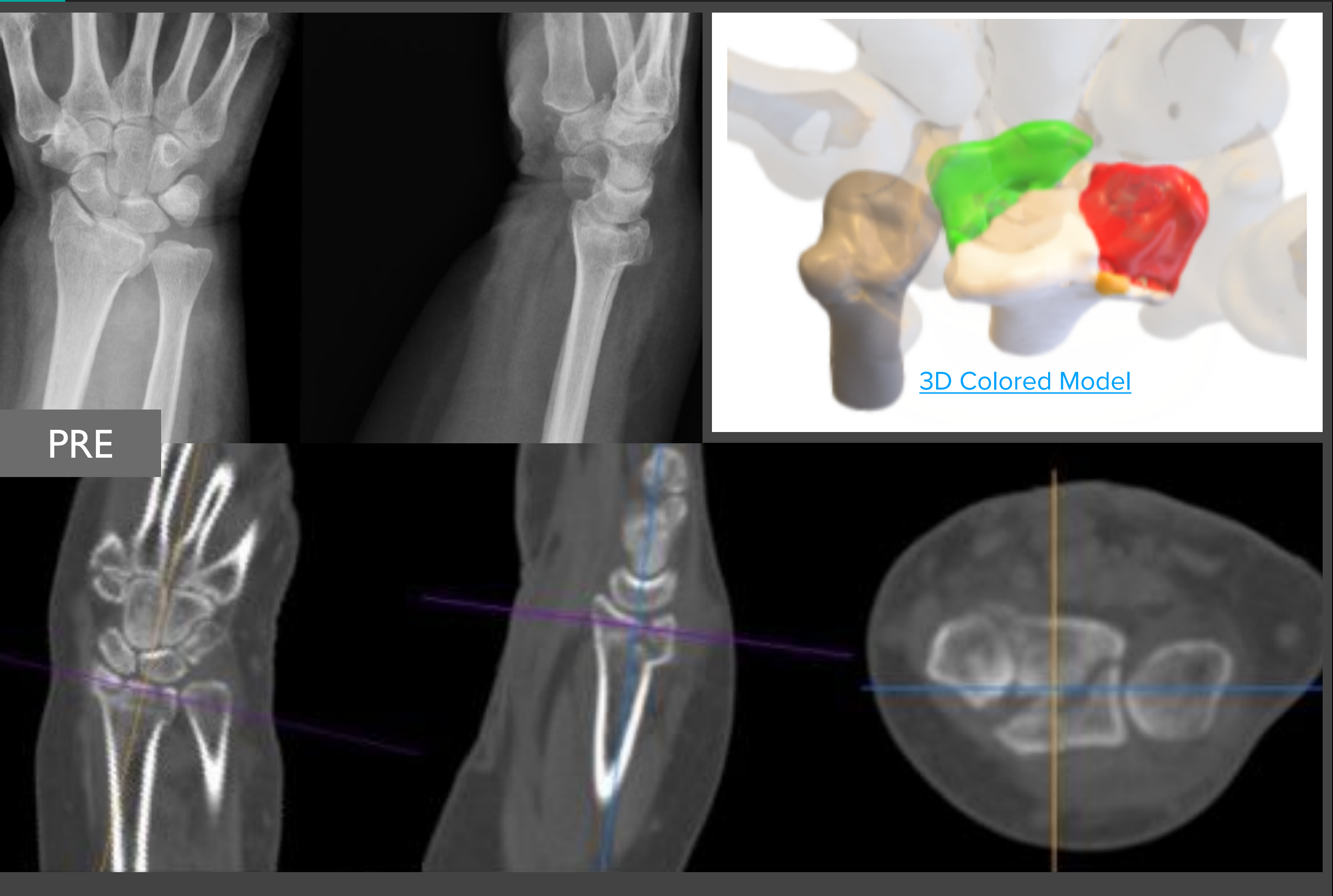

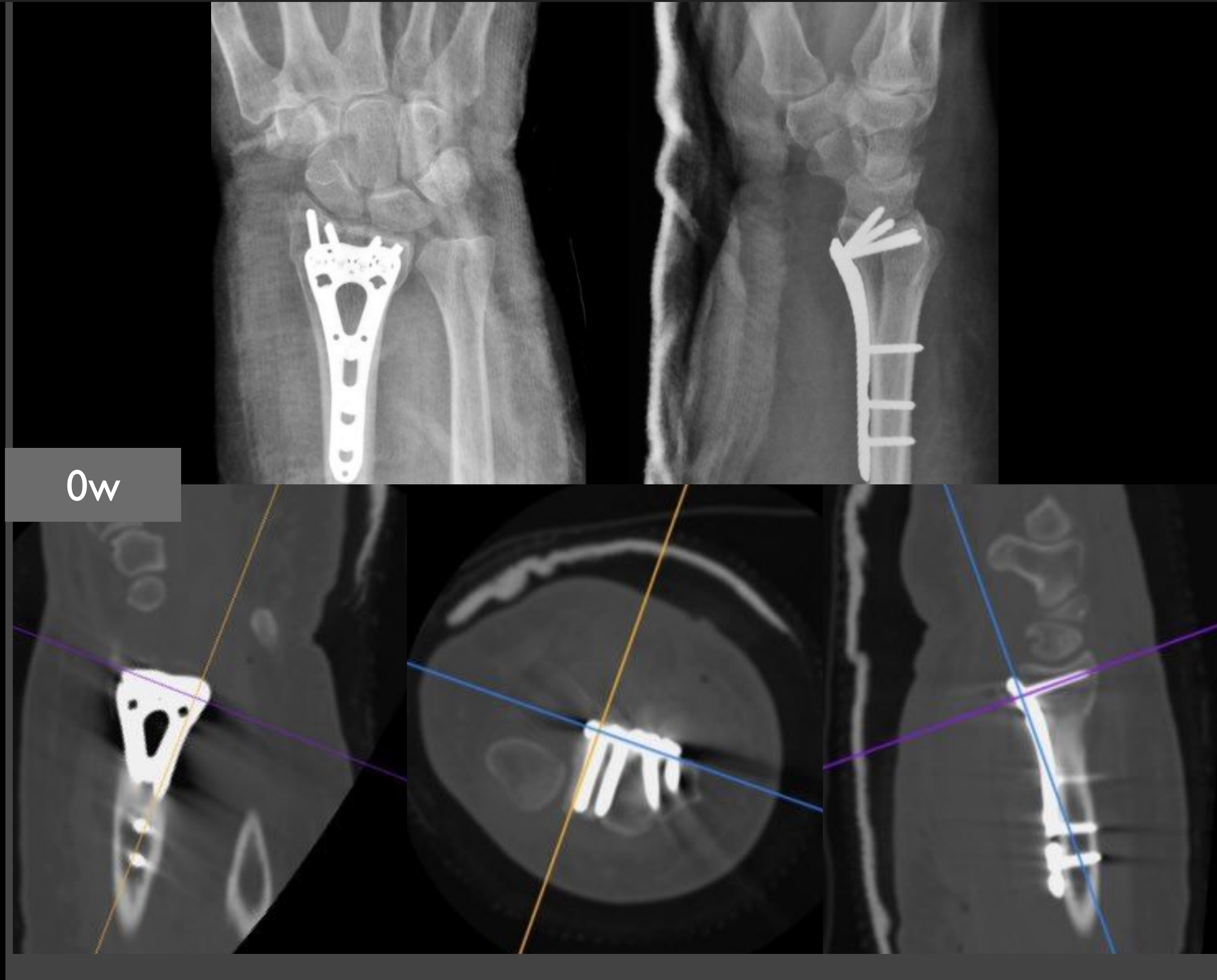

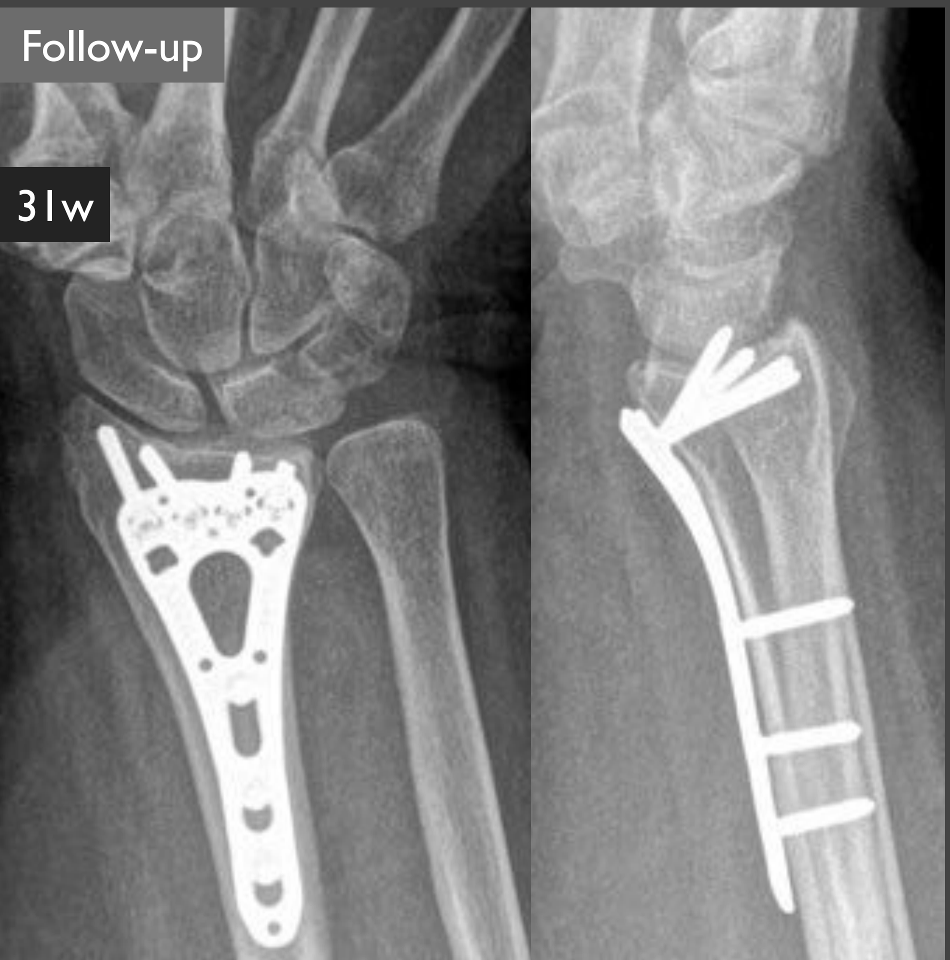

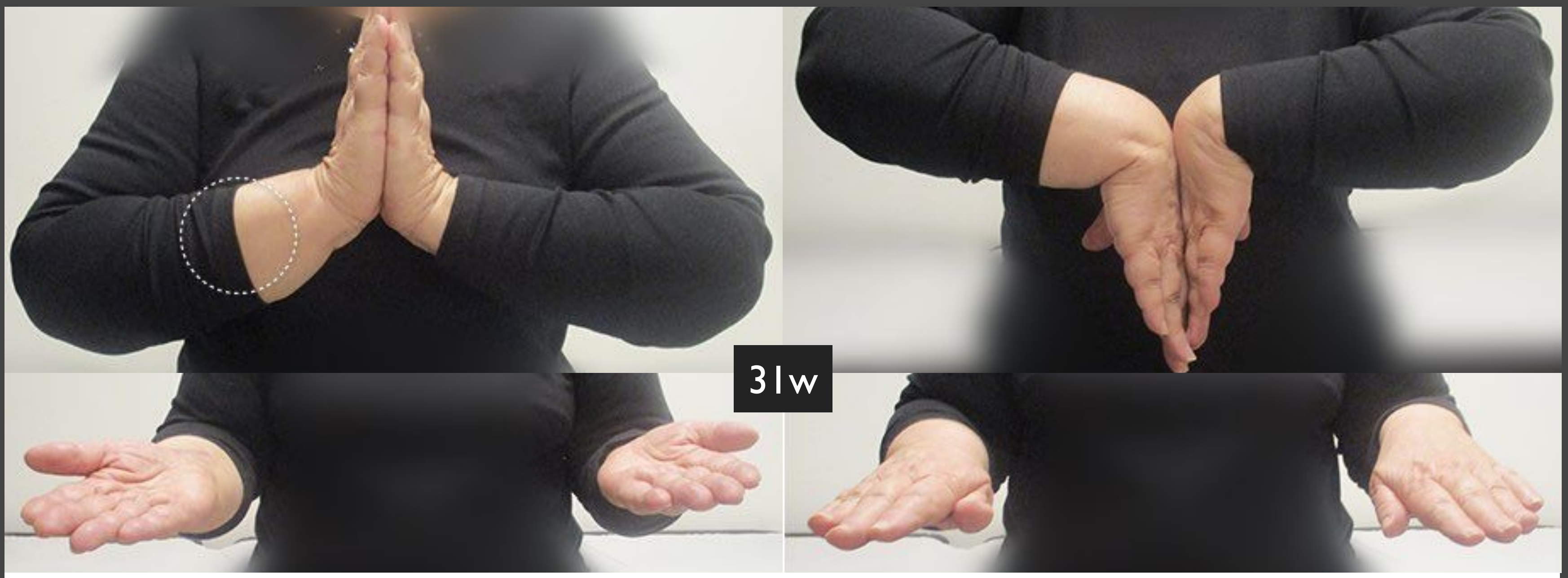

ICUC **Score** **Functional Limitation: 0** (0-4) - **Pain: 0** (0-4)

Quick DASH = 0

ICUC® ID: 23-DC-343 / 30y Male

Complete case data can be accessed by clicking the ***ICUC ID***

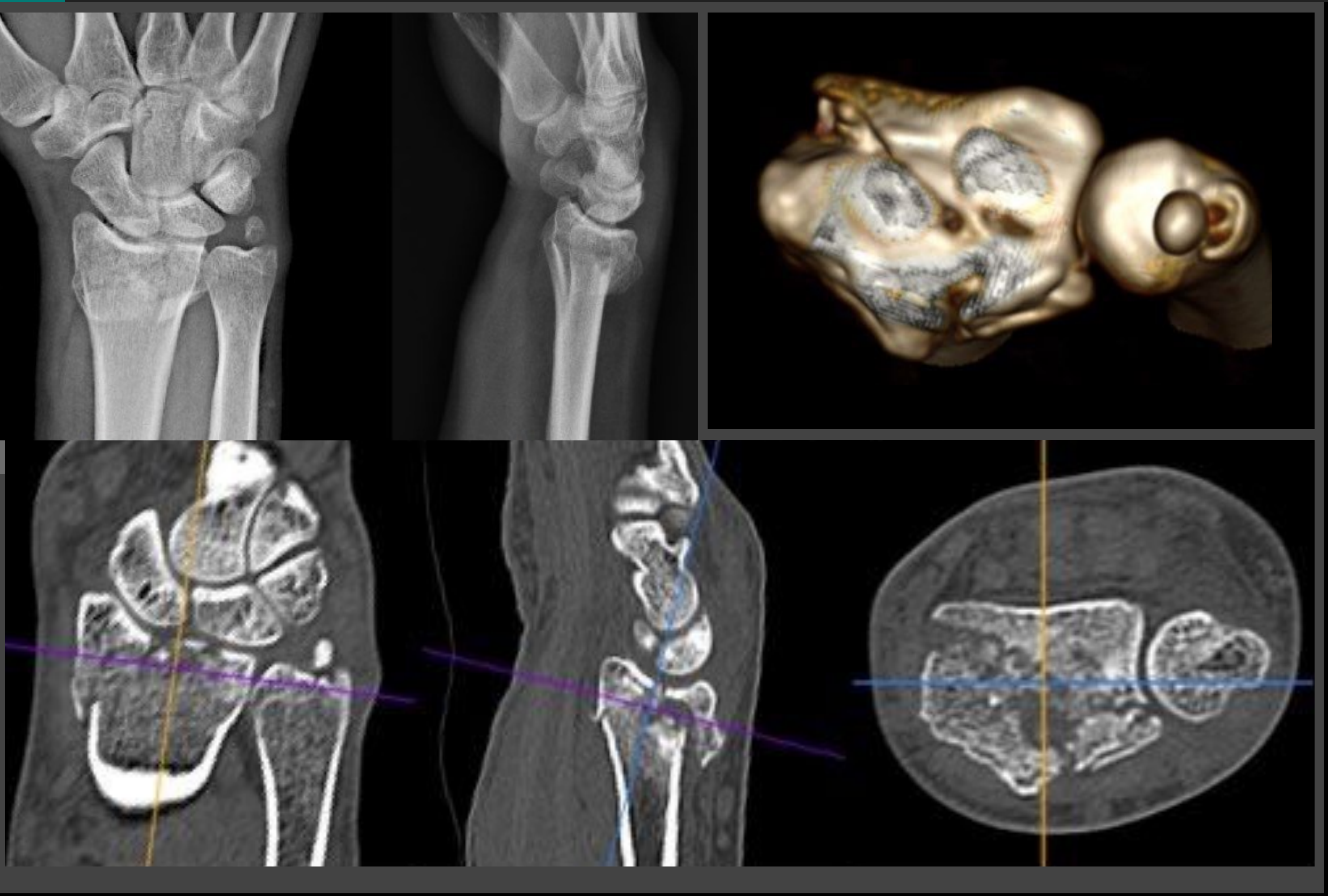

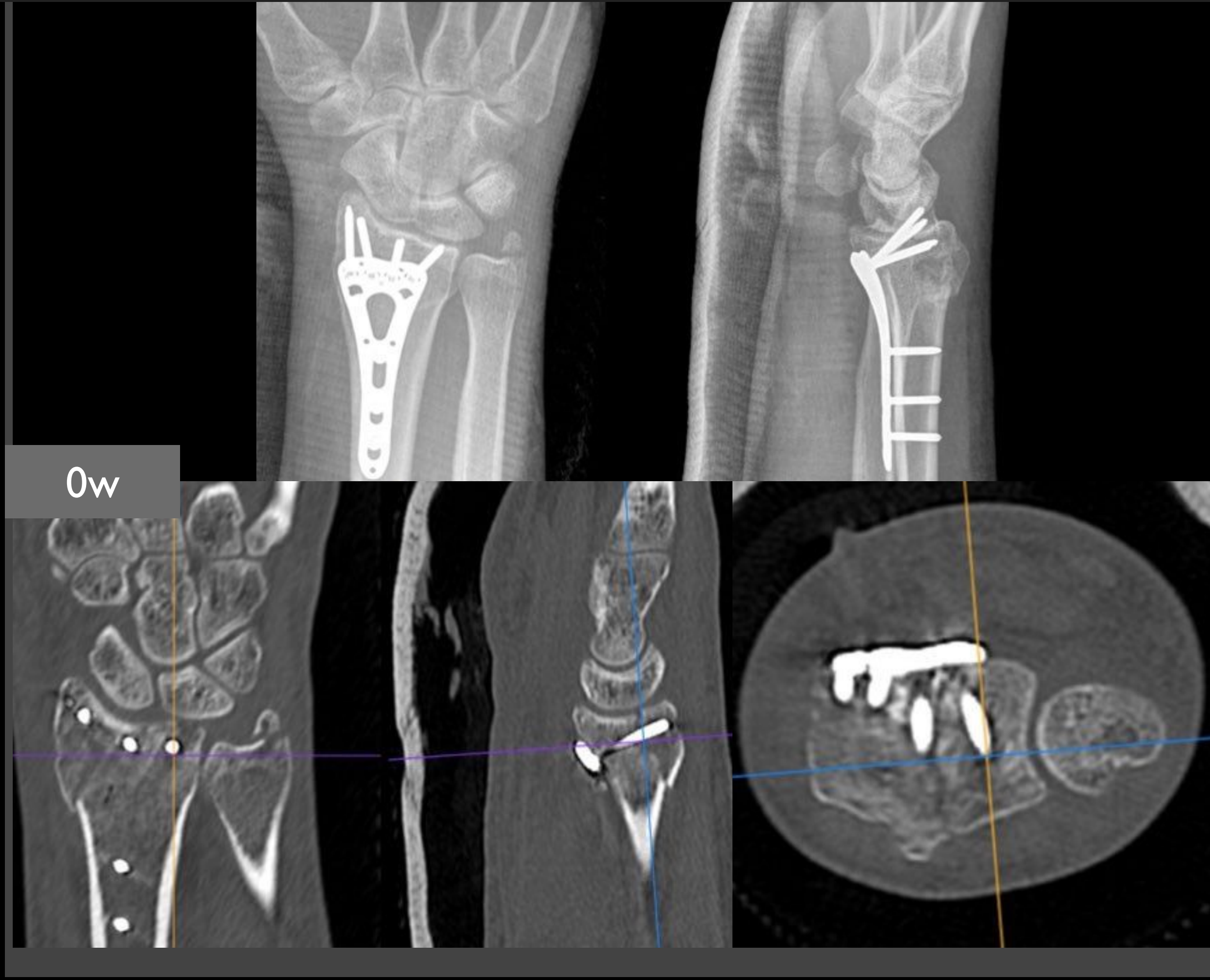

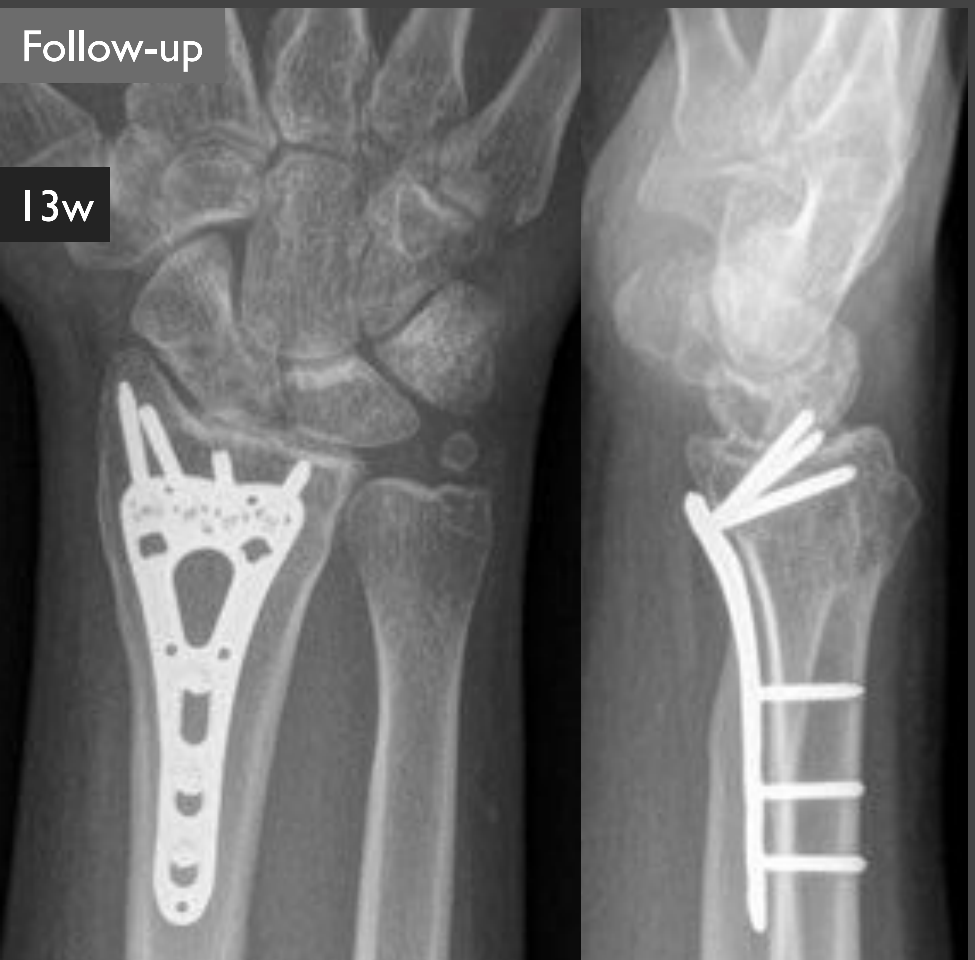

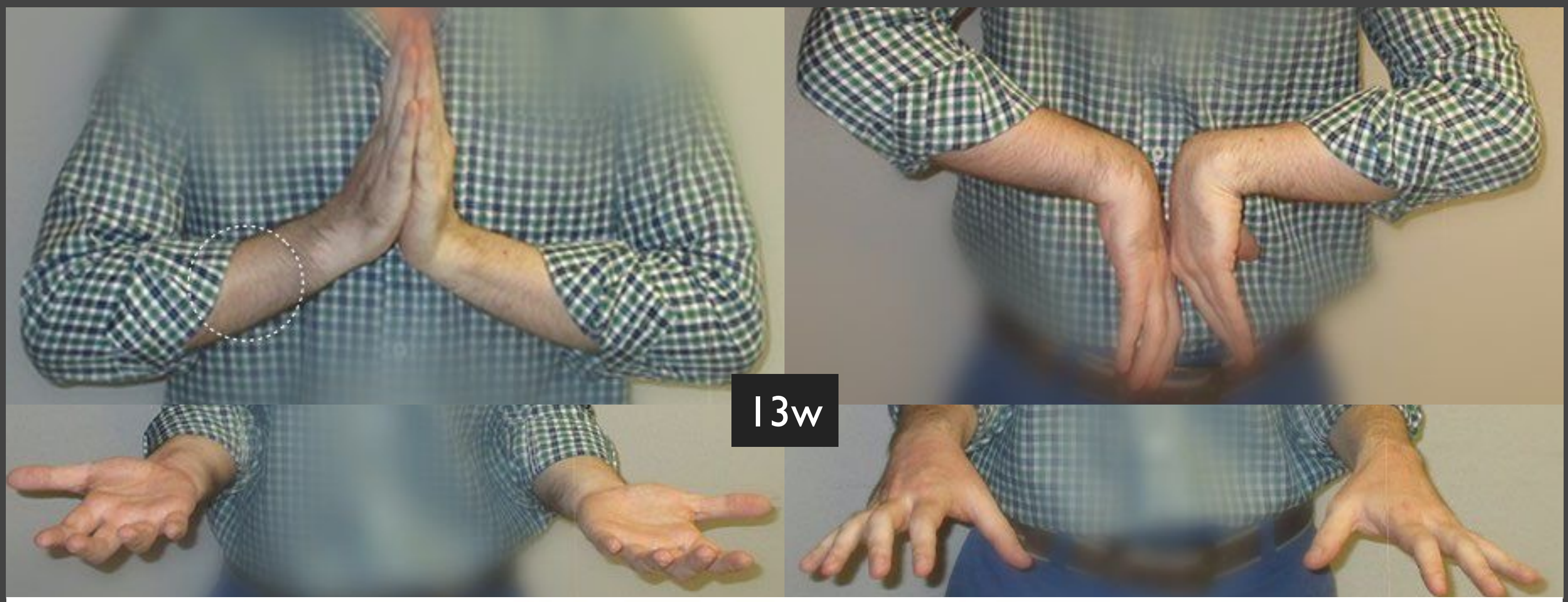

ICUC **Score** ***Functional Limitation:*** **1** (0-4) - ***Pain:*** **0** (0-4)

ICUC Score at 265w Functional limitation: 0 Pain: 0

Quick DASH = 0

ICUC® ID: 23-DC-404 / 75y Female

Complete case data can be accessed by clicking the ***ICUC ID***

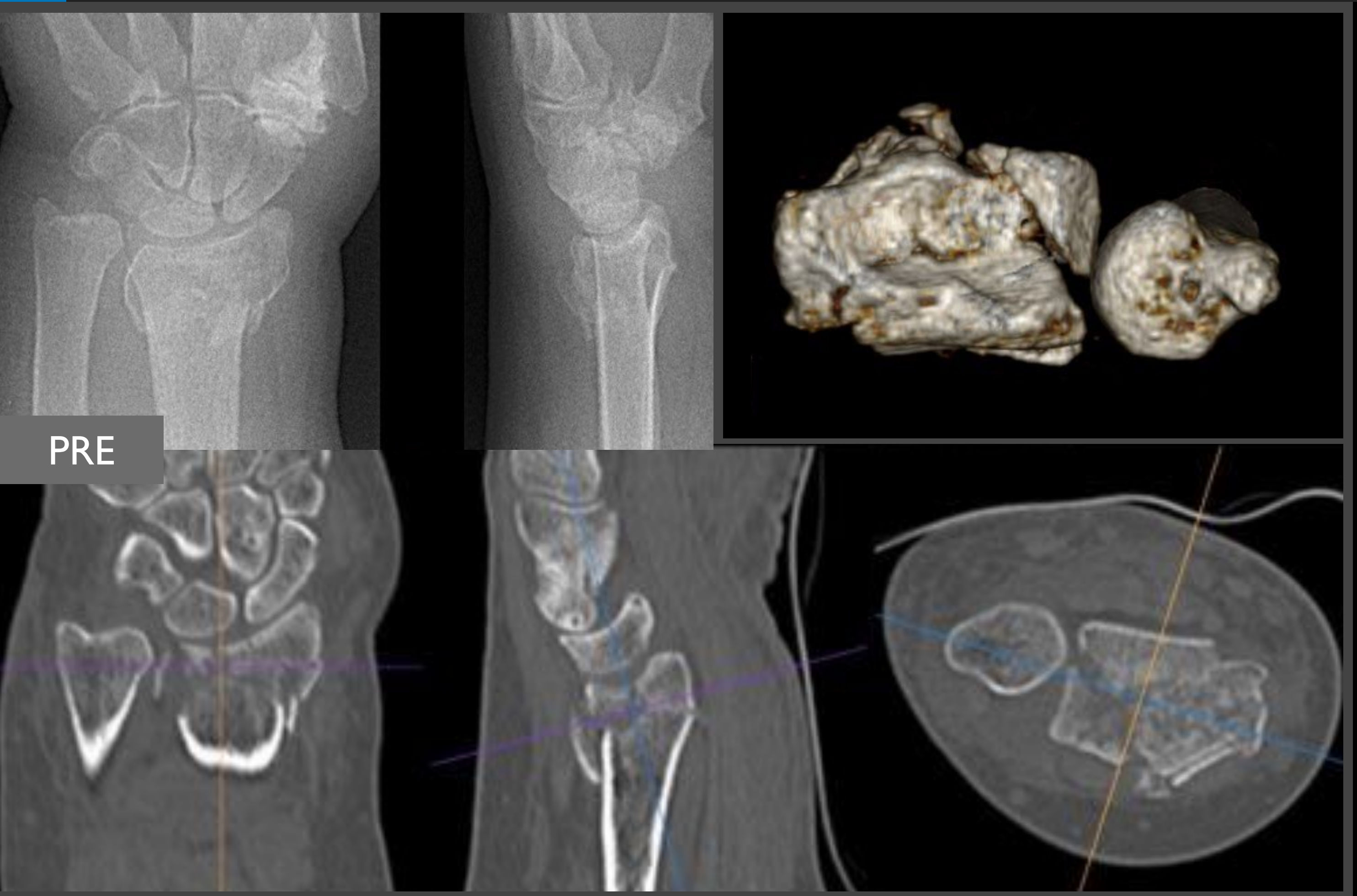

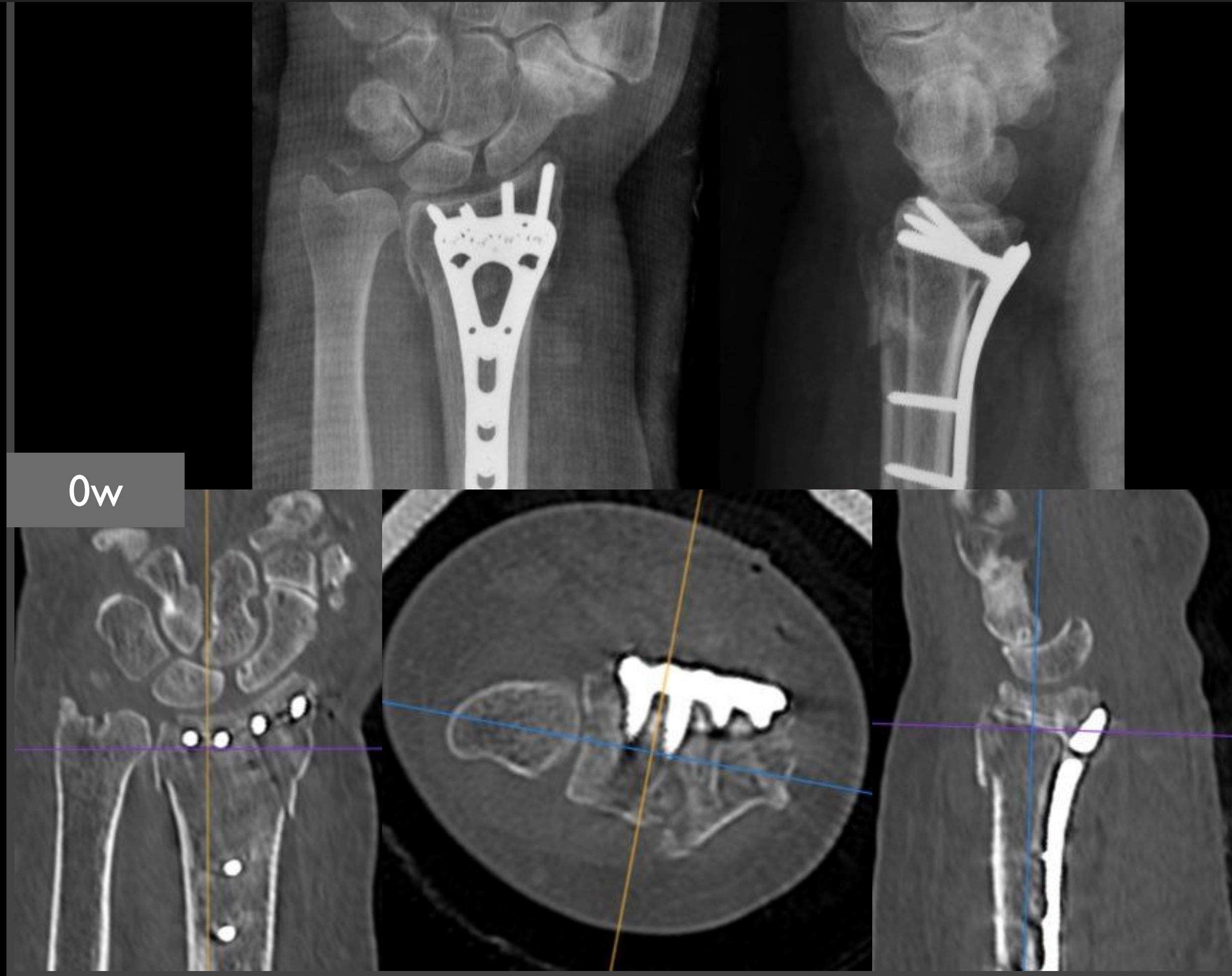

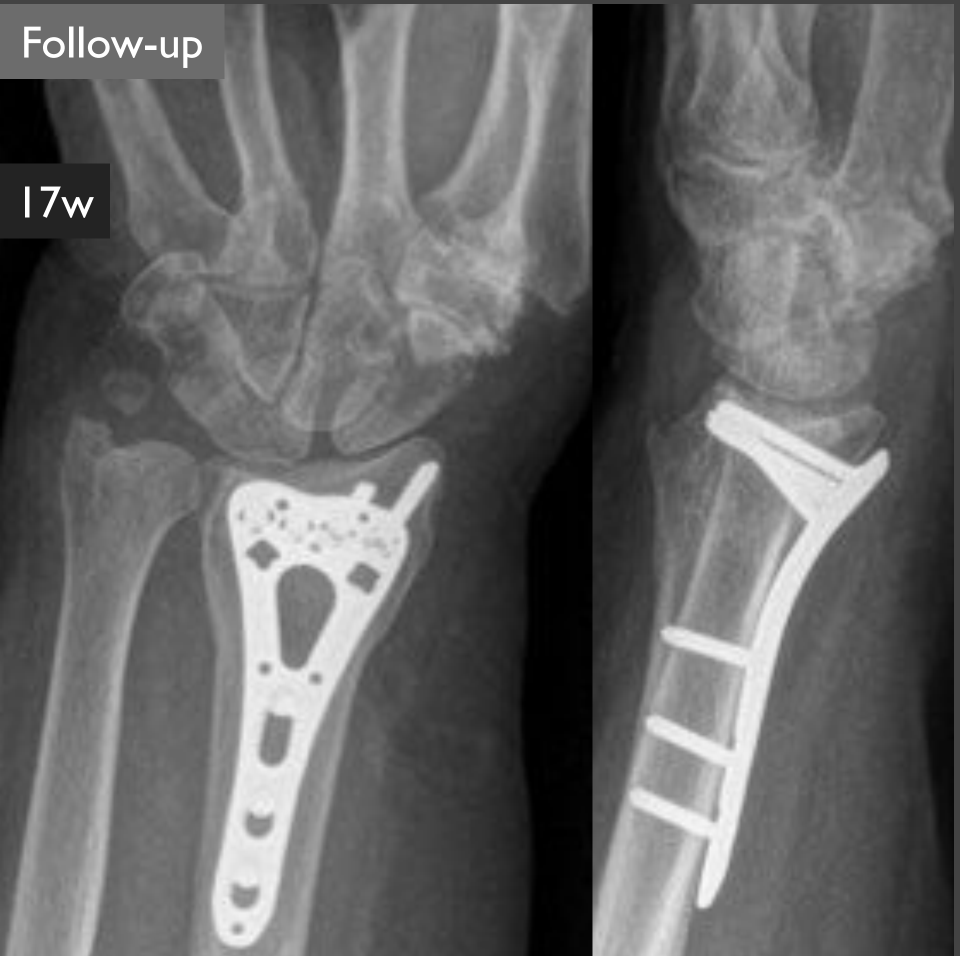

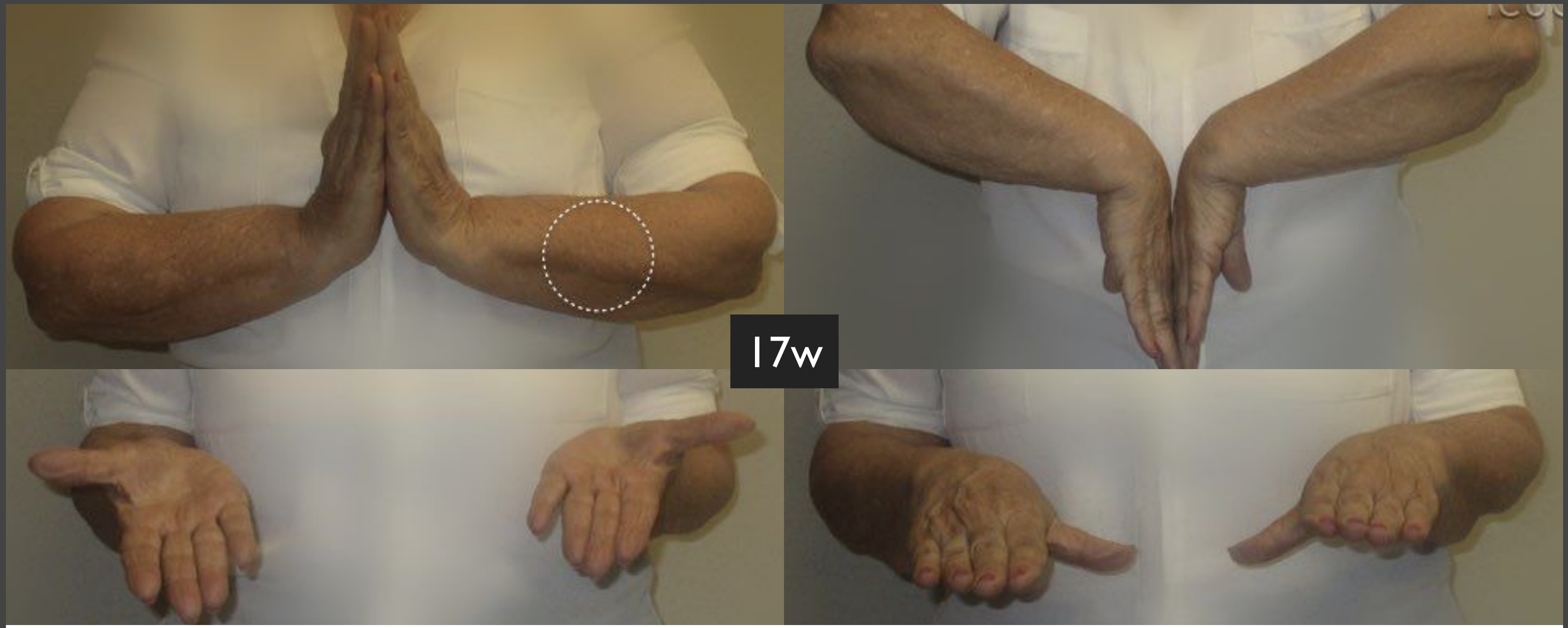

ICUC Score *Functional Limitation:* **1** (0-4) - *Pain:* **0** (0-4)

ICUC Score at 166w Functional limitation: 0 Pain: 0

Quick DASH = 0

ICUC® ID: 23-DC-511 / 60y Female

Complete case data can be accessed by clicking the ***ICUC ID***

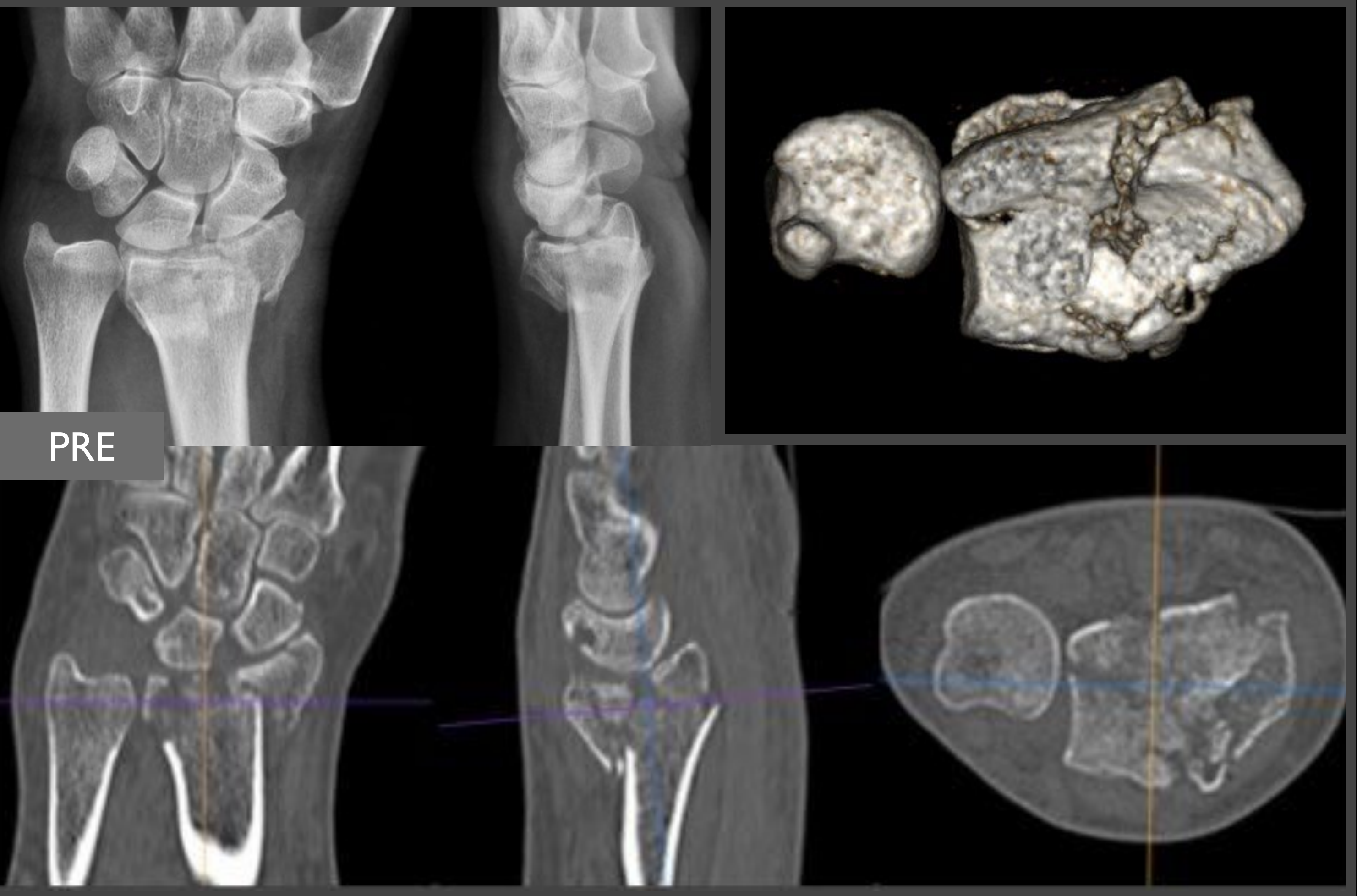

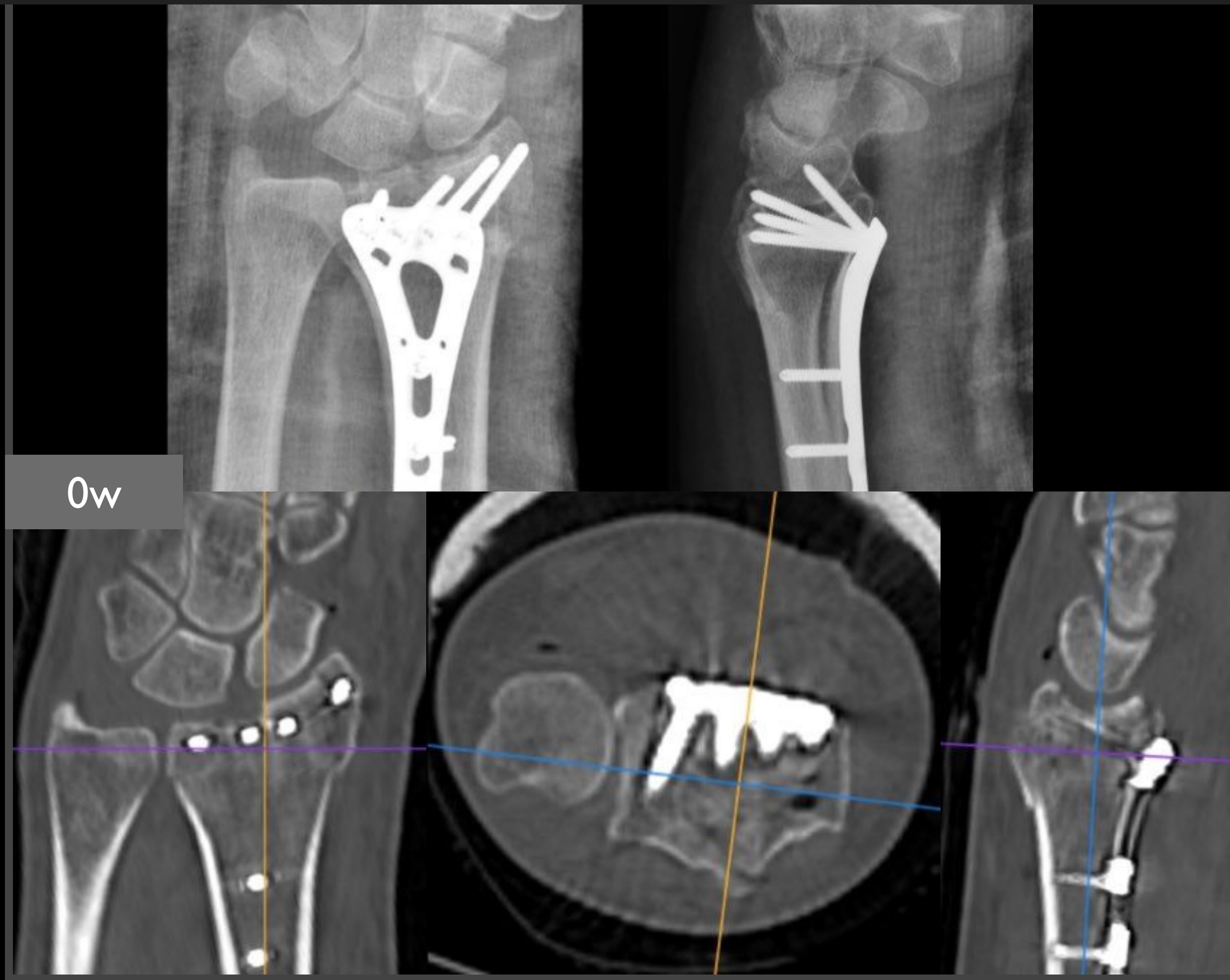

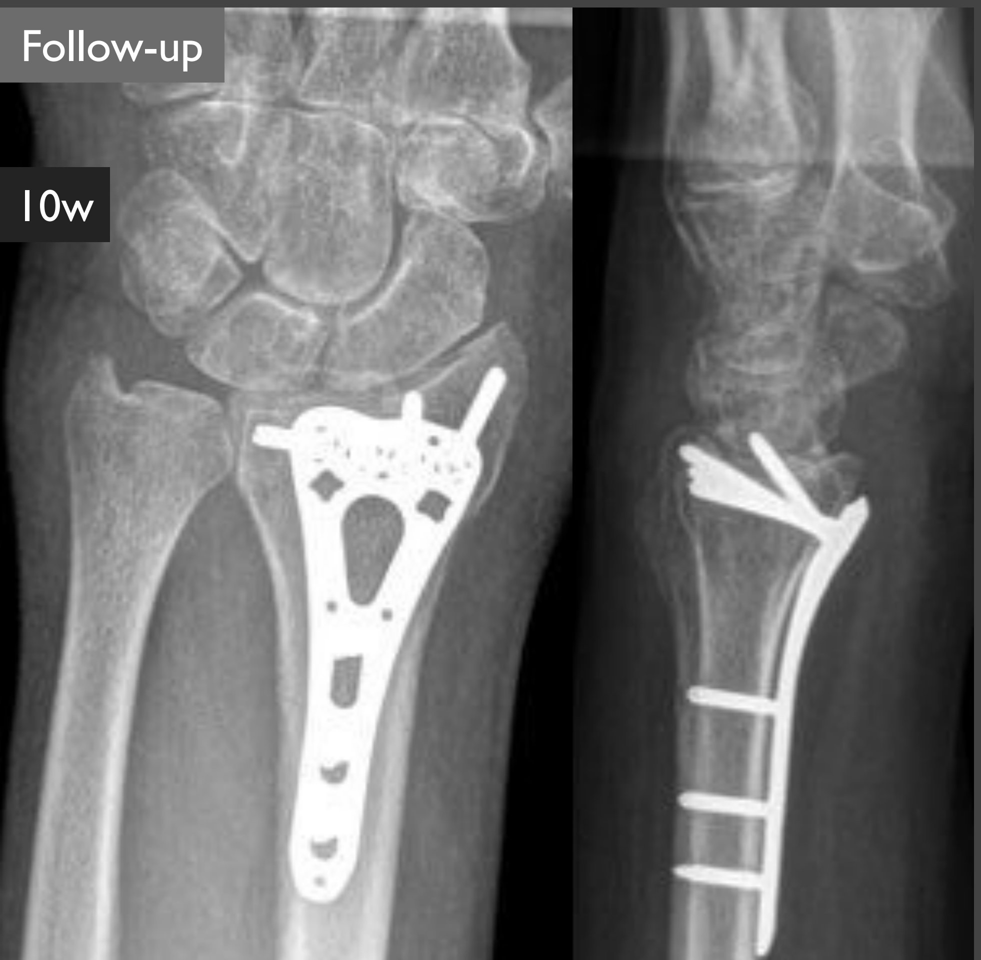

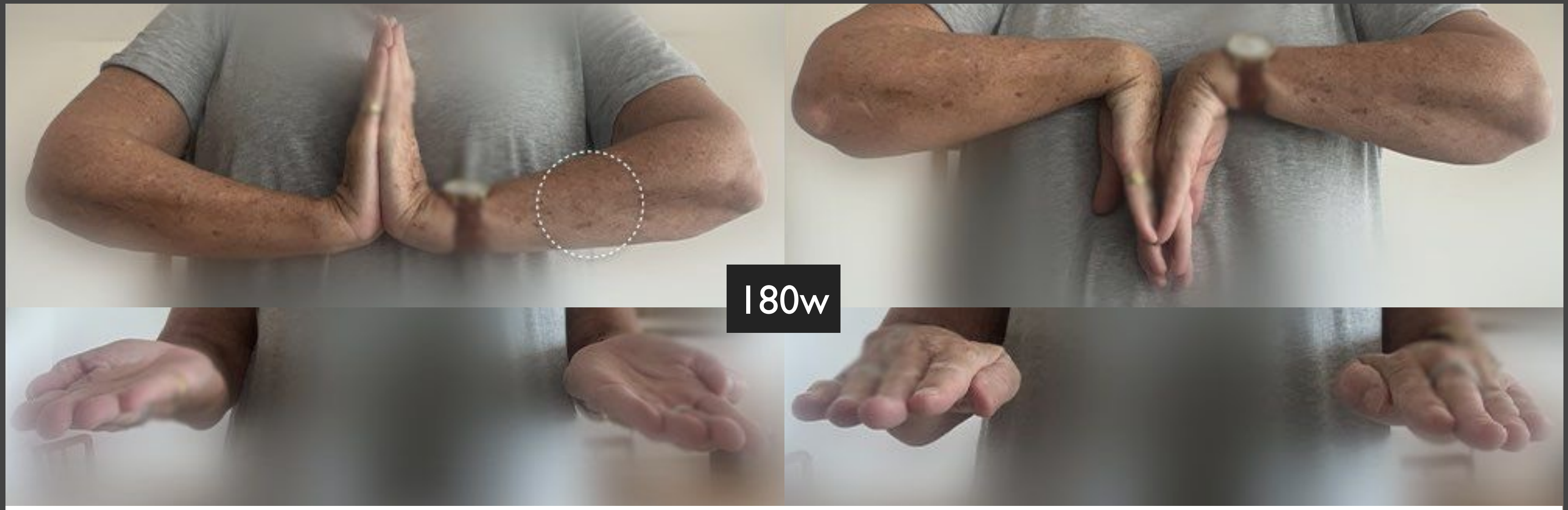

ICUC Score *Functional Limitation:* **0** (0-4) - *Pain:* **0** (0-4)

Quick DASH = 2.2

ICUC® ID: 23-DC-659 / 25y Male

Complete case data can be accessed by clicking the ***ICUC ID***

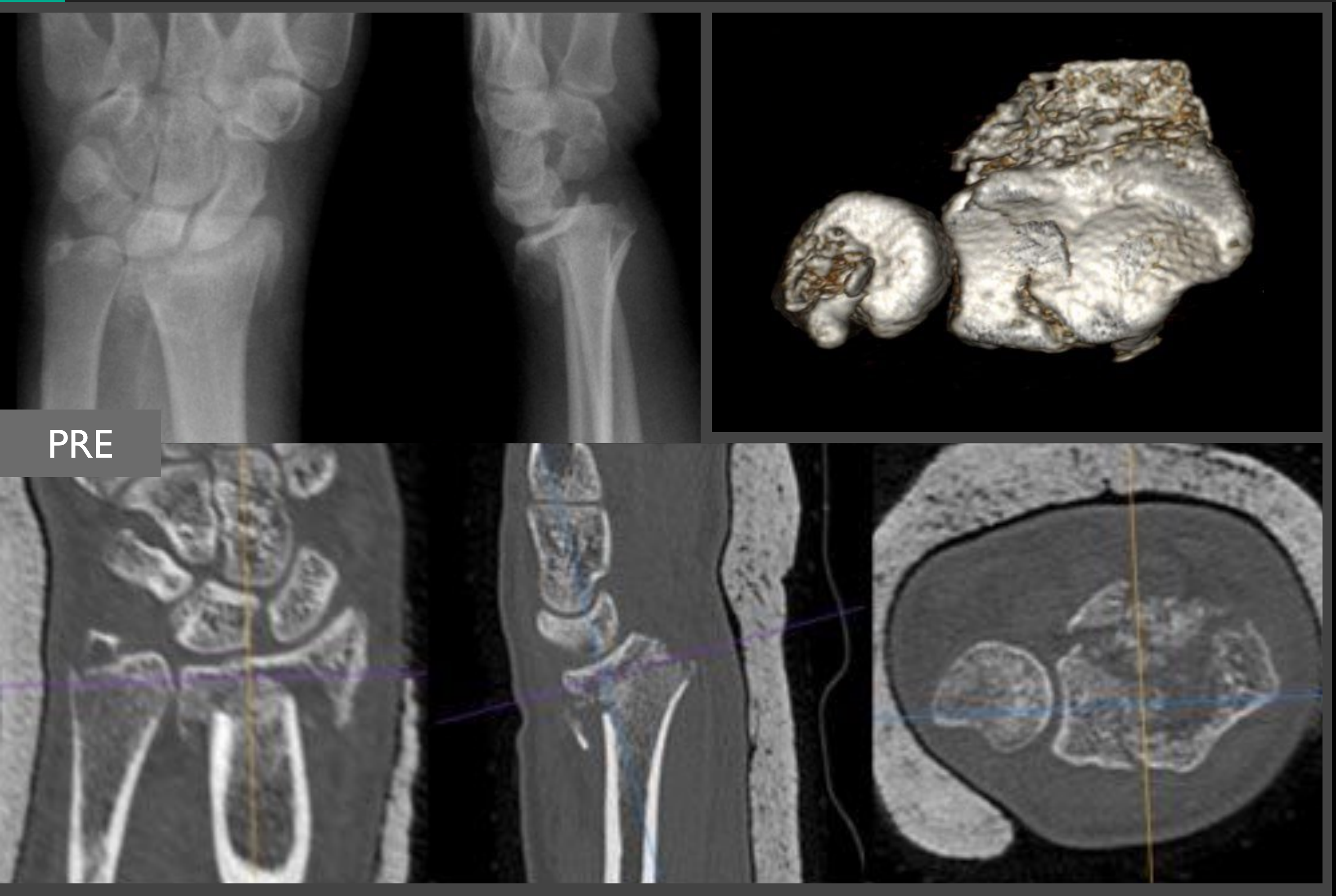

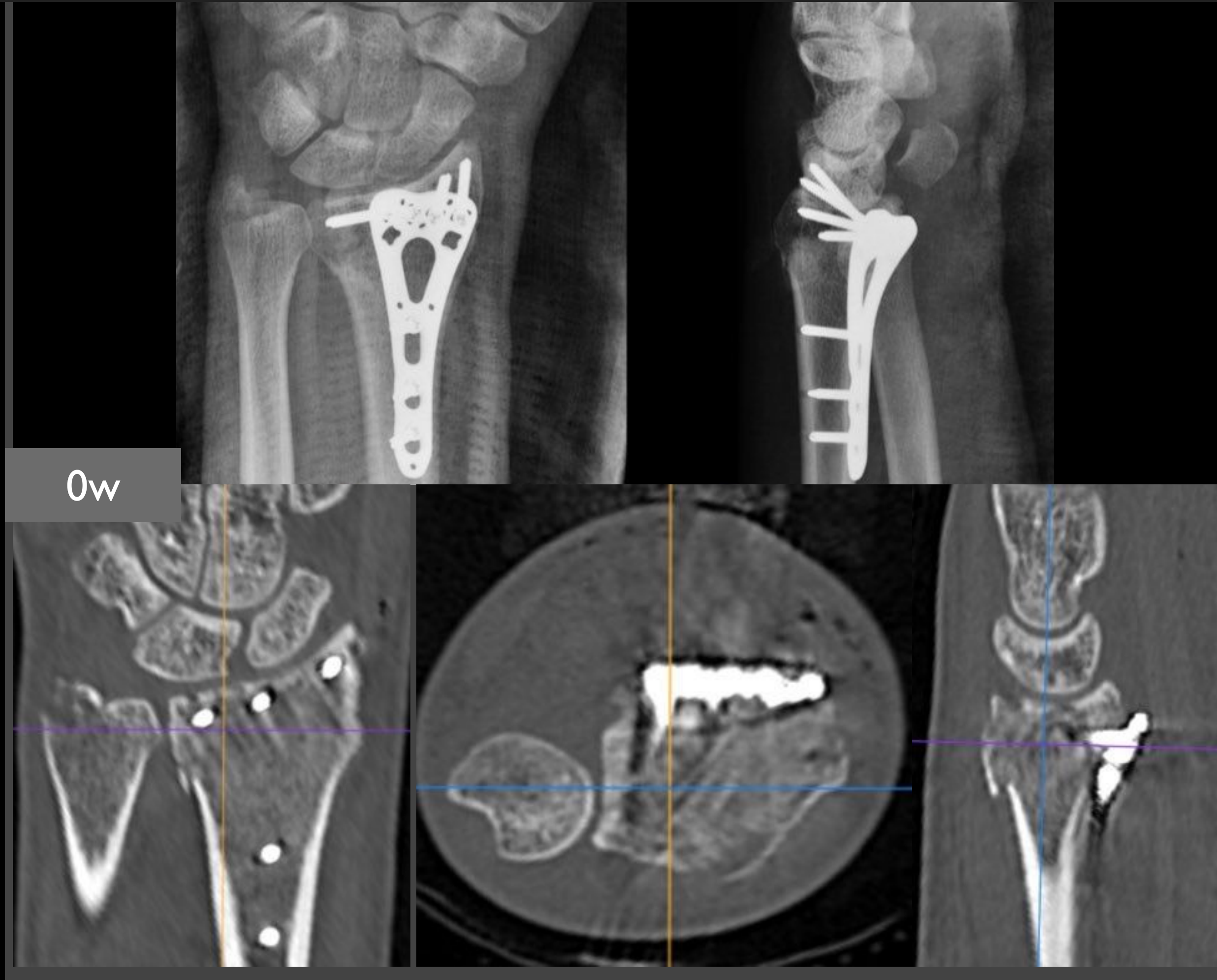

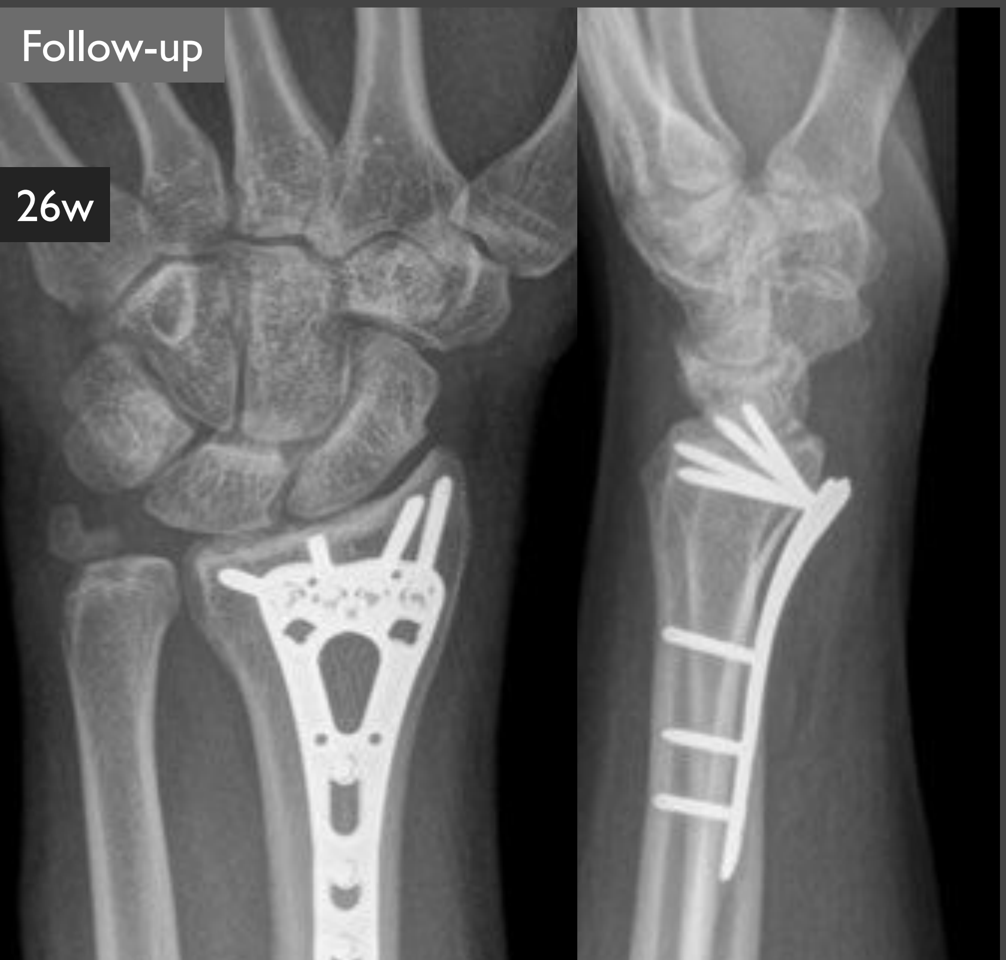

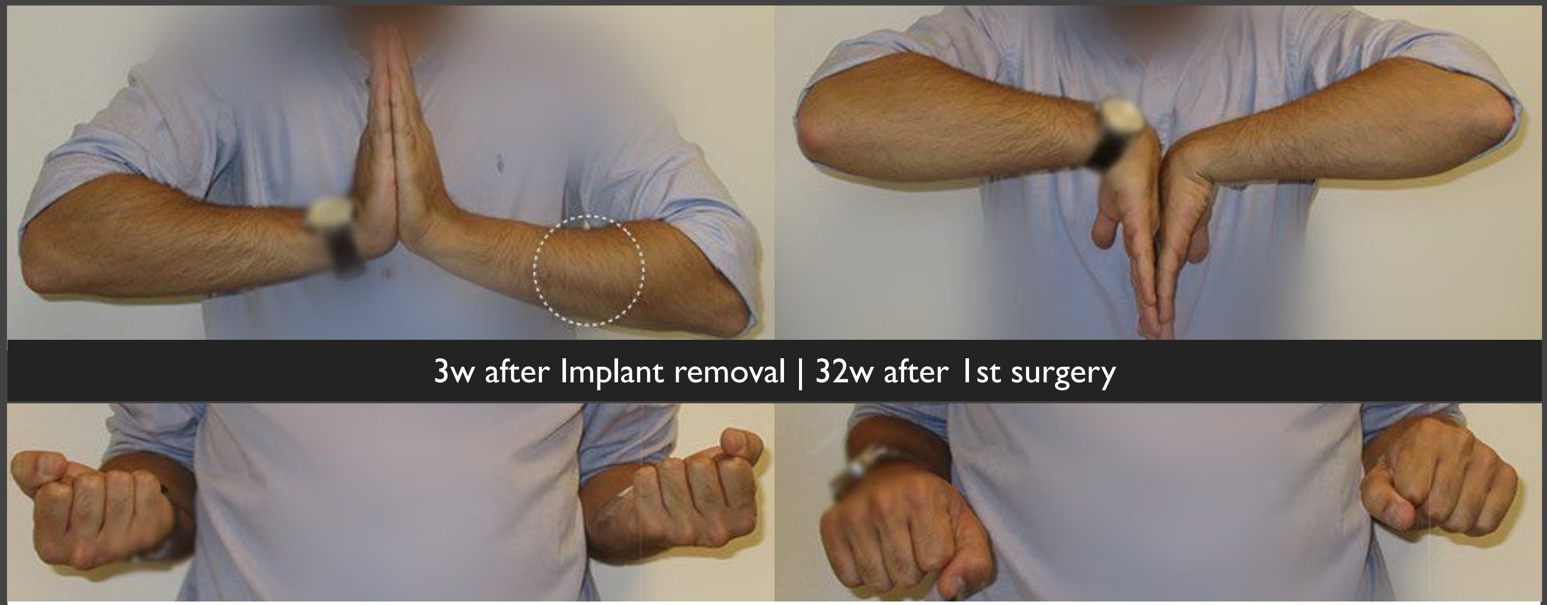

3w after Implant removal | 32w after 1st surgery

ICUC Score ***Functional Limitation:*** **1** (0-4) - ***Pain:*** **1** (0-4)

ICUC Score at 175w Functional limitation: 0 Pain: 0

Quick DASH = 0

ICUC® ID: 23-DC-683 / 55y Male

PRE

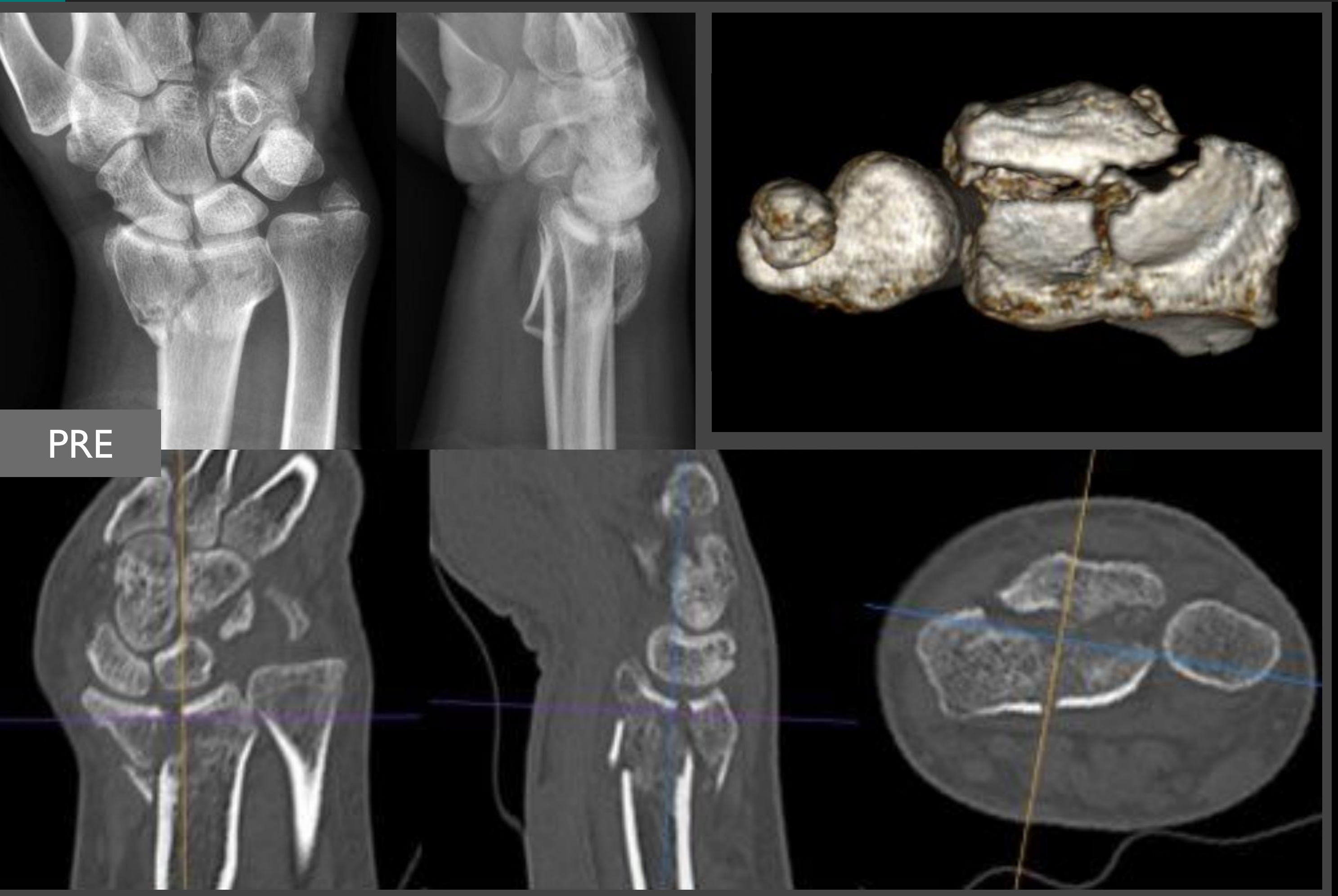

0w

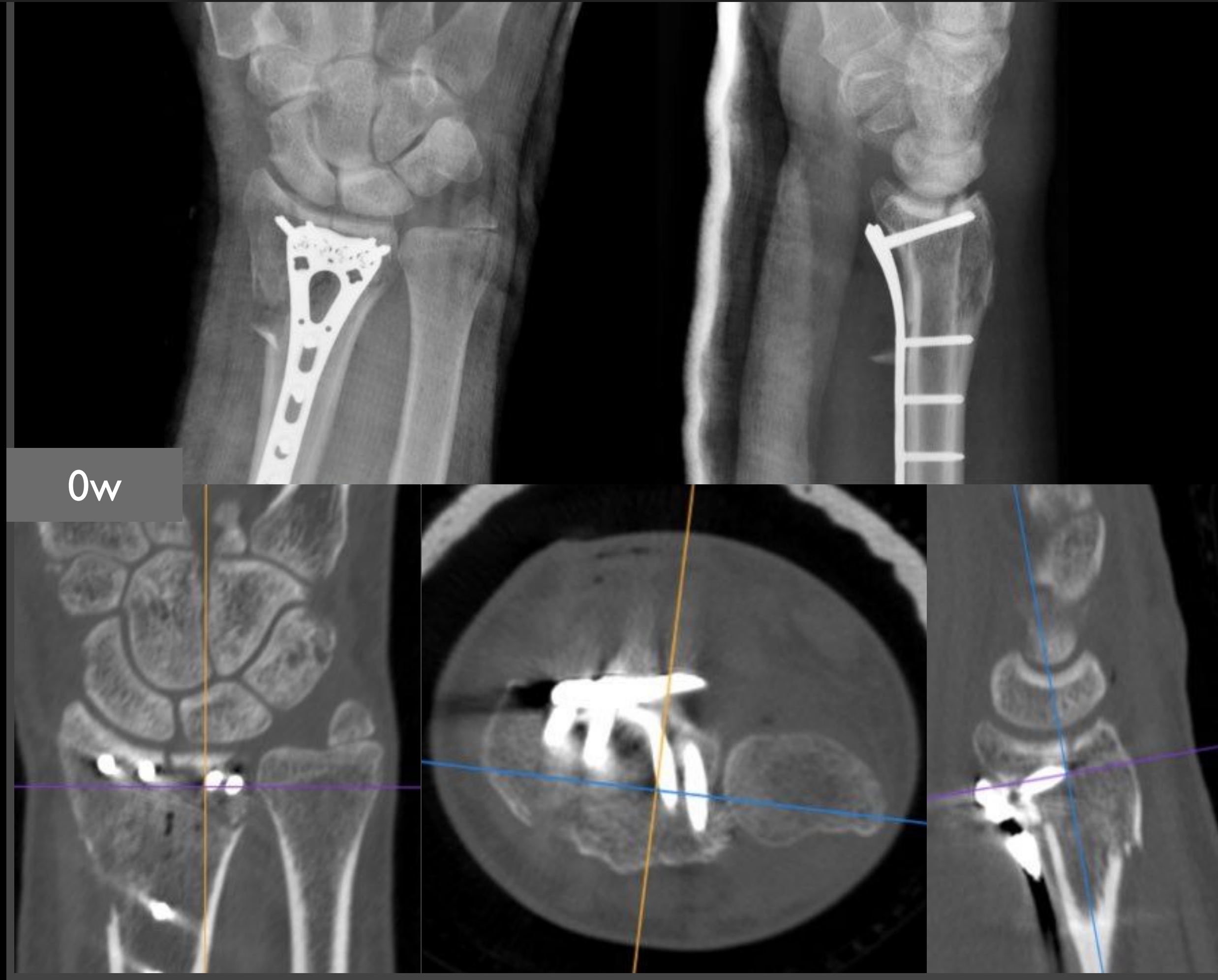

Follow-up

33w

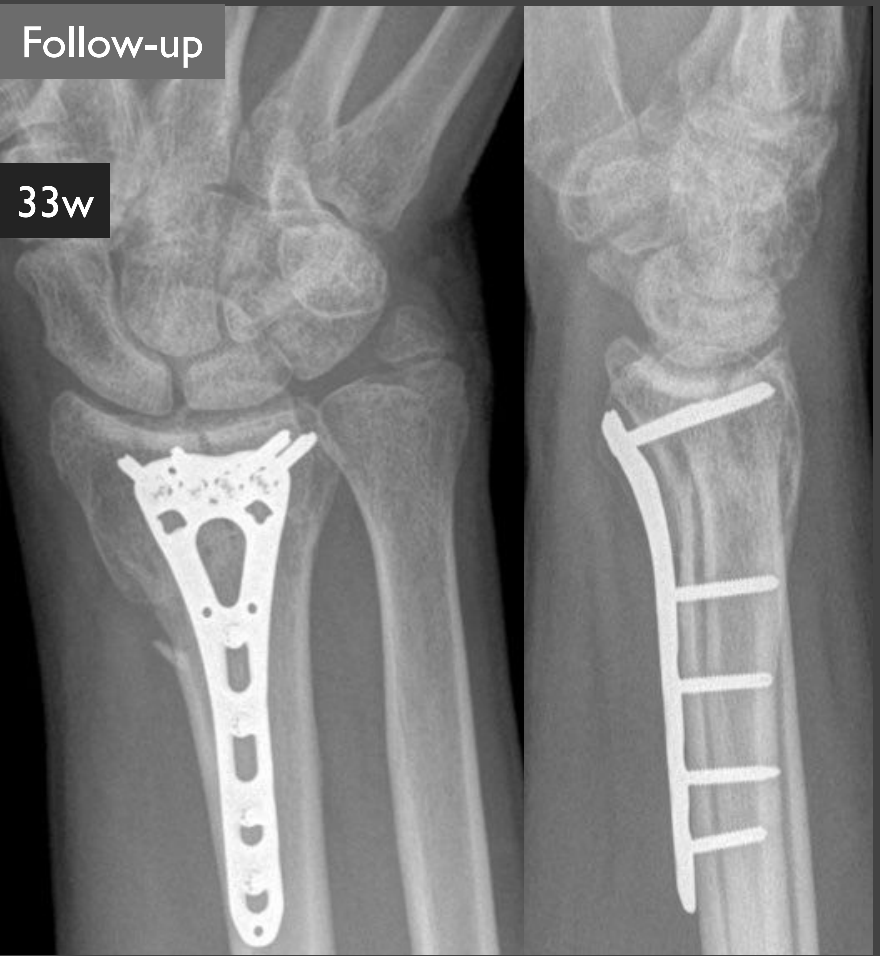

33w

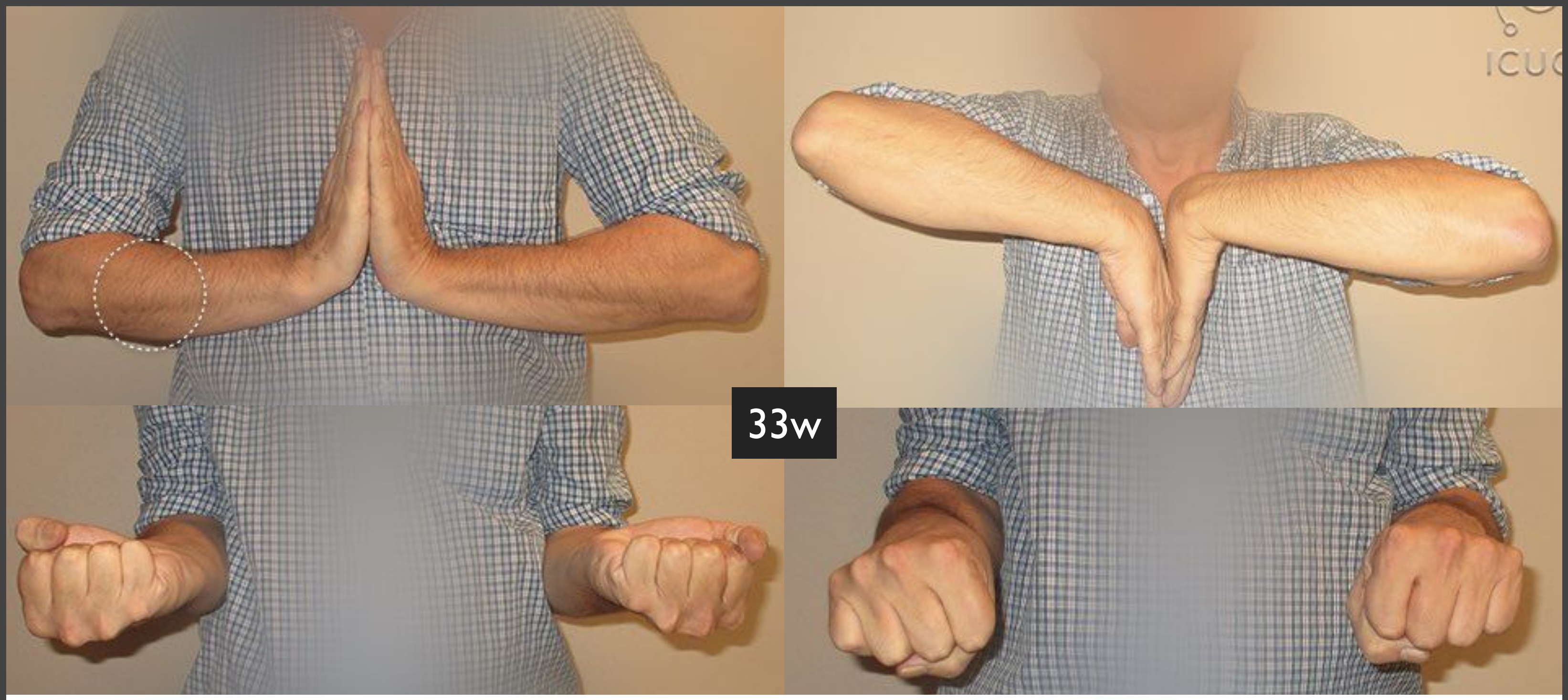

ICUC Score   Functional Limitation: 1 (0-4) - Pain: 1 (0-4)

Quick DASH = 6

ICUC® ID: 23-DC-961 / 70y Female

Complete case data can be accessed by clicking the ***ICUC ID***

PRE

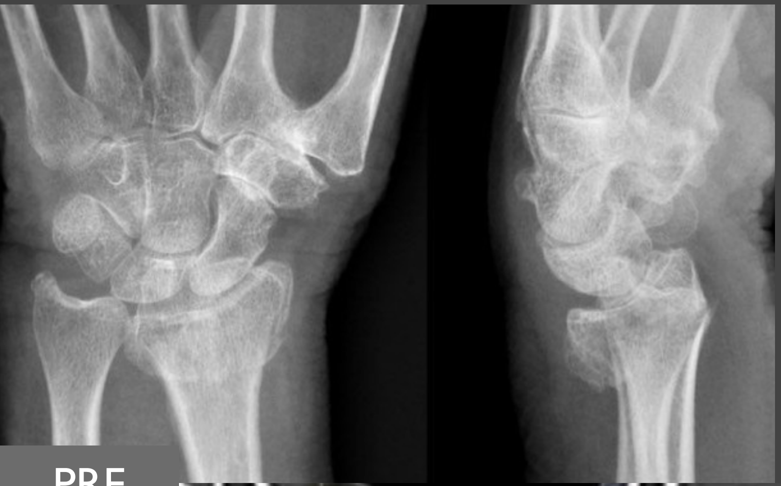

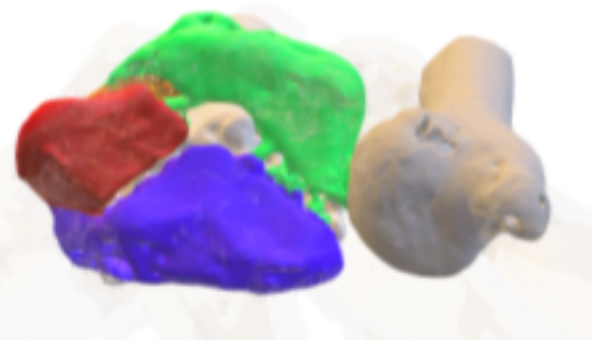

3D Colored Model

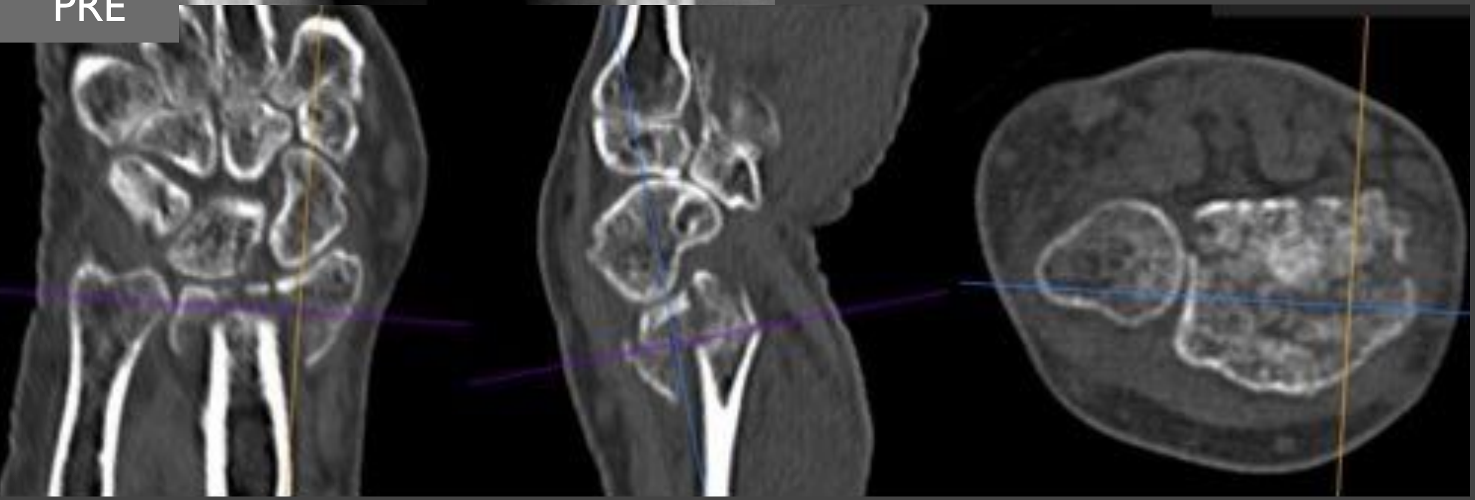

0w

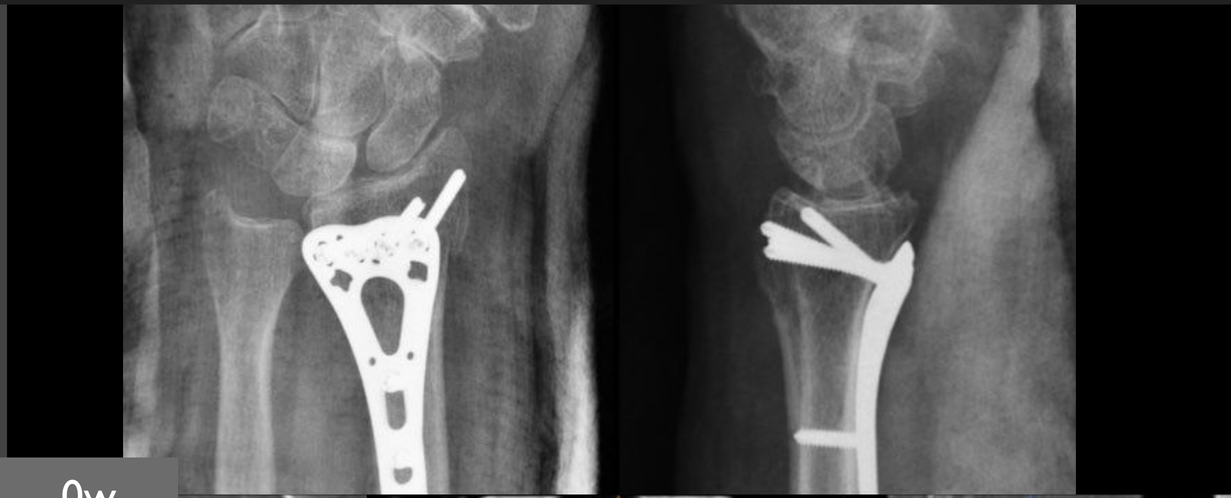

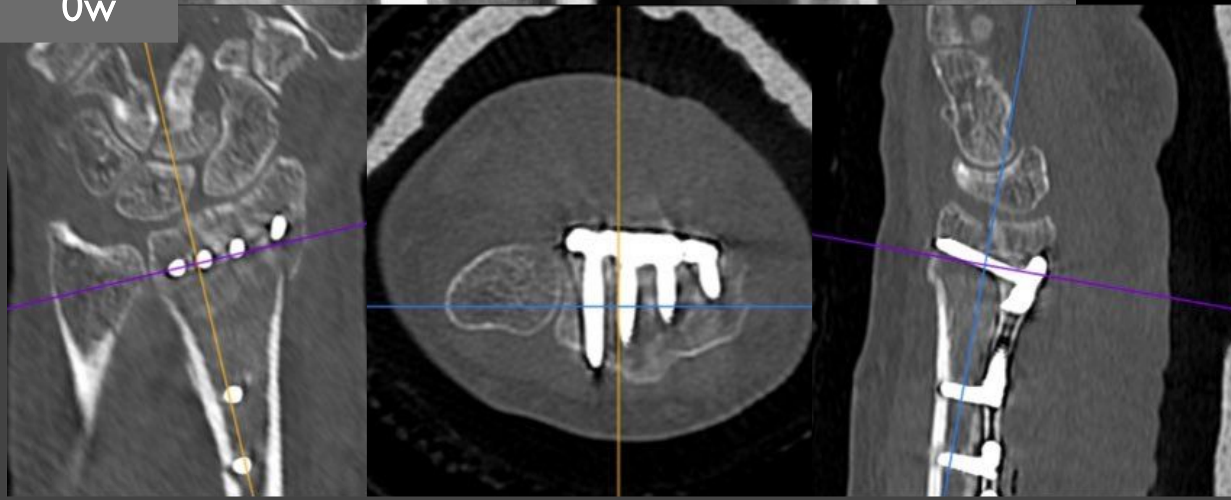

Follow-up

75w

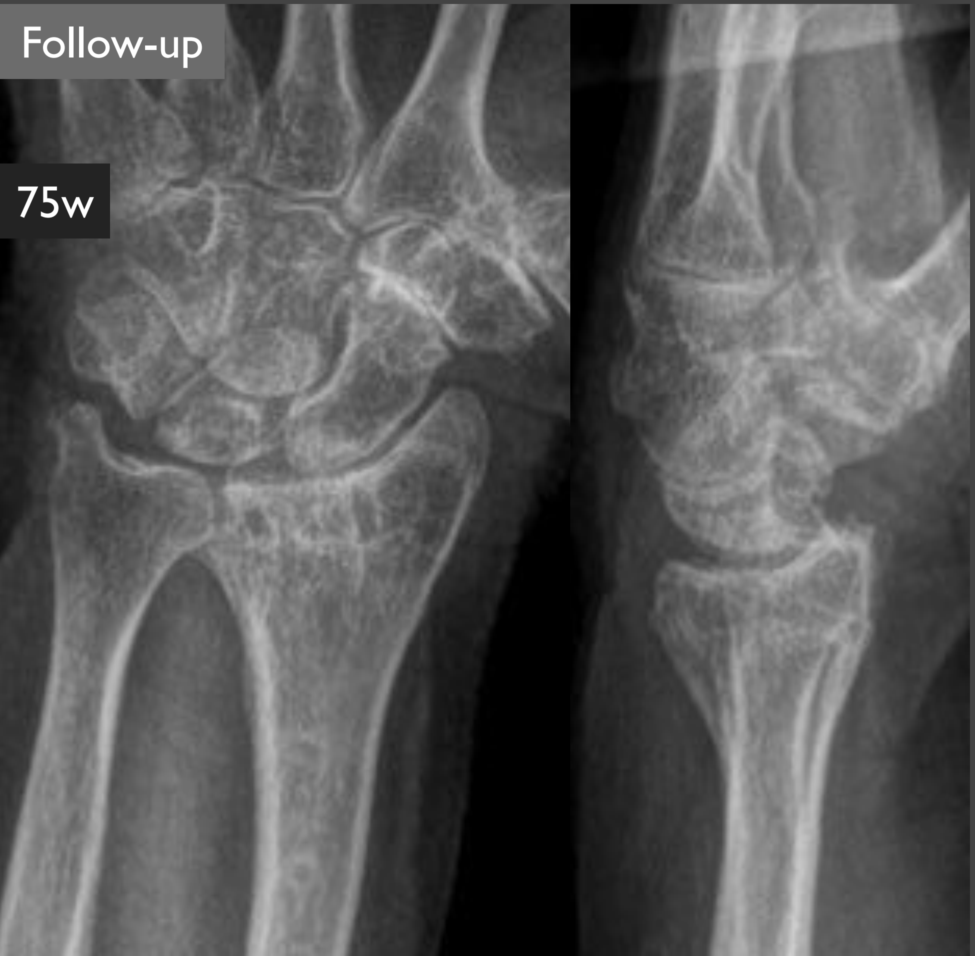

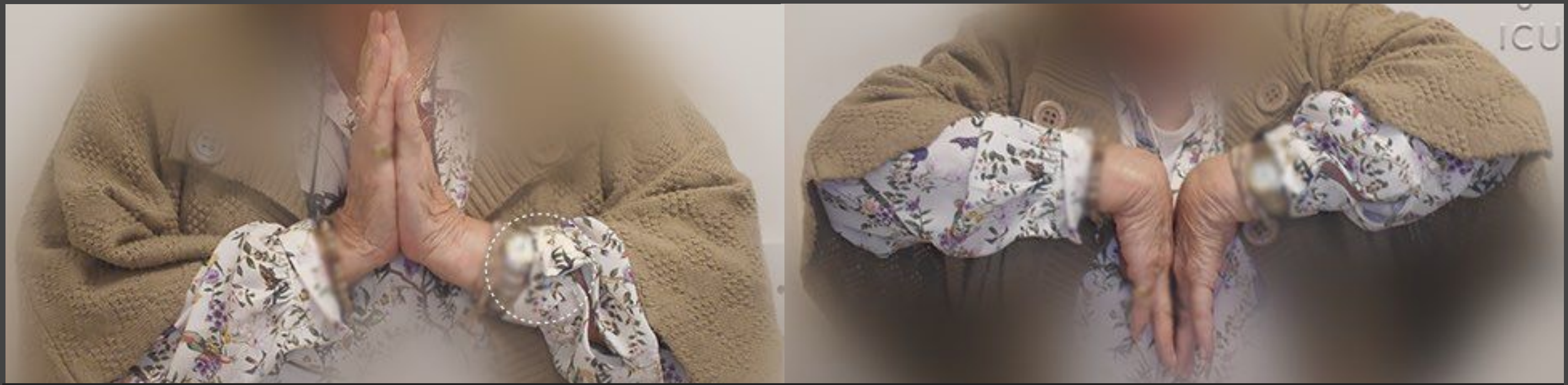

50w after Implant removal | 75w after 1st surgery

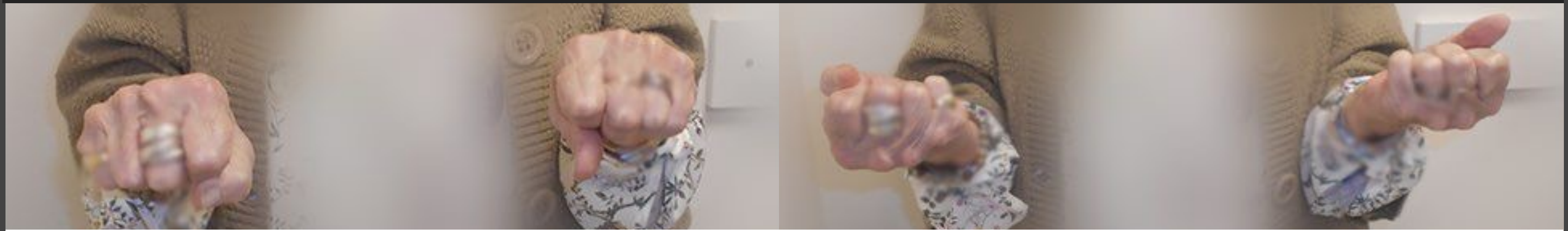

ICUC Score **Functional Limitation: 1** (0-4) - **Pain: 1** (0-4)

Quick DASH = 27

ICUC® ID: 23-DC-055 / 65y Male

Complete case data can be accessed by clicking the ***ICUC ID***

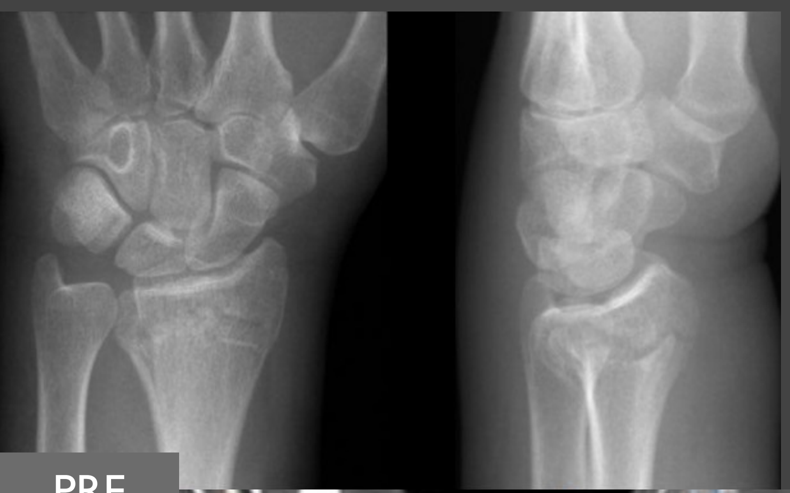

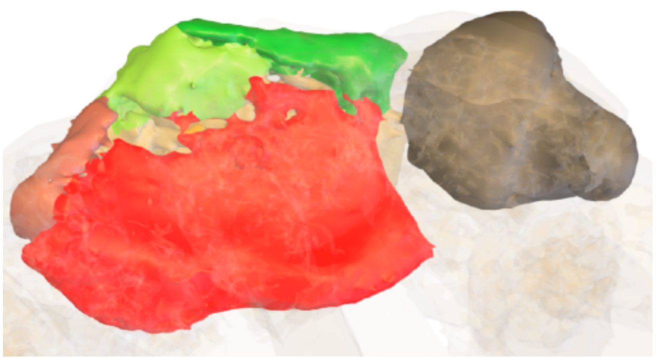

3D Colored Model

PRE

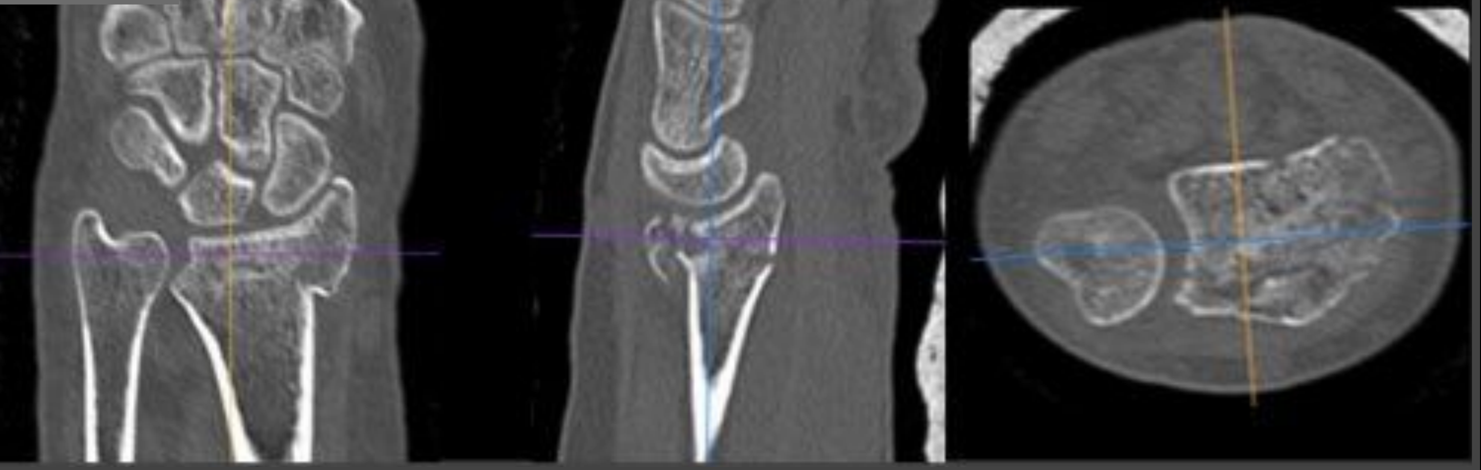

0w

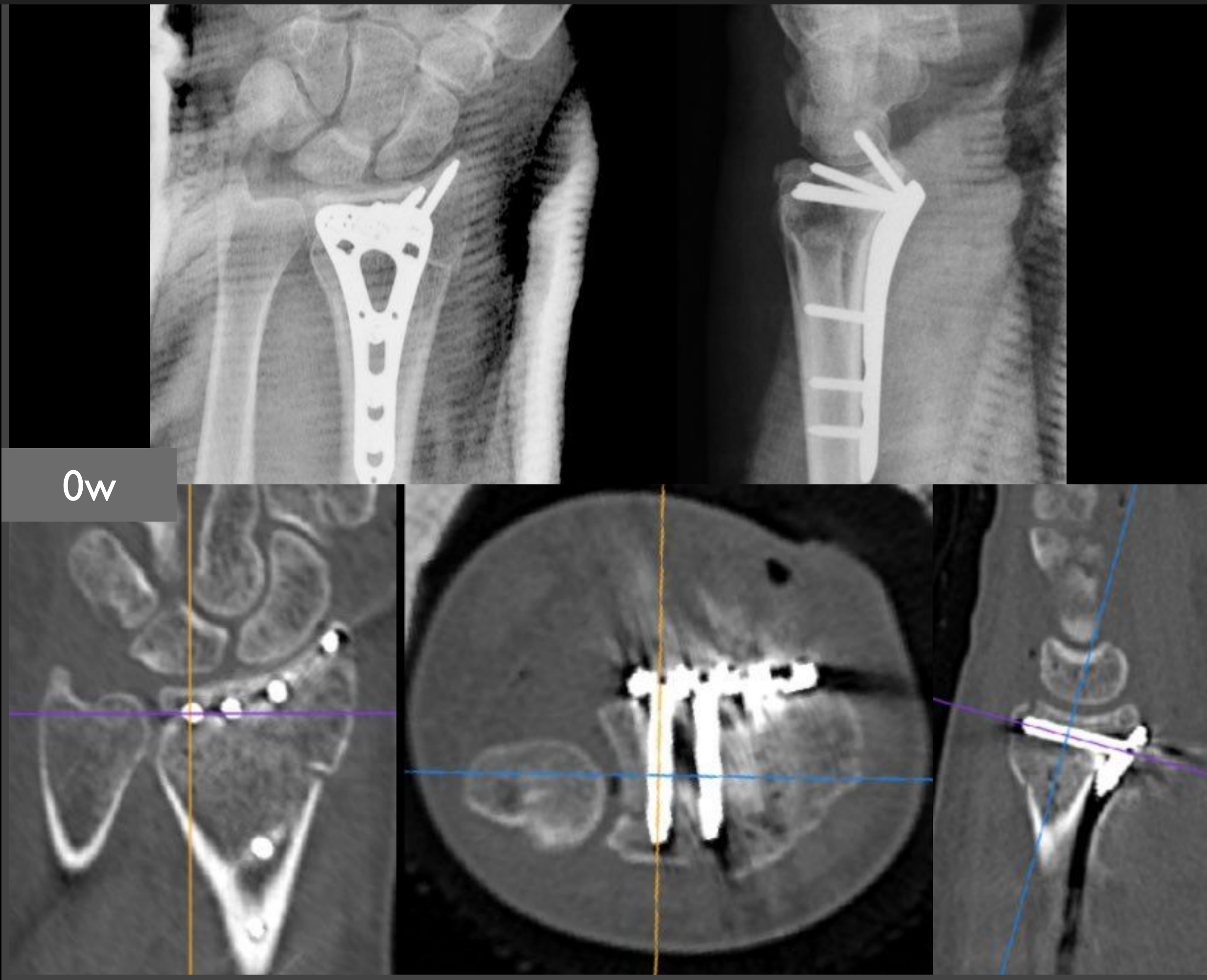

Follow-up

ICUC ***Score*** ***Functional Limitation:*** **0** (0-4) - ***Pain:*** **1** (0-4)

A perfect result is: Zero limitation and Zero pain

Quick DASH = 2

ICUC® ID: 23-DC-175 / 35y Female

*Complete case data can be accessed by clicking the **ICUC ID***

PRE

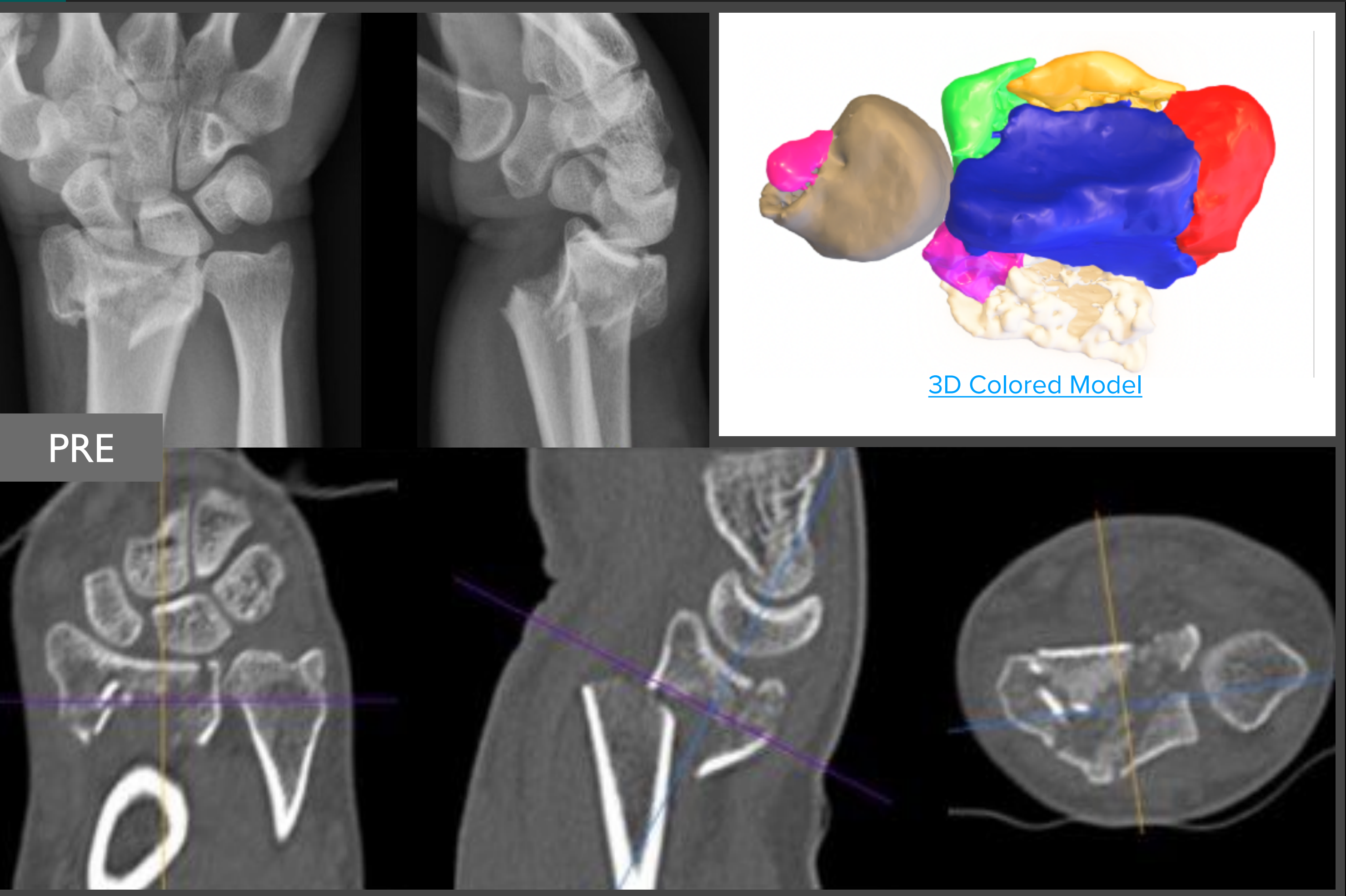

0w

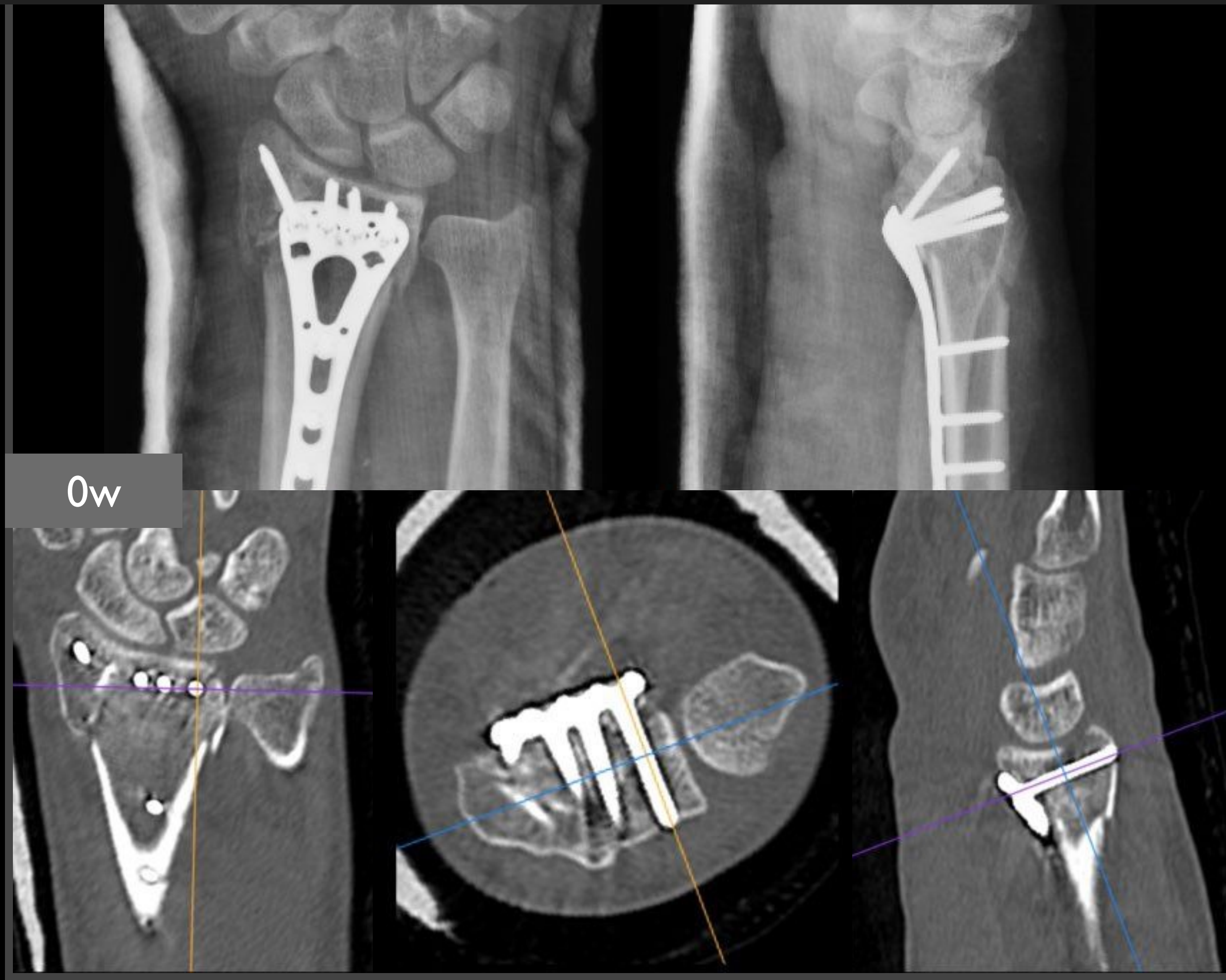

Follow-up

8w

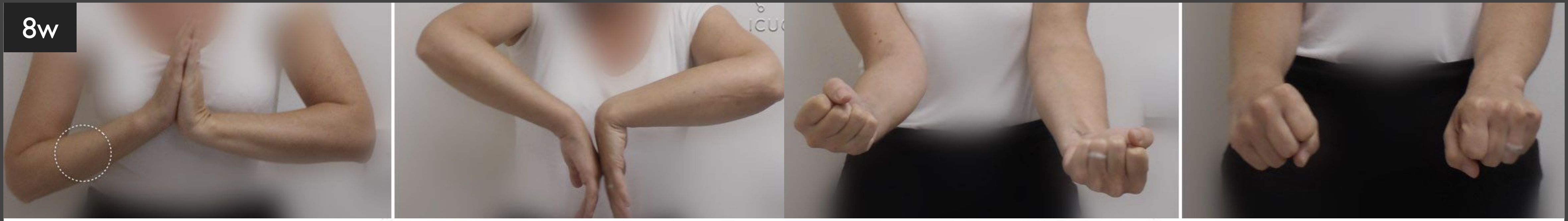

ICUC Score **Functional Limitation: 2** (0-4) - **Pain: 0** (0-4)

ICUC Score at 49w Functional limitation: 0 Pain: 0

Quick DASH = 6.8

ICUC® ID: 23-DC-599 / 20y Male

Complete case data can be accessed by clicking the ***ICUC ID***

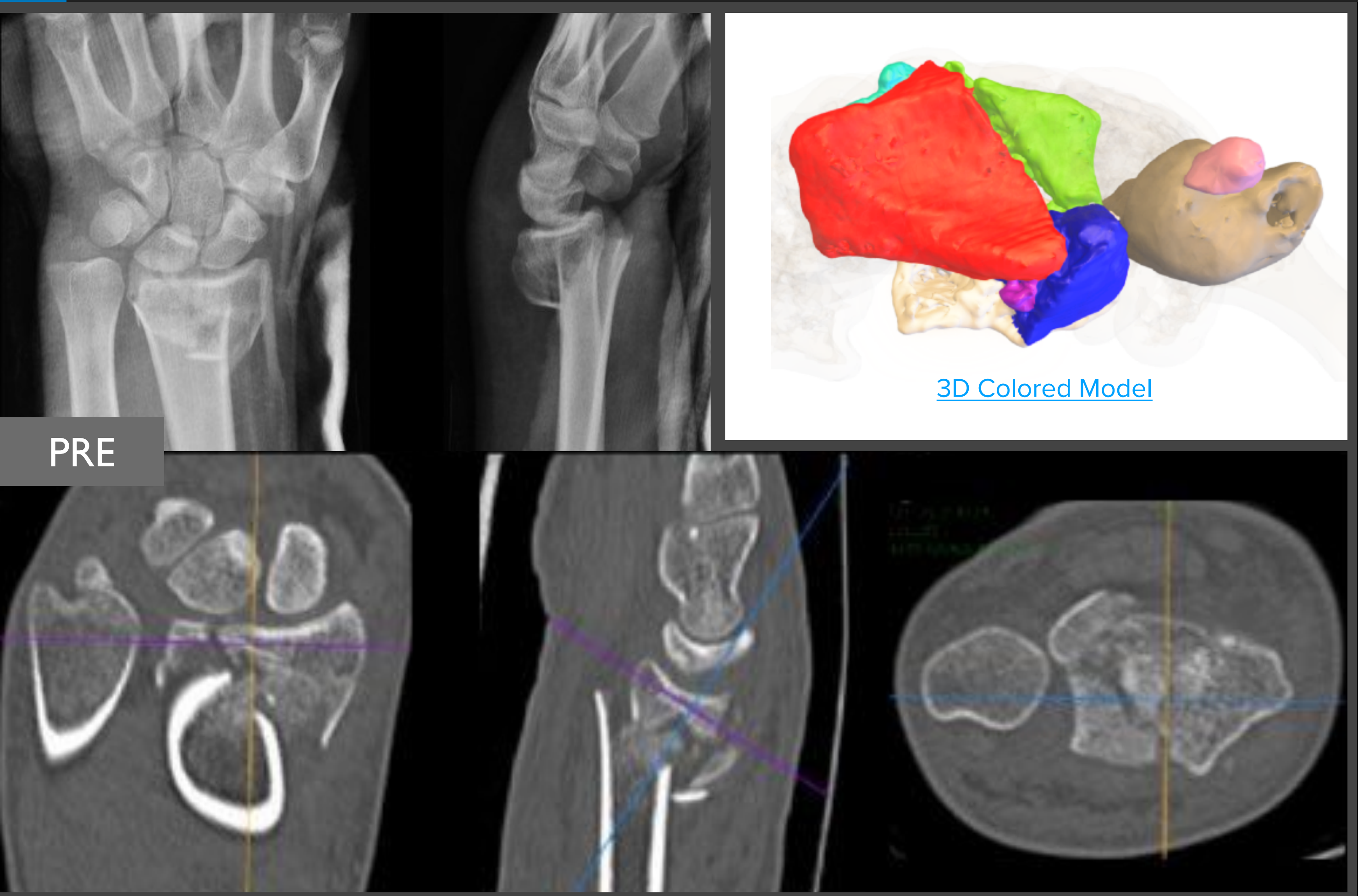

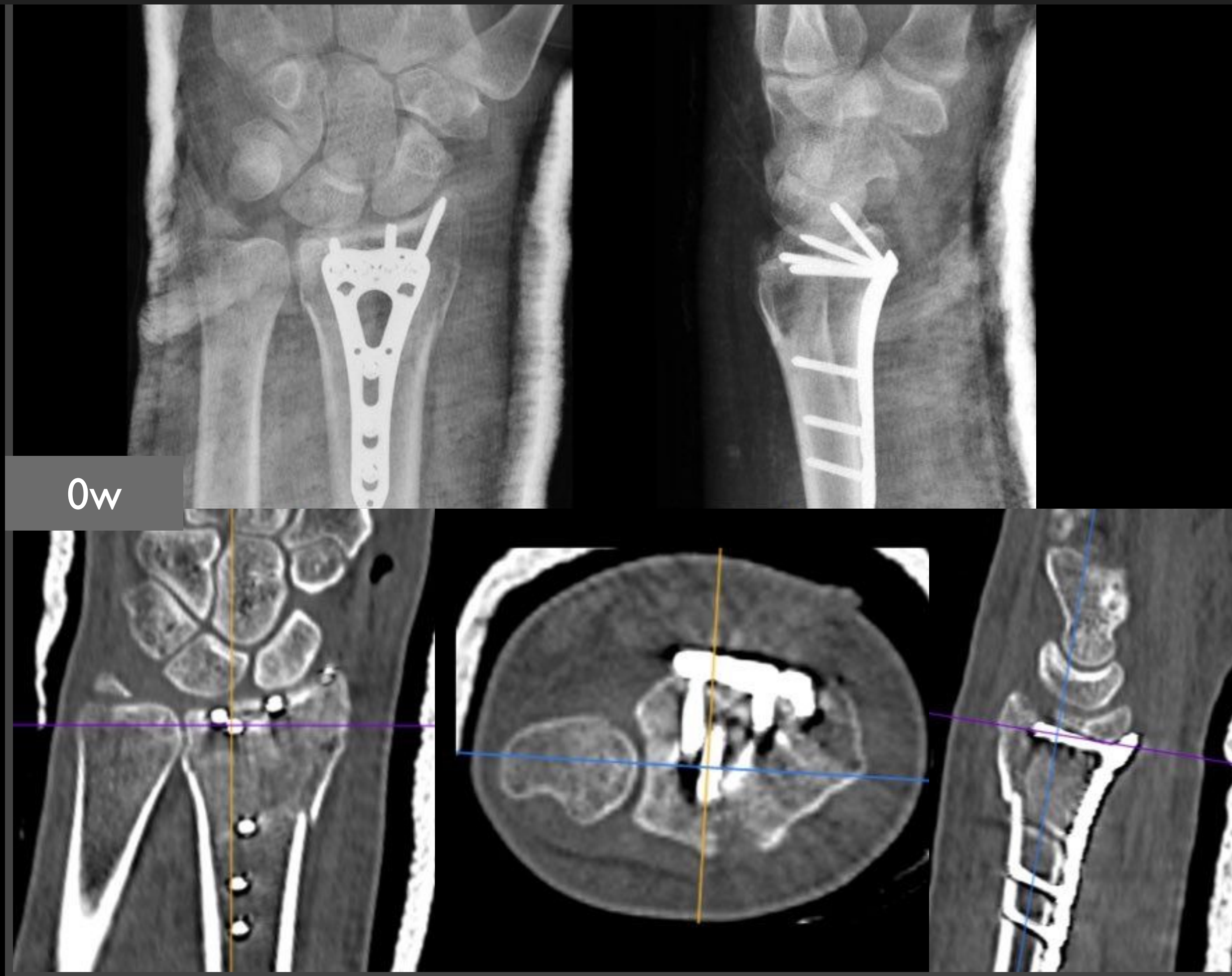

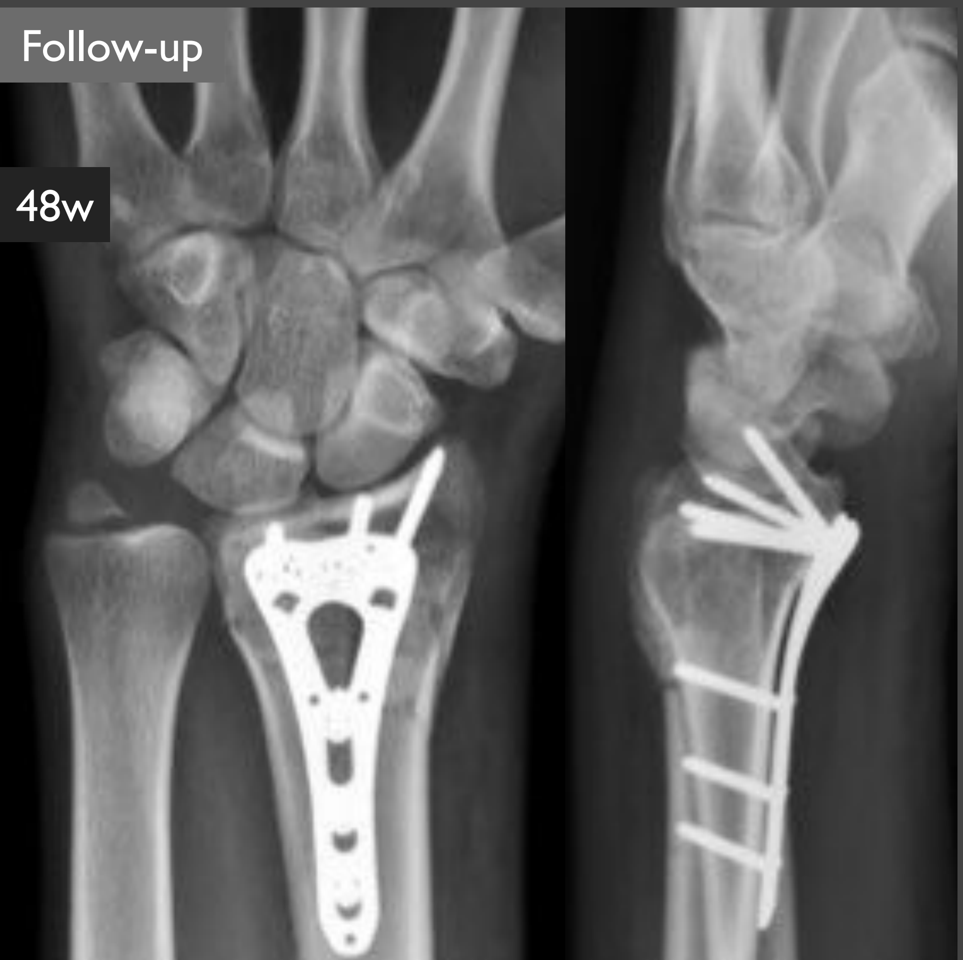

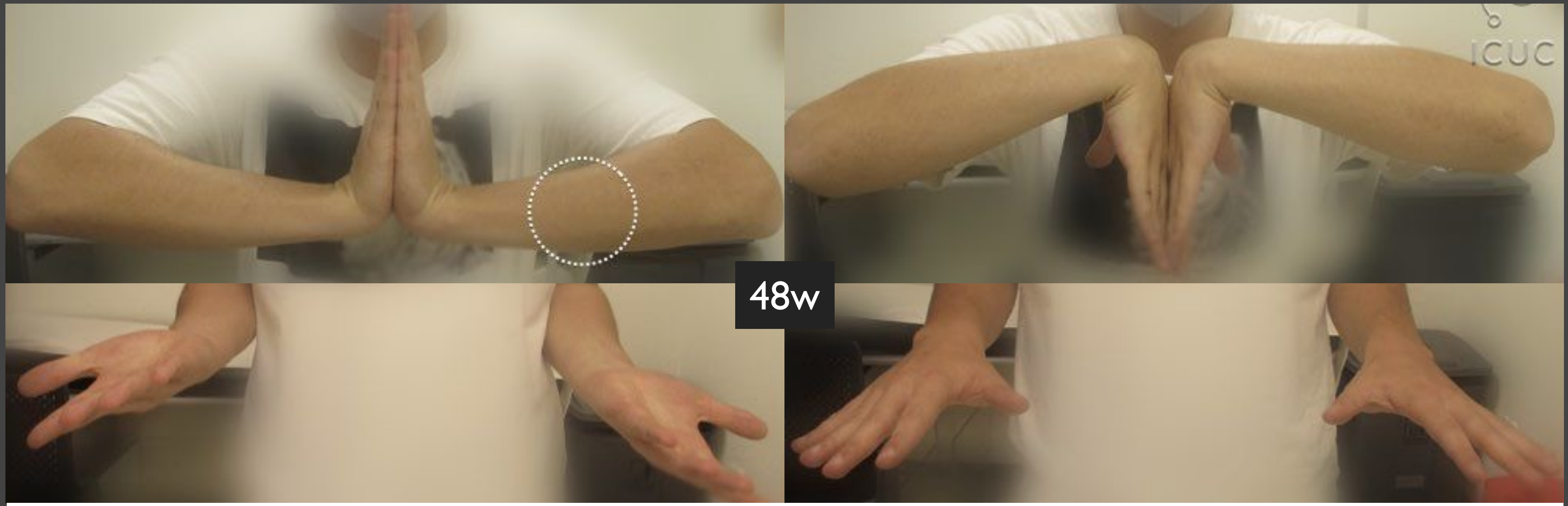

ICUC Score **Functional Limitation:** 0 (0-4) - **Pain:** 0 (0-4)

Quick DASH = 0

ICUC® ID: 23-DC-855 / 50y Female

Complete case data can be accessed by clicking the ***ICUC ID***

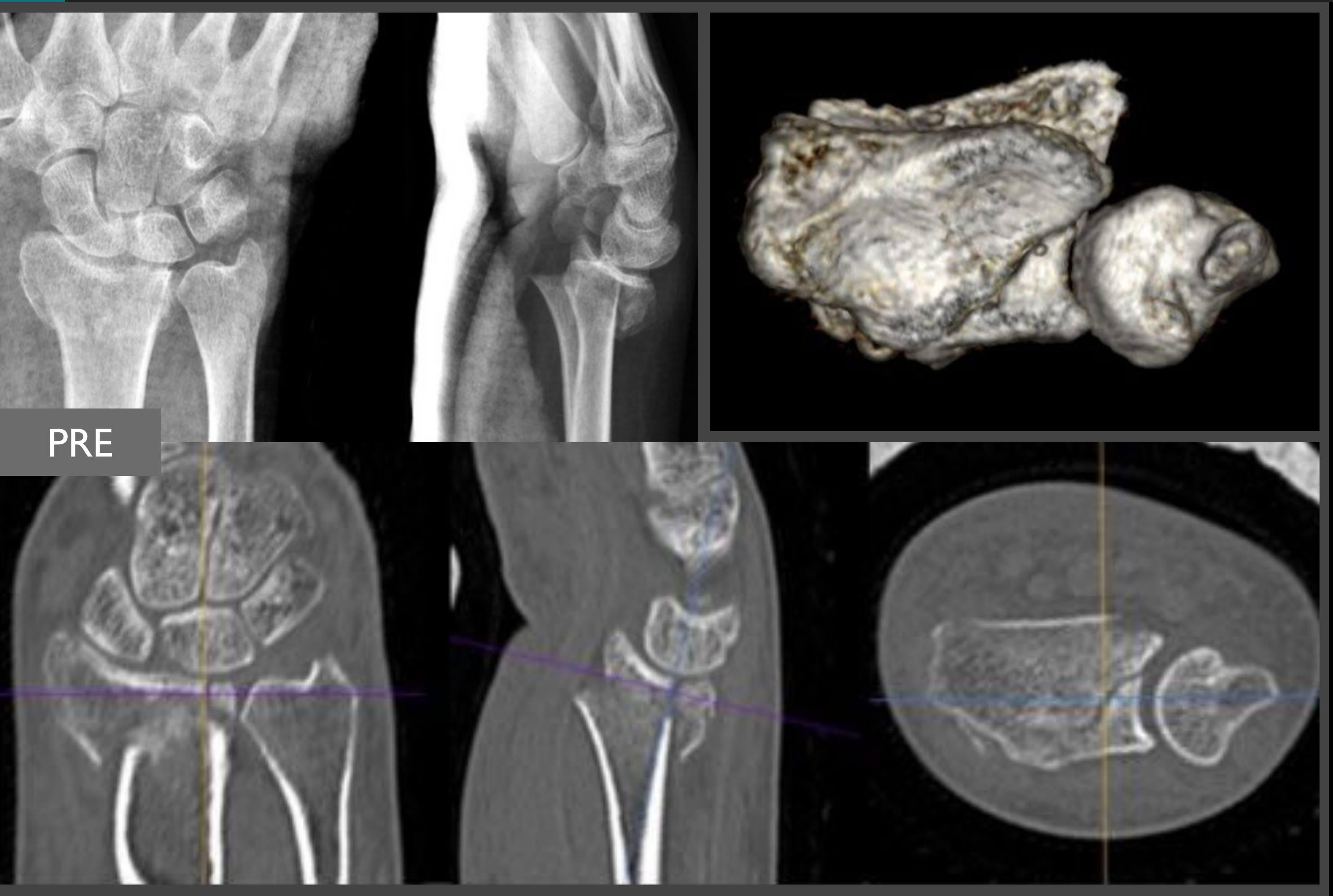

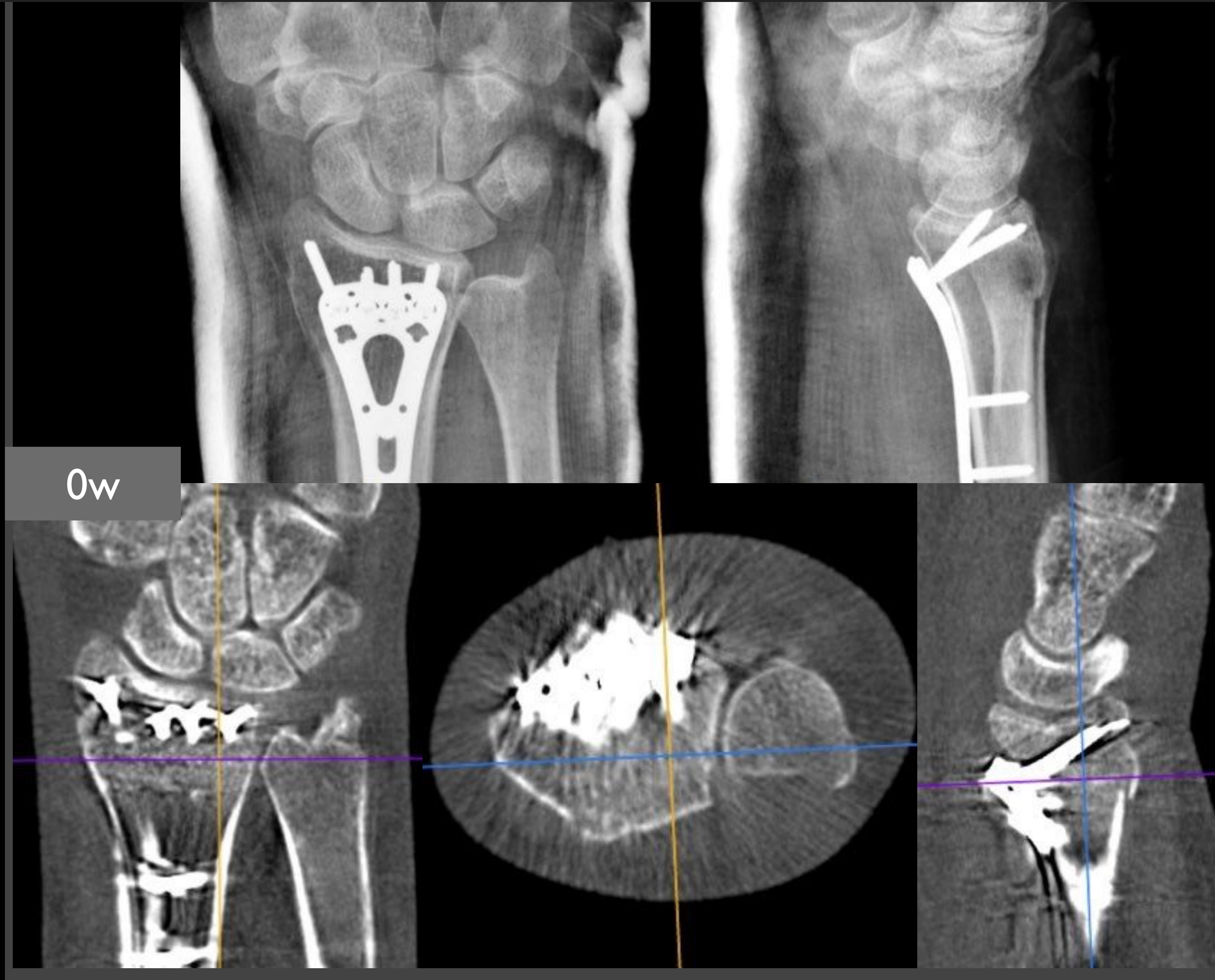

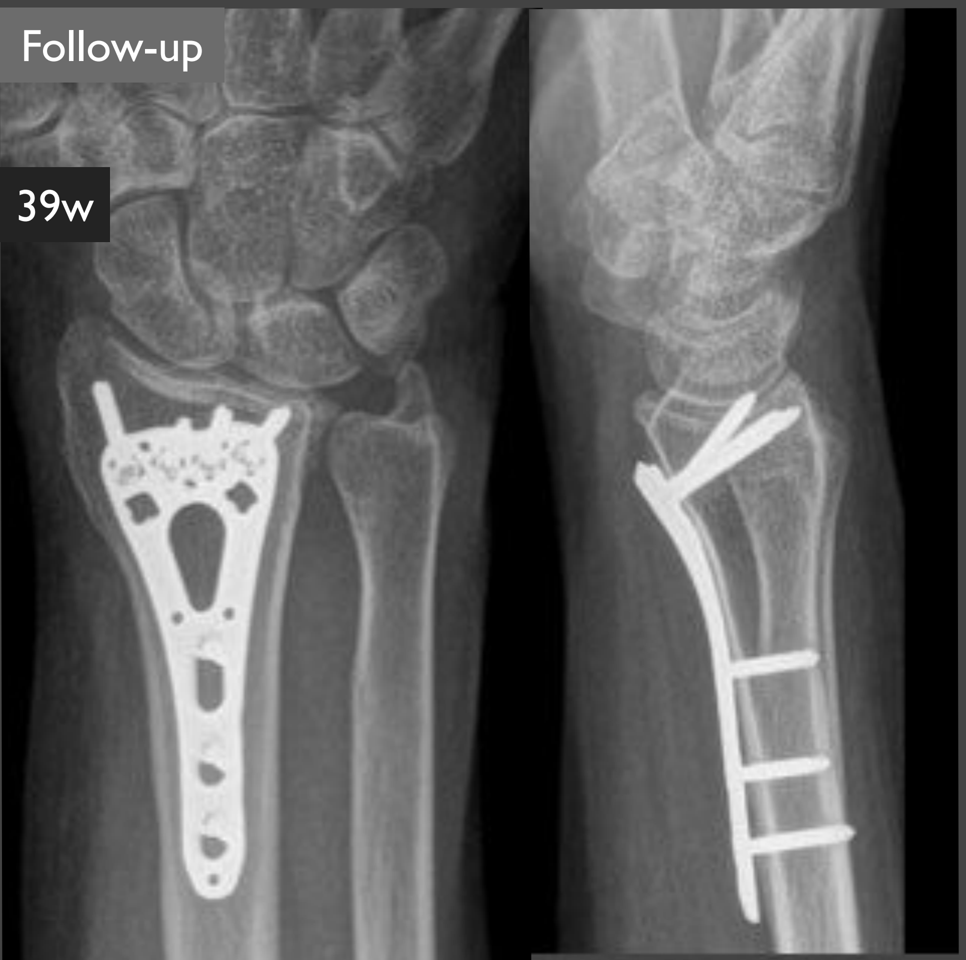

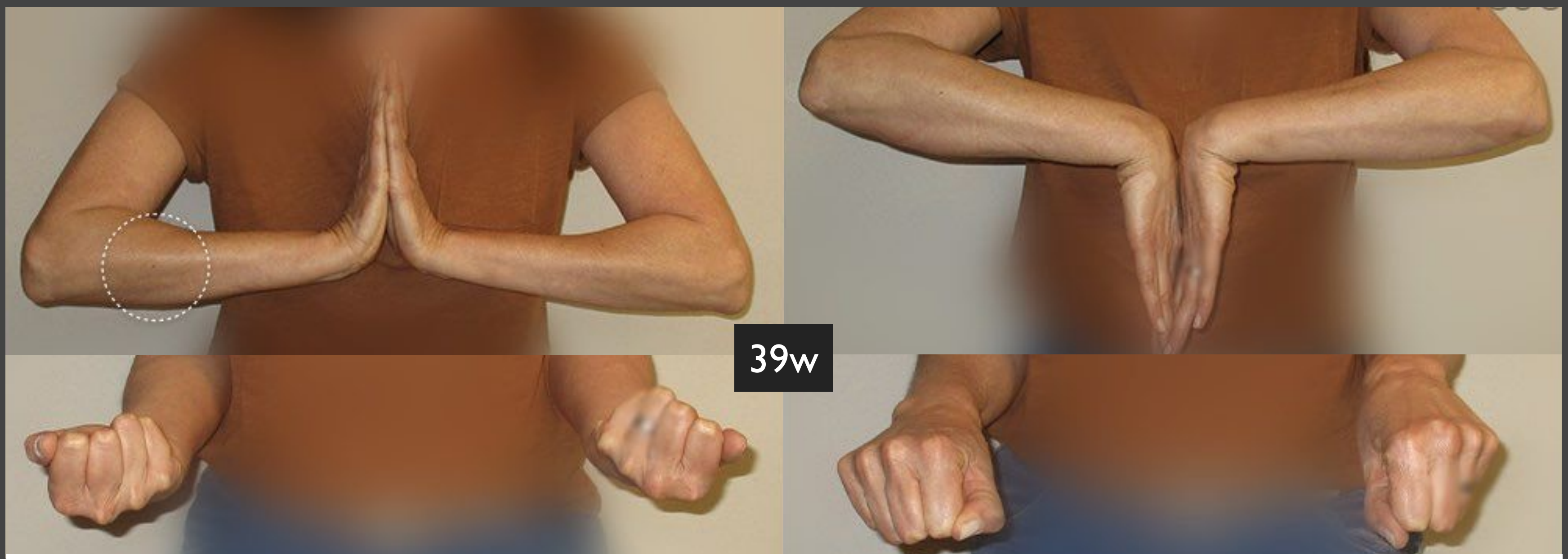

ICUC Score ***Functional Limitation:*** **1** (0-4) - ***Pain:*** **1** (0-4)

Quick DASH = 6.8

ICUC® ID: 23-DC-507 / 70y Male

Complete case data can be accessed by clicking the ***ICUC ID***

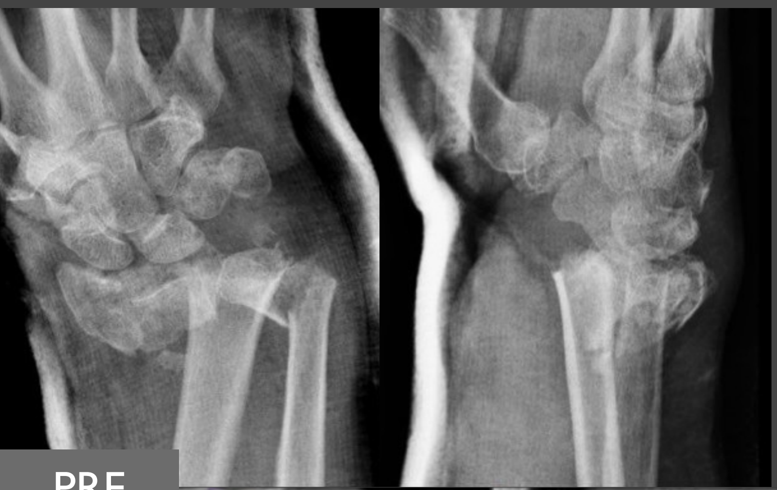

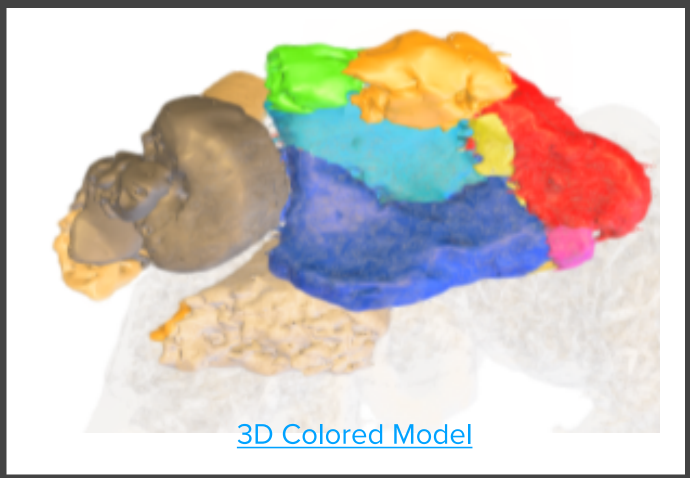

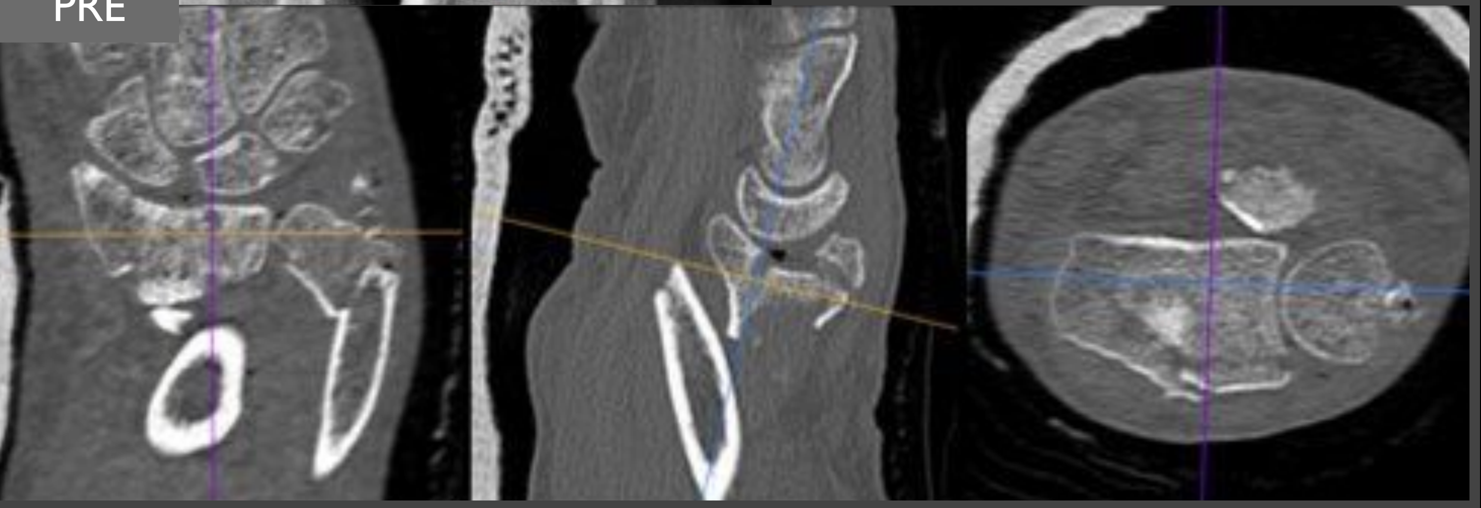

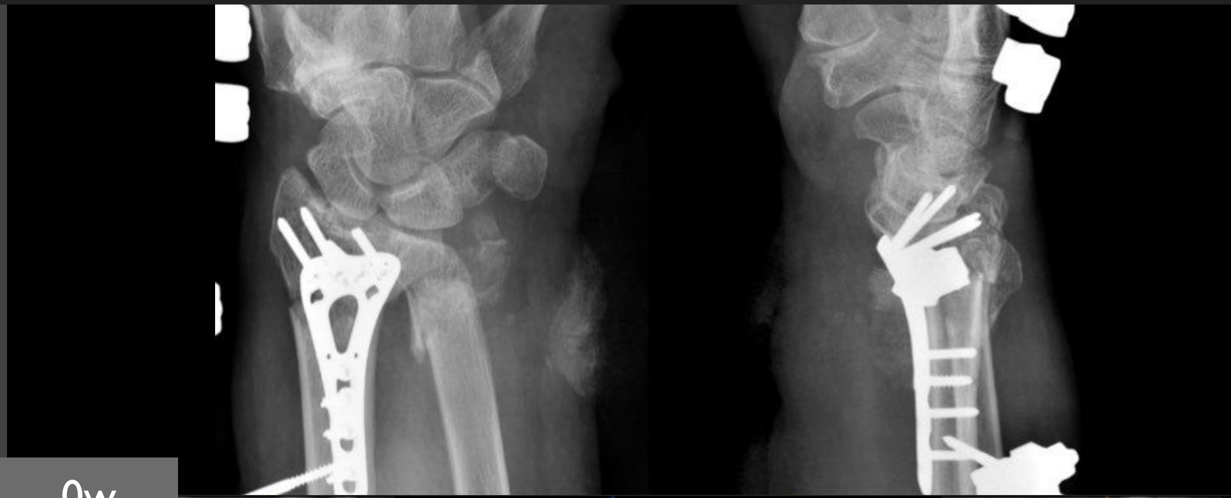

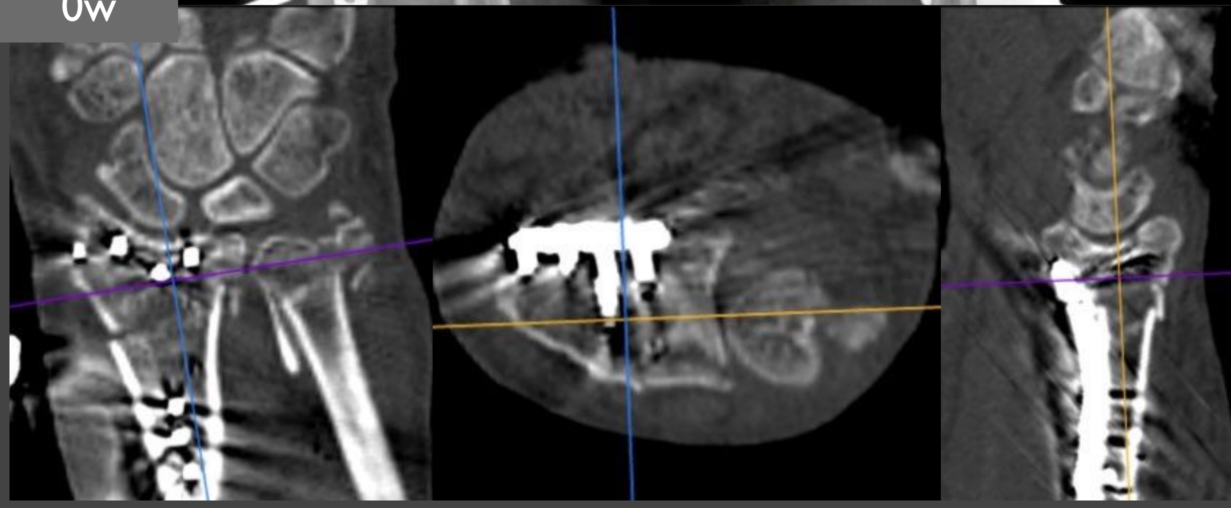

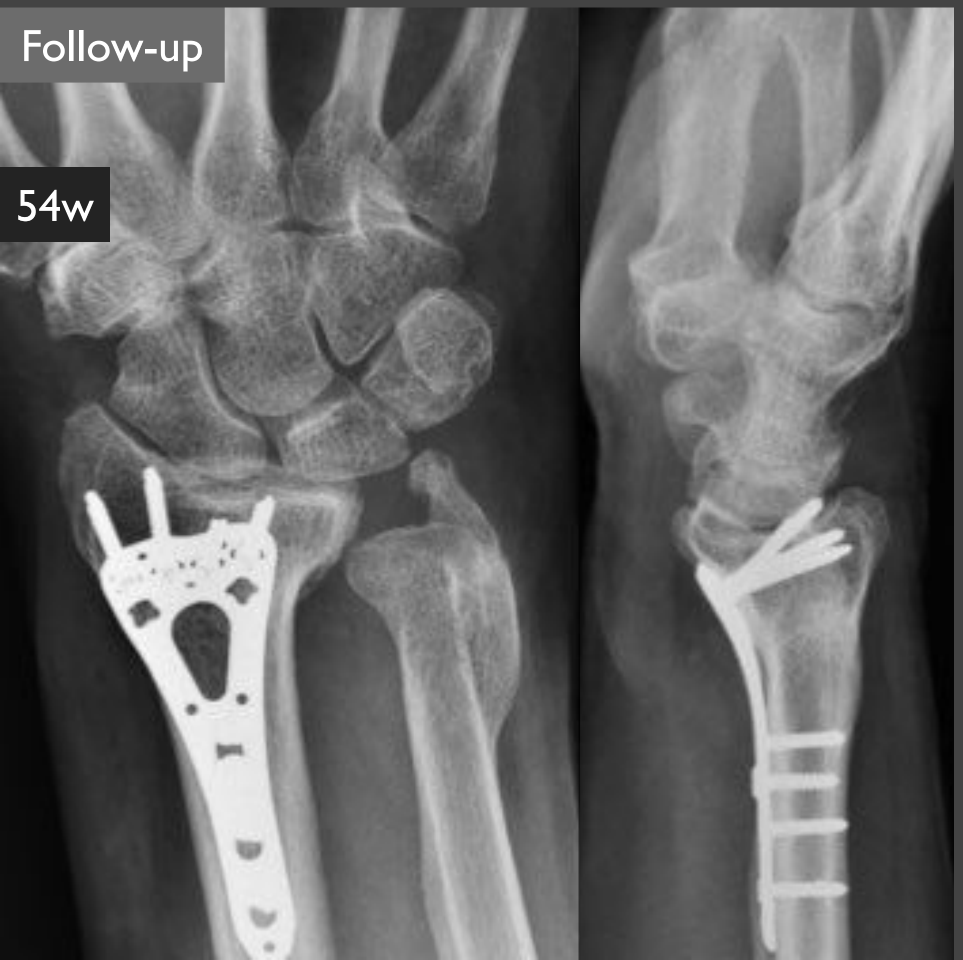

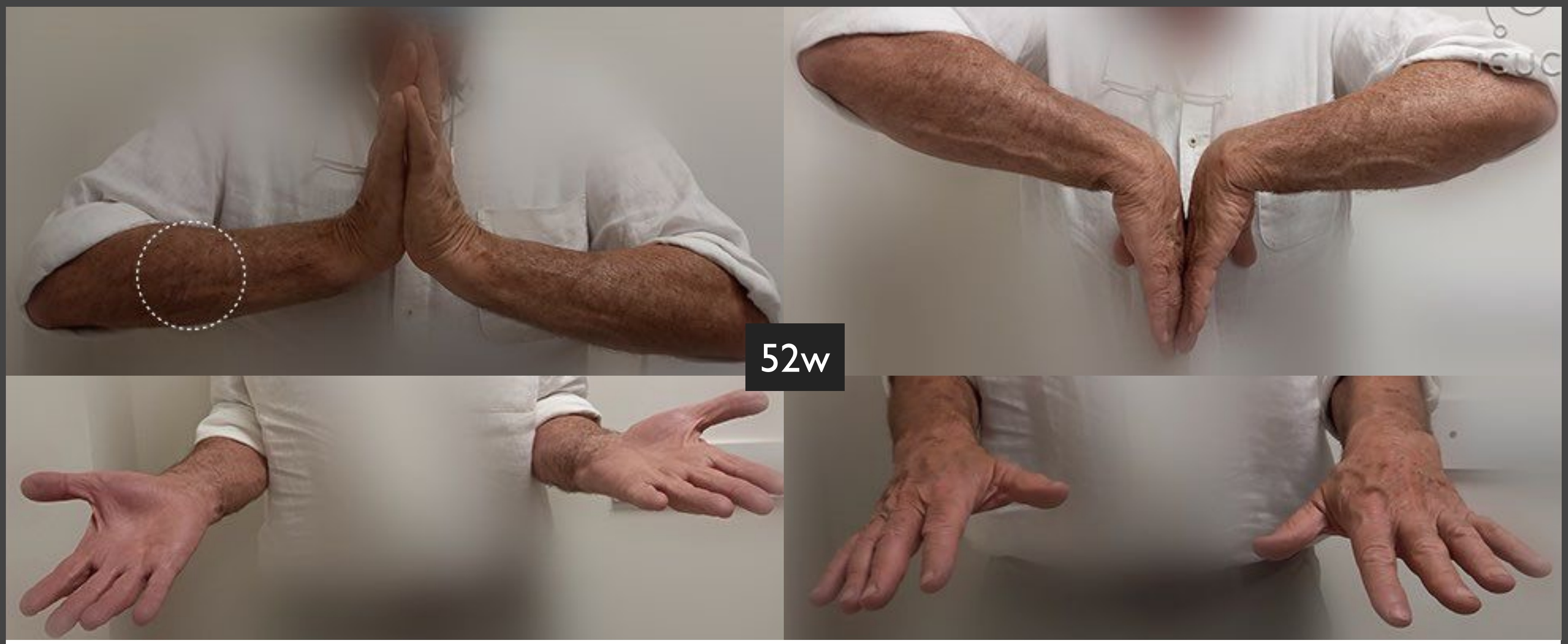

ICUC Score **Functional Limitation: 1** (0-4) - **Pain: 1** (0-4)

Quick DASH = 4,5

ICUC® ID: 23-VC-977 / 20y Male

*Complete case data can be accessed by clicking the **ICUC ID***

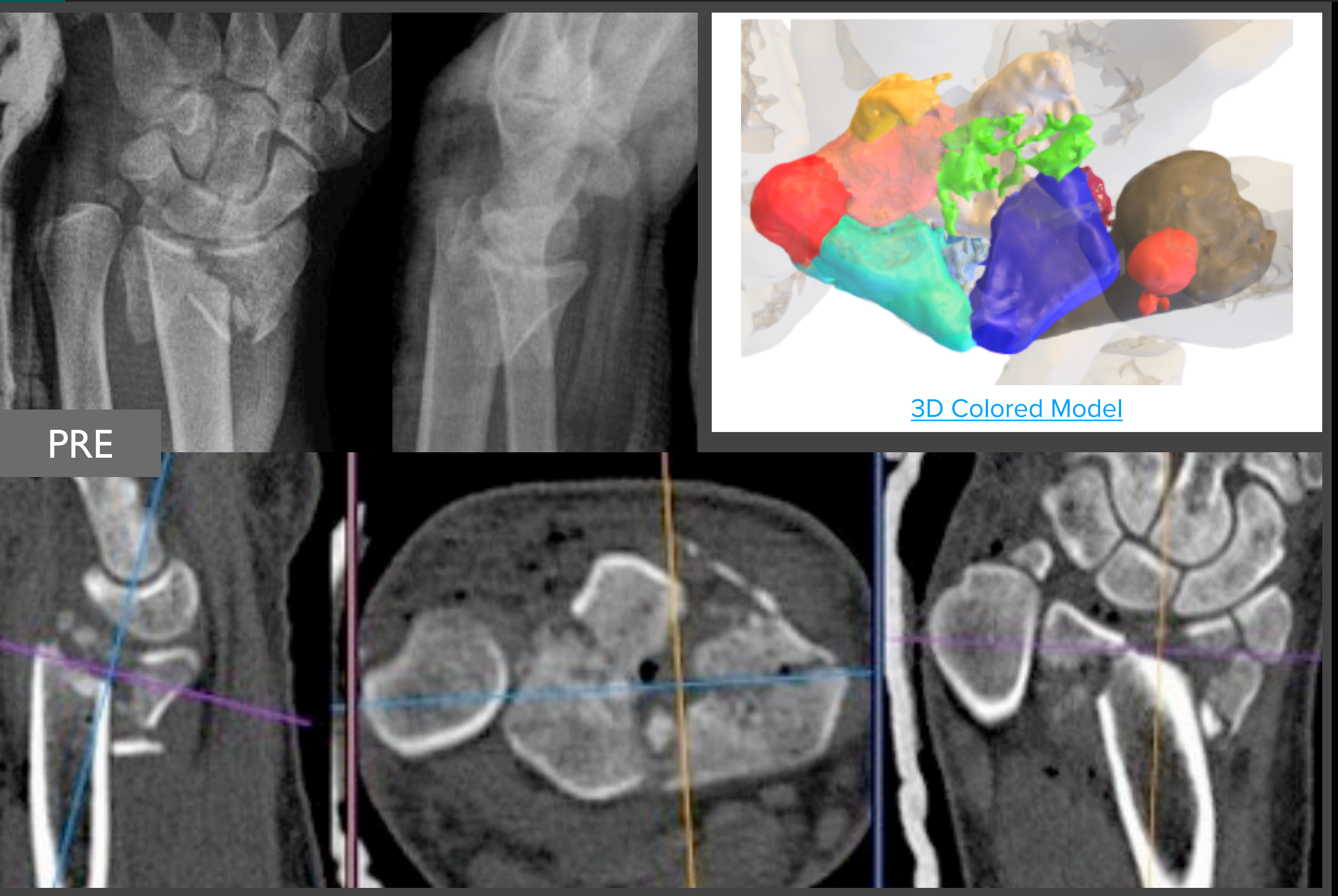

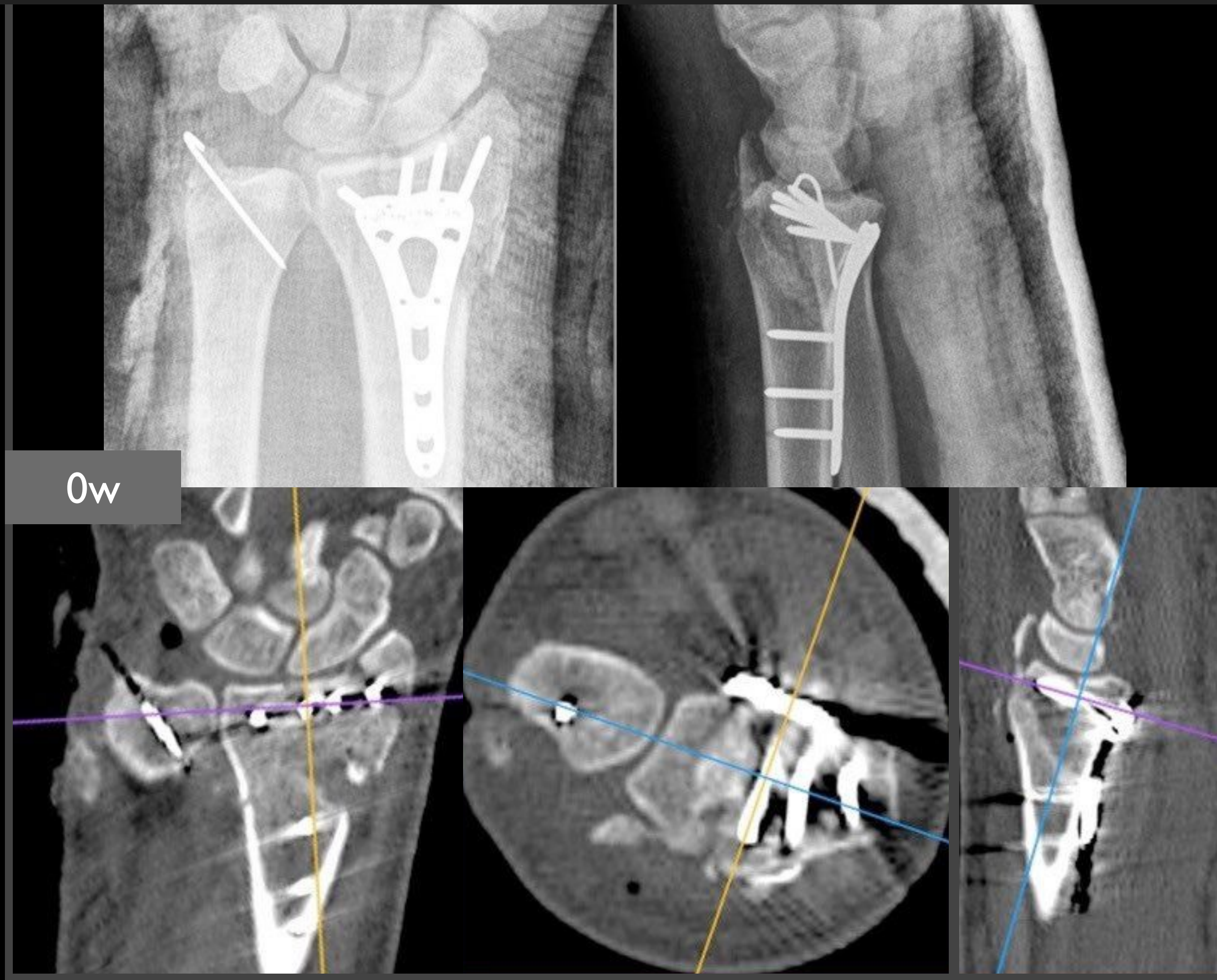

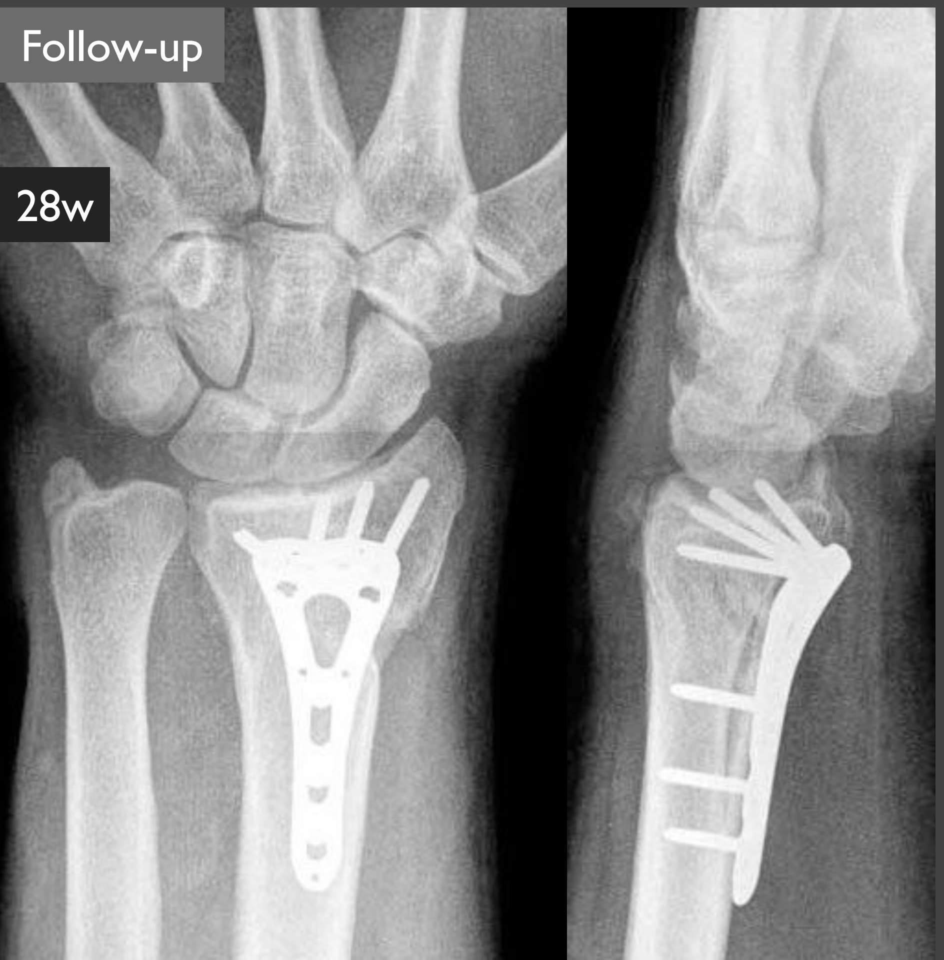

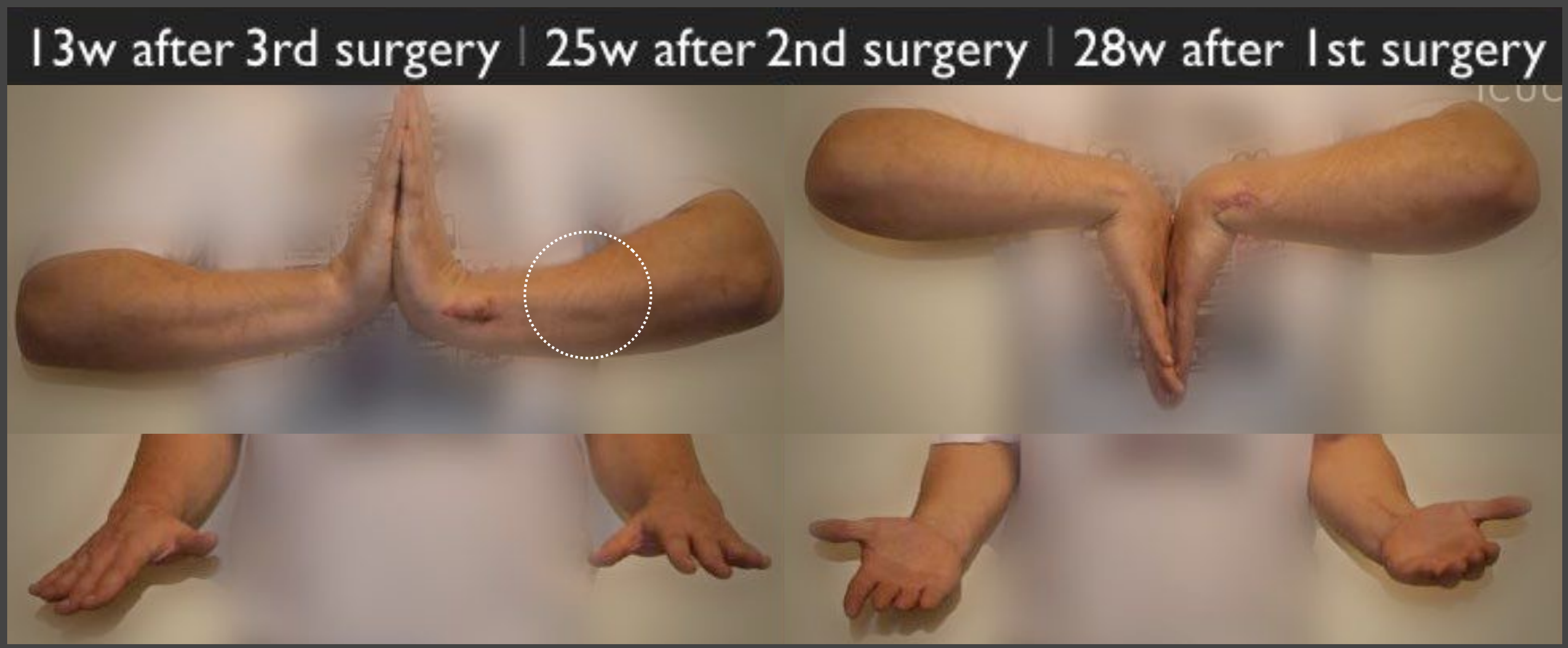

ICUC Score at 330w Functional limitation: 0 Pain: 0

Quick DASH = 0

ICUC® ID: 23-VC-635 / 55y Female

Complete case data can be accessed by clicking the ***ICUC ID***

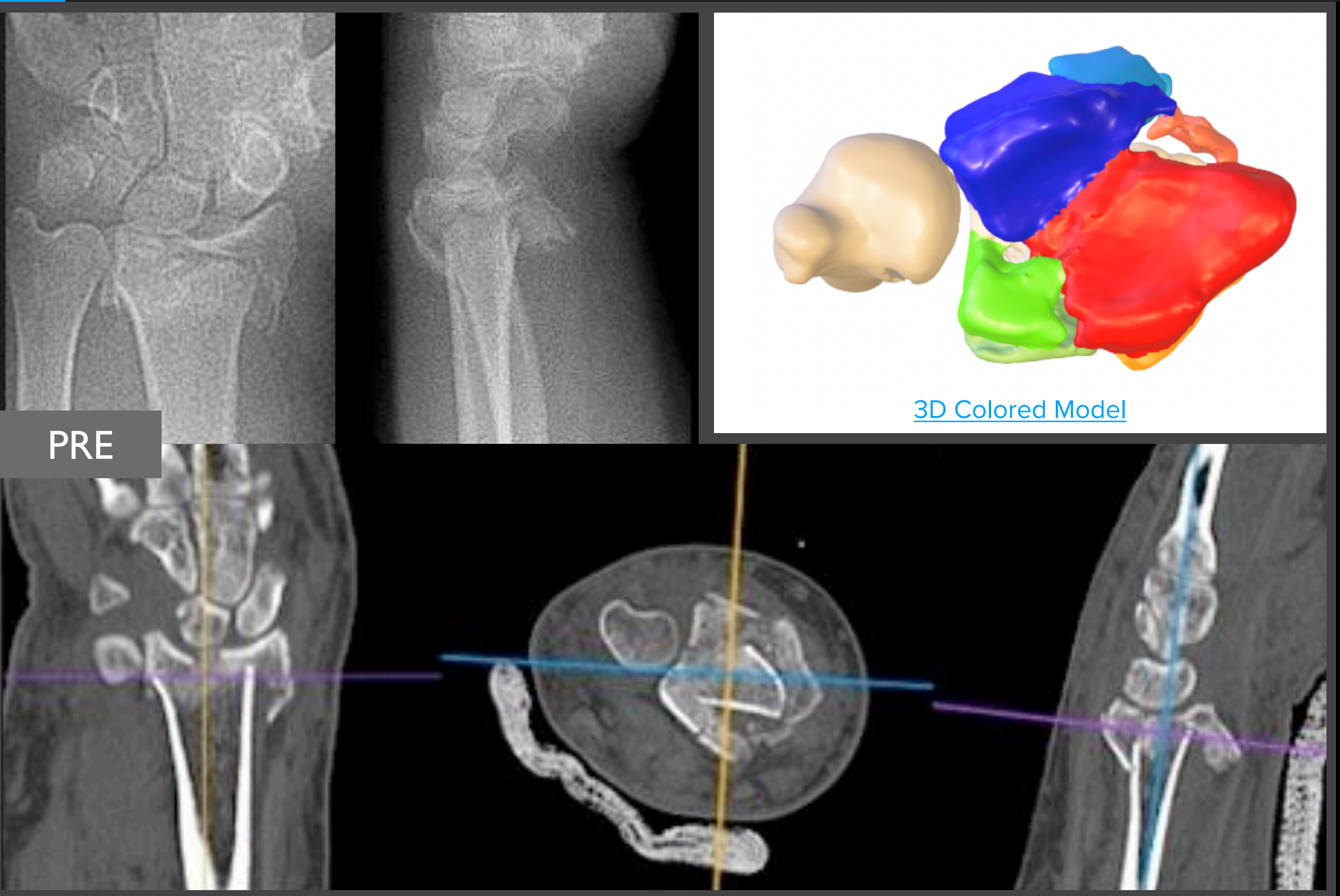

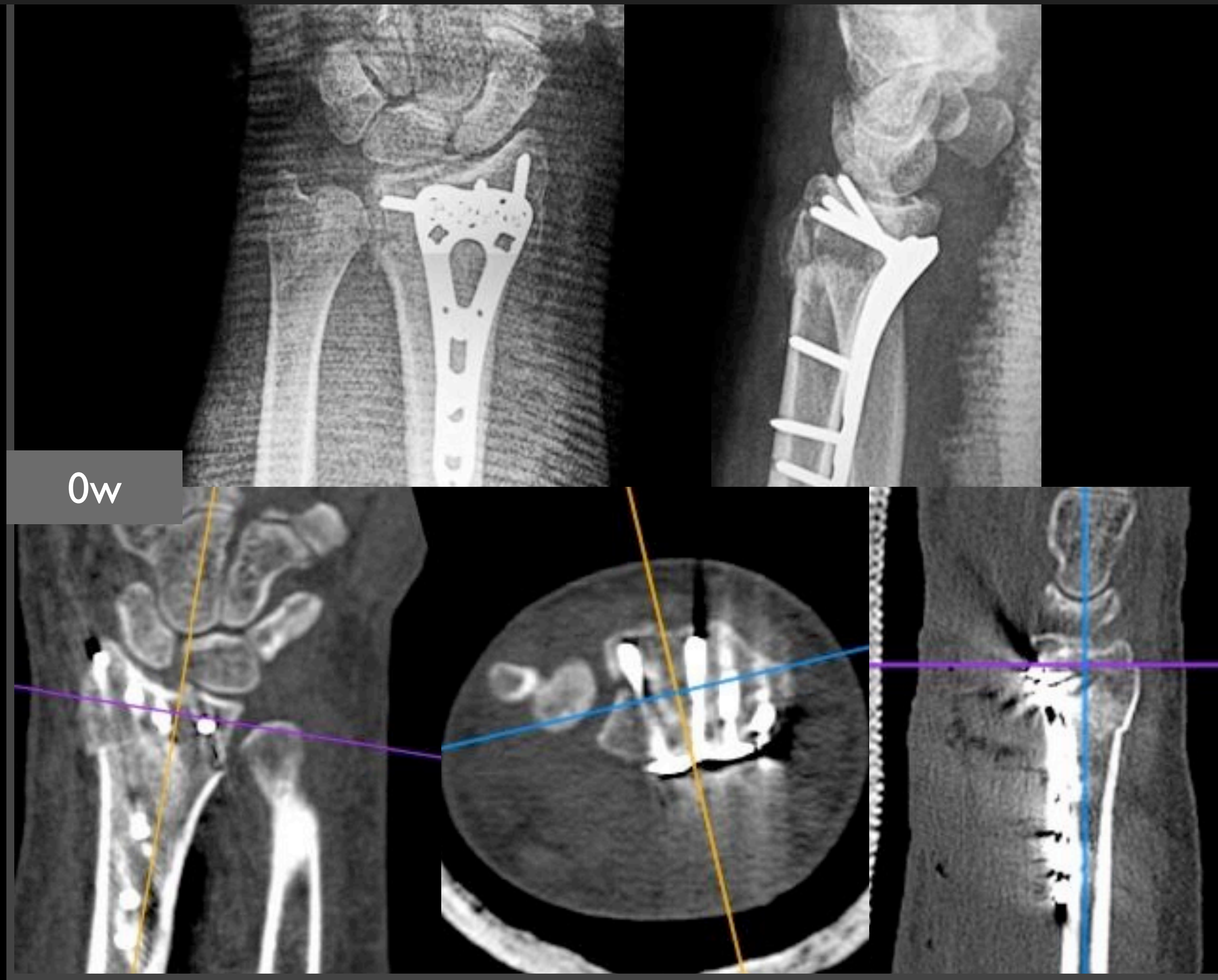

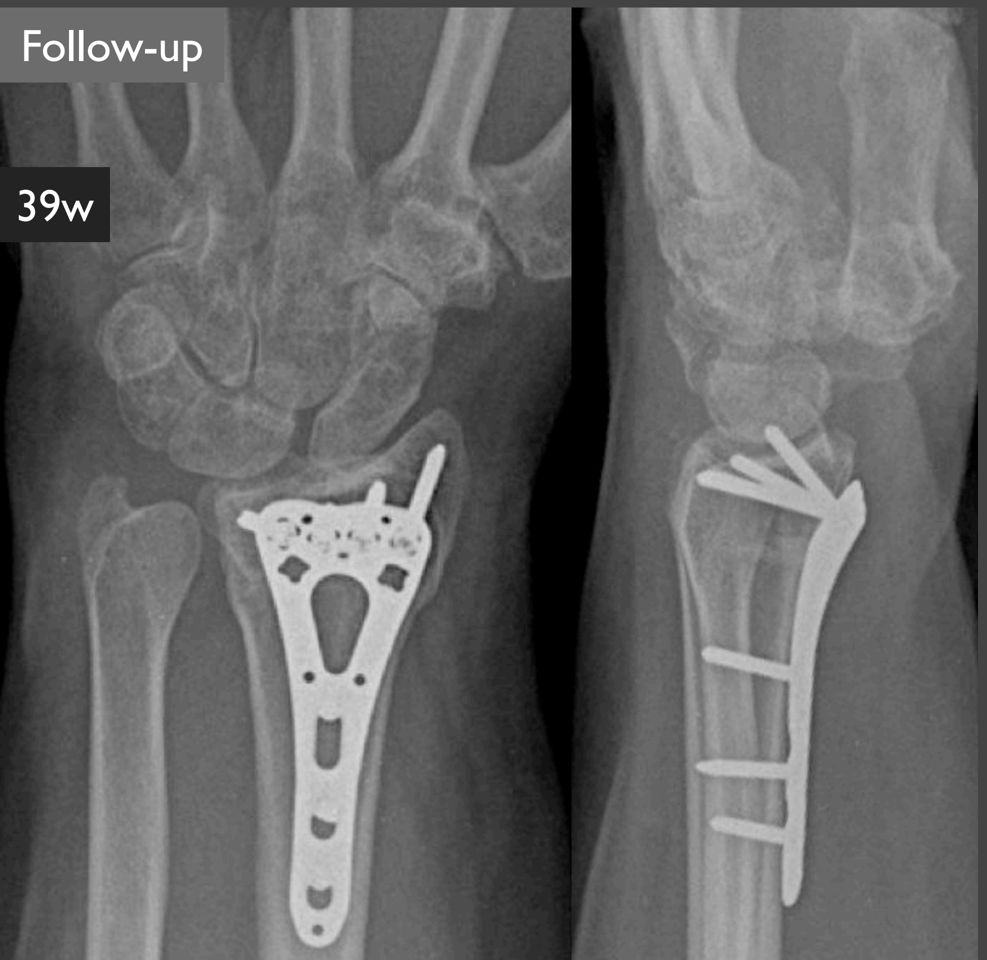

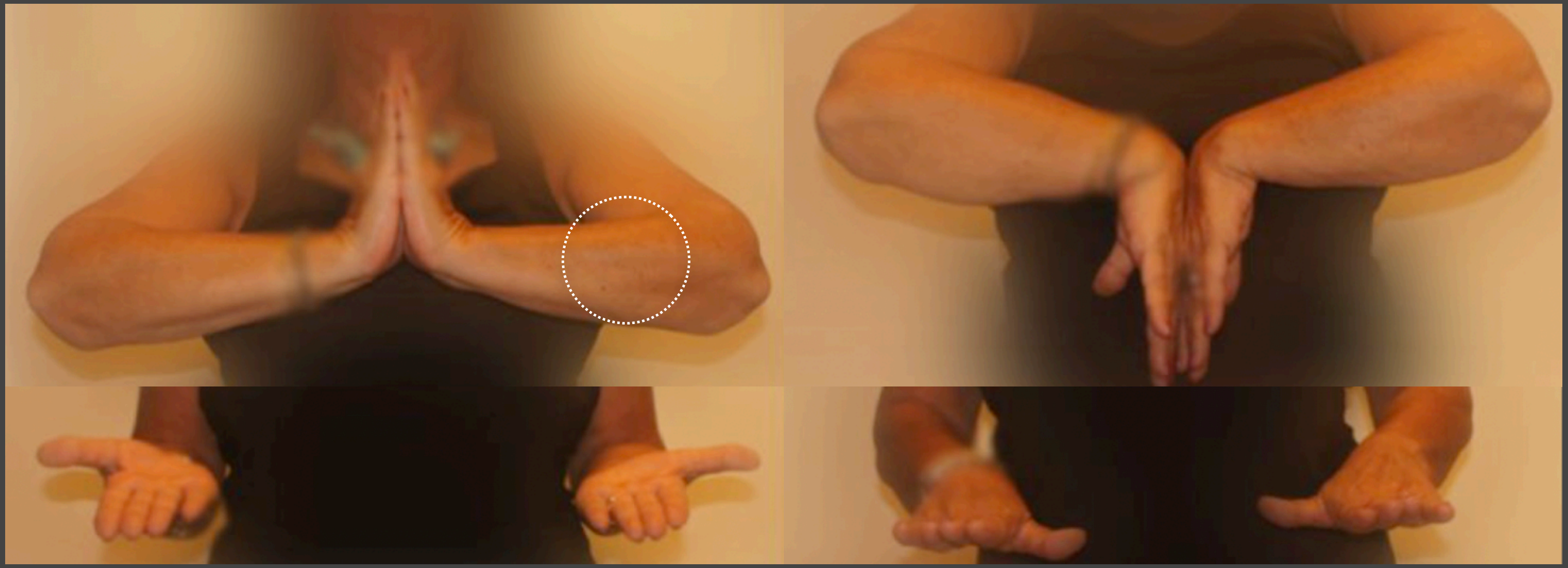

ICUC Score at 288w Functional limitation: 0 Pain: 0

Quick DASH = 0

ICUC® ID: 23-VC-022 / 50y Female

Complete case data can be accessed by clicking the ***ICUC ID***

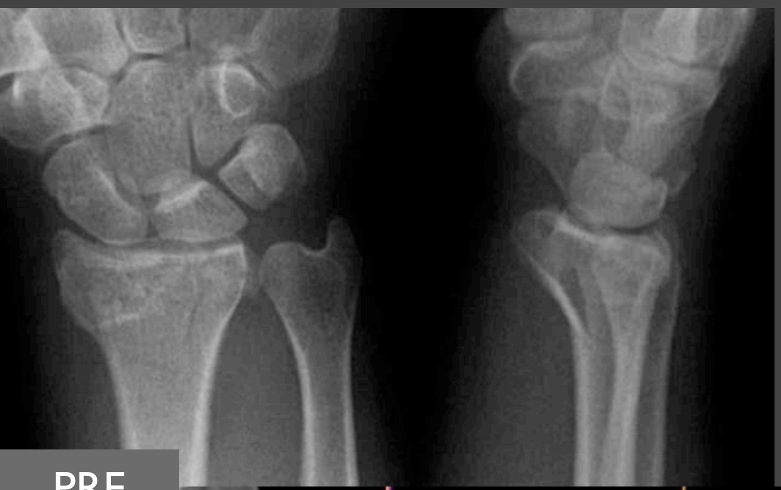

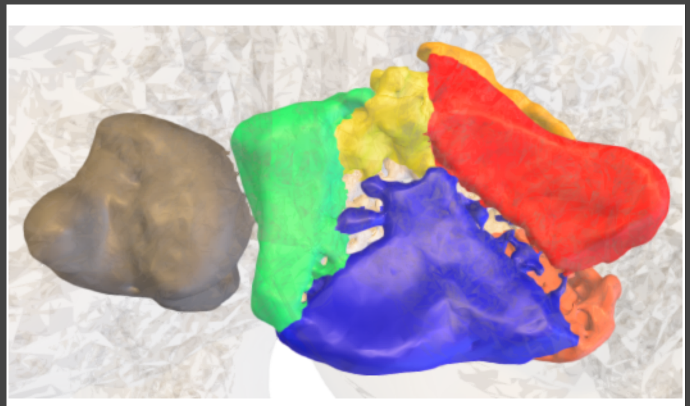

3D Colored Model

PRE

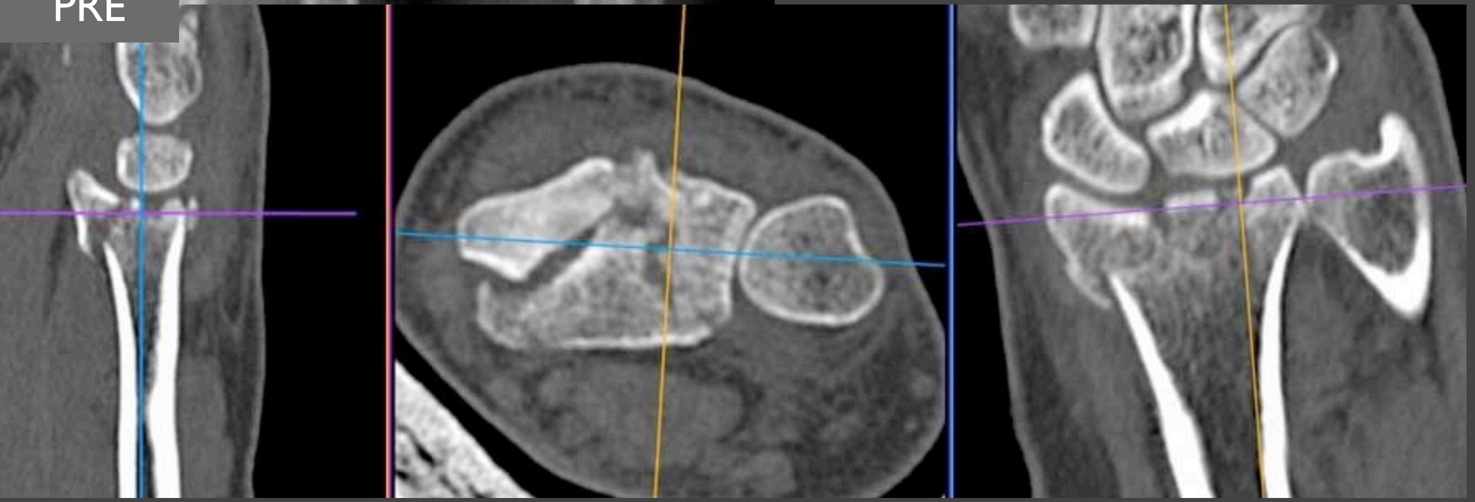

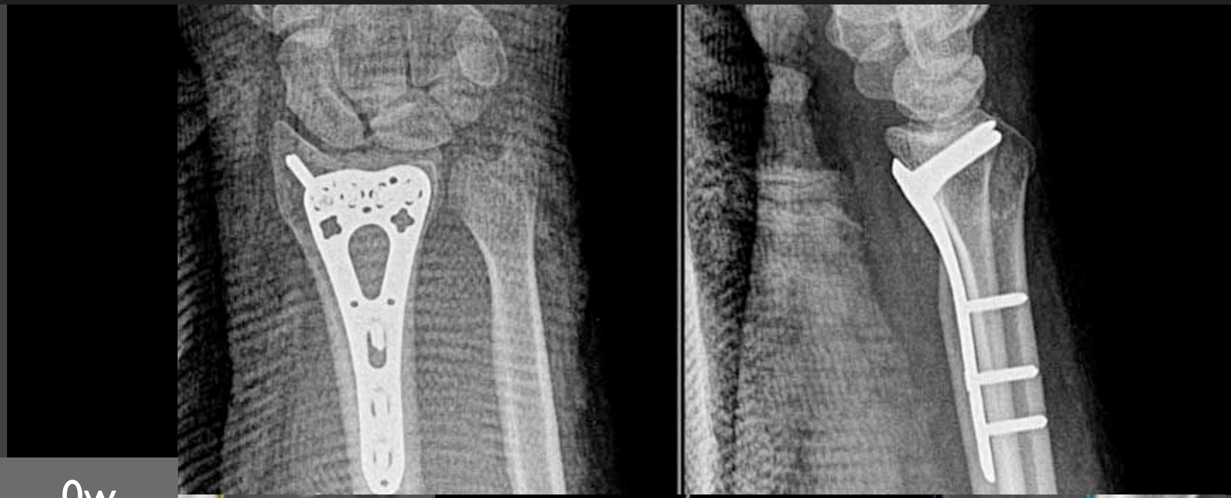

0w

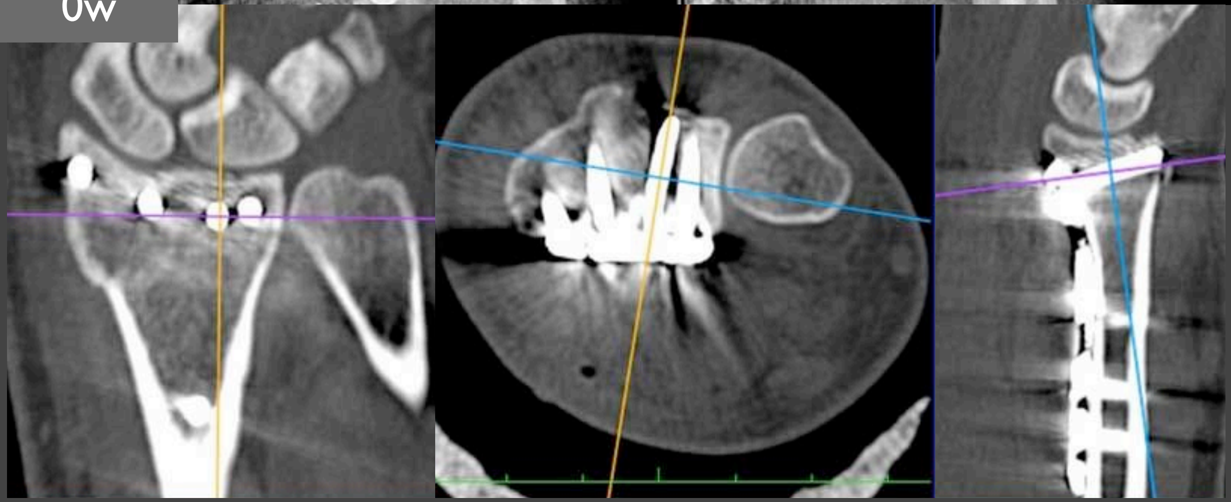

Follow-up

48w

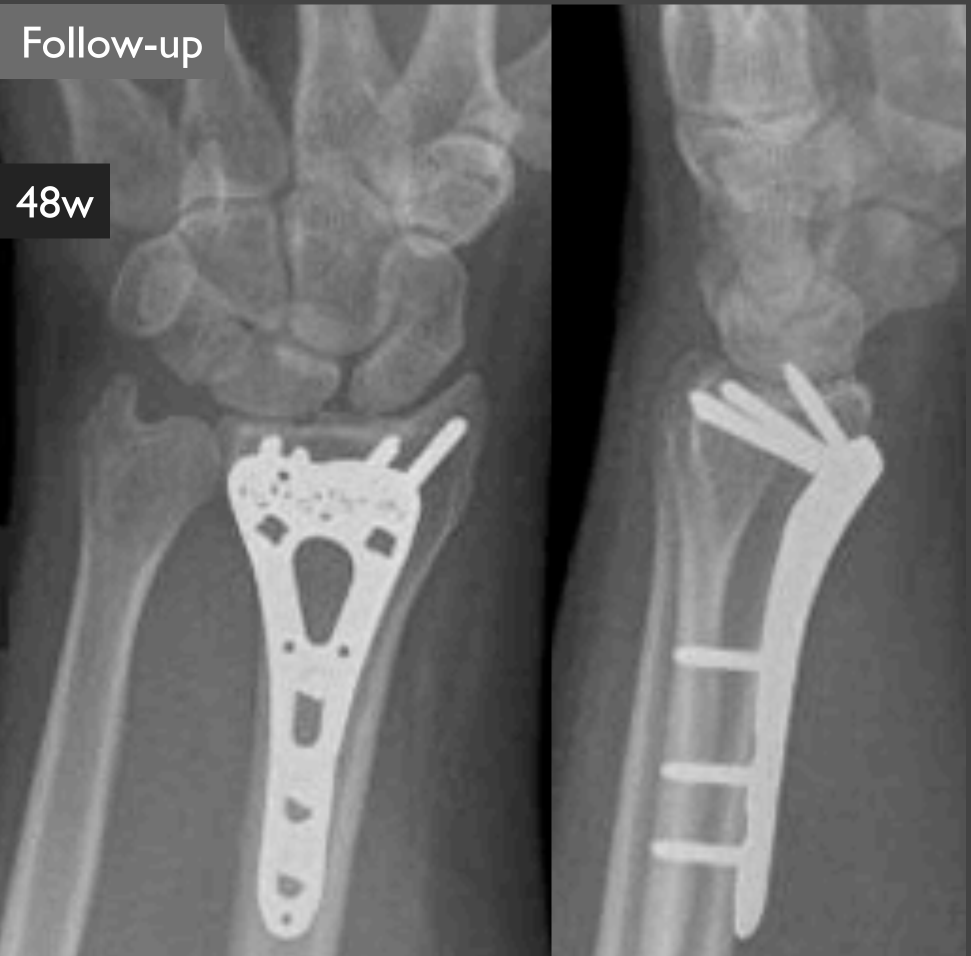

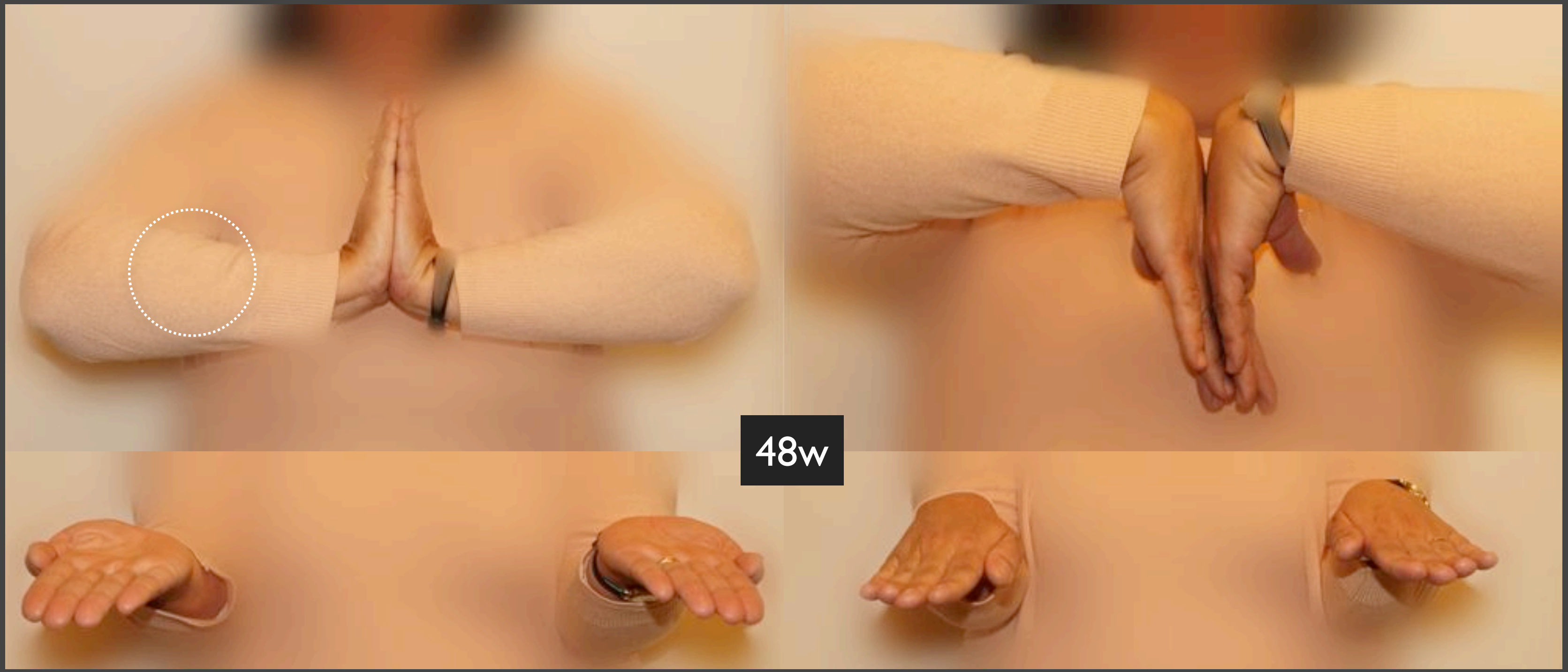

ICUC Score at 271w Functional limitation: 0 Pain: 0

Quick DASH = 0

ICUC® ID: 23-VC-189 / 60y Female

*Complete case data can be accessed by clicking the **ICUC ID***

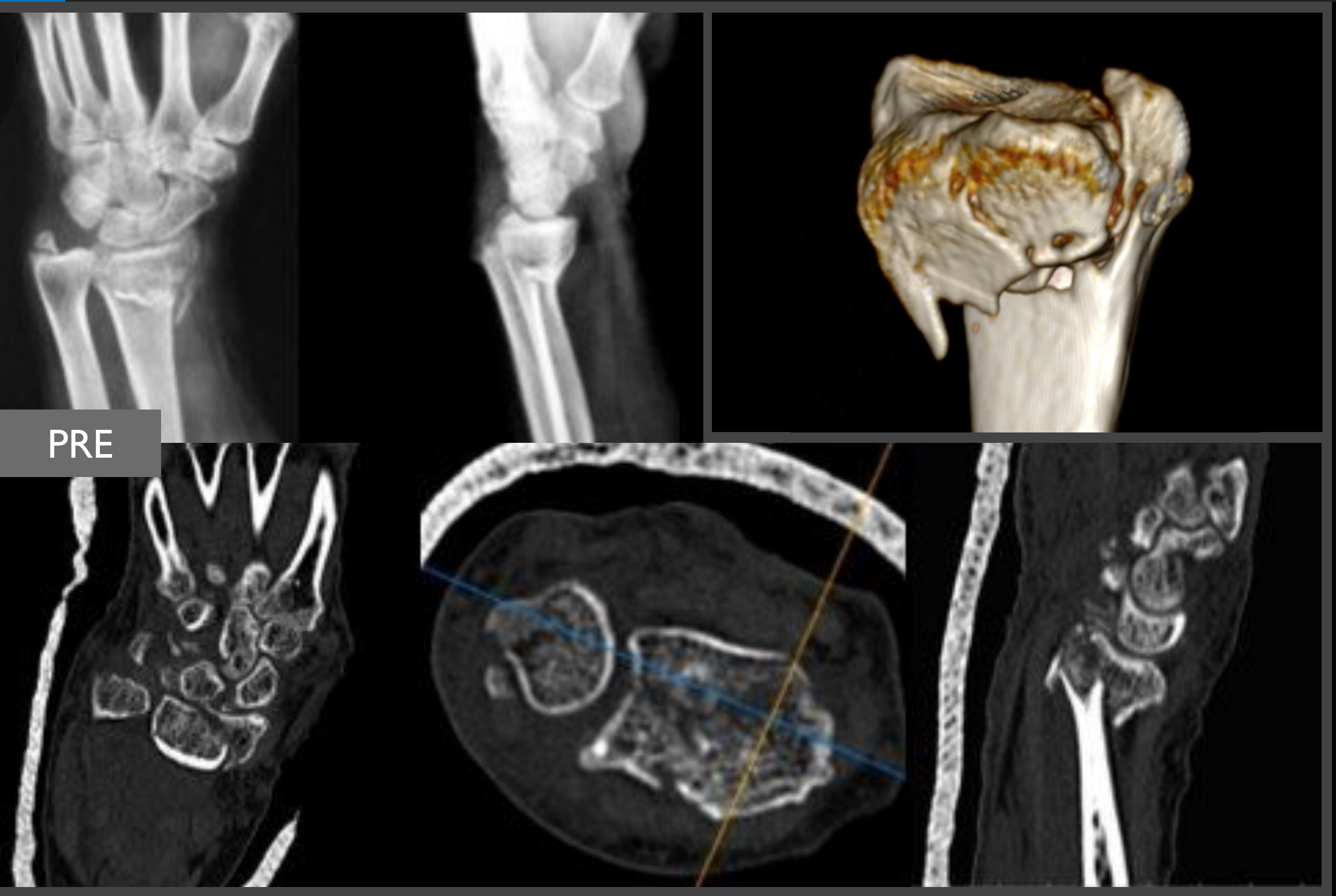

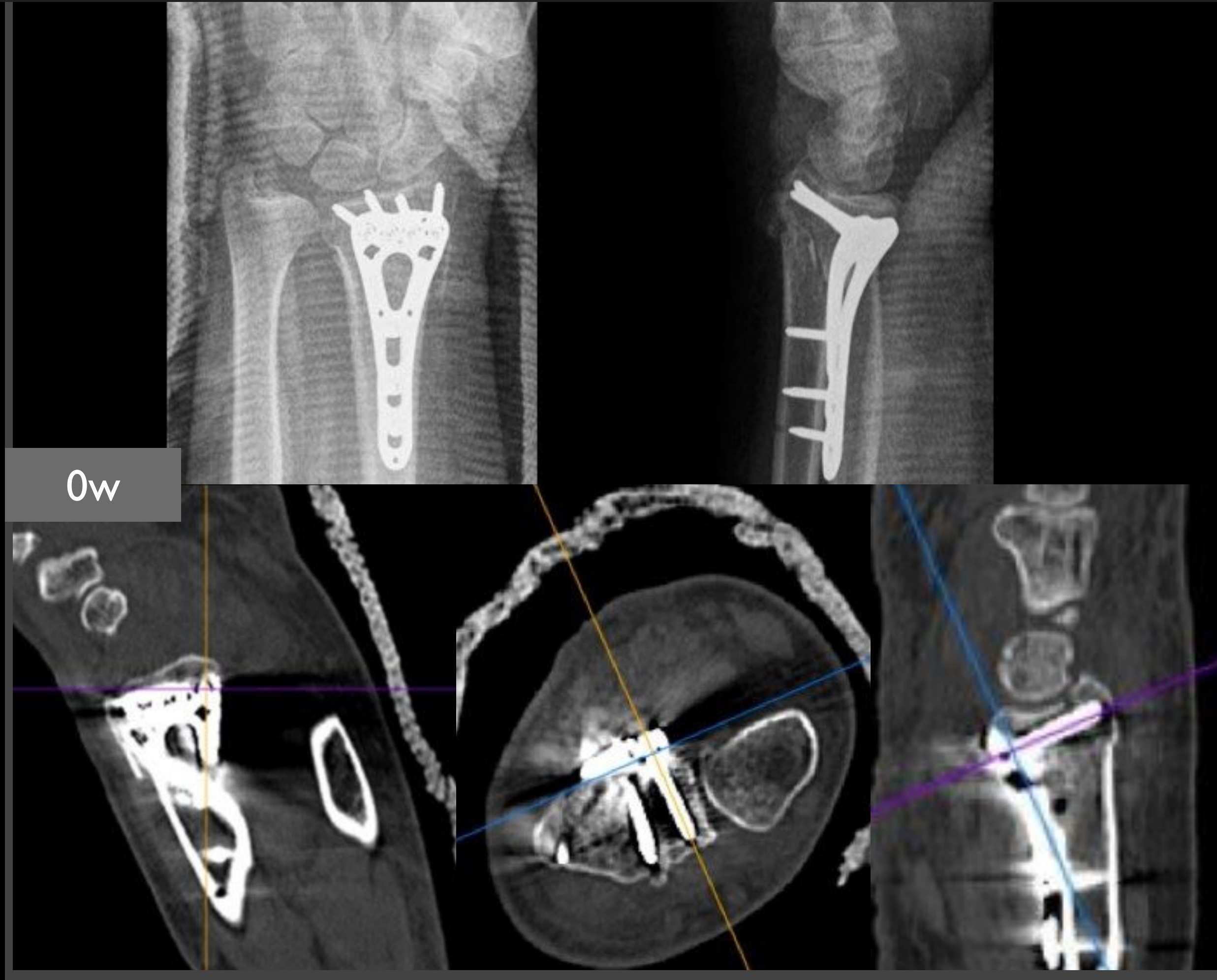

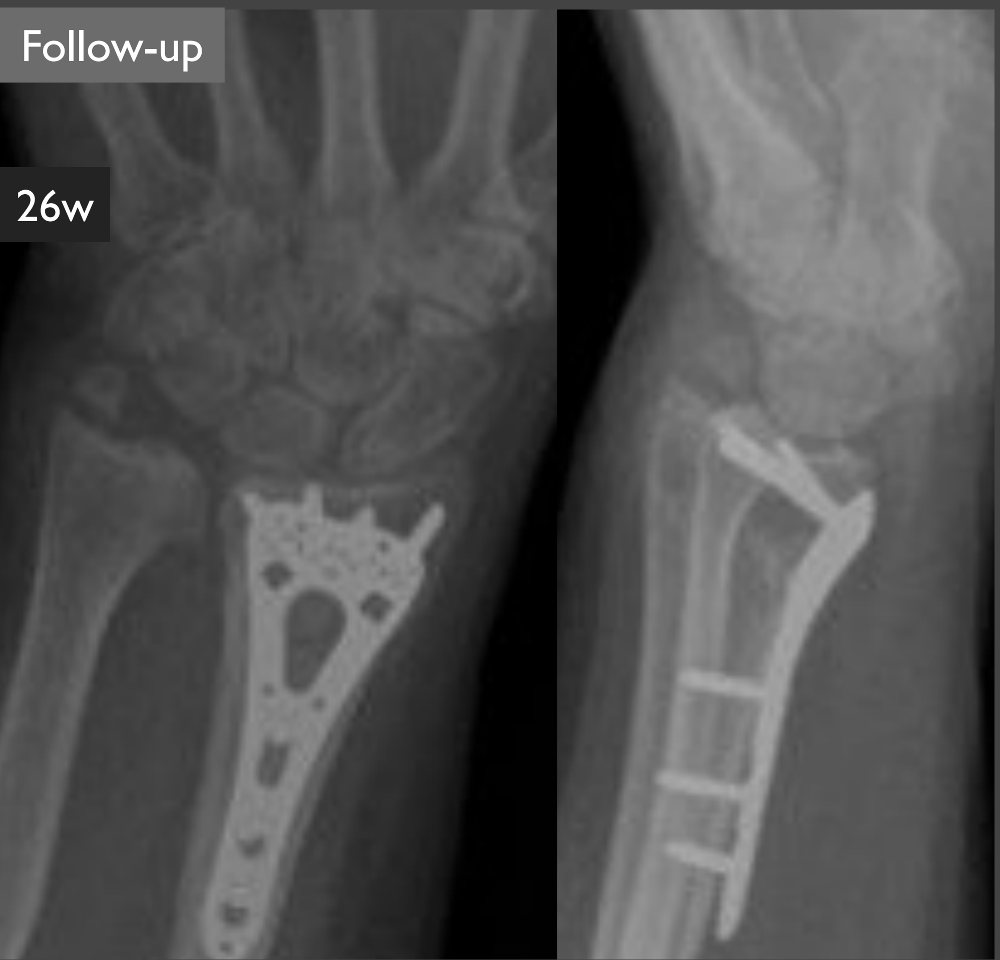

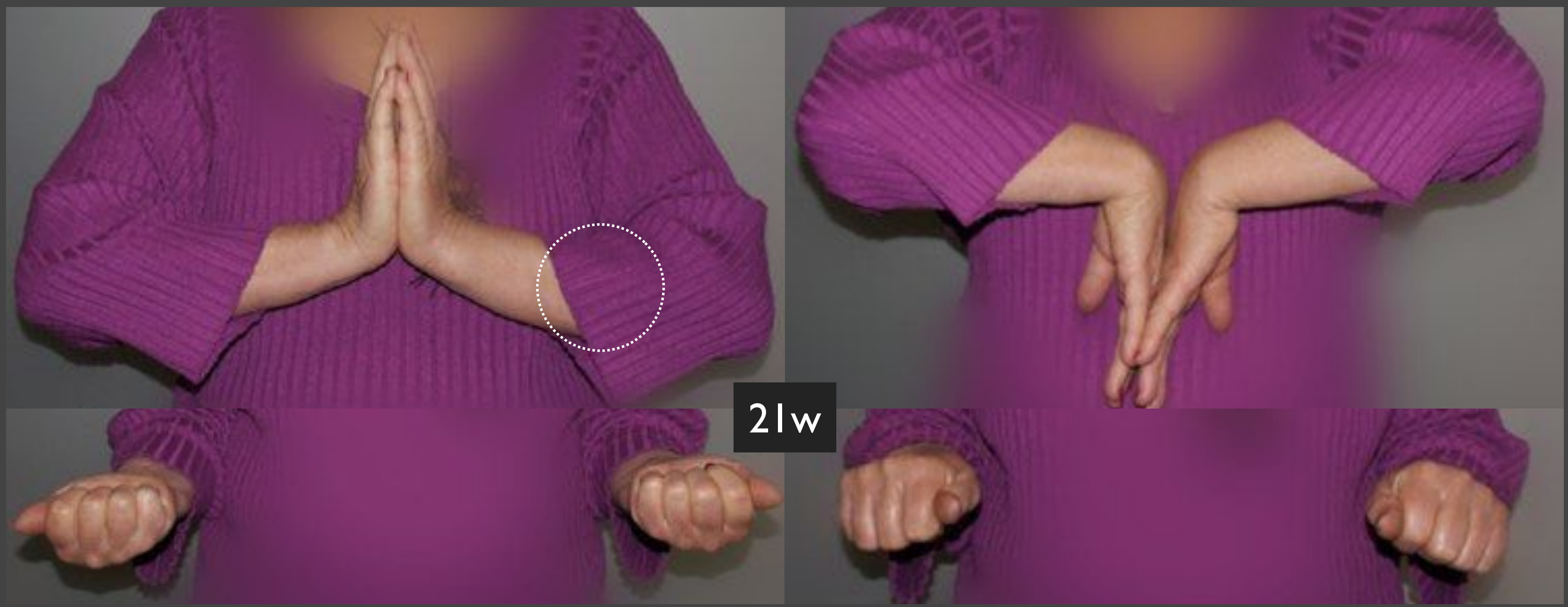

ICUC Score at 211w Functional limitation: 0 Pain: 1

Quick DASH = 0

ICUC® ID: 23-VC-705 / 70y Female

Complete case data can be accessed by clicking the ***ICUC ID***

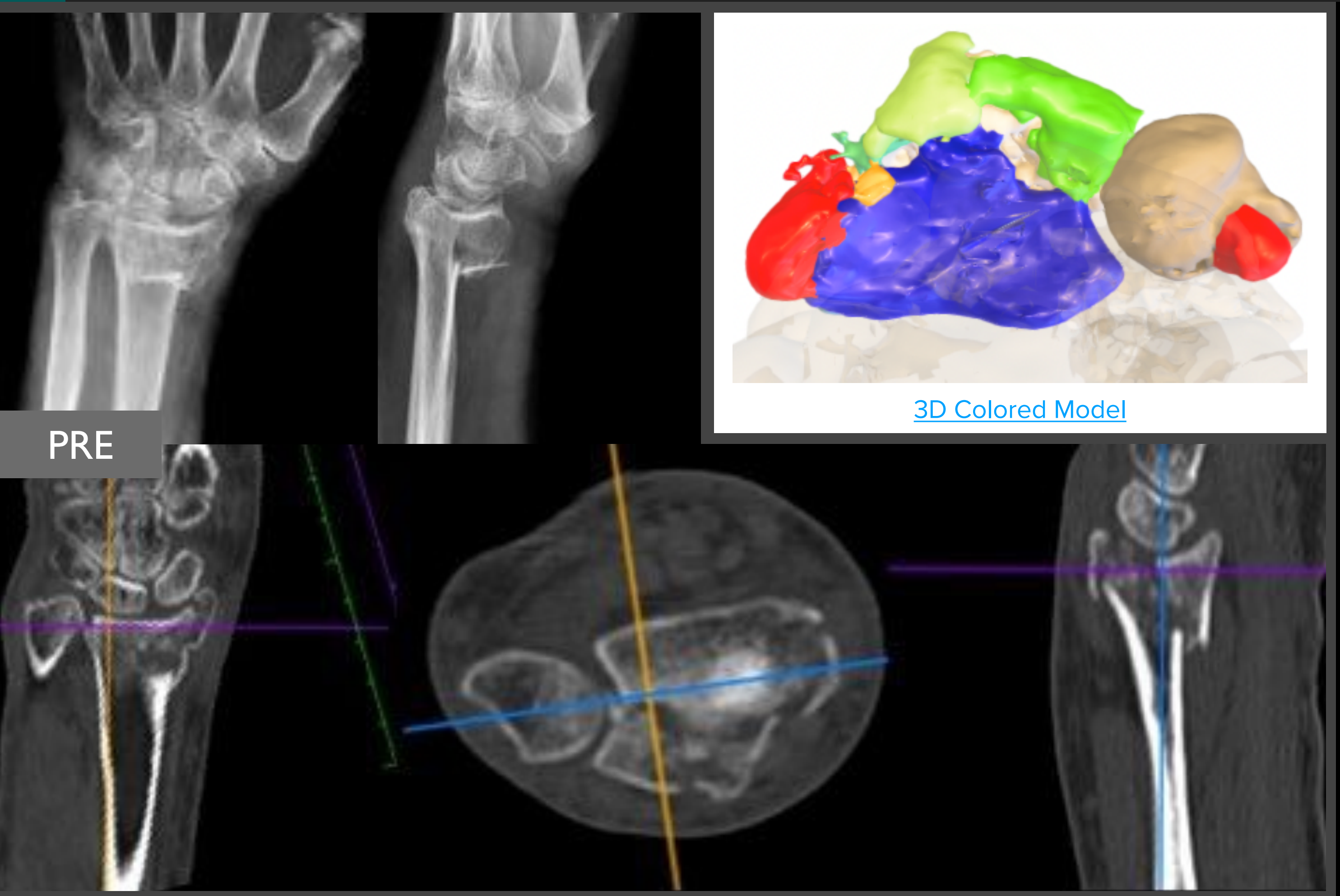

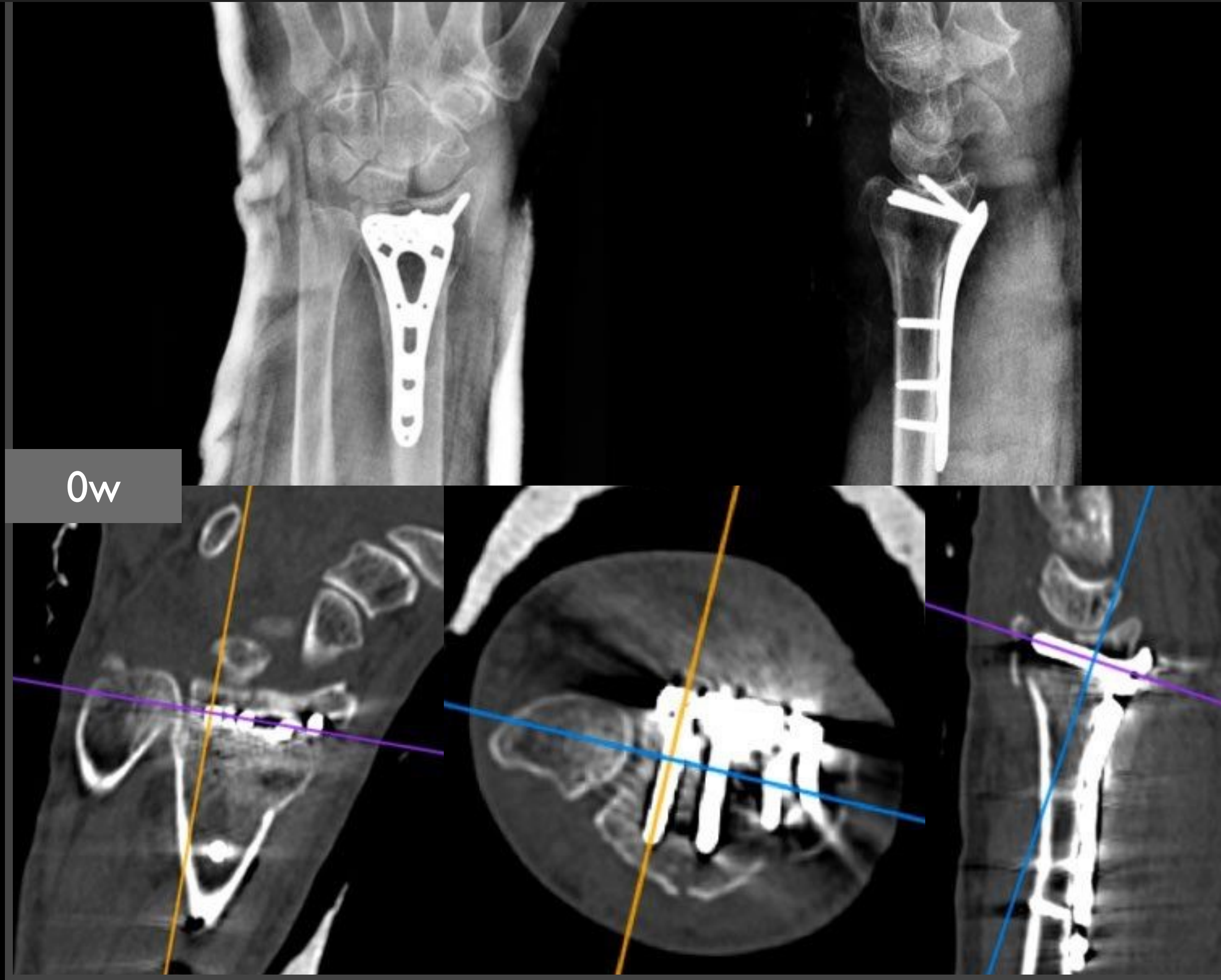

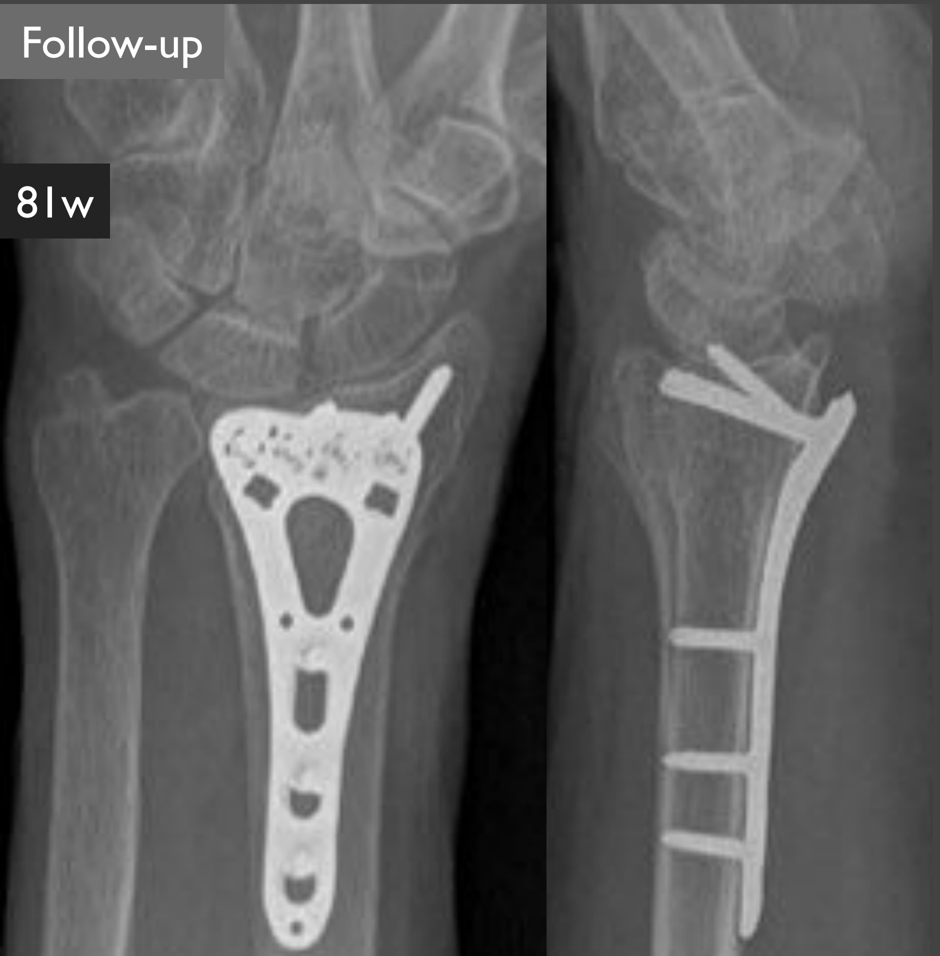

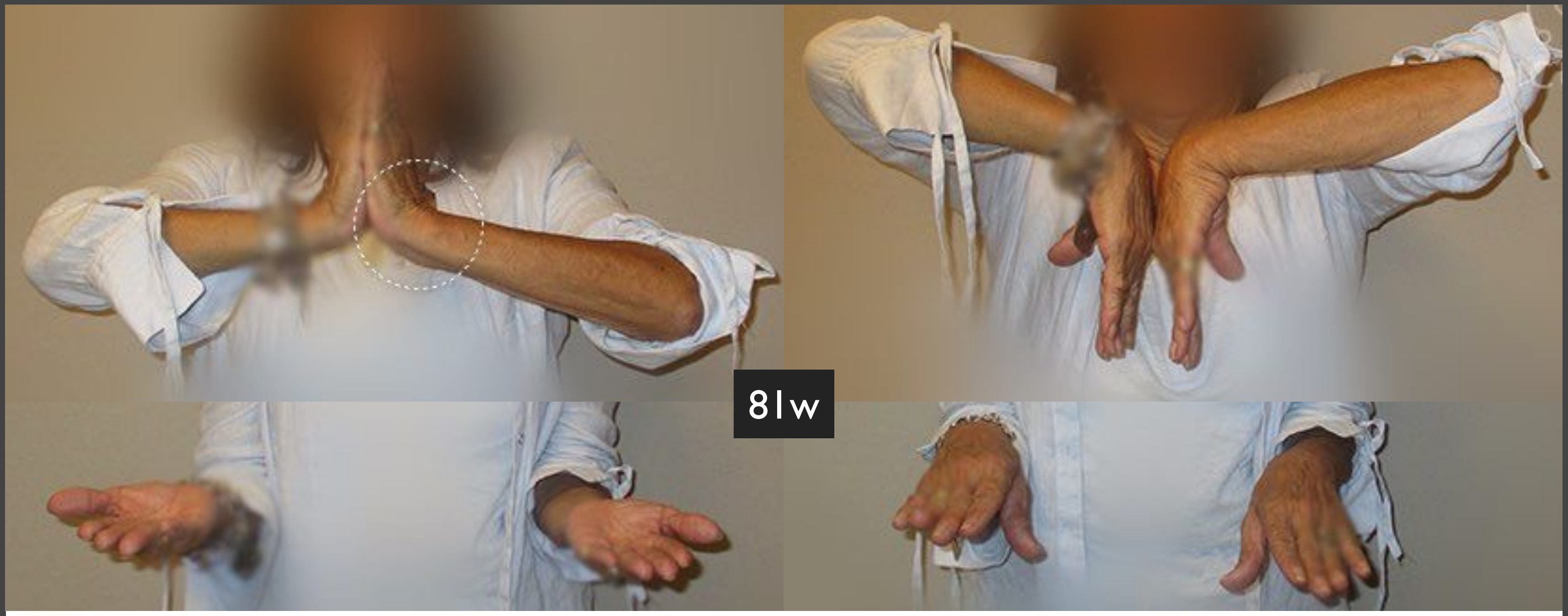

ICUC Score Functional Limitation: **0** (0-4) - Pain: **0** (0-4)

Quick DASH = 0

ICUC® ID: 23-VC-547 / 50y Male

*Complete case data can be accessed by clicking the **ICUC ID***

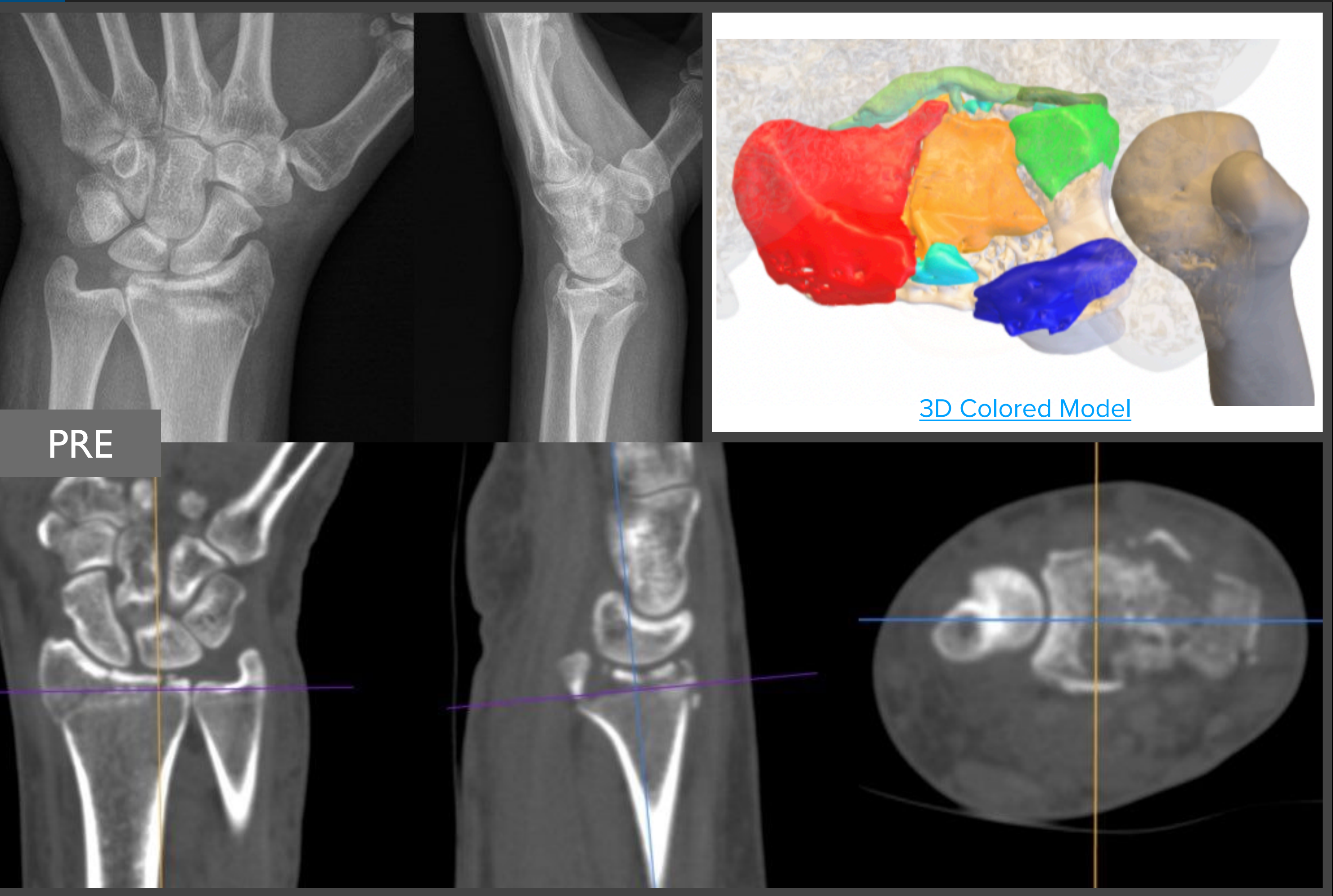

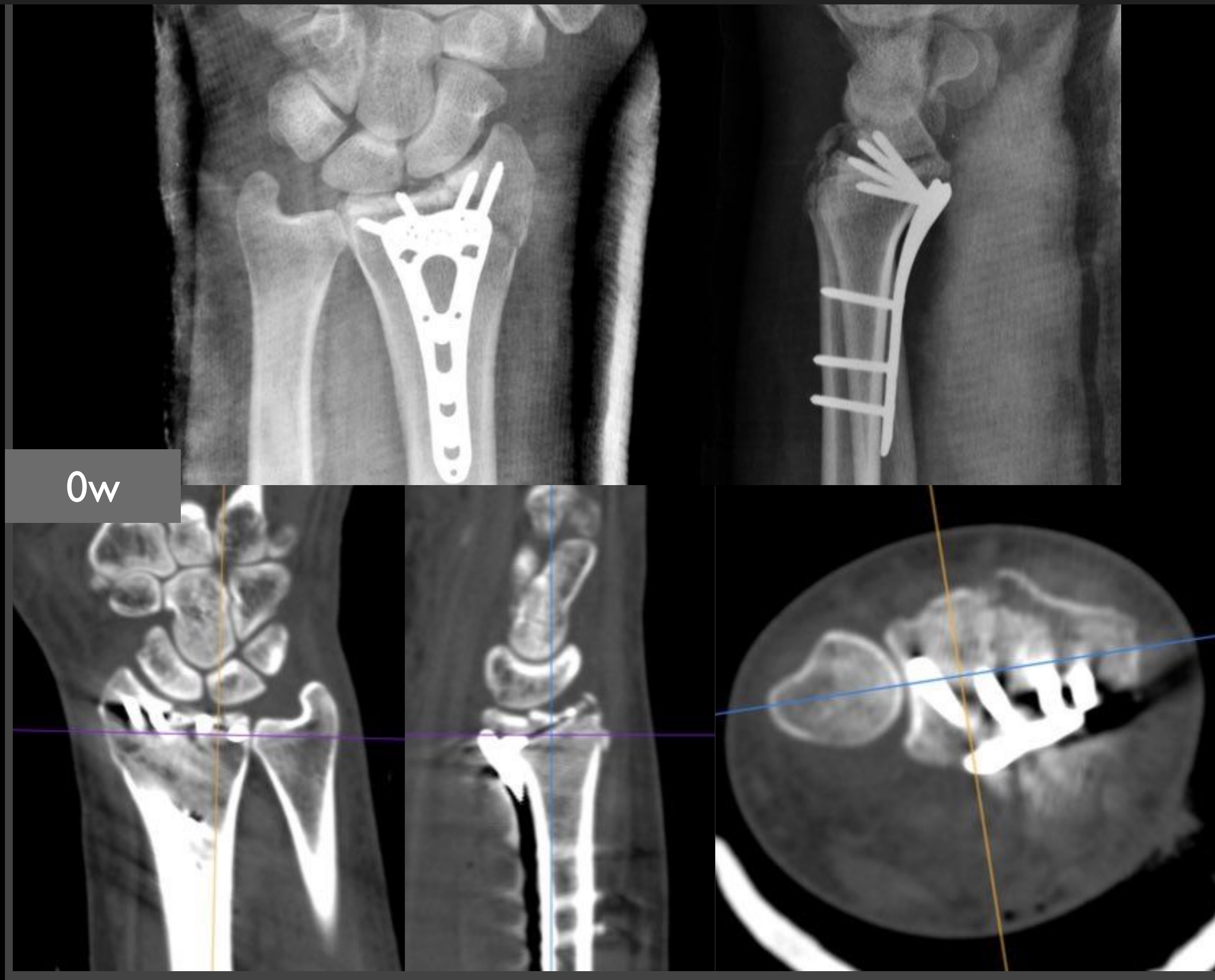

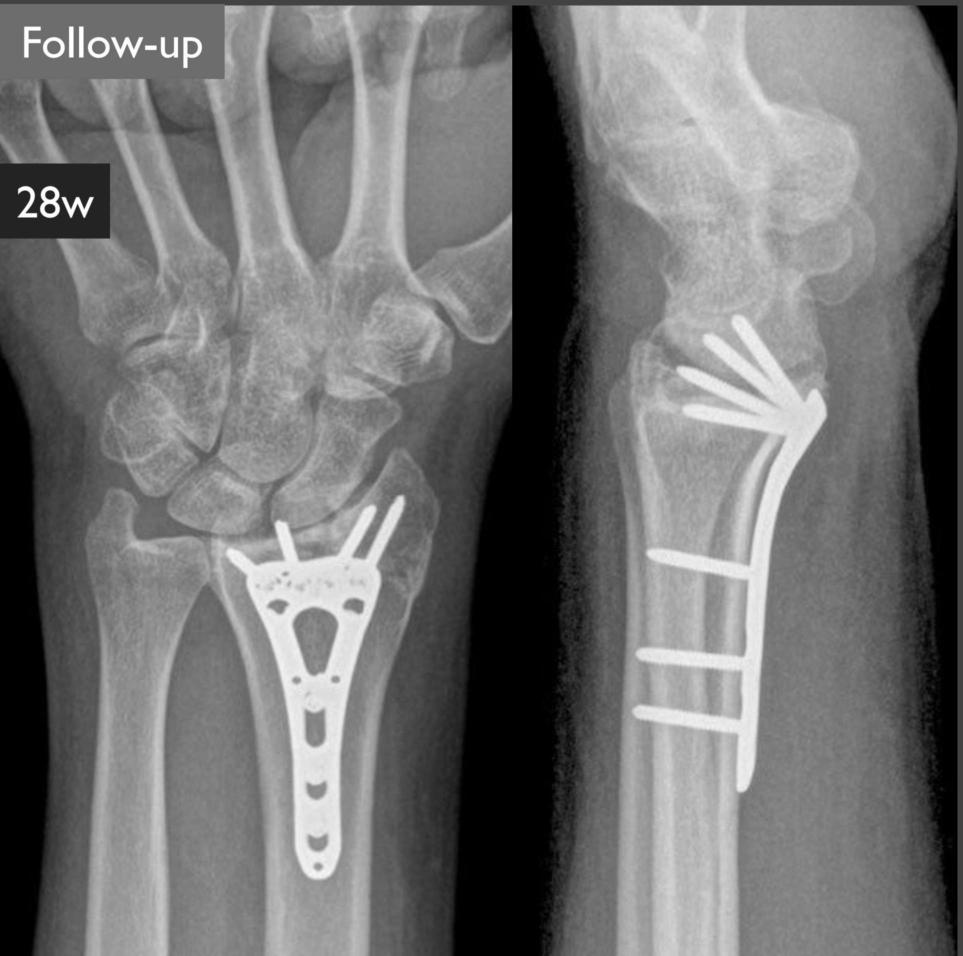

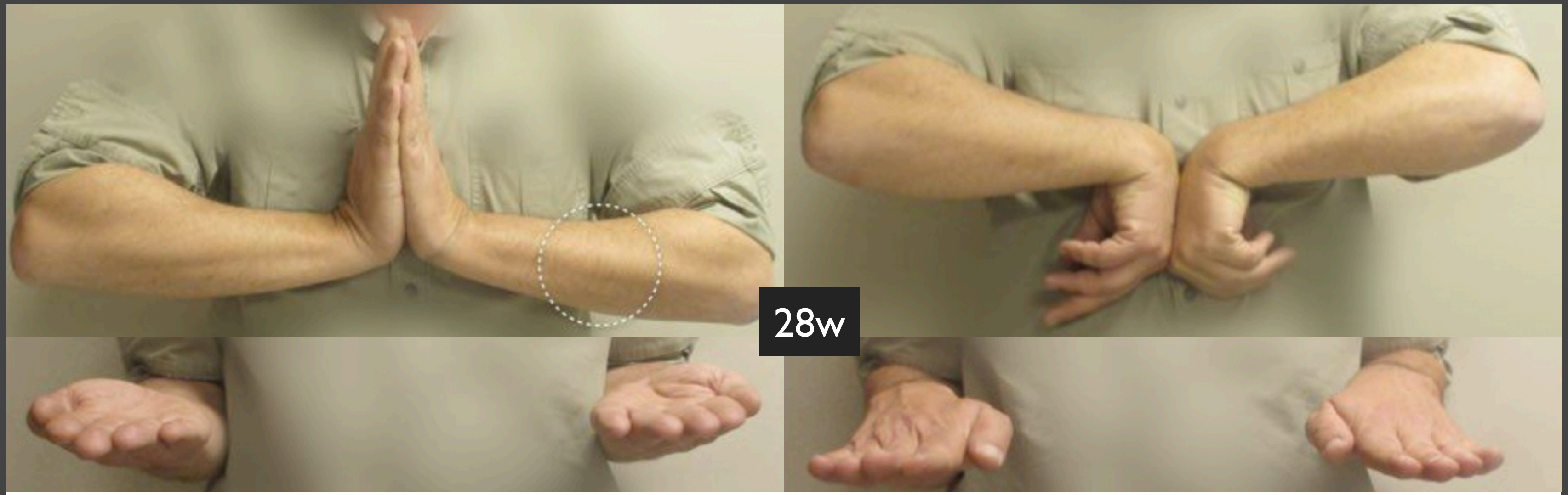

ICUC Score **Functional Limitation: 1** (0-4) - **Pain: 1** (0-4)

ICUC Score at 246w Functional limitation: 0 Pain: 0

Quick DASH = 7

ICUC® ID: 23-VC-803 / 60y Female

*Complete case data can be accessed by clicking the **ICUC ID***

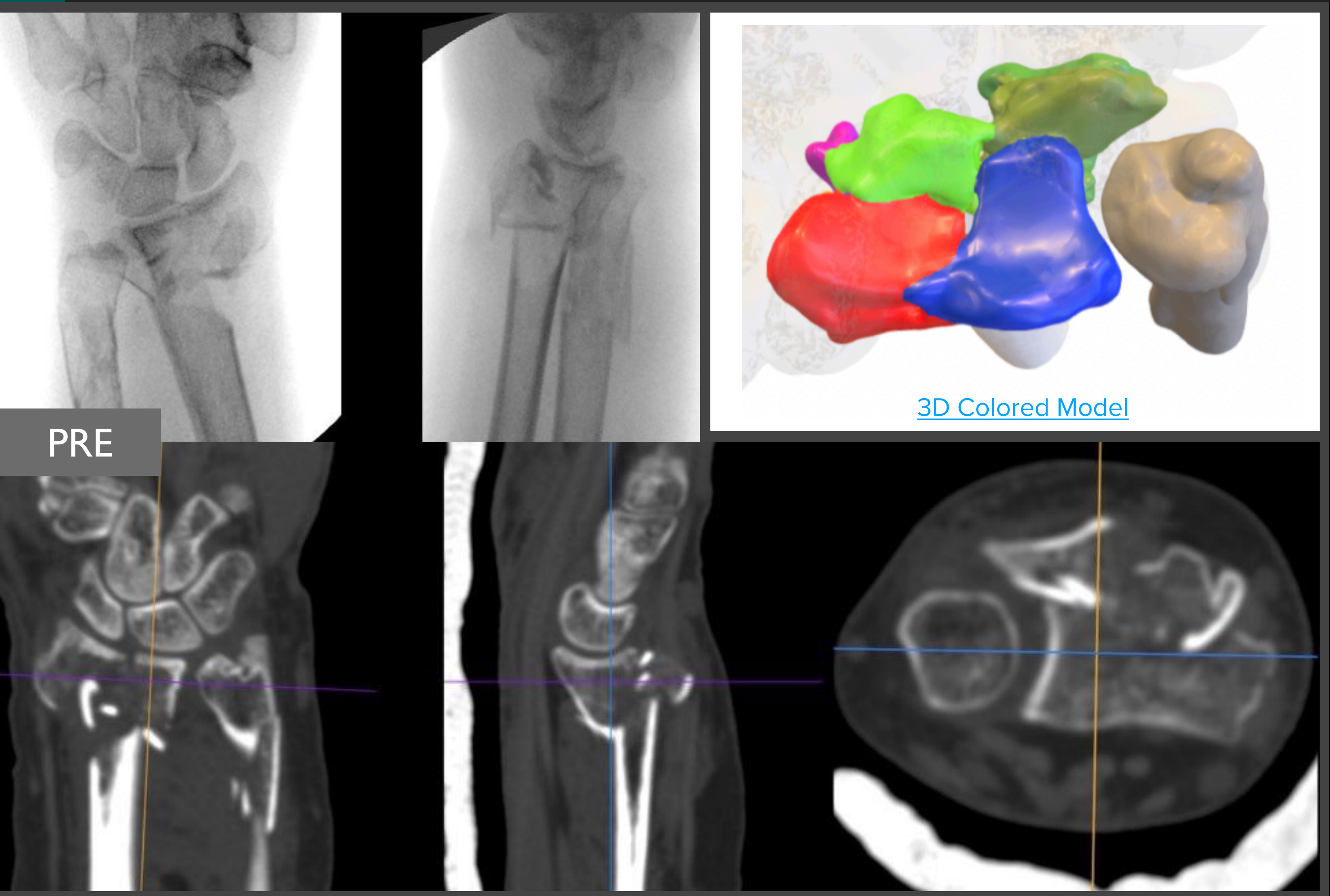

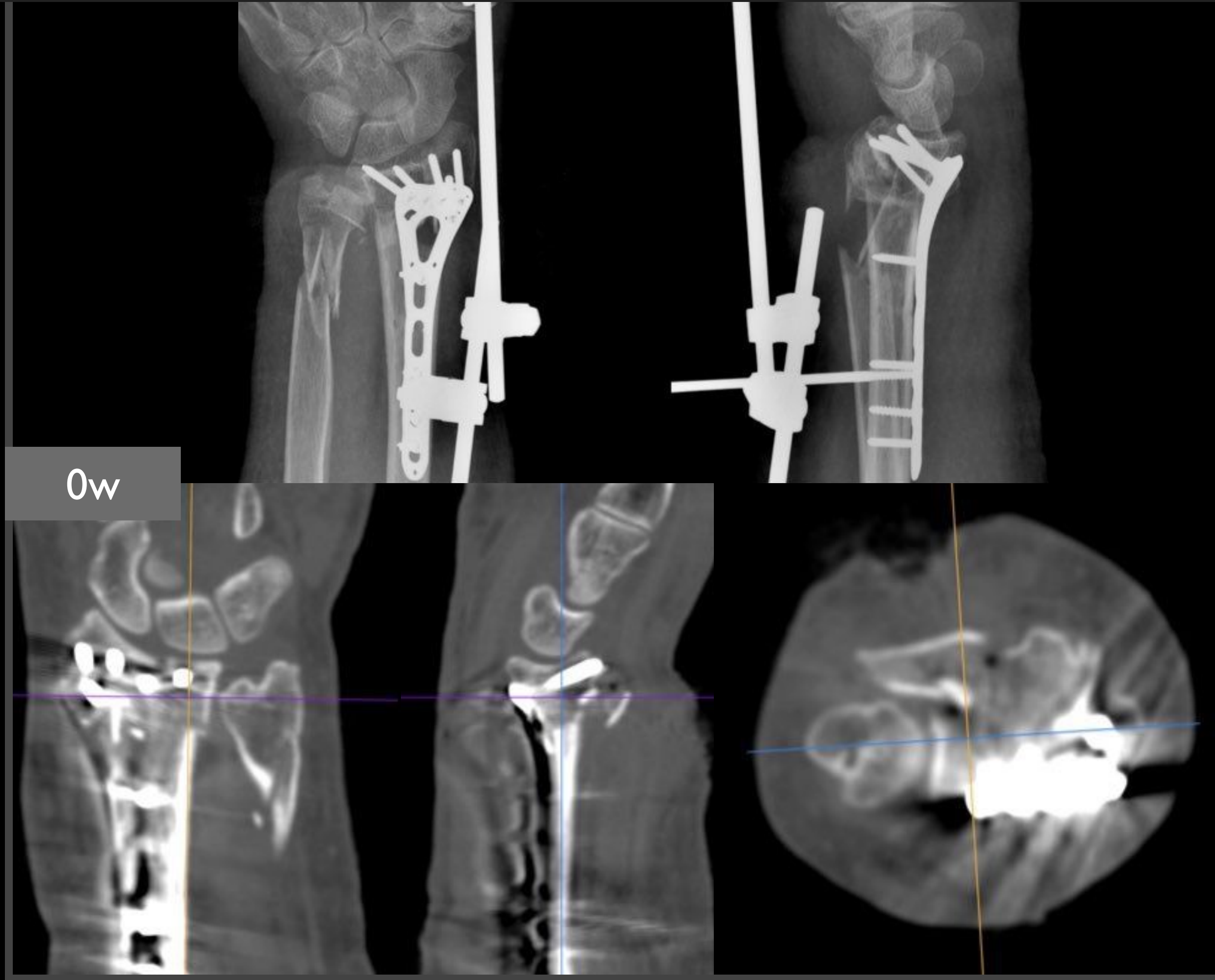

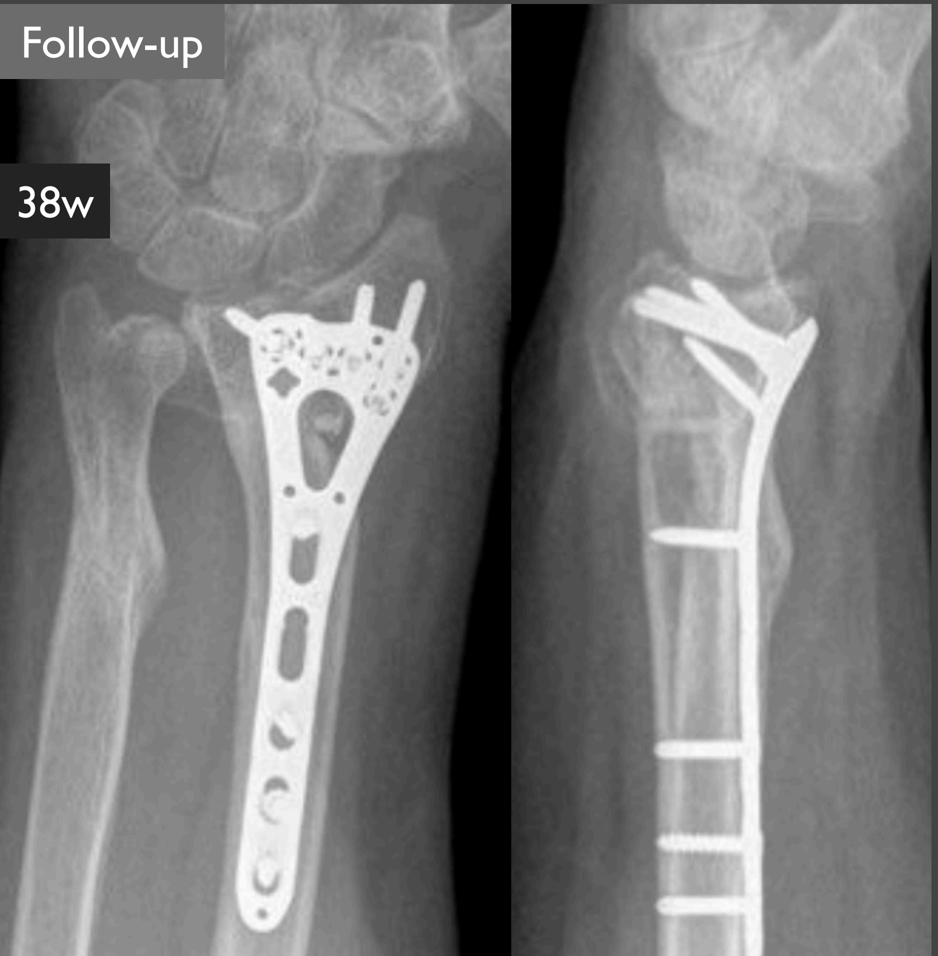

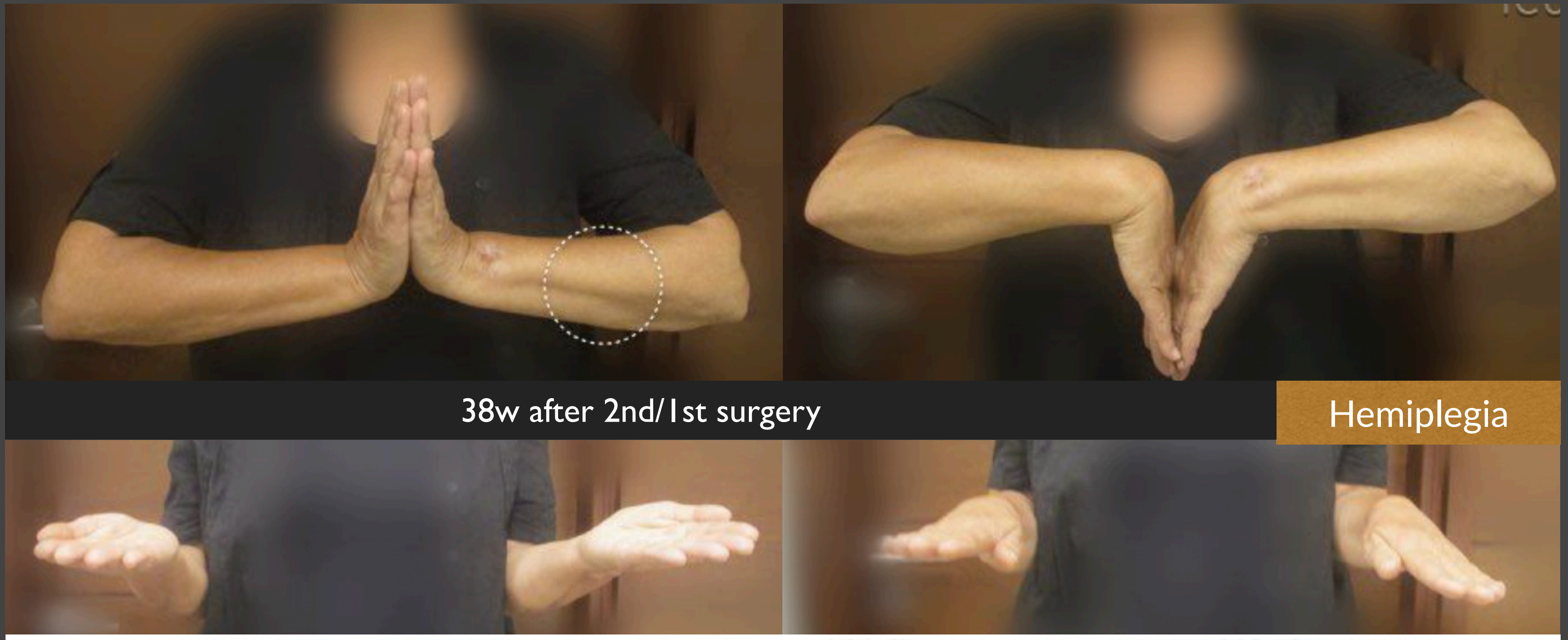

38w after 2nd/1st surgery

Hemiplegia

ICUC Score **Functional Limitation: 1** (0-4) - **Pain: 1** (0-4)

Quick DASH = 60

ICUC® ID: 23-VC-881 / 65y Female

Complete case data can be accessed by clicking the ***ICUC ID***

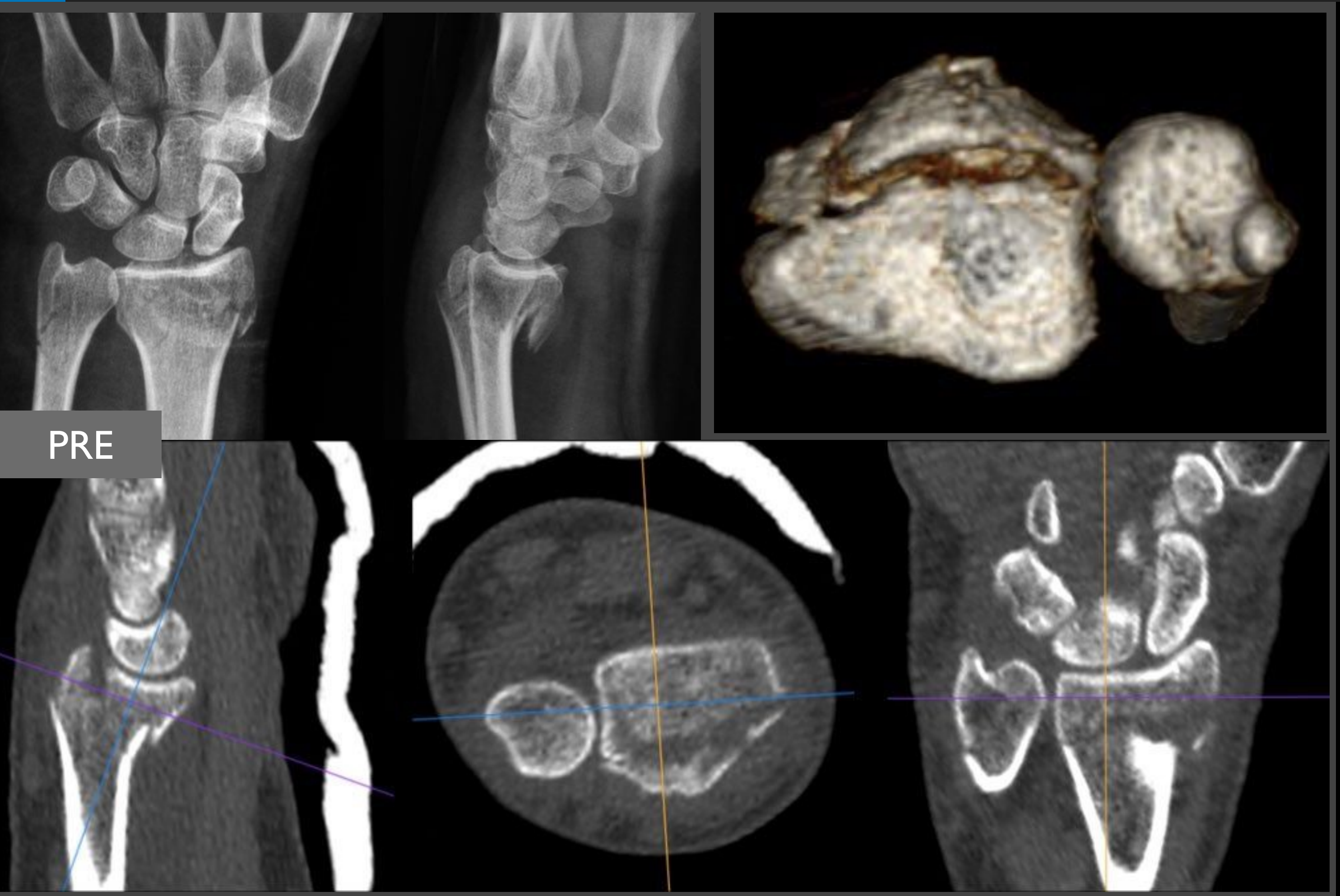

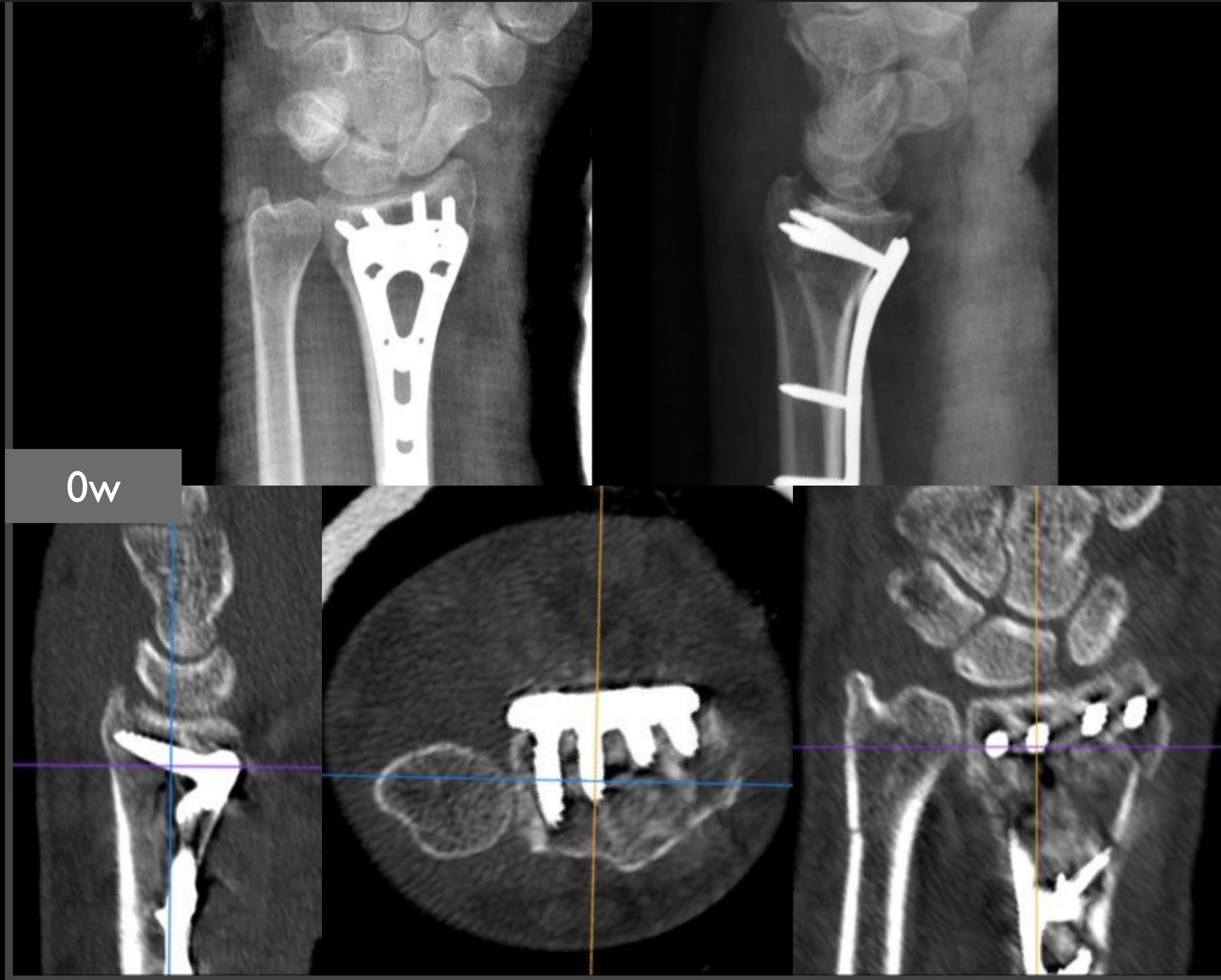

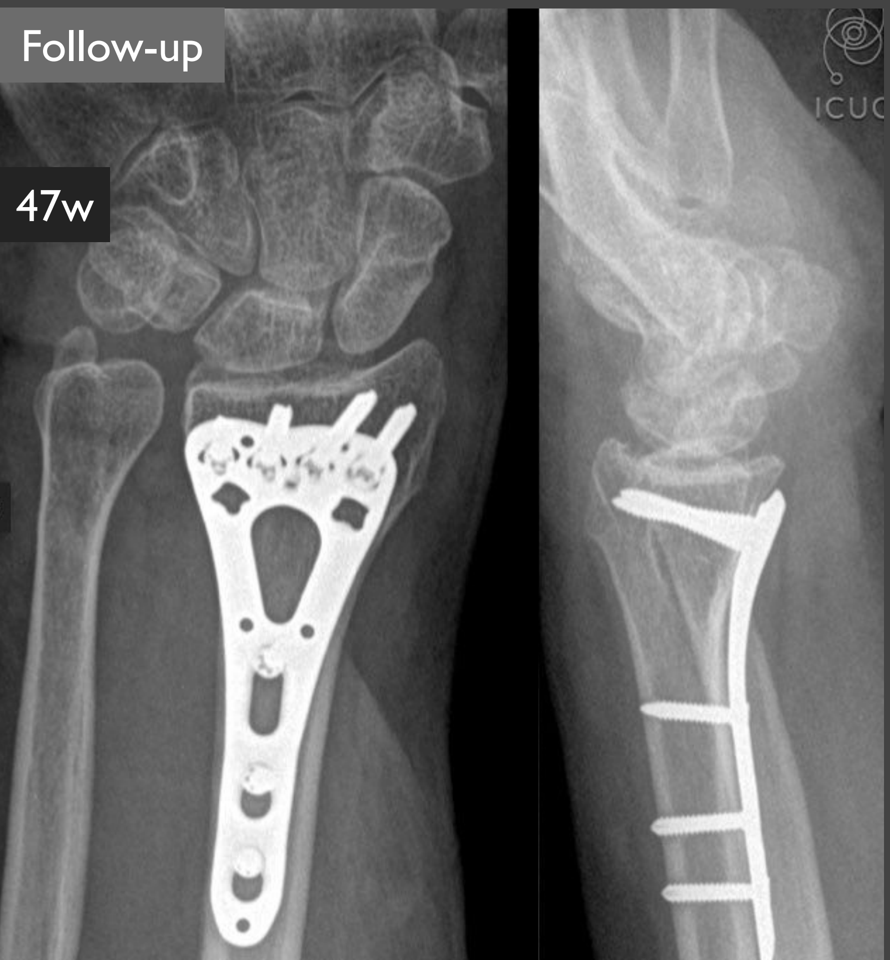

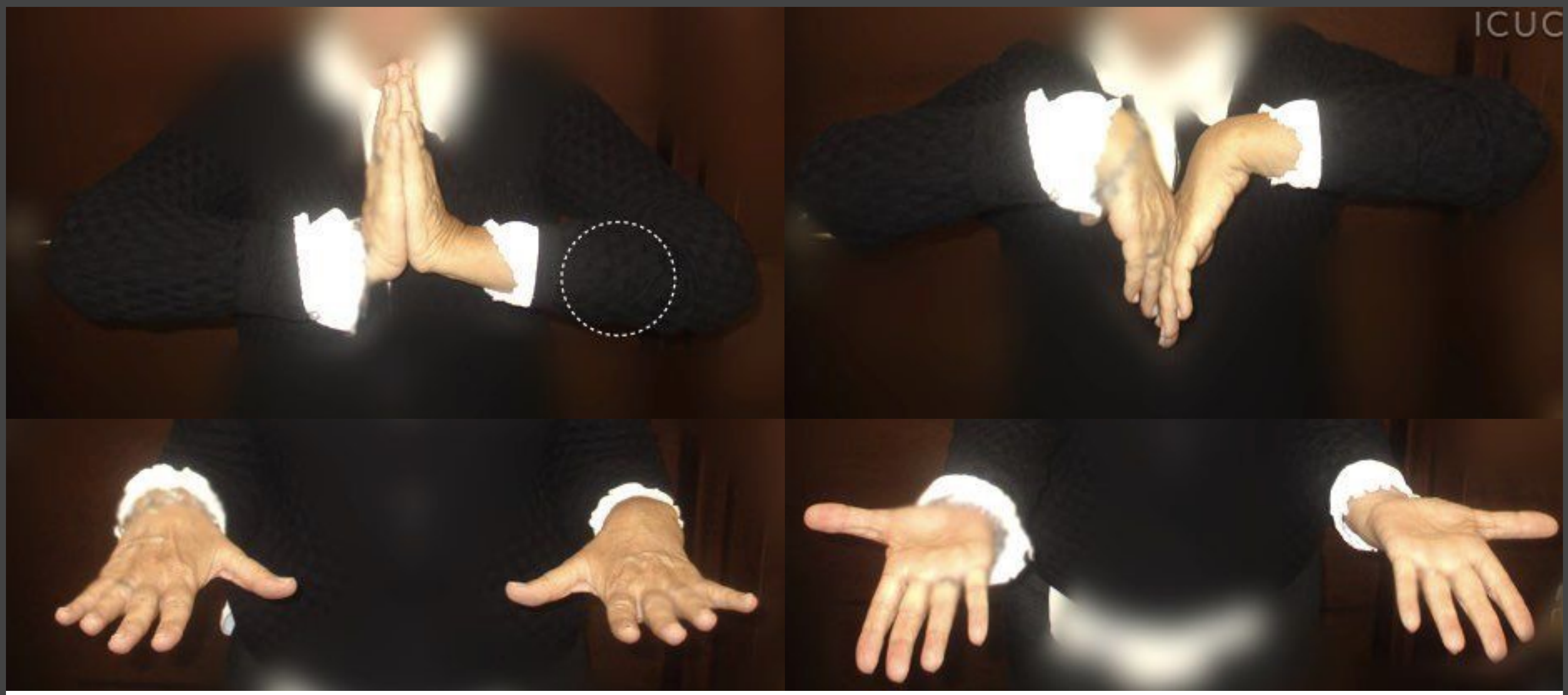

ICUC **Score** ***Functional Limitation:*** **0** (0-4) - ***Pain:*** **1** (0-4)

Quick DASH = 6

ICUC® ID: 23-VC-376 / 40y Female

Complete case data can be accessed by clicking the ***ICUC ID***

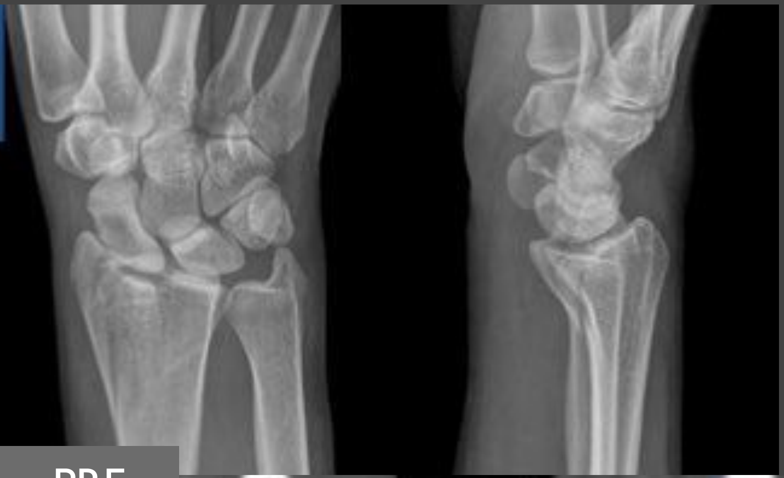

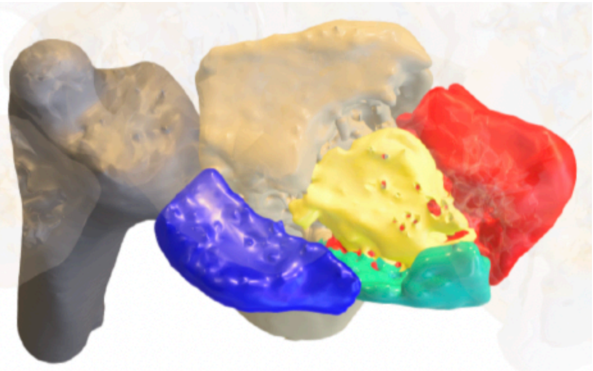

3D Colored Model

PRE

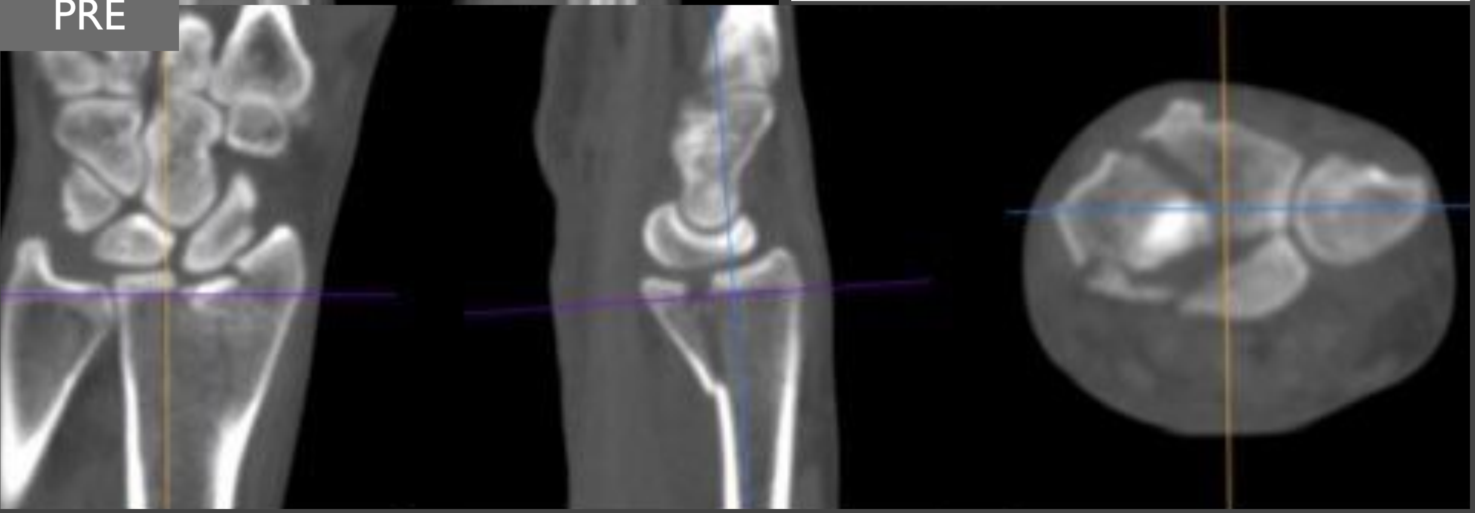

0w

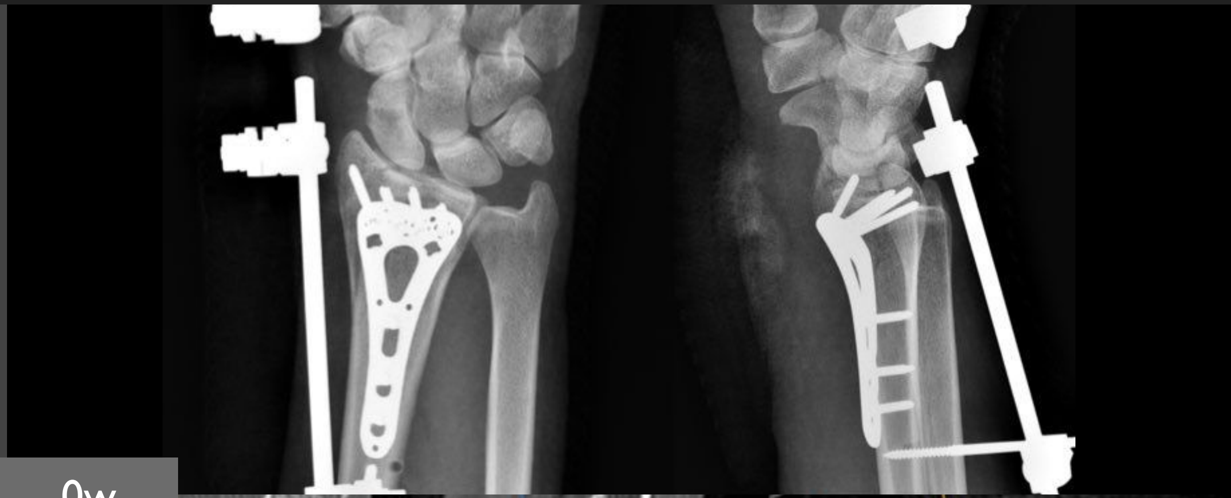

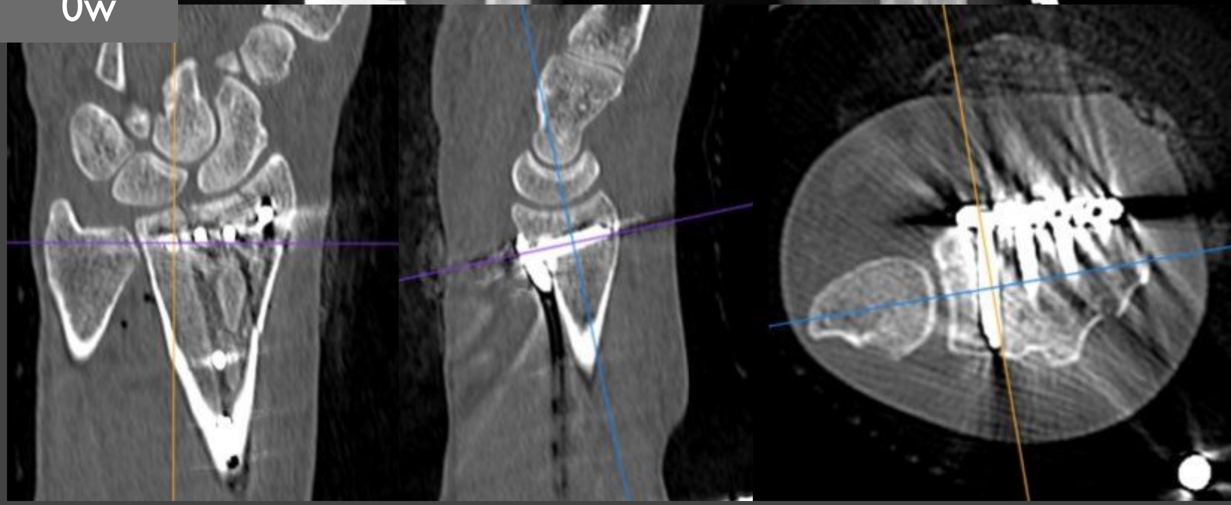

Follow-up

23w

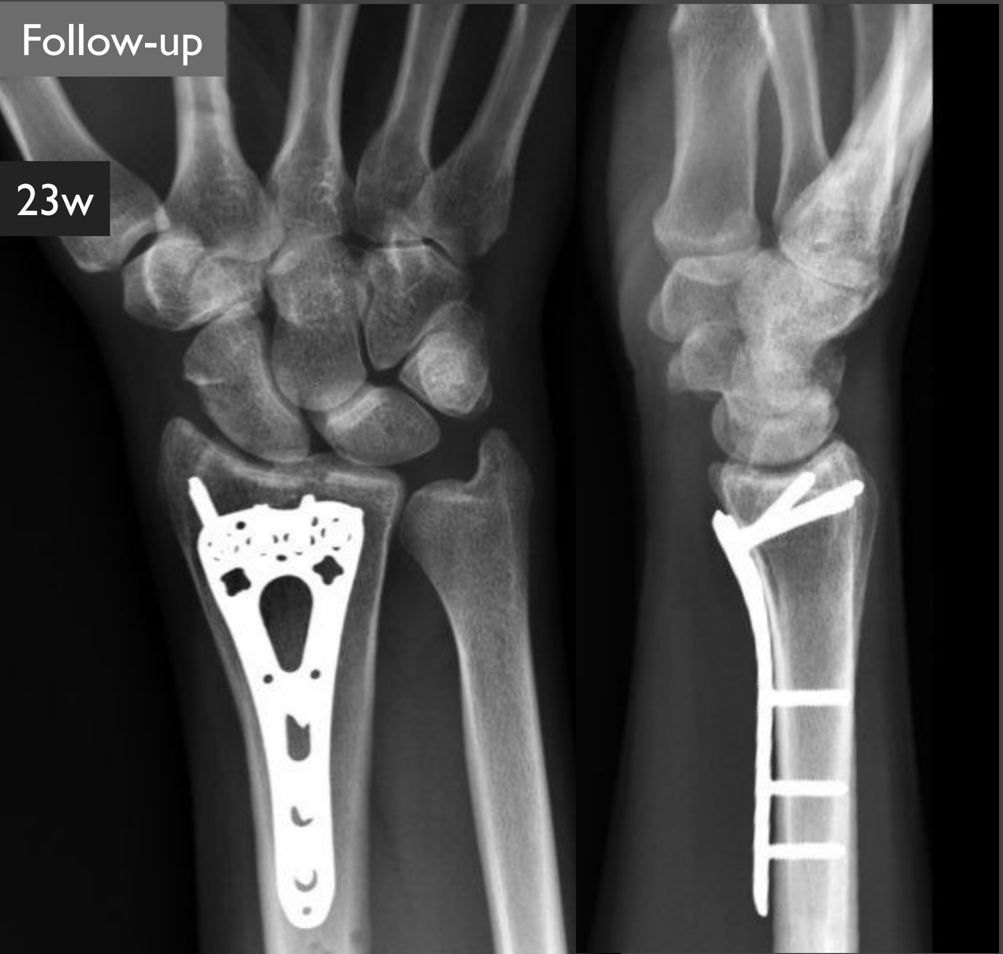

ICUC Score at 182w Functional limitation: 0 Pain: 0

Quick DASH = 0

ICUC® ID: 23-VC-761 / 80y Female

*Complete case data can be accessed by clicking the **ICUC ID***

PRE

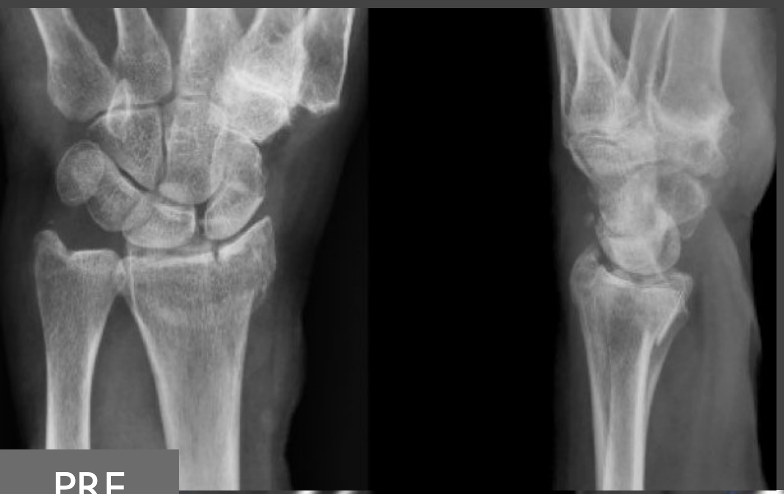

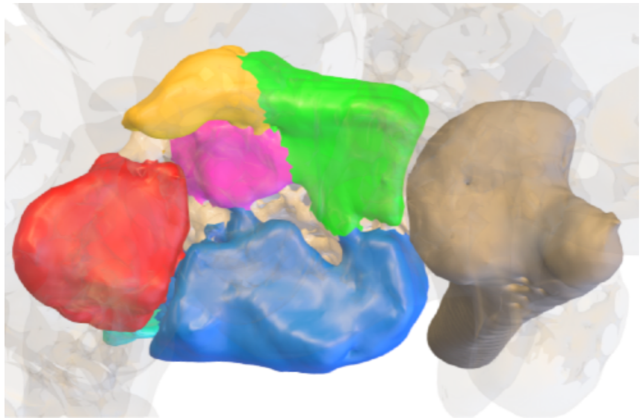

3D Colored Model

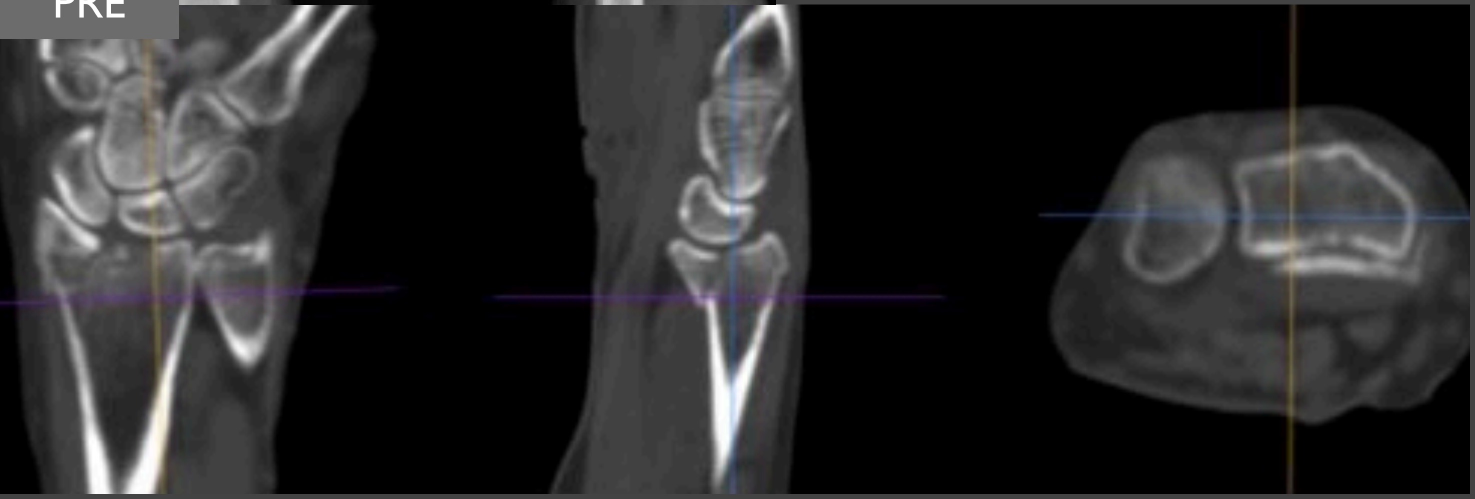

0w

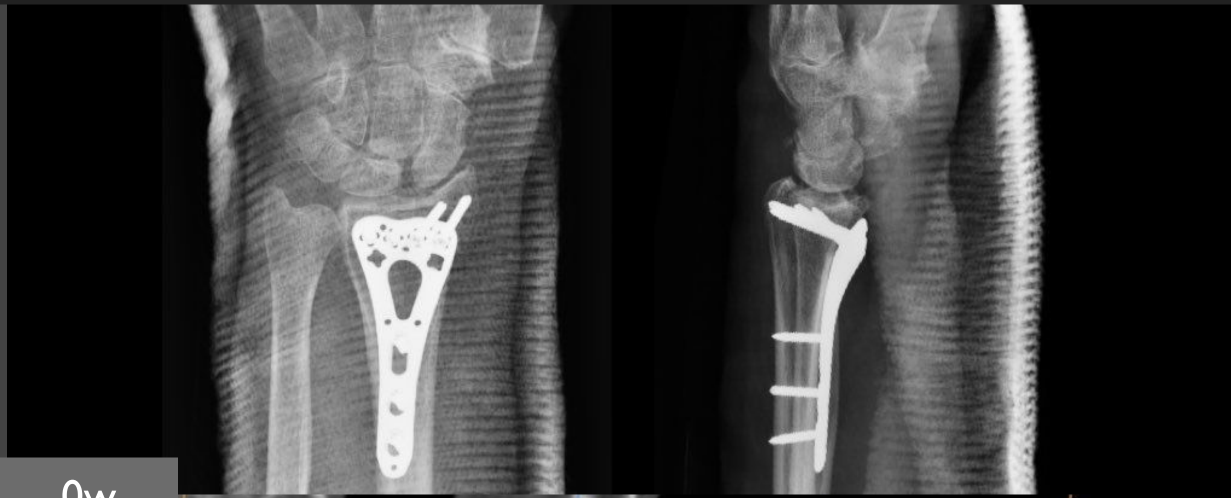

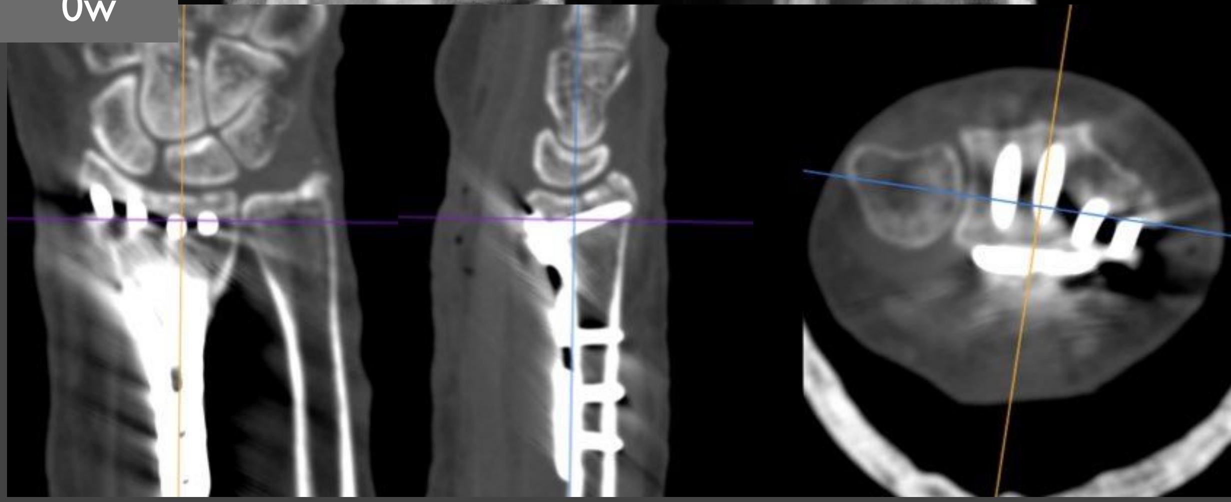

Follow-up

8w

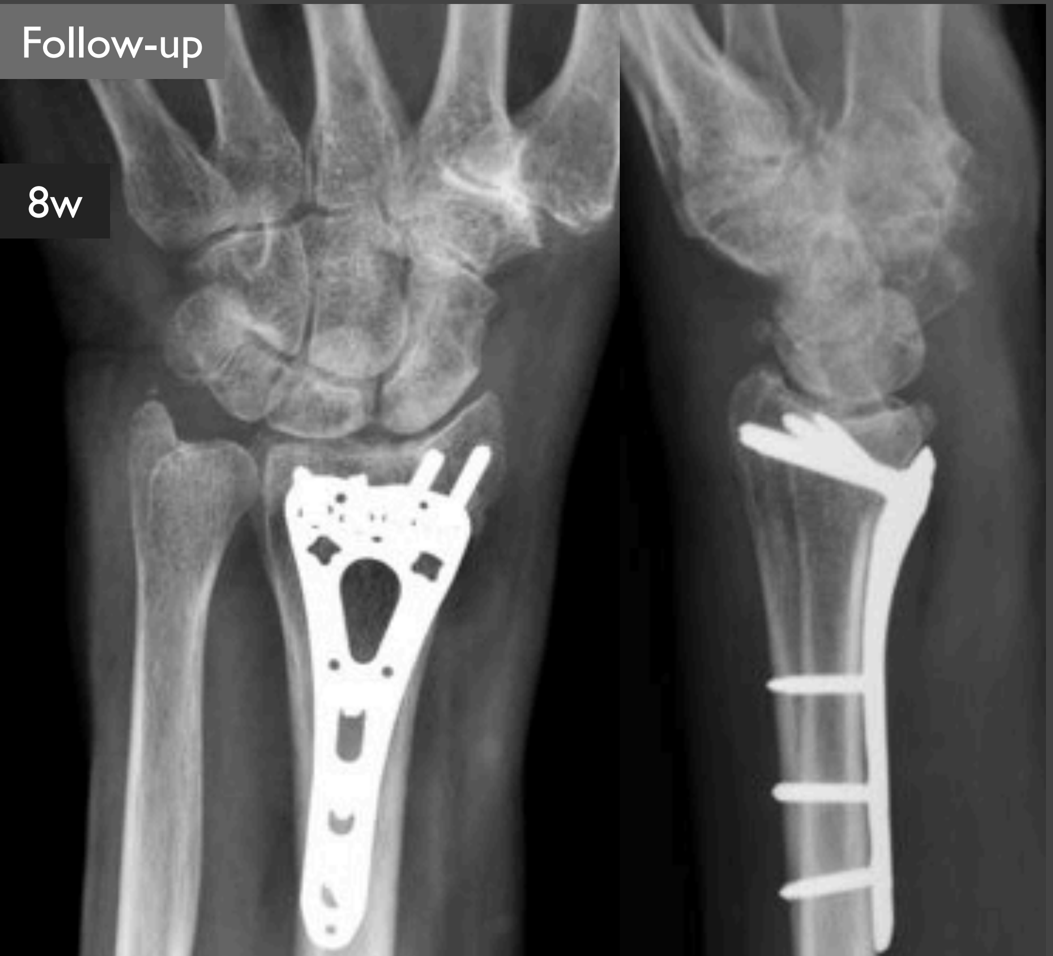

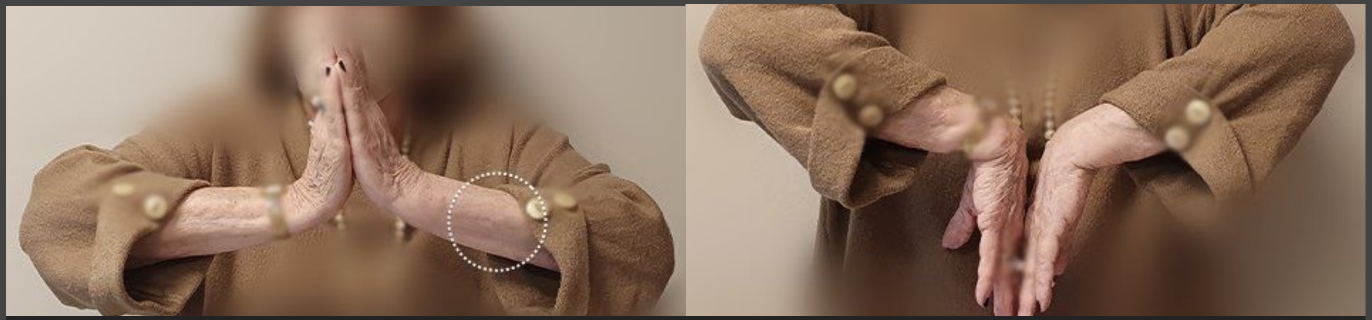

0w after 2nd surgery | 8w after 1st surgery

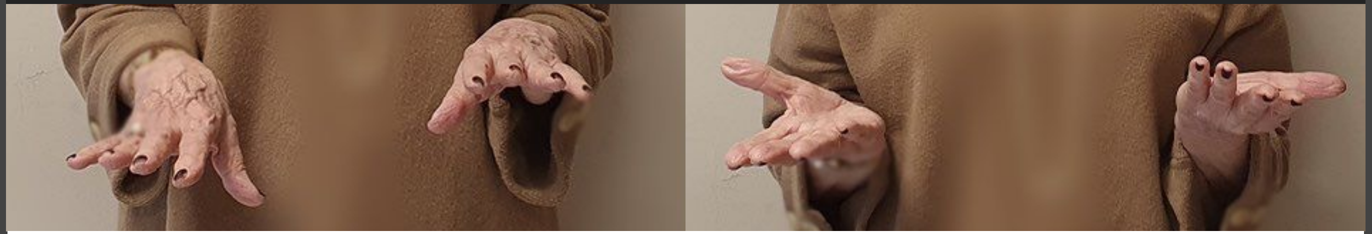

ICUC Score *Functional Limitation:* **1** (0-4) - *Pain:* **1** (0-4)

ICUC Score at 161w Functional limitation: 0 Pain: 0

Quick DASH = 9

ICUC® ID: 23-VC-780 / 65y Male

Complete case data can be accessed by clicking the ***ICUC ID***

PRE

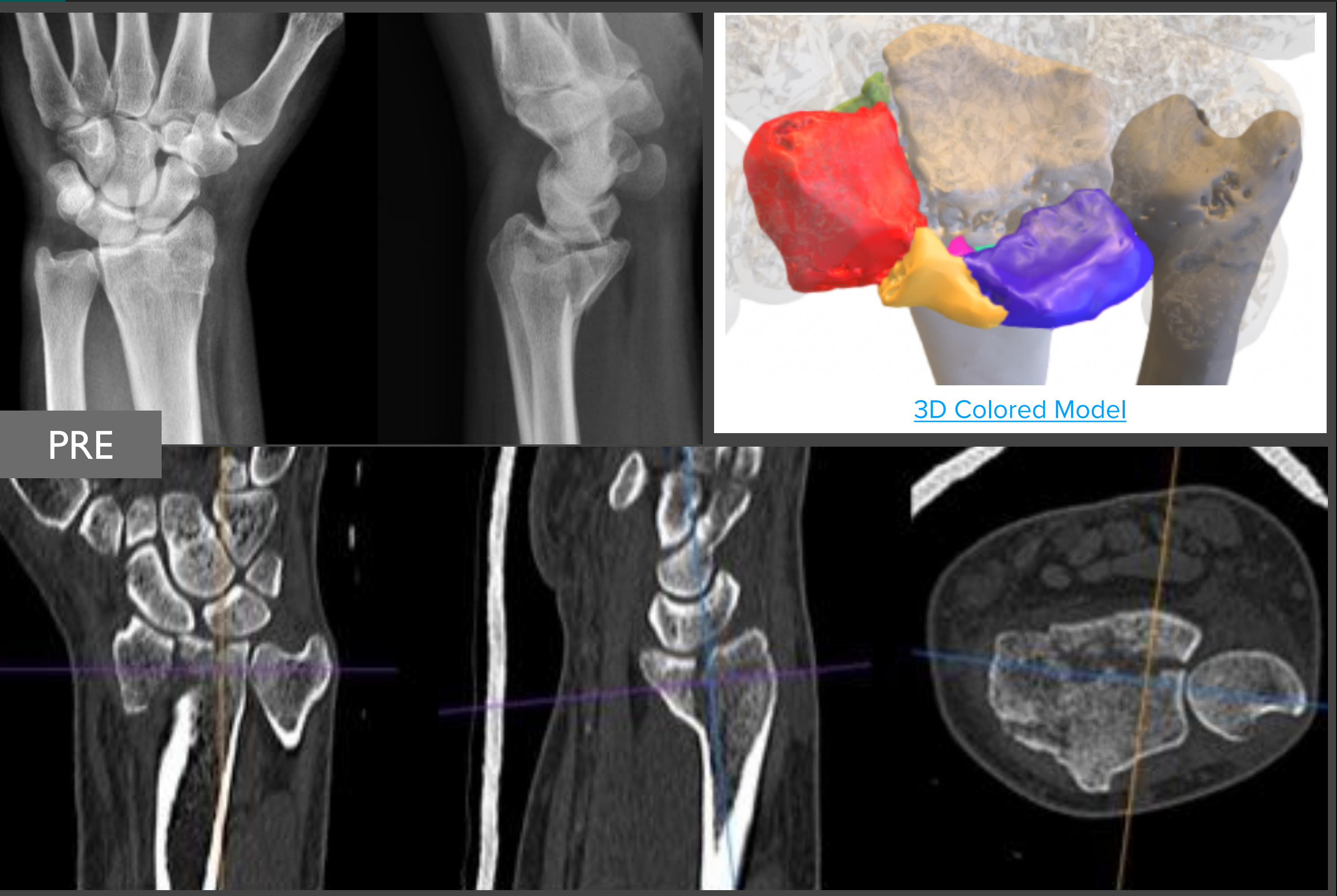

0w

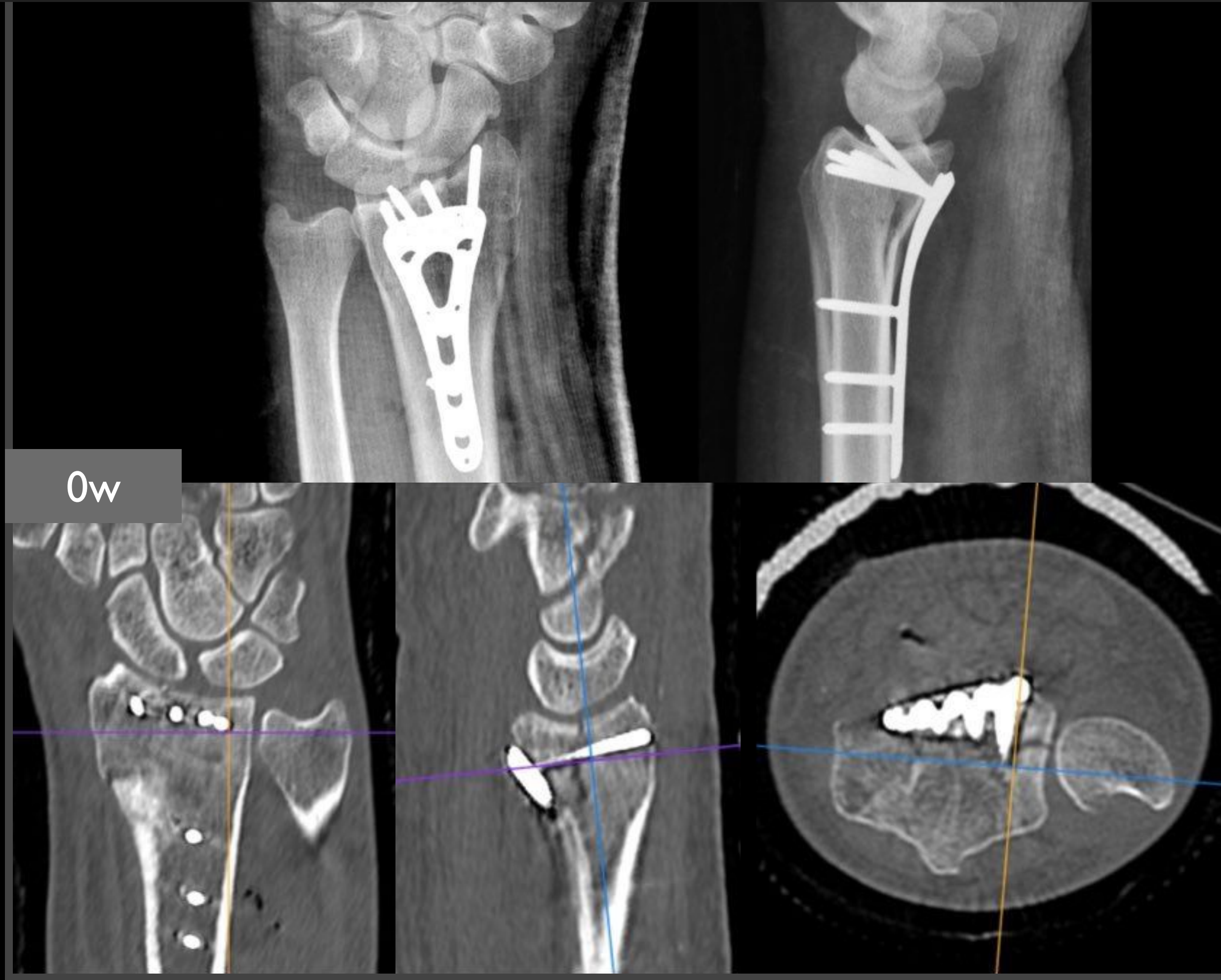

Follow-up

10w

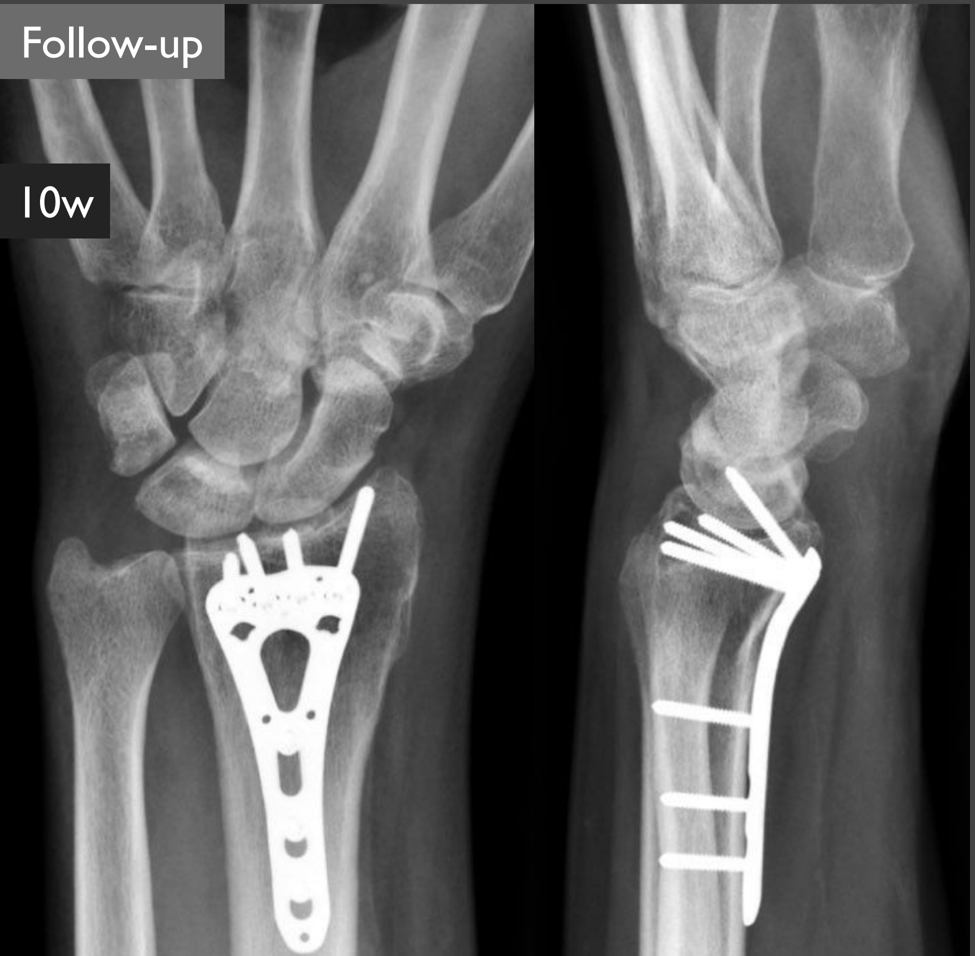

5w

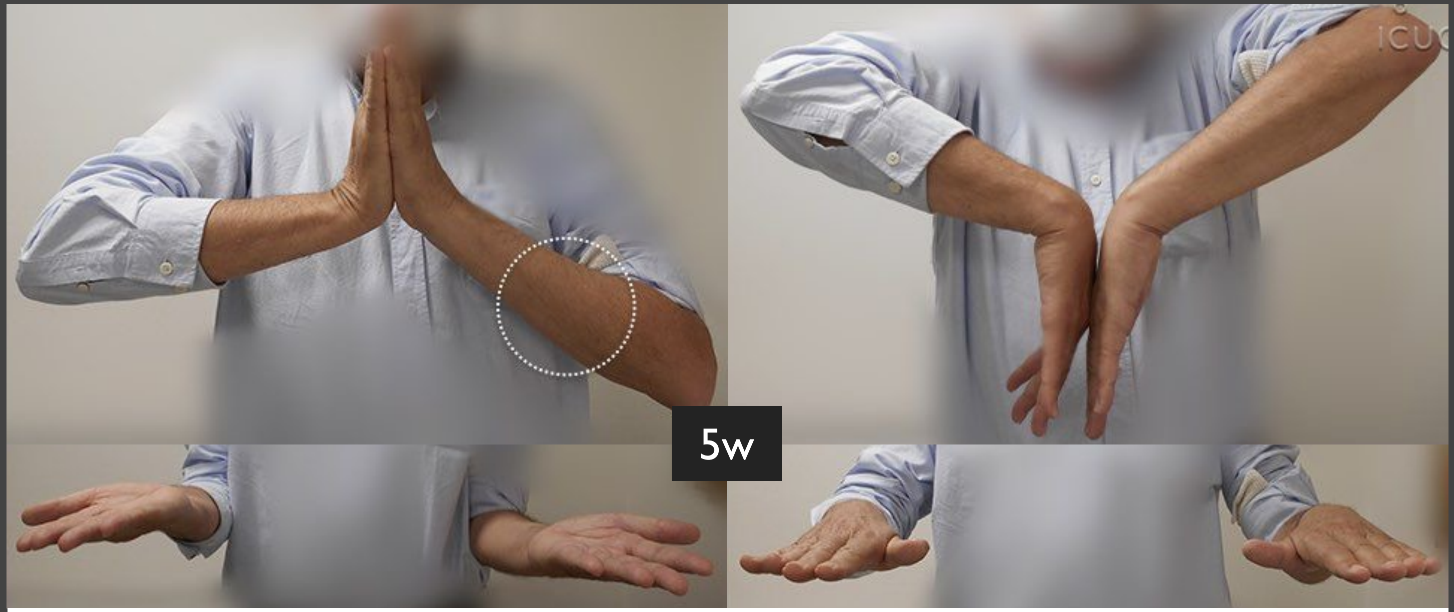

ICUC Score **Functional Limitation: 1** (0-4) - **Pain: 0** (0-4)

ICUC Score at 93w Functional limitation: 0 Pain: 0

Quick DASH = 0

ICUC® ID: 23-VC-183 / 75y Female

Complete case data can be accessed by clicking the ***ICUC ID***

PRE

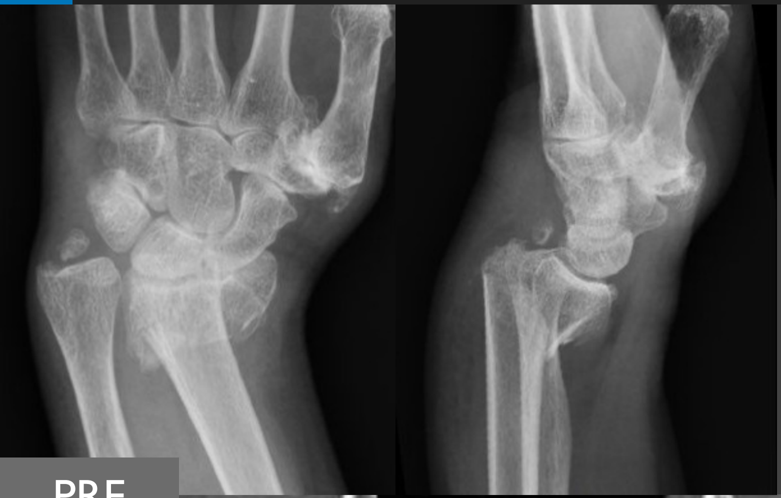

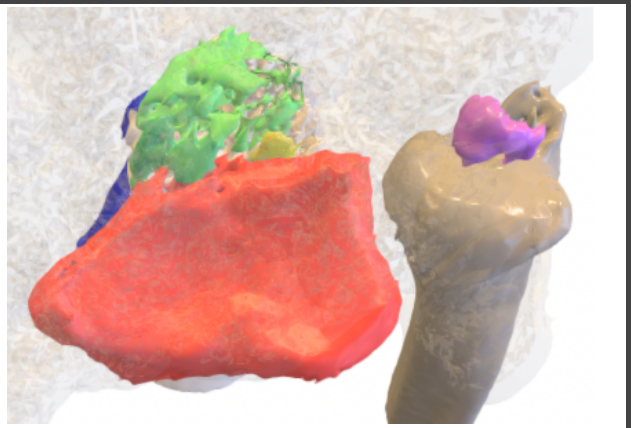

3D Colored Model

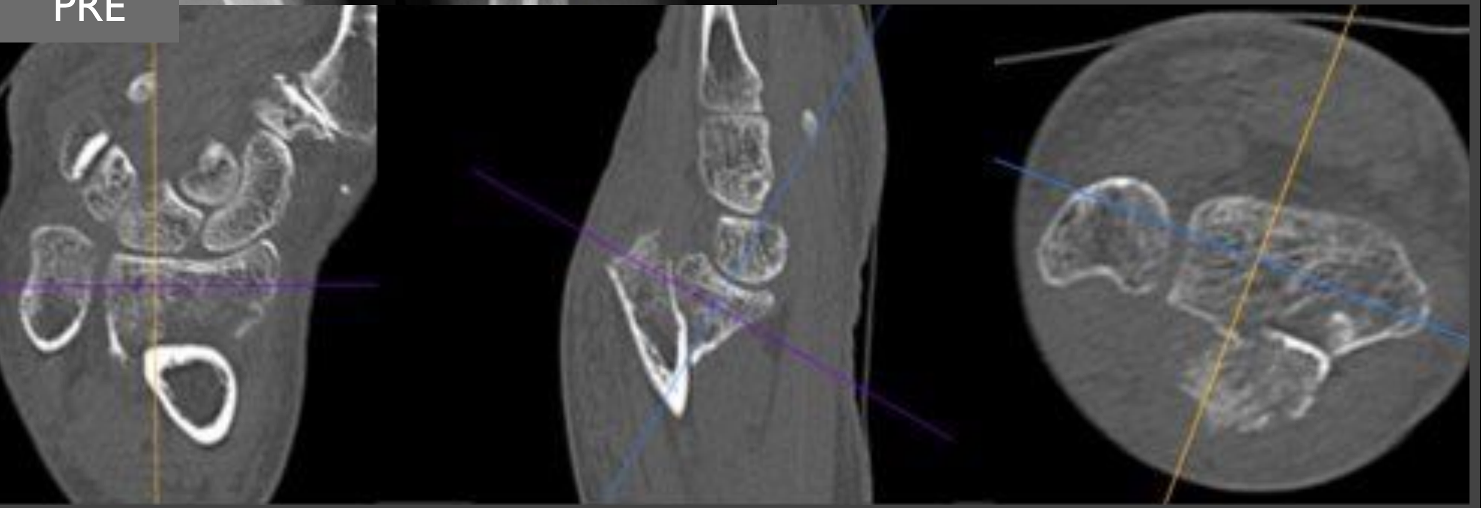

0w

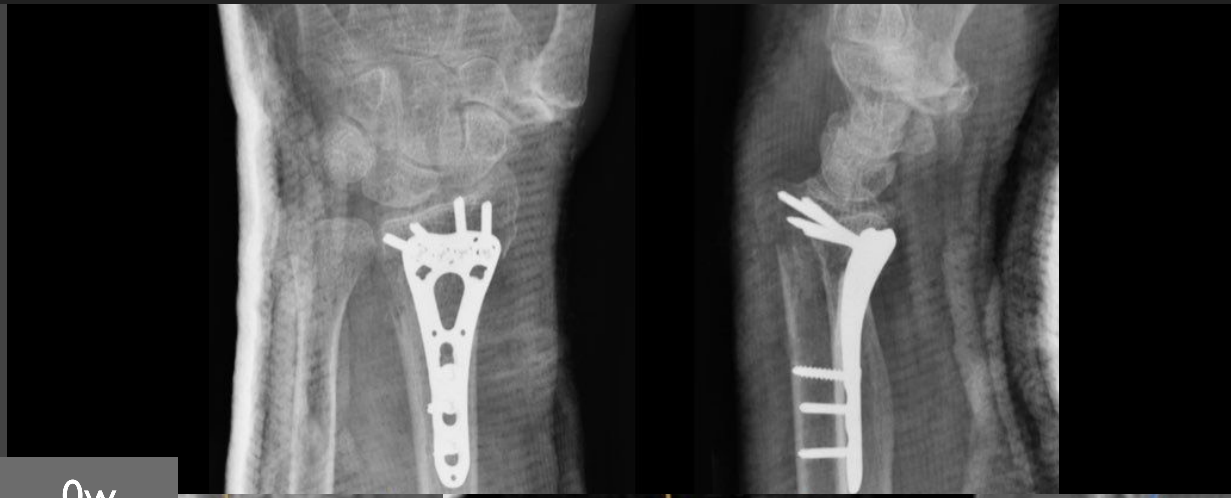

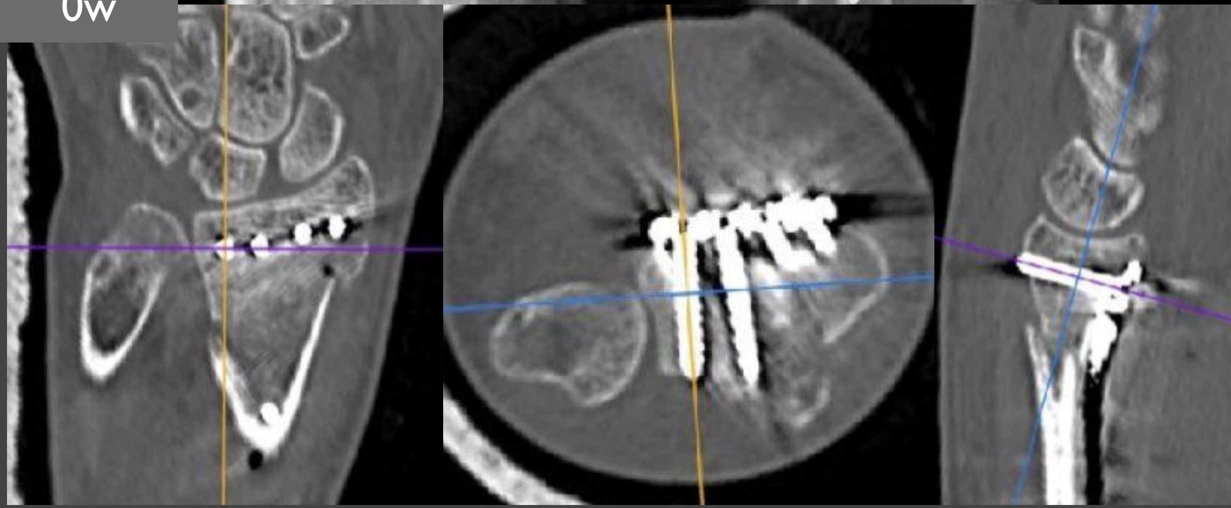

Follow-up

56w

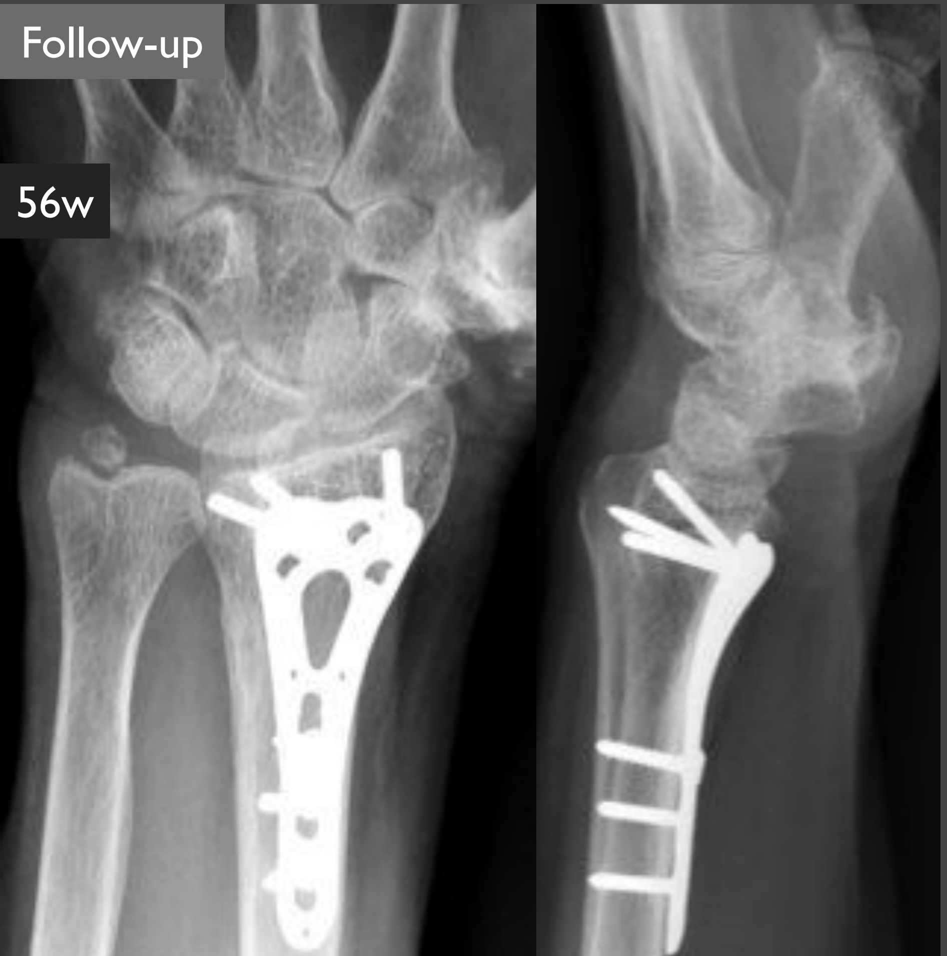

ICUC Score at 86w Functional limitation: 0 Pain: 0

Quick DASH = 0

ICUC® ID: 23-VC-255 / 60y Male

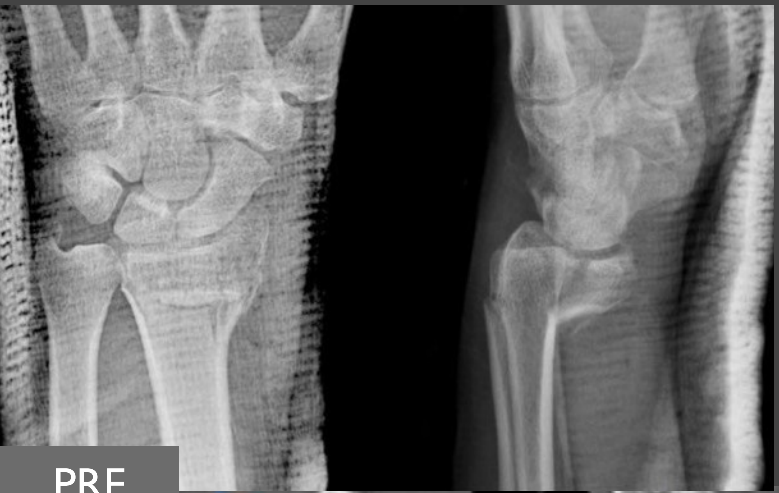

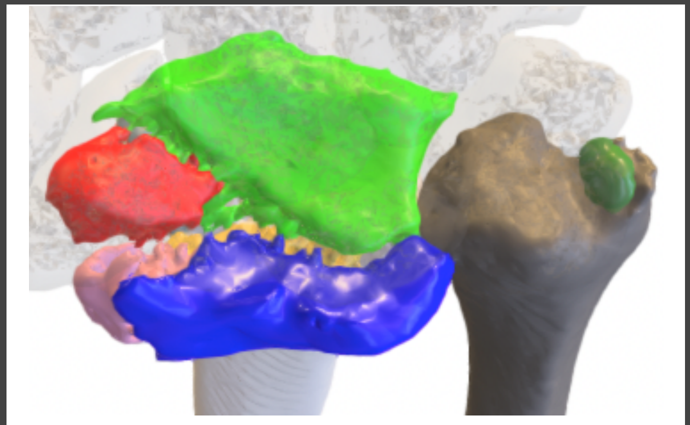

[3D Colored Model]

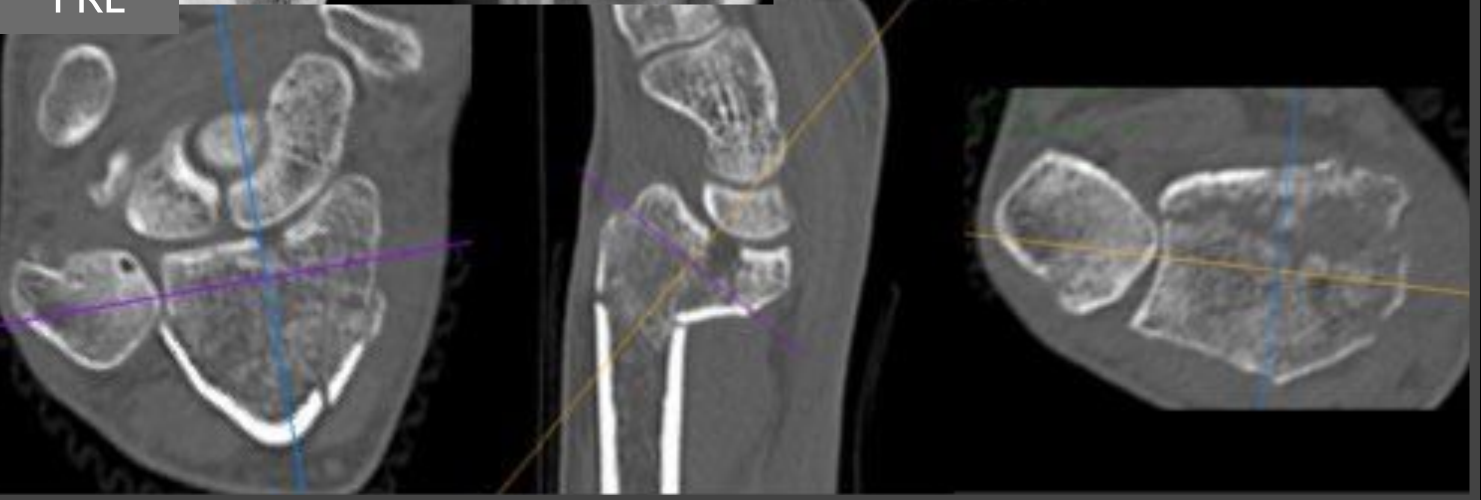

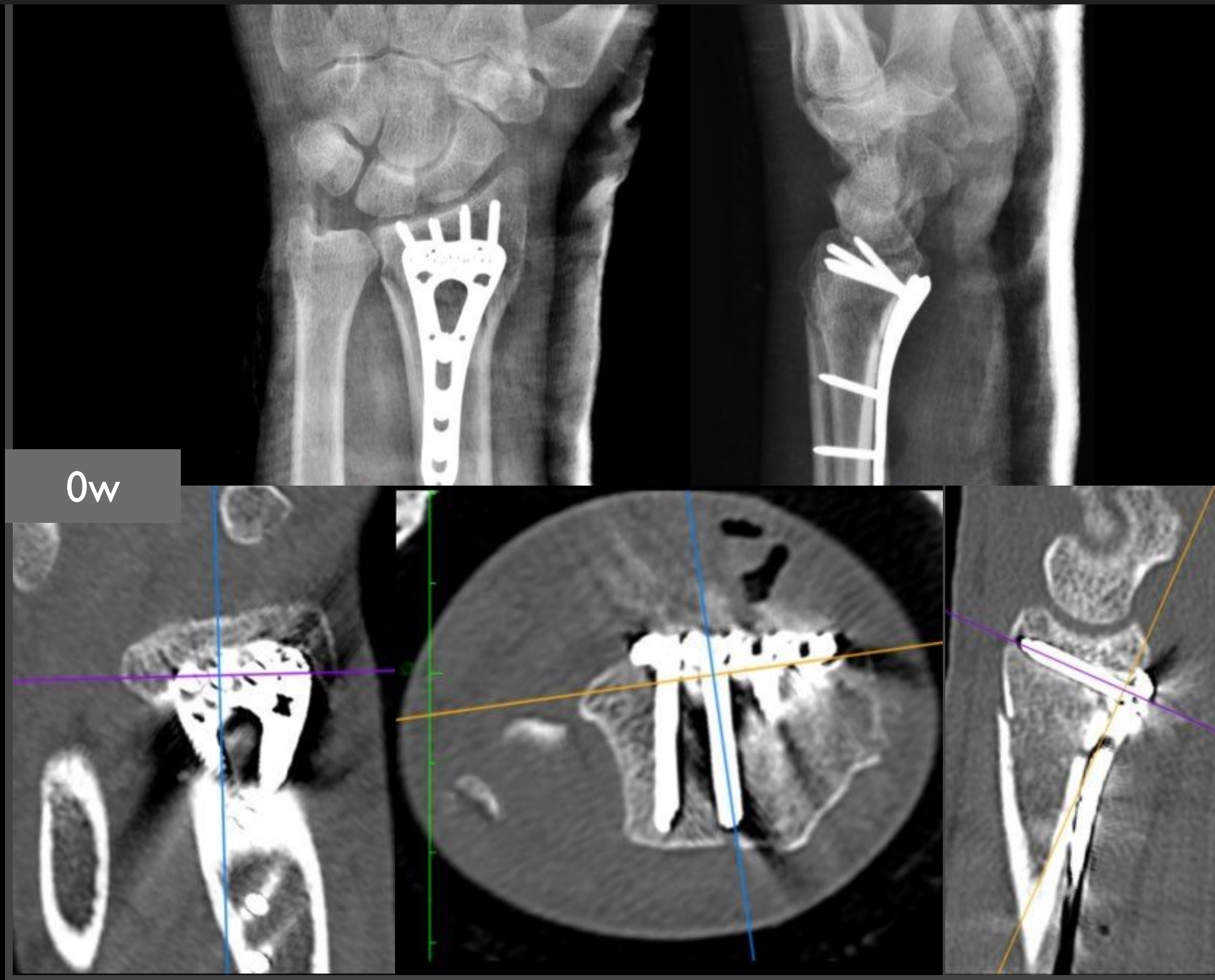

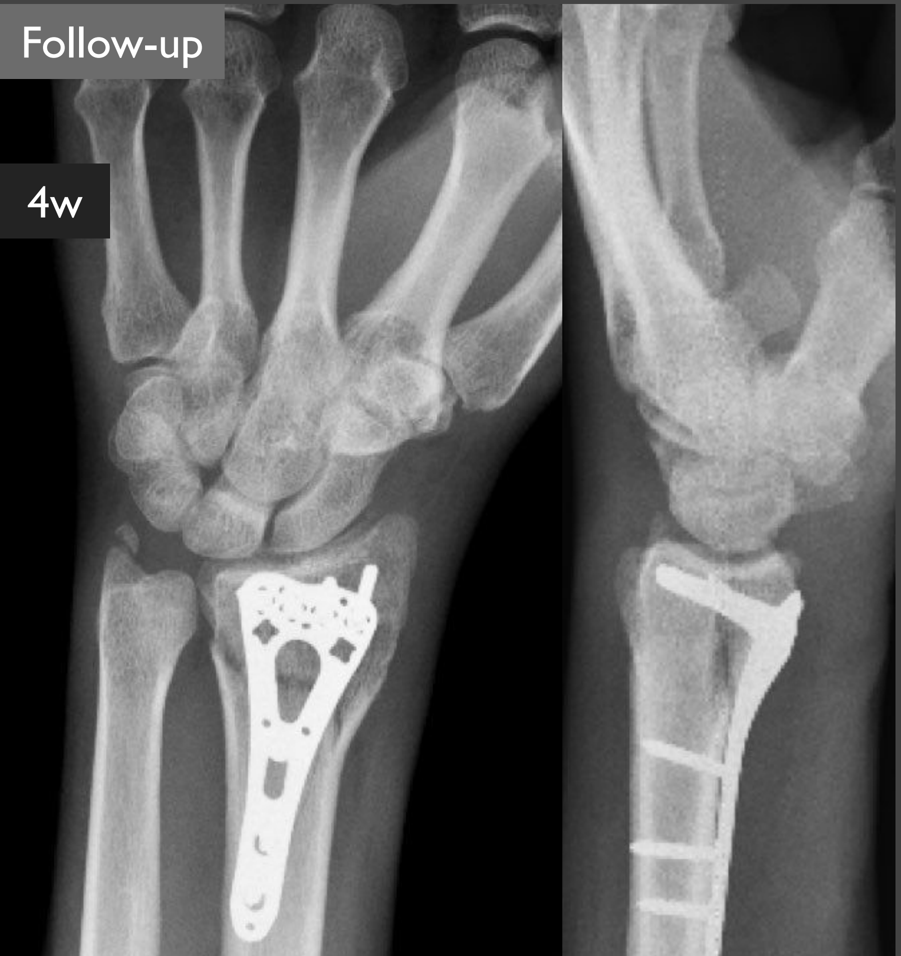

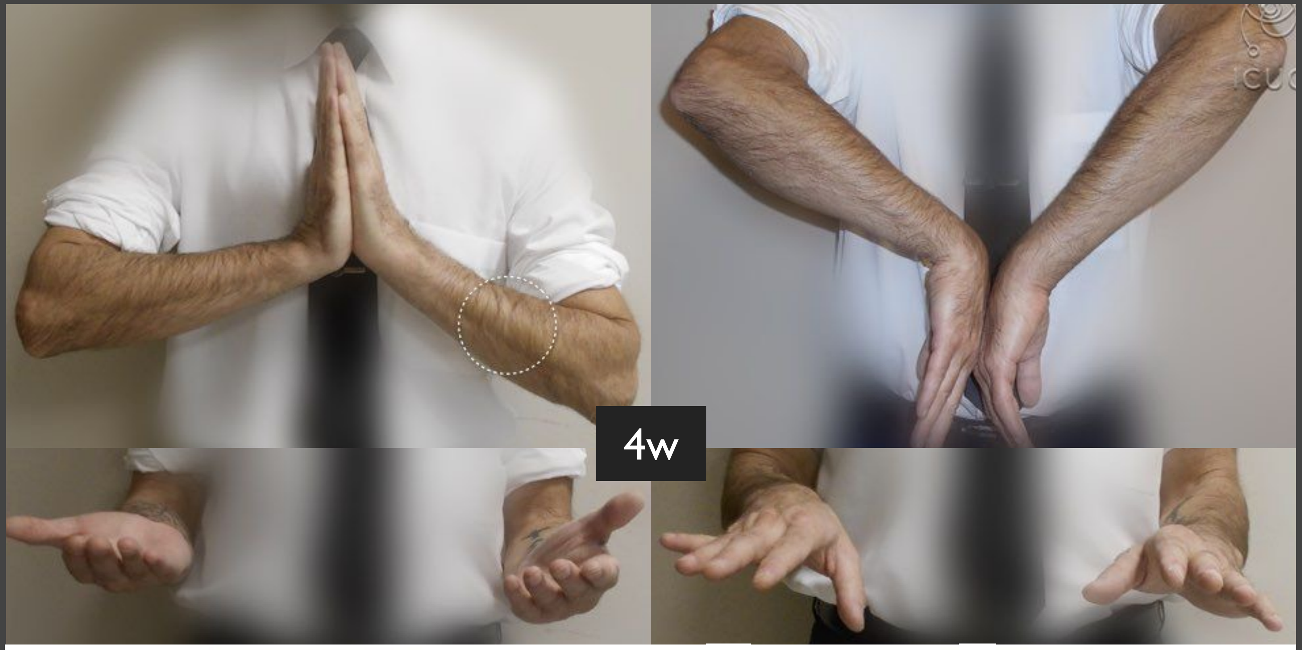

ICUC Score **Functional Limitation: 1** (0-4) - **Pain: 2** (0-4)

ICUC Score at 116w Functional limitation: 0 Pain: 0

Quick DASH = 0

ICUC® ID: 23-VC-646 / 60y Female

Complete case data can be accessed by clicking the ***ICUC ID***

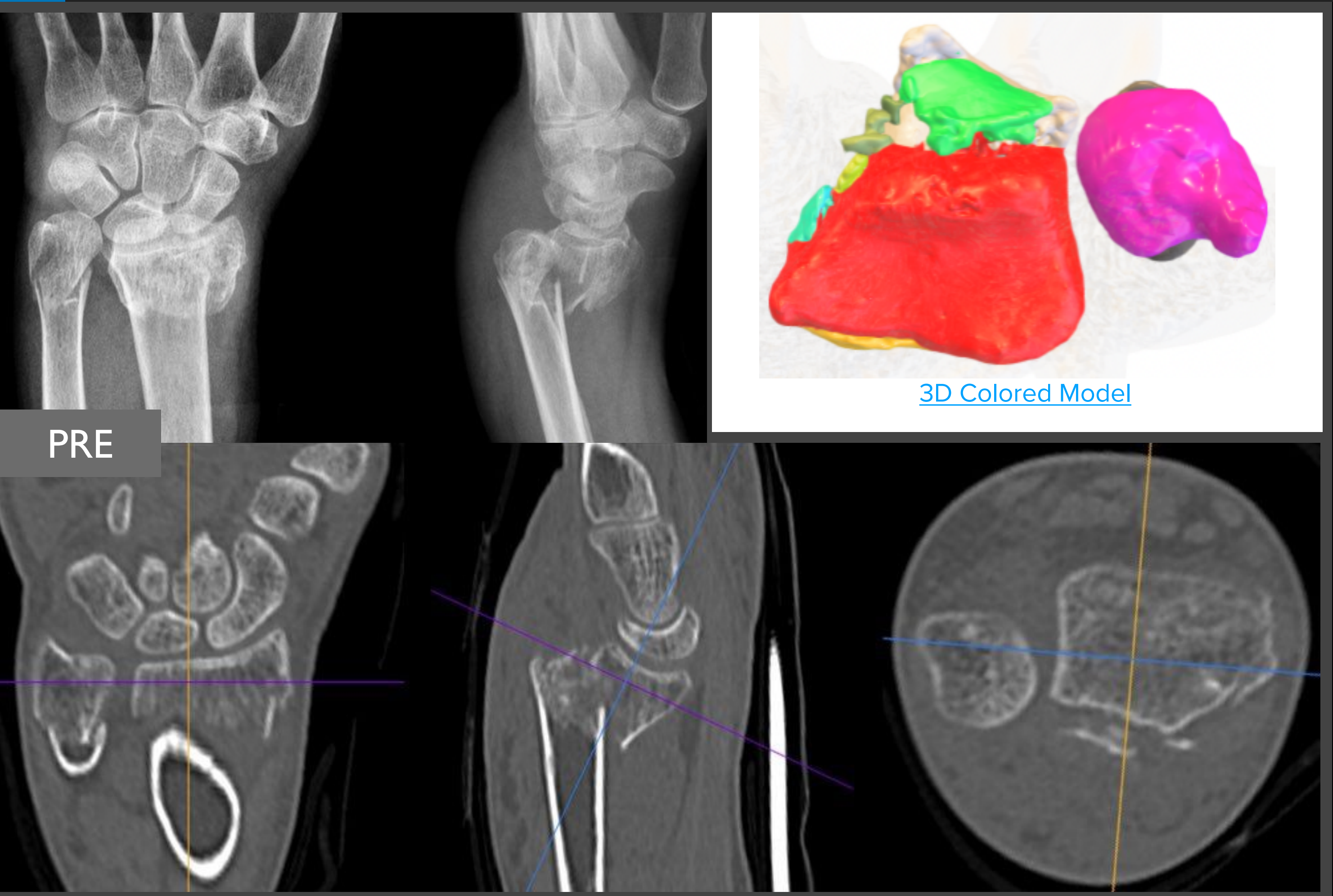

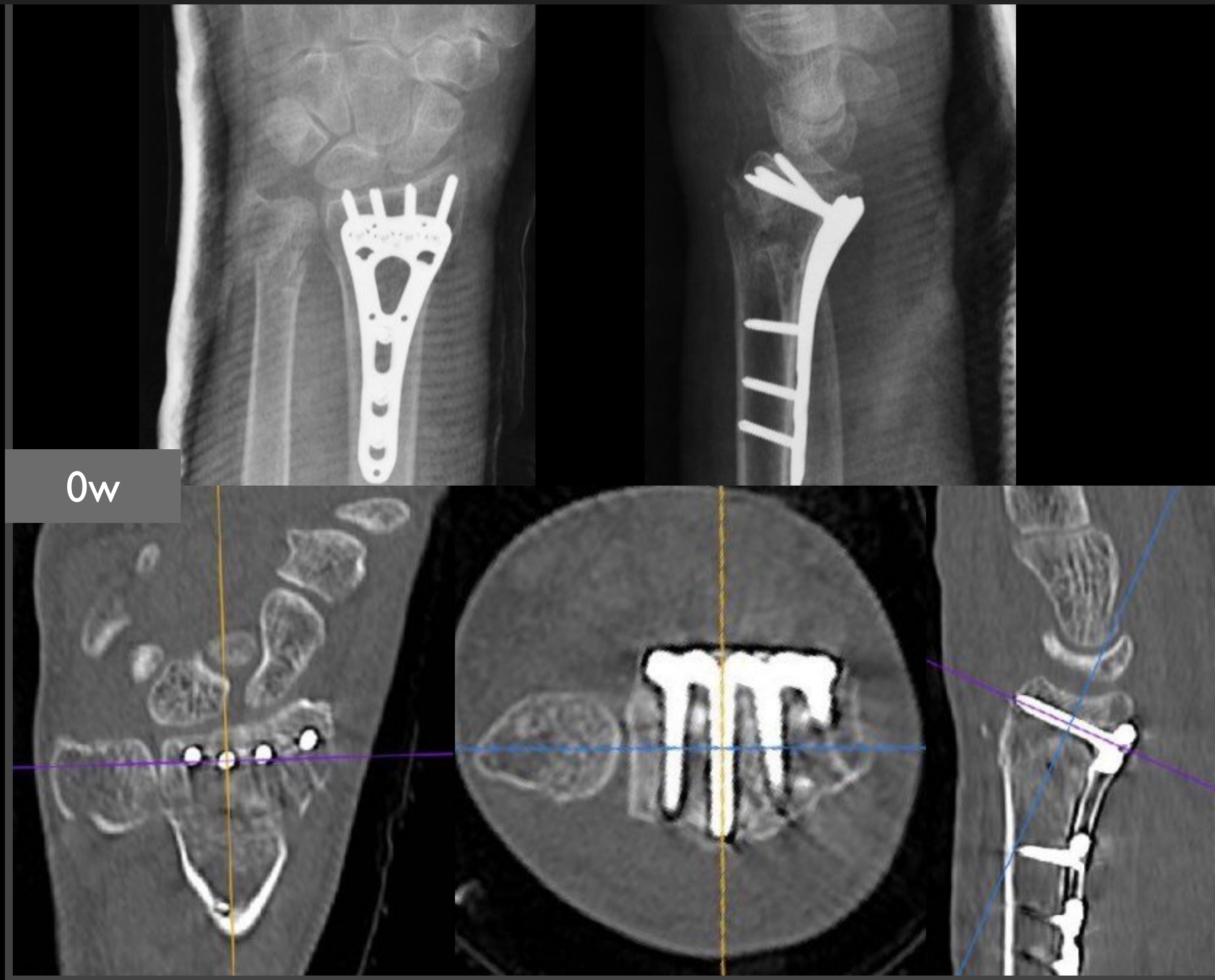

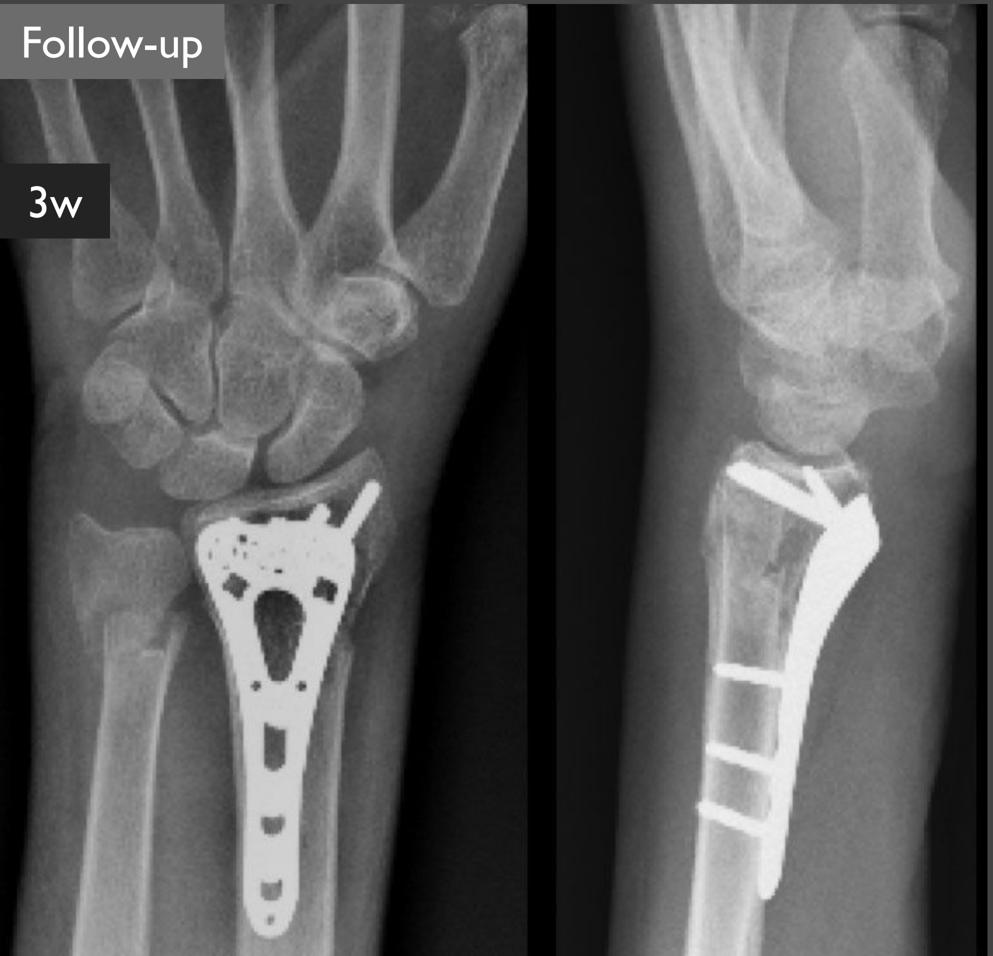

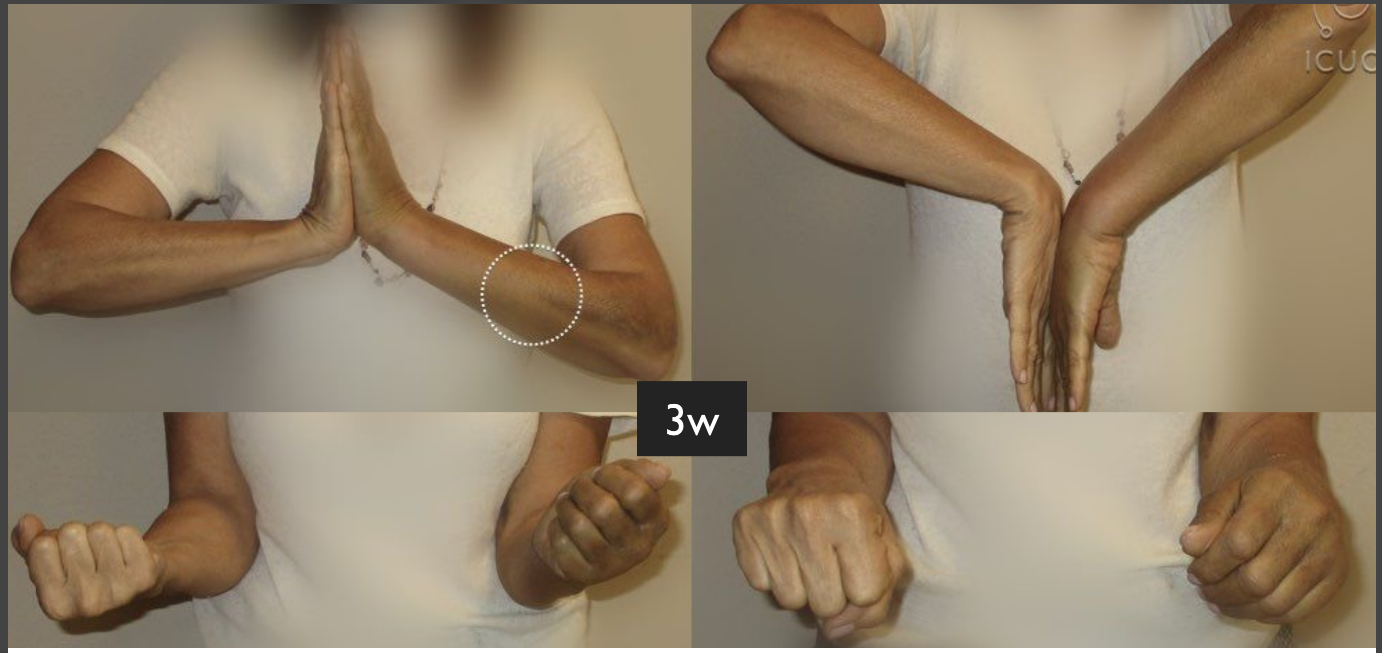

ICUC Score Functional Limitation: 2 (0-4) - Pain: 1 (0-4)

ICUC Score at 122w Functional limitation: 1 Pain: 0

Quick DASH = 9

ICUC® ID: 23-DC-826 / 55y Female

Complete case data can be accessed by clicking the ***ICUC ID***

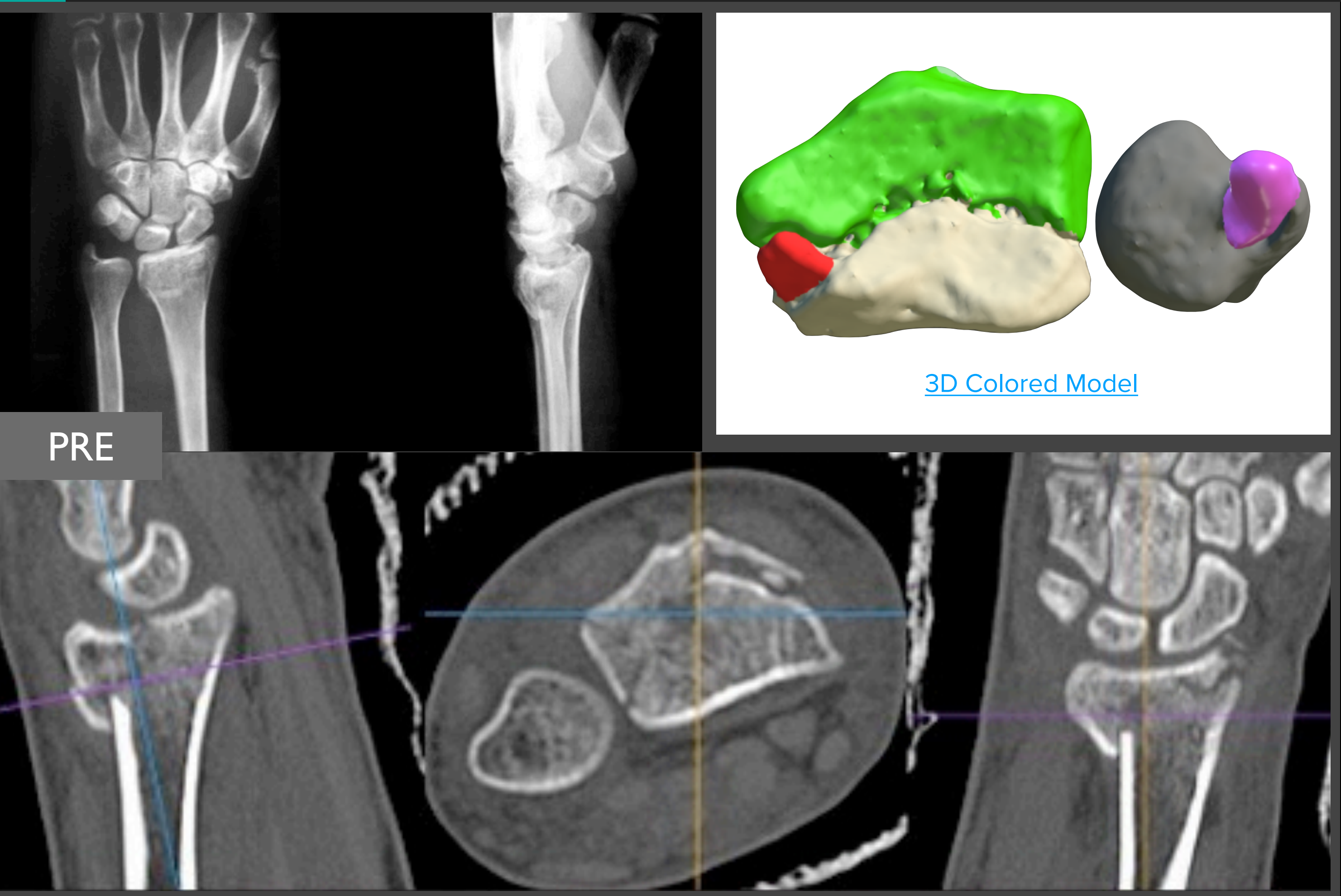

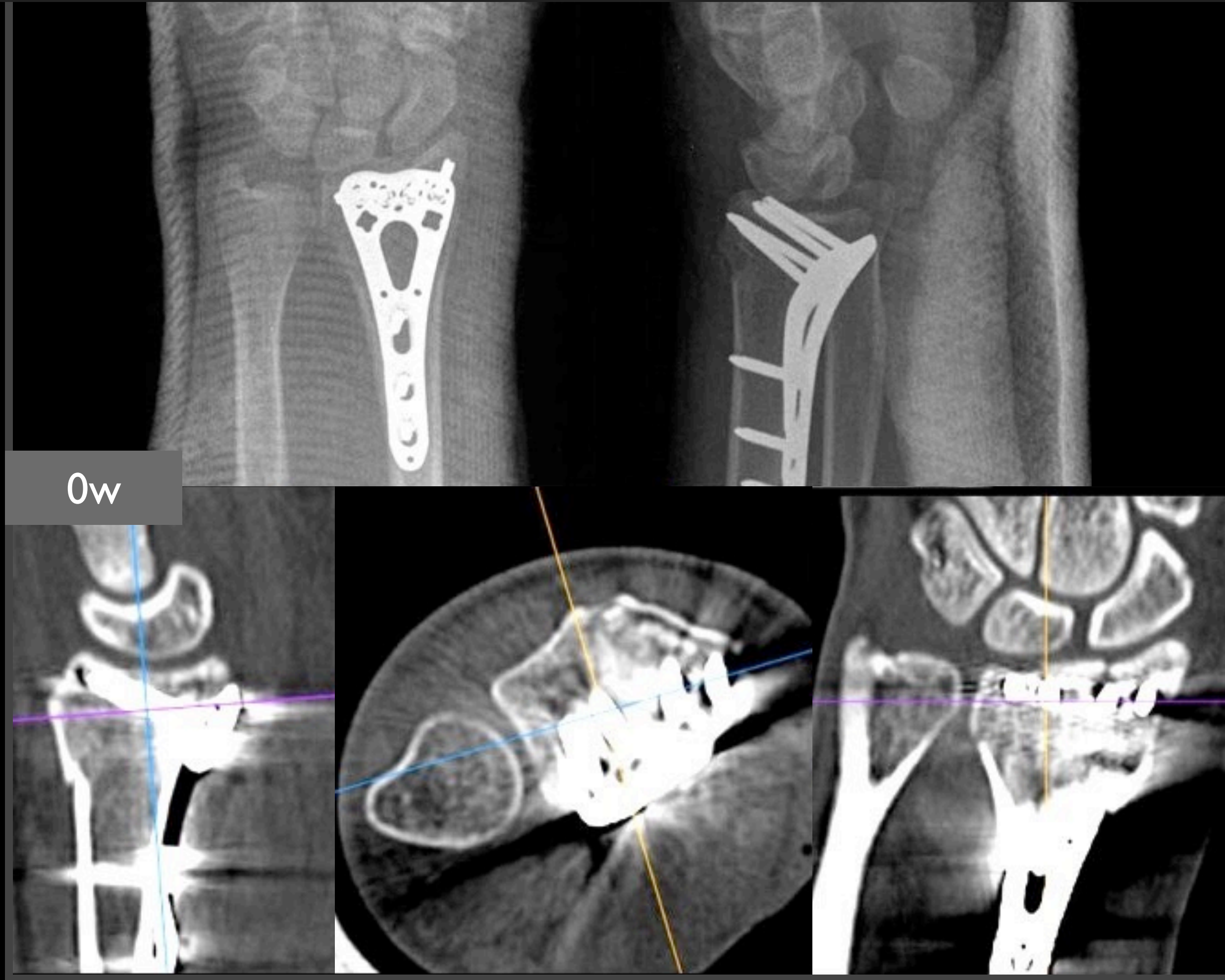

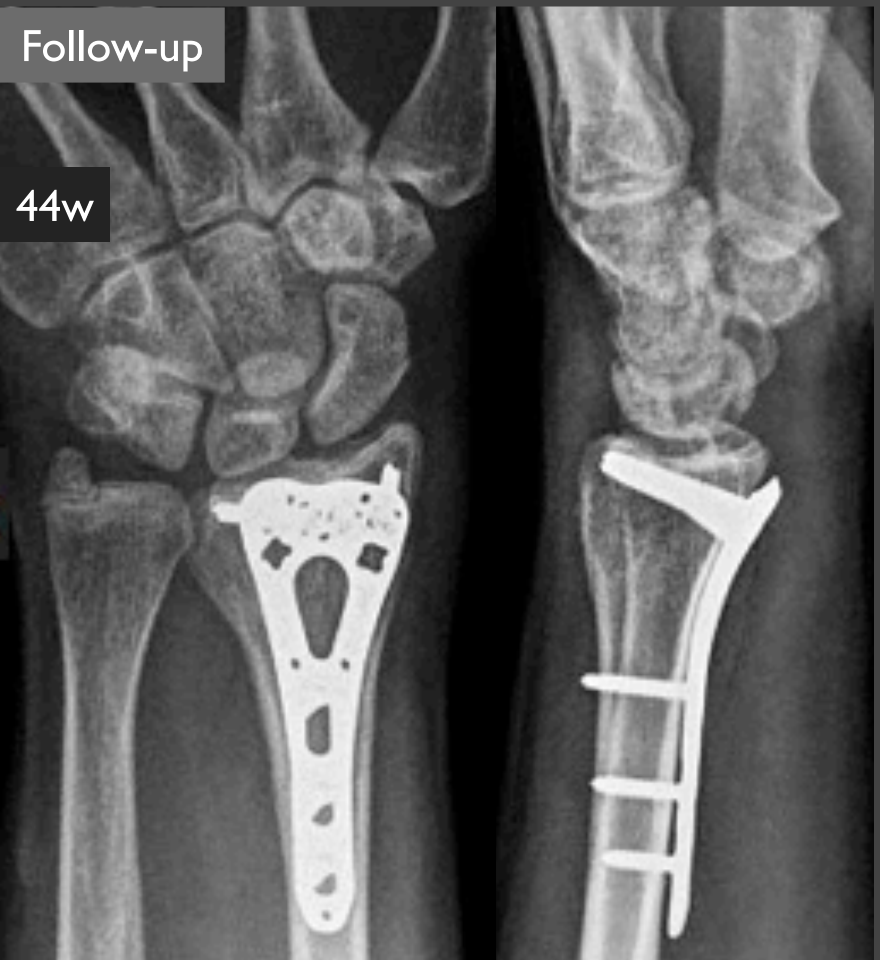

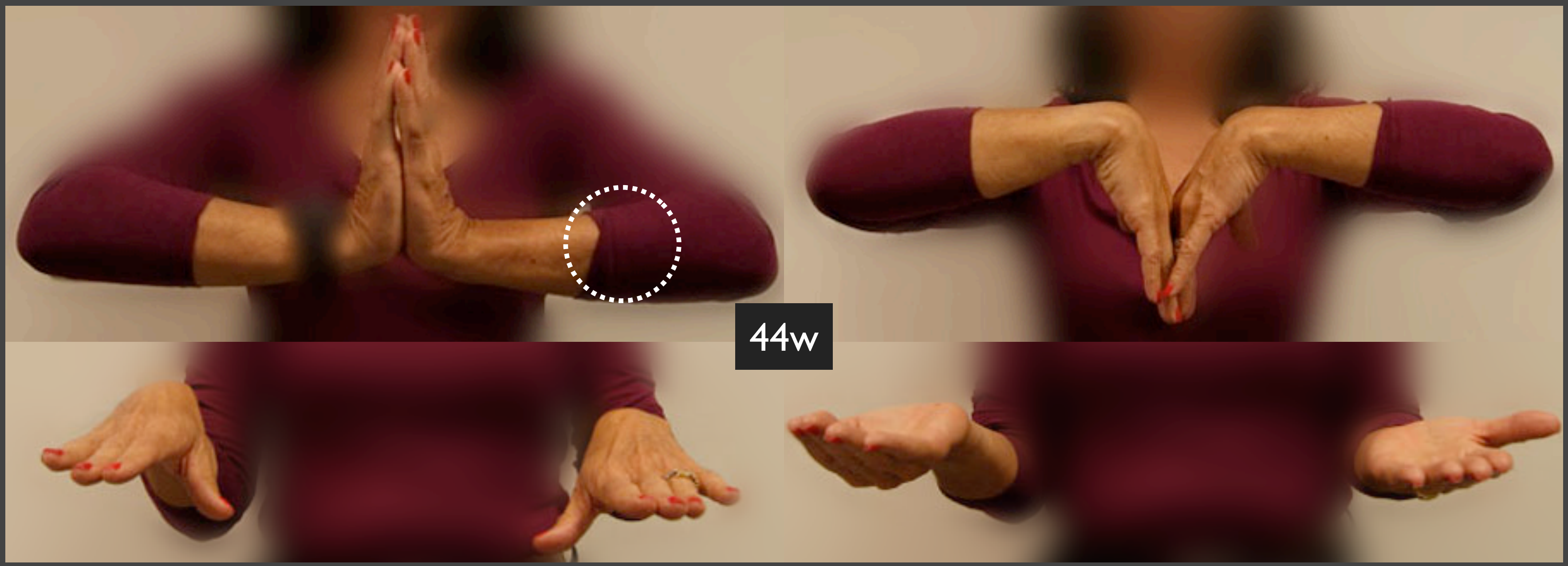

ICUC Score at 581w Functional limitation: 0 Pain: 0

Quick DASH = 0

ICUC® ID: 23-DC-654 / 40y Male

*Complete case data can be accessed by clicking the **ICUC ID***

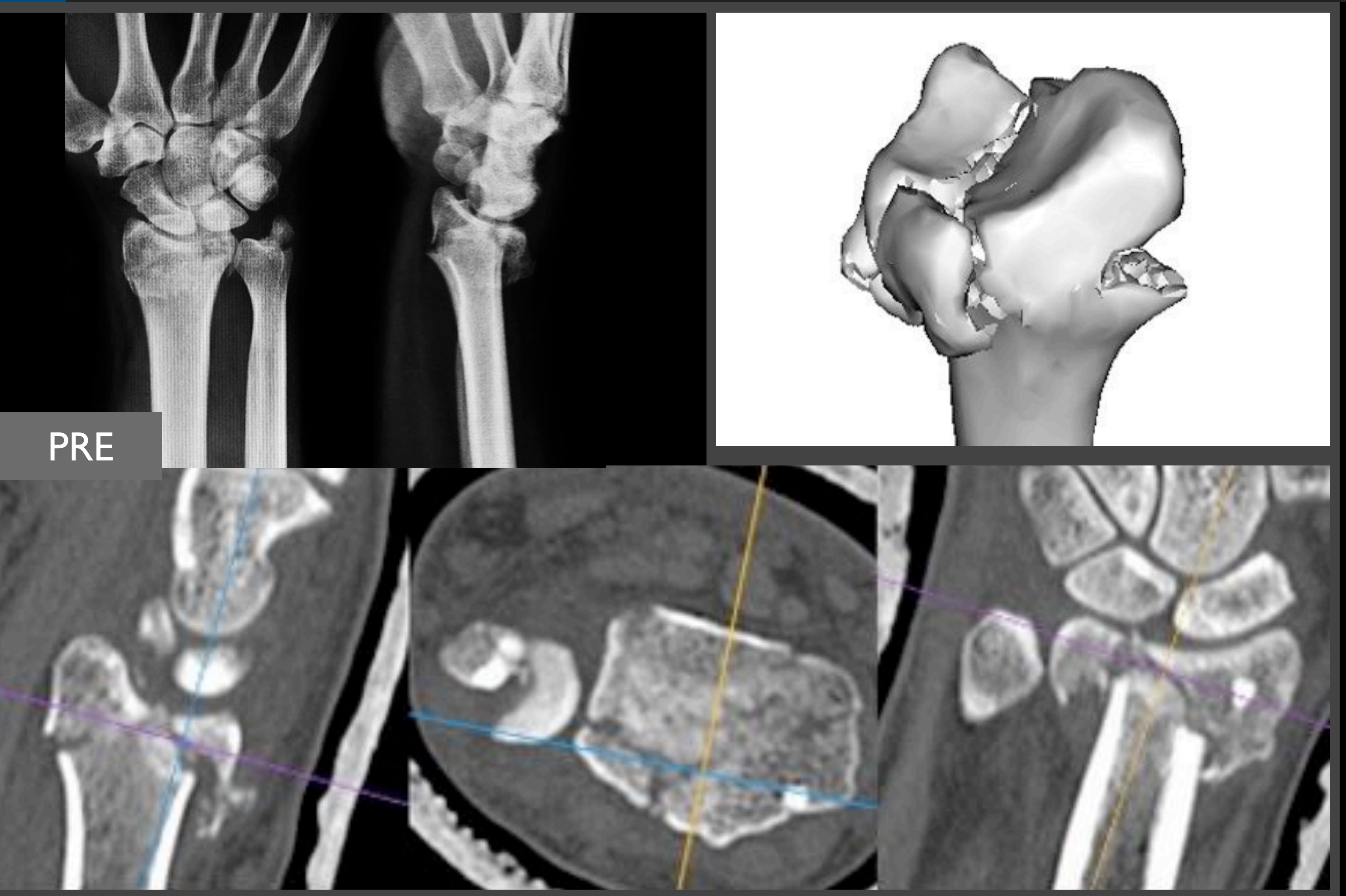

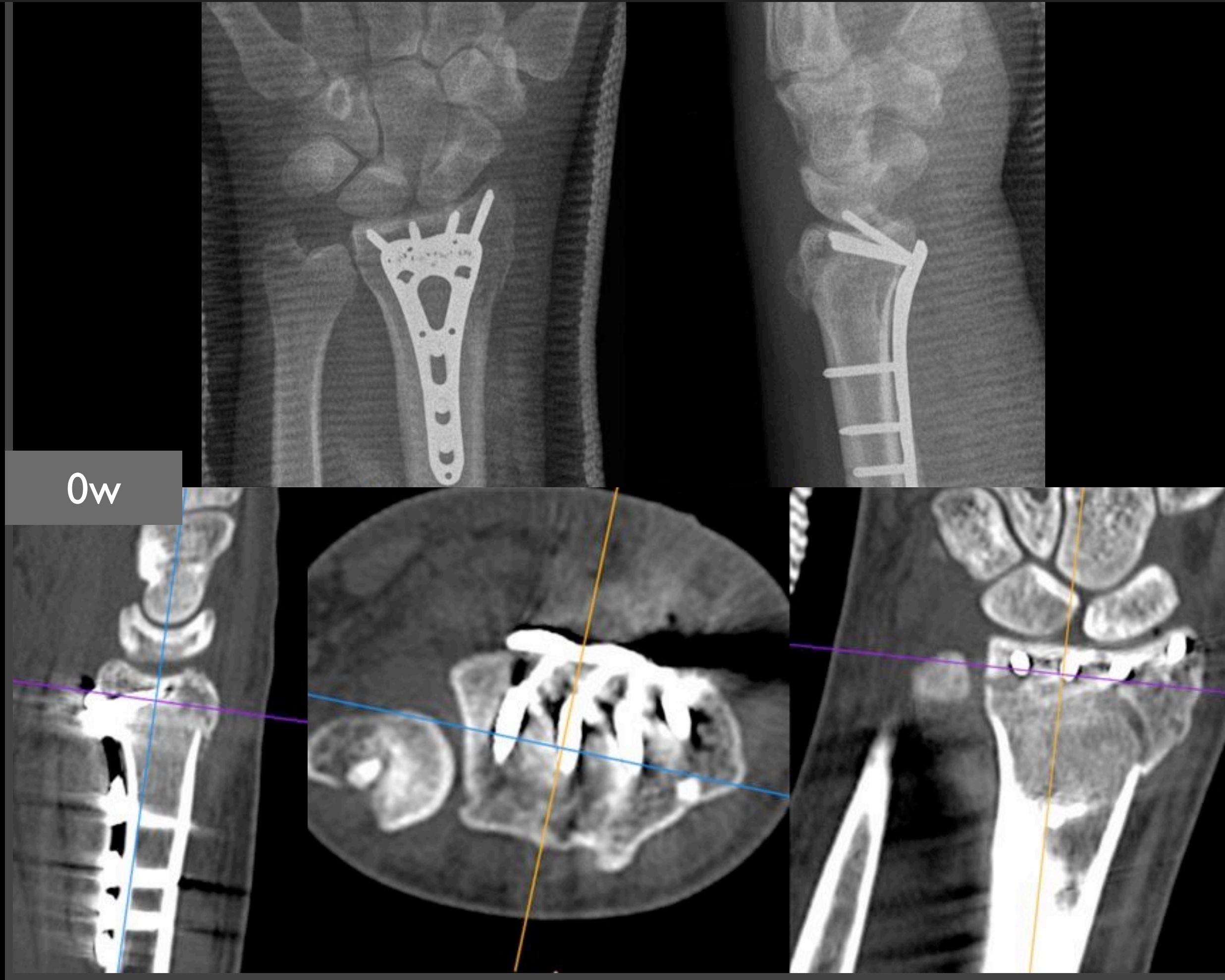

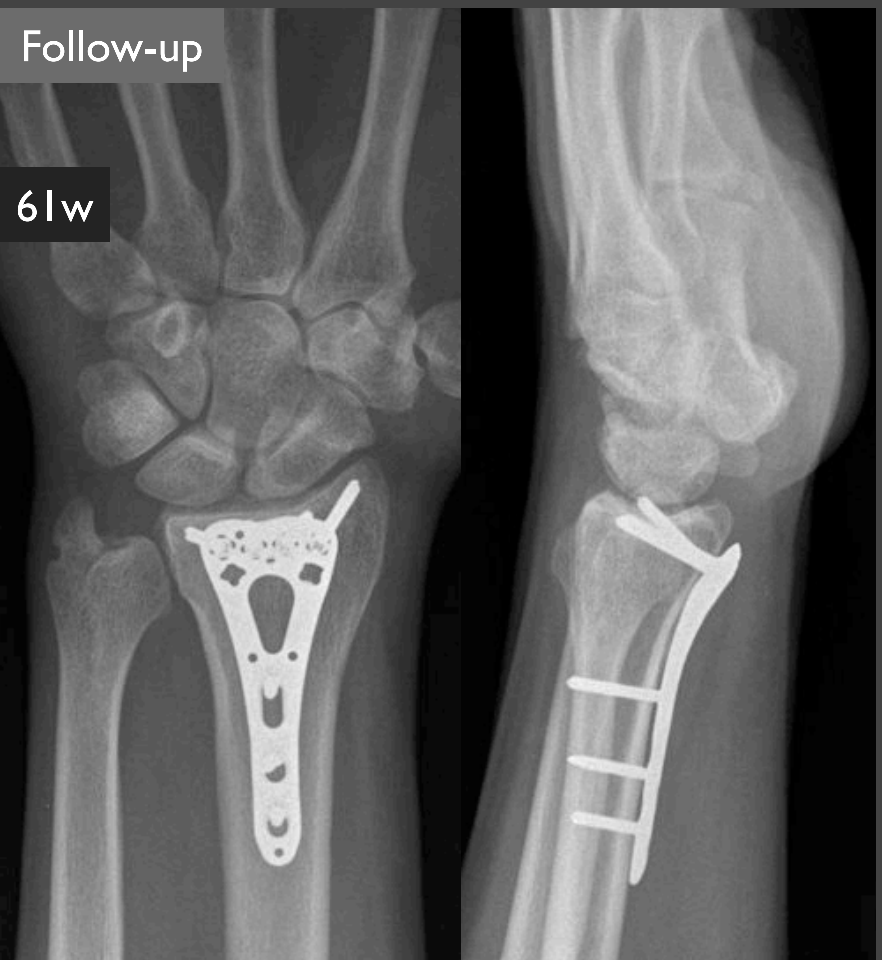

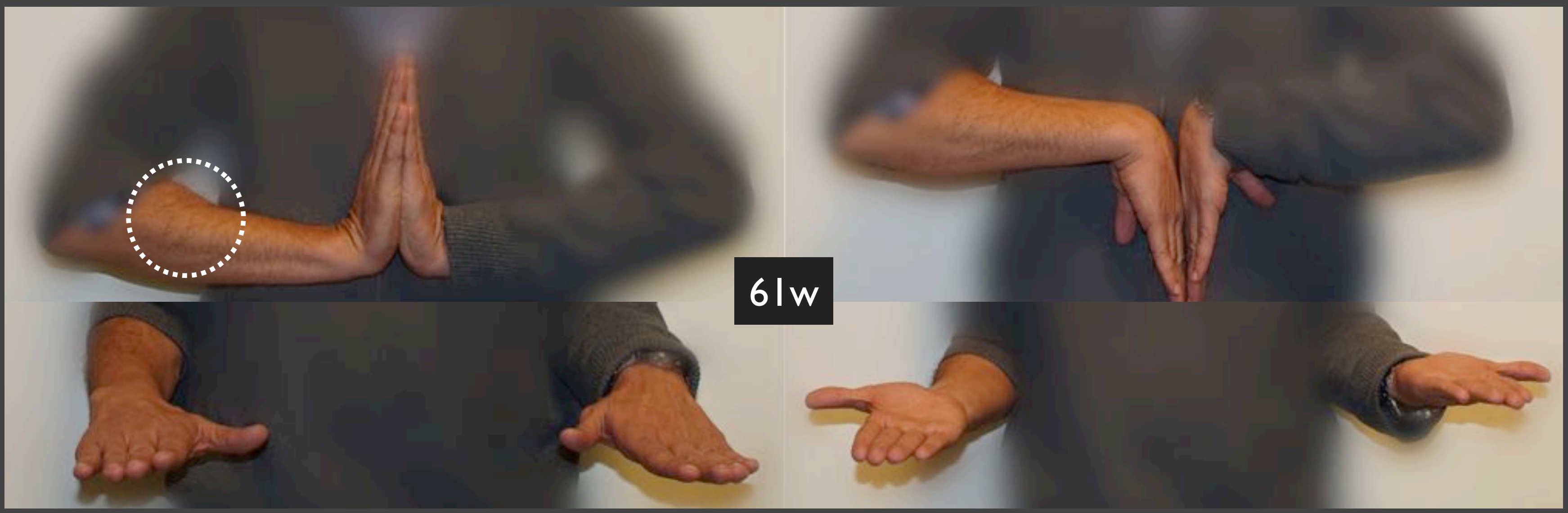

ICUC Score at 595w Functional limitation: 0 Pain: 1

Quick DASH = 5

ICUC® ID: 23-DC-572 / 55y Female

Complete case data can be accessed by clicking the ***ICUC ID***

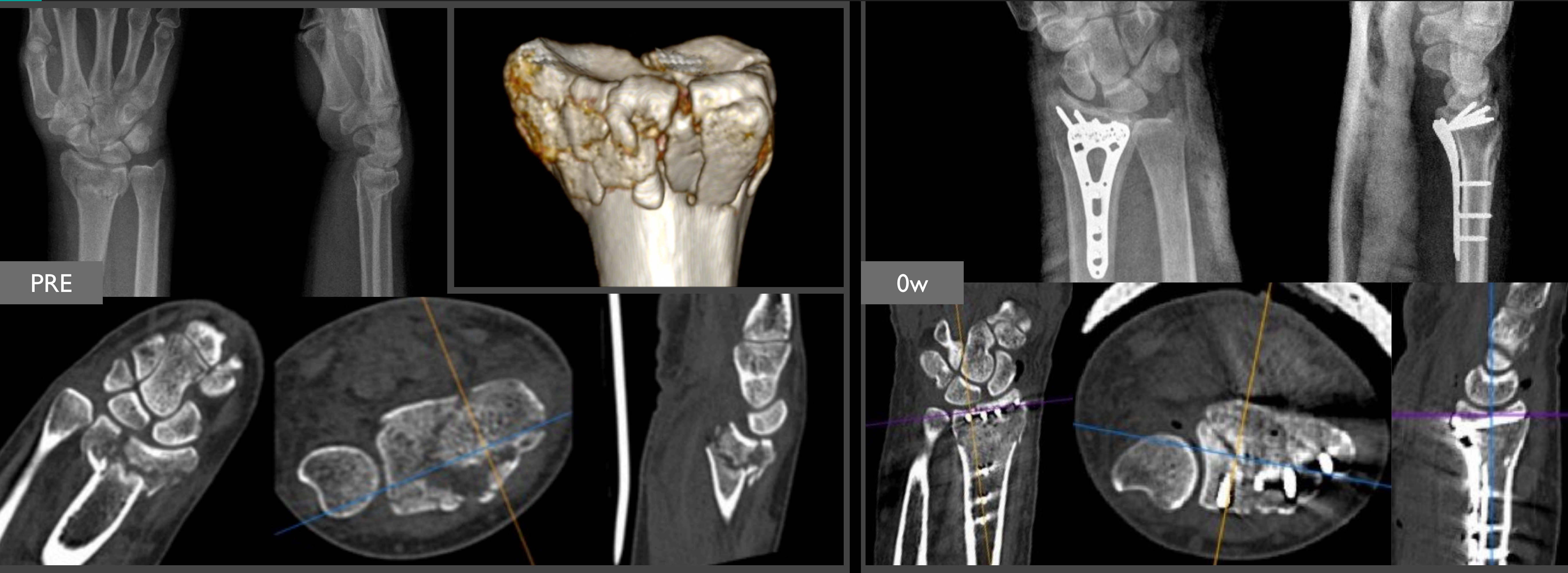

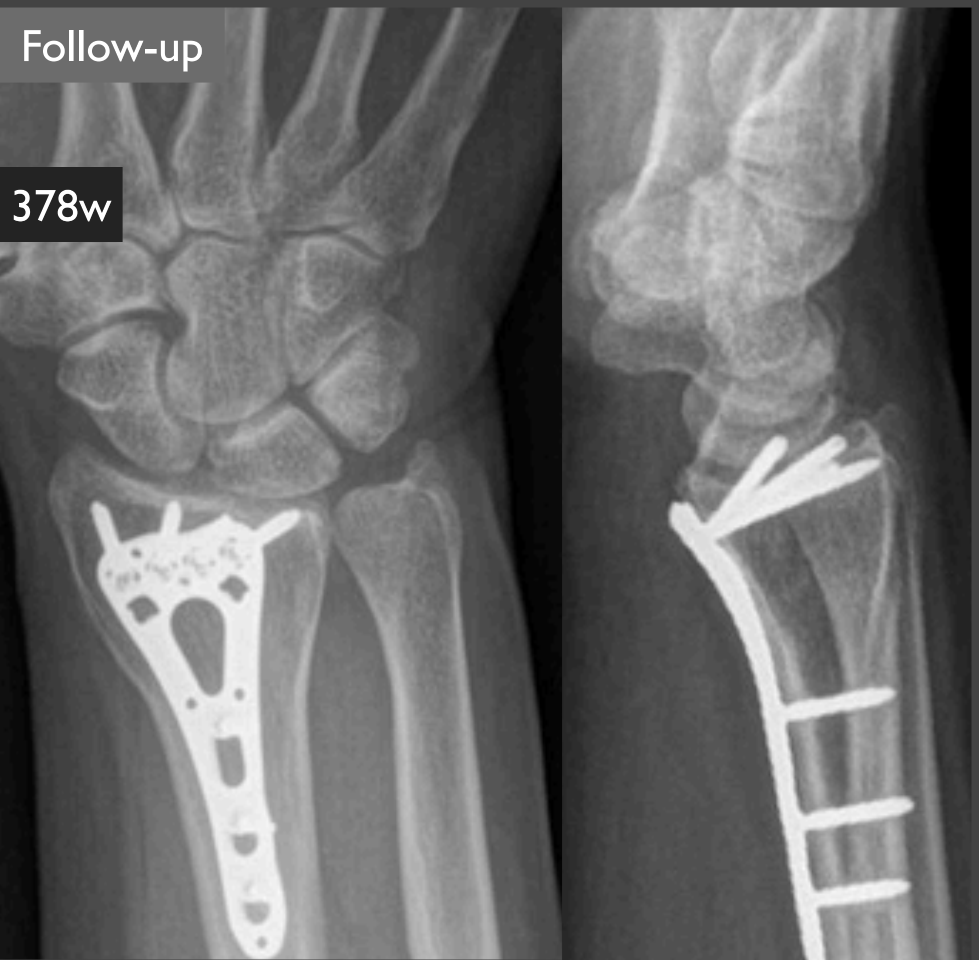

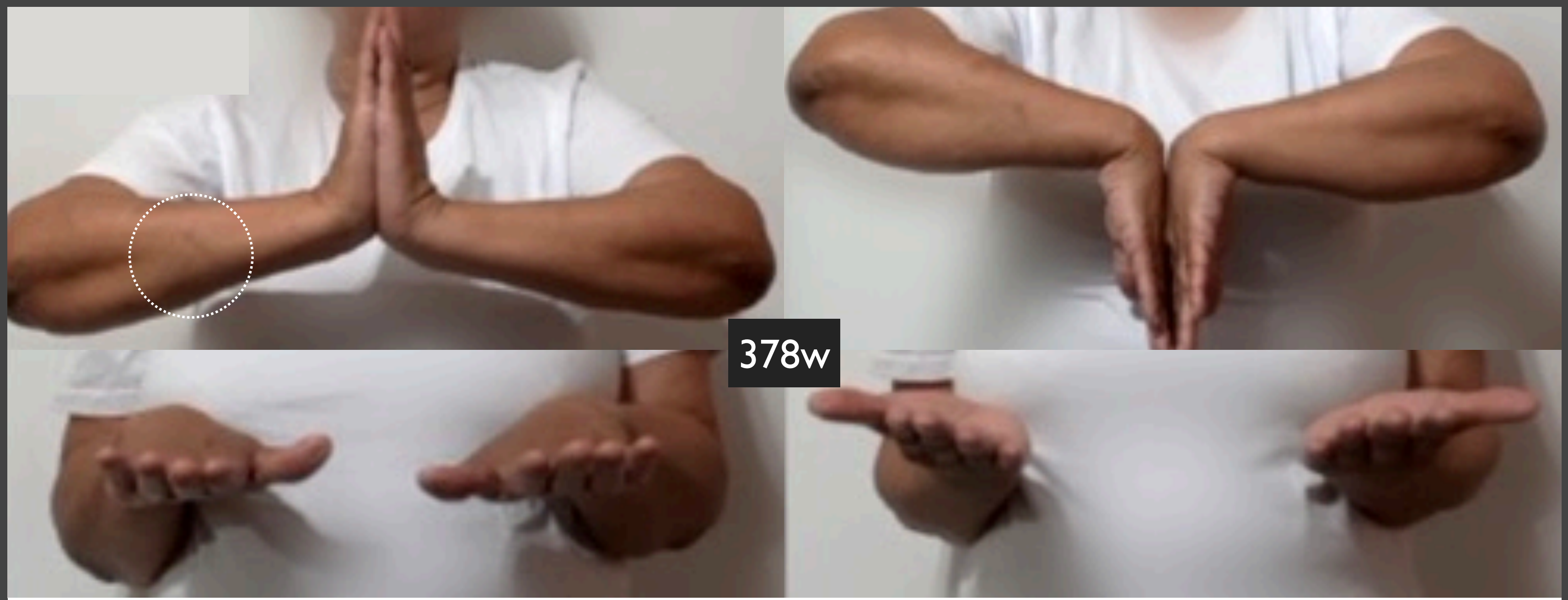

ICUC Score ***Functional Limitation:*** **0** (0-4) - ***Pain:*** **0** (0-4)

Quick DASH = 2

ICUC® ID: 23-DC-675 / 65y Female

Complete case data can be accessed by clicking the ***ICUC ID***

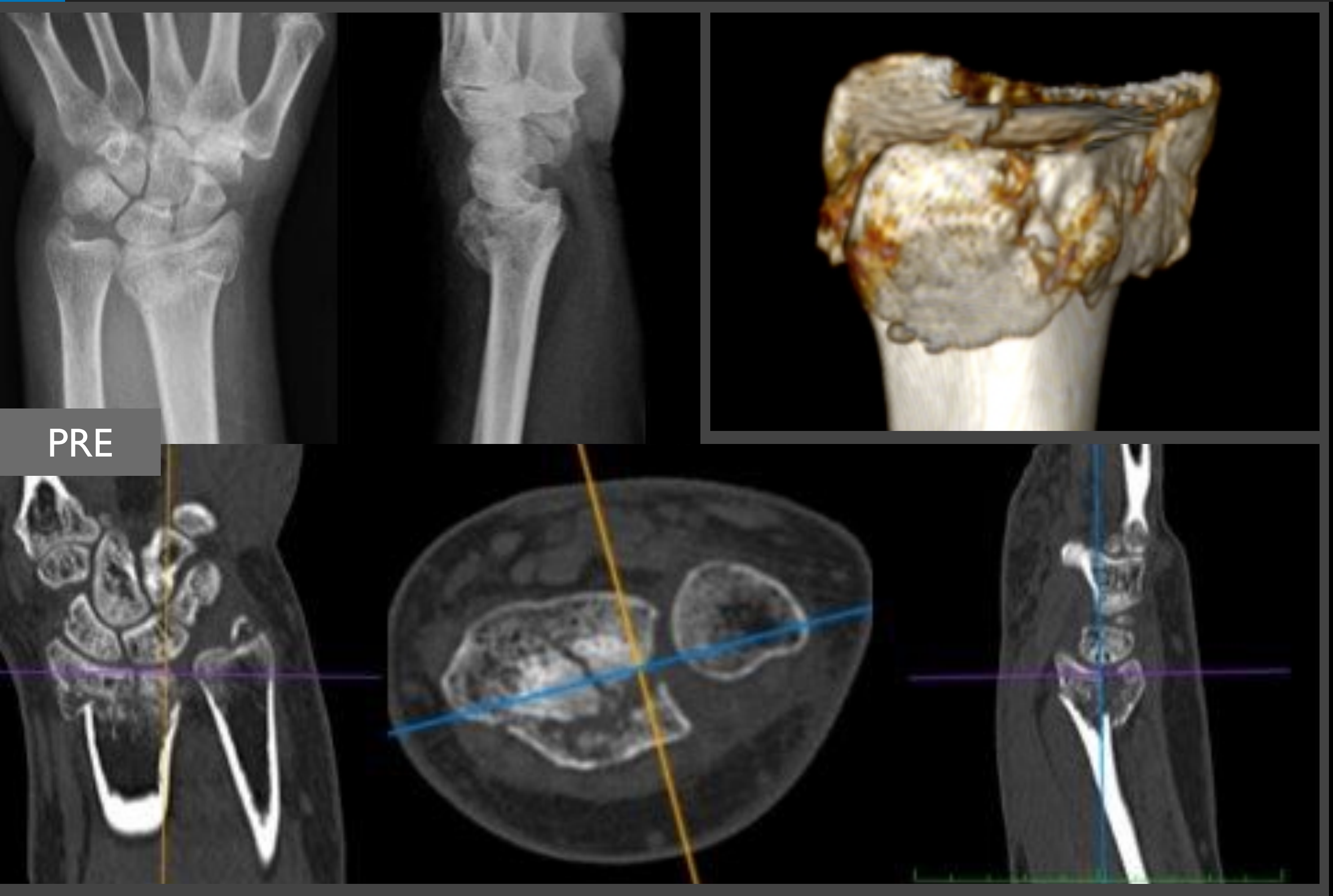

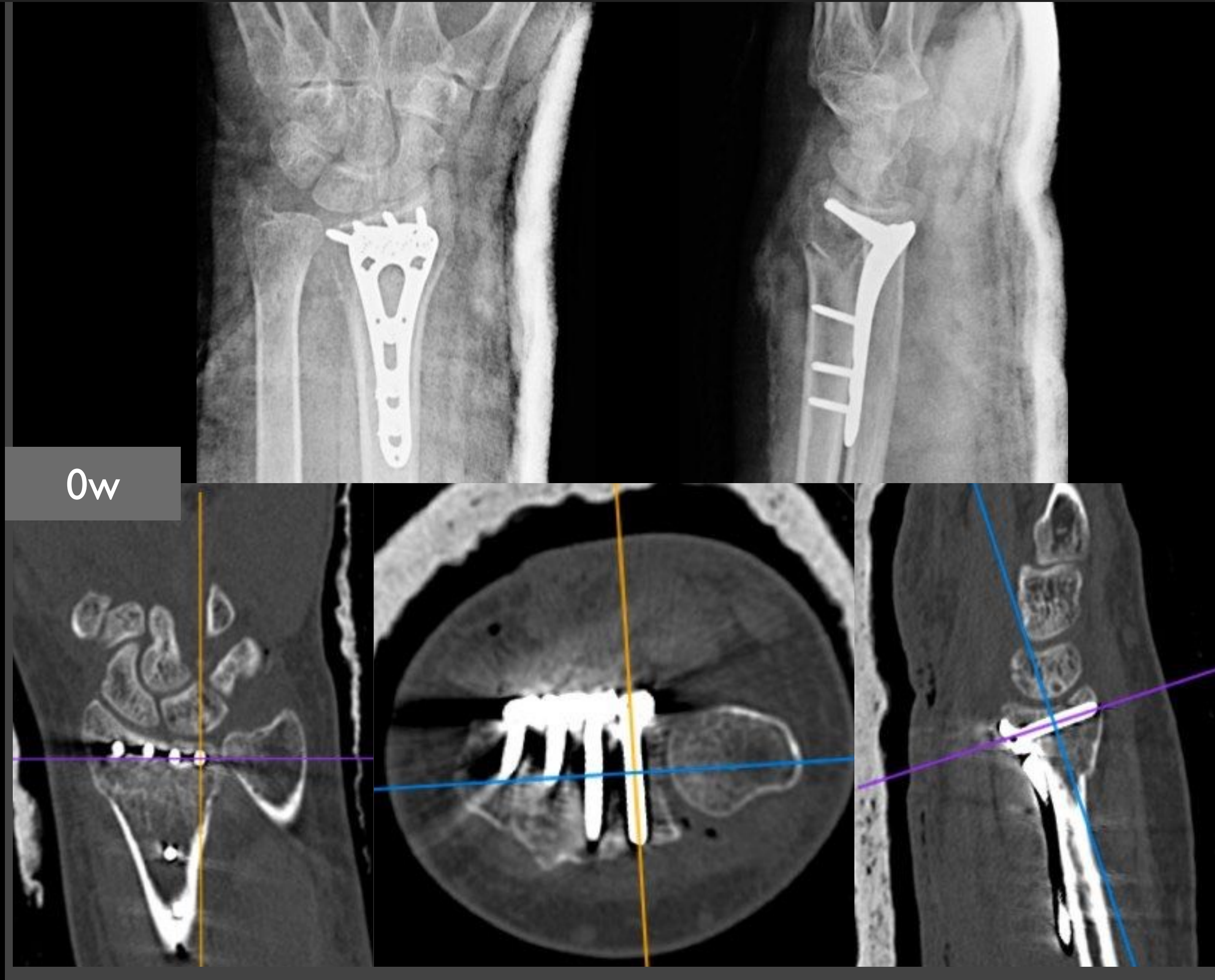

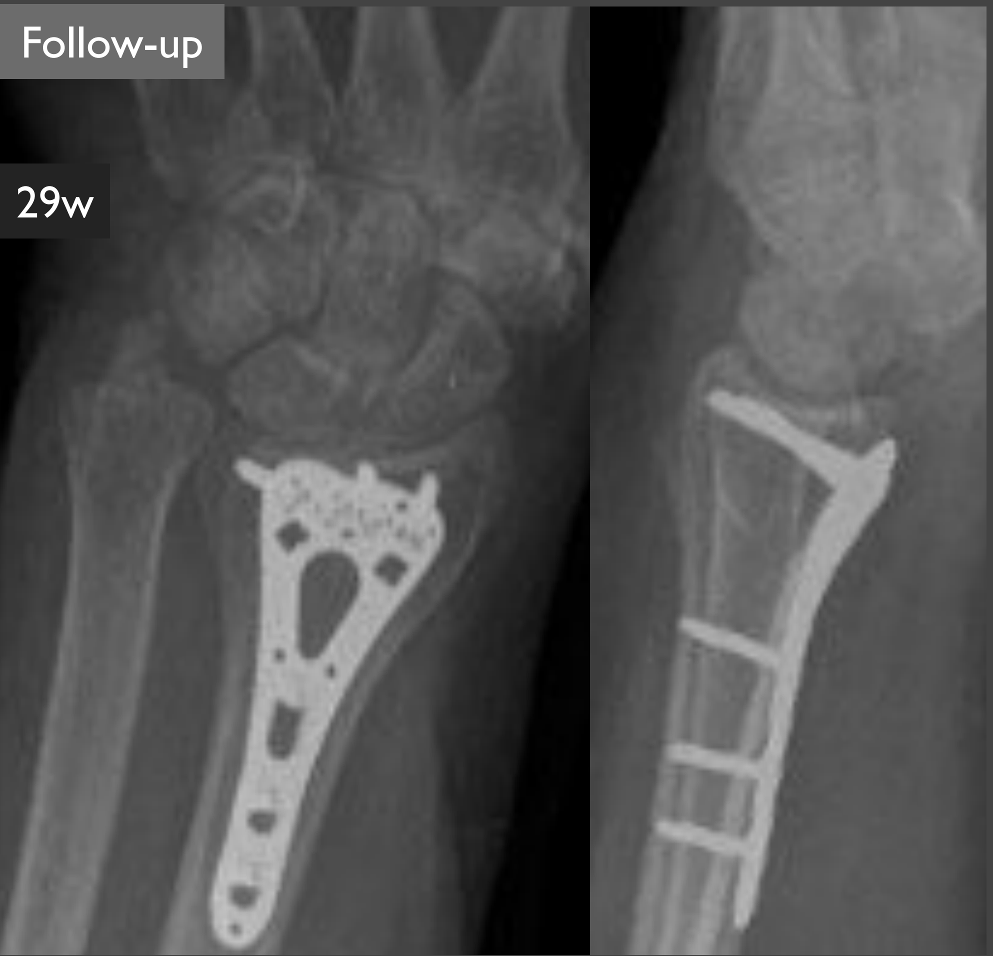

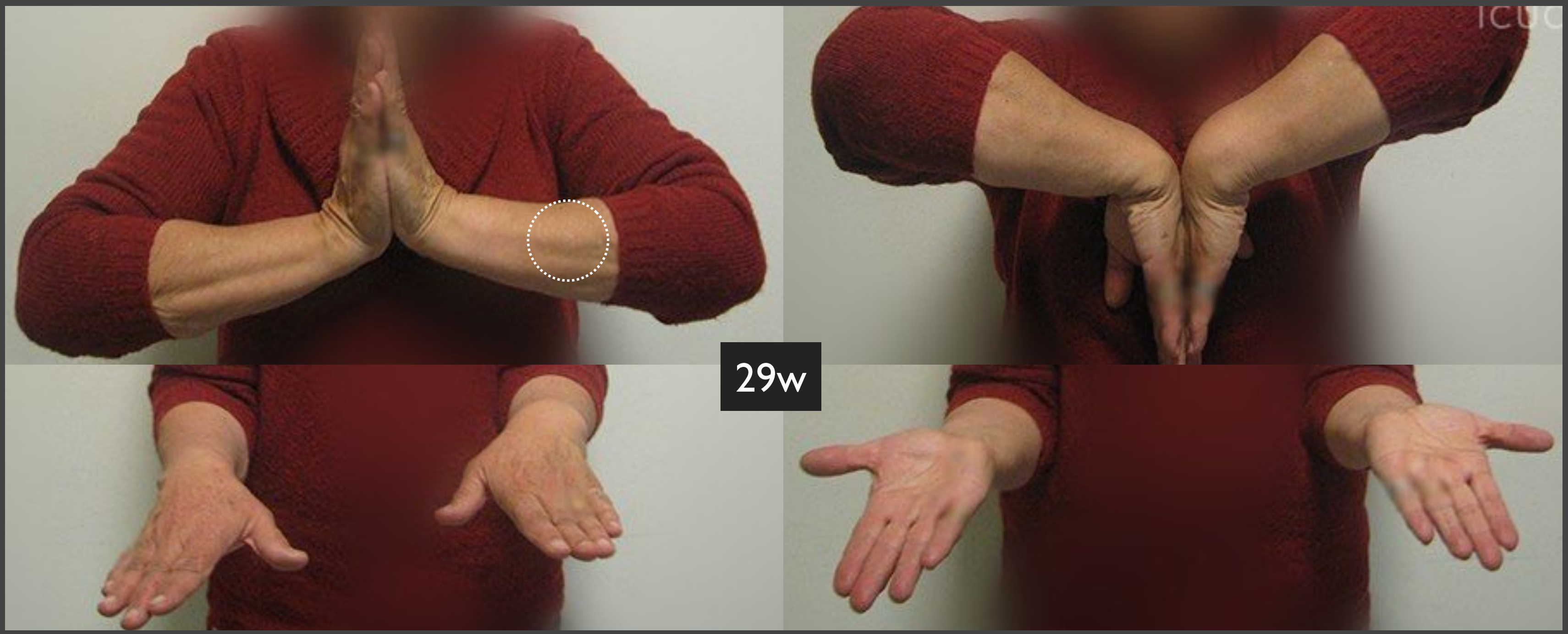

ICUC Score at 447w Functional limitation: 0 Pain: 0

Quick DASH = 0

ICUC® ID: 23-DC-591 / 30y Female

Complete case data can be accessed by clicking the ***ICUC ID***

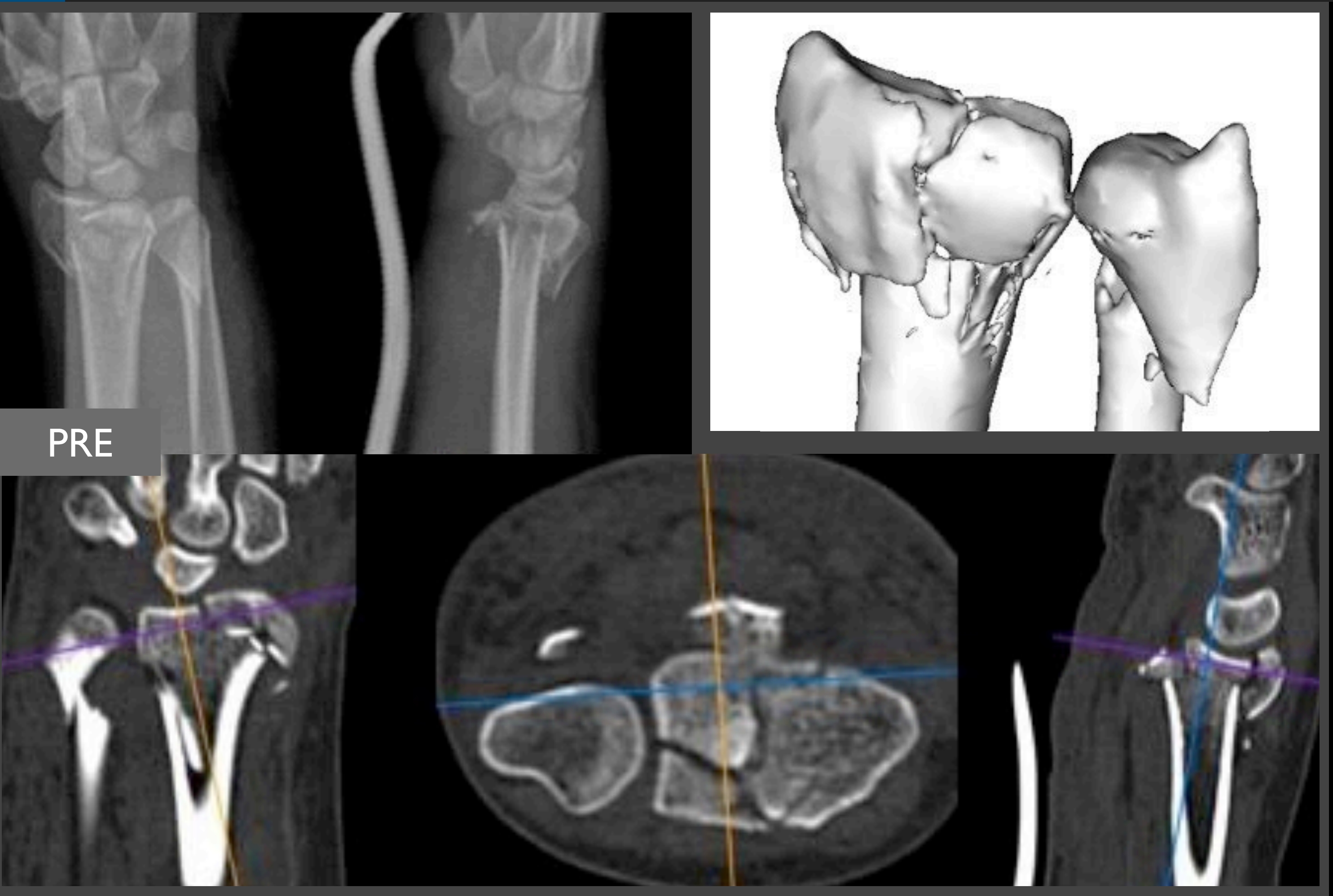

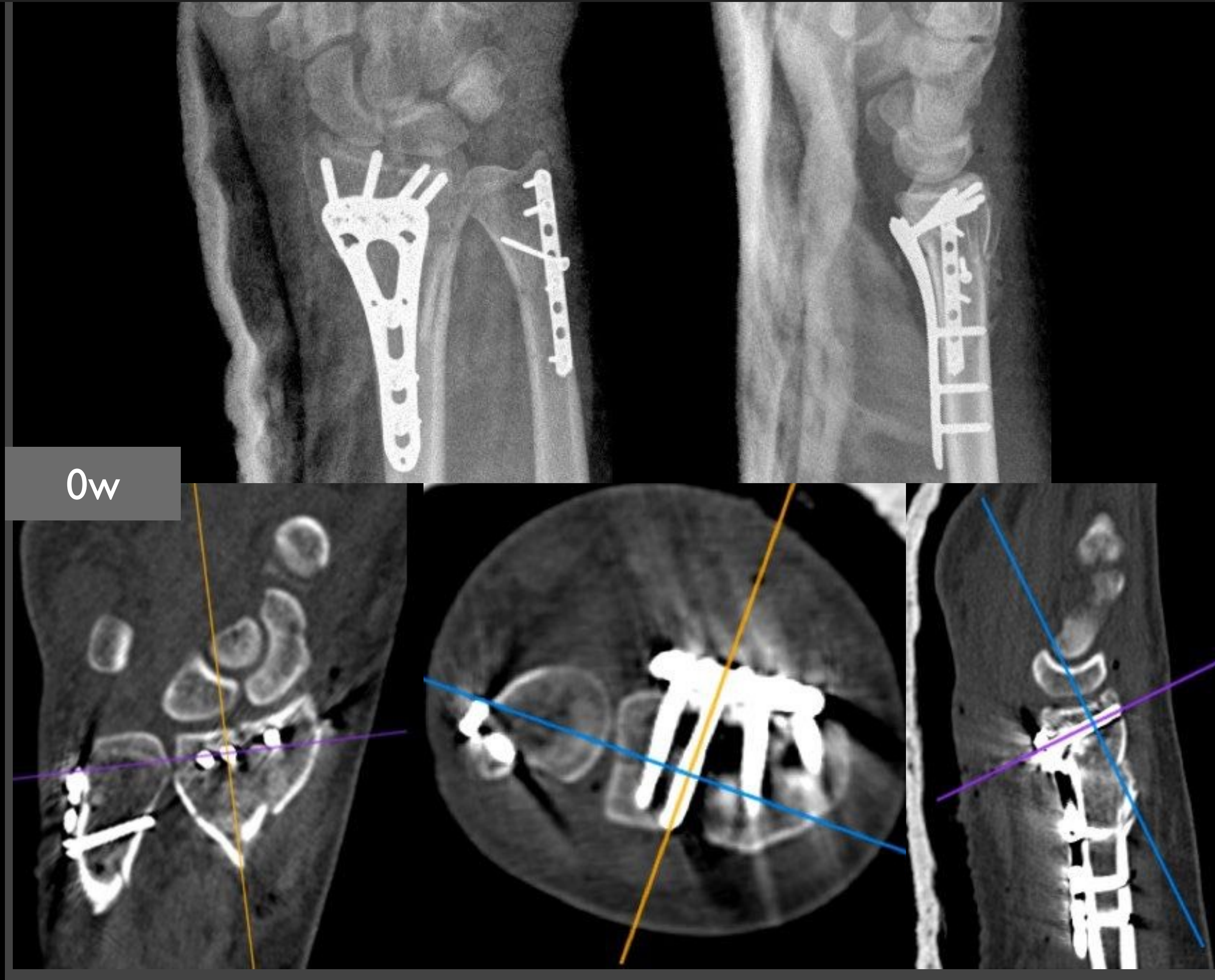

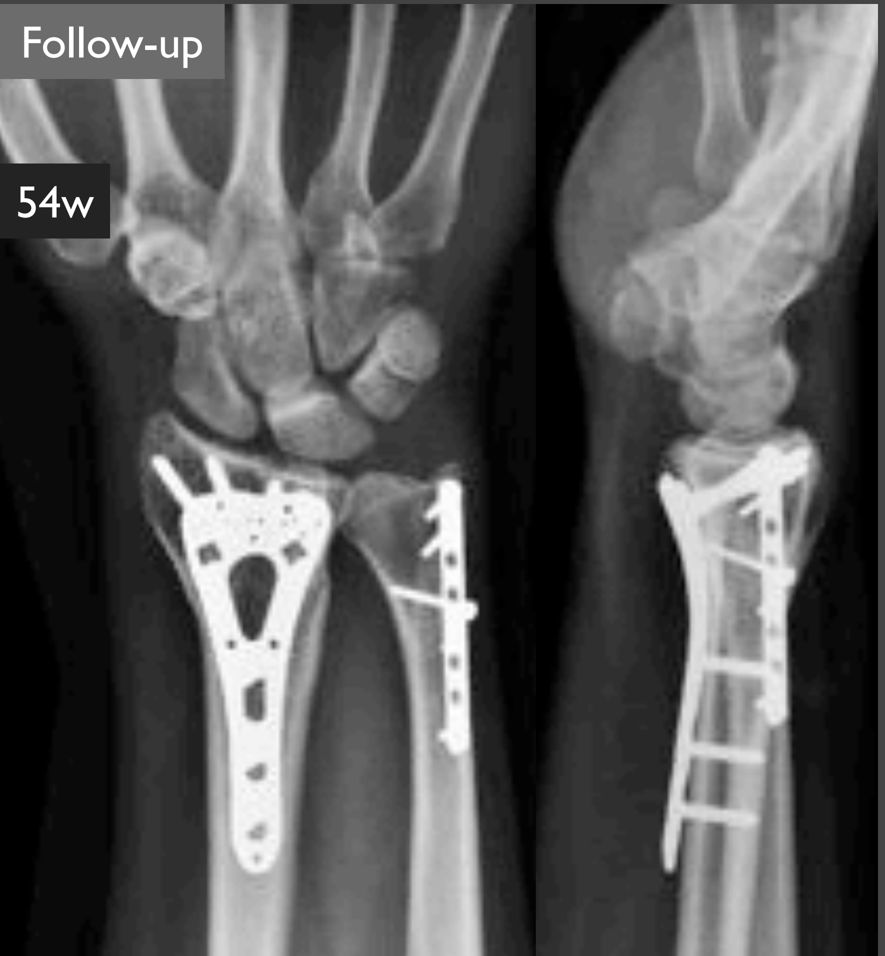

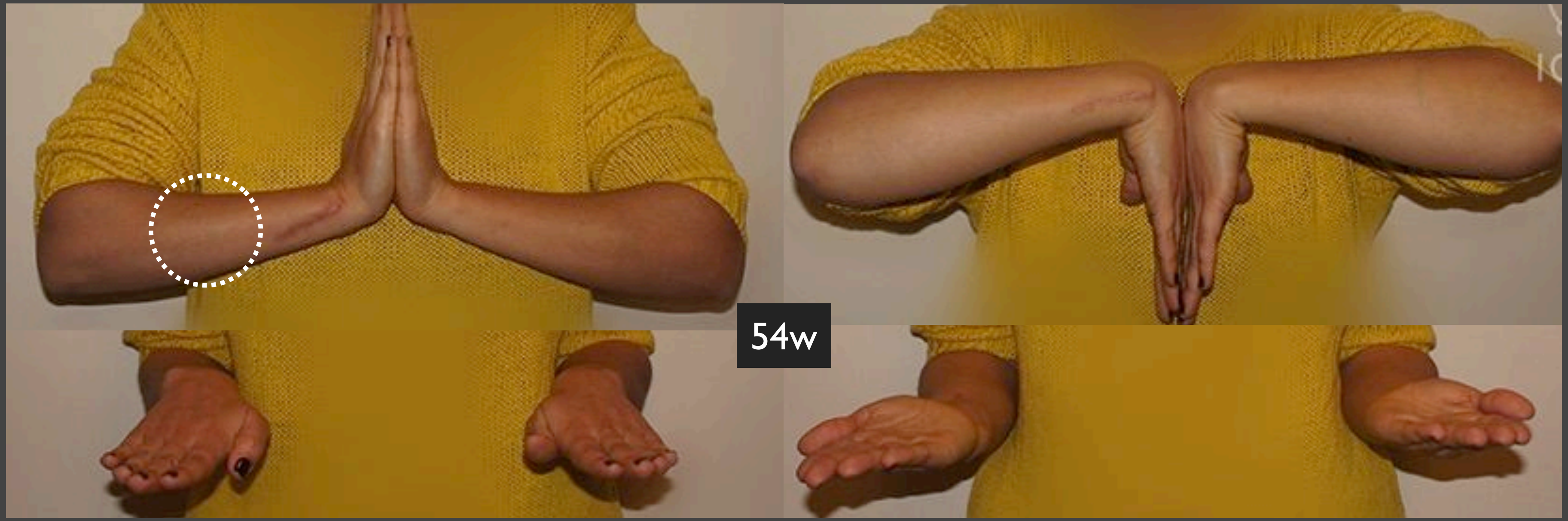

ICUC Score at 147w Functional limitation: 1 Pain: 1

Quick DASH = 11

ICUC® ID: 23-DC-310 / 65y Female

Complete case data can be accessed by clicking the ***ICUC ID***

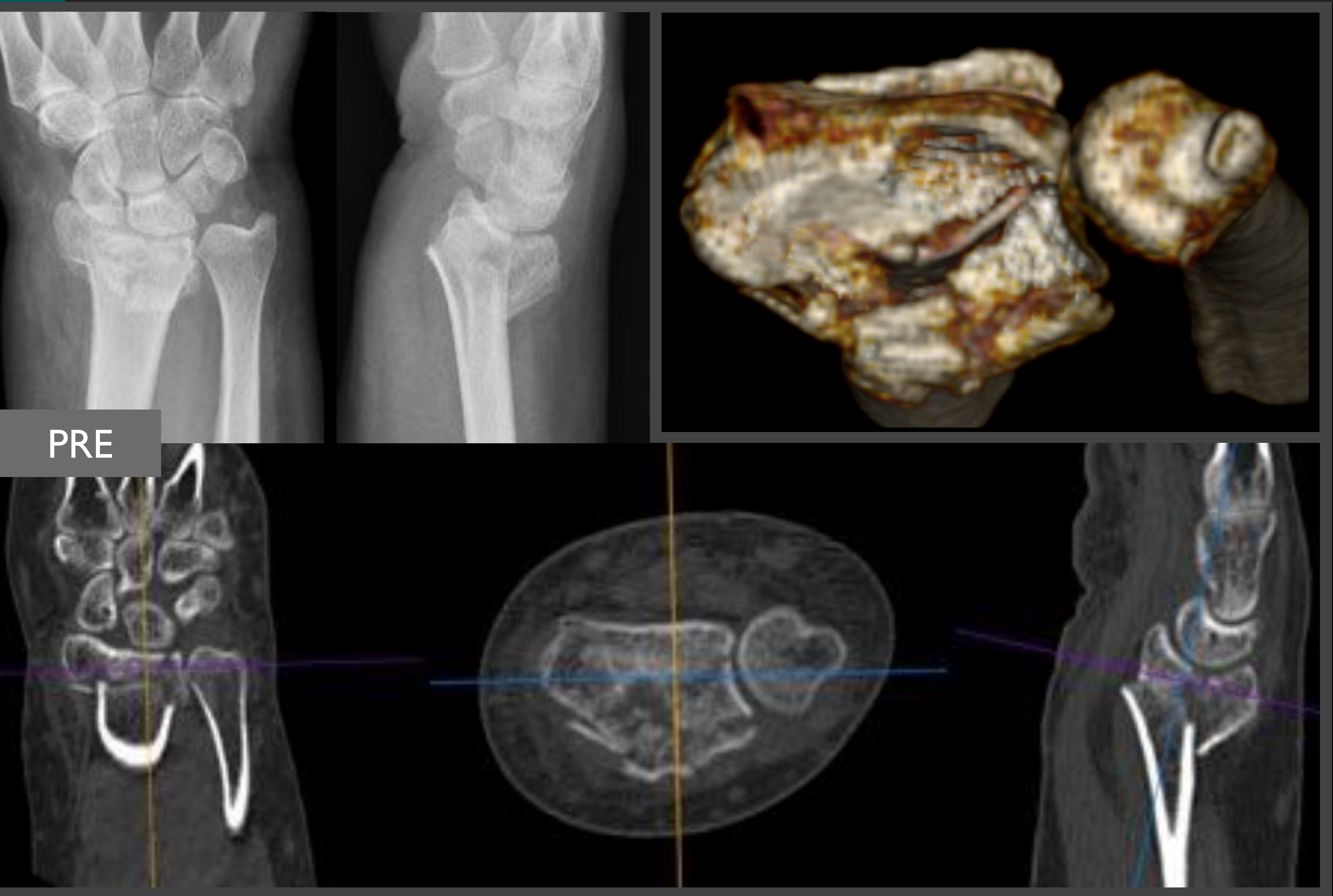

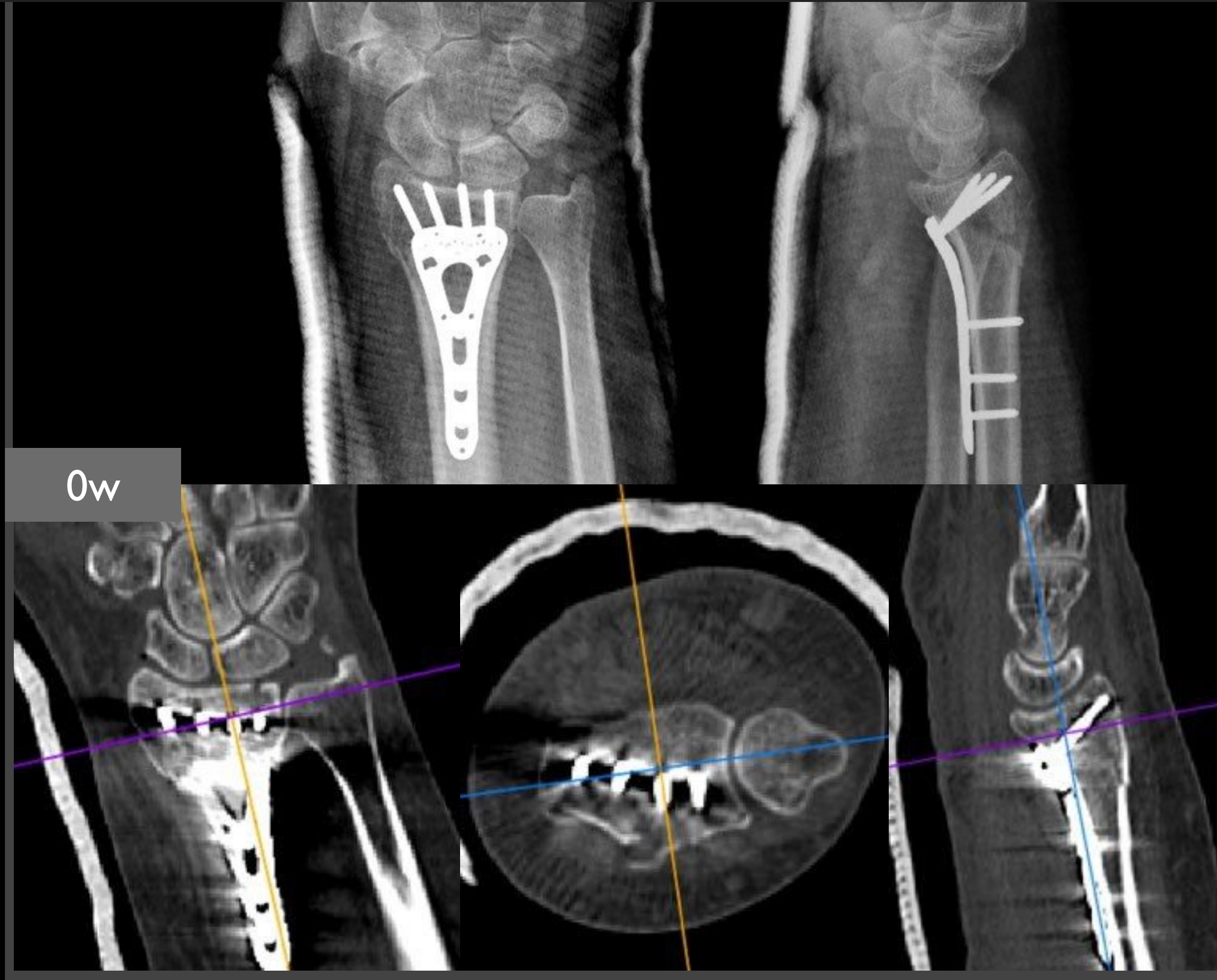

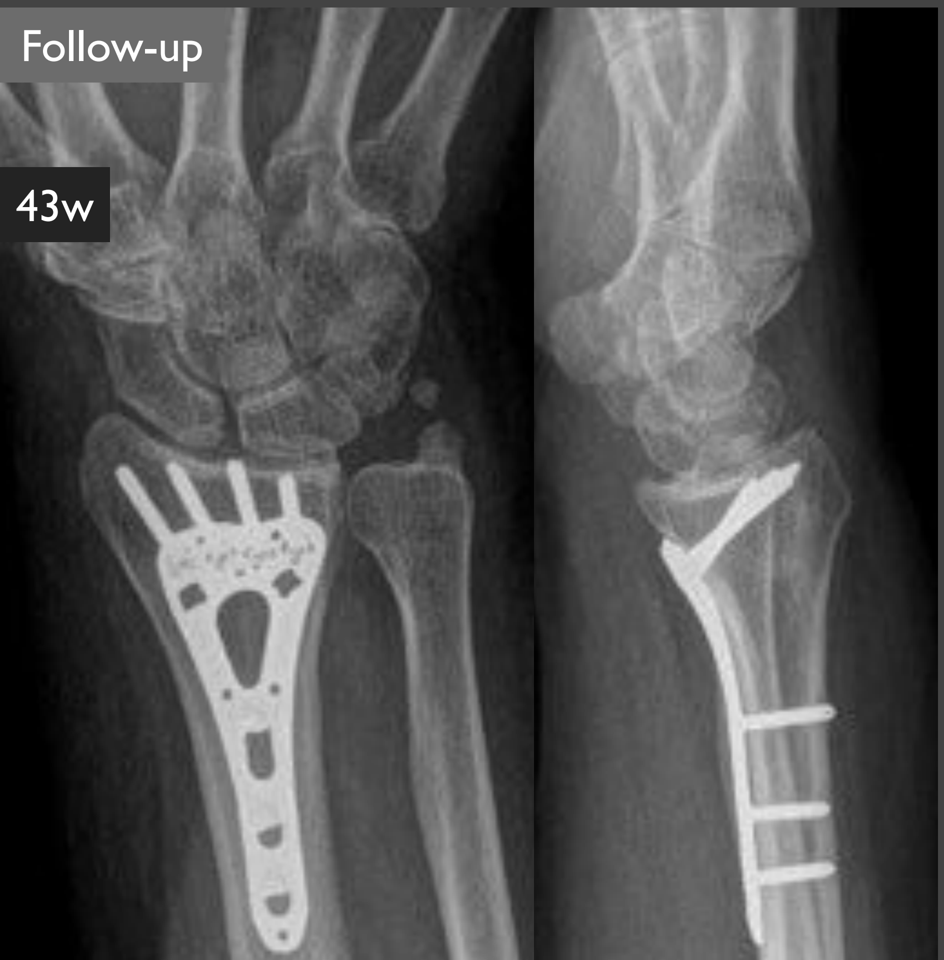

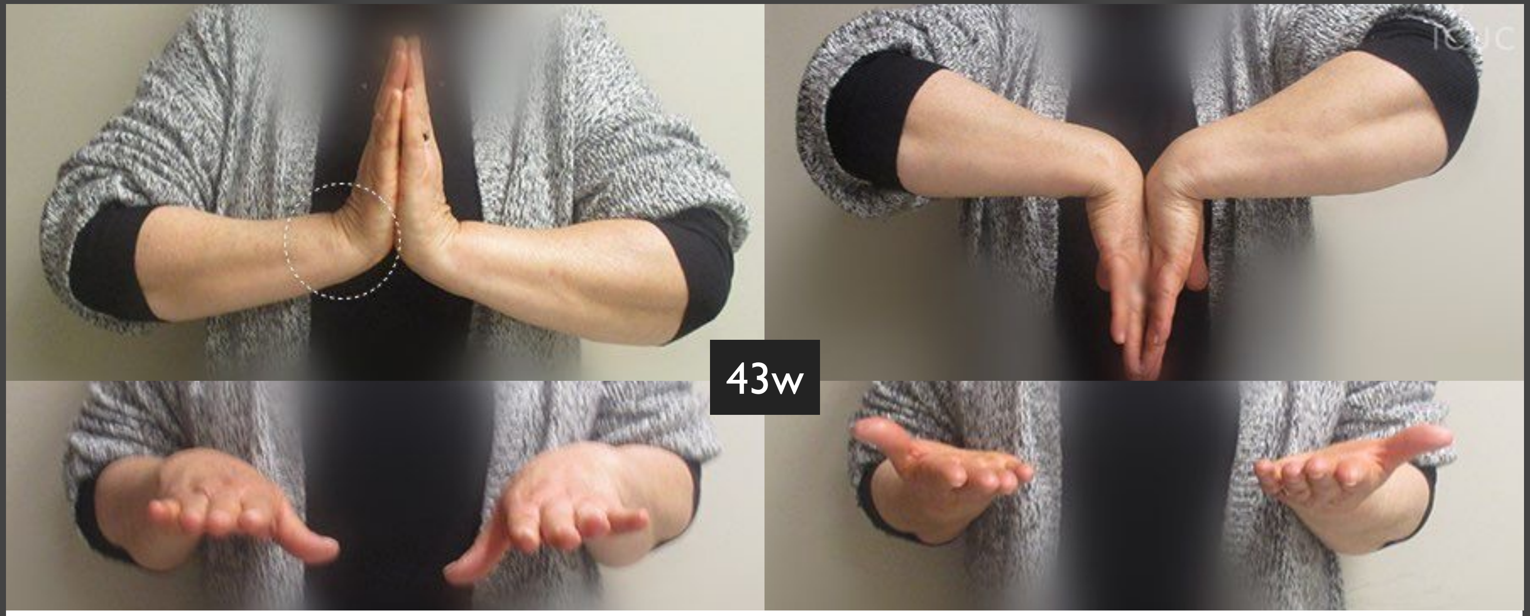

ICUC Score Functional Limitation: 1 (0-4) - Pain: 2 (0-4)

ICUC Score at 361w Functional limitation: 0 Pain: 0

Quick DASH = 7

ICUC® ID: 23-DC-075 / 35y Female

Complete case data can be accessed by clicking the ***ICUC ID***

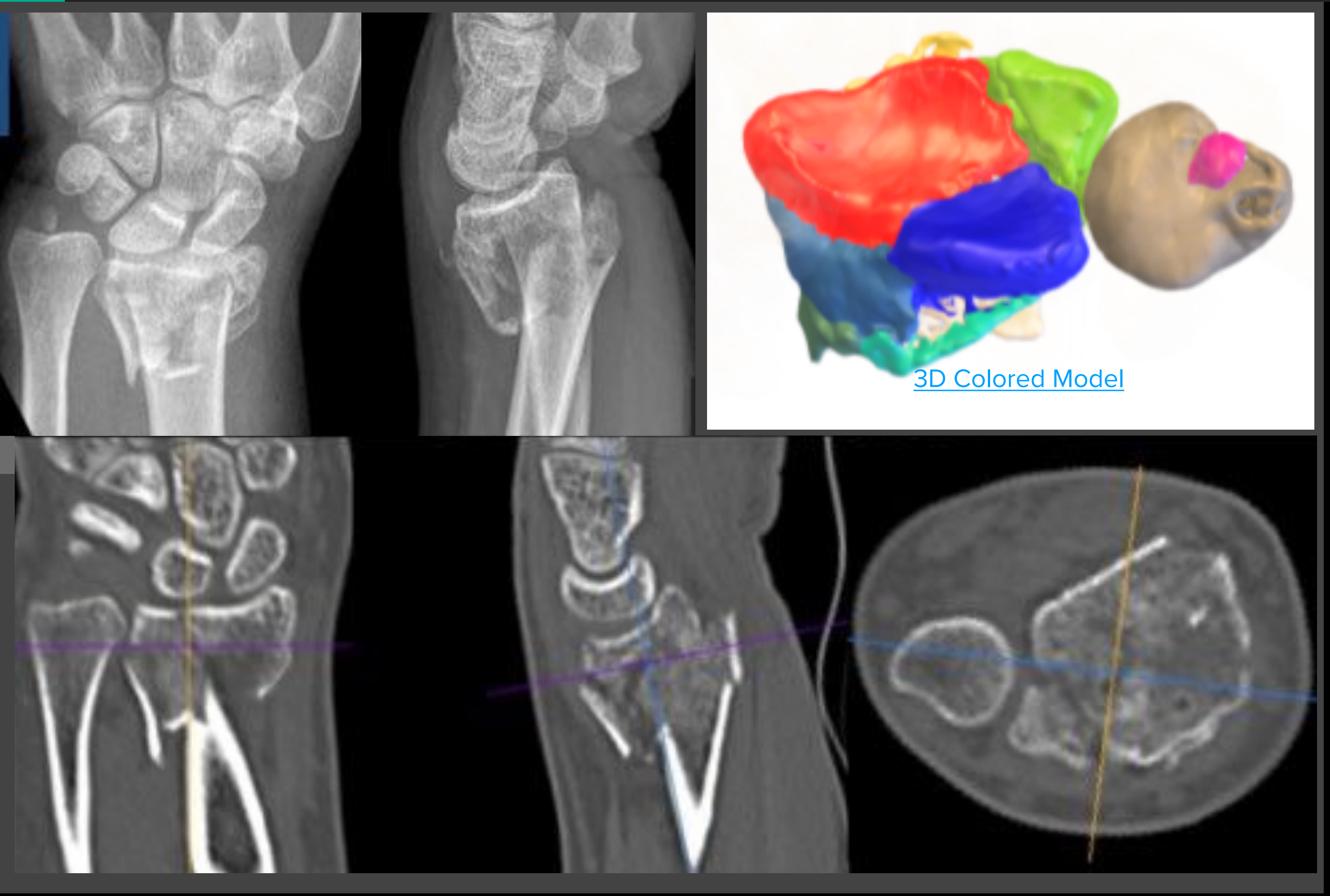

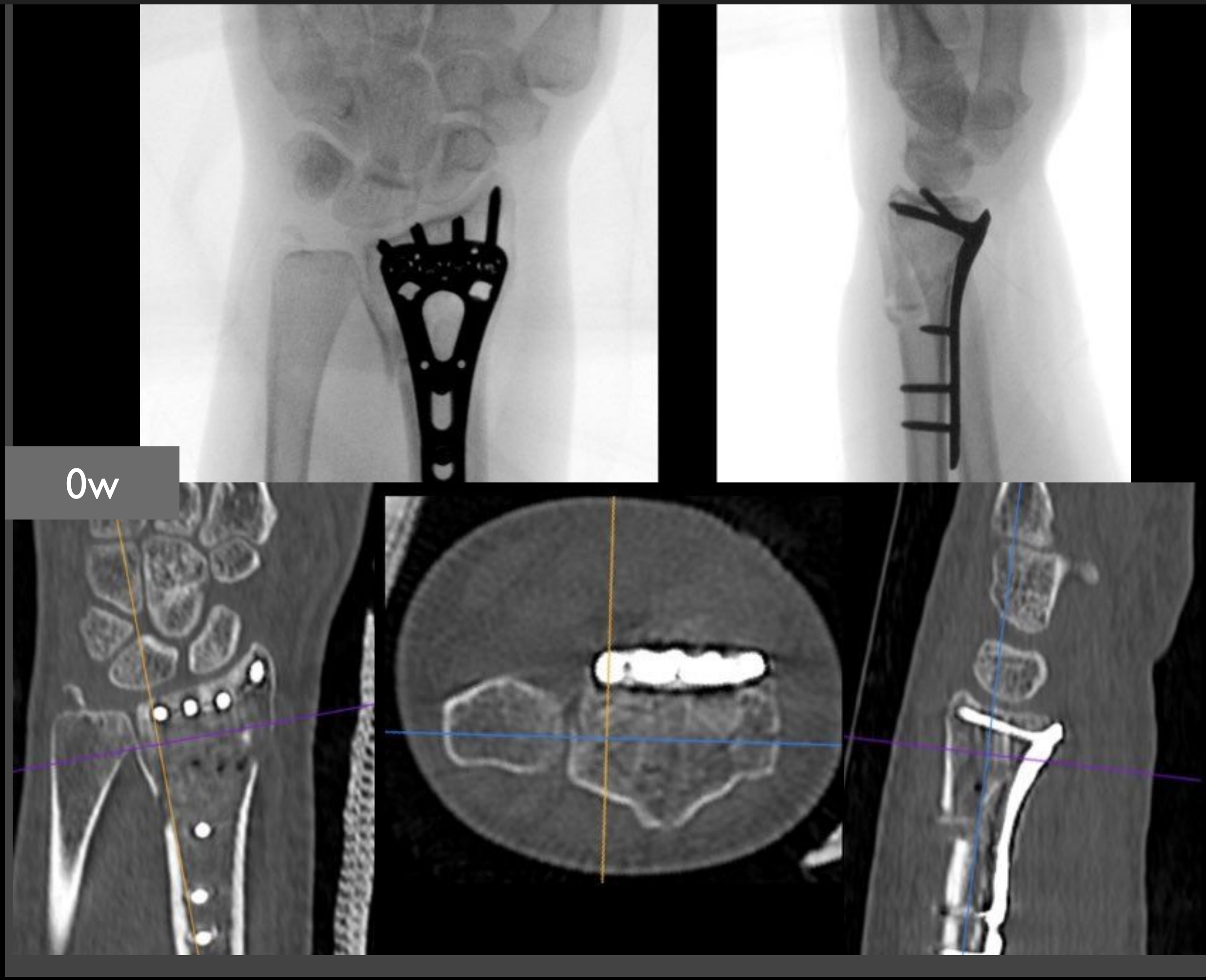

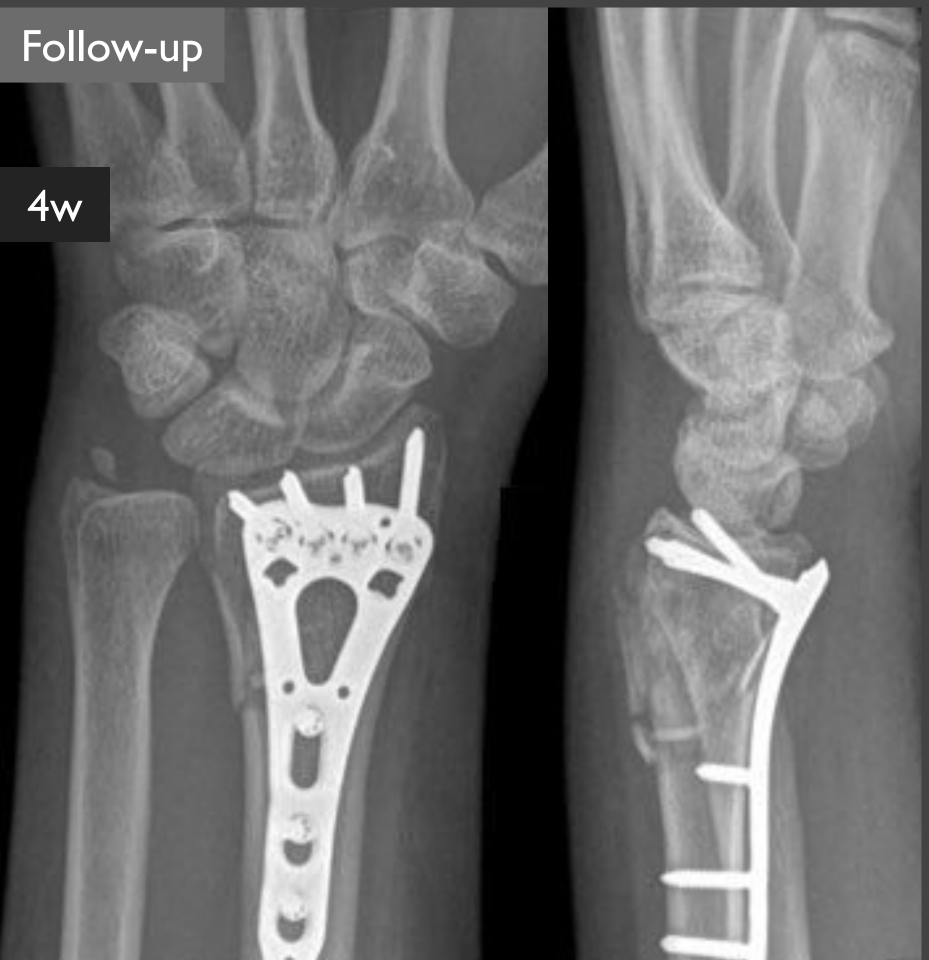

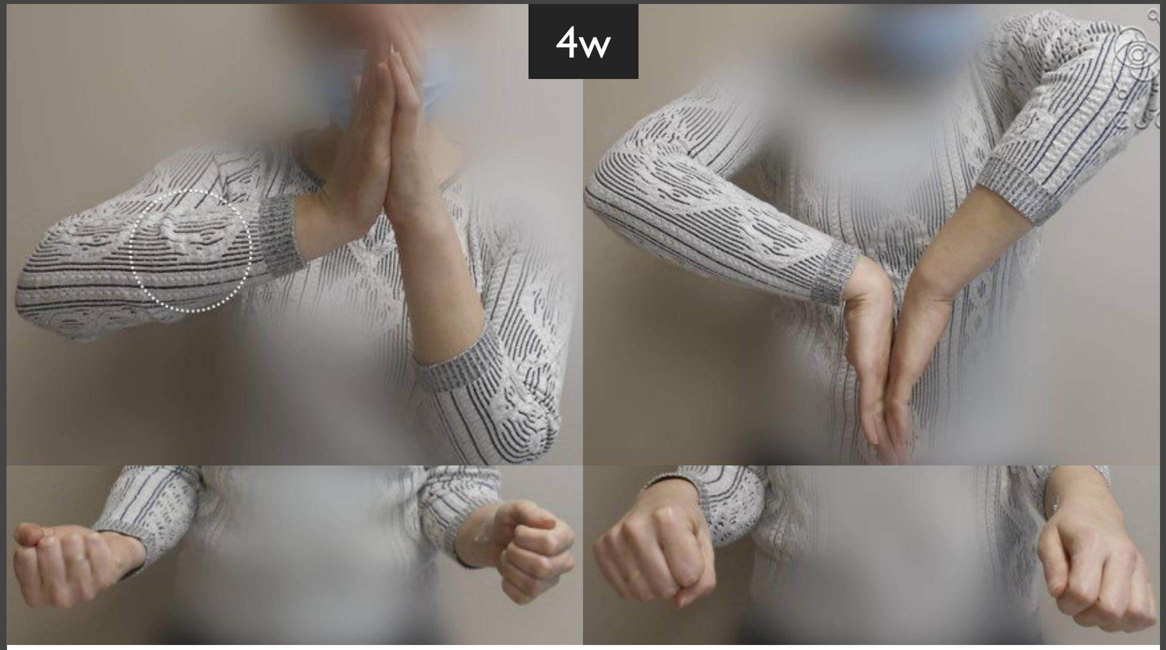

ICUC Score ***Functional Limitation:*** **3** (0-4) - ***Pain:*** **2** (0-4)

ICUC Score at 164w Functional limitation: 1 Pain: 0

Quick DASH = 5

ICUC® ID: 23-DC-358 / 65y Female

*Complete case data can be accessed by clicking the **ICUC ID***

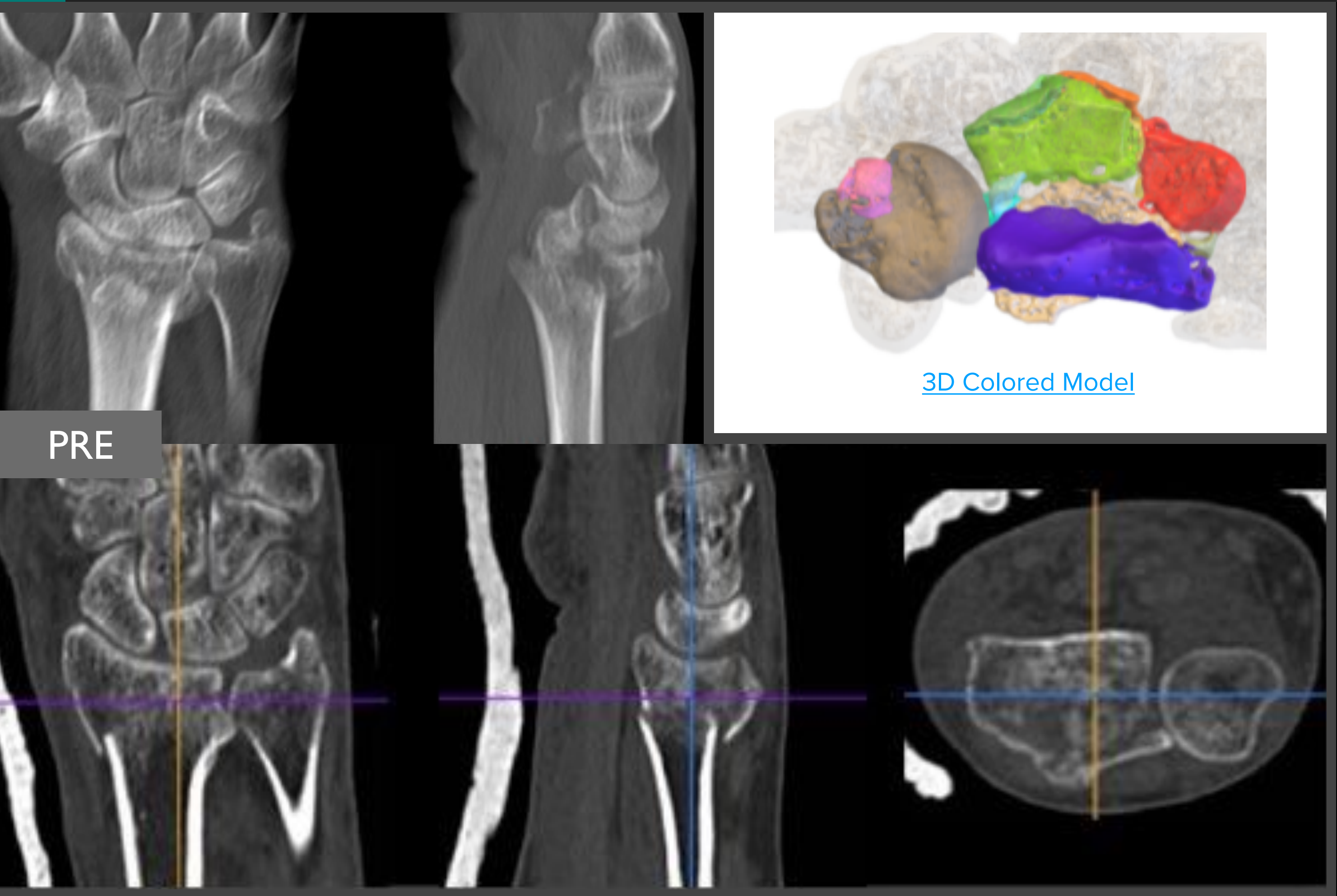

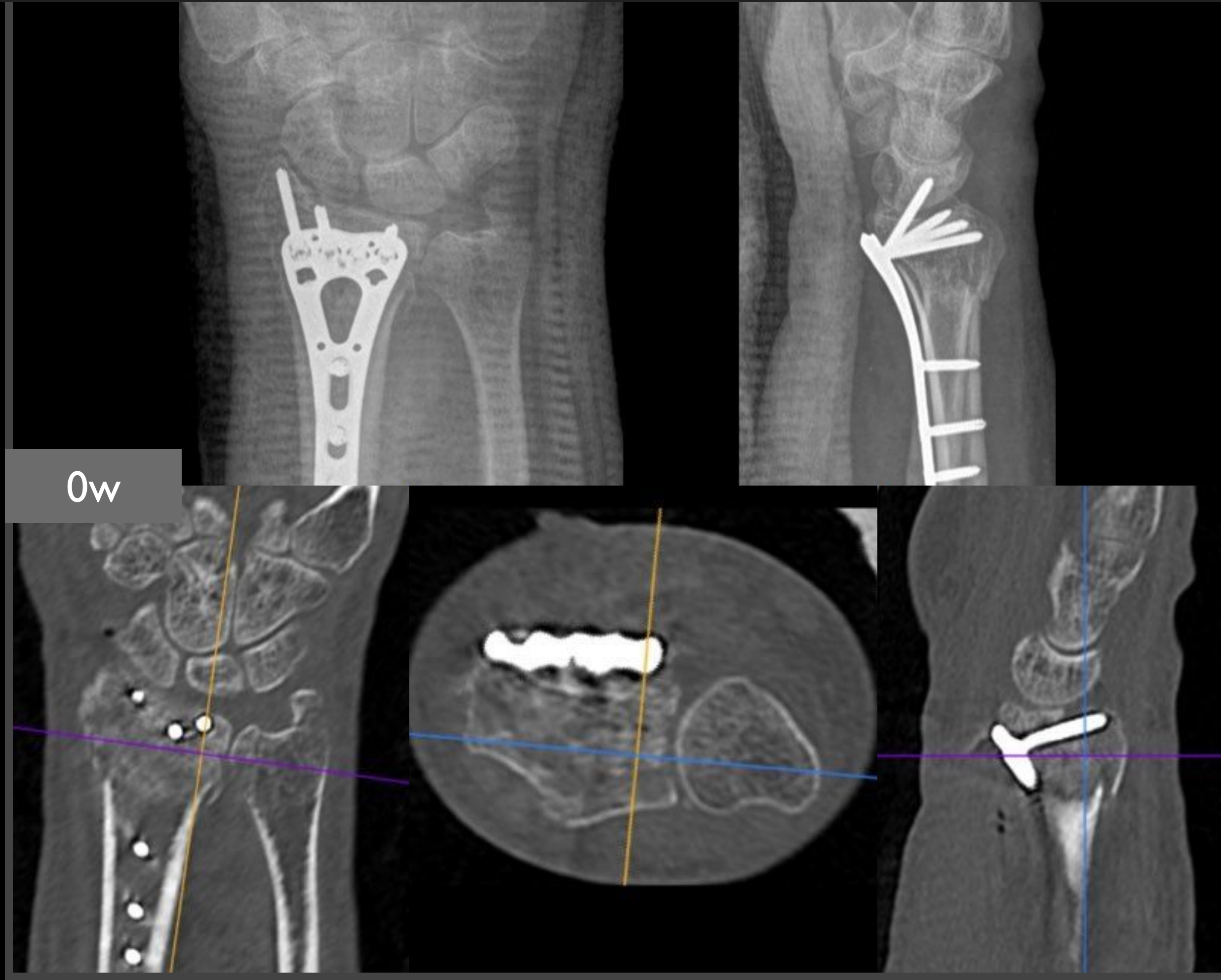

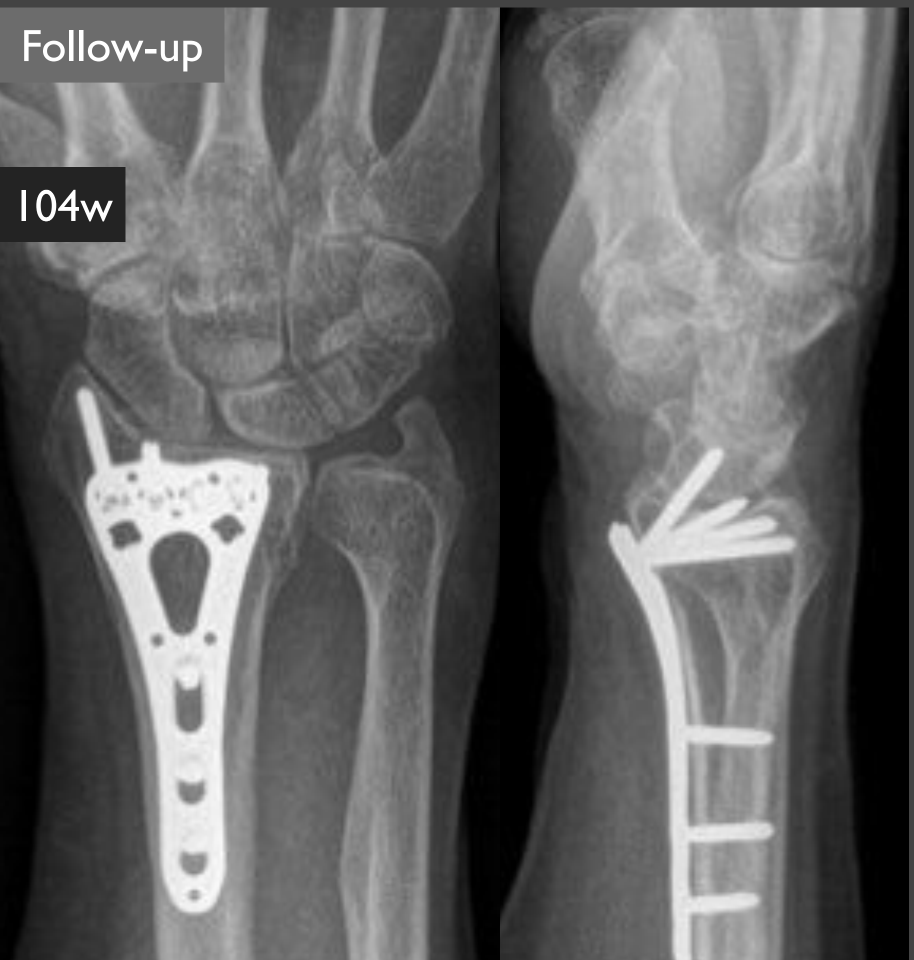

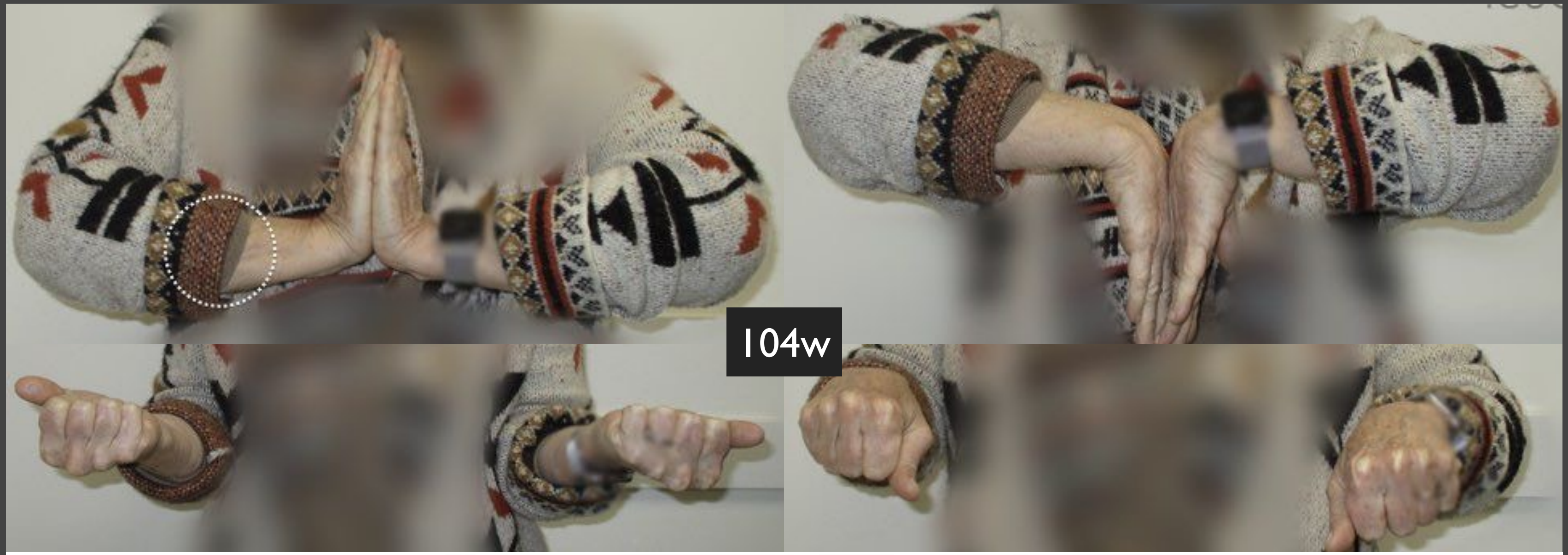

ICUC Score **Functional Limitation: 0** (0-4) - **Pain: 1** (0-4)

ICUC Score at 158w Functional limitation: 0 Pain: 0

Quick DASH = 0

ICUC® ID: 23-DC-408 / 70y Female

Complete case data can be accessed by clicking the ***ICUC ID***

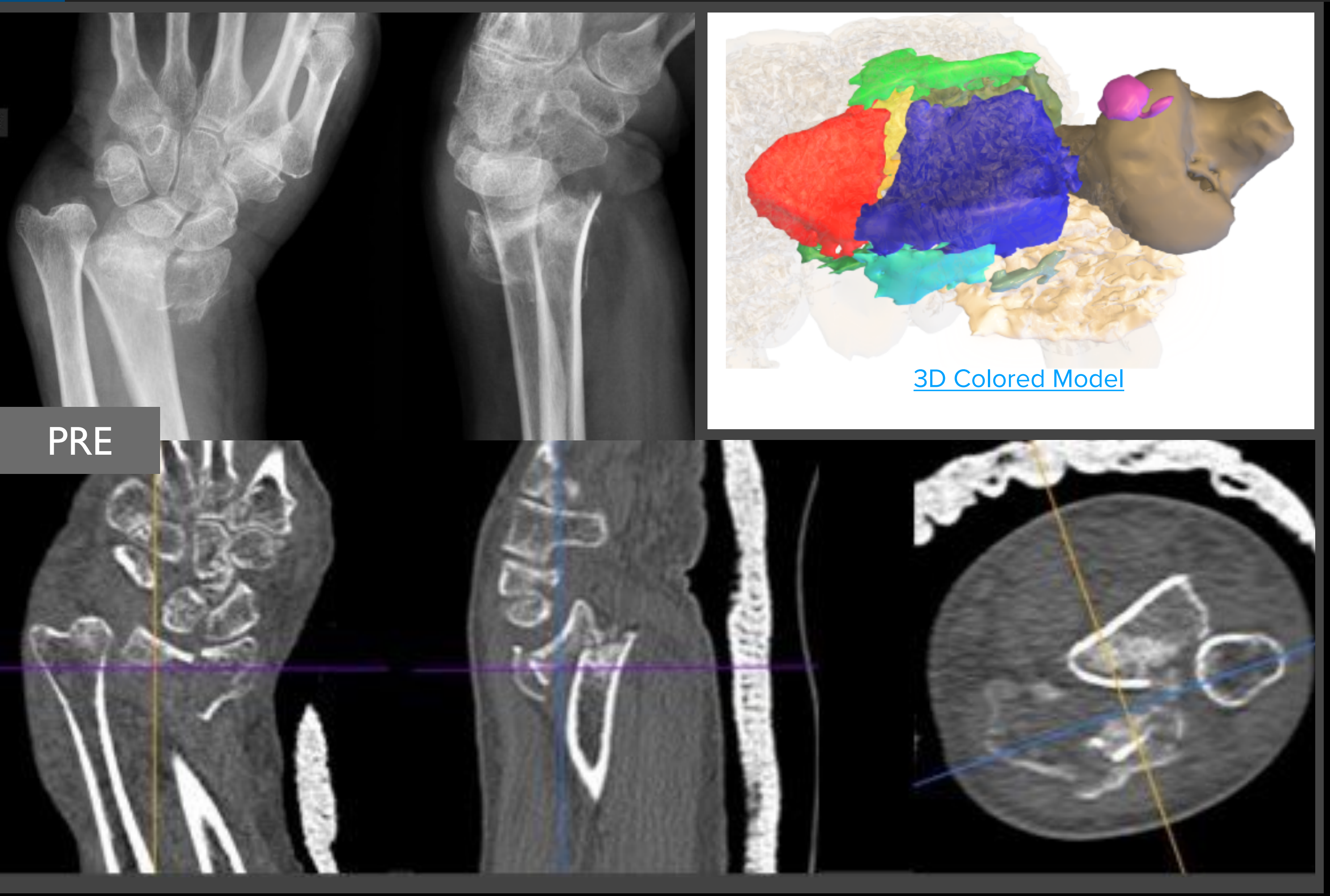

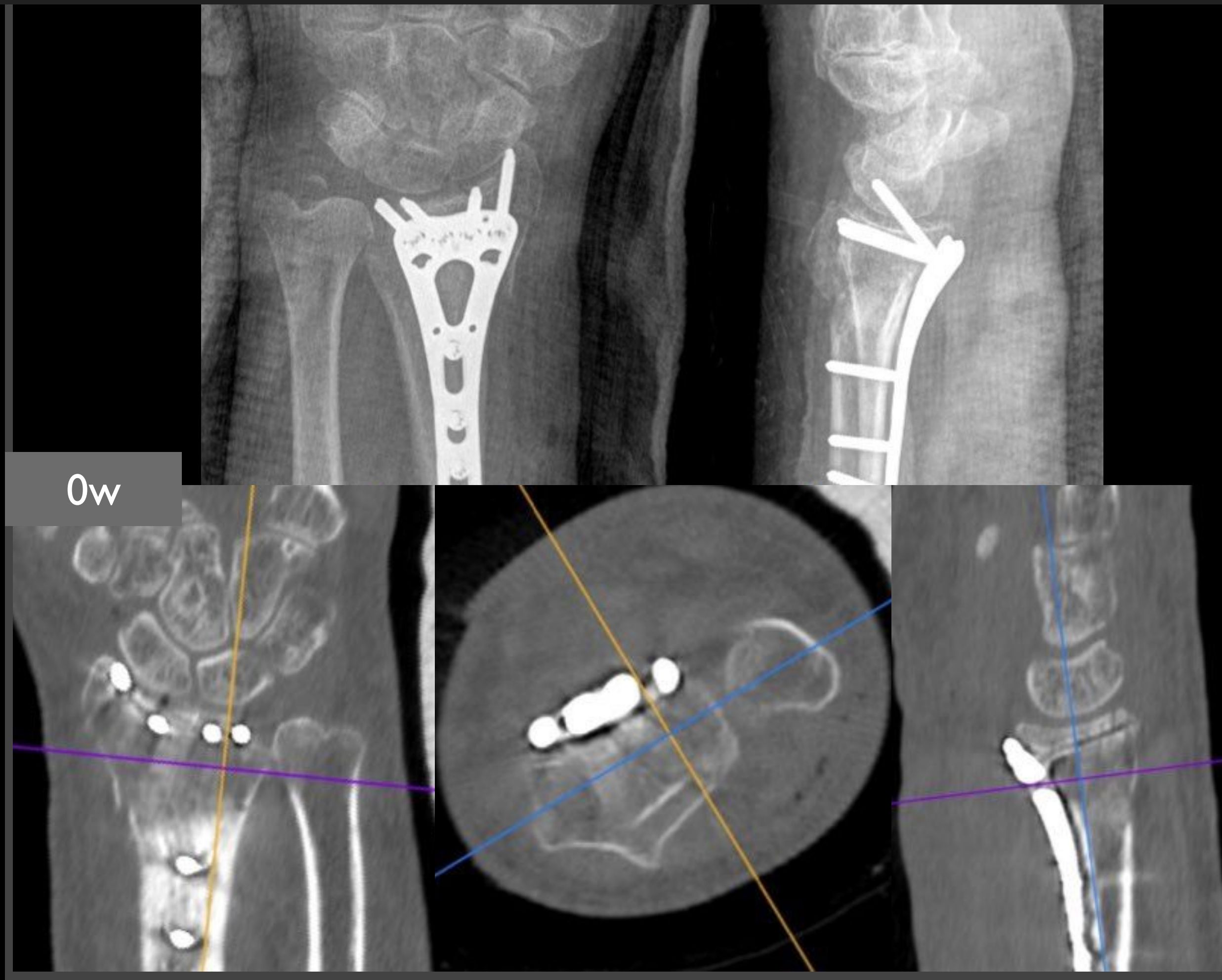

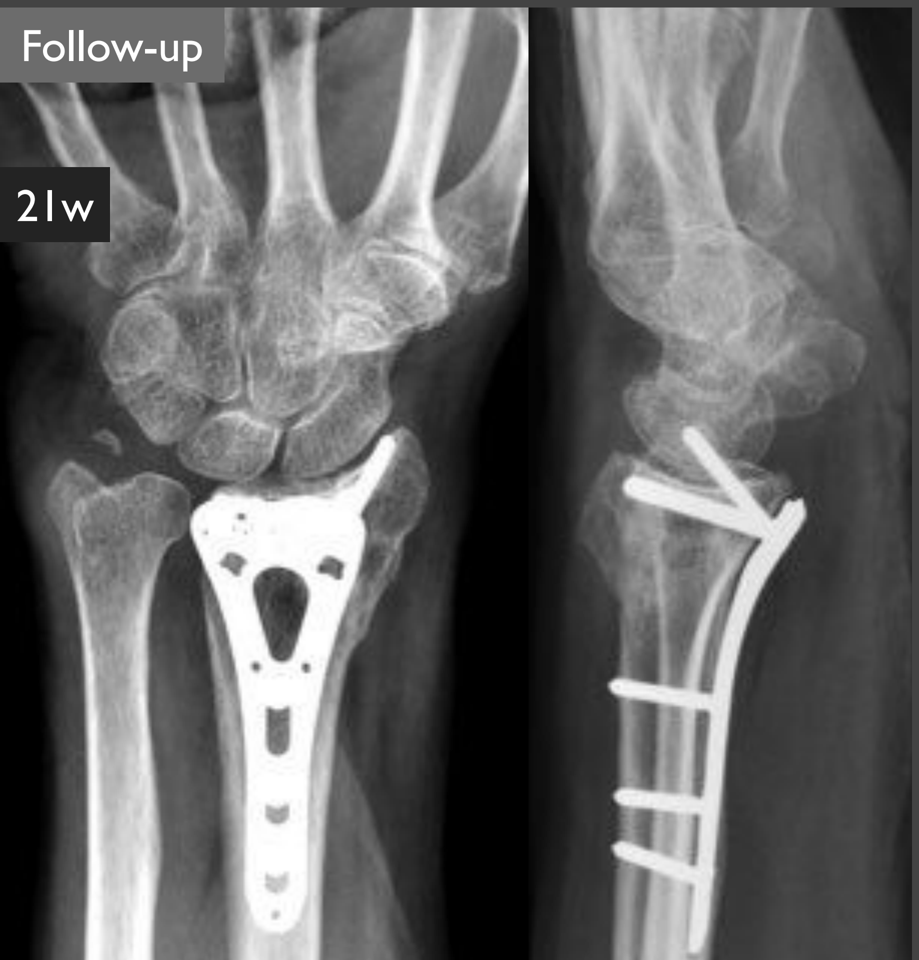

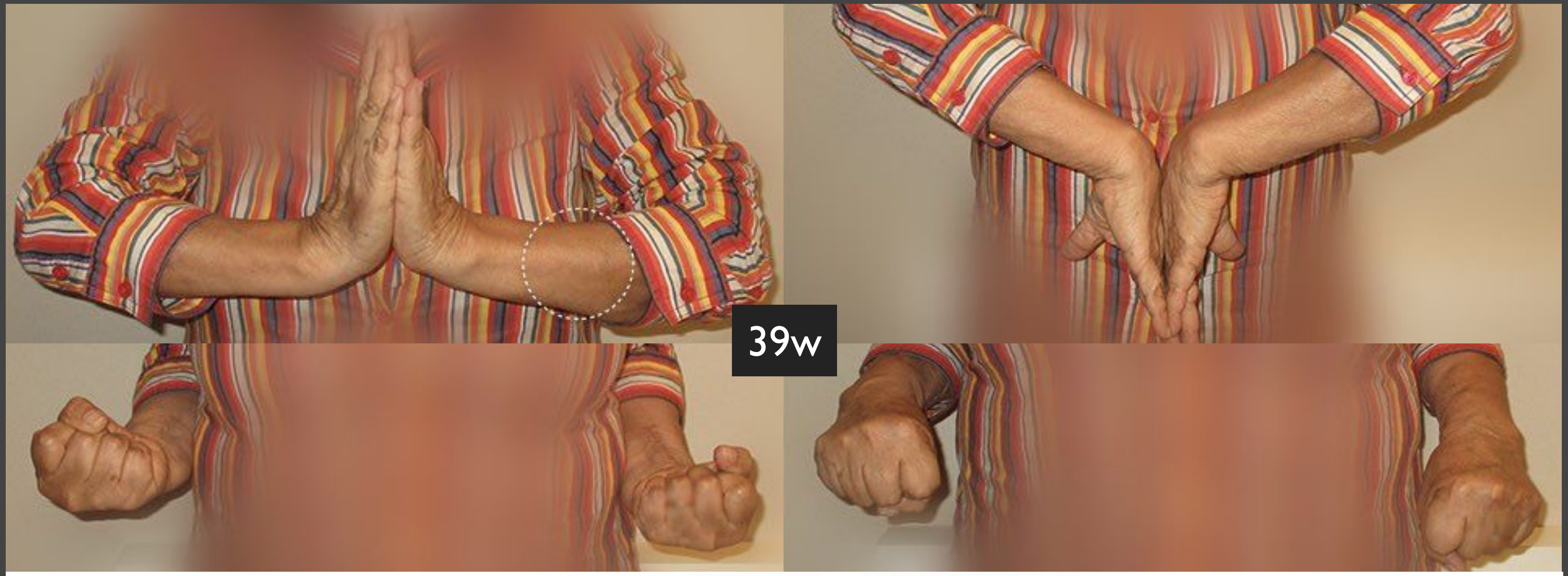

ICUC Score **Functional Limitation: 1** (0-4) - **Pain: 1** (0-4)

ICUC Score at 81w Functional limitation: 0 Pain: 0

Quick DASH = 2

ICUC® ID: 23-DC-453 / 60y Male

*Complete case data can be accessed by clicking the **ICUC ID***

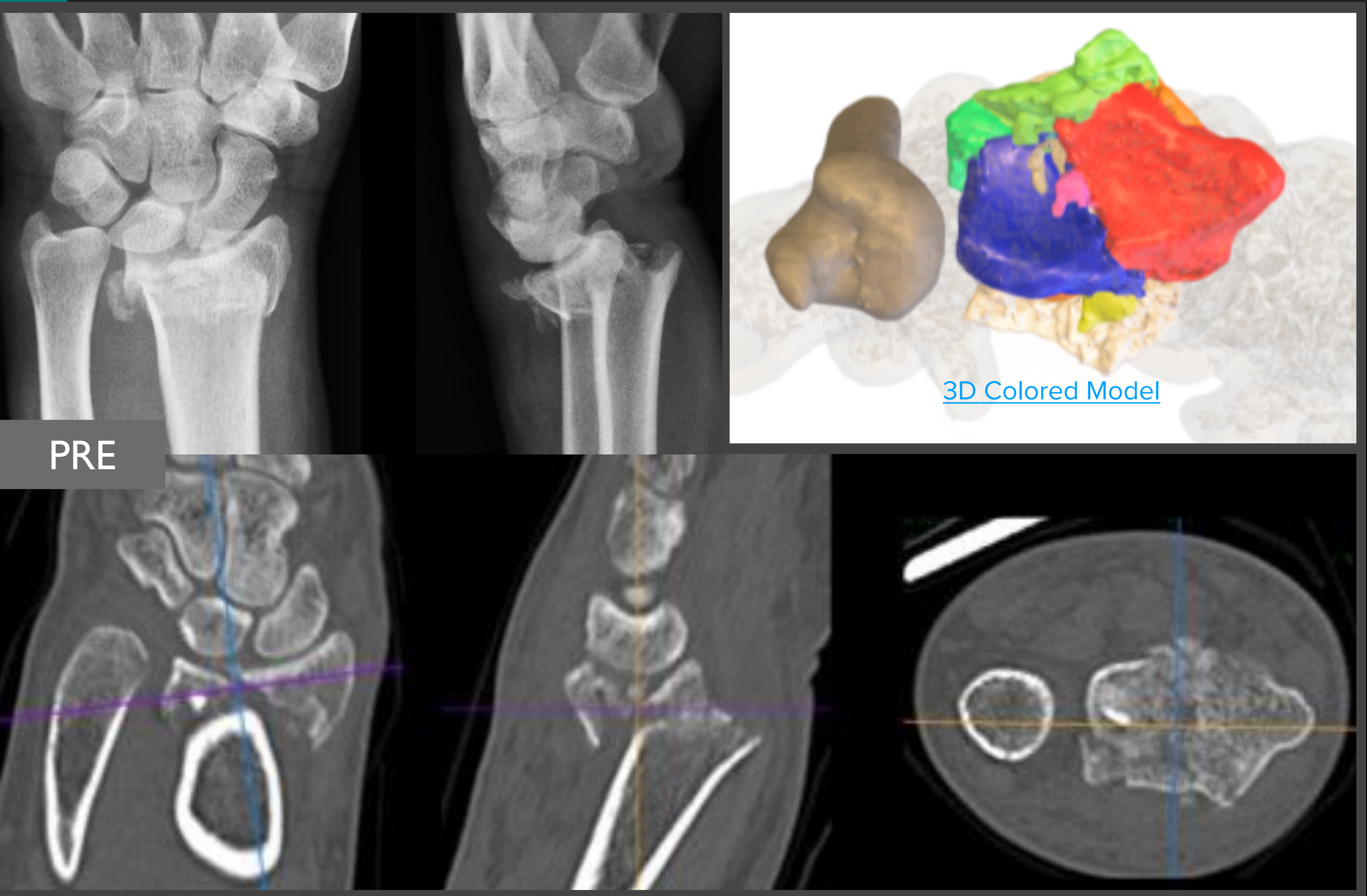

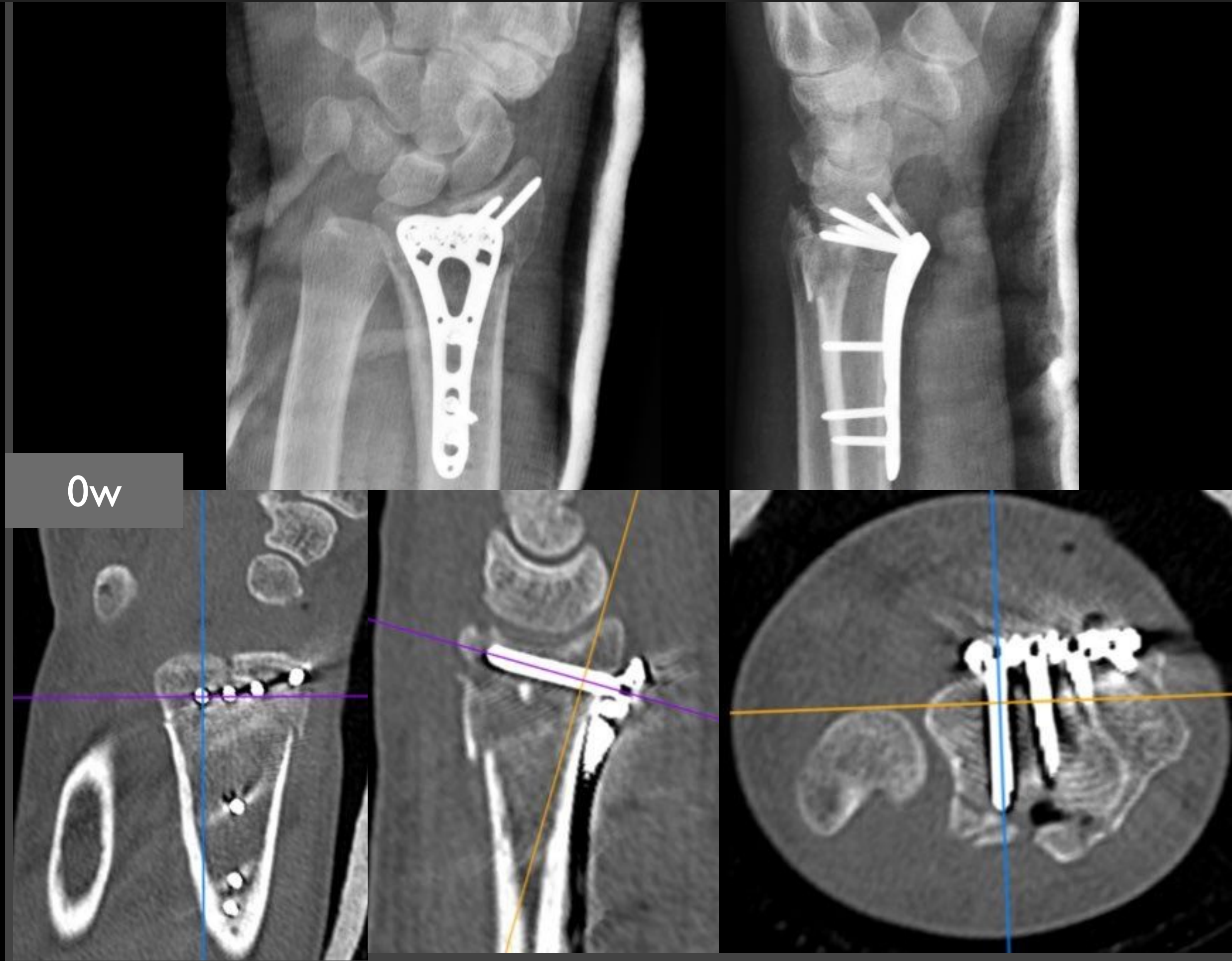

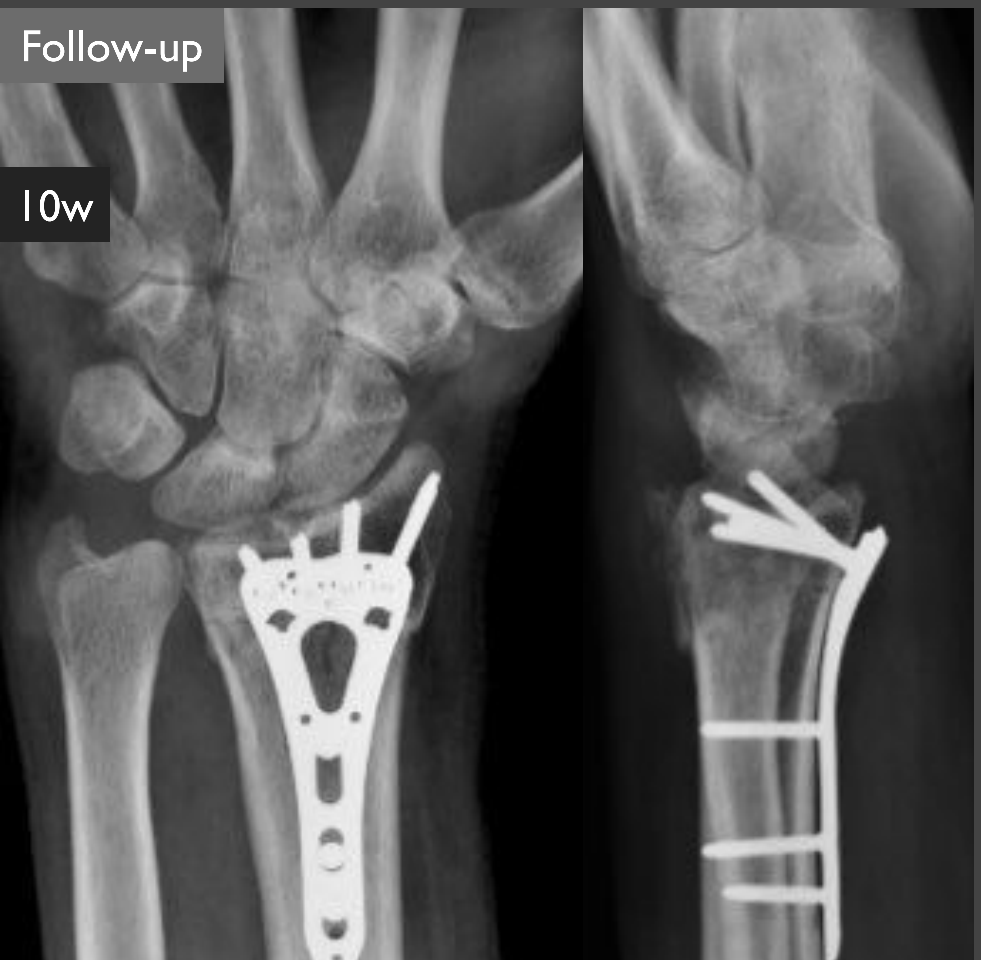

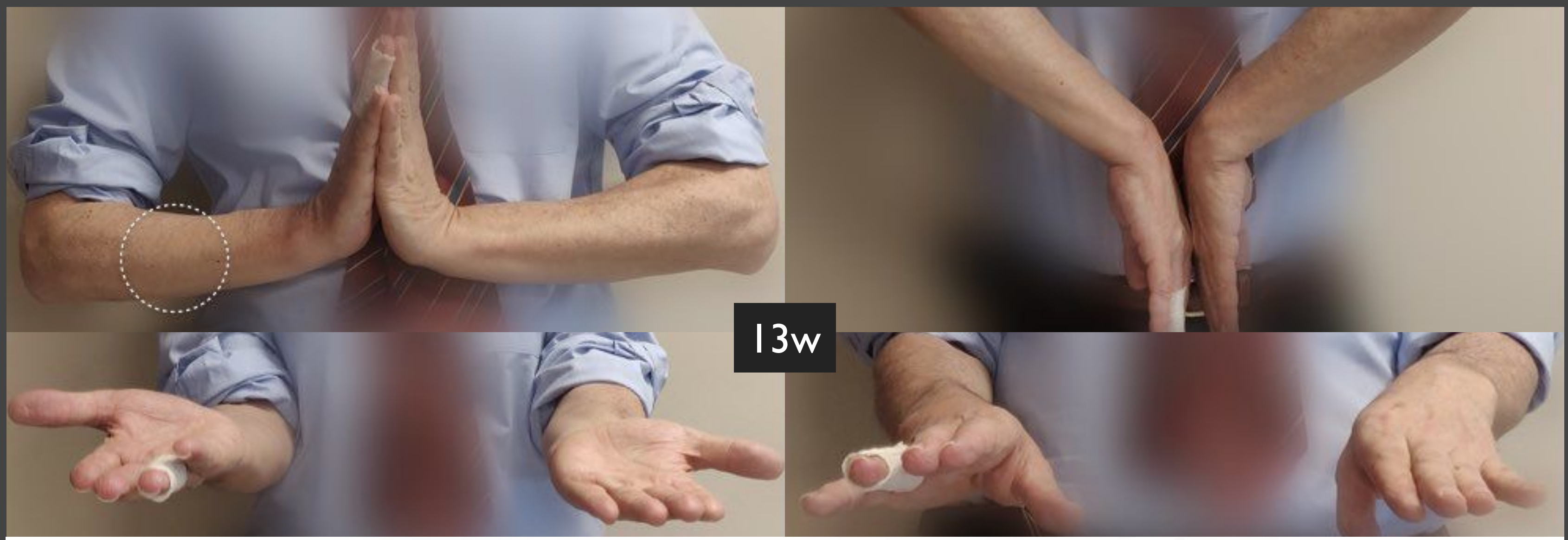

ICUC Score *Functional Limitation:* **1** (0-4) - *Pain:* **1** (0-4)

ICUC Score at 109w Functional limitation: 1 Pain: 1

Quick DASH = 11

ICUC® ID: 23-DC-605 / 55y Female

Complete case data can be accessed by clicking the ***ICUC ID***

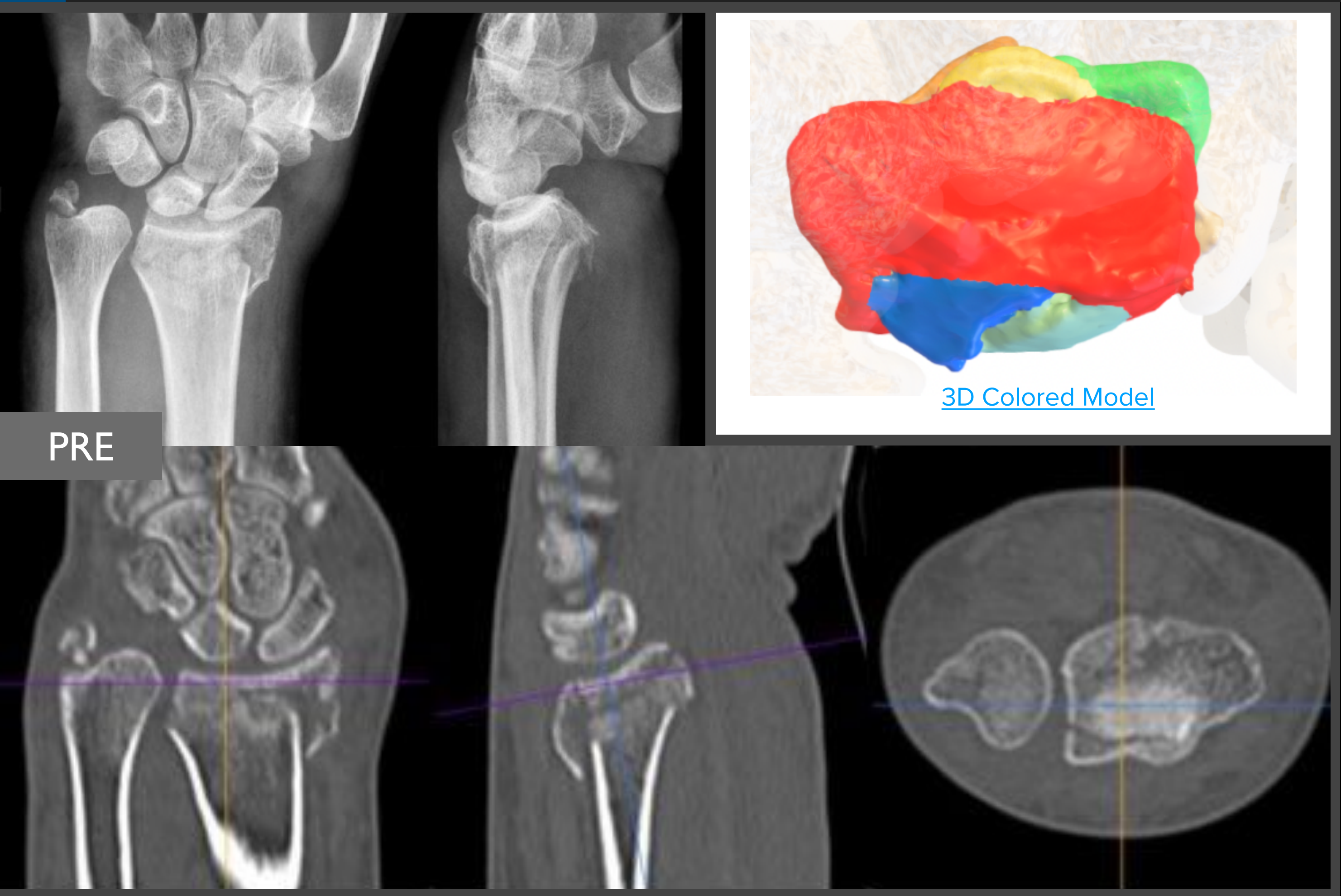

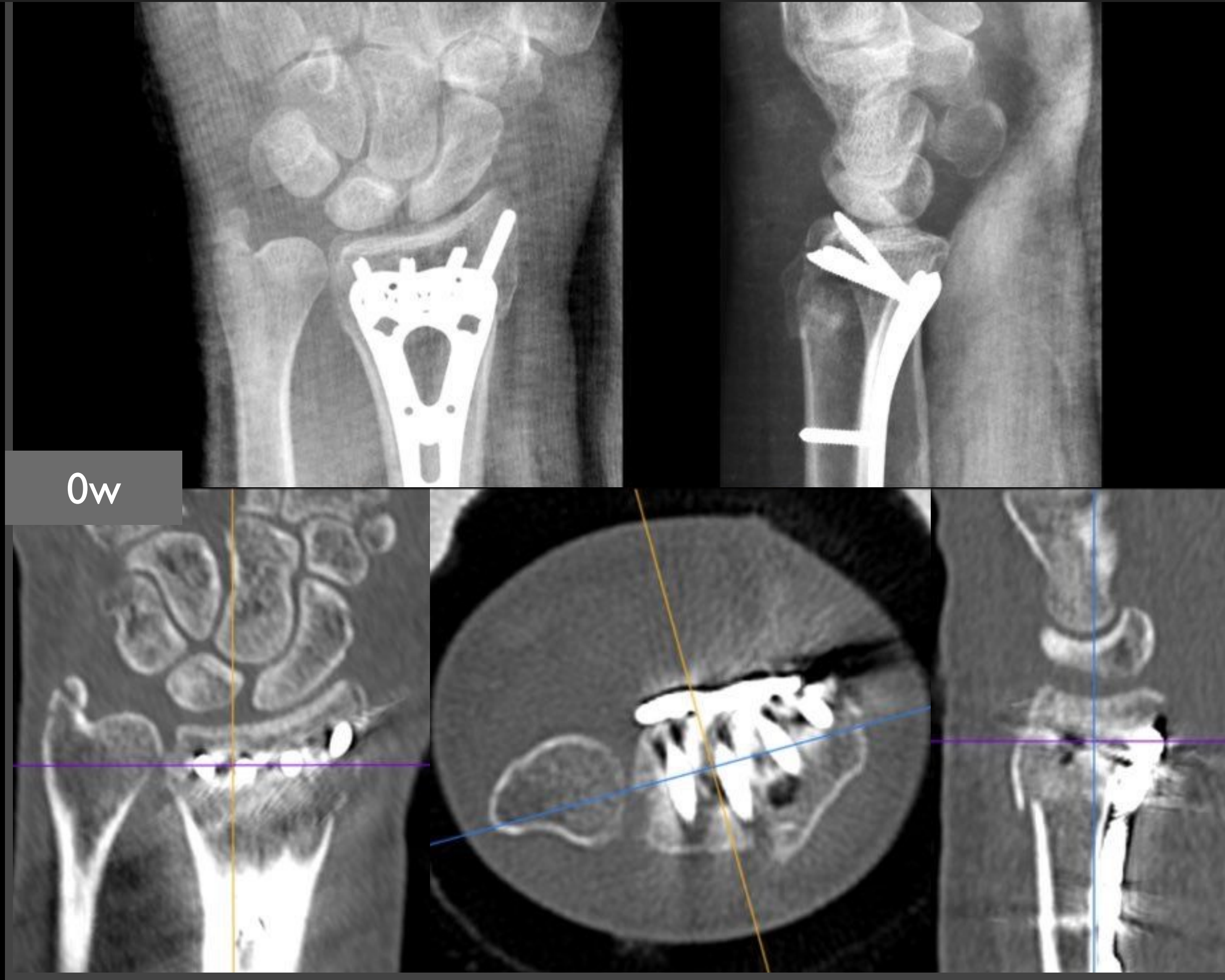

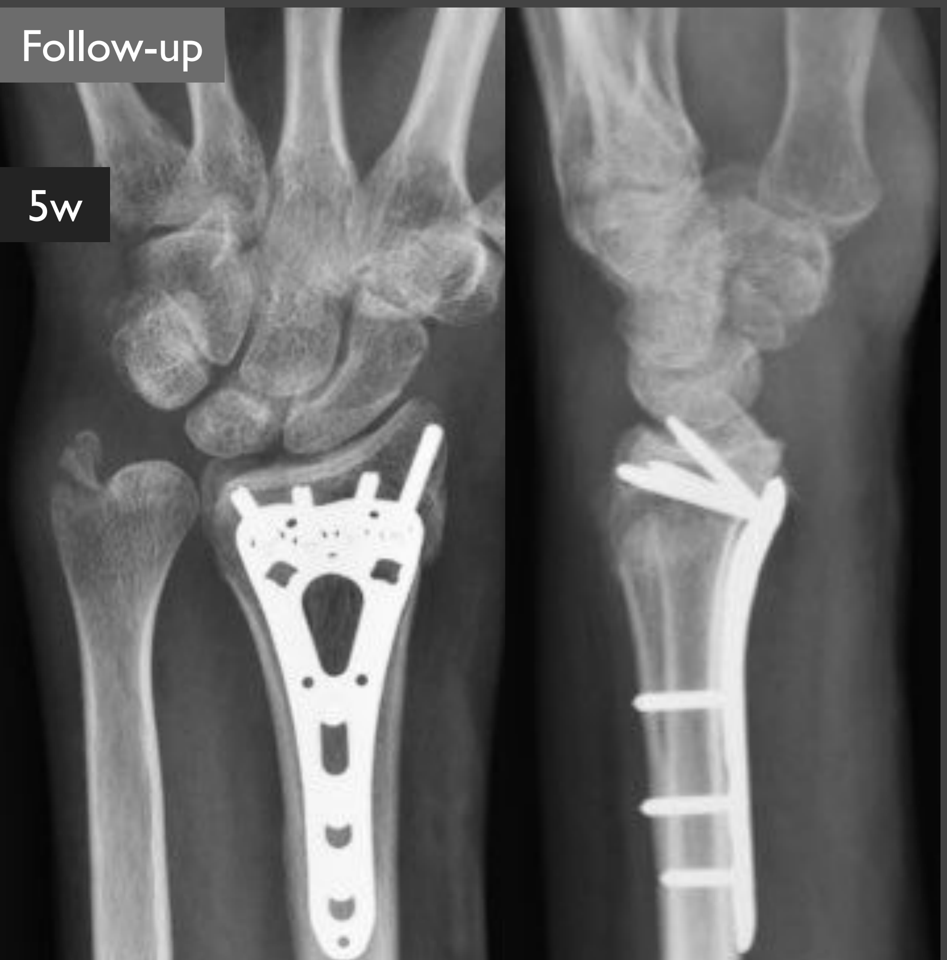

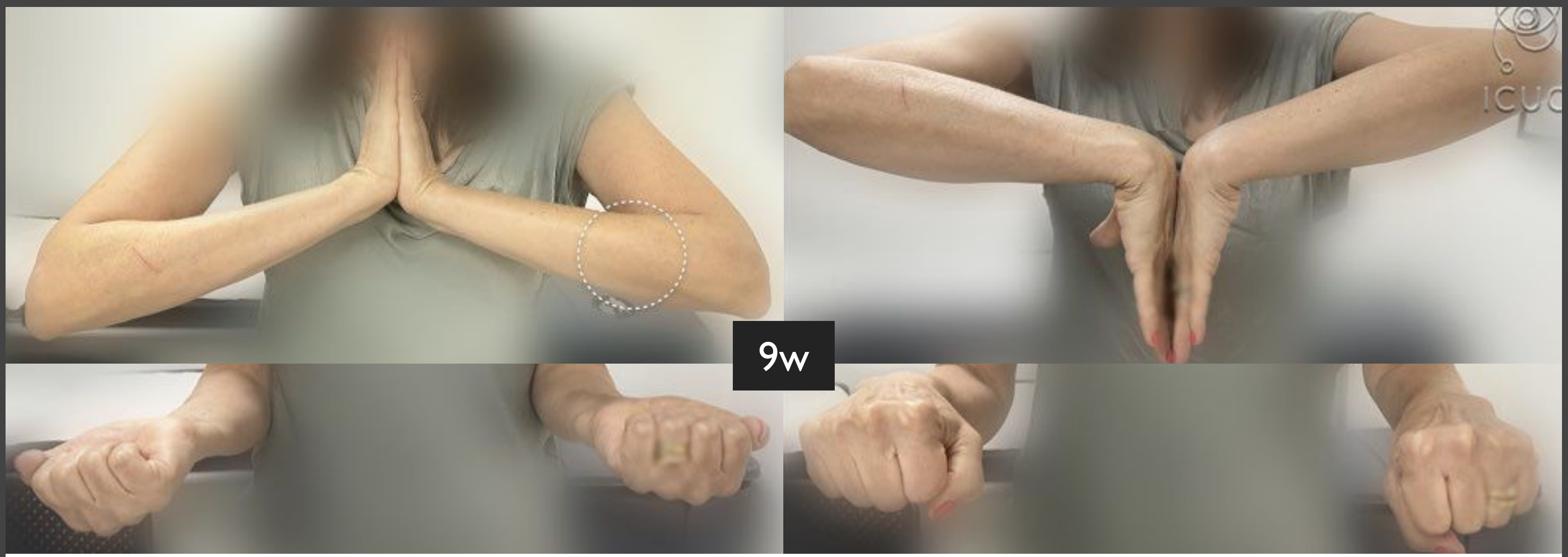

ICUC Score ***Functional Limitation:*** **0** (0-4) - ***Pain:*** **0** (0-4)

ICUC Score at 46w Functional limitation: 0 Pain: 0

Quick DASH = 0

ICUC® ID: 23-DC-623 / 70y Female

*Complete case data can be accessed by clicking the **ICUC ID***

PRE

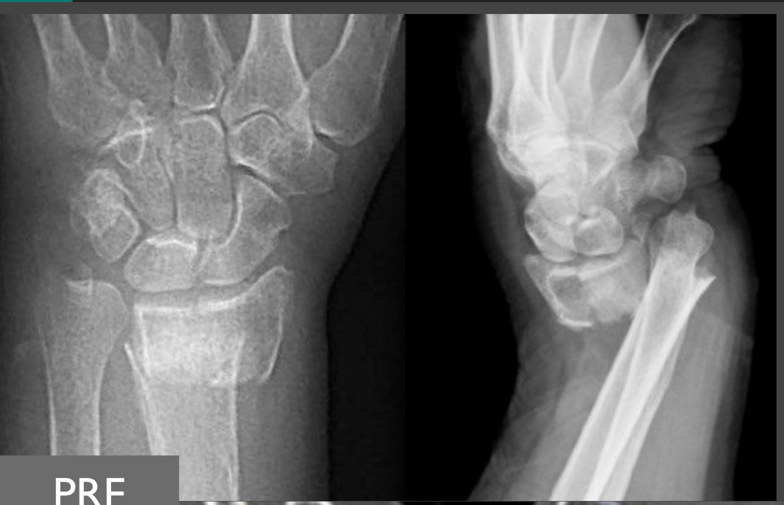

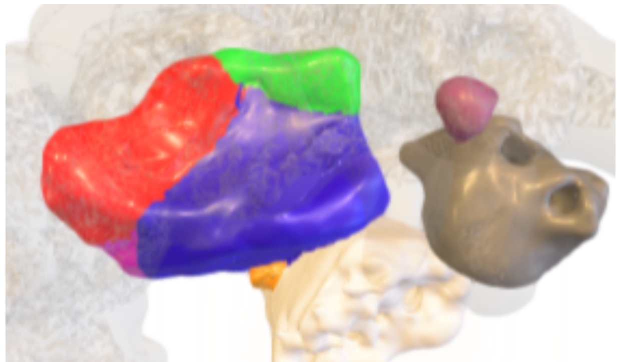

3D Colored Model

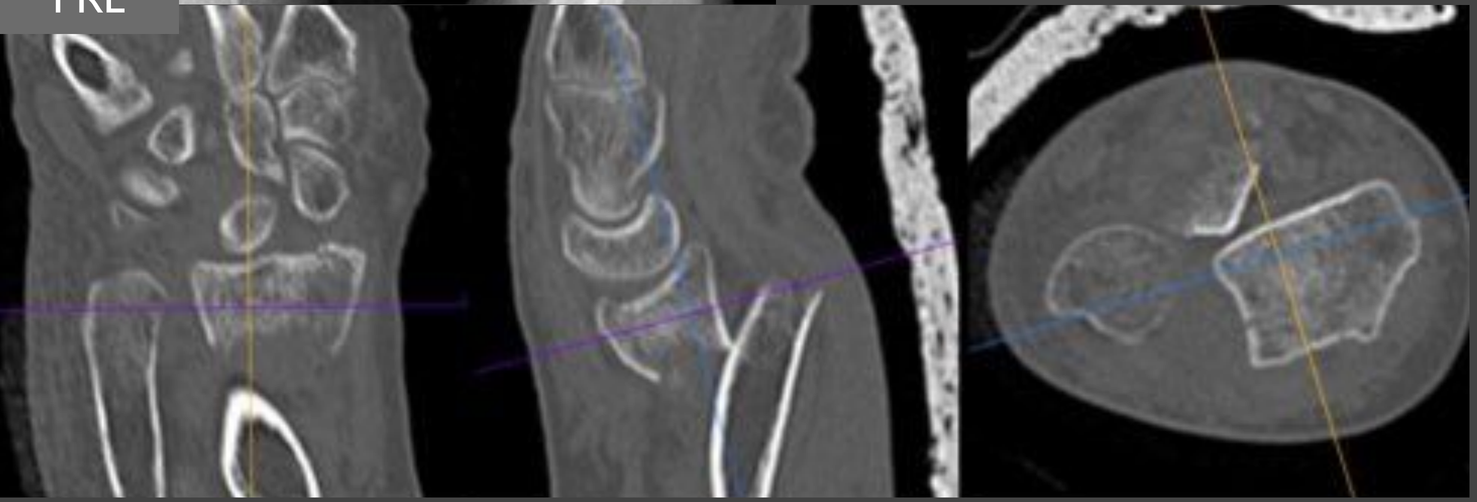

0w

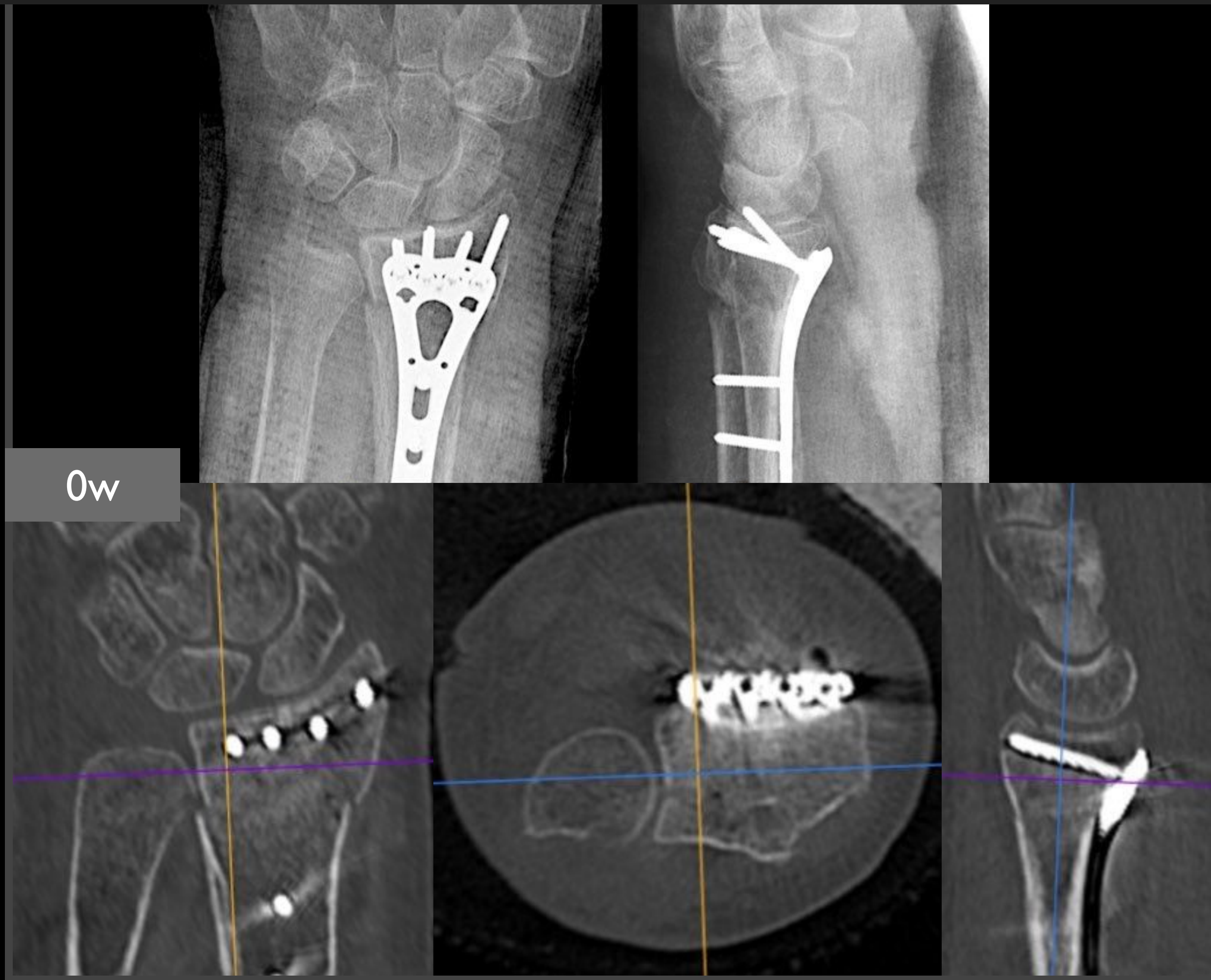

Follow-up

21w

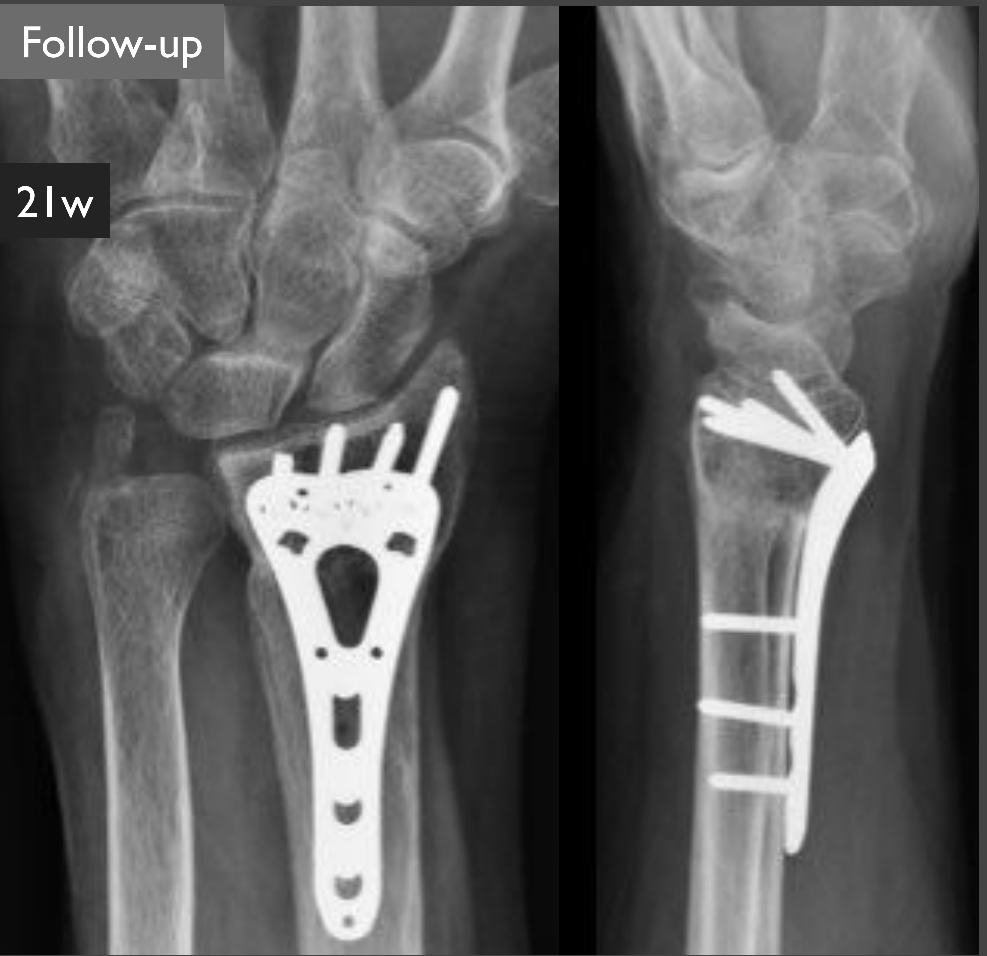

10w

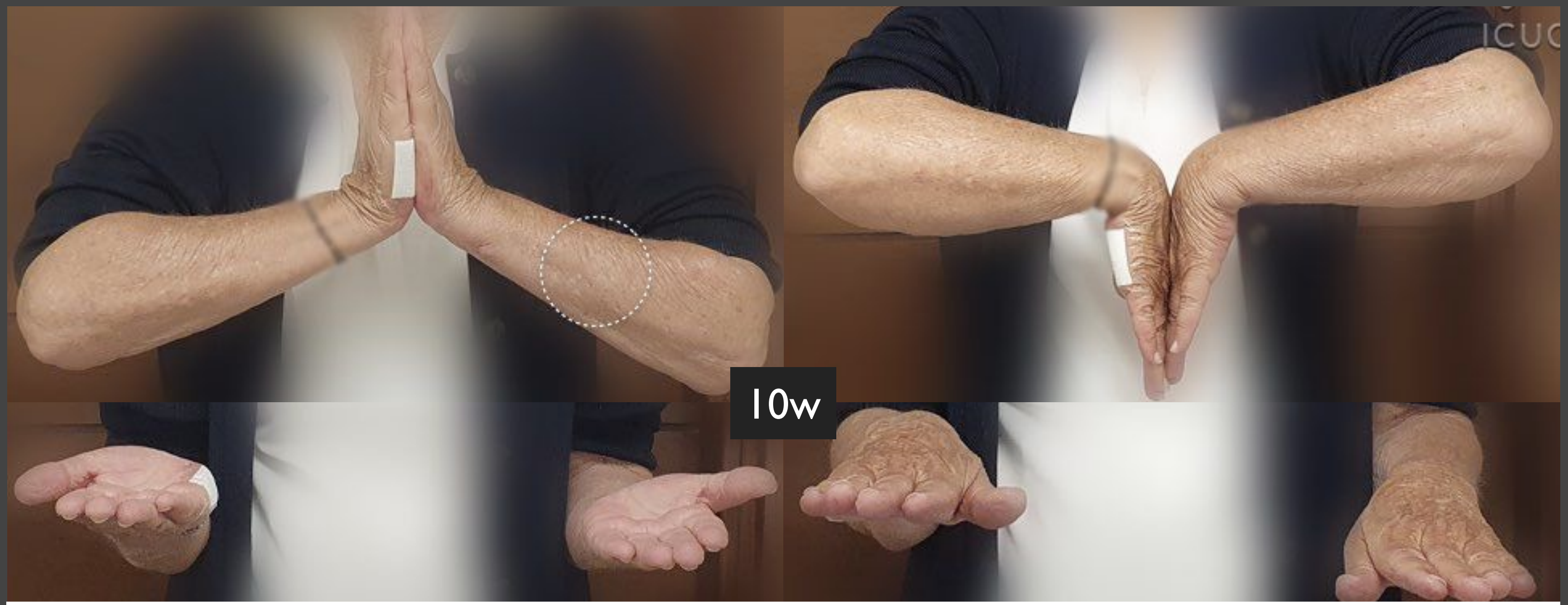

ICUC Score Functional Limitation: 1 (0-4) - Pain: 1 (0-4)

ICUC Score at 110w Functional limitation: 1 Pain: 0

Quick DASH = 23

ICUC® ID: 23-DC-902 / 70y Female

Complete case data can be accessed by clicking the ***ICUC ID***

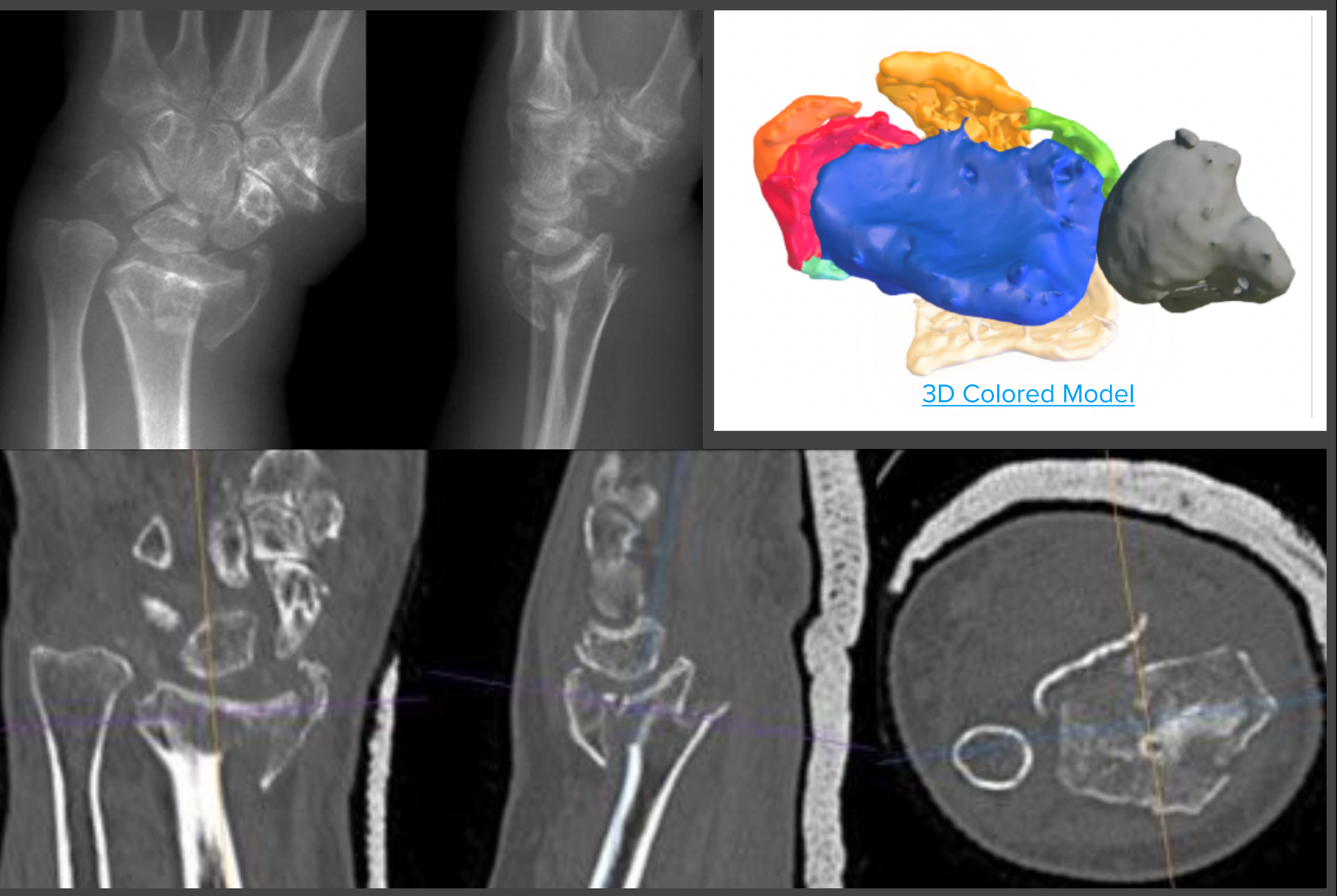

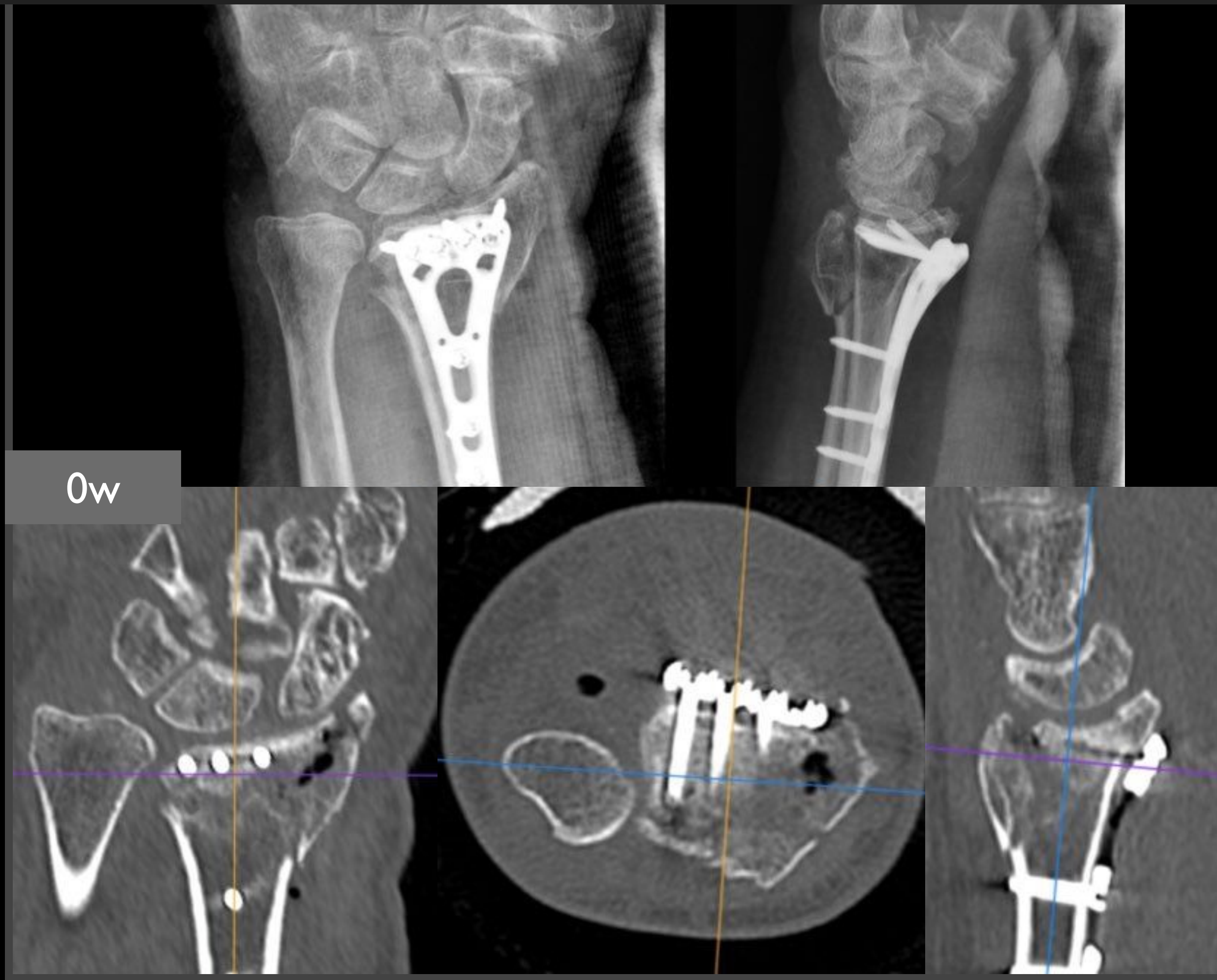

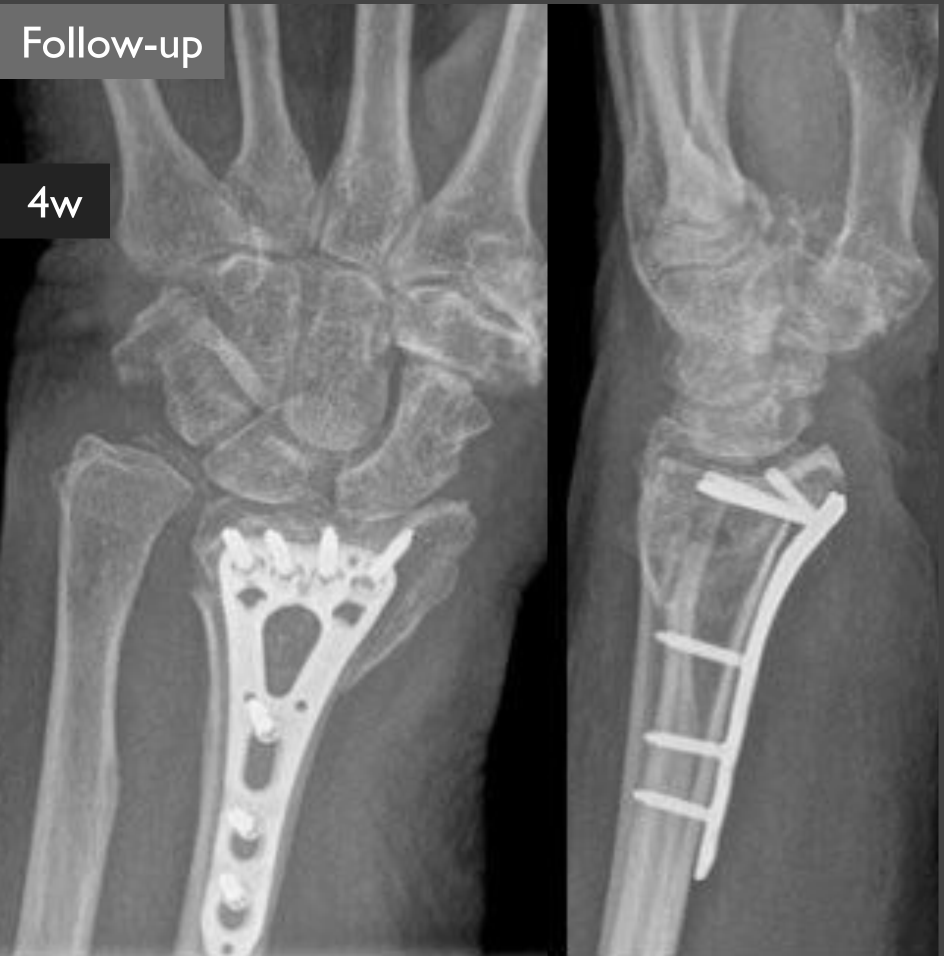

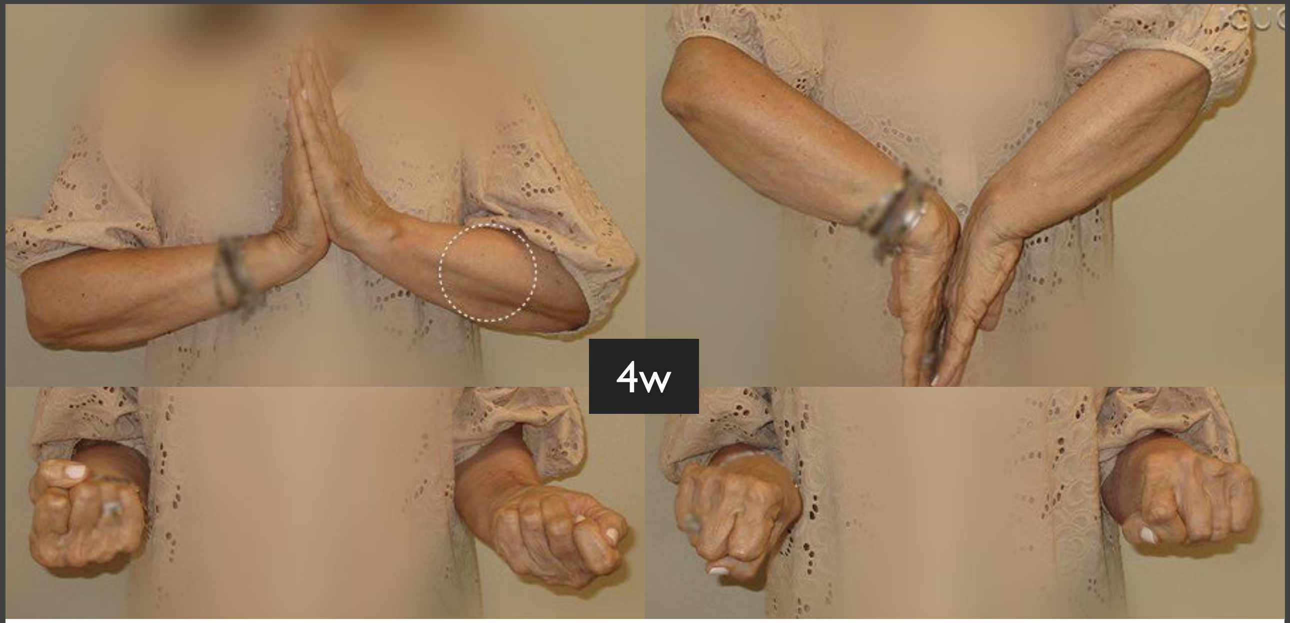

ICUC Score **Functional Limitation: 3** (0-4) - **Pain: 1** (0-4)

ICUC Score at 38w Functional limitation: 0 Pain: 1

Quick DASH = 5

ICUC® ID: 23-DI-711 / 55y Male

Complete case data can be accessed by clicking the ***ICUC ID***

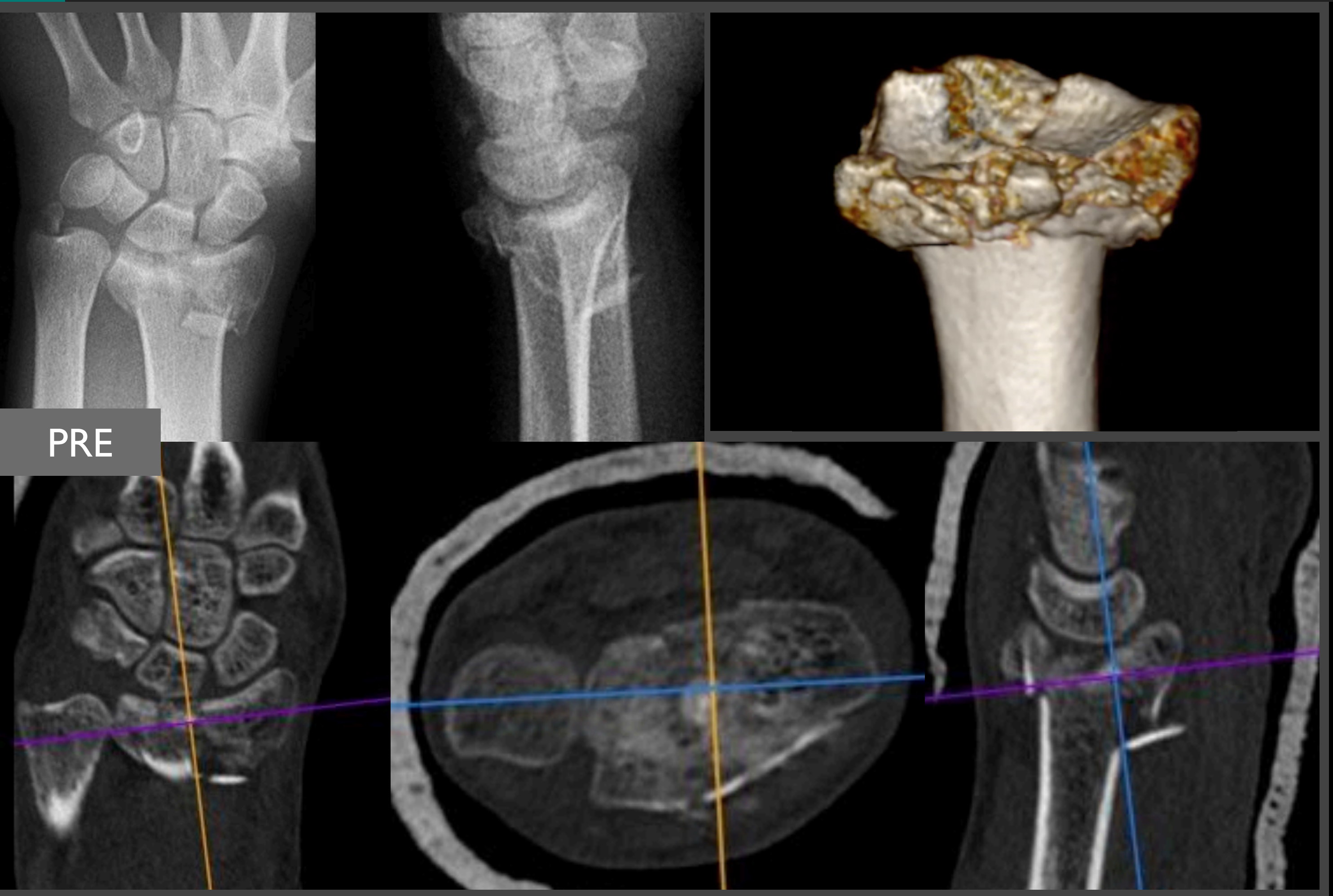

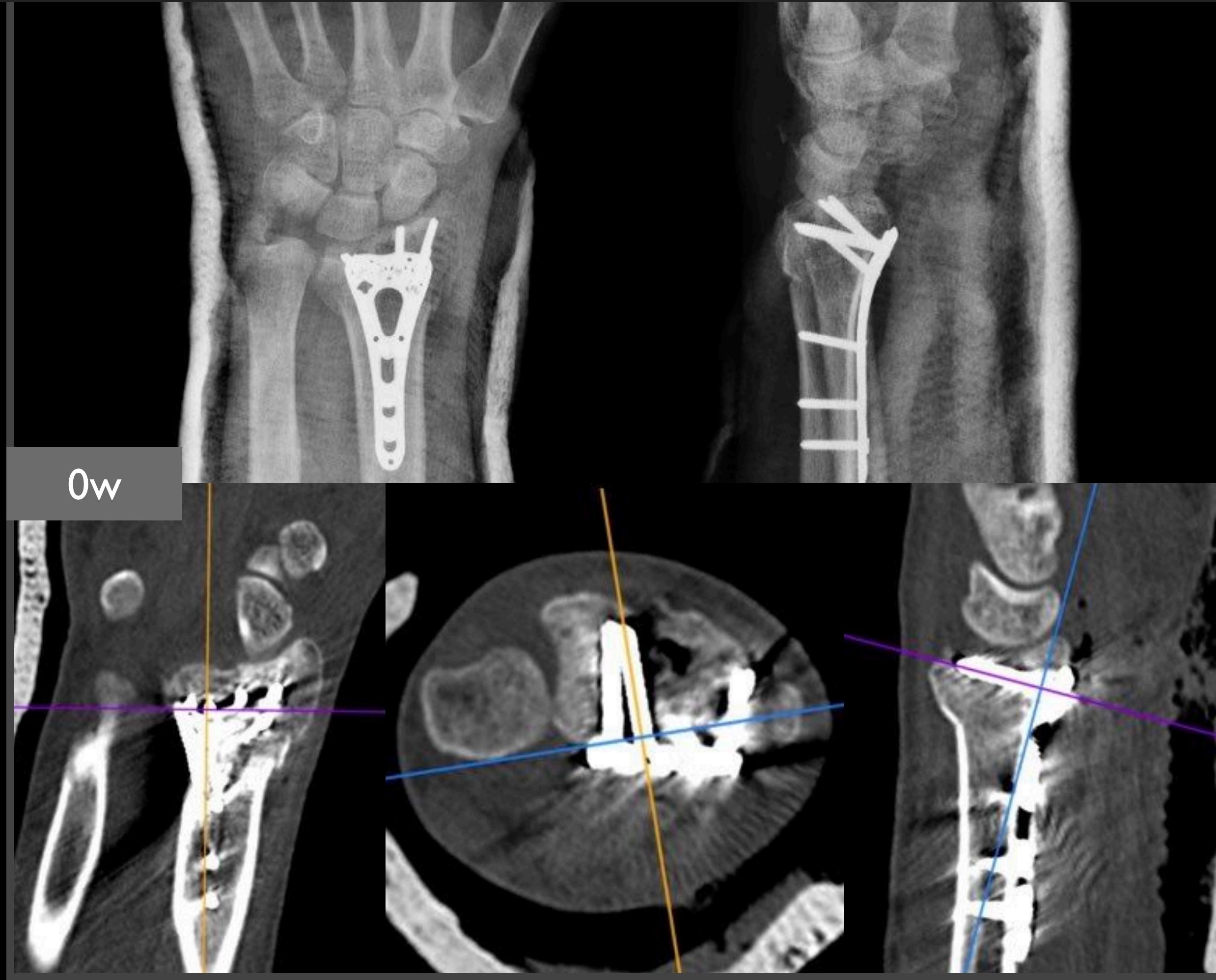

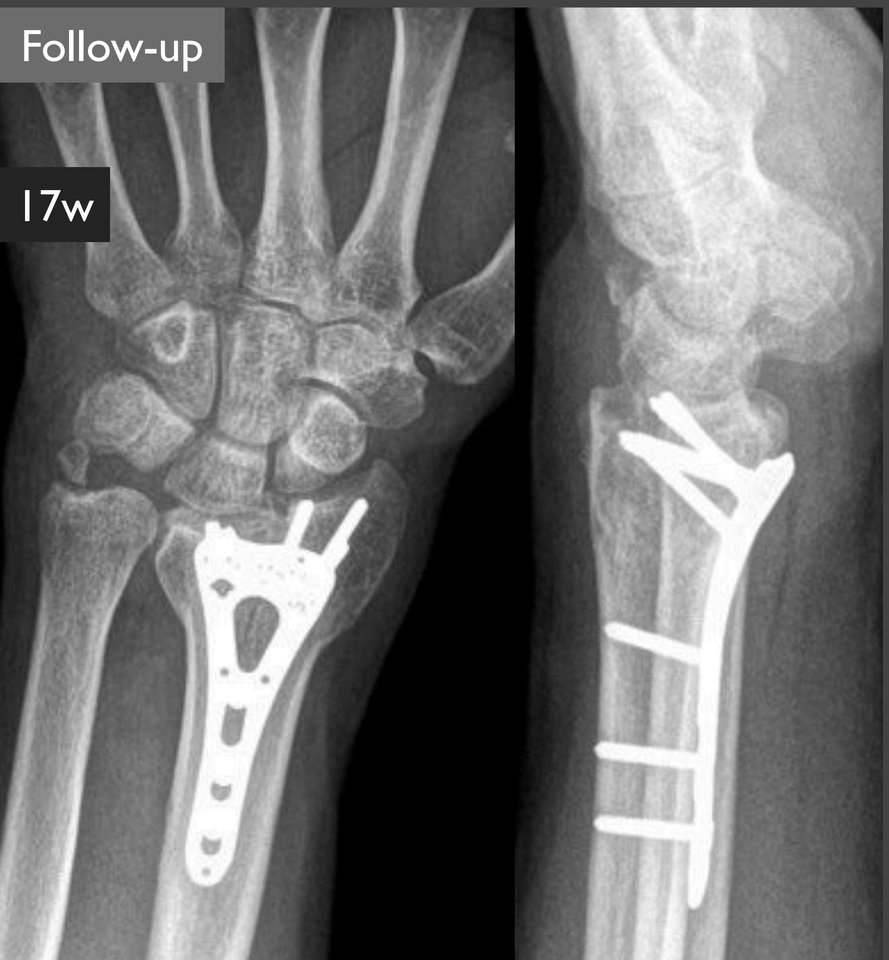

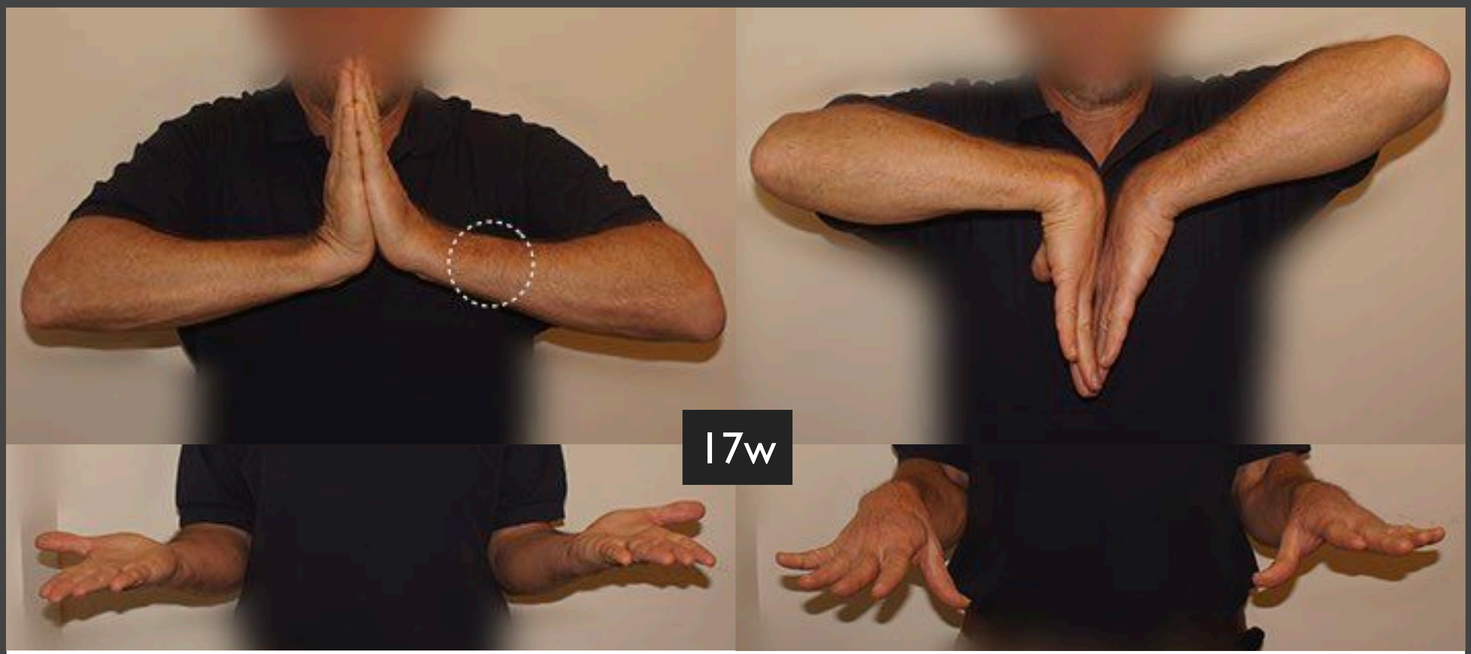

ICUC **Score** *Functional Limitation:* **1** (0-4) - *Pain:* **1** (0-4)

ICUC Score at 80w Functional limitation: 1 Pain: 0

Quick DASH = 1

ICUC® ID: 23-DC-311 / 40y Female

Complete case data can be accessed by clicking the ***ICUC ID***

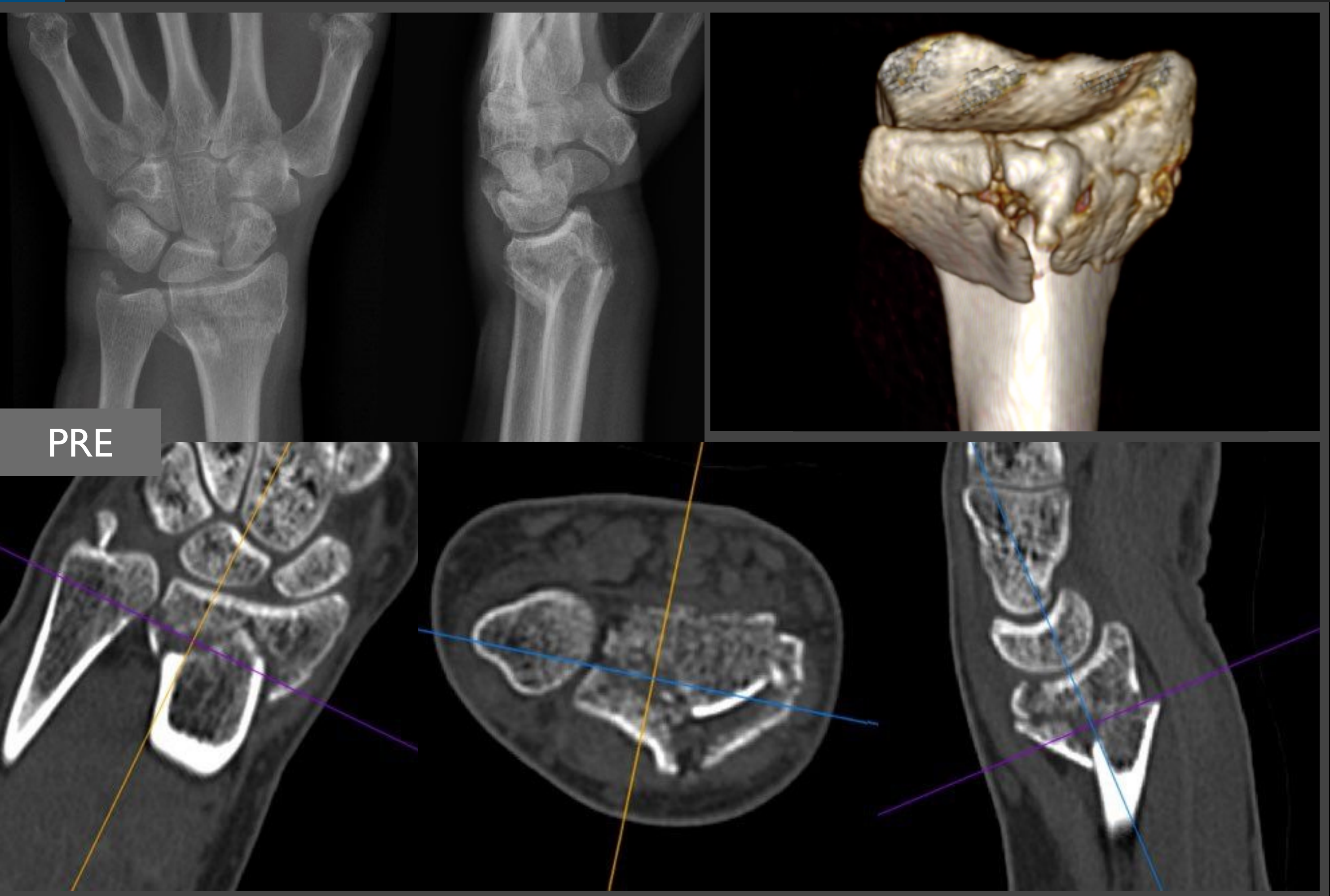

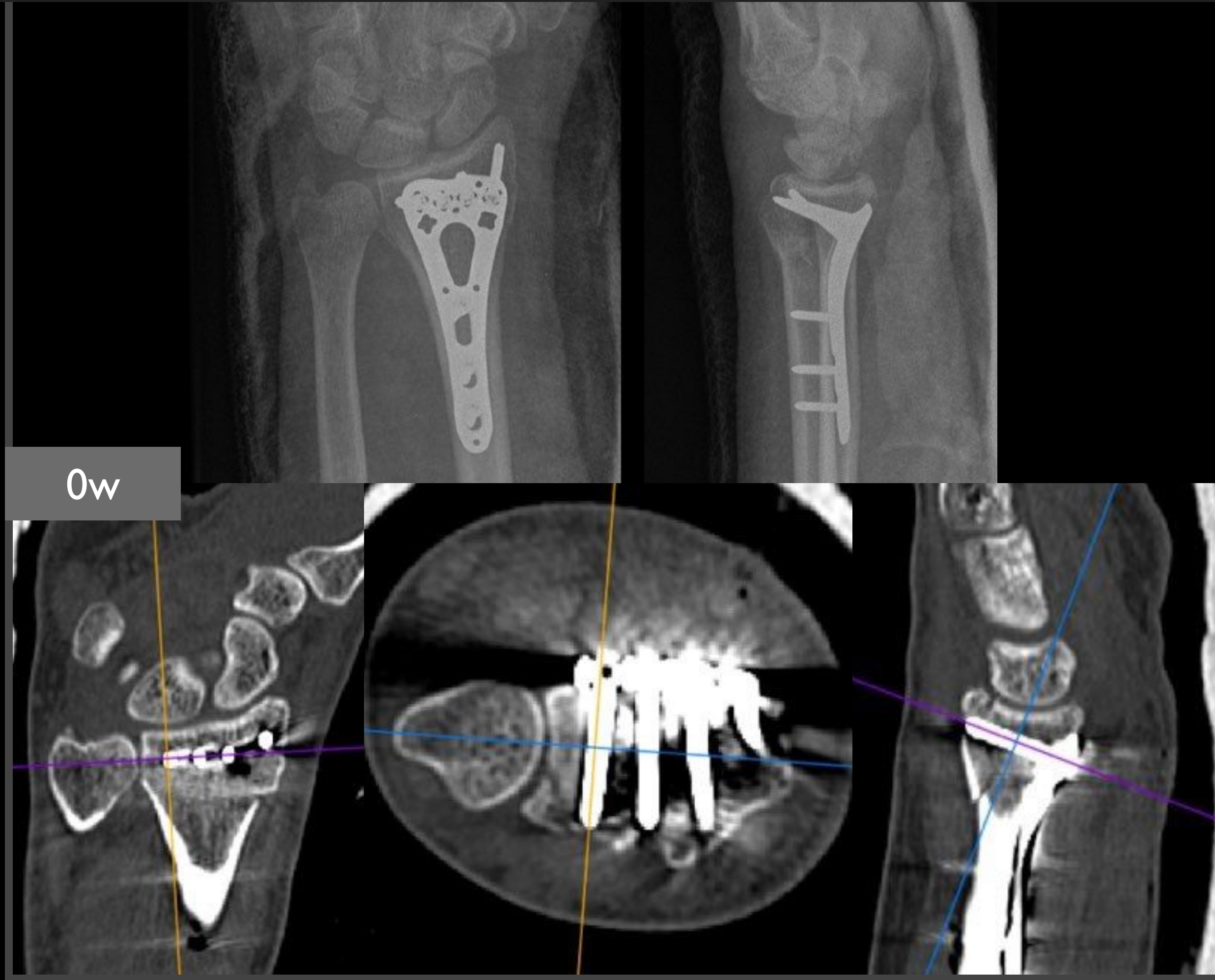

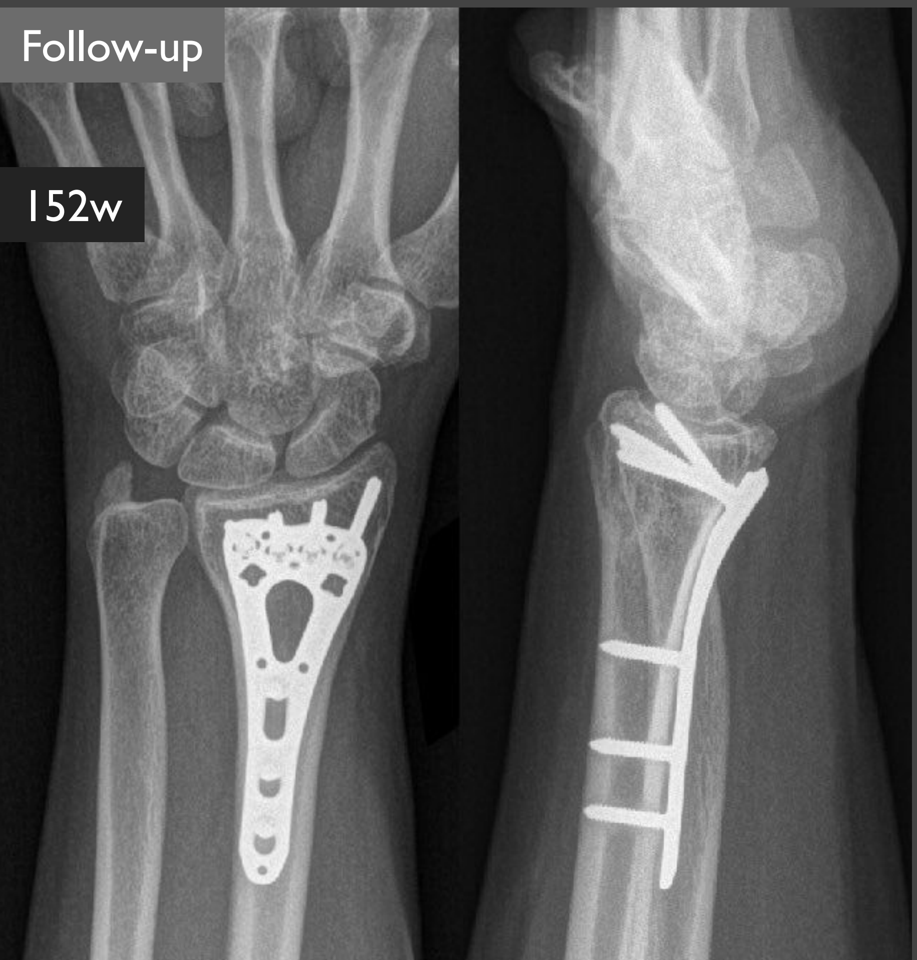

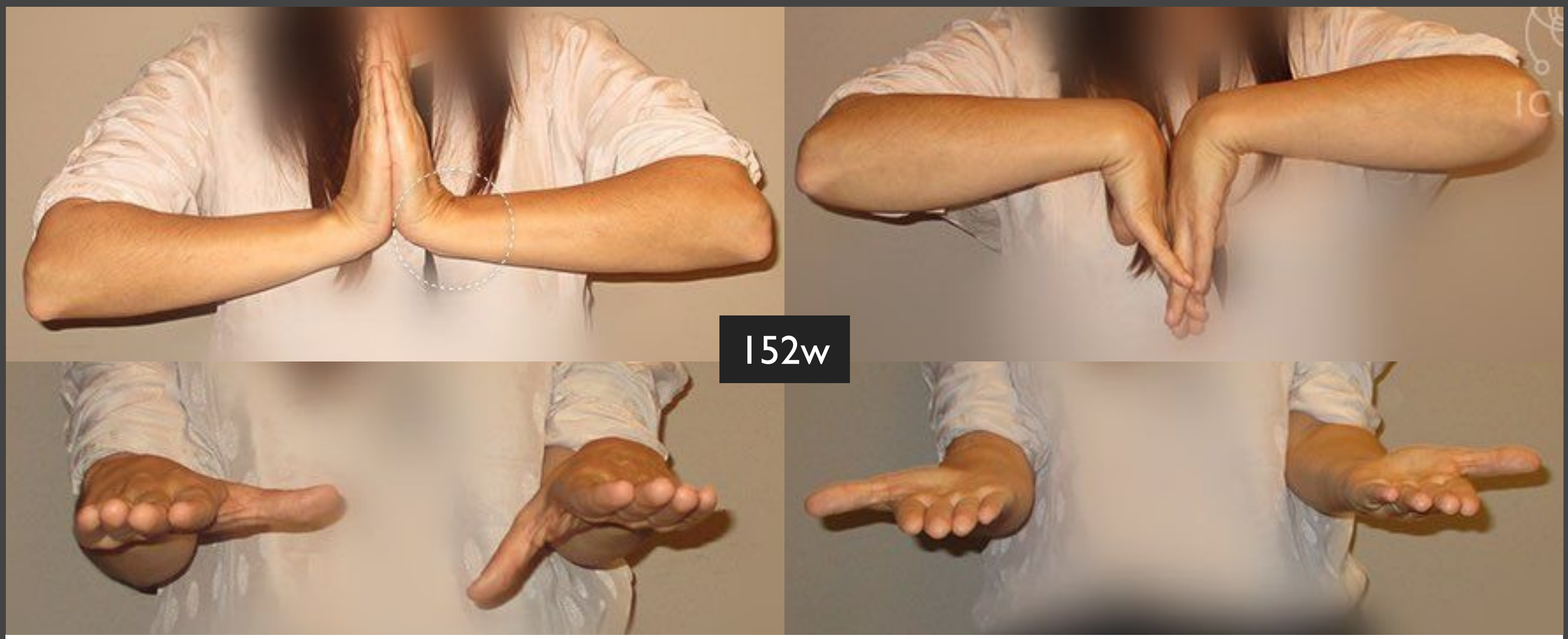

ICUC Score Functional Limitation: 0 (0-4) - Pain: 0 (0-4)

Quick DASH = 0

ICUC® ID: 23-VE-821 / 75y Female

*Complete case data can be accessed by clicking the **ICUC ID***

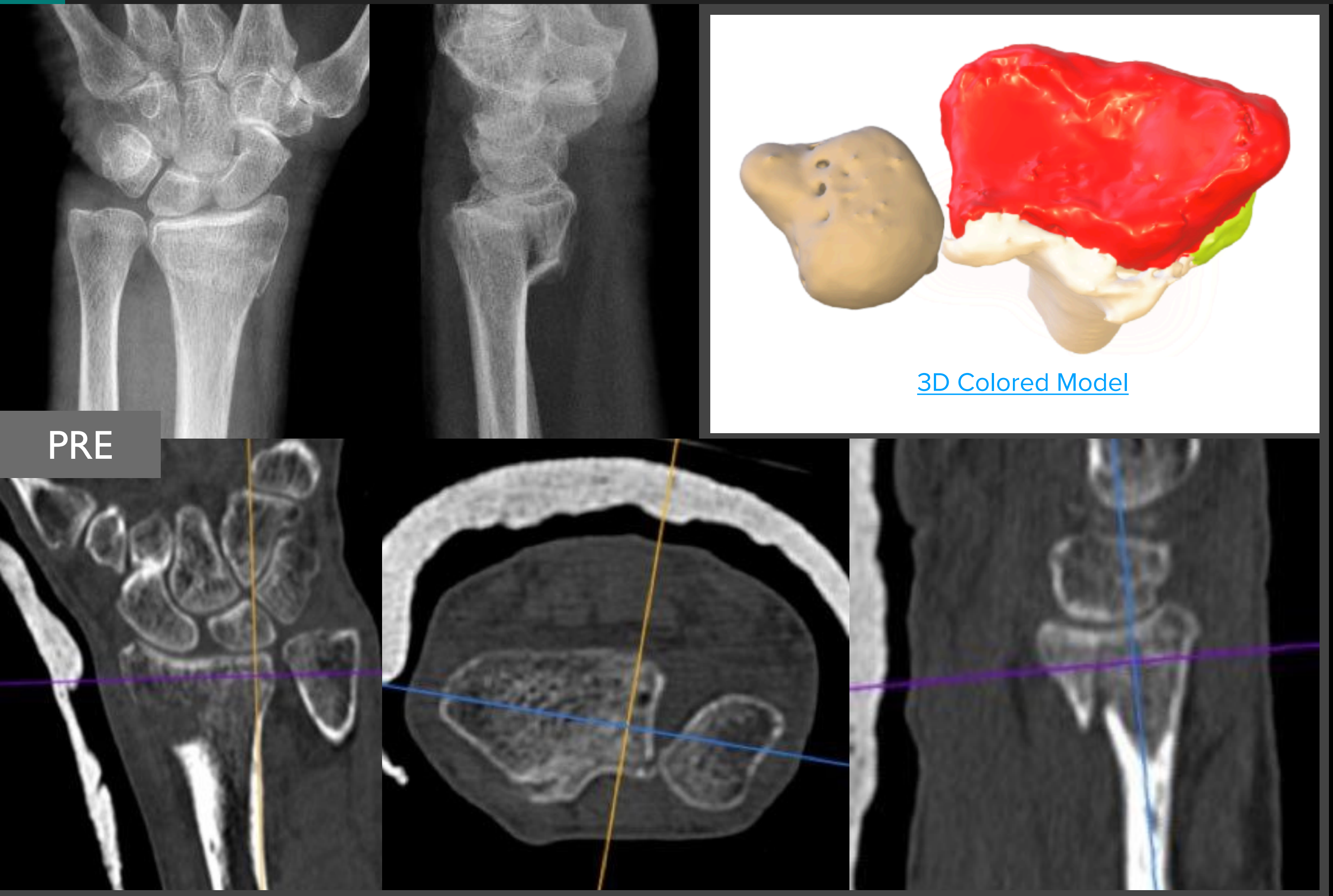

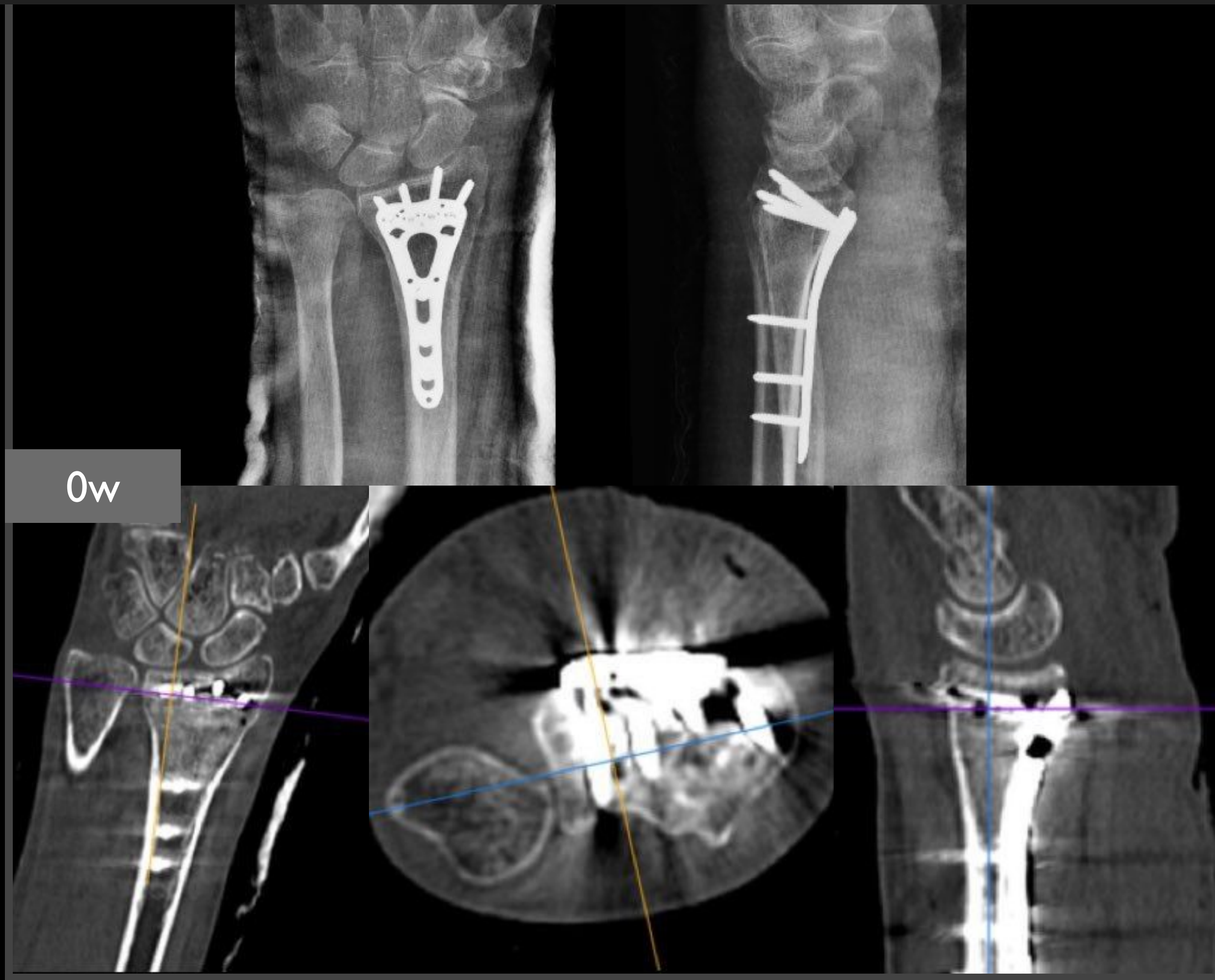

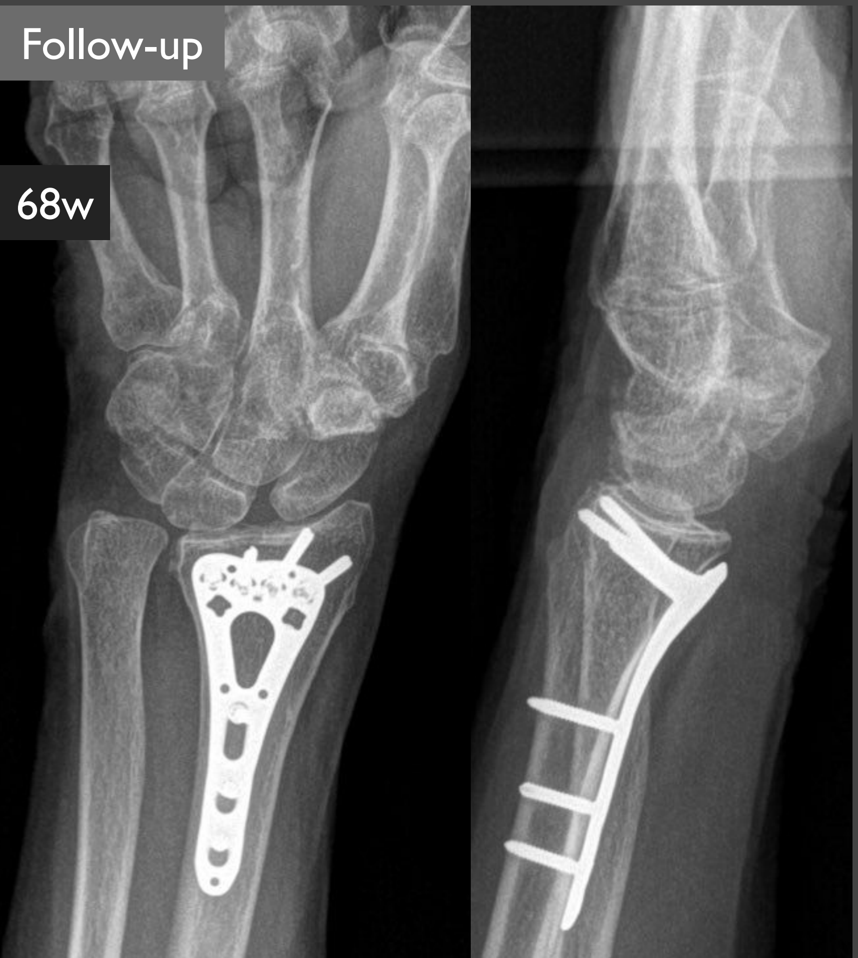

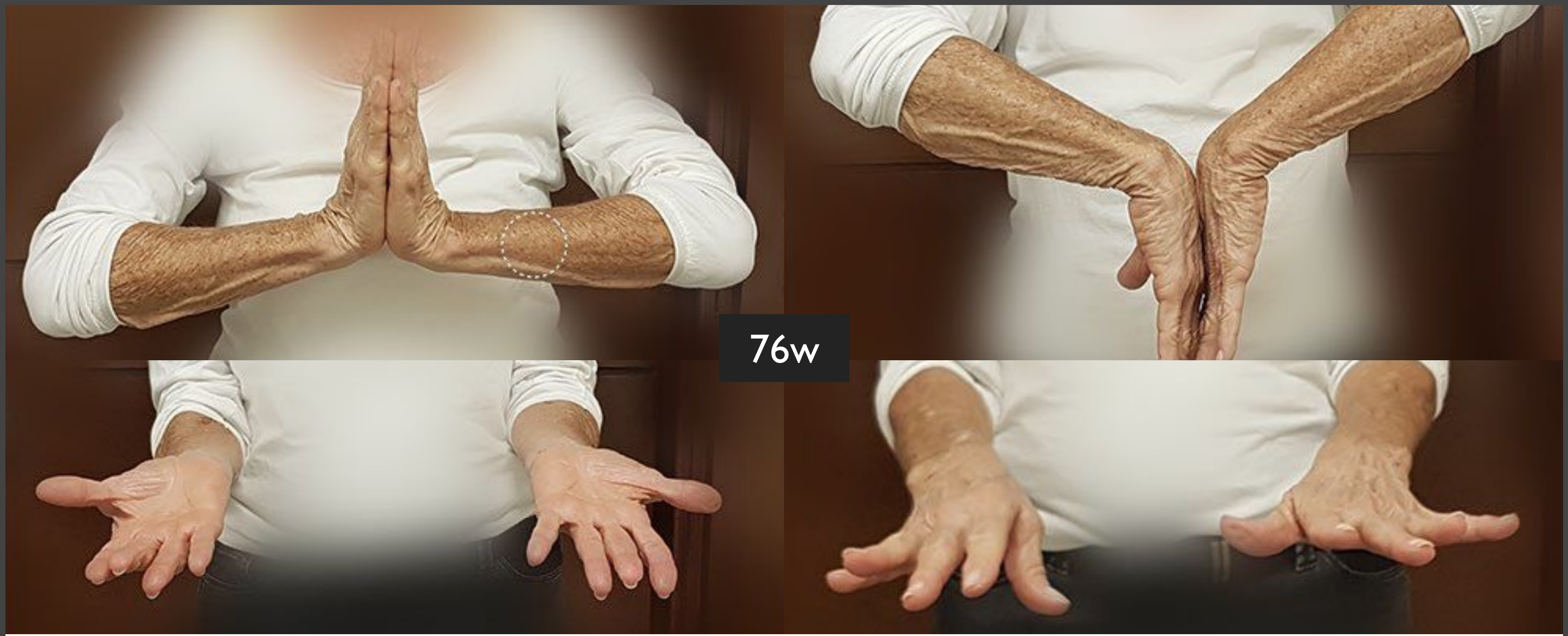

ICUC **Score** ***Functional Limitation:*** **3** (0-4) - ***Pain:*** **0** (0-4)

Quick DASH = 40

ICUC® ID: 23-DE-391 / 30y Female

Complete case data can be accessed by clicking the ***ICUC ID***

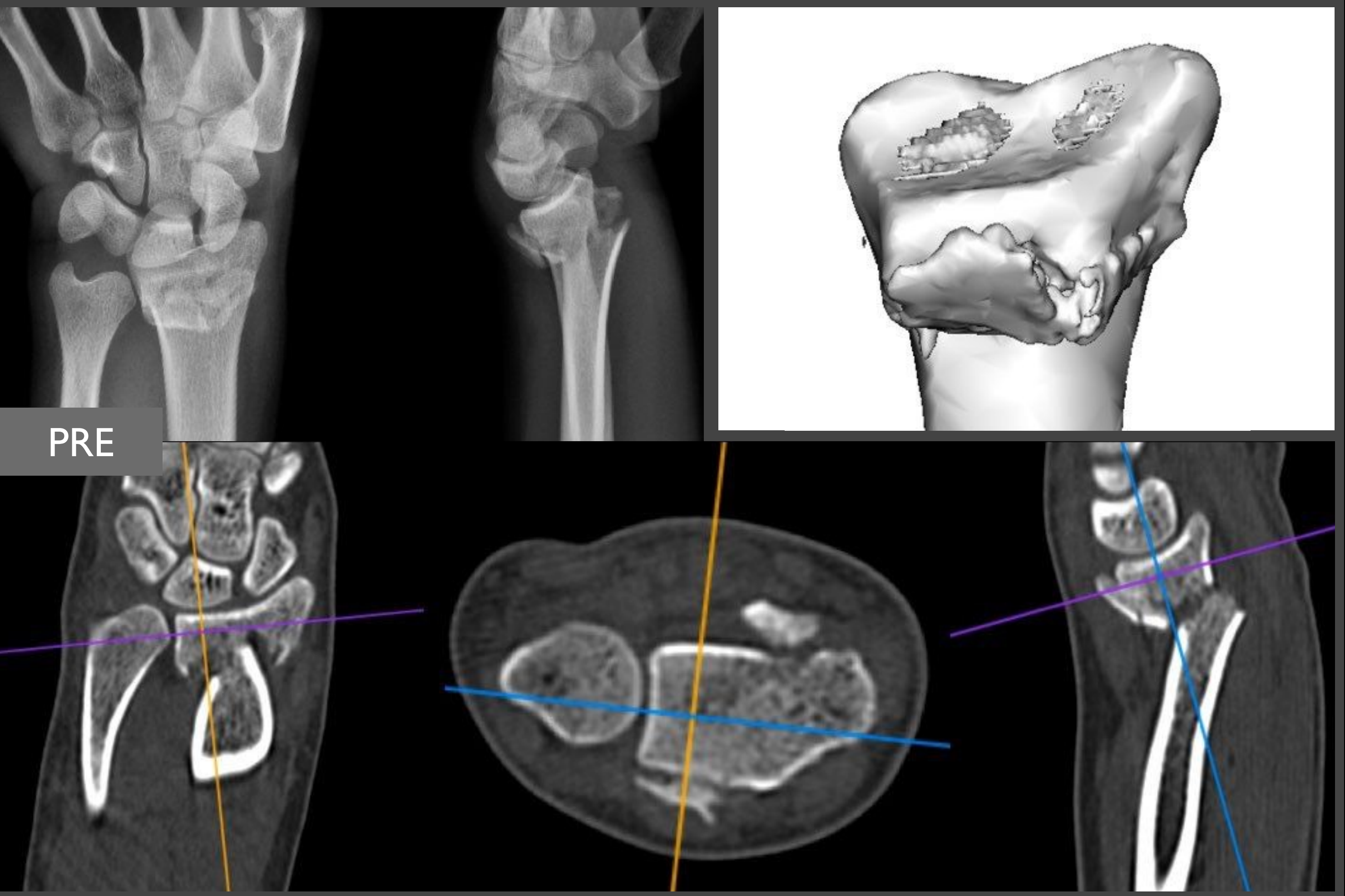

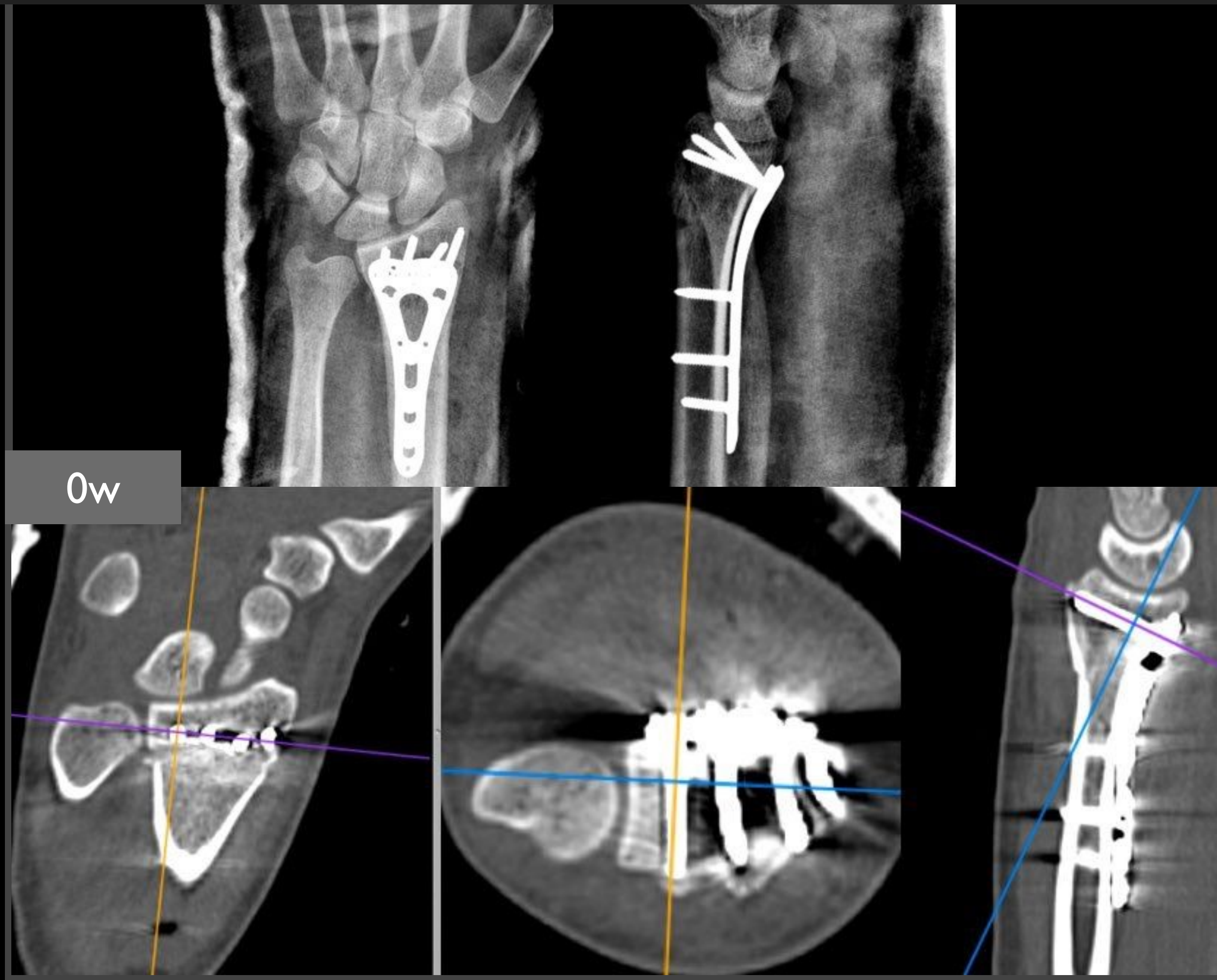

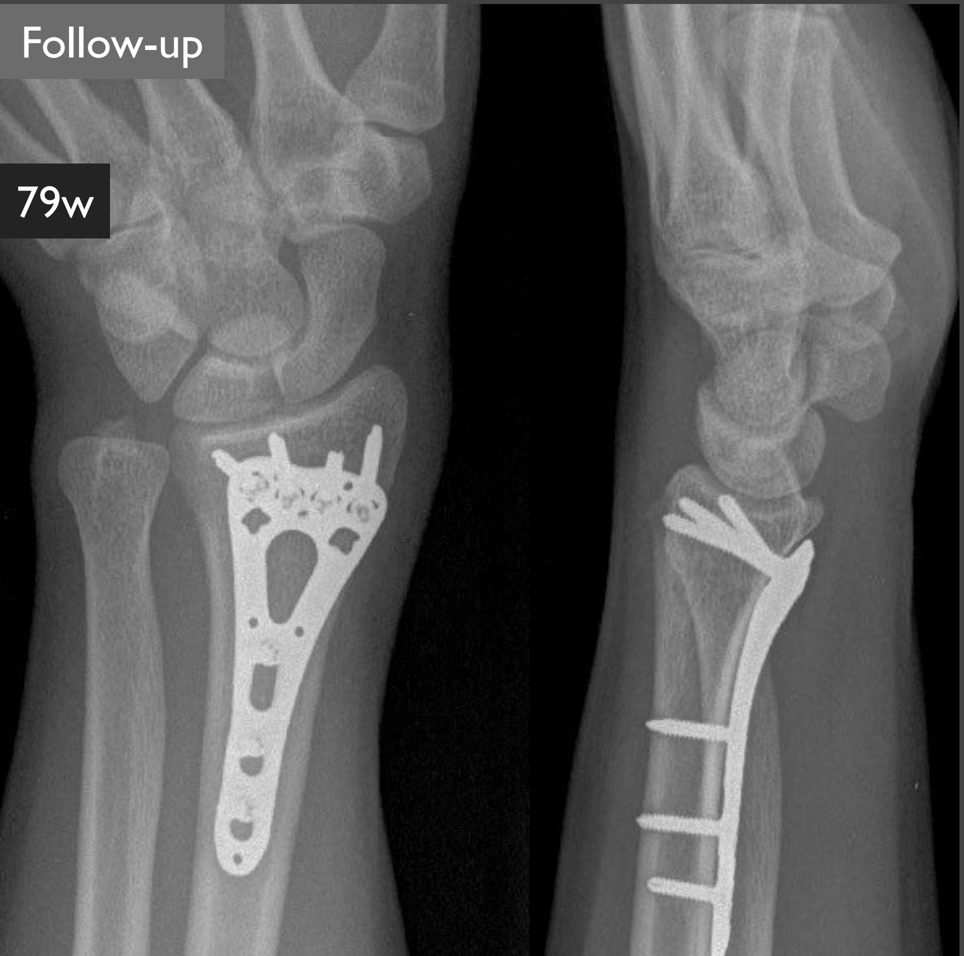

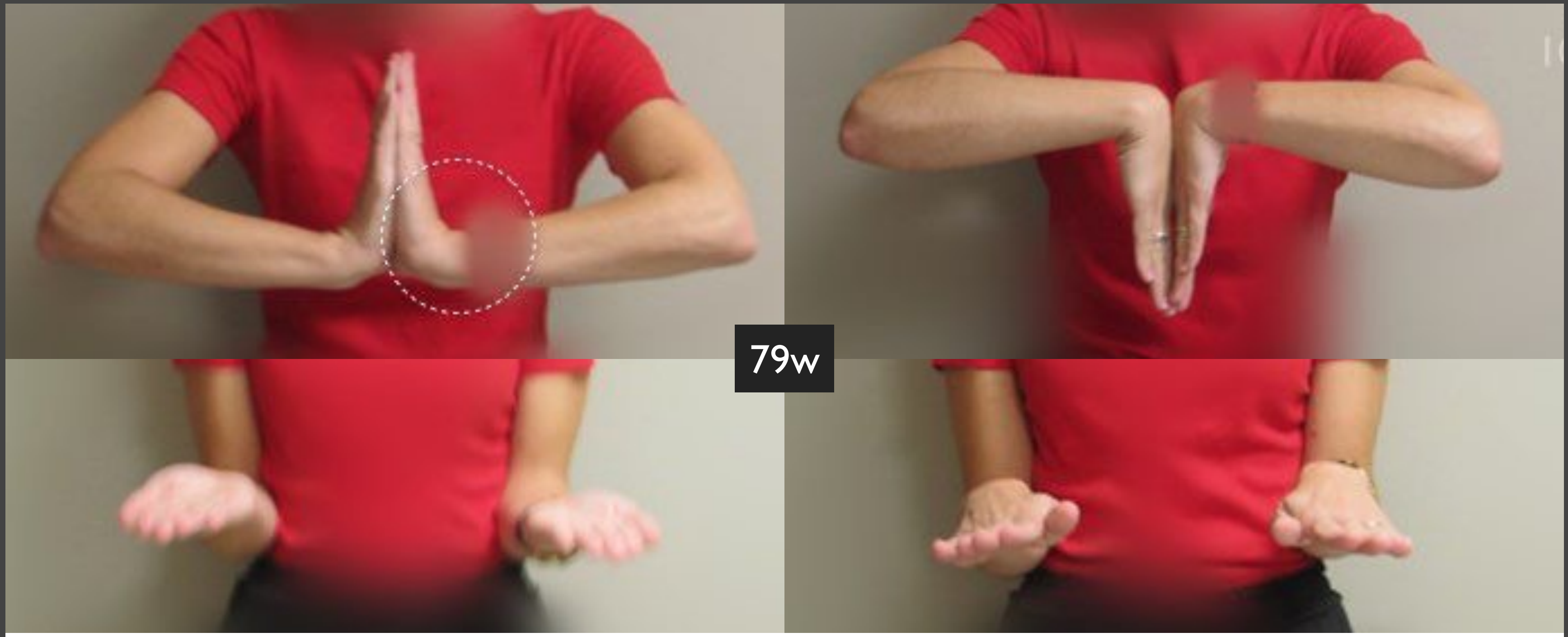

ICUC **Score** *Functional Limitation:* **0** (0-4) - *Pain:* **0** (0-4)

Quick DASH = 0

ICUC® ID: 23-DE-955 / 80y Female

Complete case data can be accessed by clicking the ***ICUC ID***

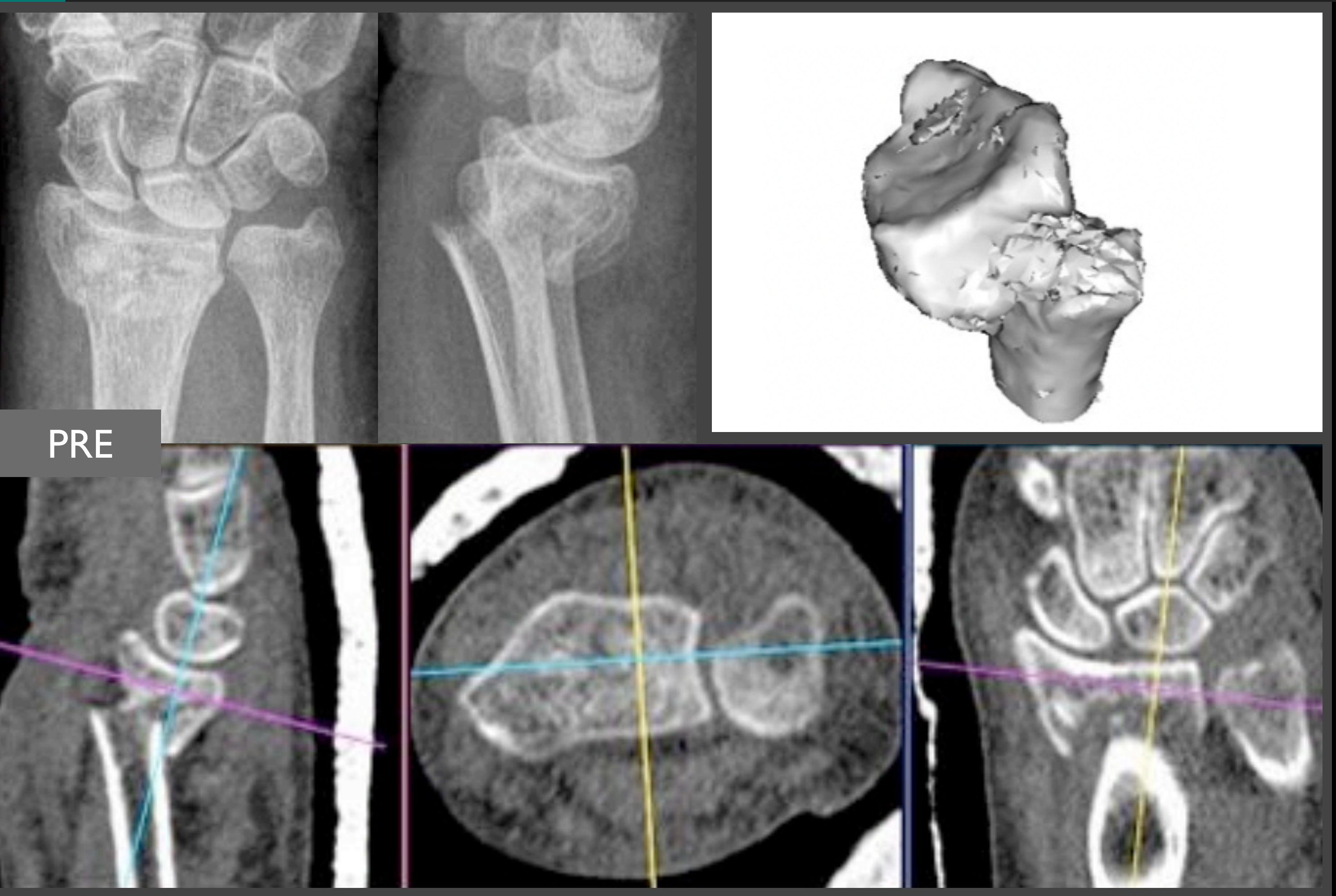

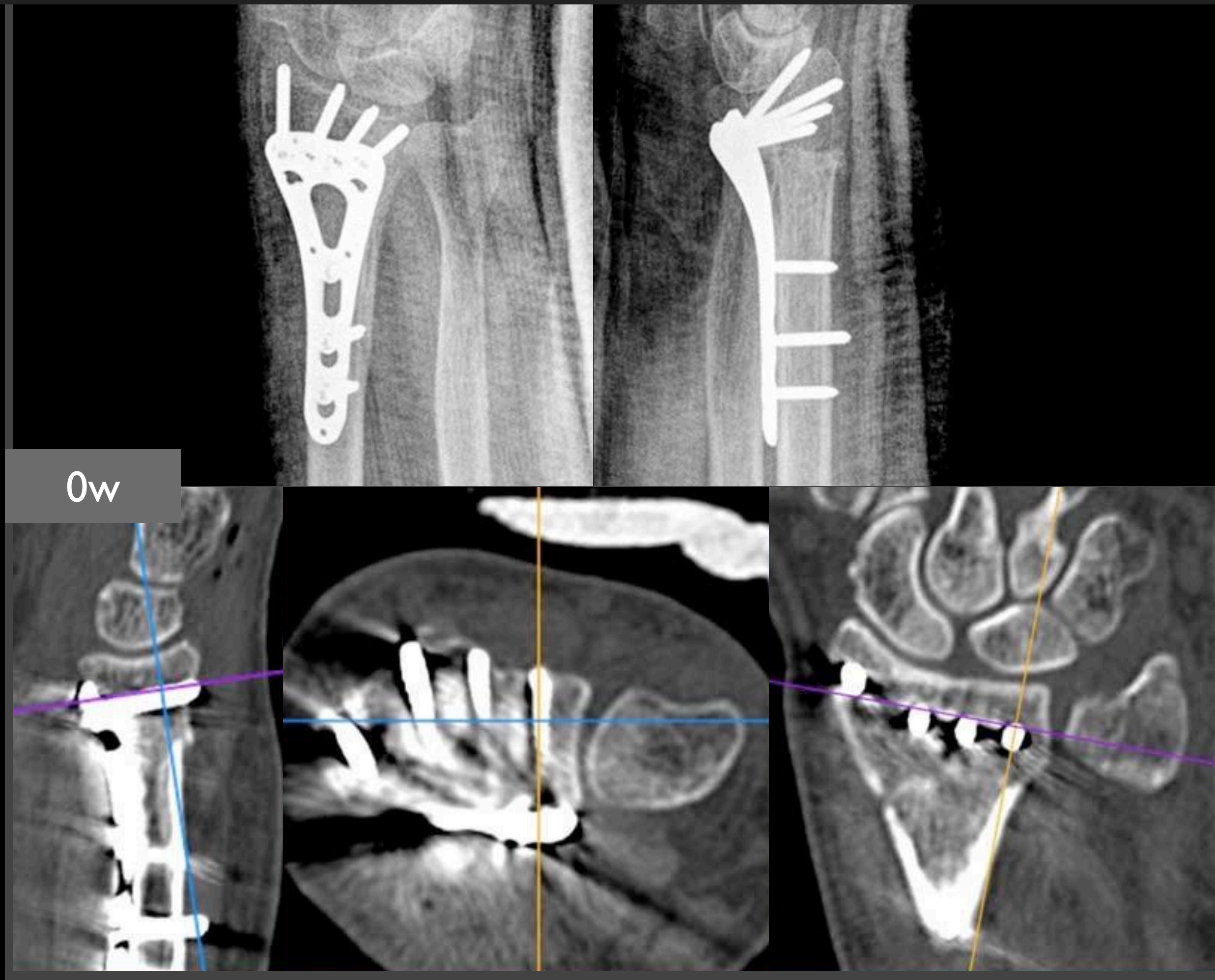

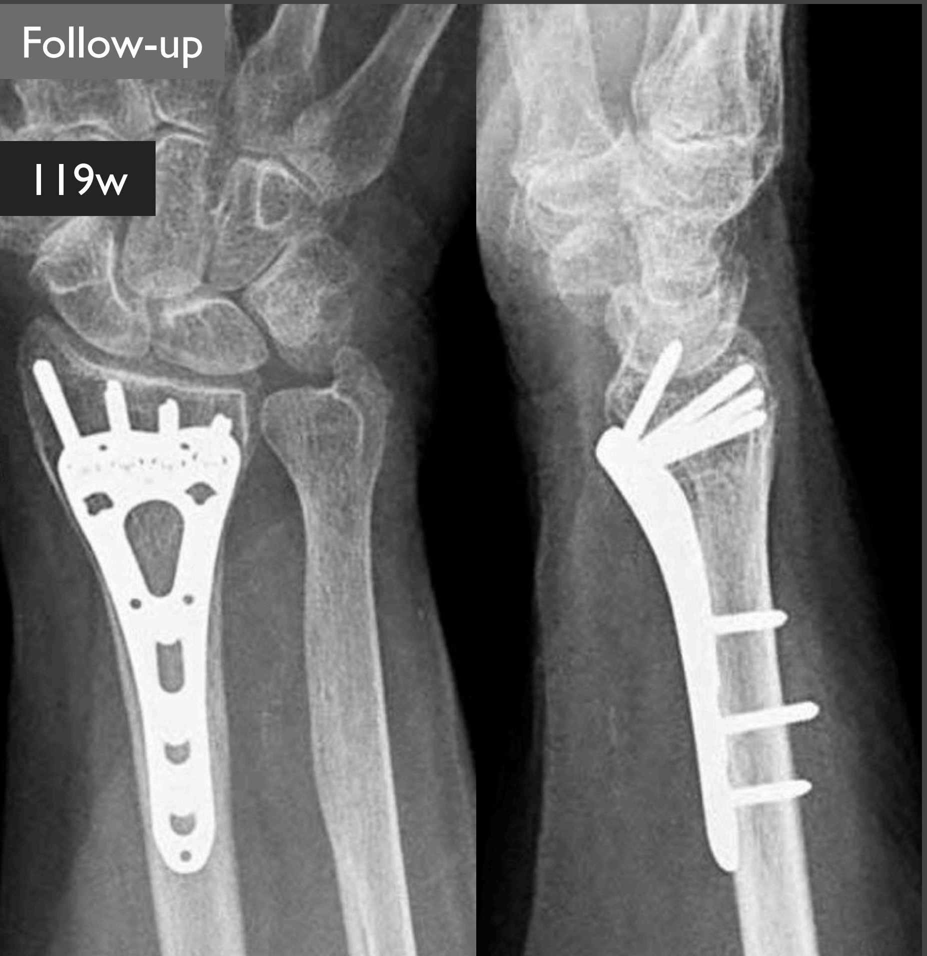

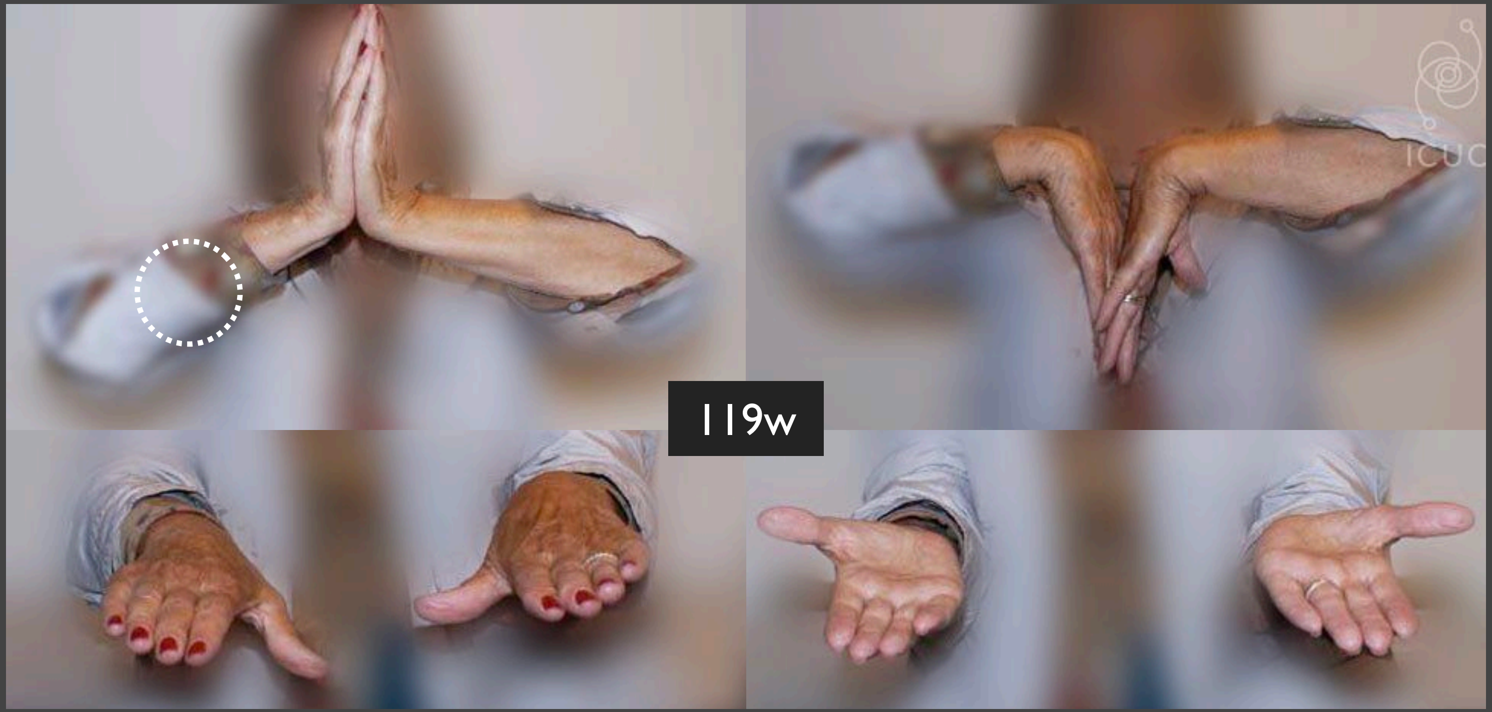

ICUC Score at 419w Functional limitation: 0 Pain: 0

Quick DASH = 0

ICUC® ID: 23-DU-569 / 85y Female

Complete case data can be accessed by clicking the ***ICUC ID***

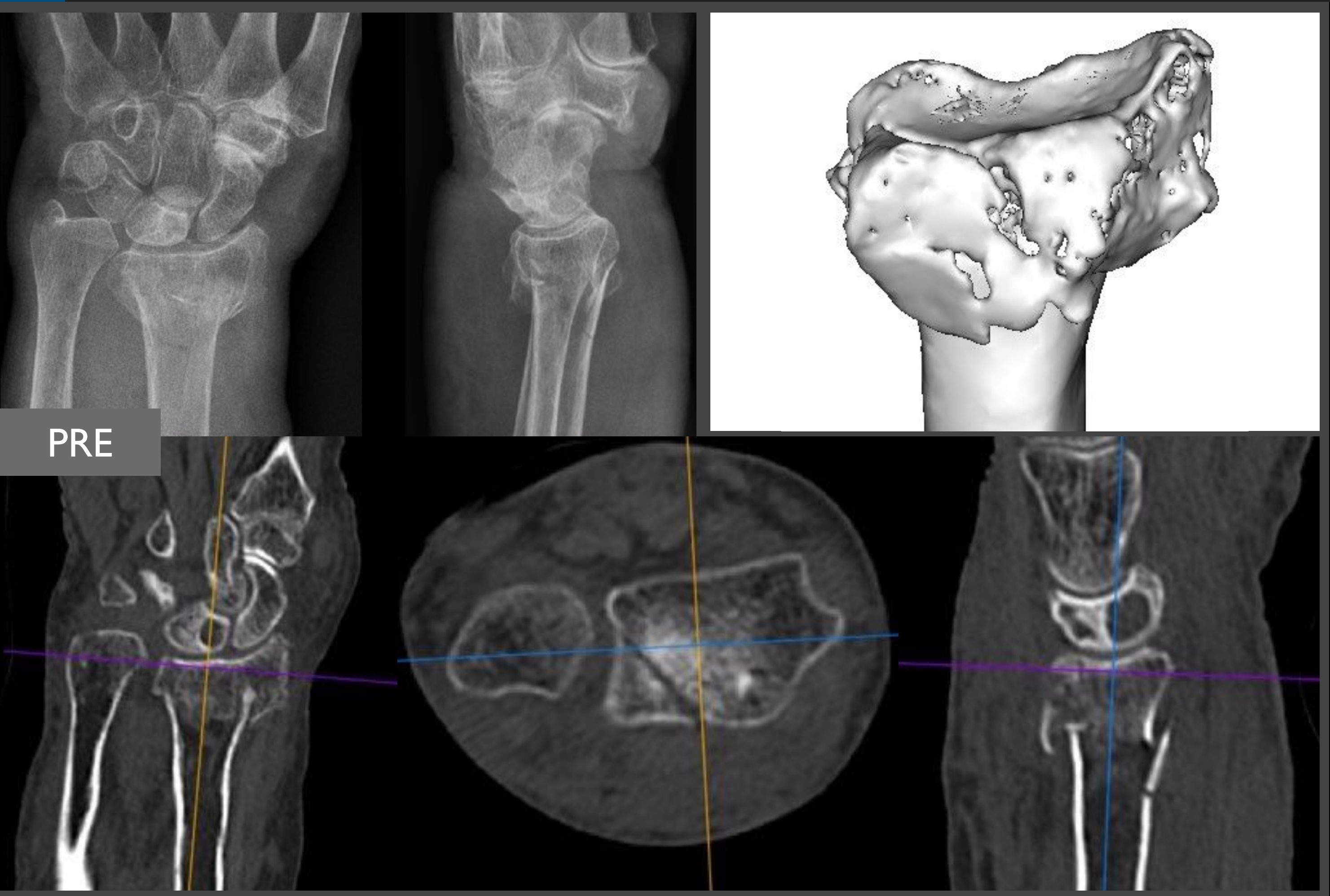

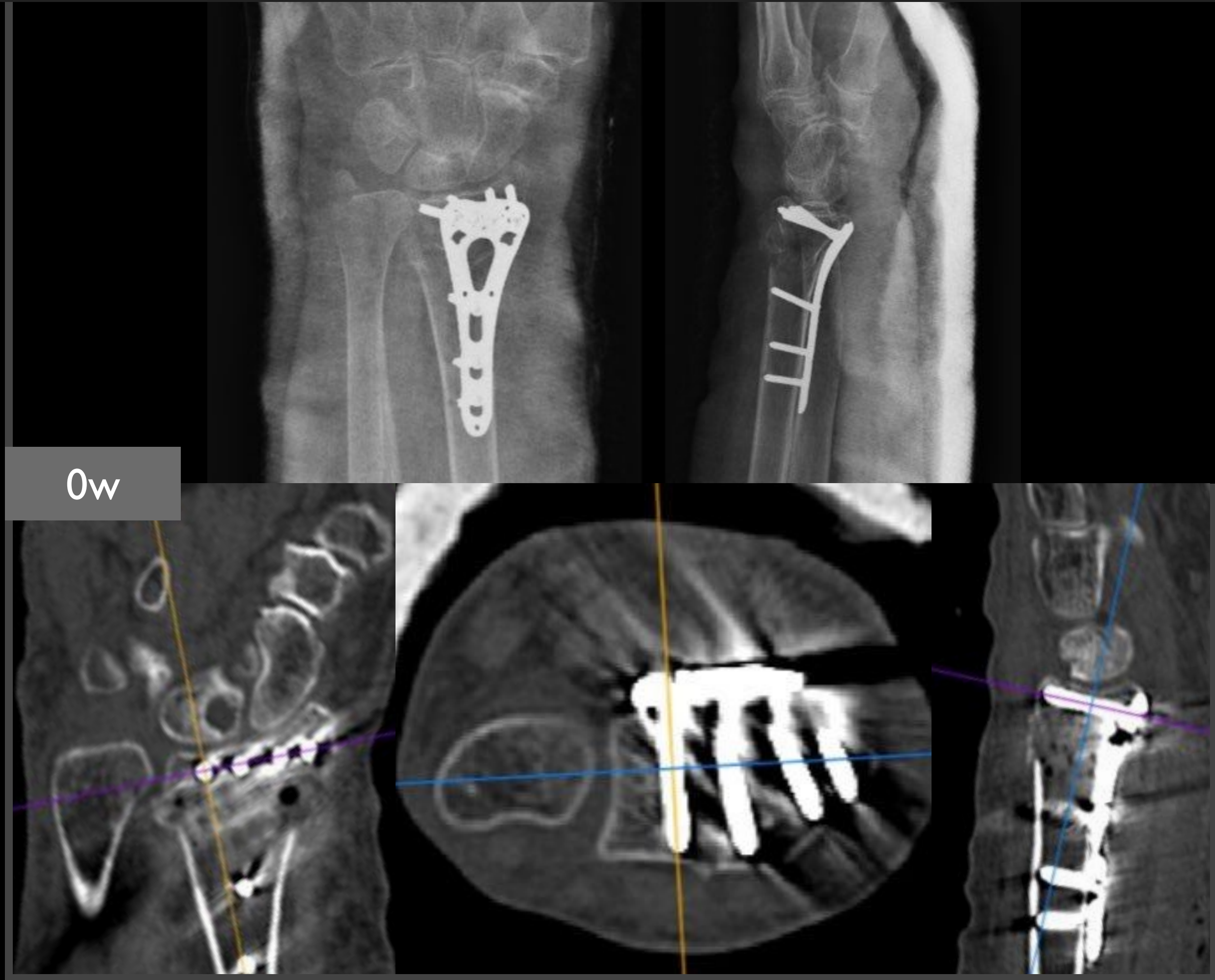

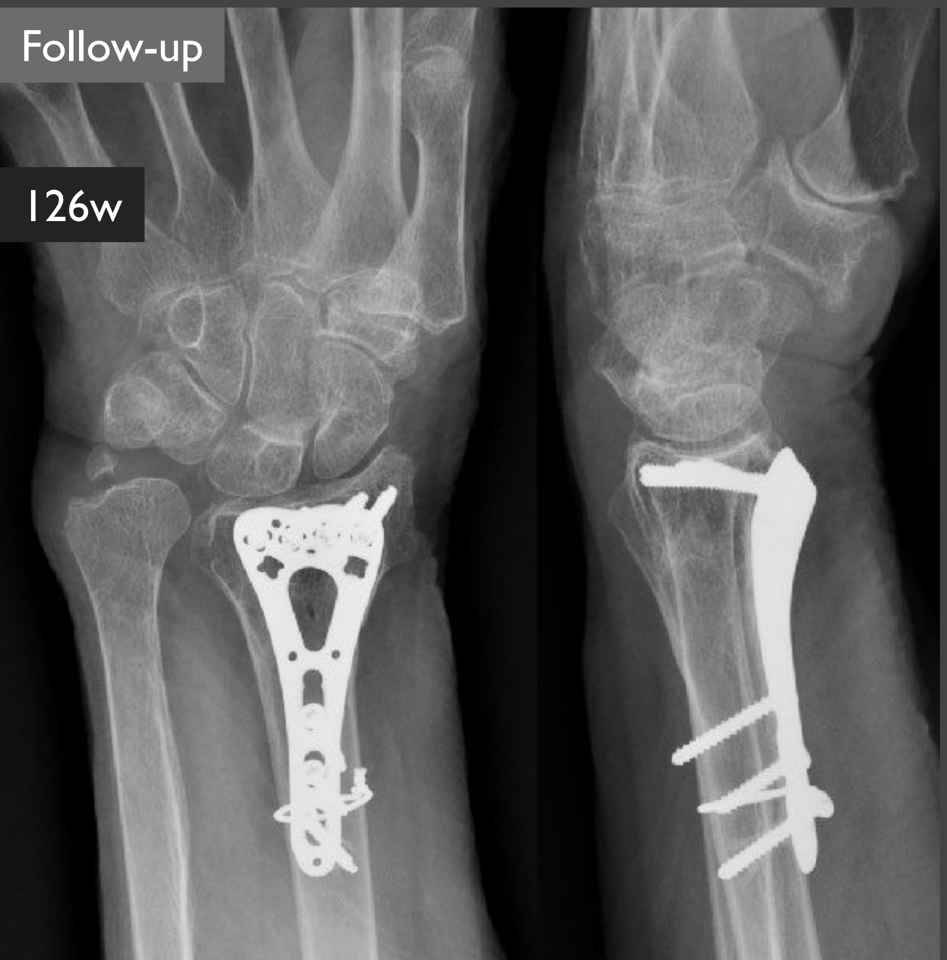

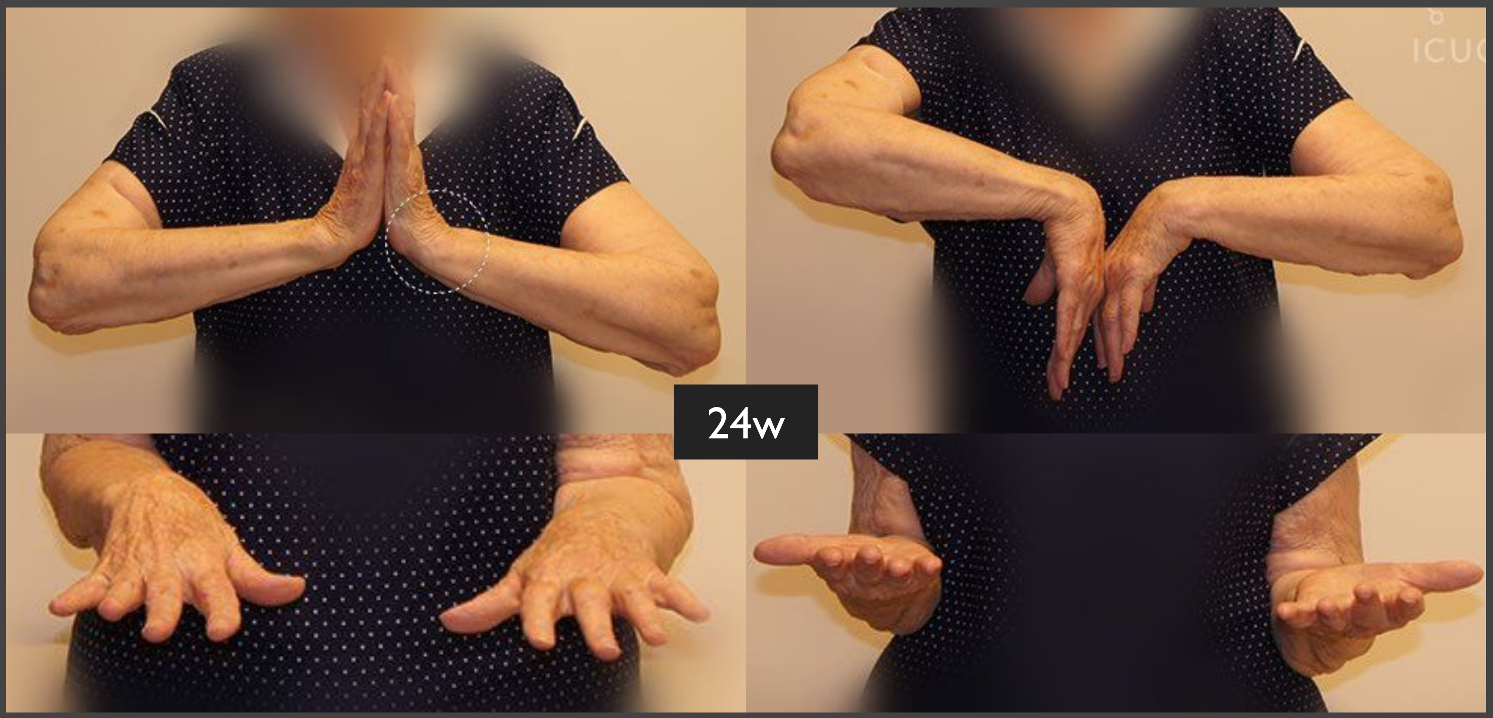

ICUC Score at 183w Functional limitation: 1 Pain: 0

Quick DASH = 3

ICUC® ID: 23-DS-041 / 60y Female

Complete case data can be accessed by clicking the ***ICUC ID***

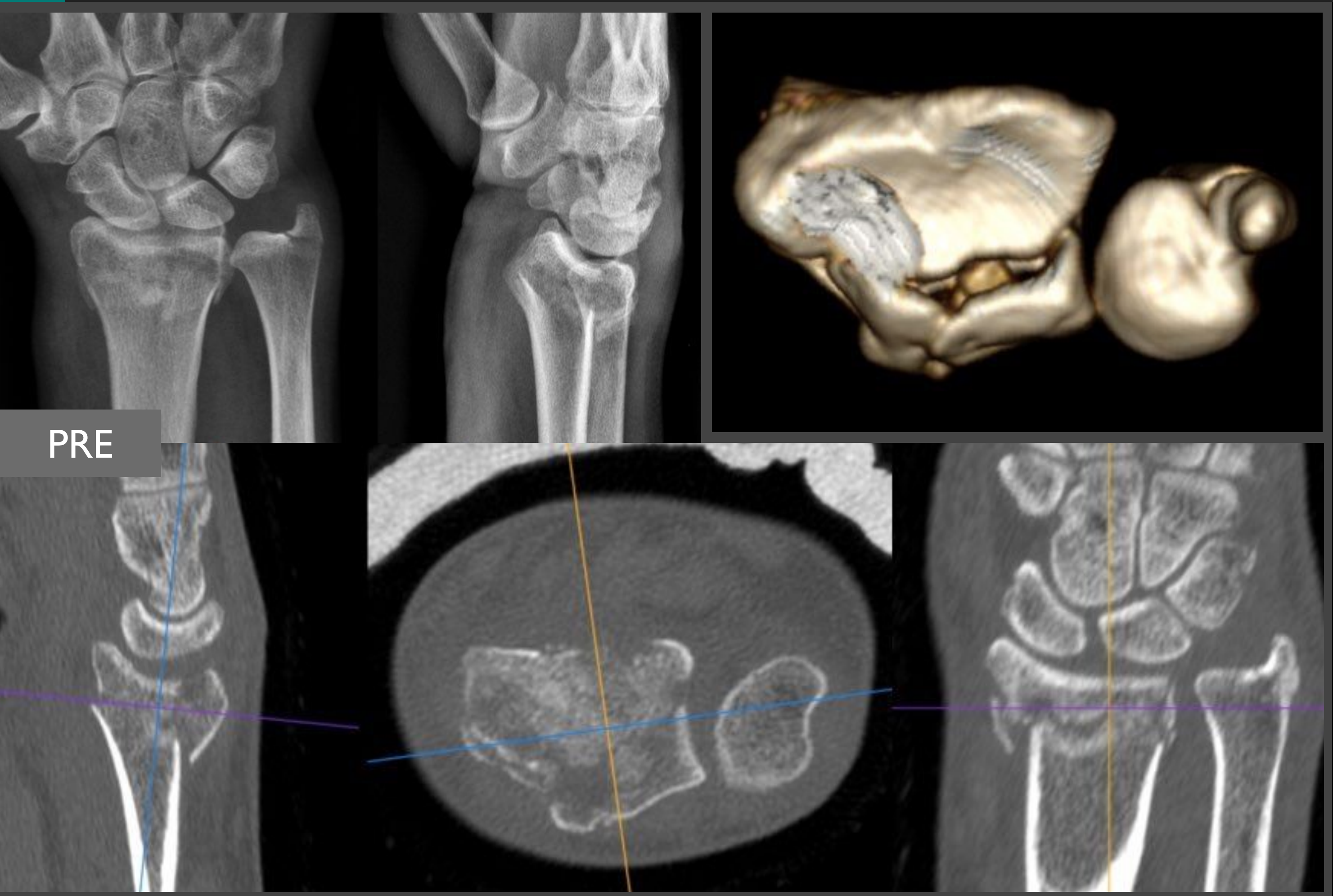

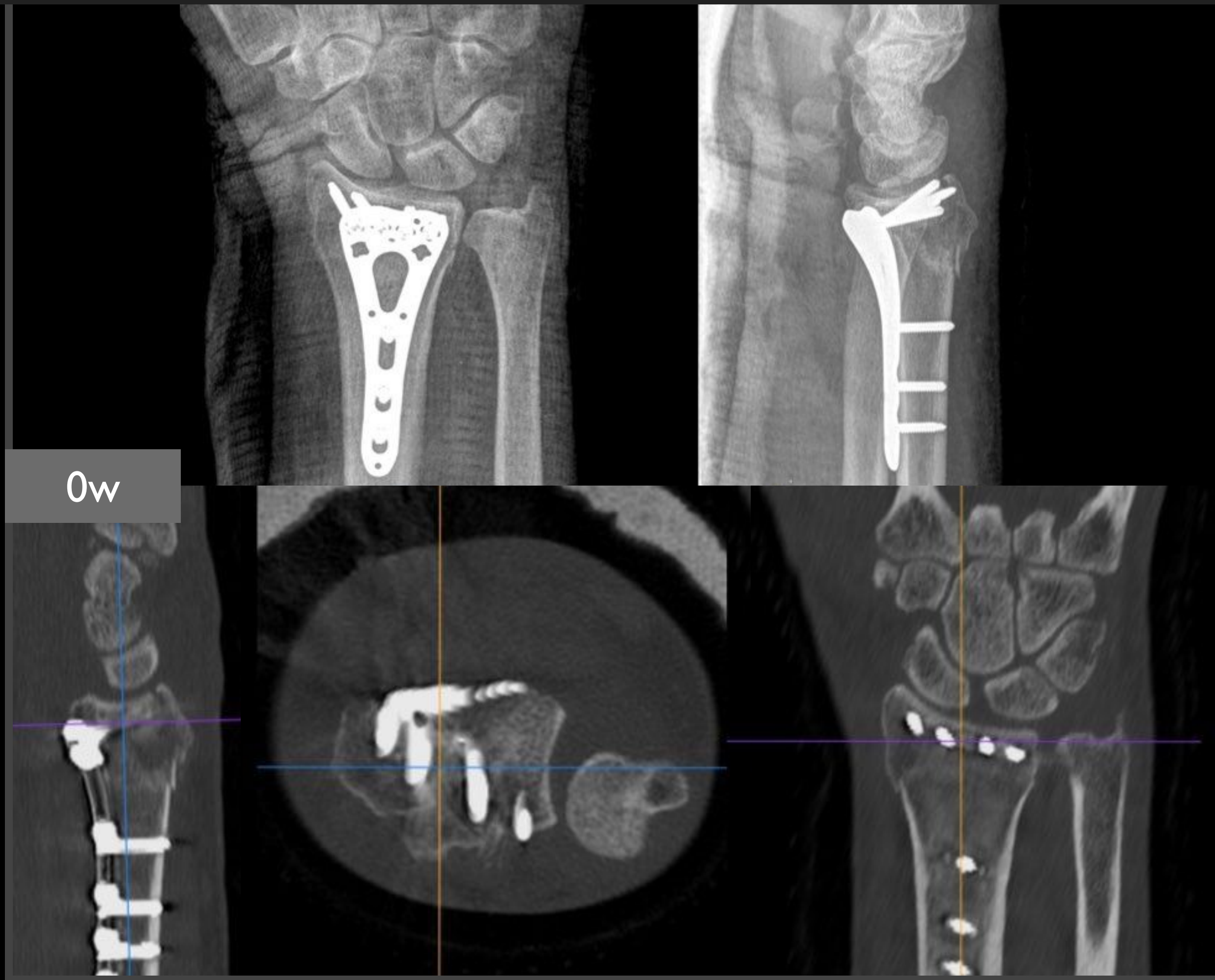

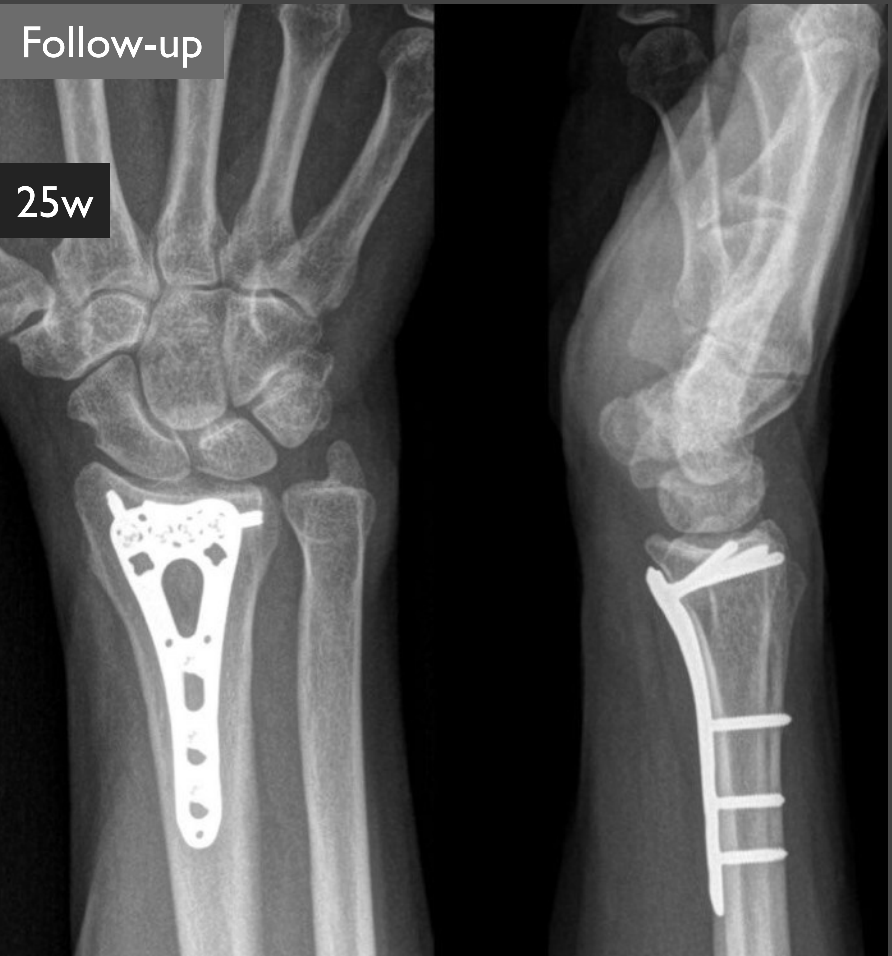

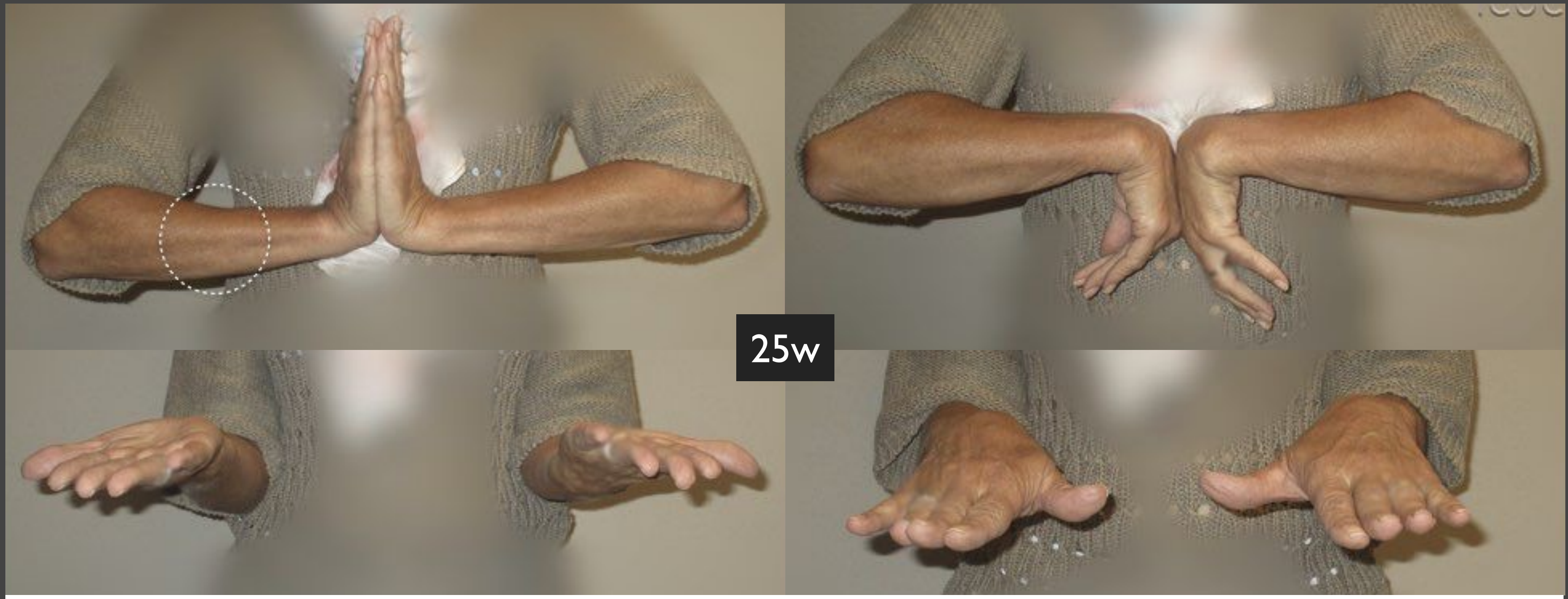

ICUC **Score** **Functional Limitation: 0** (0-4) - **Pain: 0** (0-4)

Quick DASH = 3

ICUC® ID: 23-DI-237 / 65y Female

*Complete case data can be accessed by clicking the **ICUC ID***

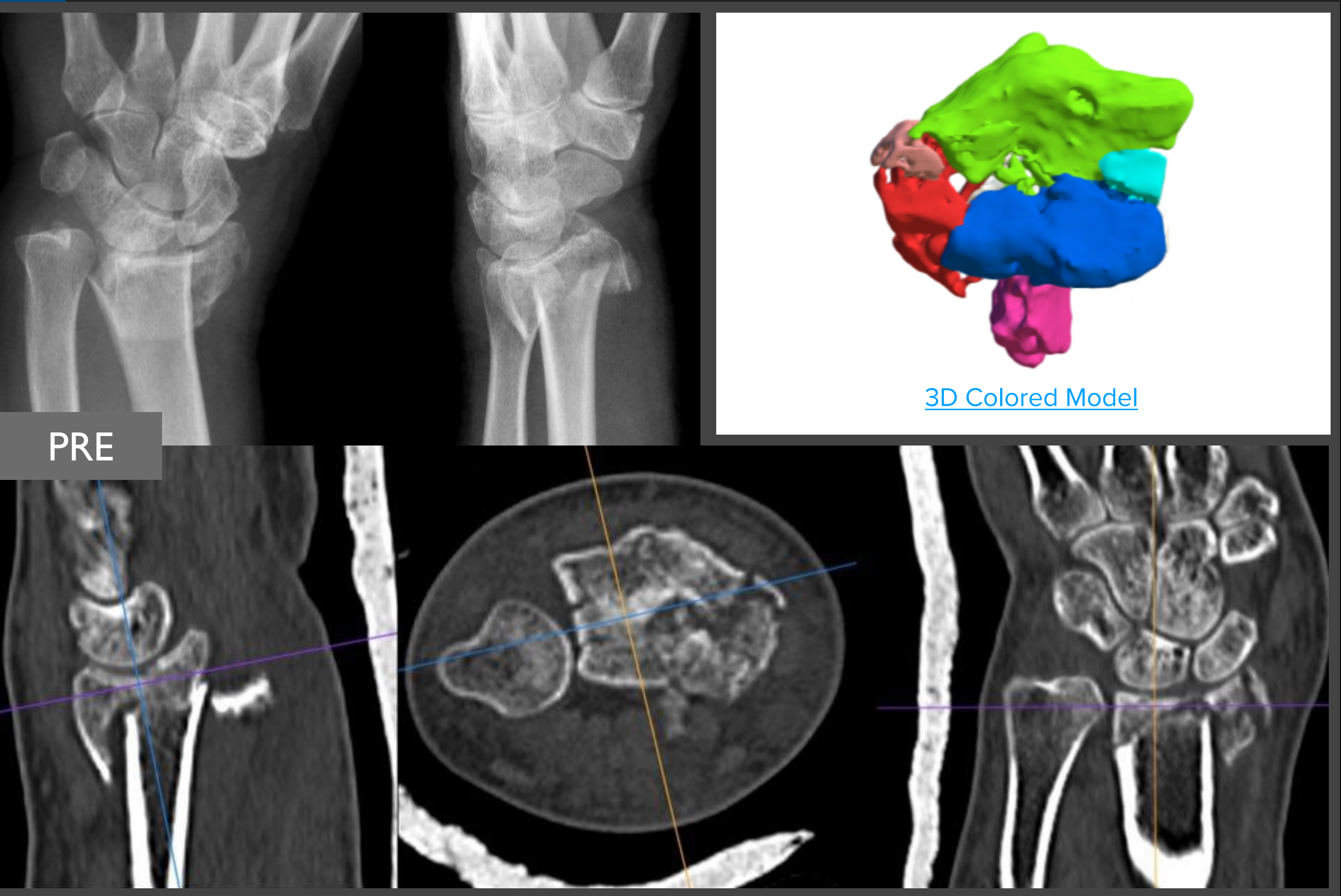

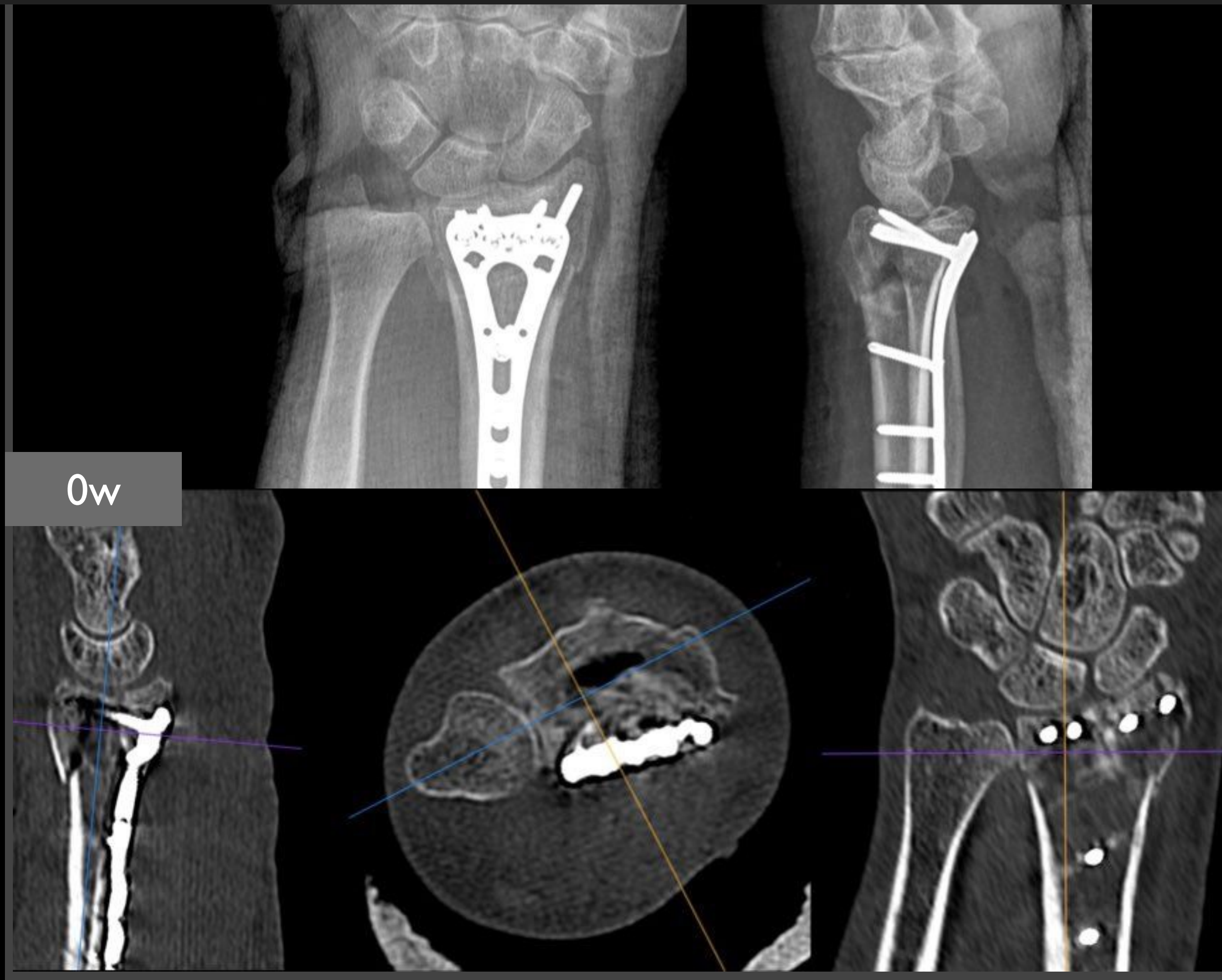

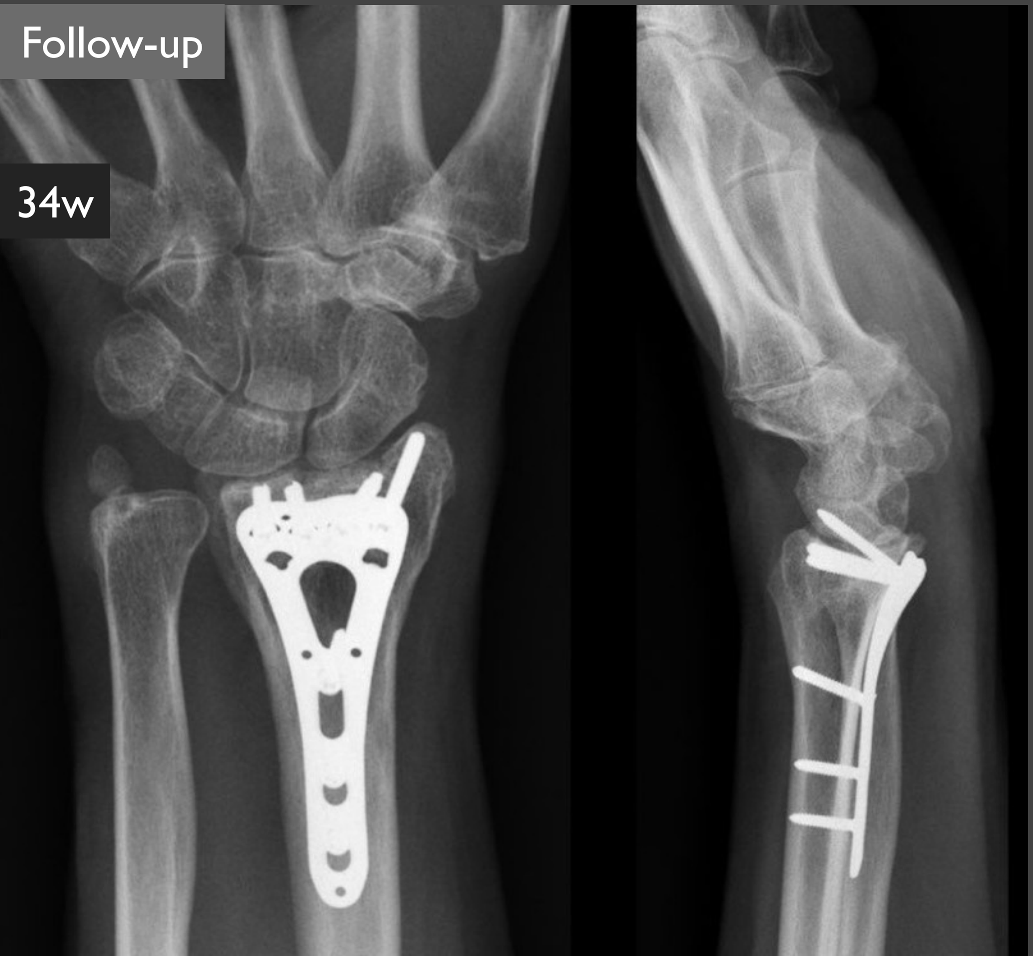

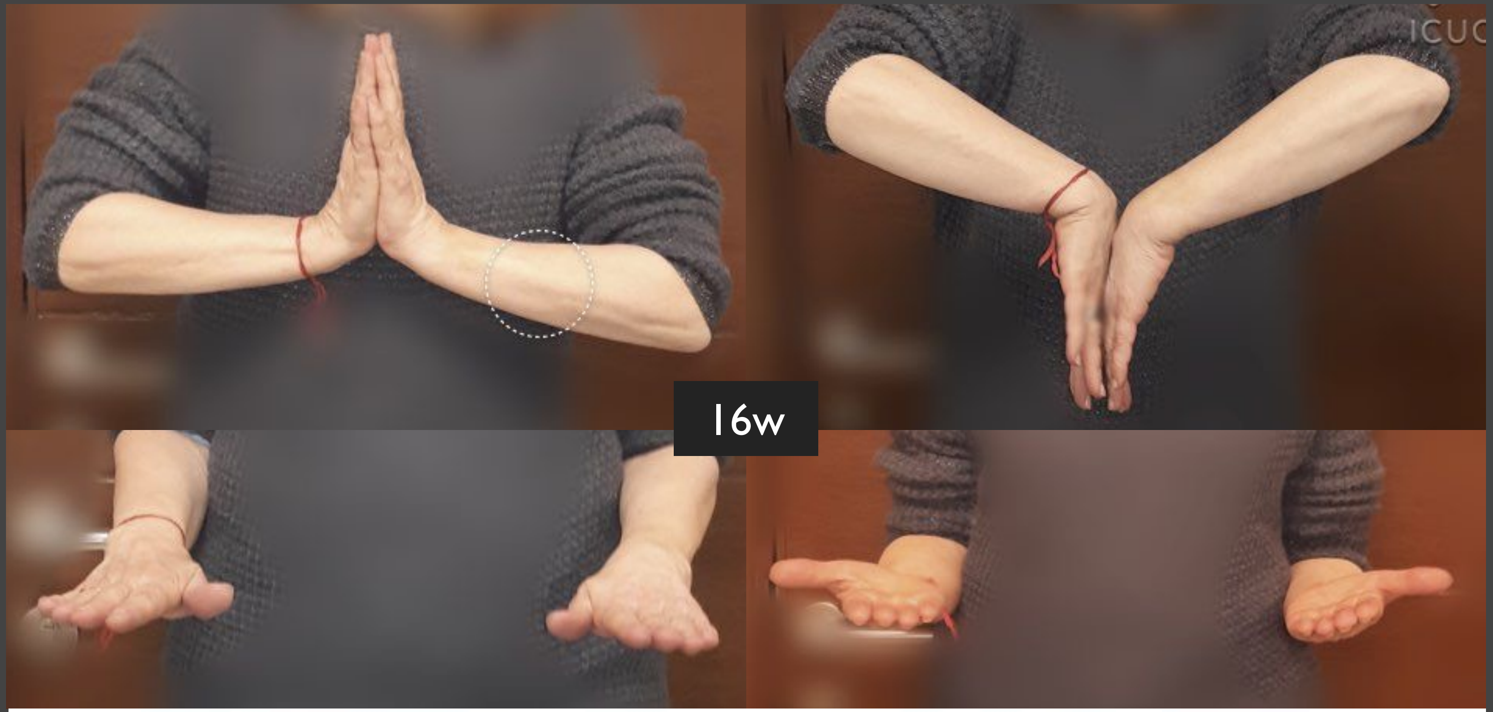

ICUC **Score** ***Functional Limitation:*** **1** (0-4) - ***Pain:*** **1** (0-4)

Quick DASH = 22

ICUC® ID: 23-DE-123 / 65y Female

*Complete case data can be accessed by clicking the **ICUC ID***

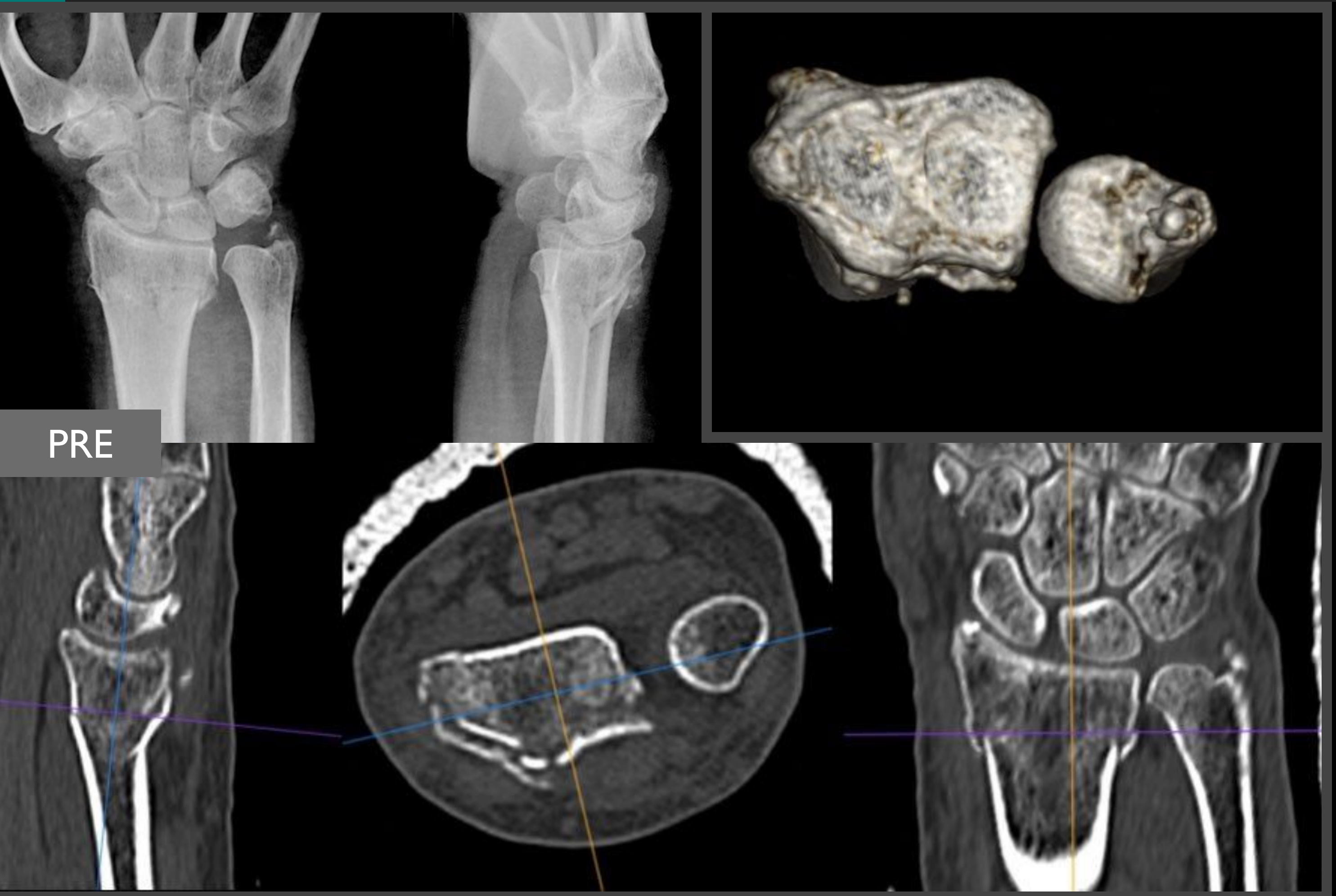

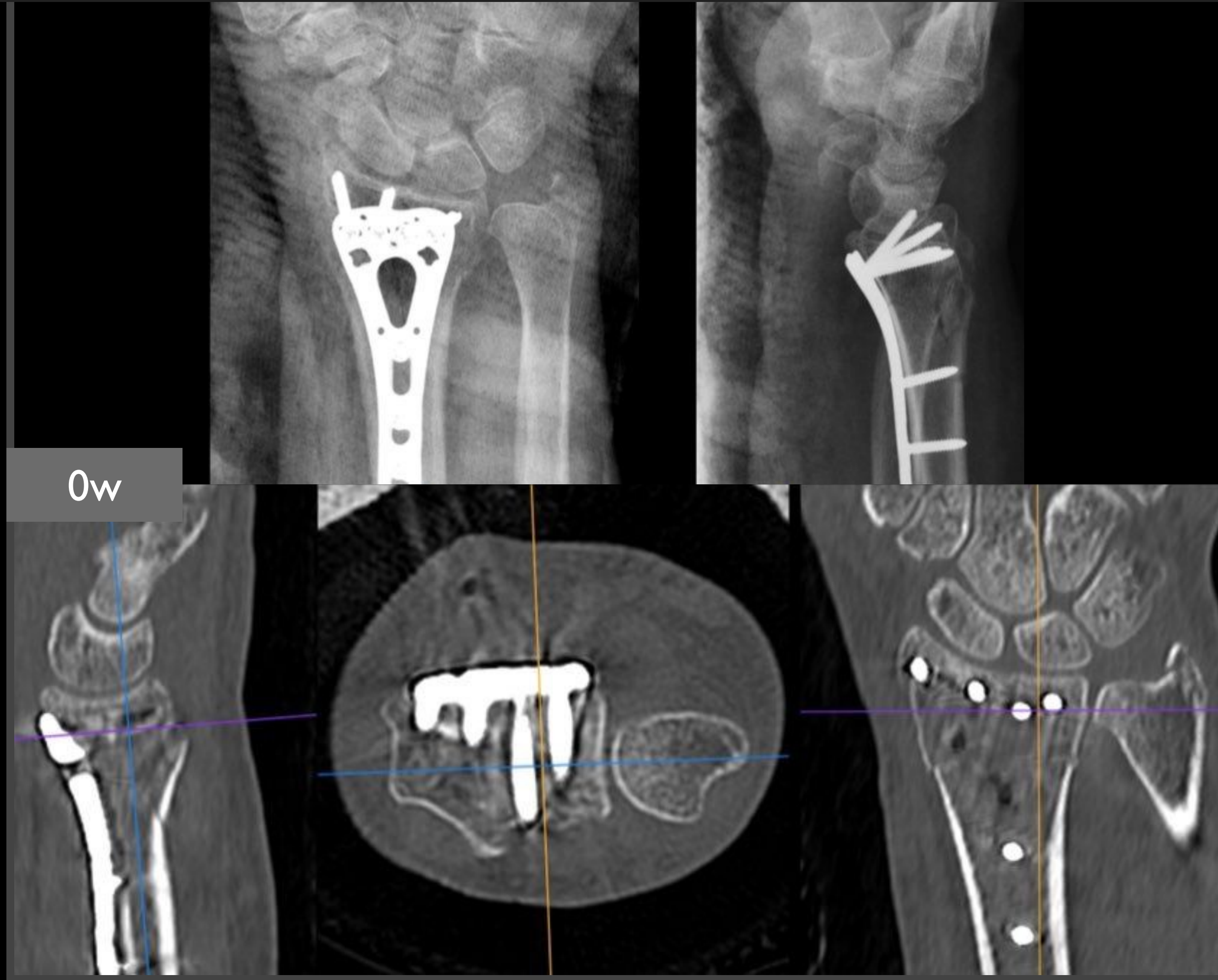

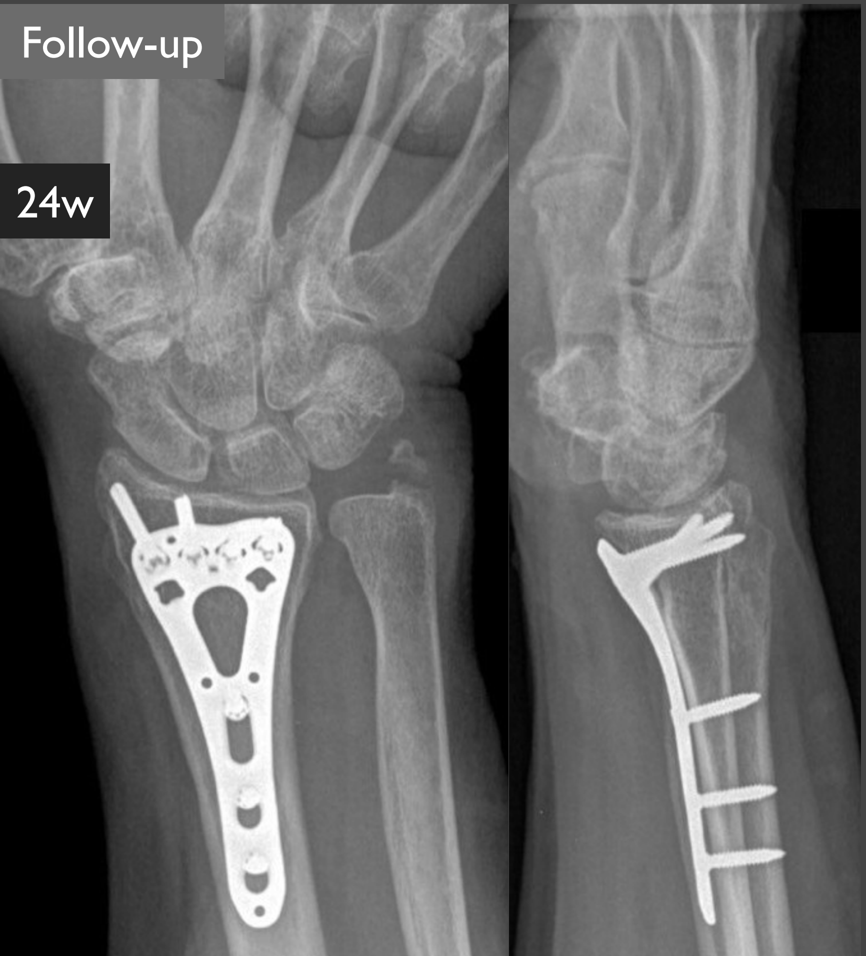

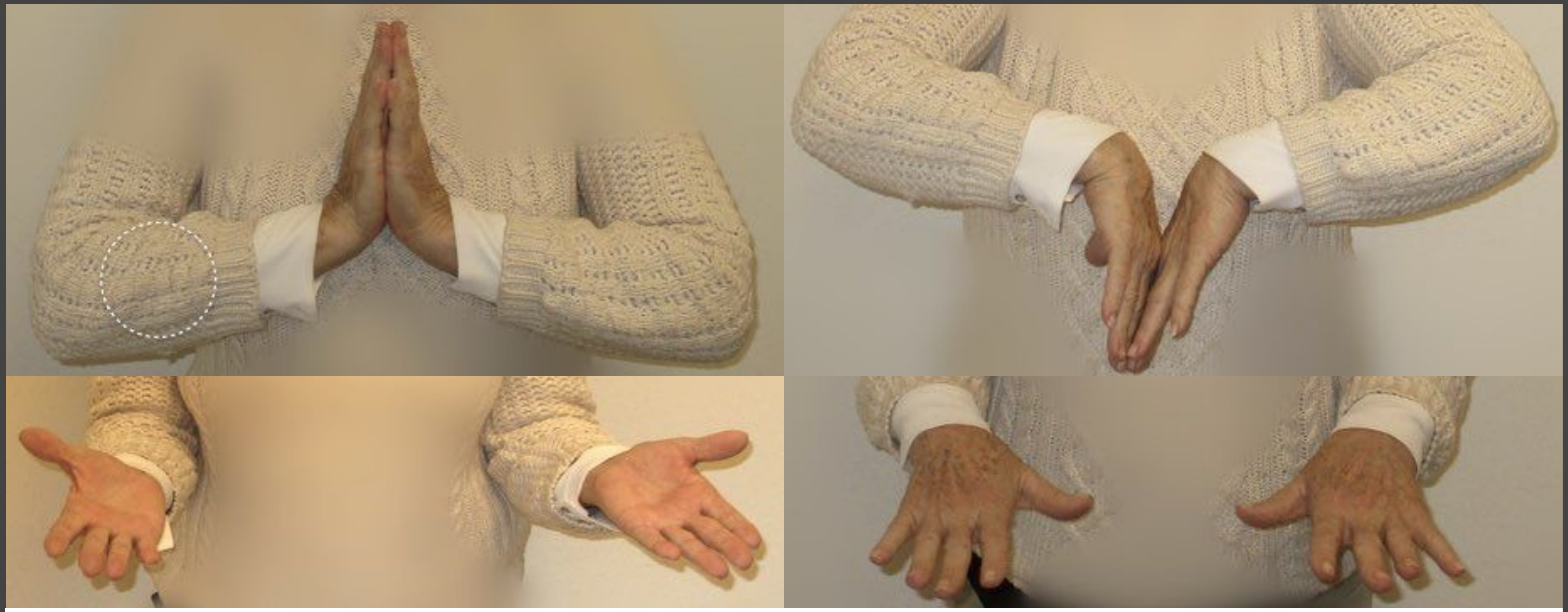

ICUC **Score** ***Functional Limitation:*** **0** (0-4) - ***Pain:*** **0** (0-4)

ICUC **Score** 33w ***Functional Limitation:*** **2** (0-4) - ***Pain:*** **2** (0-4)

Quick DASH = 22

ICUC® ID: 23-DC-1027 / 25y Male

Complete case data can be accessed by clicking the ***ICUC ID***

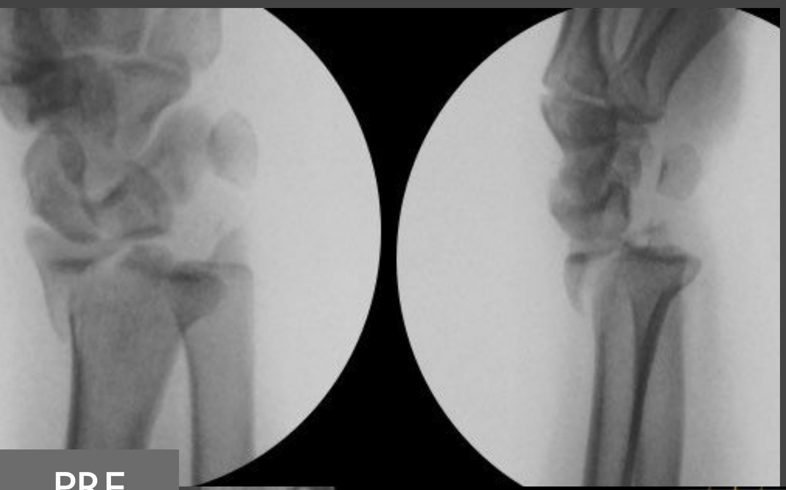

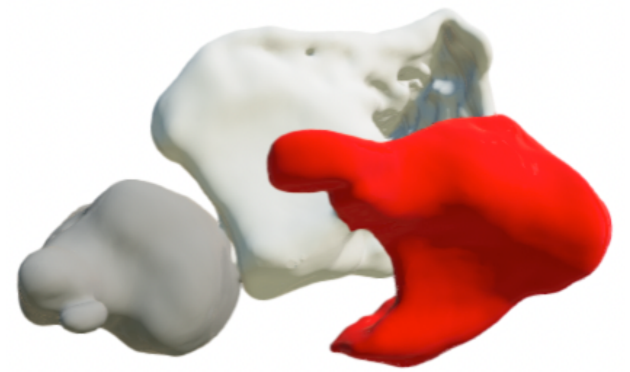

3D Colored Model

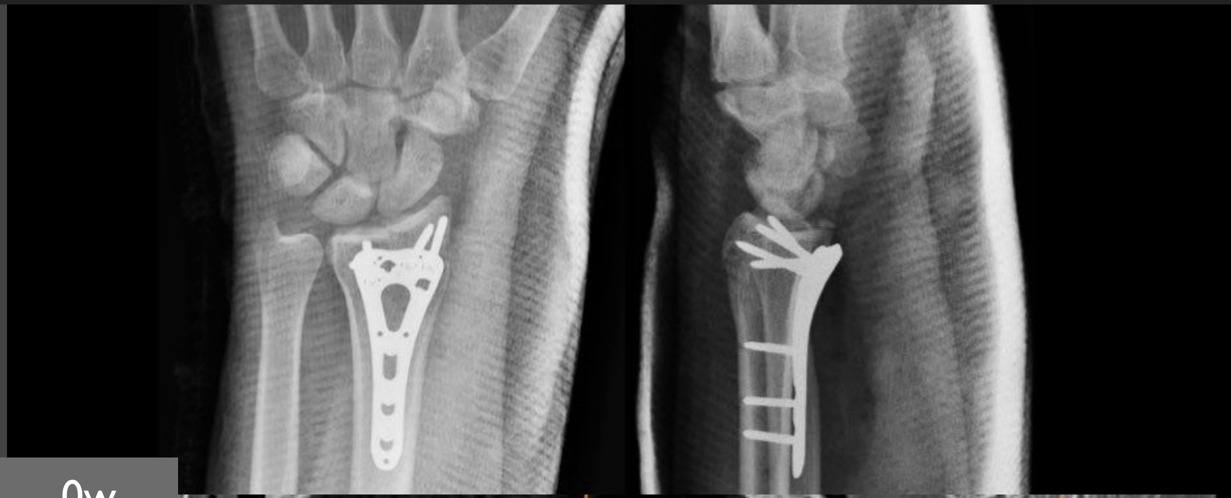

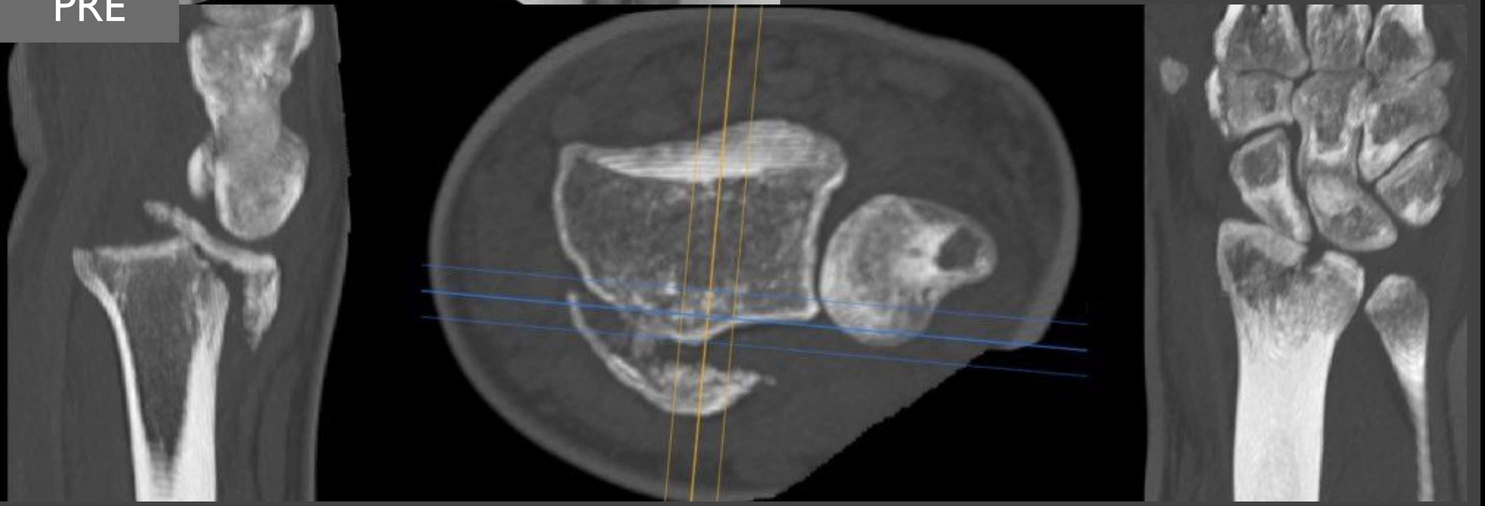

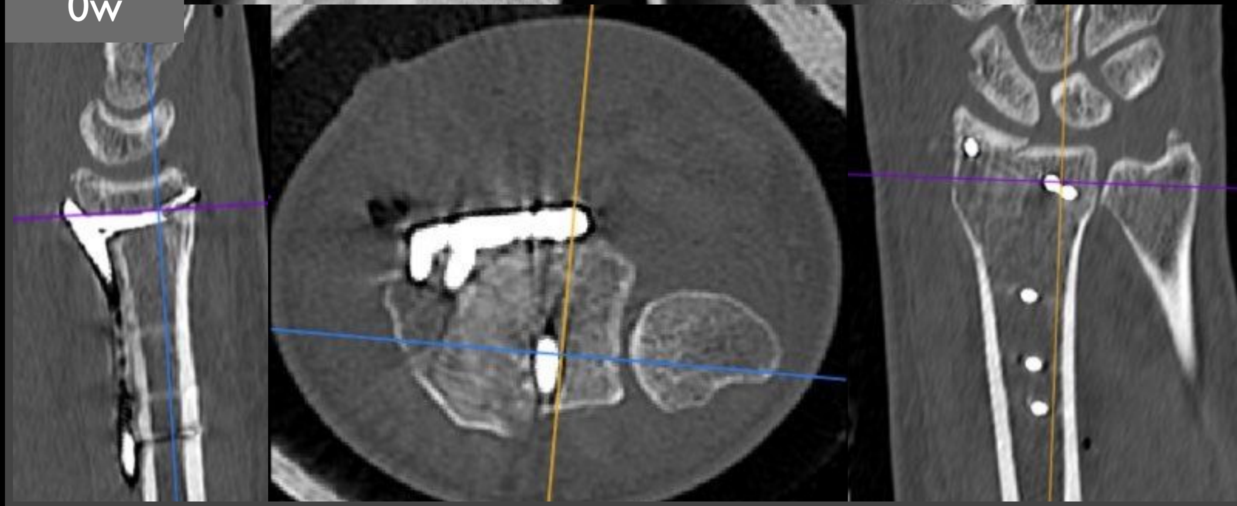

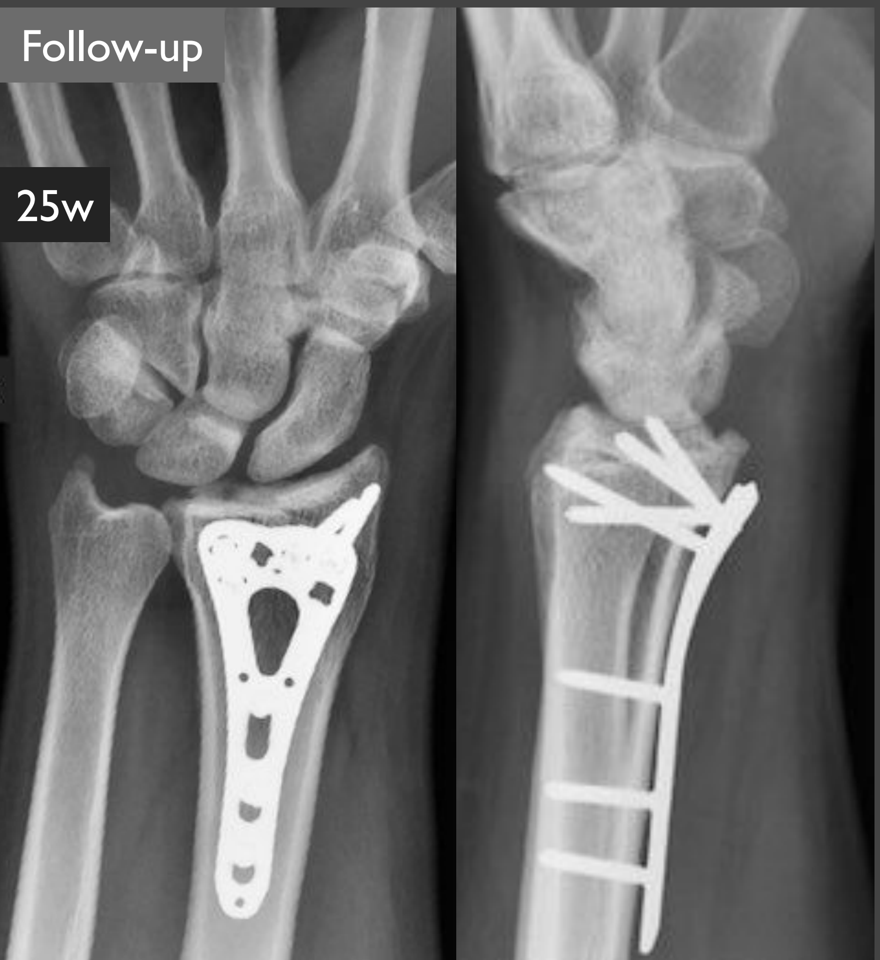

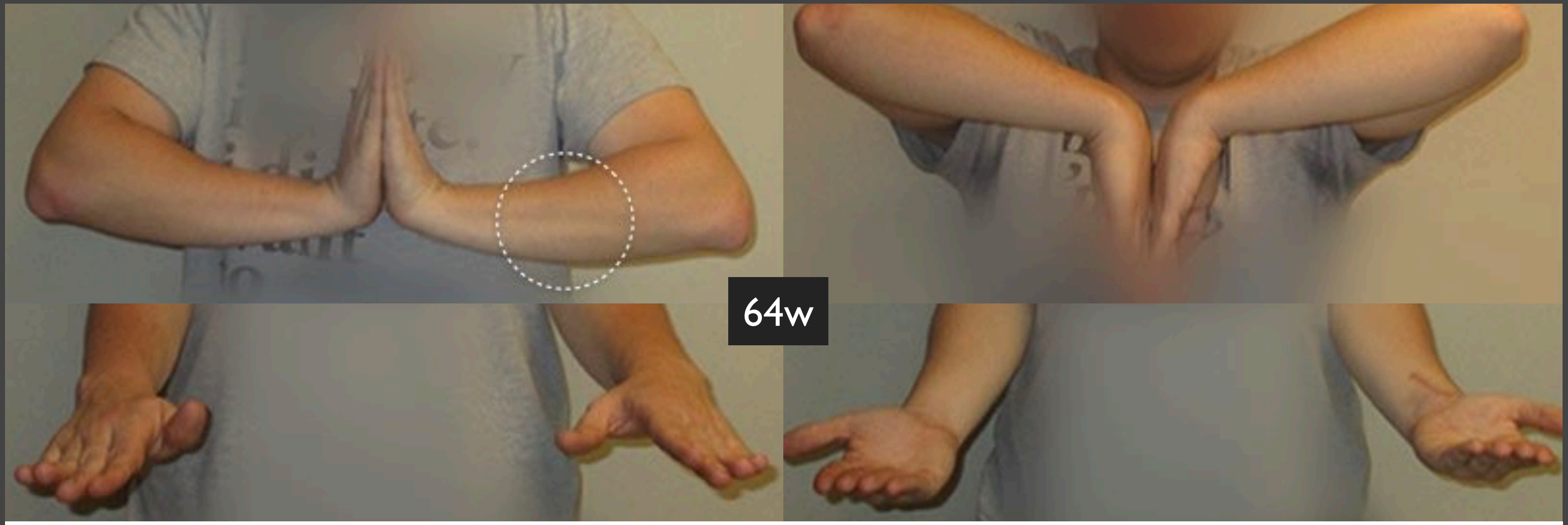

ICUC Score *Functional Limitation:* **1** (0-4) - *Pain:* **0** (0-4)

Quick DASH = 8

ICUC® ID: 23-DI-397 / 45y Male

*Complete case data can be accessed by clicking the **ICUC ID***

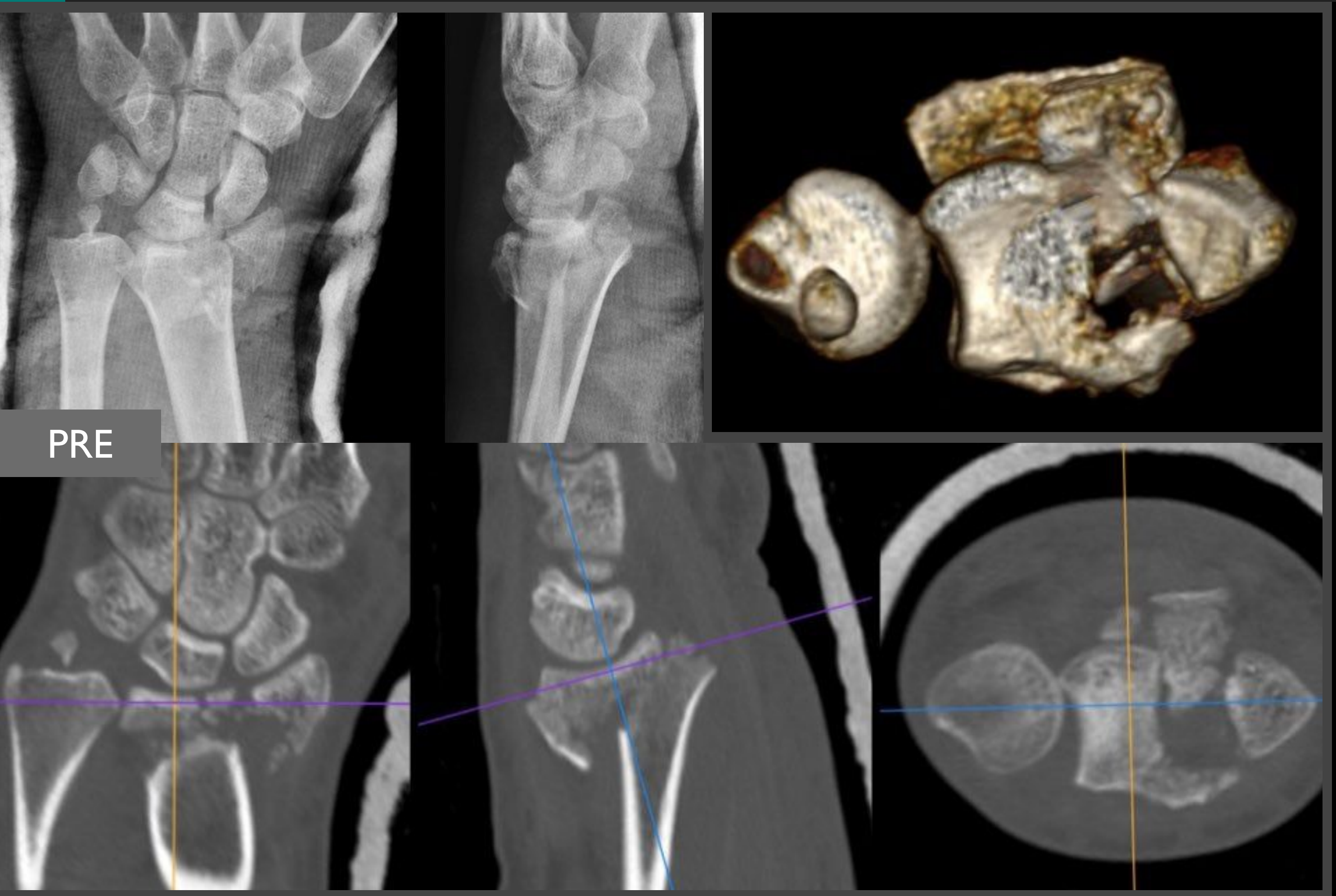

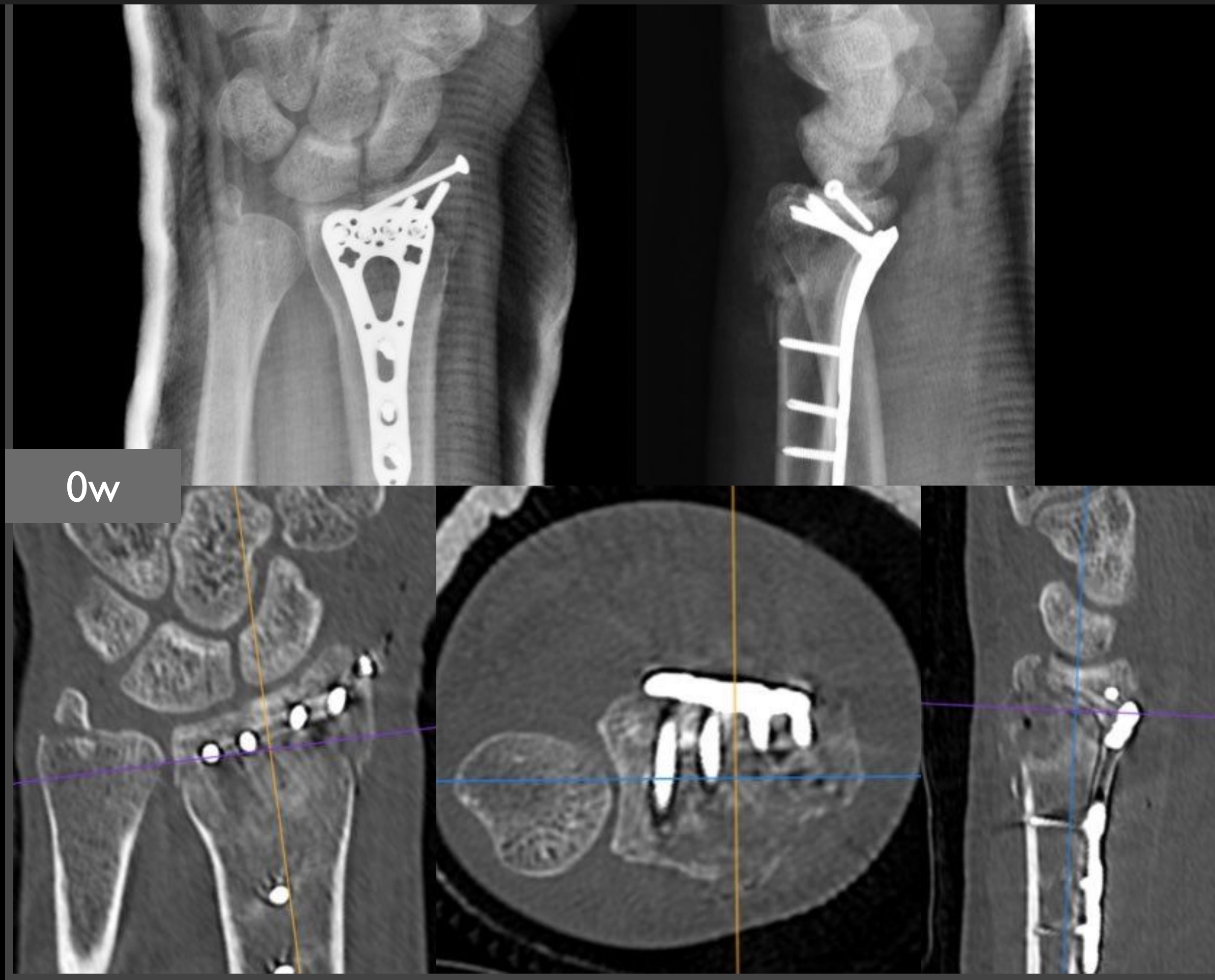

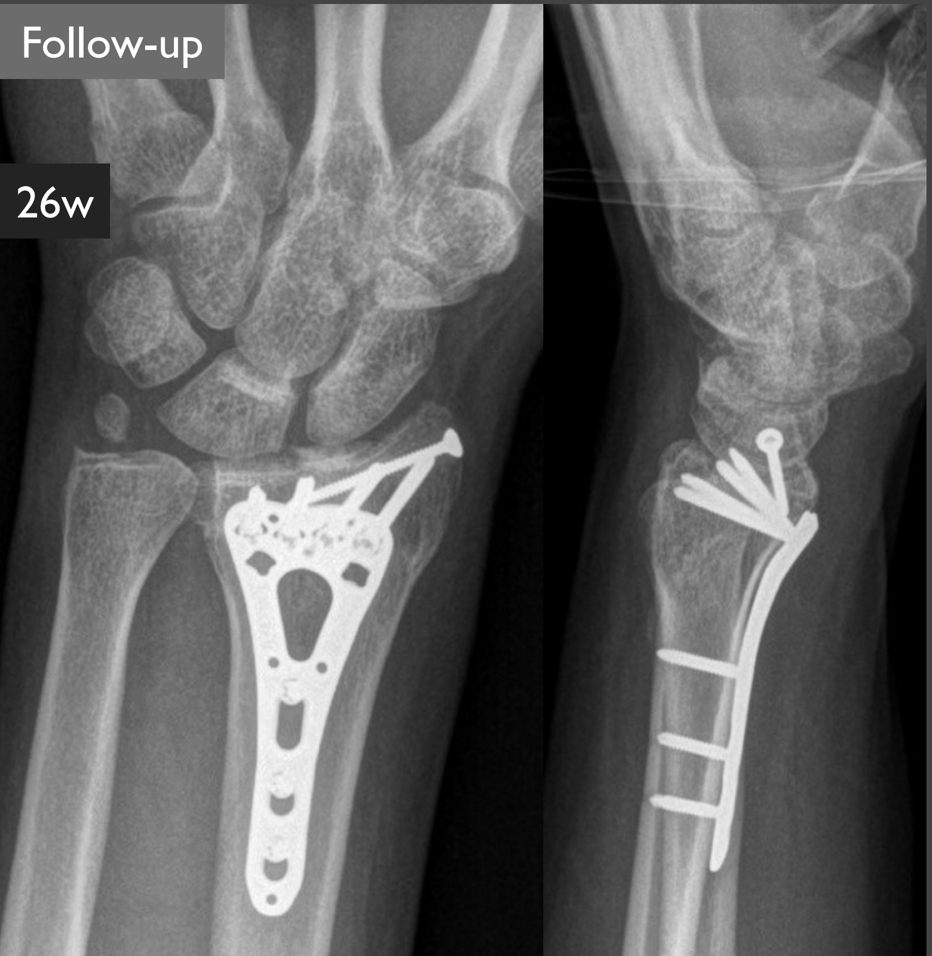

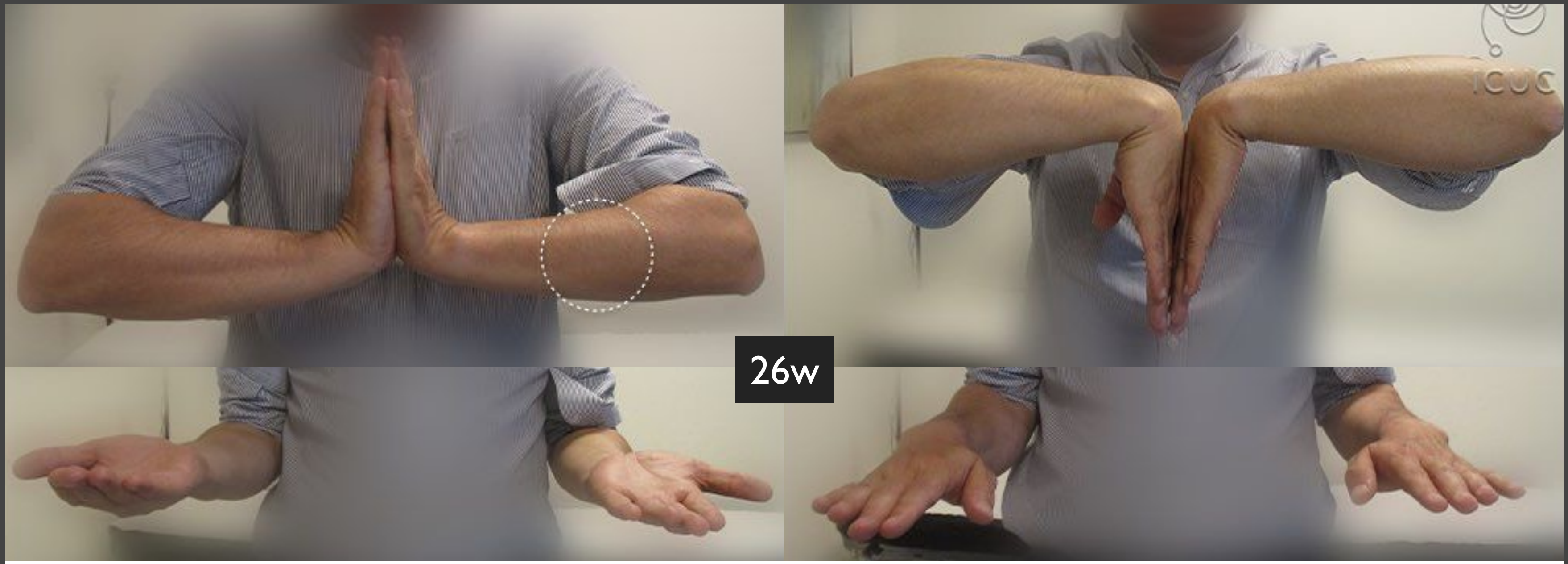

ICUC Score *Functional Limitation:* **1** (0-4) - *Pain:* **0** (0-4)

Quick DASH = 0

ICUC® ID: 23-VE-887 / 45y Female

Complete case data can be accessed by clicking the ***ICUC ID***

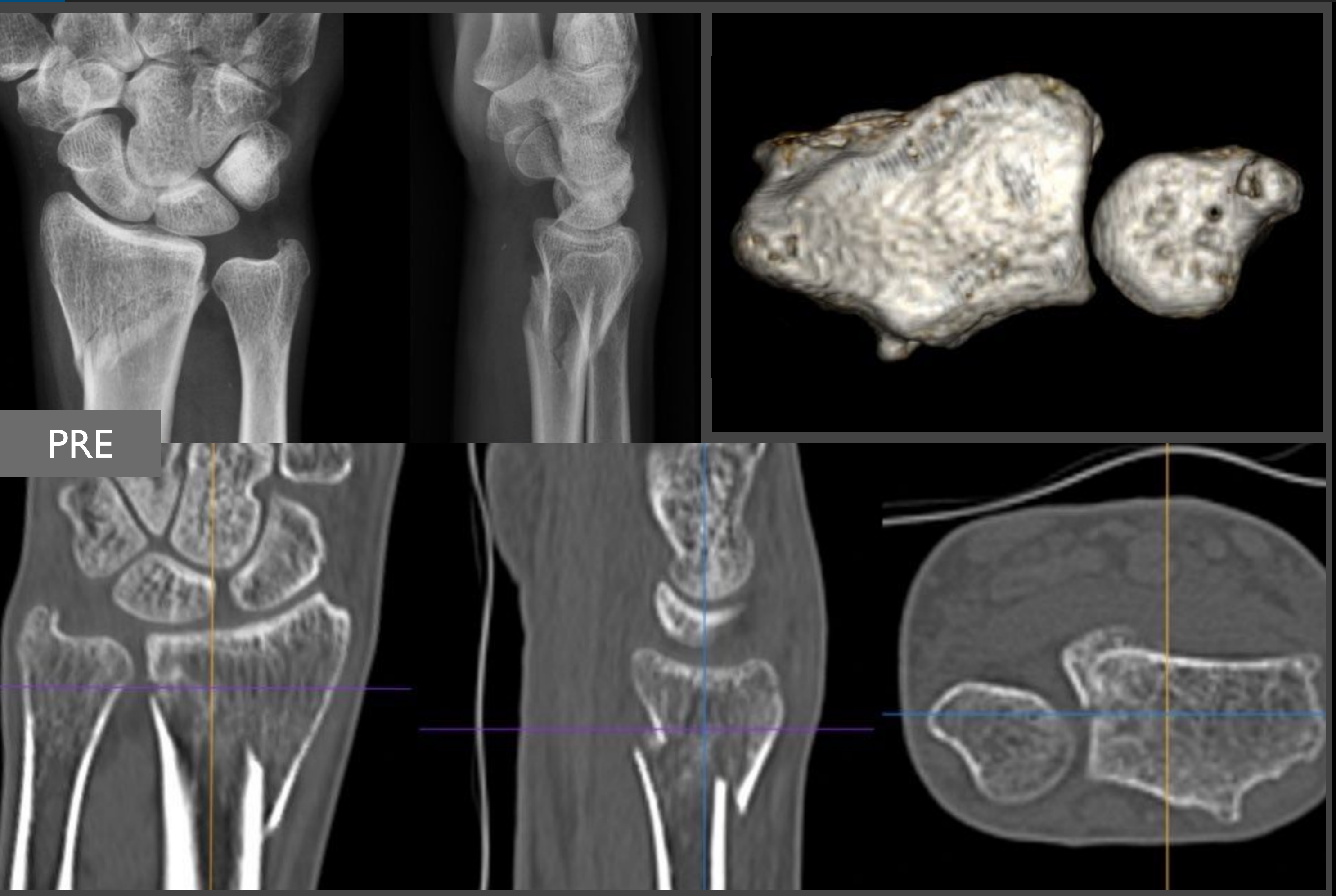

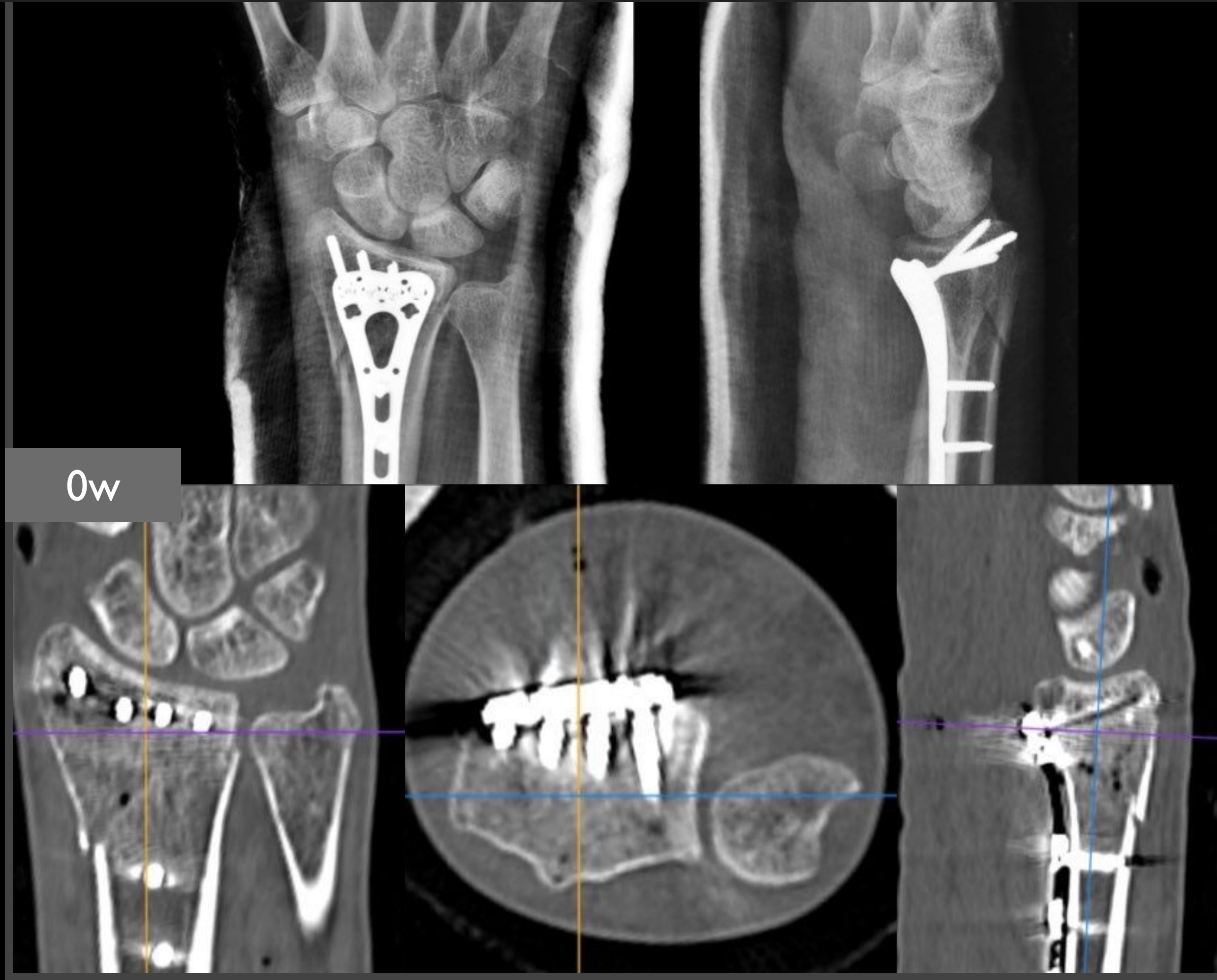

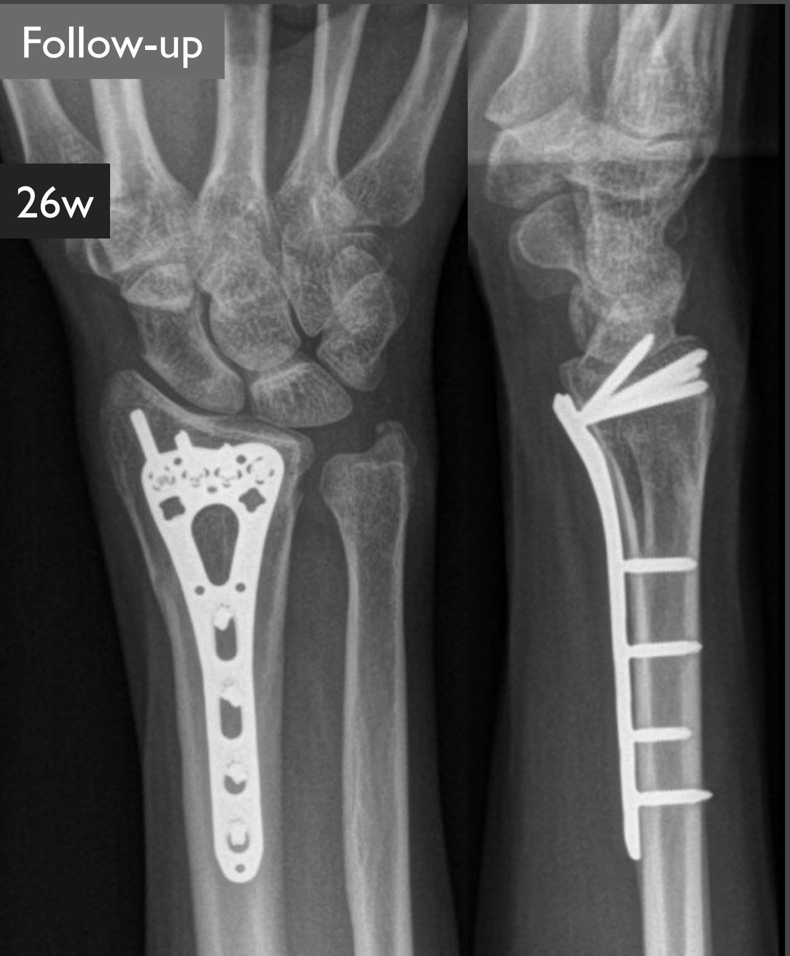

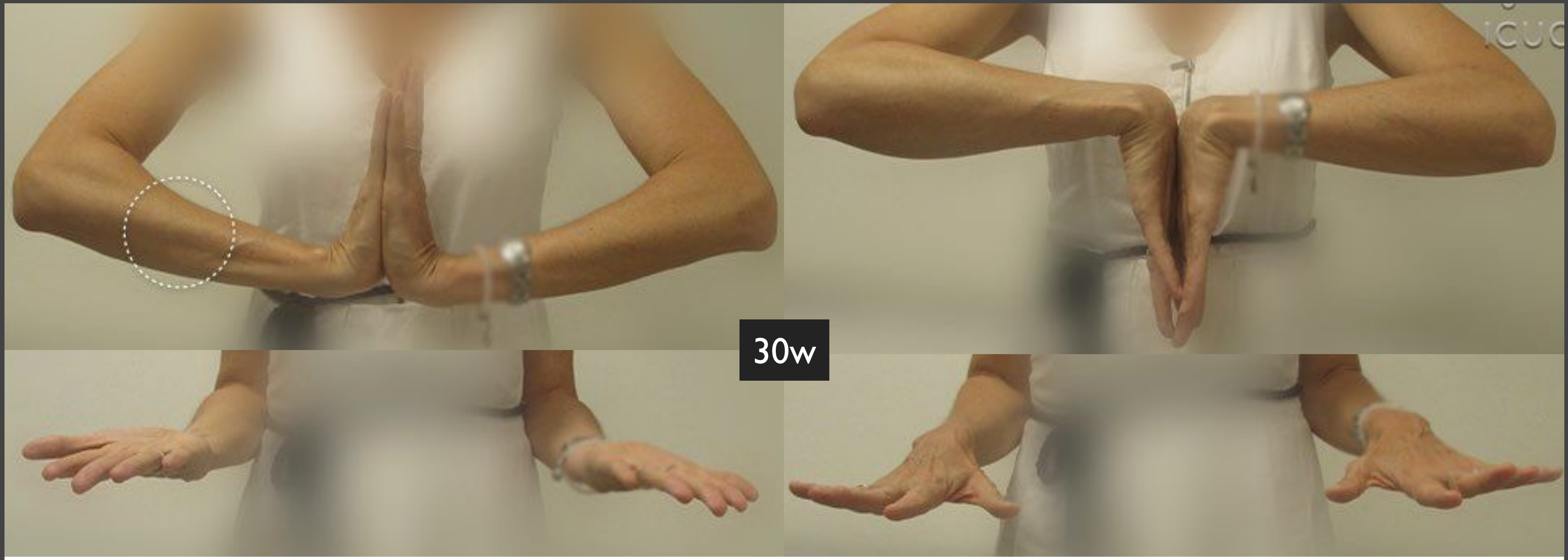

ICUC **Score** *Functional Limitation:* **1** (0-4) - *Pain:* **1** (0-4)

Quick DASH = 6

ICUC® ID: 23-DS-984 / 70y Female

Complete case data can be accessed by clicking the ***ICUC ID***

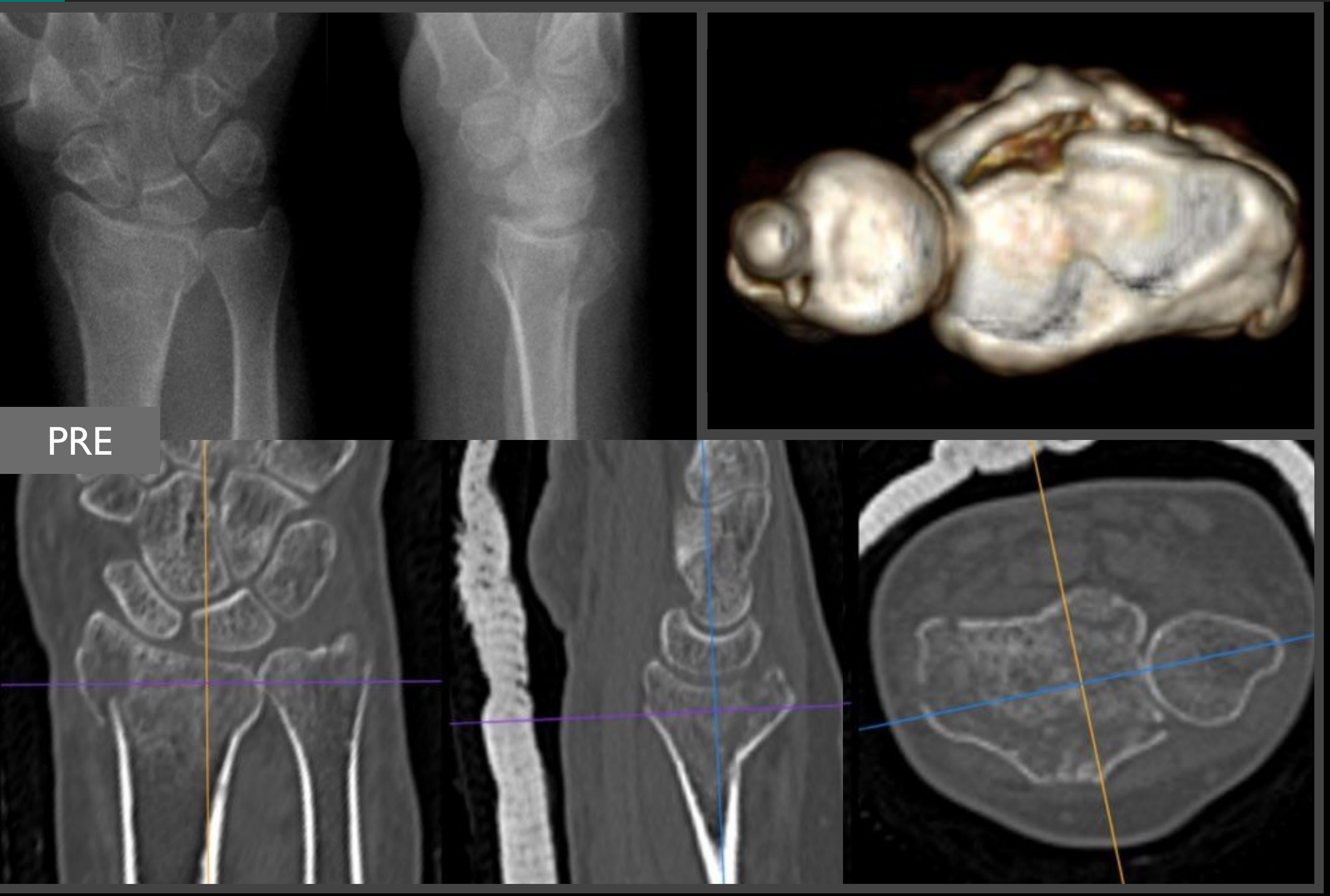

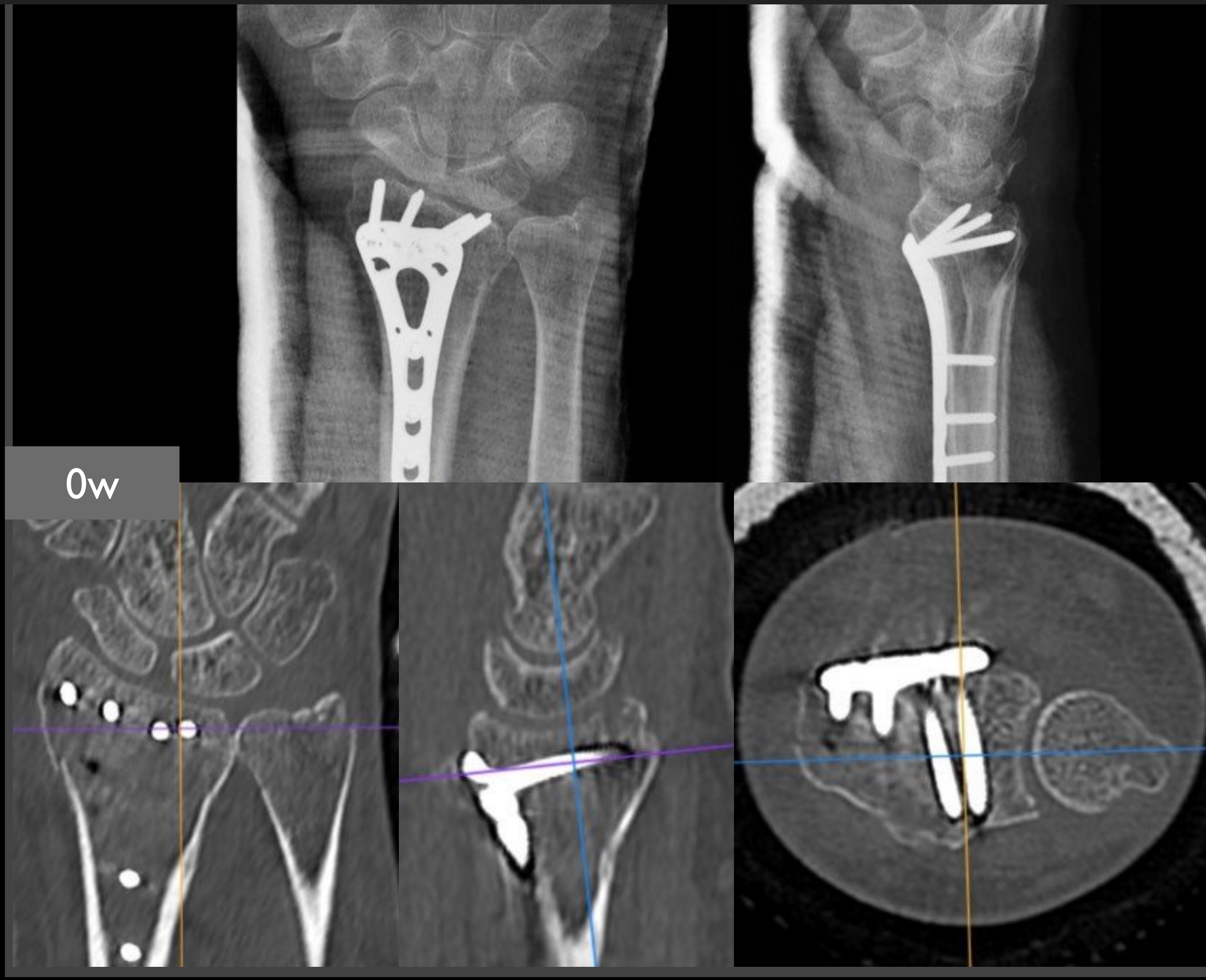

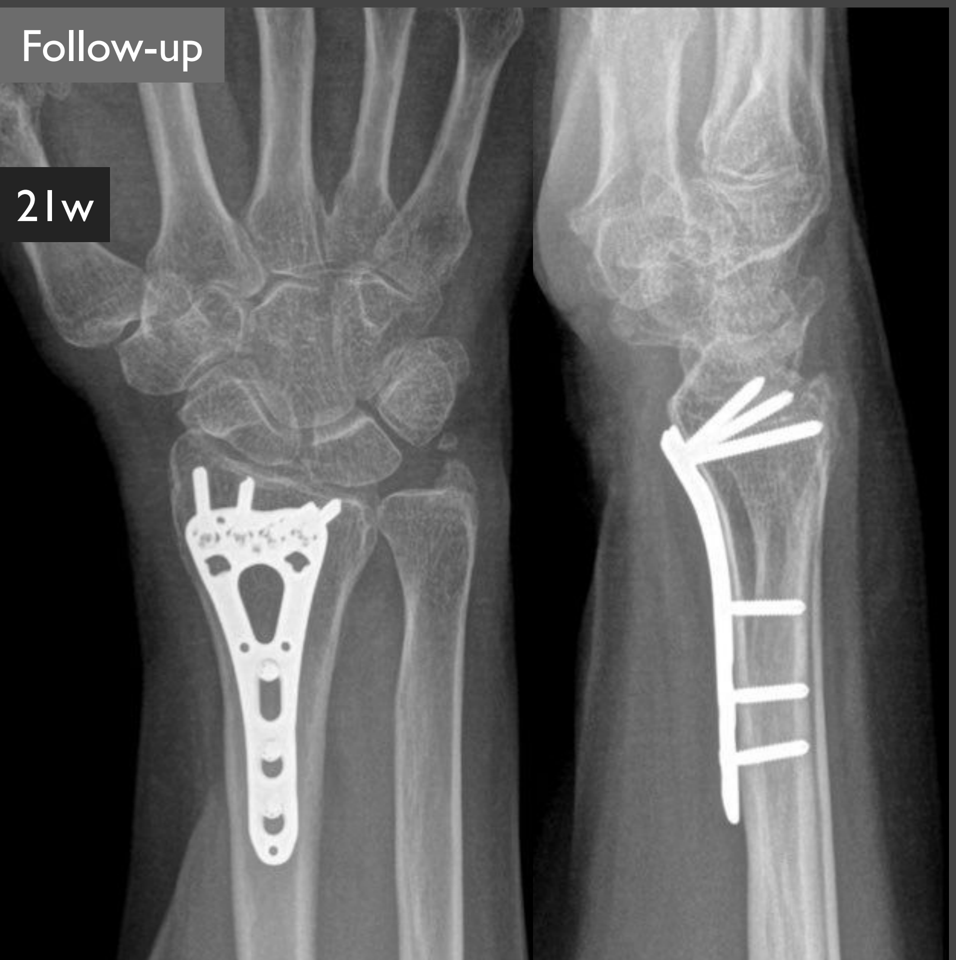

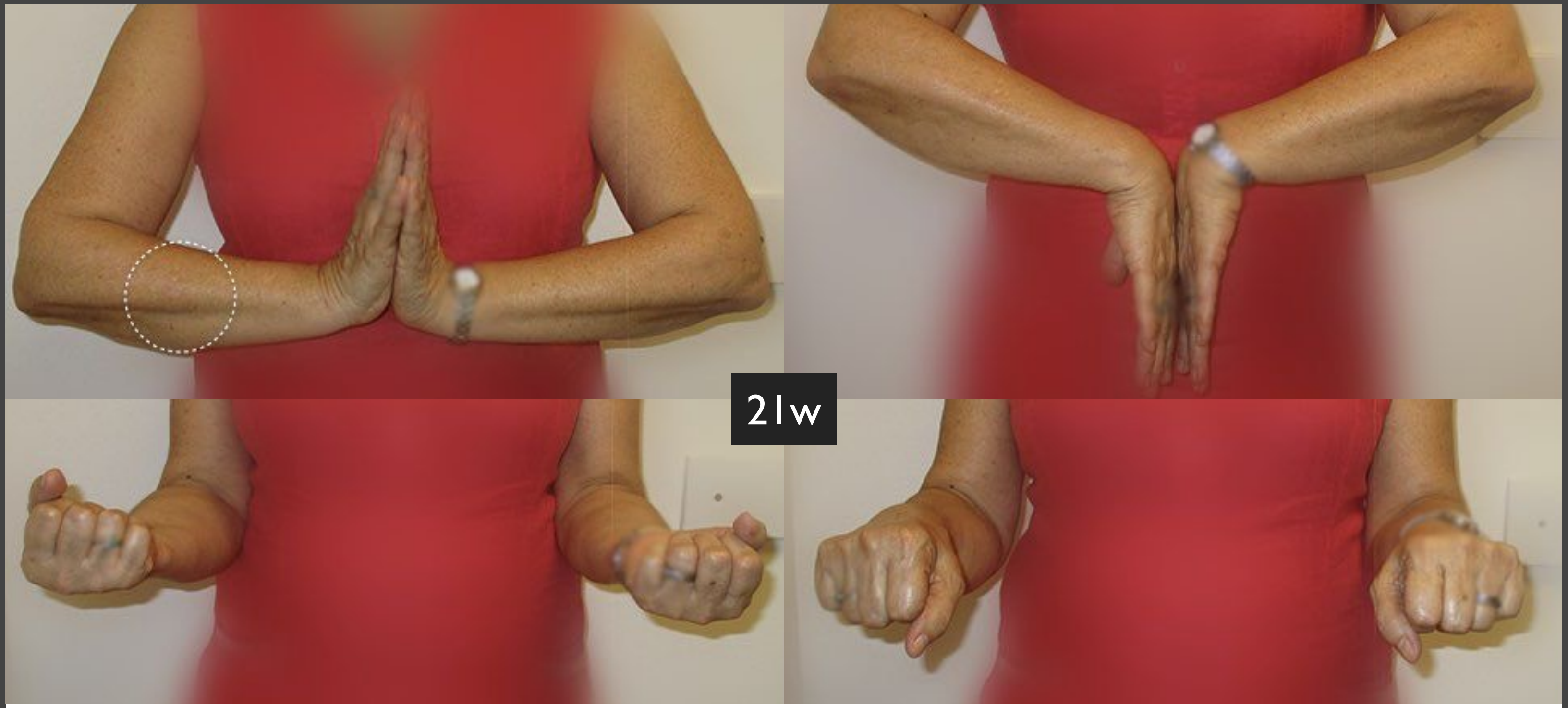

ICUC **Score** *Functional Limitation:* **1** (0-4) - *Pain:* **0** (0-4)

Quick DASH = 6

ICUC® ID: 23-VB-864 / 55y Female

Complete case data can be accessed by clicking the ***ICUC ID***

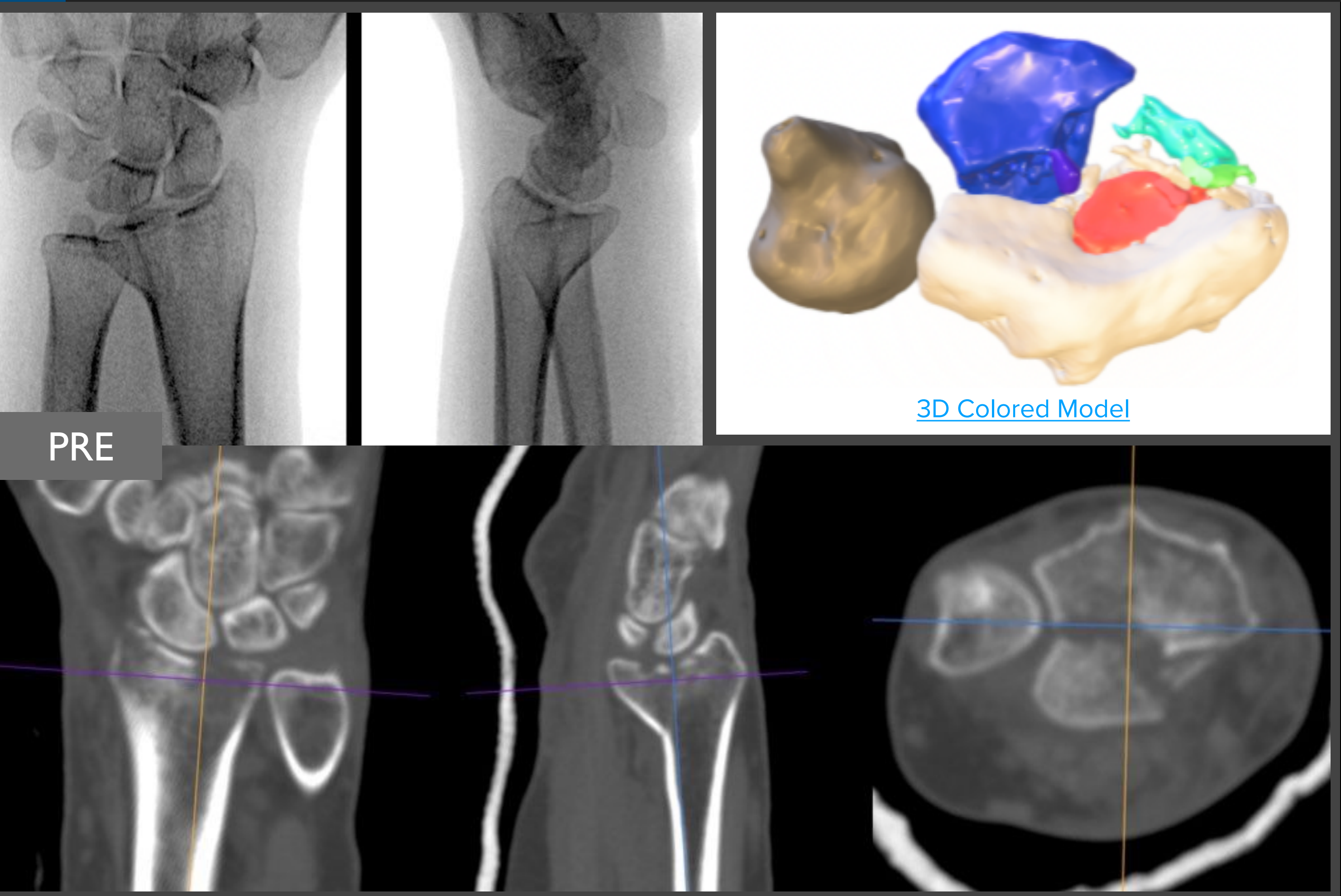

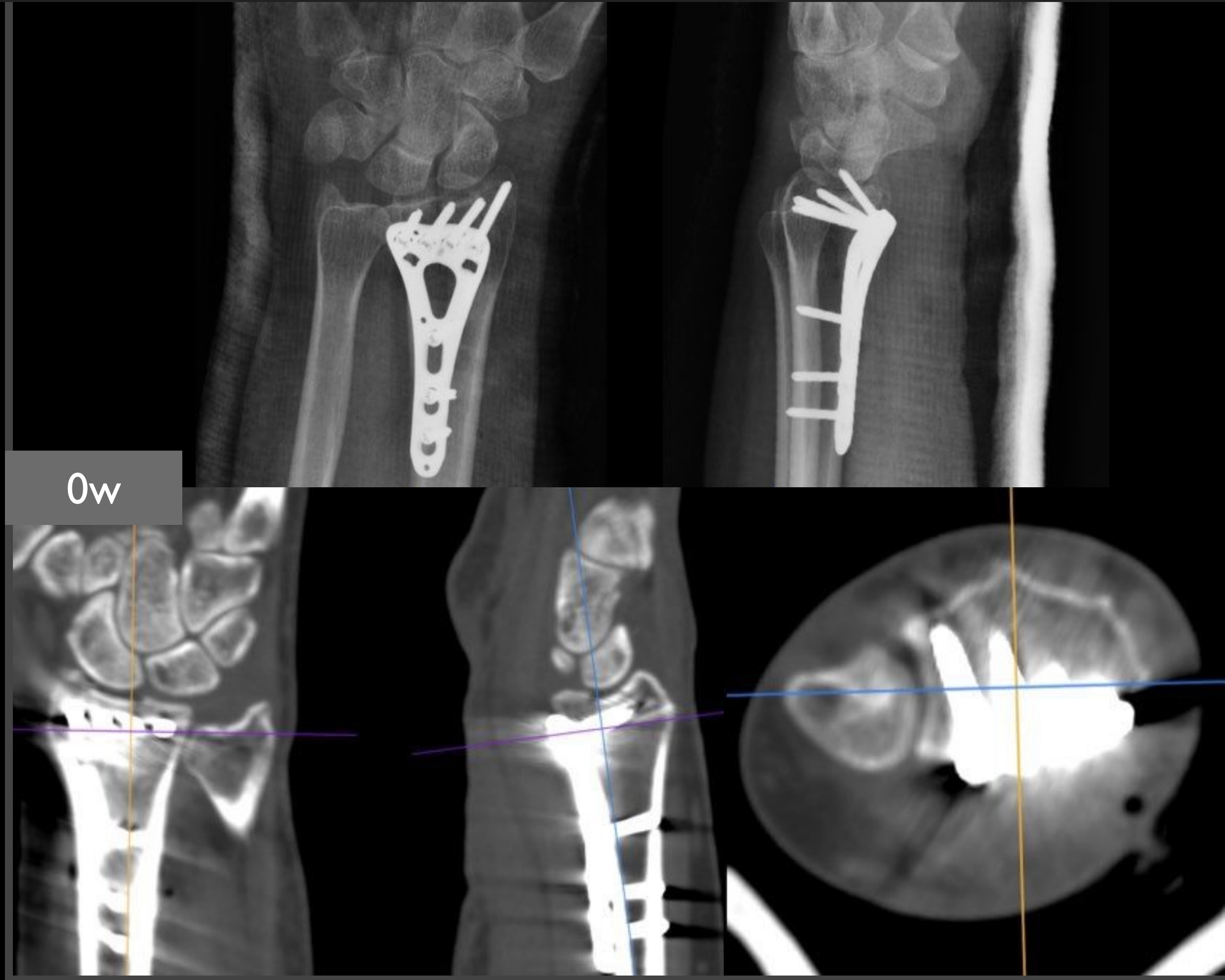

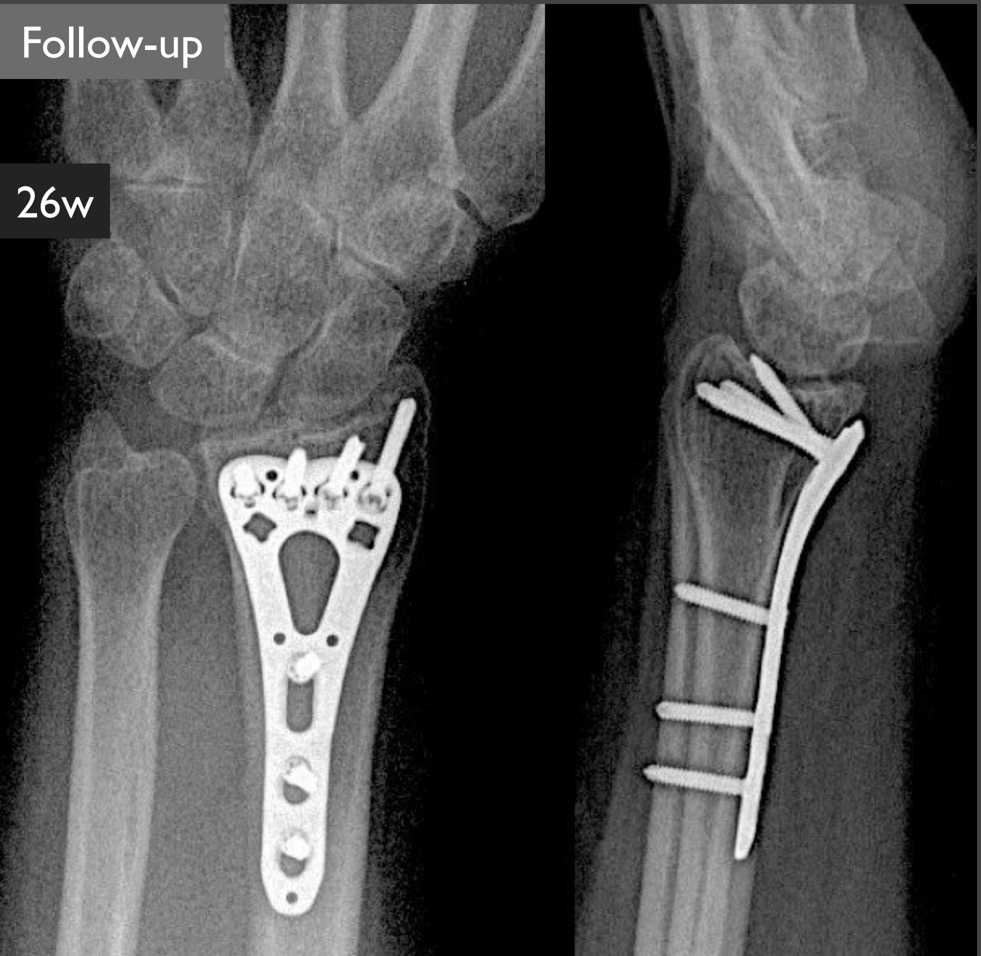

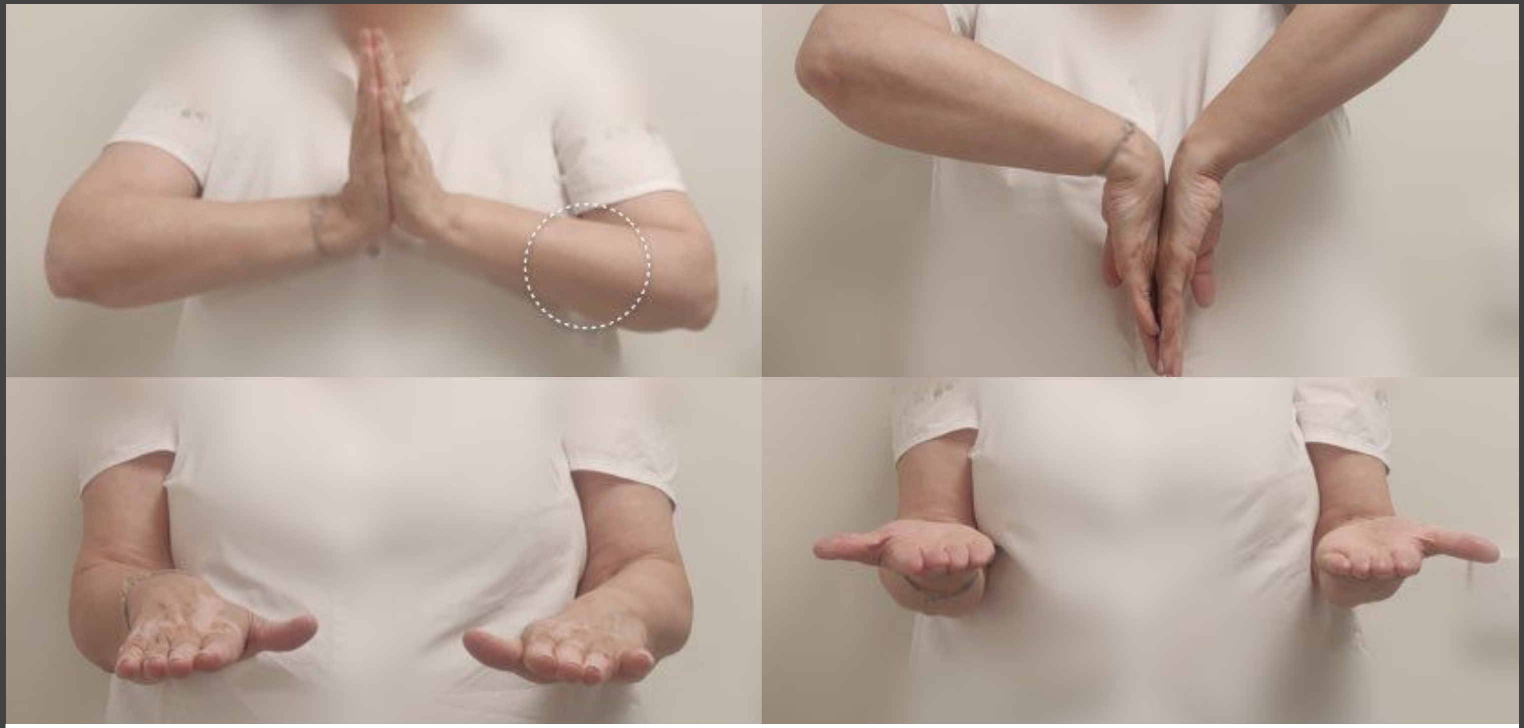

ICUC Score Functional Limitation: 2 (0-4) - Pain: 1 (0-4)

Quick DASH = 17

ICUC® ID: 23-DS-530 / 45y Male

Complete case data can be accessed by clicking the ***ICUC ID***

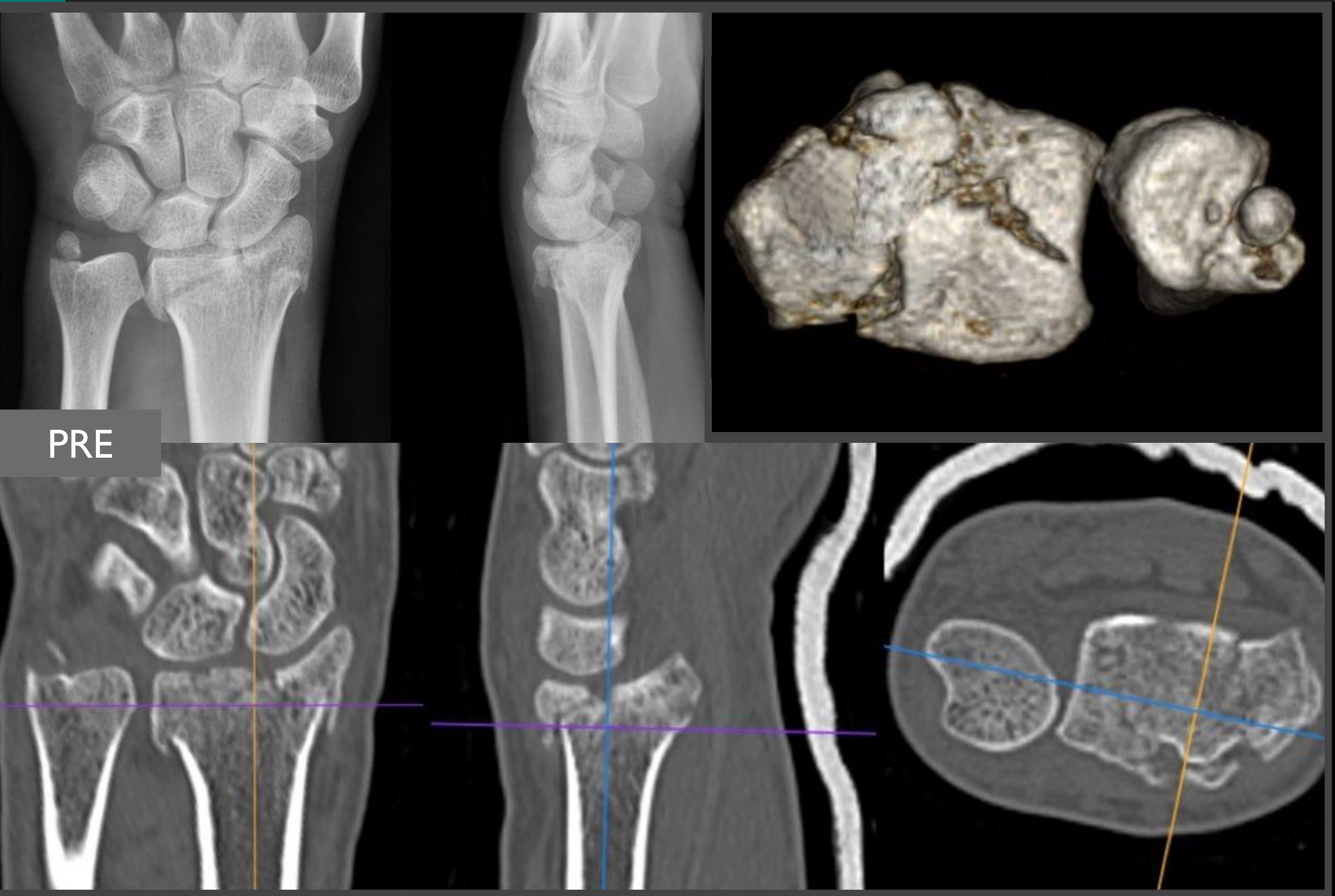

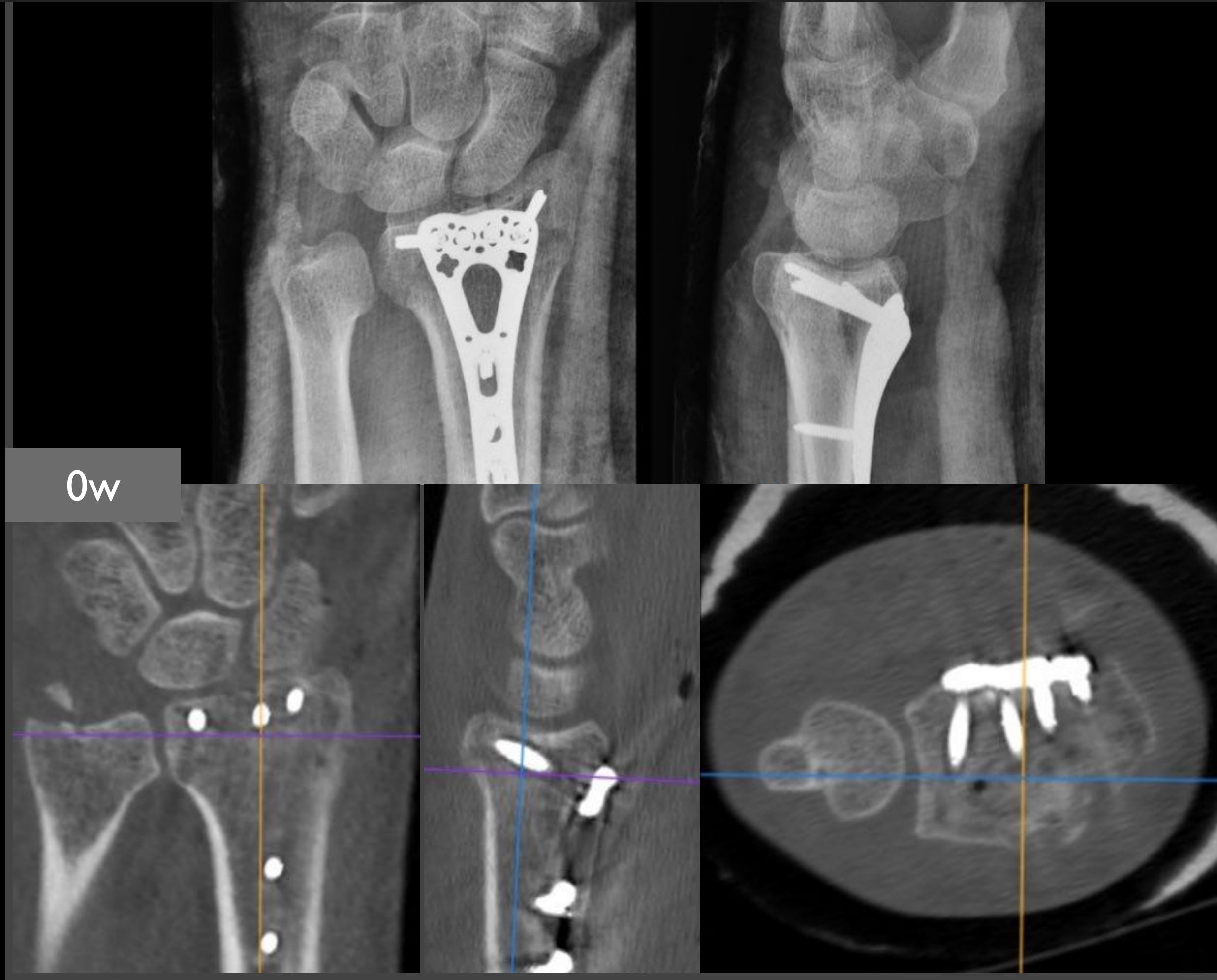

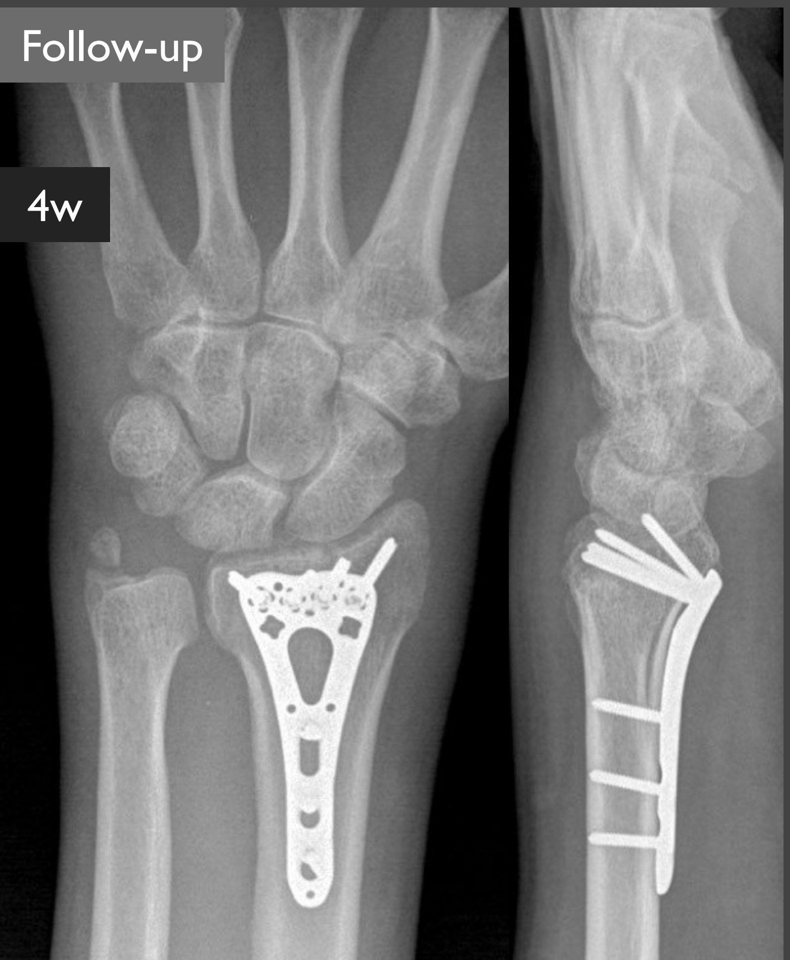

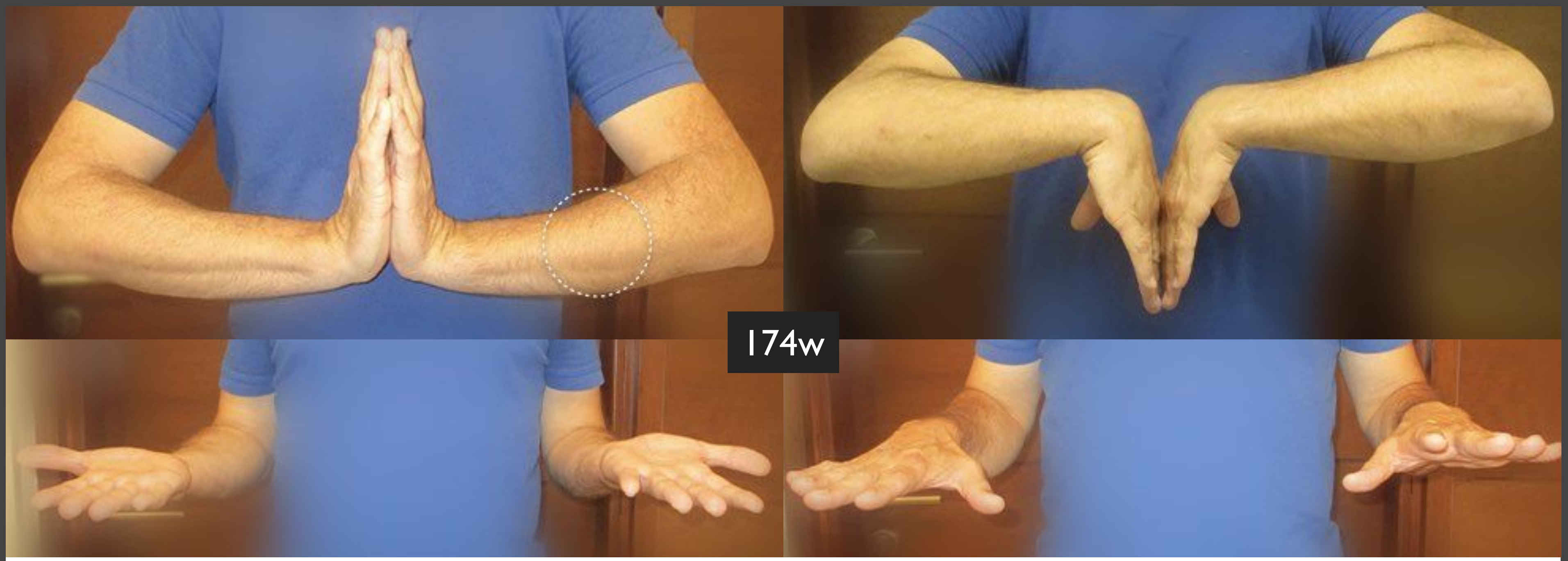

ICUC Score   *Functional Limitation:* **1** (0-4) - *Pain:* **1** (0-4)

Quick DASH = 5

ICUC® ID: 23-VC-121 / 70y Female

Complete case data can be accessed by clicking the ***ICUC ID***

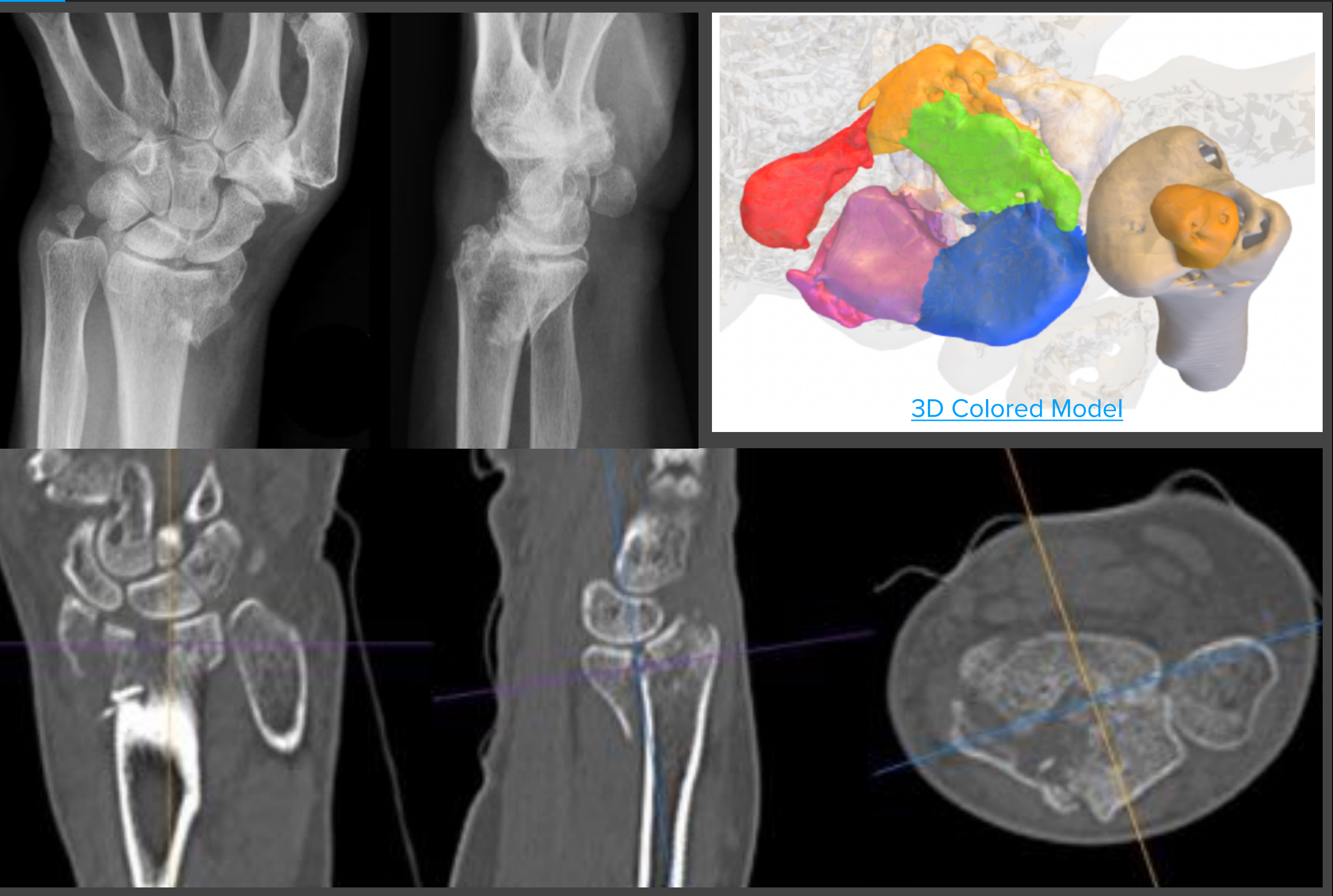

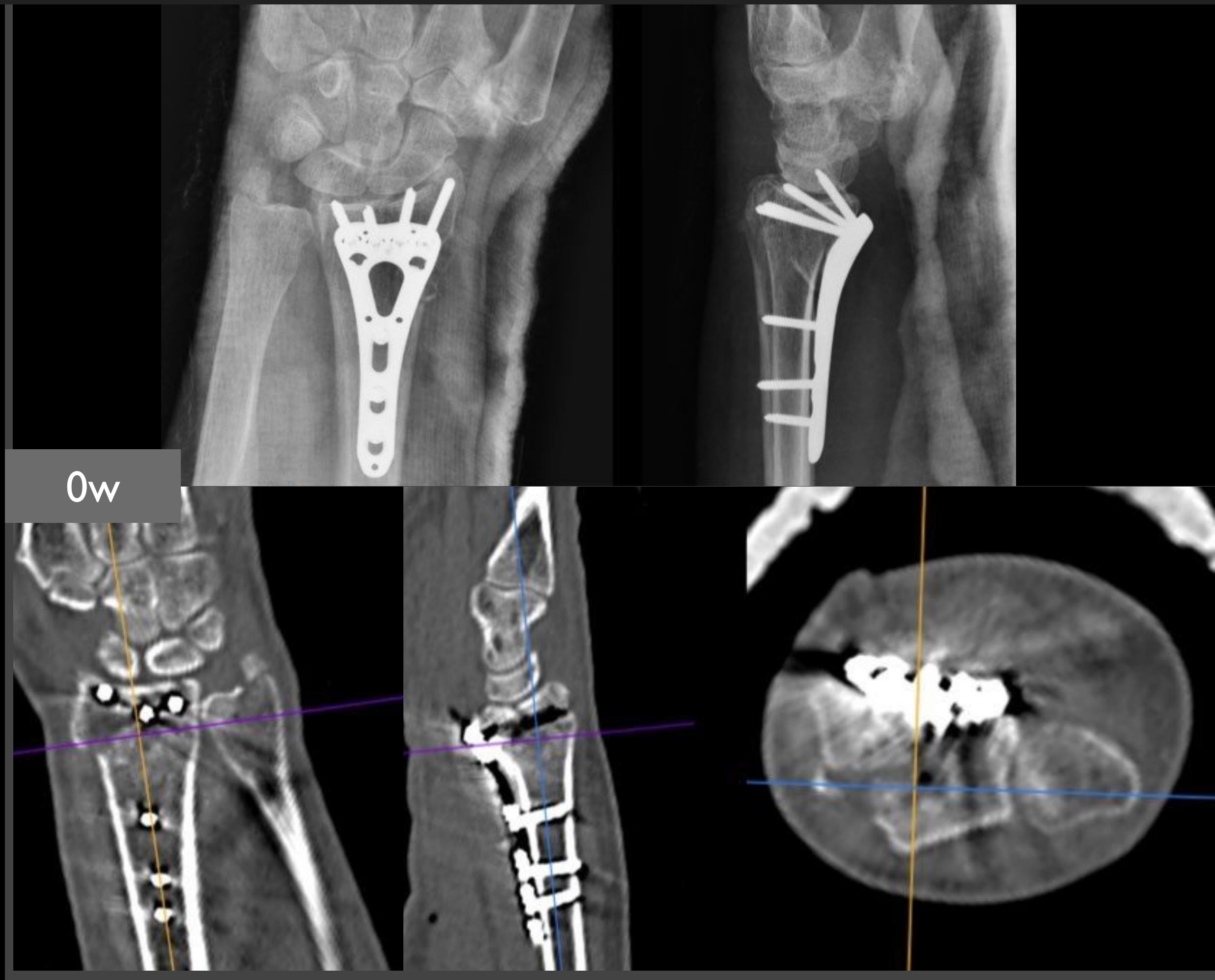

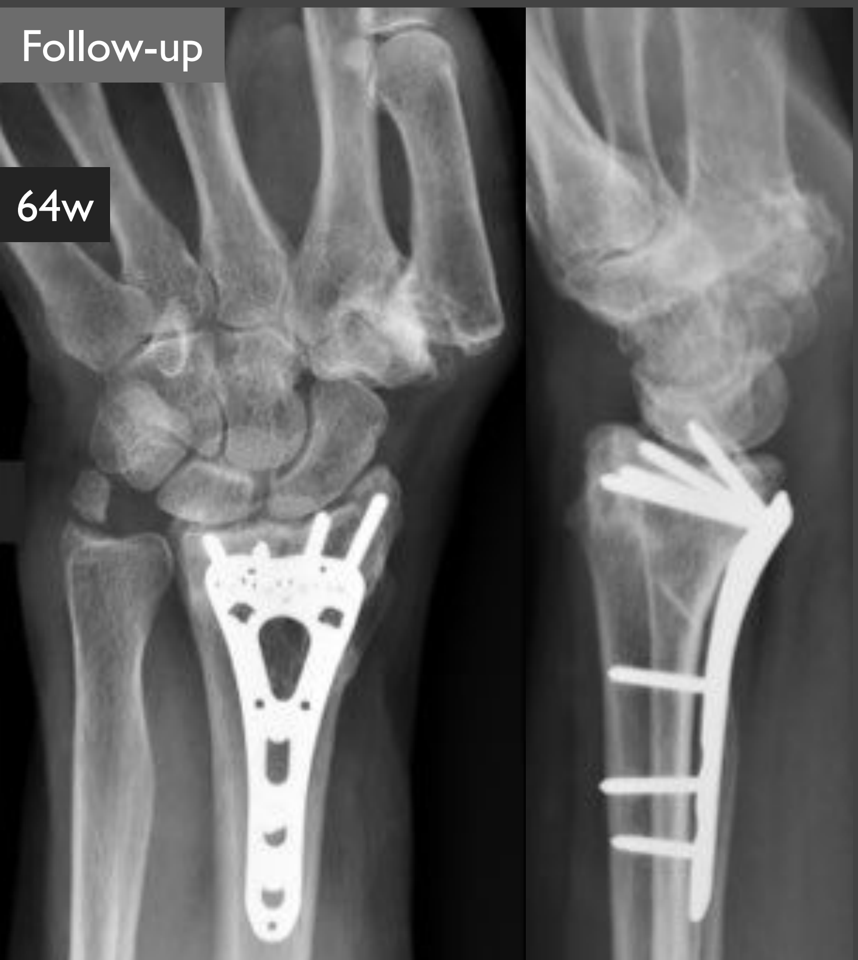

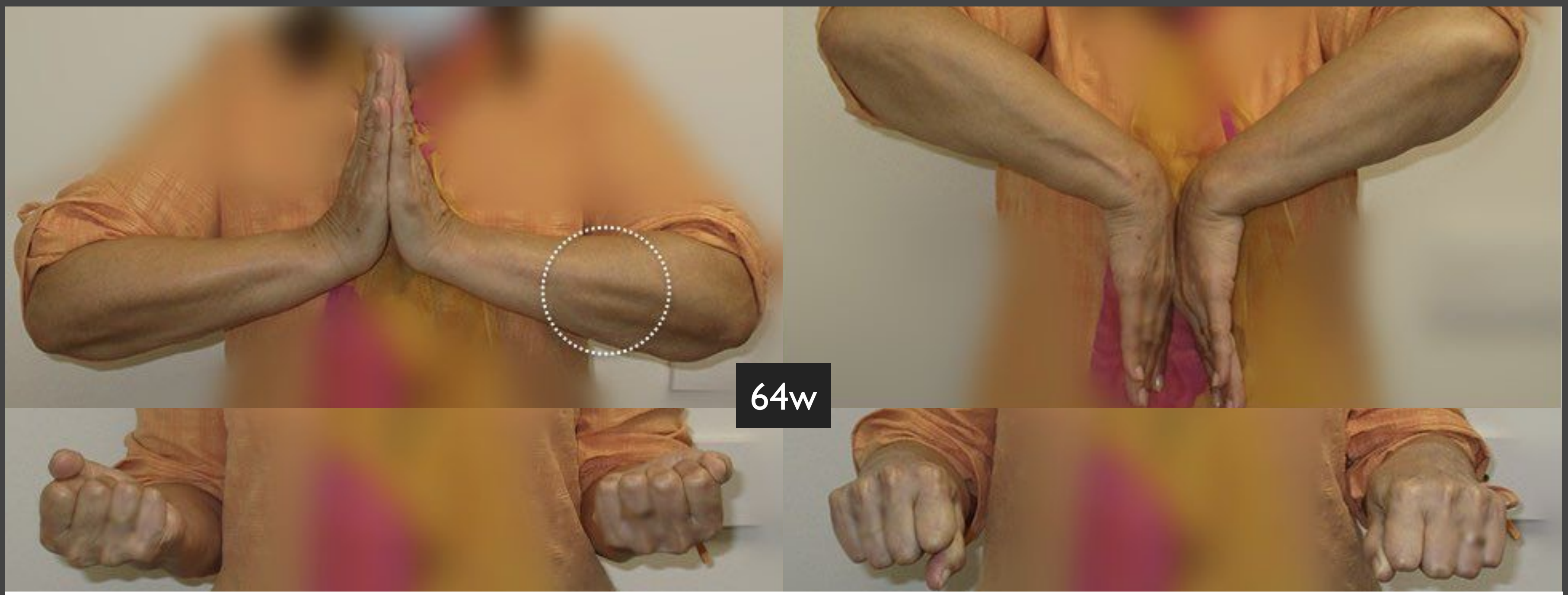

ICUC Score **Functional Limitation: 1** (0-4) - **Pain: 1** (0-4)

Quick DASH = 5

ICUC® ID: 23-VC-706 / 70y Female

Complete case data can be accessed by clicking the ***ICUC ID***

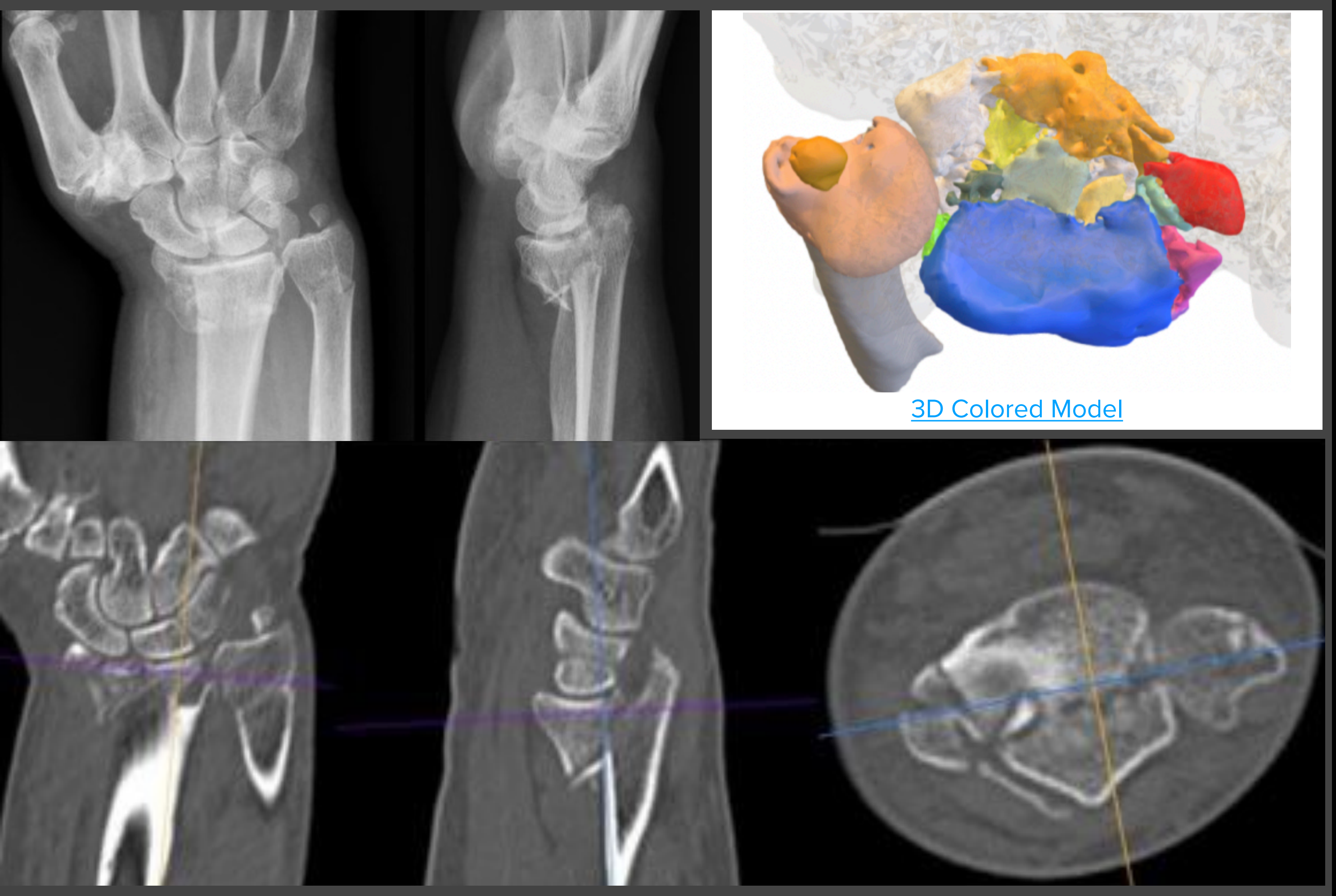

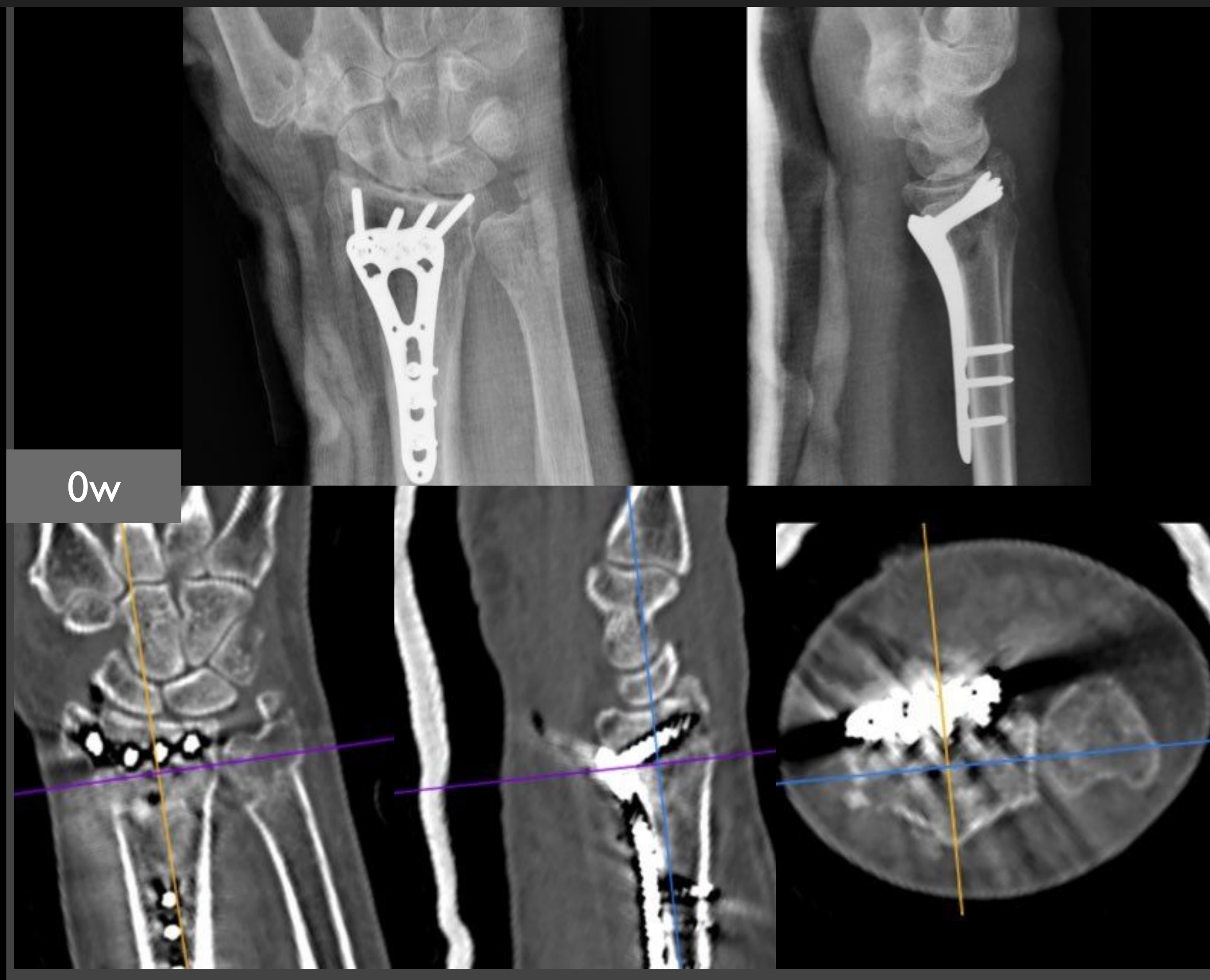

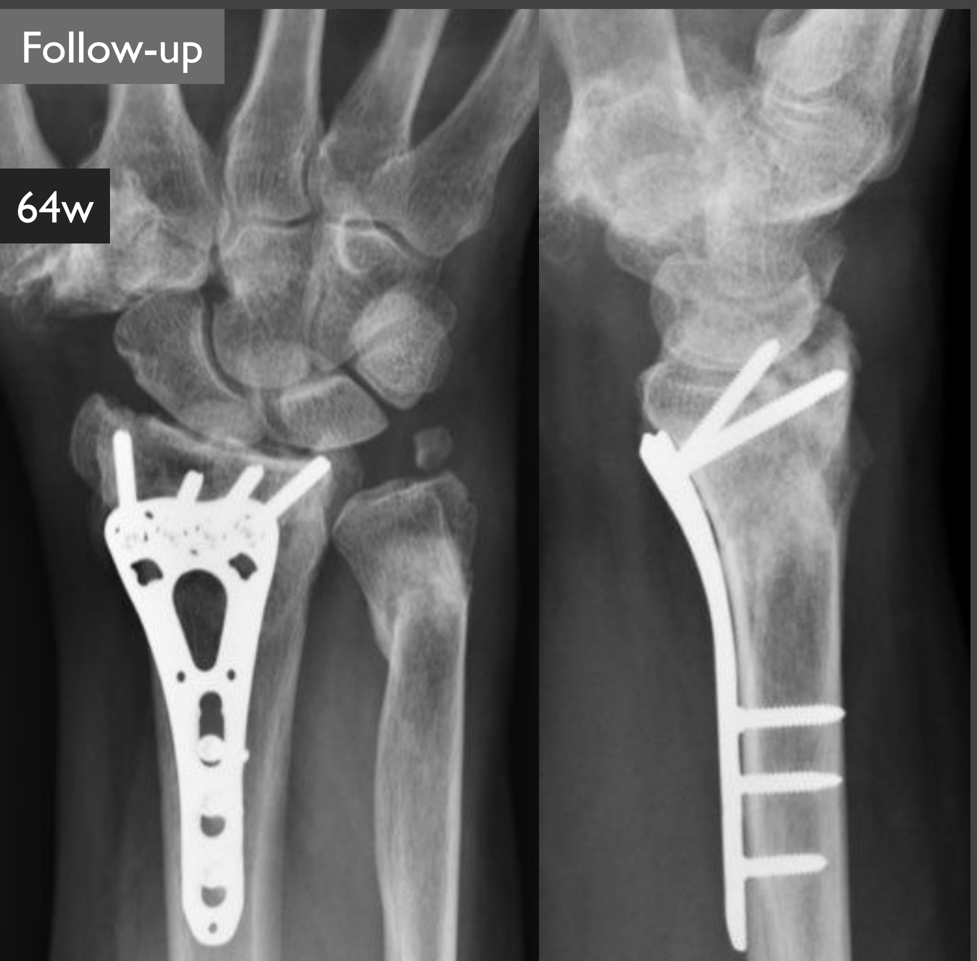

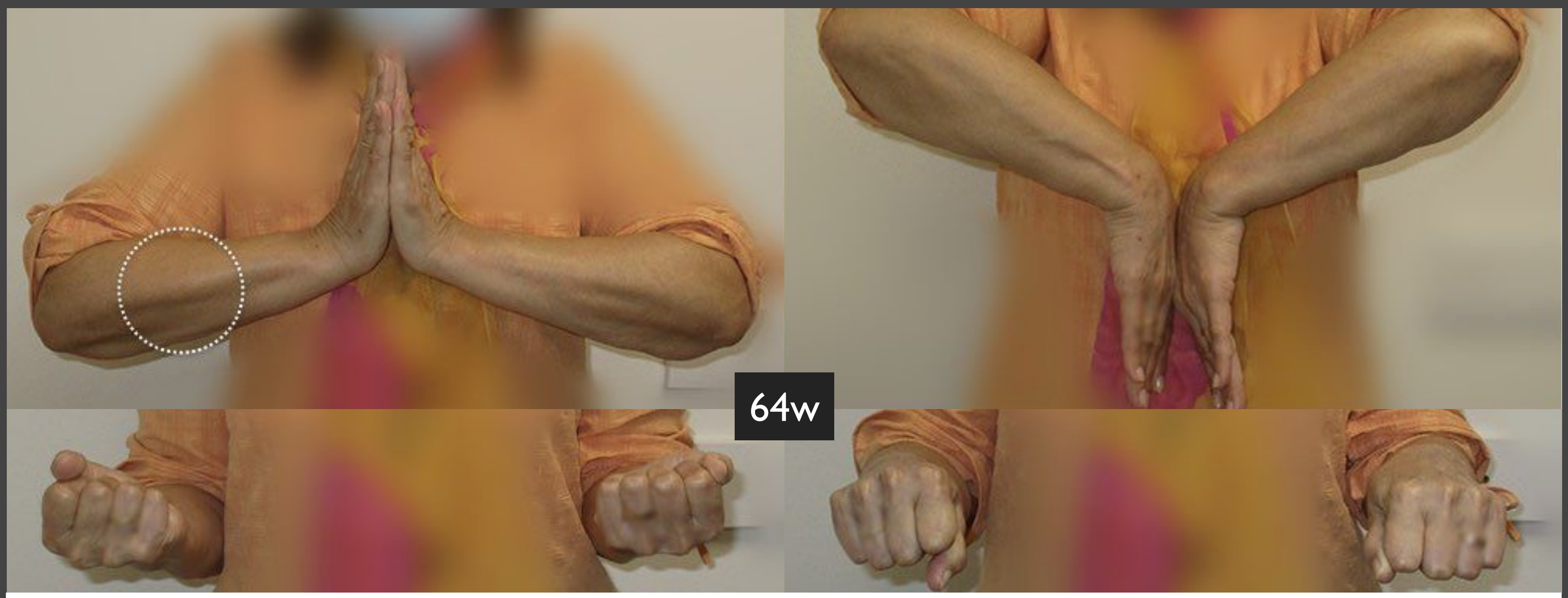

ICUC Score ***Functional Limitation:*** **1** (0-4) - ***Pain:*** **1** (0-4)

Quick DASH = 5

ICUC® ID: 23-VC-474 / 55y Female

*Complete case data can be accessed by clicking the **ICUC ID***

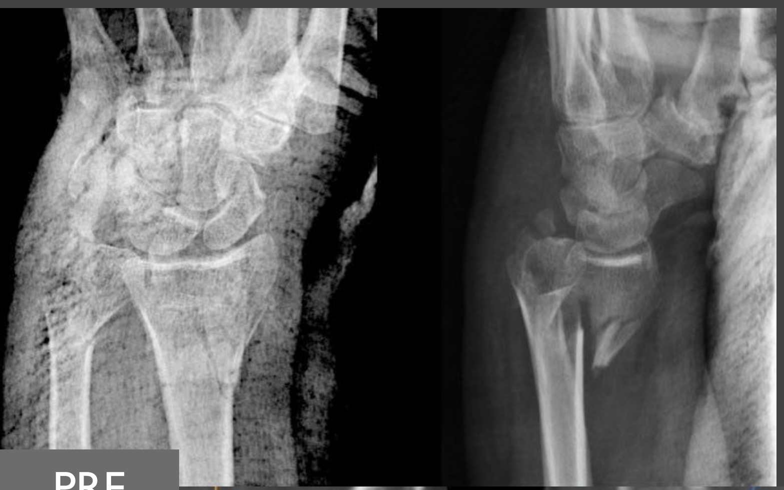

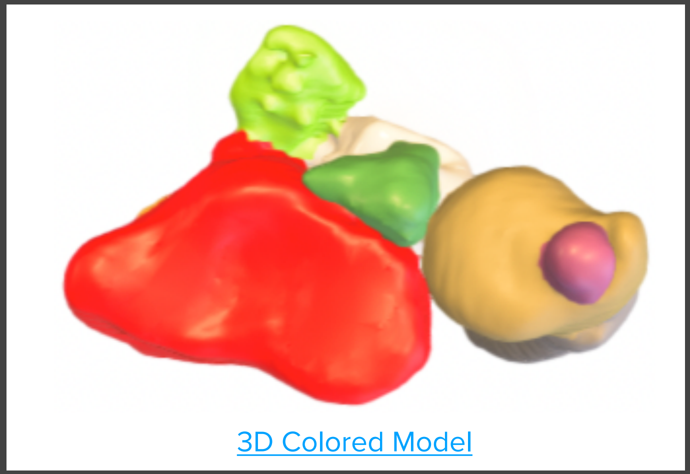

PRE

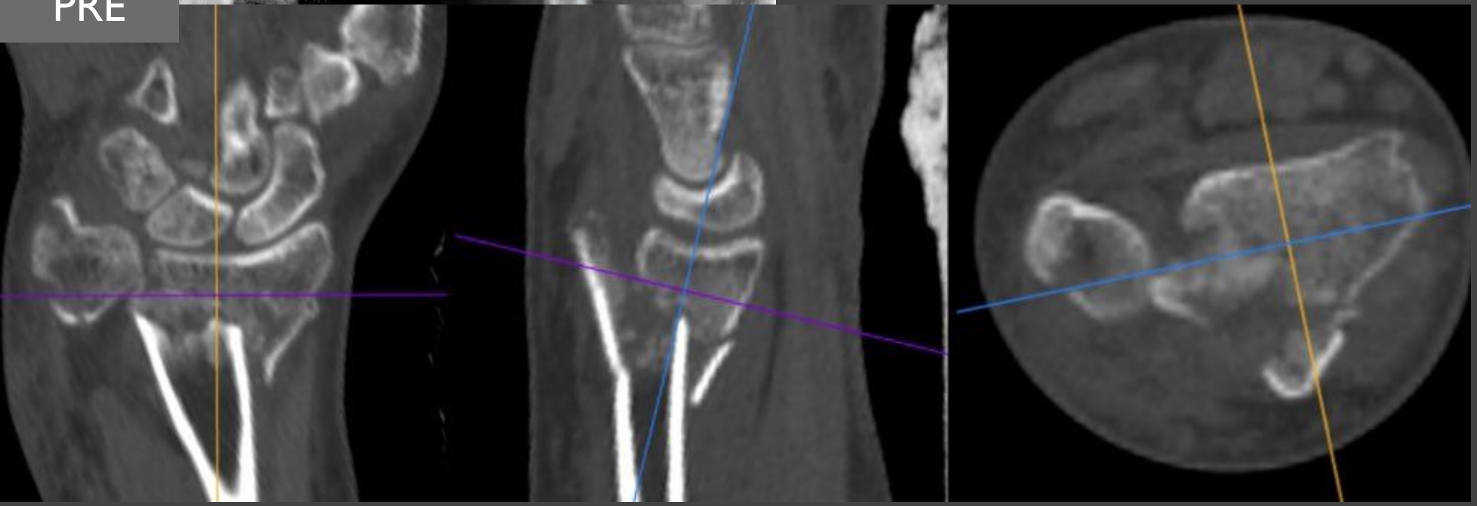

0w

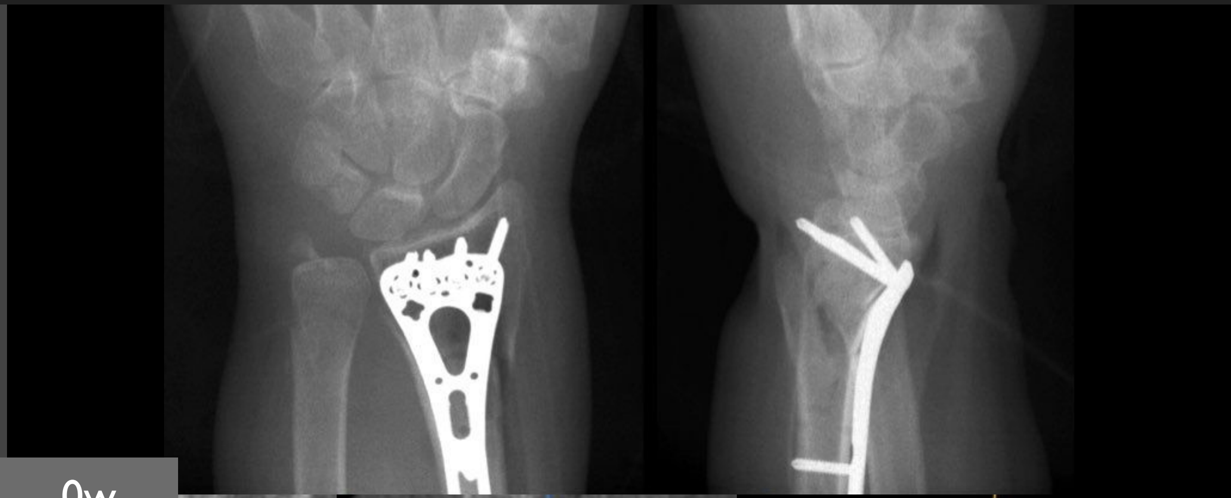

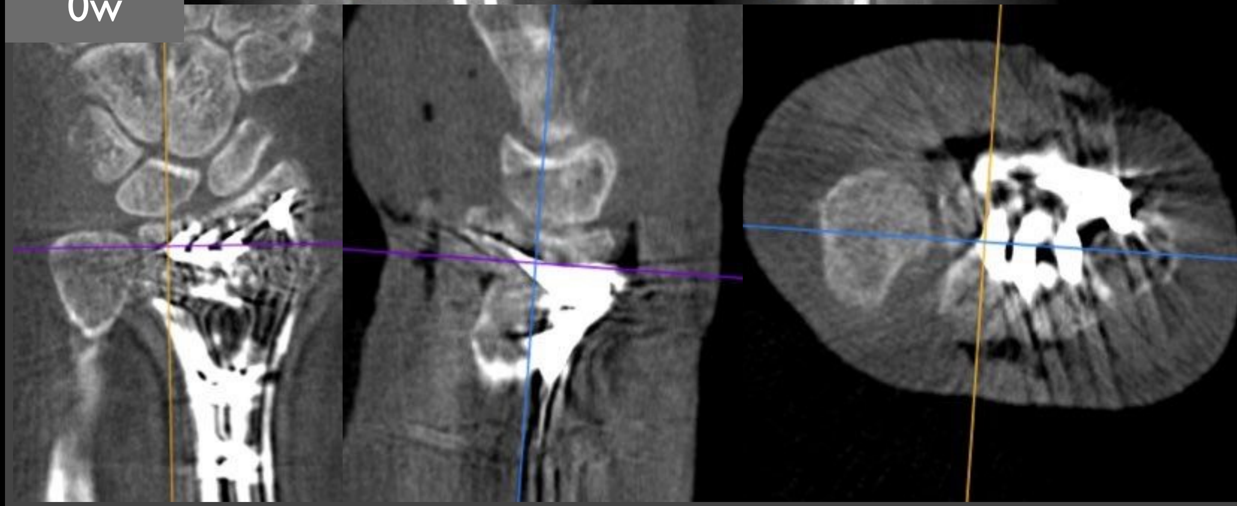

Follow-up

ICUC Score at 143w Functional limitation: 0 Pain: 0

Quick DASH = 0
