# Supplementary material for: The association of ICUC trauma score and quick DASH in a distal radius fracture cohort
Source: J Orthop Surg Res. 2024 Feb 15;19:141. doi: 10.1186/s13018-024-04623-0 (PMC10870621; doi:10.1186/s13018-024-04623-0)
Supplement: Supplementary file 2 — Additional file 2. Sequential Outcome of General Linear Model Utilizing Backward Elimination to Assess Quick DASH Score Influenced by Age, Sex, and ICUC Trauma Score. [file 13018_2024_4623_MOESM2_ESM.pdf]

Supplemental digital content

Method: We ran a General Linear Model (GLM) with backward elimination with the dependent variable Quick DASH and an initial predictor set of ICUC, sex, age at surgery (linear & quadratic terms), and the interaction of ICUC trauma score with sex and with age at surgery (linear, quadratic). Residuals were checked for conformance to normality assumptions.

Results: The final model showed only the interaction of ICUC X linear age at Surgery as significant, whereby the relation of ICUC trauma score to Quick DASH score increased to a more positive relation as age increased. Residuals reasonably conformed to normality assumptions.

```
          GLM with Backward Elimination (p=0.05 Cutoff) of qDASH          1
 Beginning with Predictors: Sex, Age_Surgery(linear & quadratic), ICUC,
  & Interactions: Sex X ICUC & Age_Surgery(linear,quadratic) X ICUC

           Data Set                                  WORK.TRAM
           Dependent Variable                            qDASH
           Selection Method                           Backward
           Select Criterion                 Significance Level
           Stop Criterion                   Significance Level
           Stay Significance Level (SLS)                 0.059
           Effect Hierarchy Enforced                    Single


                 Number of Observations Read          81
                 Number of Observations Used          81


                         Class Level Information

                       Class      Levels    Values

                       sex             2    F M


                                Dimensions

                        Number of Effects       8
                        Number of Parameters    10
```

```
          GLM with Backward Elimination (p=0.05 Cutoff) of qDASH          2
 Beginning with Predictors: Sex, Age_Surgery(linear & quadratic), ICUC,
  & Interactions: Sex X ICUC &  Age_Surgery (linear,quadratic) X ICUC
```

Backward Selection Summary

Step	**Effect Removed**	Number Effects In	Number Parms In	F Value	Pr > F
0		8	8		
1	age_su*age_su*ICUC	7	7	0.89	0.3490
2	age_surge*age_surger	6	6	0.00	0.9452
3	ICUC*sex	5	5	2.69	0.1057

4	sex	4	4	0.00	0.9561

Selection stopped because the next candidate for removal has SLS < 0.059.

Stop Details

Candidate For	Effect	Candidate Significance		Compare Significance	
Removal	age_surgery*ICUC	0.0120	<	0.0590	(SLS)

GLM with Backward Elimination (p=0.05 Cutoff) of qDASH 3
Beginning with Predictors: Sex, Age_Surgery(linear & quadratic), ICUC,
& Interactions: Sex X ICUC & Age_Surgery(linear,quadratic) X ICUC

Selected Model

The selected model is the model at the last step (Step 4).

Effects: Intercept **age_surgery ICUC age_surgery*ICUC**

NOTE: The p-values for parameters and effects are not adjusted for the fact that the terms in the model have been selected and so are generally liberal.

Analysis of Variance

Source	DF	Sum of Squares	Mean Square	F Value	Pr > F
Model	3	4791.31271	1597.10424	35.48	<.0001
Error	72	3240.78769	45.01094		
Corrected Total	75	8032.10039			

Root MSE	6.70902
Dependent Mean	6.06974
R-Square	0.5965
Adj R-Sq	0.5797
AIC	371.21571
AICC	372.07286
SBC	302.53865

GLM with Backward Elimination (p=0.05 Cutoff) of qDASH 4
Beginning with Predictors: Sex, Age_Surgery(linear & quadratic), ICUC,
& Interactions: Sex X ICUC & Age_Surgery(linear,quadratic) X ICUC
Selected Model

Parameter Estimates

Parameter	DF	Estimate	Standard Error	t Value	Pr > |t|
Intercept	1	1.936649	3.595129	0.54	0.5918
age_surgery	1	-0.017947	0.062182	-0.29	0.7737
ICUC	1	-3.853916	3.503771	-1.10	0.2750
age_surgery*ICUC	1	0.194202	0.057700	3.37	0.0012

FINAL SELECTED MODEL 10

"ETA"=% Variance in SAMPLE. "OMEGA"=Unbiased Estimate % Variance in POPULATION.
"SemiPartial"=Total Dependent Variable."Partial"=Covariate Adjusted Dep Variable

Number of Observations Read	76
Number of Observations Used	76

FINAL SELECTED MODEL 5

"ETA"=% Variance in SAMPLE. "OMEGA"=Unbiased Estimate % Variance in POPULATION.
"SemiPartial"=Total Dependent Variable."Partial"=Covariate Adjusted Dep Variable

Dependent Variable: qDASH

Source	DF	Sum of Squares	Mean Square	F Value	Pr > F
Model	3	4791.31271	1597.10424	35.48	<.0001
Error	72	3240.78769	45.01094		
Corrected Total	75	8032.10039			

R-Square	Coeff Var	Root MSE	qDASH Mean
0.596521	110.5323	6.709019	6.069737

Proportion of Variation Accounted for

Eta-Square	0.60
Omega-Square	0.58
95% Confidence Limits	(0.43,0.68)

Source	DF	Type III SS	Mean Square	F Value	Pr > F
age_surgery	1	3.7495561	3.7495561	0.08	0.7737
ICUC	1	54.4566776	54.4566776	1.21	0.2750
age_surgery*ICUC	1	509.8824333	509.8824333	11.33	**0.0012**

"ETA"=% Variance in SAMPLE. "OMEGA"=Unbiased Estimate % Variance in POPULATION.
"SemiPartial"=Total Dependent Variable."Partial"=Covariate Adjusted Dep Variable

Dependent Variable: qDASH

Total Variation Accounted For

Source	Semipartial Eta-Square	Semipartial Omega-Square	Conservative 95% Confidence Limits	
age_surgery	0.0005	-0.0051	0.0000	0.0450
ICUC	0.0068	-0.0012	0.0000	0.0855
age_surgery*ICUC	0.0576	0.0576	0.0000	0.1902

Partial Variation Accounted For

Source	Partial Eta-Square	Partial Omega-Square	95% Confidence Limits	
age_surgery	0.0012	-0.0122	0.0000	0.0563
ICUC	0.0165	0.0028	0.0000	0.1099
age_surgery*ICUC	0.1359	0.1359	0.0013	0.2766

Parameter	Estimate	Standard Error	t Value	Pr > |t|
Intercept	1.936648862	3.59512917	0.54	0.5918
age_surgery	-0.017947062	0.06218168	-0.29	0.7737
ICUC	-3.076720661	3.50377070	-1.10	0.2750
age_surgery*ICUC	**0.169047556**	**0.05770032**	**3.37**	**0.0012**

Parameter	95% Confidence Limits	
Intercept	-5.230110163	9.103407888
age_surgery	-0.141903980	0.106009857
ICUC	-10.83855490	3.130723585
age_surgery*ICUC	0.079178742	0.309225718

The interaction of age at surgery x ICUC trauma score is significant. This means the relation of ICUC trauma score to Quick DASHS varies depending on age. Conversely, less intuitively, it can be interpreted to mean that the relation of age to Quick DASH varies depending on the ICUC trauma score.
